# Effectiveness of Multiple-Strategy Community Intervention in Reducing Geographical, Socioeconomic and Gender Based Inequalities in Maternal and Child Health Outcomes in Haryana, India

**DOI:** 10.1371/journal.pone.0150537

**Published:** 2016-03-22

**Authors:** Madhu Gupta, Federica Angeli, Hans Bosma, Monica Rana, Shankar Prinja, Rajesh Kumar, Onno C. P. van Schayck

**Affiliations:** 1 Department of Community Medicine, School of Public Health, Post Graduate Institute of Medical Education and Research, Chandigarh, India; 2 Department of Health Services Research, School of Public Health and Primary Care (CAPHRI), Maastricht University, Maastricht, The Netherlands; 3 Department of Social Medicine, School of Public Health and Primary Care (CAPHRI), Maastricht University, Maastricht, The Netherlands; 4 Department of Family Practice, School of Public Health and Primary Care (CAPHRI), Maastricht University, Maastricht, The Netherlands; Oswaldo Cruz Foundation, BRAZIL

## Abstract

**Objective:**

The implemented multiple-strategy community intervention National Rural Health Mission (NRHM) between 2005 and 2012 aimed to reduce maternal and child health (MCH) inequalities across geographical, socioeconomic and gender categories in India. The objective of this study is to quantify the extent of reduction in these inequalities pre- and post-NRHM in Haryana, North India.

**Methods:**

Data of district-level household surveys (DLHS) held before (2002–04), during (2007–08), and after (2012–13) the implementation of NRHM has been used. Geographical, socioeconomic and gender inequalities in maternal and child health were assessed by estimating the absolute differences in MCH indicators between urban and rural areas, between the most advantaged and least advantaged socioeconomic groups and between male and female children. Logistic regression analyses were done to observe significant differences in these inequalities between 2005 and 2012.

**Results:**

There were significant improvements in all MCH indicators (p<0.05). The geographical and socioeconomic differences between urban and rural areas, and between rich and poor were significantly (p<0.05) reduced for pregnant women who had an institutional delivery (geographical difference declining from 22% to 7.6%; socioeconomic from 48.2% to 13%), post-natal care within 2 weeks of delivery (2.8% to 1.5%; 30.3% to 7%); and for children with full vaccination (10% to 3.5%, 48.3% to 14%) and who received oral rehydration solution (ORS) for diarrhea (11% to -2.2%; 41% to 5%). Inequalities between male and female children were significantly (p<0.05) reversed for full immunization (5.7% to -0.6%) and BCG immunization (1.9 to -0.9 points), and a significant (p<0.05) decrease was observed for oral polio vaccine (4.0% to 0%) and measles vaccine (4.2% to 0.1%).

**Conclusions:**

The implemented multiple-strategy community intervention National Rural Health Mission (NRHM) between 2005 and 2012 might have resulted in significant reductions in geographical, socioeconomic and gender inequalities in MCH in Haryana, as causal relationships cannot be established with descriptive research.

## Introduction

Maternal and child health (MCH) inequalities across socioeconomic, geographical and gender gradient is a public health concern worldwide [1,2]. This gap is much more widened in the low and middle-income group countries because of unequal distribution of resources and health facilities. In a retrospective review done by using demographic health surveillance data from 54 countries, marked MCH inequalities in low and middle-income group countries including India were observed [3,4]. In India, the maternal mortality ratio (MMR) is still as high as 167 per hundred thousand live births [5] and the infant mortality rate (IMR) is 40 deaths per thousand live births [6]. There is geographical inequality in MCH outcomes, like IMR is higher in rural (44 per thousand live births) as compared to urban areas (27 per thousand live births) [6]. Large geographical and socioeconomic inequalities in MCH status and access to MCH services continue to persist in India and have even widened across states, between rural and urban areas, and within communities [7]. There are MCH interventions available to improve MCH outcomes and reduce inequalities, however because of the issues at the level of implementation of these interventions that influence the accessibility and availability of health services especially to the most needy, these inequalities are not reduced. [8–10].

This persistence of MCH inequalities indicates the need to assess how the existing national health programs or policies on MCH are being implemented to tackle this issue in India. Simultaneously, it indicates the need for studies presenting evidence on the effectiveness of these programs, as these are highly resource-intensive interventions. Such assessments are useful for policy makers in resource constraint country like India in changing the policy or implementation strategy of these interventions.

Indian government had launched a multiple-strategy community intervention to reduce MCH inequalities across socioeconomic, geographical and gender gradient known as the National Rural Health Mission (NRHM). It was started in 2005 in the 11^th^ health plan (2005 to 2012), and continued in 12^th^ health plan (2013 to 2017)] as National Health Mission at national level. The aim was to reduce health inequalities by improving the availability of and access to better-quality healthcare, especially for people residing in rural areas (to reduce geographical inequality), for the poor (to reduce socioeconomic inequality), and for women and children (to reduce gender inequality) [11]. NRHM’s health sector plans included health system strengthening, specific MCH strategies/schemes [under reproductive and child health program (RCH-II)], and communitization (delegating powers to and empowering the community to monitor the healthcare delivery system) [12]. Details of these plans are given in previously published study protocol [13].

Briefly, health system strengthening included strengthening the health infrastructure, providing free drugs and logistics and telemedicine facilities, availability of mobile medical units and patient transport services. MCH schemes included cash incentives for hospital deliveries [*Janani Suraksha Yojna*], free delivery services for pregnant women and treatment of neonatal illnesses in hospitals and reimbursements of travel cost to hospitals [*Janani Shishu Suraksha Karyakaram*], and appointing Accredited Social Health Activists (ASHAs) to promote the access to improved healthcare at household level in villages. The intention was to reduce the infant mortality rate to 30/1,000 live births, and maternal mortality to 1/1,000 live births.

The objective of this study is to quantify the extent of reduction in MCH inequality across geographical, socioeconomic and gender sectors by using the data of demographic health surveys, held pre (2003–04), during (2007–08) and post (2012–13) NRHM implementation in Haryana, North India. The added value of looking at the data during the implementation (District level Household Survey—3, 2007–08) is to have information on the trend of MCH status and inequalities, since the implementation of NRHM health sector plans was gradual in the state. Referral transport services, human resources, drugs and logistics, ASHA scheme, and immunization were fully implemented, while all other schemes were only partially implemented. It took these schemes between 4 to 5 years to achieve full implementation [14]. The results of this study would inform the policy makers on the NRHM’s plans that have been most effective in meeting its MCH goals. Funds could accordingly be allocated to those plans that have demonstrated highest adoption and effectiveness or conversely be directed to those that have suffered under-funding but are likely to be successful with proper financial support during the implementation of the second stage of the NRHM (2013–17).

## Materials and Methods

### Ethics Statement

Authors had received the ethical approval from Post Graduate Institute of Medical Education and Research (PGIMER), Chandigarh, India, to conduct this study.

### Setting

The state chosen for this study was Haryana in North India, as it is representative of other North Indian states with similar socioeconomic development and sociocultural factors, such as the preference to have sons, female feticide, lower sex ratios and lower social status of women. At the same time, Haryana represents a unique context by being a prosperous state with a rising economy but with unequal distribution of resources, which has led to wide intra-state and inter-district differences. Despite being one of the richer states, reporting the highest per capita income in the country at Rs 1,09,064 (USD 1947.6) during 2012–13, MCH indicators are not the best in the country [15]. The state is divided into 21 districts, with a total population of 2,53,53,081 (70% rural), reports a birth rate of 21.3, a death rate of 6.3 per thousand mid-year population, and a total fertility rate of 2.3 [5,6]. The health care delivery system has been described in detail in previous protocol study [16].

### Data and Sample

The source of data in this study was nationally representative demographic health survey known as District Level Household Survey (DLHS). DLHS provides consistent and reliable estimates of status of maternal health including antenatal care (ANC), natal and post natal care (PNC); child health care including immunization status; and utilization of MCH services at district level [17]. It also provides information on status of MCH services at different health facilities including sub-centre (SC), primary health center (PHC), community health center (CHC) and district hospital (DH). Till now four rounds of DLHS has been conducted by Ministry of Health and Family Welfare, Government of India though an external agency (International Institute for Population Sciences). Surveys were implemented through regional agencies by appointing a team of five persons, consisting of one supervisor, one field editor and three female investigators, who were at least graduates.

Because the second round (DLHS-2) was conducted during the year 2002–04, the third round (DLHS-3) in 2007–08 and the fourth round (DLHS-4) in 2012–13, the three waves of data collection reflected the situation before, during and after NRHM implementation, respectively. Hence, all data of these rounds of surveys were included in this study for analysis. DLHS-2 provided information on how well RCH-II program was performing, DLHS-3 on health facility’s capacity and preparedness in terms of infrastructure when NRHM was being implemented so as to take corrective measures, and DLHS-4 on achievements and improvements after seven years of implementation of NRHM.

Detailed methodology of these surveys has been given in DLHS 2 (S1 File), 3 (S2 File) and 4 (S3 File) reports [18–21]. Briefly, a multi-stage stratified systematic sampling design was adopted in all the rounds to select 50 primary sampling units, which were census villages in rural areas and census enumeration blocks in urban areas in each district. Data was collected by using pretested structured questionnaires, namely, household, ever married woman, village and facility, which were typed in bilingual languages (regional and English). The same core sets of questionnaires were used in each survey so that comparisons could be drawn. The facility survey was conducted during DLHS-3 and 4 rounds only. Method of data collection was interview. Taking account of the multi-stage stratified systematic sampling, the DLHS researchers had applied weights to estimate the percentages of the MCH indicators. The percentages are then representative for the respective populations in Haryana in the different periods.

Background information of households during three surveys is given in S1 Table. Data was collected from 18,796, 20,394, and 27,414 currently married women aged 15–49 years during DLHS 2 (aged 15–44 years), 3 and 4, respectively. About 990 and 1,046 health facilities were visited for facility survey during DLHS 3 and DLHS 4, respectively. The response rate varied from 85% to 95% for households and married women, respectively in all the surveys.

In addition to demographic health survey data, data of concurrent evaluation of NRHM in Haryana (held during 2012–13) was used for obtaining wealth quintile wise information on MCH indicators post NRHM, as this was unavailable in DLHS-4 report. (S4 File). Data of concurrent evaluation study was collected from 18,227 currently married women aged 15–49 years quarterly in all the districts in Haryana by School of Public health, PGIMER, post-NRHM, by using the same methodology as was used in DLHS, hence results were comparable [22]. The difference in concurrent study and DLHS is that data was collected on regular basis in concurrent study, while once in DLHS.

### Measures and Data Analysis

List of independent and dependent variables considered in this study is given in S2 Table. Independent variables were socioeconomic variables i.e., standard of living (DLHS 2) / wealth index (DLHS 3), place of residence (rural or urban) and gender of the child (male or female), as available from survey data. Standard of living index is a composite measure that was computed for classifying the households into low, medium and high standard on the basis of scores during DLHS 2. Scores were given after considering household amenities such as source of drinking water, type of house, source of lighting, fuel for cooking, toilet facility and ownership of durable goods. Wealth index is computed by combining the household amenities, assets and durables at the national level and then dividing into quintiles (lowest, second, middle, fourth and highest). It was computed in DLHS 3 and in concurrent evaluation study.

From the list of various MCH indicators tracer indicators (dependent variables) were selected for the respective MCH situation, so as to have information on these selected indicators across geographical, socioeconomic and gender gradient. Tracer indicators were selected from the larger list because these were key indicators representative of each major aspect of maternal care and child preventive and treatment interventions. These indicators were similar to the ones chosen for tracking the progress for maternal, newborn and child survival as part of countdown to 2015 to meet the MCH millennium development goals for India [23]. Hence for maternal health, antenatal care indicators were: pregnant women who had three or more antenatal check ups, received two tetanus toxoid (TT) injection, consumed 100 iron folic acid (IFA) tablets, received full antenatal check-up. Natal care indicator was institutional delivery, while post-natal care indicator was post-natal check-up within two weeks of delivery. For child health, indicators were full immunization of a children aged between 12–23 months and children who received oral rehydration solution (ORS) for diarrhea management. Information on indicators on the distance and availability of health services for MCH was also obtained. Information on MMR and IMR were available at the state level and obtained from the Sample Registration System. [5,6]. Information on MMR was not available across geographical or socioeconomic gradient, while IMR was available across geographical gradient from Sample Registration System [6].

Geographical, socioeconomic, and gender inequality in MCH was assessed by estimating the absolute differences (range) in MCH indicators between urban and rural areas, between the most advantaged and least advantaged socioeconomic groups (excluding maternal and child mortality indicators), and between male and female children.

Data was analyzed using Microsoft Excel and Statistical Package for Social Sciences (SPSS) version 16. Individual data of rounds 2 and 3 and aggregated data of round 4 of DLHS and concurrent evaluation study were used for analysis. Outcome variables and inequality measures were compared before, during and after the introduction of the NRHM, from 2002–04 to 2012–13, to assess improvements in MCH outcomes and inequalities in Haryana. Since the NRHM is implemented in all areas in Haryana, the situation during the pre-NRHM implementation period served as a control. It is expected that rate difference score either decreased or reached near to 0 post NHRM. The unweighted numbers were available for the MCH indicators in each group (rural/urban, poor/rich, male/female) and for each respective time period (pre, during and post NRHM). The reported weighted percentages and the unweighted numbers allowed the reconstruction of cross-tabulations (e.g. rural-urban differences regarding three or more antenatal care visits) for the three separate periods. Chi^2^ tests then indicated whether there was a significant difference (e.g. rural-urban) within each period. Testing the statistical significance of the interaction between the inequality measures (e.g. urban-rural) and period (2002–2004, 2007–2008, and 2012–2013), logistic regression analyses indicated whether these differences changed across time (e.g. whether the urban-rural difference decreased between 2002–2004 and 2012–2013). A p-value of 0.05 was used.

## Results

Overall improvements were observed in proportion of literate population of age 7 years and above from 70.9% to 77.7%, currently married women with 10 or more years of schooling from 24.5% to 37.5%, households with electricity from 91.2% to 97.7%, access to improved toilet facilities from 48.7% to 83.9%, wealth index from 19.3% in low rank to 9% in low & second quintile. Nearly 70% of women were belonging to rural area and 90% of the households to Hindu religion. (S1 Table).

### Maternal and child health outcomes

Status of MCH indicators pre, during and post NRHM implementation in Haryana as per DLHS rounds 2, 3 and 4 is given in S3 Table. Reference period for obtaining the MCH related history was three years preceding the respective surveys. The proportions of pregnant women having three or more ANCs increased significantly (p<0.05) from 43% to 74.5%, at least one TT injection from 83.5% to 93.6%, institutional delivery from 35.7% to 77%, PNC within 2 weeks of delivery from 49% to 67.2%; and children who received ORS for diarrhea from 32.3 to 44.8%. MMR although had declined from 1.85 per thousand live births (2002–04) to 1.21 per thousand live births (2012–13) at state level, yet the decline was not significant. IMR had declined significantly (p<0.05) from 59 to 40 per thousand live births.

### Geographical inequalities

The difference (Diff) between MCH indicators in urban and rural areas pre, during and post NRHM implementation as per DLHS rounds 2, 3 and 4 is given in Table 1. Significant (p<0.05) decline in difference of MCH indicators between urban and rural areas was observed for proportion of pregnant women who had three ANCs from 23% to 5.4%, full ANC check ups from 8% to 6.8%, institutional delivery from 22% to 7.6%, PNC with in 2 weeks of delivery from 2.8% to 1.5%; children who received full vaccination (children age 12–23 months who have received all the primary vaccines i.e., Bacillus Calmitte Guerin (BCG) vaccine for tuberculosis; 3 doses of Diptheria pertussis and tetatnus vaccine (DPT), 3 doses of Oral Polio Vaccine (OPV) and measles vaccine) from 10% to 3.5% and ORS for diarrhea from 11% to -2.2%. P value shown in Table 1 indicates statistically significant difference in geographical inequality across time periods. The difference in urban and rural areas for proportion of pregnant women who had PNC with in two weeks of delivery, children with full vaccination and who received ORS became non significant in the post NRHM period.

**Table 1:** Geographical inequalities in maternal and child health indicators in rural and urban area during pre, during and post NRHM implementation (expressed as absolute difference in proportion of indicators in urban and rural area).

Indicators	Pre NRHM (2002–2004)			During NRHM (2007–2008)			Post NRHM (2012–2013)			P value
	Rural	Urban	Diff	Rural	Urban	Diff	Rural	Urban	Diff	
**Maternal Health: Women who had (%)**										
Three or more ANC	40.8	63.9	23*	47.2	66.1	18.9*	72.4	77.8	5.4*	0.00
Full ANC check up	9.5	17.9	8*	10.2	22.6	12.4*	19.2	26.0	6.8*	0.00
Received two TT injections	75	84.3	9.3*	76.9	87	10.1*	54.4	65.2	10.8*	0.02
Consumed IFA for at least 3 months	15.9	21.6	6*	28.1	31.7	3.6*	27.6	32.6	5*	0.08
Institutional delivery rate	27.3	56.4	29*	42.2	61.4	19.2*	74.3	81.9	7.6*	0.00
PNC with in 2 weeks of delivery	9.6	6.8	2.8*	46.5	58.7	12.2*	68.5	70.0	1.5	0.00
**Child Health (%)**										
Children age 12–23 months who received Full vaccination	56.7	66.3	10*	55.9	70.8	14.9*	51.0	54.5	3.5	0.00
Children with diarrhoea who received ORS	29	40	11*	28.3	44.2	15.9*	45.6	43.4	-2.2	0.01
Infant Mortality Rate (per thousand live births)	66	47	19*	60	44	16*	44	32	12*	0.09

*p<0.05

### Socioeconomic inequalities

The difference between rich and poor was observed to be significantly (p<0.05) narrowed down for proportion of pregnant women who received 2 TT injection from 30.3% to 7%, institutional deliveries from 48.2% to 13%, fully immunized children from 48.3% to 14 points%, proportion of children who received ORS for diarrhea from 41% to 5%, in pre to post NRHM period. (Table 2). Although pregnant women who had three or more ANCs increased considerably among the least (16.8% to 57%) and the highest socioeconomic group (17% to 80%), yet the improvement in this particular indicator was much more among those in the highest wealth quintile, resulting in increase in inequalities between lowest and highest wealth quintile groups post- NRHM implementation from 0.2% to 23%. P value shown in Table 2 indicates statistically significant difference in the socioeconomic inequality across time periods.

**Table 2:** Trend of socioeconomic inequalities across various maternal and child health indicators in Haryana from pre to post NRHM.

Maternal and Child Health Indicators (%)	Pre NRHM (2002–04)			During NRHM (2007–08)				Post NRHM (2012–13)			P value
	Standard of living index			Wealth Index				Wealth Index			
	Low	High	Diff	Lowest	Highest		Diff	Lowest	Highest	Diff	
**Maternal Health: Women who had (%)**											
Three or more ANC	16.8	17	0.2	16.3	72.4		46.5*	57	80	23*	0.00
Full ANC check up	3.6	20.1	16.5*	1.4	23.5		20.1*	20	36	16*	0.00
Received two TT injections	58.5	88.5	30.3*	48.5	91.8		35.6*	77	84	7*	0.00
Consumed IFA for at least 3 months	8.5	25.4	16.9*	2.2	28.1		22.3*	30	44	14*	0.00
Institutional delivery rate	11.8	60	48.2*	14.8	70.9		52.5*	75	88	13*	0.00
PNC with in 2 weeks of delivery	6.4	8.6	2.2*	26.9	66.7		39.8*	55.4	72.1	16.7*	0.00
**Child Health (%)**											
Children (age 12–23 months) who received Full vaccination	30.6	78.9	48.3*	28.5		74.4	44.4*	62	76	14*	0.00
Children with diarrhoea who received ORS	7.8	48.5	40.7*	10.3		44.7	34.4*	32	37	5	0.00

*p<0.05

### Gender inequalities

Inequalities between male and female children was significantly (p<0.05) reversed for full immunization (5.7% to -0.6%) and BCG immunization (1.9 to -0.9 points), and a significant (p<0.05) decrease was observed for oral polio vaccine (4.0% to 0%) and measles vaccine (4.2% to 0.1%). (Table 3). P value shown in Table 3 indicates statistically significant difference in gender inequality across time periods.

**Table 3:** Child based gender inequalities in Haryana (expressed as absolute difference in proportion of indicators among male and female children).

Indicators (%)	Pre NRHM (2002–04)			During NRHM (2007–08)			Post NRHM (2012–13)			P value
	Male	Female	Diff	Male	Female	Diff	Male	Female	Diff	
	n = 1221	n = 993		n = 1178	n = 947		n = 1142	n = 896		
**Children aged 12–23 months who received (%)**										
Full Immunization	61.8	56.1	5.7*	62.5	56.0	6.5*	51.9	52.5	-0.6	0.00
No Vaccination	11.3	12.7	-1.4	1.7	2.1	-0.4*	6.5	6.8	-0.3	0.00
BCG vaccine	84.3	82.4	1.9	87.8	84.9	2.9*	83.8	84.7	-0.9	0.00
3 doses DPT vaccine	75.6	70.7	4.9	71.7	65.8	5.9*	72.2	73.5	-1.3	0.00
3 doses of OPV vaccine	74.7	70.1	4.6*	70.2	64.7	4.6*	71.1	71.1	0	0.00
Measles vaccine	67.6	62.2	4.2*	70.9	66.7	5.4*	69.8	69.7	0.1	0.00

*p<0.05

Trend of availability and accessibility of health facilities from 2007–08 to 2012–13 in Haryana is presented in S4 Table. Percentage of villages having ASHA’s increased from 81% to 96%. Accessibility of health facilities improved with proportion of villages with SCs center with in 3 km increased from 77% to 80.4% and PHCs with in 10 km from 82.3% to 87.3%. Increase in availability of services observed for SCs with additional ANM (74.2% to 83.5%); PHCs functioning on 24x7 hours basis (39% to 79.3%), having referral services for pregnancies/delivery on 24x7 hours basis (47% to 65.6%), conducting at least 10 deliveries during last month on 24x7 hours basis (38.8% to 74.3%); Community health centers (CHCs) having 24x7 hours normal delivery (88% to 100%), designated as first referral units (44% to 72%), having 24x7 new born care services (62.2% to 91.5%); District hospitals having pediatrician (77.7% to 95.2%), ultrasound facility (90.5%), critical care area (44.4% to 76%).

## Discussion

The results of the present study have highlighted that overall there is improvement in the broader social determinants of MCH (access to safe drinking water, sanitation facilities and clean fuel for cooking; literacy level of women), MCH coverage indicators, mortality statistics (maternal mortality ratio and infant mortality rate), and also reduction in geographical, socioeconomic and gender inequality, when compared with the situation before and after NRHM implementation. There is also improvement in accessibility and availability of health facilities after NRHM implementation. However, the coverage of MCH indicators and availability of health facilities is not yet 100%. This indicates that the aim with which the NRHM was implemented i.e., to reduce MCH inequalities by improving the availability of and access to better-quality healthcare, especially for people residing in rural areas (to reduce geographical inequality), for the poor (to reduce socioeconomic inequality), and for women and children (to reduce gender inequality) has been achieved to some extent but not fully. That is why probably the goal to reduce the infant mortality rate to 30/1,000 live births, maternal mortality to 1/1,000 live births could not be achieved till 2012–13.

Earlier studies that have used demographic health surveillance data in India to report MCH inequalities have shown marked inequalities during antenatal period, postnatal period and natal period regarding skilled birth attendance, which concentrated disproportionately among the rich [24–26]; and higher malnutrition burden among poor children coupled with a concomitant rise in economic inequalities [27]. Gender disparity in immunization programs favoring males has been reported in urban areas, developed states and Muslim communities in India [28]. Poor household economic status (46%), mother's illiteracy (35%) and rural residence (15%) contributed to 96% of total socio-economic inequalities in child survival at the national level [29]. In addition to these factors mass media exposure were the critical pathways reported through which economic factors operate on MCH inequalities [30]. However, most of the earlier studies reflect the situation before NRHM implementation, which is similar to the situation observed during that period in this study also (2002–04). The strength of this study is that it documents the trend of MCH inequalities across geographical, socioeconomic and gender sectors in three time periods (pre-, during- and post-NRHM), so as to have better understanding of the dynamics of MCH inequalities and effectiveness of NRHM in reducing those.

Sanneving et al (2013) reviewed literature to use framework developed by Commission on social determinants of health to categorize and explain determinants of inequity in maternal and reproductive health in India, and concluded that economic status, gender, and social status are all closely interrelated when influencing use of and access to maternal and reproductive health care [31]. Therefore we had chosen to specifically to look into the status of these inequalities pre, during and post NRHM implementation.

Regarding the trend in MCH inequality in this study, it was observed that the initially MCH coverage indicators improved irrespective of area (rural or urban) or socioeconomic status (standard of living/wealth index) or gender (for children) after the launch of NRHM in the state. Since, in urban areas and among women who were at advantage due to their economic status or children due to being male, MCH indicators were better at the baseline (2002–04) as compared to their counterparts, hence MCH inequalities across these gradients increased during 2007–08. However, we witnessed that this gap reduced when MCH status reassessed during 2012–13.

This reduction of gap suggests that concerted efforts were done especially in rural areas and for the poor women to improve the MCH service coverage among them, probably after 2007–08. These efforts are reflected in the facility survey data (collected during DLHS 3 and 4), in the form of increased availability of ASHA’s in almost all the villages, additional ANM in the SCs and accessibility of health facilities like sub centers with in 3 km and PHCs with in 10 km of villages. Additionally, availability of round the clock services at PHC level increased considerably. Since functional services at PHC’s were provided close to villages, there was increase in proportion of PHCs with at least 10 deliveries in a month. This indirectly indicates increased utilization of functional services at PHCs in rural areas. Provisions of round the clock delivery and newborn care services also improved at CHC level (first referral units). This made the referral of complicated pregnancies/deliveries manageable. One possible reason for the noted coverage increases could be the simultaneous increase in the number and type of health facilities as shown by the data. High coverage of institutional delivery care with significant financial risk protection in the public sector has also been reported in a recent study in Haryana [32]. All these efforts jointly may have resulted in improvement in MCH indicators especially in rural areas and among least advantaged women and decline in geographical and socioeconomic inequalities post NRHM.

The present study probably the first one to document that there is bridging of MCH inequalities across geographical area, socioeconomic and gender gradient after NRHM implementation. Some of the indicators are even better in rural areas as compared to urban area like receiving ORS for diarrhea, and immunization among female children during 2012–13. In the time period covered (including the introduction of the NRHM), inequalities in MCH indicators may have decreased, but time-dependent changes (other than the introduction of the NRHM) may have occurred simultaneously (e.g. decreased income inequality, increased gross domestic product or other policies/regulations). We acknowledge that the inequalities improved in Haryana, but that more research is needed to know whether this is due to NRHM or a general economic progress in the background (improving everything including food and hygiene/sanitary situation, but also giving the country the possibility of implementing NRHM) in the same period. Moreover, causal relationships can never by fully proven with descriptive research. According to Mckeown, the improvements in the health indicators are less due to the human agency in the form of health-enhancing measures than to largely invisible economic forces that changed broad social conditions, that needs further exploration in this situation [33].

However, alternate explanations for reduction in inequalities may not account for, if we consider the logical framework analysis approach (input-process-output-outcome-impact analysis) for assessing the effectiveness of an intervention [34]. By applying this approach to explain the results of this study, it has been observed that after implementation of NRHM there has been considerable increase in inputs and processes (which has been presented in S4 Table) that provides the causal link in improving the output in terms of improved MCH coverage indicators (S3 Table) and ultimately outcomes and impact in terms of reduction in MCH inequalities and mortality (Tables 1–3). Evaluation of the program/intervention by comprehensively measuring the inputs, processes, outputs, outcomes, and impact over a longer time horizon is considered as a best available option when there is no control population for establishing the cause and effect relationship, as is the case in the present study [32]. WHO commission on social determinants of health does recognize the role of health systems in reducing inequalities as intermediary determinant [35]. Hence it can be stated that NRHM perhaps played a role through influencing health system in terms of improving access of MCH services, decreasing differential vulnerability and exposure to impact MCH inequalities.

During our analysis, we used absolute differences to measure the inequalities, which is considered more informative and useful to plan future interventions to reduce inequalities [36]. However, additional logistic regression analyses looked at the inequalities from a relative perspective, which added to the absolute perspective. The DLHS percentages indicated a general trend of reducing inequalities in Haryana. The logistic regression analyses were based on reconstructed cross-tabulations, using unweighted total numbers and weighted percentages. The testing for statistical significance (of reducing relative inequalities) should thus be interpreted cautiously. Also data on MMR might be fairly inconclusive because of wide uncertainty around the measure, particularly looking at per 1,000 births. Future studies should be planned after carefully considering these aspects.

The results of this study have important public health implications globally as monitoring inequality is becoming an emerging priority for health post 2015 [37,38]. The post 2015 sustainable development goals stress leaving no one behind and with goal ten focusing on inequality within and among countries. These results also have implications in terms of continuation of the program implementation in the rural areas with a special focus on poor women and children with same rigor in India. As we know that there are political preferences in what should be implemented or not at the national or state level, the program component, which is being implemented successfully, should be continued irrespective of political party in power at that moment [39]. This is quite pertinent to India as NRHM was implemented by the previous government as one of their major thrust area and also as part of commitment to meet Millennium development goals 4 and 5. The present government should take decisions or mend program implementation after carefully considering and deliberating upon what good the existing program has done in reducing maternal and child mortality statistics in future. The results of this study have shown that substantial gains were observed for schemes that aimed at increasing the institutional delivery rate among the poor and in rural areas. These schemes were free referral transport services, free hospital delivery, financial incentives for institutional delivery, improved access to delivery points and availability of ASHAs in the villages. Hence, these schemes should be further strengthened. While schemes aimed at improving child health like integrated management of neonatal and childhood illness needs more attention. We are on right path of improving MCH outcomes along with reduction in MCH geographical and socioeconomic inequalities to some extent, but the pace of achievement needs to be heightened to achieve sustainable development goals post 2015.

## Supporting Information

S1 Table Background information of population and households surveyed and characteristics of women interviewed during DLHS round 2, 3 and 4. (PDF)

S2 Table List of independent and dependent variables. (PDF)

S3 Table Status of maternal and child health indicators pre, during and post NRHM implementation in Haryana as per DLHS rounds 2, 3 and 4. (PDF)

S4 Table Trend of availability and accessibility of health facilities during and after NRHM implementation in Haryana. (PDF)

S1 File District Level Household Survey. Haryana Report. Round 2. 2002–04.
(PDF)

S2 File District Level Household and Facility Survey. Haryana Report. Round 3. 2007–08.
(PDF)

S3 File District Level Household and Facility Survey. Haryana Report. Round 4. 2012–13.
(PDF)

S4 File Concurrent Evaluation of National Health Mission, Haryana. PGIMER School of Public Health, Chandigarh.
(PDF)
