# Supplementary material for: Effectiveness of Multiple-Strategy Community Intervention in Reducing Geographical, Socioeconomic and Gender Based Inequalities in Maternal and Child Health Outcomes in Haryana, India
Source: PLoS One. 2016 Mar 22;11(3):e0150537. doi: 10.1371/journal.pone.0150537 (PMC4803212; doi:10.1371/journal.pone.0150537)
Supplement: S1 Table — (PDF) [file pone.0150537.s001.pdf]

S1 Table. Background information of population and households surveyed and characteristics of women interviewed during DLHS round 2, 3 and 4.

**Indicators**	**Pre NRHM**			**During NRHM**			**Post NRHM**		
	**DLHS-2 (2002-04)**			**DLHS-3 (2007-08)**			**DLHS-4 (2012-13)**		
**Sample Size**	**Total**	**Rural**	**Urban**	**Total**	**Rural**	**Urban**	**Total**	**Rural**	**Urban**
Households surveyed	20,205	13,832	6,373	21,406	15,615	5,791	33,772	19,216	14,556
Currently married women age 15-49 years	18,796	13,307	5,489	20,394	15,306	5,088	27,414	16,093	11,321
**Profile of population**									
Literate age 7+ years (%)	70.9	66.1	81.9	73.4	70.2	82.7	77.7	73.5	85.5
Below age 15 years (%)	12.2	12.4	11.6	32.2	33.1	29.3	26.7	27.7	24.8
Mean household size	5.5	5.6	5.1	5.2	5.4	4.9	4.9	5.1	4.6
Sex ratio at birth (Males per 100 Females)	113	113	113	110	109	112	113	112	114
**Percentage of households**									
Having electricity	91.2	88.2	97.8	92.4	90.5	97.5	97.7	97.2	98.7
Improved source of drinking water	91.7	78.5	98.8	96.0	94.7	99.5	99.1	98.9	99.4

Having access to improved toilet facility	48.7	31.0	87.5	50.7	39.0	82.1	83.9	77.5	94.8
Use clean fuel for cooking	31.9	13.0	73.0	26.5	11.1	68.1	47.4	27.0	82.1
**Education of currently married women aged 15-49 (15-45 years in DLHS 2) years (%)**									
Illiterate	44.6	52.0	26.8	38.8	43.4	24.9	33.9	40.0	23.4
with 10 or more years of schooling	24.5	15.7	45.9	25.9	19.9	44.5	37.5	28.0	53.9
