# Supplementary material for: Effectiveness of Multiple-Strategy Community Intervention in Reducing Geographical, Socioeconomic and Gender Based Inequalities in Maternal and Child Health Outcomes in Haryana, India
Source: PLoS One. 2016 Mar 22;11(3):e0150537. doi: 10.1371/journal.pone.0150537 (PMC4803212; doi:10.1371/journal.pone.0150537)
Supplement: S2 Table — (PDF) [file pone.0150537.s002.pdf]

**S2 Table. List of independent and dependent variables.**

| **Independent Variables** | **Dependent Variables** | |
|---|---|---|
| **Geographical** | **Maternal Health Indicators** | **MCH Tracer indicators** |
| **Type of Locality** | **Antenatal care: Pregnant women who had** | **Antenatal Care** |
| Rural | Registration in the first trimester | Three or more ANC |
| Urban | Three or more ANC | Full ANC check up |
| **Standard of living index/Wealth quintile** | Full ANC check up | Received two TT injections |
| Low/lowest quintile | At least one TT injection | Consumed IFA for at least 3 months |
| Fourth quintile | Received two TT injections | **Natal Care (%)** |
| Middle/Mid quintile | Consumed IFA for at least 3 months | Institutional delivery rate |
| Second quintile | **Natal Care (%)** | **Post Natal Care (%)** |
| High/Highest quintile | Institutional delivery rate | Mothers who received post natal care within 2 weeks of delivery |
| **Education** | Safe delivery | **Immunization (%)** |
| Non literate | **Post Natal Care (%): Mothers who received post natal care within** | Children age 12-23 months who received Full vaccination |
| 0-9 years of schooling | 48 hours of delivery | **Children with illness in the last 2 weeks where Mother gave ORS for diarrhoea** |
| >=10 years of schooling | 2 weeks of delivery | |

| **Religion** | **Child Health Indicators** | |
|---|---|---|
| Hindu | **Immunization (%): Children age 12-23 months who received** | |
| Sikh | Full vaccination | |
| Muslim | No vaccination | |
| Others | BCG vaccination | |
| **Caste** | DPT vaccine | |
| Schedule caste (SC) | OPV vaccine | |
| Schedule Tribe (ST) | **Breast Feeding Practices** | |
| Other Backward Caste (OBC) | **Women awareness about** | |
| General | Diarrhea management | |
| **Gender** | ARI | |
| **Sex of child** | **Prevalence of Diarrhoea/ARI** | |
| Female child | **Children with illness in the last 2 weeks where Mother gave ORS for diarrhoea** | |
| Male child | **Impact Indicators** | |
| | Maternal Mortality Ratio | |
| | Infant Mortality Rate | |
| | **Geographical inequality** | |
| | Absolute difference in maternal and child health indicators between urban and rural areas | |
| | **Socioeconomic inequality** | |
| | Absolute difference in maternal and child health indicators | |

| | | |
|---|---|---|
| | between most advantaged and least advantaged socioeconomic groups ( i.e., low vs high standard of living index, lowest vs highest quintile of wealth index) | |
| | **Gender inequality** | |
| | Absolute difference in child health indicators between male and female children. | |
