# Supplementary material for: Effectiveness of Multiple-Strategy Community Intervention in Reducing Geographical, Socioeconomic and Gender Based Inequalities in Maternal and Child Health Outcomes in Haryana, India
Source: PLoS One. 2016 Mar 22;11(3):e0150537. doi: 10.1371/journal.pone.0150537 (PMC4803212; doi:10.1371/journal.pone.0150537)
Supplement: S4 Table — (PDF) [file pone.0150537.s004.pdf]

S4 Table. Trend of availability and accessibility of health facilities during and after NRHM implementation in Haryana.

**Indicator**	**During NRHM**	**Post NRHM**
	**(2007-08)**	**(2012-13)**
**Health programmes at village level**	**N=721**	**N=708**
Percentage of villages having ASHA	80.8	96.1
Percentage of Villages having Village Health Nutrition and Sanitation Committee	68.2	43.5
**Accessibility of health facility (%)**		
Villages with Sub-Health Centre within 3 km	77	80.4
Villages with PHC within 10 km	82.3	87.3
**Availability of Health Infrastructure, Staff and Services of Sub-Health Centre (%)**	**N=625**	**N=673**
Sub-Health Centre located in government building	54.9	57.7
Sub-Health Centre with ANM	92.3	90.6
Sub-Health Centre with male health worker	51.8	58.2
Sub-Health Centre with ANM residing in Sub-Health Centre quarter where facility is available	31.4	35.6
Sub-Health Centre with additional ANM	74.2	83.5
**Primary Health Centre (PHC)**	**N=263**	**N=246**

PHCs functioning on 24 X 7 hours basis	39.2	79.3
PHCs having Lady Medical Officer	30.8	34.2
PHCs with at least 4 beds	64.3	75.7
PHCs with AYUSH (Ayurveda, Unani, Sidha and Homeopathy) doctor	1.5	19.1
PHCs having residential quarter for Medical Officer	43	47.9
PHCs having new born care services on 24 X 7 hours basis .	94.2	91.1
PHCs having referral services for pregnancies/delivery on 24 X 7 hours basis	46.6	65.6
PHCs conducted at least 10 deliveries during last one month on 24 X 7 hours basis	38.8	74.3
**Community Health Centre (CHC)**	**N=84**	**N=106**
CHCs having 24 X 7 hours normal delivery services	88.1	100
CHCs having Obstetrician/Gynaecologist	13.1	13.2
CHCs having Anesthetist	10.7	8.5
CHCs having functional Operation Theatre	60.7	46.2
CHCs designated as First referral units (FRUs)	44.1	71.7
CHCs designated as FRUs offering caesarean section	21.6	14.5
CHCs having new born care services on 24 X 7 hours basis	62.2	91.5
**District Hospital (DH)**	**N=18**	**N=21**

DHs having Paediatrician	77.7	95.2
DHs having regular radiographer	66.6	38.1
DHs having 2D Echo facility	22.2	38.1
DHs having ultrasound facility	NA	90.5
DHs having three phase connection	100	100
DHs having critical care area	44.4	76.2
DHs having suggestion and complaint box	55.5	100
