# Supplementary material for: Effectiveness of Multiple-Strategy Community Intervention in Reducing Geographical, Socioeconomic and Gender Based Inequalities in Maternal and Child Health Outcomes in Haryana, India
Source: PLoS One. 2016 Mar 22;11(3):e0150537. doi: 10.1371/journal.pone.0150537 (PMC4803212; doi:10.1371/journal.pone.0150537)
Supplement: S1 File — Haryana Report. Round 2. 2002–04. (PDF) [file pone.0150537.s005.pdf]

DLHS-2

HARYANA

Reproductive and Child Health

District Level Household Survey

2002-04

International Institute for Population Sciences,
(Deemed University)
Mumbai – 400 088

Ministry of Health & Family Welfare,
Government of India,
New Delhi – 110 011

Indian Institute of Health Management Research
Jaipur-302 011

Reproductive and Child Health

District Level Household Survey (DLHS-2)

Haryana

2002-04

International Institute for
Population Sciences,
(Deemed University)
Mumbai – 400 088

Ministry of Health & Family
Welfare, Government of India,
New Delhi – 110 011

Indian Institute of
Health Management Research
Jaipur-302 011

Contributors

Indian Institute of Health Management Research (IIHMR), Jaipur

Dhirendra Kumar
J.P. Singh
Laxman Sharma
J.B. Singh
Anil Singh Jha
Gowtham Ghosh

International Institute for Population Sciences (IIPS), Mumbai

F. Ram
B. Paswan
L. Ladu Singh
Uttam Sonkamble
Ananta Basudev Sahu

CONTENTS

		Page
	Tables	iv
	Figures	vii
	Maps…	vii
	Preface and acknowledgement	ix
	Key Indicators	xi
	Salient Findings	xiii
**CHAPTER I**	**INTRODUCTION**	
1.1	Background and Objectives of the Survey	1
1.2	Survey Design	2
1.3	House Listing and Sample Selection	2
1.4	Questionnaire	3
1.5	Fieldwork and Sample Coverage	5
1.6	Data processing	5
1.7	Sample Weights	5
1.8	Sample Implementation	6
1.9	Basic Demographic Profile of the State	8
**CHAPTER II**	**BACKGROUND CHARACTERISTICS OF HOUSEHOLD**	
2.1	Age – Sex Structure	11
2.2	Household Characteristic	12
2.3	Educational Level	14
2.4	Marital Status of the Household Population	17
2.5	Marriages	18
2.6	Morbidity Rates	19
2.7	Morbidity Rates by District	20
2.8	Housing Characteristics	21
2.9	Housing Characteristics by District	23
2.10	Iodization of Salt	24
2.11	Iodization of Salt by District	26
2.12	Availability of Facilities and Services in Rural India	26
2.13	Availability of Education Facility and Health Services by District	28
**CHAPTER III**	**CHARACTRERISTICS OF WOMEN, HUSBANDS AND FERTILITY**	
3.1	Background Characteristics of Women	33
3.2	Educational Level of Women	35
3.3	Background Characteristics of Husbands' of Eligible Women	36
3.4	Educational Level of Husbands' of Eligible Women	38
3.5	Children Ever Born and Surviving	39
3.6	Completed Fertility by District	41
3.7	Birth Order	42
3.8	Birth Order by District	43
3.9	Fertility Preference	45
3.10	Pregnancy Outcomes	46

		Page
**CHAPTER IV MATERNAL HEALTH CARE**		
4.1	Antenatal Check-Ups	50
4.2	Antenatal Check-Ups at Health Facility	52
4.3	Antenatal Check-Ups by District	54
4.4	Components of Antenatal Check-Ups	55
4.5	Antenatal Care Services	56
4.6	Antenatal Care Indicator by District	61
4.7	Pregnancy Complication and Treatment	62
4.8	Delivery Care	65
4.8.1	Place of Delivery	65
4.8.2	Assistance During Home Delivery	67
4.8.3	Delivery Assisted by Skilled Person	69
4.9	Reasons for Not Going to Health Institutions for Delivery	70
4.10	Delivery Characteristics by District	71
4.11	Complication during Delivery	72
4.12	Post Delivery Complication and Treatment	74
4.13	Obstetric Morbidity by District	78
**CHAPTER V CHILD CARE AND IMMUNIZATION**		
5.1	Breastfeeding	83
5.1.1	Breastfeeding by District	86
5.2	Immunization of Children	87
5.3	Source of Immunization	92
5.4	Reasons for Not Immunizing the Children	92
5.5	Vitamin A and Iron Supplementation	93
5.6	Immunization Coverage by District	95
5.7	Child Morbidity and Treatment	96
5.7.1	Awareness of Diarrhoea	96
5.7.2	Treatment of Diarrhoea	97
5.7.3	Awareness of Pneumonia	99
5.7.4	Treatment of Pneumonia	99
5.7.5	Awareness of Pneumonia and Incidence of Pneumonia by District	101
**CHAPTER VI FAMILY PLANNING**		
6.1	Knowledge of Family Planning Methods	105
6.1.1	Knowledge of Family Planning Methods by District	108
6.1.2	Knowledge of No-Scalpel Vasectomy (NSV)	108
6.1.3	Knowledge of No-Scalpel Vasectomy (NSV) by District	109
6.2	Current Use of Family Planning Methods	109
6.2.1	Current Use of Family Planning Methods by District	111
6.2.2	Current Use and Ever Use of Family Planning Methods by Women	112
6.2.3	Current Use and Ever Use of Family Planning Methods by Husbands	113
6.3	Reasons for Not Using Male Methods	114
6.4	Source of Contraceptive Methods	115

Page

6.5 Problems with Current Use of Contraceptive Method……..……………………… 117
6.6 Treatment for Contraceptive Related Health Problems.…..……………………… 118
6.7 Advice to Non-Users to Use Contraception…………..… 119
6.7.1 Future Intension to Use Contraceptive ….……………..………………………… 120
6.7.2 Future Intension to Use Among Women by Number of Living Children ………. 121
6.8 Reasons for Discontinuation and Non-Use of Contraception…………………… 122
6.8.1 Reasons for Not Using Contraceptive Methods………..………………………… 122
6.9 Unmet Need for Family Planning Services..…………..………………………… 123
6.9.1 Unmet Need for Family Planning Services by District…..……………………… 125

CHAPTER VII ACCESSIBILITY AND PERCEPTION ABOUT GOVERNMENT HEALTH FACILITIES

7.1 Home Visit By Health Worker……………………..…………………………… 127
7.2 Home Visit By Health Worker by District..…………..………………………… 129
7.3 Matter Discussed during Home Visit or Visits to Health Facilities.……………… 130
7.4 Visit to Health Facility………………..…………..…………………………… 132
7.5 Visit to Health Facility by District……………..…..…………………………… 133
7.6 Client's Perception of Quality of Government Health Services.………………… 133
7.7 Reasons for Not Visiting Government Health Centre…..………………………… 134
7.8 Family Planning Services and Advice Received…………..……………………… 135
7.9 Availability of Pills and Condom……..…………..…………………………… 135
7.10 Quality of Care of Family Planning Services…………..………………………… 136
7.11 Quality of Care of Family Planning Services District……………..……………… 138
7.12 Quality of Care of Maternal Health Care….…………..………………………… 139

CHAPTER VIII REPRODUCTIVE HEALTH PROBLEMS AND AWARENESS OF RTIs/STIs and HIV/AIDS

8.1 Awareness of RTI/STI…………….. …………………………………………… 141
8.1.1 Knowledge of Mode of Transmission of RTI/STI ……………………………… 145
8.2 Prevalence of RTI/STI ………………………………………………………… 147
8.3 Menstruation Related Problems……………………………………………….. 152
8.4 Prevalence of RTI/STI by District………..……………………………………… 153
8.5 HIV/AIDS……………………………………………………………………… 154
8.5.1 Knowledge of HIV/AIDS……………………………………………………… 154
8.5.2 Knowledge of Mode of Transmission about HIV/AIDS………………………… 158
8.5.3 How to avoid HIV/AIDS……………………………………………………… 160
8.5.4 Misconception about HIV/AIDS………………………………………………… 162
8.5.5 Knowledge of Curability of HIV/AIDS………………………………………… 164
8.6 Awareness of RTI/STI and HIV/AIDS by District……………..………………… 165

APPENDICES

Appendix A Estimation of Sampling Errors ……………………….…………….… 167
Appendix B DLHS Staff ………………………………………………………... 175
Appendix C Questionnaire ……………………………………………………..… 179

TABLES

		Page
Table 1.1	Number of household interviewed	7
Table 1.2	Number of women and husband interviewed	8
Table 1.3	Basic demographic indicator	10
Table 2.1	Household population by age and sex	12
Table 2.2	Household characteristics	13
Table 2.3	Educational level of the household population	14
Table 2.4	Marital status of the household population	17
Table 2.5	Marriage	18
Table 2.6	Morbidity rates	19
Table 2.7	Morbidity rates by district	20
Table 2.8	Housing characteristics	22
Table 2.9	Housing characteristics by district	24
Table 2.10	Iodization of salt	25
Table 2.11	Iodization of salt by district	26
Table 2.12	Distance from the nearest education facility	27
Table 2.13	Distance from the nearest health facility	27
Table 2.14	Availability of services	28
Table 2.15	Availability of facility and services by district	29
Table 3.1	Background characteristics of women	34
Table 3.2	Level of education of eligible women	36
Table 3.3	Background characteristics of men	37
Table 3.4	Level of education of men	39
Table 3.5	Children ever born and living	40
Table 3.6	Completed fertility by district	41
Table 3.7	Birth order	42
Table 3.8	Birth order by district	44
Table 3.9	Fertility preference	46
Table 3.10	Outcomes of pregnancy	47
Table 4.1	Antenatal check-up	51
Table 4.2	Place of antenatal check-up	53
Table 4.3	Antenatal check-ups by district	54
Table 4.4	Components of Antenatal check-ups	55
Table 4.5	Antenatal care	57
Table 4.6	Antenatal care indicators by district	61
Table 4.7	Pregnancy complications	63
Table 4.8	Treatment for pregnancy complications	65
Table 4.9	Place of delivery	66
Table 4.10	Assistance during home delivery and safe delivery	68
Table 4.11	Reasons for not going to health institutions for delivery	71
Table 4.12	Delivery characteristics by district	72
Table 4.13	Delivery complications	73
Table 4.14	Post delivery complications	75
Table 4.15	Treatment for post delivery complications	77
Table 4.16	Pregnancy, delivery and post delivery complications	78

		Page
Table 5.1	Initiation of breastfeeding	84
Table 5.2	Exclusive breastfeeding by child's age	85
Table 5.3	Breastfeeding by district	86
Table 5.4	Vaccination of children	88
Table 5.5	Childhood vaccination received by 12 months of age	91
Table 5.6	Source of childhood vaccination	92
Table 5.7	Reason for not giving vaccination	93
Table 5.8	Vitamin a and IFA supplementation for children	94
Table 5.9	Childhood vaccination by district	95
Table 5.10	Awareness of diarrhoea	97
Table 5.11	Treatment of diarrhoea	98
Table 5.12	Awareness of pneumonia	100
Table 5.13	Treatment of pneumonia	101
Table 5.14	Knowledge of diarrhoea management and pneumonia by district	102
Table 6.1	Knowledge of contraceptive methods	106
Table 6.2	Knowledge of contraceptive methods by district	107
Table 6.3	No-scalpel vasectomy (NSV)	108
Table 6.4	No-scalpel vasectomy by district	109
Table 6.5	Contraceptive prevalence rate	110
Table 6.6	Contraceptive prevalence rates by district	112
Table 6.7	Use of contraception by women	113
Table 6.8	Use of contraception by men	114
Table 6.9	Reasons for not using male methods	115
Table 6.10	Source of modern contraceptive methods	116
Table 6.11	Health problems with current use of contraception	117
Table 6.12	Follow-up visit and Sought treatment for health problems with current use of Contraception	118
Table 6.13	Advice on contraceptive use and future intention to use	119
Table 6.14	Future intention to use	120
Table 6.15	Future use of contraception by number of living children	121
Table 6.16	Reasons for discontinuation of contraception	122
Table 6.17	Reason for not using contraceptive method	123
Table 6.18	Unmet need for family planning services	124
Table 6.19	Unmet need by district	125
Table 7.1	Home visit by health worker	128
Table 7.2	Home visit by health worker by district	130
Table 7.3	Matter discussed during contact with a health worker	131
Table 7.4	Visit to health facility	132
Table 7.5	Visit to health facility by district	133
Table 7.6	Quality of government health facility	134
Table 7.7	Reason for not preferring government health facility	135
Table 7.8	Advise to adopt family planning method	136
Table 7.9	Availability of regular supply of condoms/pills	136
Table 7.10	Information of other modern method before sterilization	137

		Page
Table 7.11	Information on side effect and follow-up for current method …………..………	137
Table 7.12	Quality of care indicators for contraceptive users by district …………………..	138
Table 7.13	Advised to have delivery at health facility and follow-up services for Post Partum check-up …………….……..……………………………..……….	139
Table 7.14	Quality of care indicators for maternal care …………….……………..………	140
Table 8.1	Source of Knowledge about RTI/STI among women………………..……….	143
Table 8.2	Source of Knowledge about RTI/STI among men….. ……………..……….	144
Table 8.3	Source of Knowledge about mode of transmission of RTI/STI among women…	145
Table 8.4	Source of Knowledge about mode of transmission of RTI/STI among men……	146
Table 8.5	Symptoms of RTI/STI among women…….…………………………..……….	147
Table 8.6	Symptoms of RTI/STI among men …………………………………………….	150
Table 8.7	Abnormal vaginal discharge…………………..……………………..……….	151
Table 8.8	Menstruation related problems…………………………………………….…...	152
Table 8.9	Reproductive Health care indicators by district…………………………. …..	153
Table 8.10	Source of knowledge about HIV/AIDS among women ……………..……….	156
Table 8.11	Source of knowledge about HIV/AIDS among men …………………..………	157
Table 8.12	Source of knowledge about mode of transmission of HIV/AIDS among women	158
Table 8.13	Source of knowledge about mode of transmission of HIV/AIDS among men ...	159
Table 8.14	Knowledge about avoidance of HIV/AIDS among women …………..………	161
Table 8.15	Knowledge about avoidance of HIV/AIDS among men ……………..………	162
Table 8.16	Misconception about transmission of HIV/AIDS among women ………………	163
Table 8.17	Misconception about transmission of HIV/AIDS among men ………..………	164
Table 8.18	Knowledge of curability about HIV/AIDS …………………………..……….	165
Table 8.19	Awareness of RTI/STI and HIV/AIDS by district …………………..……….	166

FIGURES

		Page
Figure 2.1	Age-sex-pyramid …………………………………………………………..……….	11
Figure 2.2	Percentage literate by age and sex ……….…………………………..……….	15
Figure 3.1	Birth order 3 & above by selected background characteristic ………..……….	43
Figure 3.2	Birth order 3 & above by district ………………………………..……….	44
Figure 3.3	Fertility preference………………………………………………..……….	45
Figure 4.1	Source of antenatal care …………………………………………..……….	50
Figure 4.2	Full antenatal care by background characteristic ……………………..……….	60
Figure 4.3	Percentage of women with pregnancy complication and by symptoms …………	62
Figure 4.4	Place of delivery and assistance during delivery …………………..……….	69
Figure 4.5	Delivery assisted by skilled person by background characteristic ……………….	70
Figure 4.6	Percentage of women with delivery complication and by symptoms …………….	74
Figure 4.7	Percentage of women with post delivery complication and by symptoms.……….	76
Figure 5.1	Initiation of breastfeeding ………………………………………..……….	85
Figure 5.2	Percentage of children age 12-23 months who have received specific vaccination	89
Figure 5.3	Percentage of children age 12-23 months who have received all vaccination	90
Figure 5.4	Child vaccination by age …………………………………………..……….	91
Figure 6.1	Knowledge of family planning method ……………………………..……….	107
Figure 6.2	Practise of family planning method ……………………………..……….	111
Figure 6.3	Source of family planning among current users of modern contraceptive methods	116
Figure 7.1	Distribution of districts by home visit by health worker ……………..……….	129
Figure 8.1	Awareness of RTI/STI by sex according to residence …………………………..	142
Figure 8.2	Symptoms of RTI/STI among women ………………………………..………	148
Figure 8.3	Symptoms of RTI/STI among husband………………. ……………..……	149
Figure 8.4	Awareness of HIV/AIDS by sex according to residence ……………..……….	155

MAPS

Map 1	Percent Girl Marrying Below Legal Age at Marriage…………………..…….	30
Map 2	Percentage of Households Using Salt that Contains 15 ppm Level of Iodine ..……	31
Map 3	Percentage of Women Received Three or More Antenatal Check ups..……..……..	80
Map 4	Percentage of Delivery Attended by Skilled Person …..........…………….……….…	81
Map 5	Percentage of Children (Age12-23 Months) Who Have Received Full Vaccination	103
Map 6	Current Use of Any Family Planning Method…………………………..……..….	126

PREFACE AND ACKNOWLEDGEMENT

Government of India had launched the Reproductive and Child Health (RCH) programme to ensure that couples have access to adequate information and services for reproductive health care. As a first step, family planning target has been withdrawn and an effort is being made to provide a package of reproductive services at different levels of health care centres.

Monitoring of the services is also being improved. New indicators are being added to assess quality of services and provision of an integrated reproductive health care service. The District Level Household Survey (DLHS) was initiated by Government of India and financed by the World Bank covering all the districts in the country. For the second time, district level estimates will be available for most of the critical reproductive health indicators. These important initiatives are certainly quite satisfying for all those who are concerned with taking ICPD reproductive health agenda ahead. The project is being coordinated by International Institute for Population Sciences, Mumbai and implemented by a number of consulting agencies.

For the purpose of data collection, uniform questionnaires, sampling design and field procedures were used throughout the country. The survey thus provided comparable data for all the districts in the state. The present report provides salient findings of Haryana and covered all the districts. The findings of selected indicators of reproductive and child health services from the state of Haryana are presented in the report.

It is believe that the data generated through the survey will meet the requirements of the Programme Administrators and Policy Makers for making effective interventions for providing quality services and achieving multiple objectives.

The DLHS-RCH could not have been successfully completed without cooperation and support from innumerable sources at various stages of the project. Although, it is not possible to acknowledge everyone involve in the survey, several organizations and individuals deserve special mention.

We would like to take this opportunity to acknowledge Shri P. K. Hota, Secretary, Ministry of Health and Family Welfare (MoHFW), Government of India. Our special thanks are due to Shri Y.N. Chaturvedi, Shri A.R. Nanda and Shri J.V.R. Prasada Rao, former Secretaries, Department of Family Welfare, GoI, who have gave us an opportunity to participate as consulting organization in the survey of the national importance. Our special thanks are due to Shri S. K. Sinha, Additional Director General, Ministry of Health and Family Welfare, GoI. Thanks are also due to Dr. K.V. Rao, Shri S. K. Das and Shri.D.K. Joshi, former Chief Directors for their help. We are also thankful to Shri Partha Chattopadhyaya, Chief Director and Mr.K.D. Maiti, Director, Mrs. Rashmi Verma and Mr. Rezimohn, Assistant Director, Statistics division of MoHFW for all the support extended by them. Our special thanks are due to Dr.T.K.Roy, former Director and Senior Professor, IIPS, Mumbai, for his timely advice and valuable guidance. Thanks are due to Dr. G. Rama Rao, Officiating Director, IIPS, Mumbai. We also acknowledge the contribution of Dr. F .Ram, Dr. B. Paswan, Dr.L.Ladu Singh coordinators of the project at

IIPS, Mumbai. Our thanks are also due to the Directors or census Operations and the state Department of Health and Family Welfare in all the states and union territories. It also gives us immense pleasure to thanks to Dr. G.N.V. Ramana Rao, Public Health Specialist, World Bank, New Delhi for the able guidance and technical support to the project. We would also like to thanks to NSSO for their help providing UFS Block for DLHS-2.

Thanks are also due to Research Officers Dr. Manoj Alagarajan and Mr. Uttam Sonkamble, IIPS, for their assistance at various stages of the project.

We would like to thank our staff members who were associated with this study, especially Dr. Dhirendra Kumar, Associate Professor and Project Coordinator, Mr.J.P.Singh Sr. Research Officer, Mr. Laxman Sharma, Mr. J.B. Singh, Research Officers and Mr. James.E.J.and Ms Ubida Sulthana, Trainee Research Officers. We appreciate the hard work of Mr. N.K. Jacob for typing the report. We thank the administrative staff of IIHMR for the effective logistic arrangements to conduct the fieldwork in time. We also express our appreciation for the efforts made by the house-listing teams, interviewers, supervisors and editors in the data collection. Mr. Atal Khandelwal and Mr. Rakesh Mathur from computer cell deserve a special mention for their support in the data processing.

We would be failing in our duty if we do not thank our respondents who spent their valuable time with tremendous patience.

S.D. Gupta, M.D, Ph.D.
Director
Indian Institute of Health Management Research, Jaipur
June, 2006.

SALIENT FINDINGS

For the assessment of district level Reproductive and Child Health indicators, Government of India proposed to undertake district level household surveys through non-governmental agencies on an annual basis. The District Level Household Survey (DLHS) was the result of government's initiative. In Haryana, Indian Institute of Health management Research, Jaipur, was entrusted the work of carrying out of the survey. The survey for Phase-1 of the DLHS covering 9 districts of the state was conducted during May 2002 to August 2002. The survey for Phase-2 covering the remaining districts of the state was carried out during May 2004 to August 2004. The focus of the survey was on: i) Coverage on antenatal care (ANC) and immunization services, ii) Extent of safe deliveries, iii) Contraceptive prevalence rate and unmet need for family planning, iv) Awareness about RTI/STI and HIV/AIDS and v) Utilization of government health services and users' satisfaction. The salient findings of the survey are presented here.

For both the phases together, the data was collected from 20,205 households in Haryana. From these households, 18,796 eligible women (usual resident or visitors who stayed in the sample household the night before the interview, currently married aged 15-44 years whose marriage was consummated) and 13,200 husbands of eligible women were interviewed.

Of the total households interviewed in Haryana, nearly 32 percent were from urban areas. There were 90 percent Hindu households and 5 percent each Sikh and Muslim households. Twenty three percent of the households belonged to either scheduled castes or scheduled tribes. Only 8 percent of the households lived in *Kachcha*, about 47 percent are in Semi-*pacca* and 45 percent are in *pucca* houses. About half of the households belonged to medium economic status (44 percent in medium SLI)

About 71 percent of population aged seven and above are literate. Percent literate among females is 59 where as it is 81 percent for male. Proportion of non-literate is much higher among the older cohort compared to the younger ones. Nearly 45 percent of eligible women in the state are non-literate and 25 percent have completed 10 or more years of schooling. In Haryana the level of literacy among the eligible women and their husbands are low. As regards distribution of non-literate women, lesser proportion of younger women below age 30 are illiterate compared to older women age 30 and above, same is the case of non-literate husbands.

The reporting of the marriages during three yeas prior to survey gives the mean age at marriage among the boys and girls in the state is 23 and 19 years respectively. Twenty nine percent of boys and 28 percent of girls in the state got married before attaining the minimum legal age at marriage of 21 and 18 years respectively. In all the districts, except Ambala, Panchkula and Rohtak more than 20 percent of boys got married below the legal minimum age at marriage. Except in Ambala and Kurukshetra, in all the districts more than 10 percent of the girls got married below the legal minimum age at marriage.

About half of the households (55 percent) use cooking salt that is iodized at the recommended level of 15 parts per million or higher level of iodine content; whereas 26 percent of households used salts that are not iodized at all. Lowest proportion of households (12 percent)

in Kaithal, Panchkula and Sonipat are using non-iodised salt; whereas in Bhiwani the highest proportion of households (48 percent) used non-iodized salt. More than half of the households in all the districts of Haryana consume adequately iodized salt except the districts of Bhiwani, Fatehabad, Gurgaon, Hisar, Mahendragarh, Rewari and Sirsa.

On an average, women on the verge of completion of reproductive period have given birth to 4.1 children. The completed fertility in the state varies from the lowest of 3.5 children ever born per women in a Panchkula and Rewari to the highest of 6.2 children in Gurgaon.

The share of births of order 3 and above in the total births that occurred three years prior to survey is 38 percent. In most of the districts, proportion of higher order births is quite high, ranging from the lowest of around 24 percent in Panchkula to the highest of about 56 percent in Gurgaon.

The data collected on the utilization of ANC services for the women who had their last live/ still birth during three years prior to survey shows that the ANC coverage in the state is high as 88 percent of the women received at least one ante-natal care during pregnancy. About three percent of the women during their pregnancy were visited by health worker at their residence for providing ANC. Forty six percent of the women visited government health facilities and 27 percent received ANC from private health facilities. The percent of women who got some kind of ANC during pregnancy range between 67 percent in Gurgaon to all the women in Ambala. In 13 districts out of 19, 90 percent or more women got some antenatal care.

Though 88 percent of the women in Haryana received ANC, only 60, 49 and 45 percent women had check-up or test of abdomen, blood and blood pressure and urine respectively. Sixty eight percent women received Iron and Folic Acid (IFA) tablets and 86 percent got at least one TT injection. A full package of ANC including minimum three ANC visits, at least one TT injection and 100 or more IFA tablets/Syrup was received by 12 percent of women.

Minimum three ANC and timing of first check up are crucial for maternal and childcare. In Haryana around 45 percent of women got ANC in the first trimester and nearly 49 percent had minimum three antenatal check-ups. An extent of ANC in first trimester varies from minimum of 28 percent Gurgaon to the maximum of 63 percent in Ambala. In Gurgaon, only 34 percent of women had minimum three ANC whereas in Ambala more than 81 percent women had got minimum three ANC.

Nearly 35 percent of the total deliveries in Haryana were conducted in the health institutions; 9 percentages point up from RCH Round I. The majority of the institutional deliveries were conducted in private institutions (25 percent of total deliveries) as against in government institutions (11 percent of total deliveries). Thirty seven percent of the total deliveries, that took place at home, were assisted by midwifery trained persons i.e. doctor/nurse, ANM and TBA. So in all, 43 percent of the deliveries, slightly up from RCH Round I (33 percent), in the state were assisted by skilled personnel. The extent of institutional deliveries varies from the highest of 62 percent in Ambala to the lowest of 20 percent in Gurgaon. The percent of the institutional deliveries increases substantially with women’s education and economic status.

In Haryana, 31, 20 and 24 percent of the women experienced pregnancy, delivery and post delivery complications respectively. About 49 percent of the women sought treatment for the pregnancy and 54 percent for the post-delivery complications. The pregnancy complication varies from the lowest of 18 percent in Hisar to the highest of 48 percent in Karnal.

In most of the districts and the state as a whole, the practice of breast-feeding is almost universal. However, the practice of initiation of breastfeeding within two hours of birth of the child is not common. In Haryana, only 17 percent women started breastfeeding the child within two hours of birth and nearly 63 percent started after one day of birth. There is great deal of variation in the pattern of breastfeeding across the districts. In Yamunanagar district only 8 percent of the women breastfed the child within two hours of birth and in Panchkula district, the percentage is highest (33 percent).

In Haryana 83, 73, 73 and 65 percent of the children received the BCG vaccine, three doses of DPT, Polio and measles vaccine respectively. There is 18 percentage points drop from BCG to measles. It means that large number of children that have contact with services providers are missed out of subsequent services. The complete schedule of immunization including BCG, three doses of DPT and Polio each and measles was received by 59 percent of the children, whereas 12 percent of the children did not receive a single vaccination under routine programme. About 38 percent of the children received supplementation of at least one dose of vitamin A and only 6 percent children received IFA tablets/liquid for iron supplementation.

The extent of complete immunization consisting of BCG, three injections of DPT, three doses of Polio and measles is the lowest in Gurgaon (33 percent) and highest in Ambala (93 percent). In four districts, more than 75 percent of the children received complete immunization.

In Haryana, 72 percent of the women were aware of diarrhoea management and 30 percent were aware of Oral Rehydration Salt (ORS). During a two-week period prior to survey, children of 18 percent of the women suffered from diarrhoea and 32 percent women treated diarrhoea among children by giving ORS. In comparison to awareness about diarrhoea management, the awareness about danger sings of pneumonia is quite low. Only half of the women reported awareness about danger sings of pneumonia. Eleven percent of the women reported that their children suffered from cough, cold and difficulty in breathing in two-week period prior to survey and 78 percent of them sought treatment.

The knowledge of family planning methods is universal in all districts of Haryana, all the women reporting knowledge of one method or the other. However, the knowledge of any spacing method is marginally low, but the proportion *per se* is quite high (95 percent). The knowledge of any modern methods is also universal in the state, though the knowledge of all modern methods is 95 percent. The proportion knowing all modern methods (males and females' sterilization, IUD, oral pills and condom) varies from about 30 percent in Sirsa to 90 percent in Kurukshetra.

In DLHS, knowledge about No-scalpel vasectomy has been asked to husbands of eligible women. About half (50 percent) of the husbands were aware of no-scalpel vasectomy in the state. The proportion of husbands knowing No-scalpel vasectomy varies from about 41 percent in Panipat to 60 percent in Mahendragarh.

The contraceptive prevalence rate (any methods) in the state is 60 percent, 2 percentage point up from RCH Round I, comprising of prevalence of about 54 percent of modern methods and 6 percent of traditional methods. Thirty seven percent of the couples adopted sterilization. The user of the two male methods sterilization and condom is only 11 percent. There has been positive association between contraceptive use and female education, economic development and availability of health facility. The highest contraceptive prevalence is in Ambala (71 percent), followed by Panchkula (70 percent) and lowest is in Gurgaon (42 percent).

In Haryana, a total of 15 percent of women are found to have unmet need for family planning, with 9 percent for limiting and 6 percent for spacing. The total unmet need varies from 6 percent in Ambala to 27 percent in Gurgaon.

Only 3 percent of the women in the state reported that either ANM/LHV or health worker visited them at their residence at least once in the past three months. Most of the women (81percent) who were visited by ANM felt that ANM had given them sufficient time to discuss health-related matters.

In all the districts, except Panchkula and Karnal, less than 5 percent of the women reported the visit of ANM/LHV to their residence. In the 6 districts only 1-2 percent of the women reported visits of ANM/LHV and in the remaining 3-9 percent of the women reported visit of ANM/LHV.

It has been observed that in three months period prior to survey, 31 percent of the eligible women who were required to consult health facility visited any of the government health facilities. Very small proportion of the women who visited the health facility rated facility as excellent. On the other hand, nearly 17 percent of the women who did not visit the government health facility reported government health facility “non-conveniently located” or “poor quality of services”(25 percent) as reason.

The district level variation in the utilization of the government health facilities ranges from 18 percent in Karnal to 45 percent in Mahendragarh. A large percentage of women visited to private health facilities (69 percent), ranges from 49 percent in Panchkula to 79 percent in Kurukshetra.

In Haryana, 51 and 55 percent of women are aware of RTI/STI and HIV/AIDS respectively. The corresponding level of awareness among husbands of eligible women is 54 and 86 percent. The percent of women who are aware of RTI/STI and HIV/AIDS is lowest in Bhiwani (15 percent) and Sirsa (31 percent) respectively to highest in Kurukshetra (93 percent) and Ambala (76 percent) respectively. Similarly awareness level of husbands of eligible women of RTI/STI and HIV/AIDS are lowest in Karnal and Panchkula (38 percent each) and Sirsa (76 percent) respectively to the highest in Yamunanagar (78 percent) and Kurukshetra (98 percent) respectively. Out of 19, in 8 districts the awareness of HIV/AIDS is below state figure for women and in 10 districts for their husbands.

About 31 percent of women and 6 percent of husbands of eligible women in the state reported having at least one symptoms of RTI/STI. In all the districts the reported prevalence of RTI/STI among husbands was low. The prevalence of RTI/STI is lowest in Ambala and Faridabad (15 percent each) for women and in Kurukshetra, Ambala and Hisar (2 percent each) for husbands to highest in Karnal (55 percent) for women and in Kaithal (14 percent) for husbands. About 16 percent of women reported vaginal discharge with lowest in Fatehabad (6 percent) to highest in Gurgaon (31 Percent). Thirty eight percent of women sought treatment for vaginal discharge problem and 43 percent of husbands sought treatment with at least one symptoms of RTI/STI. It may be noted that in Faridabad, Jhajjar, Kurukshetra, Rewari and Sonipat districts higher proportion of women compared to husbands sought treatment for their reproductive health problems.

CHAPTER I

INTRODUCTION

1.1 Background and Objectives of the Survey

The Reproductive and Child Health (RCH) programme that has been launched by Government of India (GoI) in 1996-97 is expected to provide quality services and achieve multiple objectives. It ushered a positive paradigm shift from method-oriented, target-based activity to providing client-centred, demand-driven quality services. Also, efforts are being made to reorient provider's attitude at grassroots level and to strengthen the services at outreach levels.

The new approach requires decentralization of planning, monitoring and evaluation of the services. The district being the basic nucleus of planning and implementation of the RCH programme, Government of India has been interested in generating district level data on utilization of the services provided by government health facilities, other than that based on service statistics. It is also of interest to assess people's perceptions on quality of services. Therefore, it was decided to undertake District Level Household Survey (DLHS) under the RCH programme in the country.

The Round I of RCH survey was conducted during the year 1998–99 in two phases (each phase covered half of the districts from all states/union territories) in 504 districts, for which International Institute for Population Sciences (IIPS), Mumbai was designated as the nodal agency.

In Round II, survey was completed during 2002-04 in 593 districts as per the 2001 Census. In DLHS-RCH, information about RCH has been collected using a slightly modified questionnaire. In Round II, some new dimensions, such as test of cooking salt to assess the consumption of salt fortified with iodine, collection of blood of children, adolescents and pregnant women to assess the level of anaemia and measurement of weight of children to assess the nutritional status, were incorporated.

The main focus of the DLHS-RCH has been on the following aspects:

- Coverage of ANC and immunization services
- Proportion of safe deliveries
- Contraceptive prevalence rates
- Unmet need for family planning
- Awareness about RTI/ STI and HIV/AIDS
- Utilization of government health services and users' satisfaction.

For the purpose of conducting DLHS-RCH, all the states and the union territories were grouped into 16 regions. A total of twelve research organizations including Population Research Centres (PRCs) were involved in conducting the survey in 16 regions with IIPS as the nodal agency.

1.2 Survey Design

In Round II, a systematic, multi-stage stratified sampling design was adopted. In each district, 40 Primary Sampling Units (PSUs – Villages/Urban Frame Size) were selected with probability proportional to size (PPS) using the 1991 Census data. All the villages were stratified according to population size, and female literacy was used for implicit arrangement within each strata. The number of PSUs in rural and urban areas was decided on the basis of percent of urban population in the district. However, a minimum of 12 urban PSUs was selected in each district in case the percent urban was low. The target sample size in each district was set at 1,000 complete residential households from 40 selected PSUs. In the second stage, within each PSU, 28 residential households were selected with Circular Systematic Random Sampling (CSRS) procedure after house listing. In order to take care of non-response due to various reasons, sample was inflated by 10 percent (i.e. 1,100 households).

For selecting the urban sample, the National Sample Survey Organization (NSSO) provided the list of selected urban frame size (UFS) blocks in the district. The UFS blocks were made available separately for each district for urban areas. The maps of selected blocks were obtained from the NSSO field office located in each state/union-territory.

But in each state, in two districts, the PSUs that were surveyed in Round I of DLHS-RCH (also known as RHS-RCH) were also selected for survey in Round II. This was done in order to measure the changes more accurately. Two districts, one with the highest proportion of safe delivery and another with the lowest proportion of safe delivery among those surveyed during Round I of the survey were selected for this purpose. In all other districts, fresh sample of PSUs were selected.

1.3 House Listing and Sample Selection

The household listing operation was carried out in each of the selected PSU segment prior to the data collection that provided the necessary frame for selecting the households. The household listing operation also involved preparation of location map and layout sketch map of the structures and recording the details of the households in these structures in each selected PSU. This exercise was carried out by independent teams each comprising one lister, one mapper and one supervisor under the overall guidance and monitoring of the survey coordinator of households of the selected regional agencies.

A complete listing of households was carried out in villages with households up to 300. In case of villages with more than 300 households but less than or equal to 600 households, two segments of more or less same size were formed and one segment was selected at random and household listing was carried out. In case of villages with more than 600 households, segments each of about 150 households were formed and two segments were selected for listing using the systematic random sampling method.

Small villages with less than 50 households were linked with a nearest village. After combining it with the nearest village, the same sampling procedure was adopted as mentioned above.

For the urban PSUs, the selected UFS blocks needed no segmentation as they were of almost equal size and contained less than 300 households.

No replacement was made if selected household was absent during data collection. However, if a PSU was inaccessible, a replacement PSU with similar characteristics was selected by the IIPS and provided to the regional agency for survey.

1.4 Questionnaire

DLHS-RCH collected information on a various indicators pertaining to RCH that would assist policymakers and programme managers to formulate and implement the goals set for RCH programmes. The International Institute for Population Sciences (IIPS), Mumbai, the Nodal Agency for DLHS–RCH project has made necessary modifications in the two Questionnaires: Households Questionnaire and Women's Questionnaire and added three more Questionnaires i.e., Husband's Questionnaire, Village Questionnaire and Health Questionnaire, in consultation with MoHFW and World Bank. These Questionnaires were discussed and finalized in training cum workshop organized at IIPS during the first week of November 2001.

These modified questionnaires had been canvassed of round II of the DLHS–RCH survey, taking into consideration the views of all the regional agencies involved. The house–listing teams and the interviewers and the supervisors for the main survey were given rigorous training based on the manuals developed for the purpose by the Nodal Agency.

All the questionnaires were bilingual, with questions in both regional and English language.

The Details of questionnaires are as follows:

Household Questionnaire: The household questionnaire lists all usual residents in each sample household including visitors who stayed in the household the night before the interview. For each listed household member, the survey collected basic information on age, sex, and marital status, relationship to the head of the household, education and the prevalence /incidence of tuberculosis, blindness and malaria. Information was also collected on the main source of drinking water, type of toilet facility, source of lighting, type of cooking fuel, religion and caste of household head and ownership of other durable goods in the household. In addition, a test was conducted to assess whether the household used cooking salt that has been fortified with iodine. Besides, details of marriages and deaths which happen to usual residents within reference period were collected. Efforts were also made to get information about maternal deaths.

Women Questionnaire: Women questionnaire is designed to collect information from currently married women age 15 – 44 years who are usual residents of the sample household or visitors who stayed in the sample household the night before the interview. The women questionnaire covered the following sections:

Section I: Background Characteristics: In this section the information collected on age, educational status and birth and death history of biological children including still birth, induced and spontaneous abortions.

Section II: Antenatal, Natal and Post natal Care: In this section the questionnaire collect information only from the women who had live birth, still birth, spontaneous or induced abortion during last three years preceding the survey date. The information on whether women received antenatal and postpartum care, who attended the delivery and the nature of complications during pregnancy for recent births were also collected.

Section III: Immunization and childcare: This section gives information about feeding practices, the length of breastfeeding, immunization coverage and recent occurrence of diarrhoea, and pneumonia for young children (below age 3 years).

Section IV: Contraception: This section provides information on knowledge and use of specific family planning methods. Questions were included about reasons for non-use, intentions about future use, desire for additional child, sex preference for next child etc.

Section V: Assessment of quality of Government health services and client satisfaction. In this section the questions are targeted to assess the quality of family planning and health services provided by Government health facilities. The information was also collected about the rating of Government health facilities and staffs and reasons for not visiting to government health facilities by eligible woman.

Section VI: Awareness about RTI/STI and HIV/AIDS: In this section the information were collected about women's knowledge of RTI/STI about awareness, Source of knowledge, aware of mode of transmission, curability, symptoms and treatment seeking behaviour. About HIV/AIDS; Awareness, Source of knowledge, aware of mode of transmission and prevention etc were canvassed.

Husband Questionnaire: In DLHS-RCH, round II, husband questionnaire was used to collect information from eligible women's husbands about age; educational status, knowledge and source of knowledge of RTI/STI and HIV/AIDS reported symptoms of RTI/STI and male participation. Apart from these information desires for children, reasons for not using F.P. methods, future intention to use F.P. methods and knowledge about no scalpel vasectomy (NSV) has also been collected.

Health Questionnaire: In DLHS-RCH, round II, a health questionnaire is included. The information collected were on weight of children age 0–71 months old and the blood sample to assess the haemoglobin levels of children age 0–71 months old, adolescents 10–19 years old and pregnant eligible women. This information is useful for assessing the levels of nutrition prevailing in the population and prevalence of anaemia among women, adolescent girls and children.

Village Questionnaire: A village questionnaire is also added in this round of DLHS. The information collected on the availability and accessibility of various facilities in the village especially on accessibility of educational and health facilities.

1.5 Fieldwork and Sample Coverage

The fieldwork for RCH Round II was done in two phases. During Phase I, 10 districts were covered from May 2002 to August 2002 and remaining 9 districts were covered during Phase II from May 2004 to August 2004.

During Round II, a total of 20,205 households were covered. From these surveyed households, 18,796 currently married women (aged 15-44 years) and 13,200 husbands of eligible women were interviewed.

1.6 Data processing

All the five types of completed questionnaires were brought to the headquarter of regional agencies and data were processed using microcomputers. The process consisted of office editing of questionnaires, data entry, data cleaning and tabulation. Data cleaning included validation, range and consistency checks. For both data entry and tabulation of the data, IIPS developed the software package. The district and state level reports were prepared by regional agencies, whereas national report is prepared by the nodal agency.

1.7 Sample Weights

In generating district level demographic indicator sample weight for household, women and husband, weight have been used and these for a particular district are based on three selection probabilities f_1^i, f_2^i and f_3^i pertaining to i^{th} PSU of the district. These probabilities are defined as

f_1^i = Probability of selection of ith PSU in a district

$$= \frac{(n_r * H_i)}{H}$$

Where, n_r is the number of rural PSU to be selected in a district, H_i refers to the number of household in the ith PSU and $H = \sum H_i$, total number of household in a district.

f_2^i = Probability of selecting segment (s) from segmented PSU
(in case the ith selected PSU is segmented)

= (Number of segments selected after segmentation of PSU) / (number of segment created a PSU)

The value of f_2^i is to be equal to one for un-segmented PSU.

f_3^i = probability of selecting a household from the total listed households of a PSU or in segment(s) of a PSU

$$= \frac{28 * HR_i}{HL_i}$$

Where HR_i is the household response rate of the ith sampled PSU and HL_i is the number of households listed in i^{th} PSU in a district.

For urban PSU, f_1^i is computed either as the ratio of number of urban PSUs to be included from the district to the total number of UFS blocks of the district or as the ratio of urban population of the selected PSU to the total urban population of the district.

The probability of selecting a household from the district works out as;

$$f^i = \left(f_1^i * f_2^i * f_3^i\right)$$

The non-normalized household weight for the ith PSU of the district is, $w^i = \frac{1}{f^i}$, while the normalized weight used in the generation of district indicators as

$$n_i^d = \frac{\sum_i n_i}{\sum_i n_i * w^i} * w^i, \text{ i= 1,2,3…………….40.}$$

Where n_i is the number of households interviewed in the ith PSU. The weight for women and husband are computed in the similar manner after multiplication of expression for f^i by the corresponding response rate. State weights for households, women and husbands are further derived from the district weights n_i^d for the ith psu in d^{th} district using external control so that for sample results do not deviate from the corresponding information about the population.

Let, $n_s = \sum_i n_i^d$ and $N_I = \sum_i N_i^d$, denote the number of households in the sample and census of a particular state, then state level households weights are work out as;

$$n_i^s = n_i^d * \frac{\left(n_i^d / n_s\right)}{\left(N_i^d / N_{sc}\right)}$$

, where n_i^d household sample in i^{th} district, n_s is the total sample in the state, N_i^d is the census population in the ith district and N_{sc} is the census population in the state. These households' weights are controlled for rural-urban separately.

Considering sample and census currently married women in 15-44 years and married males above 15 years for specified state by districts and rural-urban residence, state level women and husbands' weights are obtained for estimation of state level indicators.

1.8 Sample Implementation

Table 1.1 shows the period of fieldwork, number of households interviewed and household's response rates. A total of 20,205 households are interviewed, about two-thirds were rural. The overall household response rate – the number of households interviewed per 100 occupied

household - was 99 percent. The household response rate was more than 98 percent in every district.

Table 1.1 NUMBER OF HOUSEHOLDS INTERVIEWED

Month and year of fieldwork and number of households interviewed by district, Haryana, 2002-04

State/District	Month and year of field work		Number of households interviewed			Response rate
	From	To	Total	Rural	Urban	
**State**	-	-	20,205	13,832	6,373	99.1
**State-phase I**	05/2002	08/2002	10550	7131	3419	
**State-phase II**	05/2004	08/2004	9655	6701	2954	
Bhiwani	07/2002	08/2002	1,082	768	314	99.4
Faridabad	07/2002	07/2002	1,049	483	566	98.3
Gurgaon	07/2002	07/2002	1,024	848	176	98.9
Jind	08/2002	08/2002	1,055	742	313	99.1
Kaithal	05/2002	06/2002	1,063	756	307	98.2
Mahendragarh	06/2002	06/2002	1,077	761	316	99.1
Panchkula	07/2002	08/2002	1,016	723	293	98.7
Panipat	06/2002	06/2002	1,022	628	394	97.5
Sirsa	05/2002	06/2002	1,066	755	311	99.5
Yamunanagar	07/2002	07/2002	1,096	667	429	99.4
Ambala	06/2004	07/2004	1,074	711	363	99.8
Fatehabad	07/2004	07/2004	1,067	746	321	98.9
Hisar	07/2004	08/2004	1,097	768	329	99.9
Jhajjar	06/2004	07/2004	1,065	753	312	99.2
Karnal	05/2004	06/2004	1,042	748	294	98.5
Kurukshetra	06/2004	07/2004	1,093	767	326	99.7
Rewari	06/2004	07/2004	1,080	759	321	99.6
Rohtak	07/2004	07/2004	1,072	699	373	99.6
Sonipat	05/2004	06/2004	1,065	750	315	98.9

Note: Table based on unweighted cases.

In the interviewed households, interviews were completed with 18,796 currently married women who are the usual member of the household or stayed night before the household interview and 13,200 husbands of eligible women were also interviewed (Table 1.2). The number of completed interviews per 100 identified eligible women and husbands in the households with completed interviews were 89 and 68 percent respectively. The variation in the women’s response rate by district was highest in Ambala (96 percent) and lowest in Bhiwani, Gurgaon, Kaithal, Panipat, Sirsa, Rewari and Sonipat districts (87 percent), similarly husband’s response rate was found to be highest in Jhajjar (81 percent) and lowest in Gurgaon (57 percent).

Table 1.2 NUMBER OF WOMEN AND HUSBANDS INTERVIEWED

Number of women and husbands interviewed by district, Haryana, 2002-04

State/District	Number of women interviewed			Response rate	Number of husbands interviewed			Response rate
	Total	Rural	Urban		Total	Rural	Urban	
State	18,796	13,307	5,489	89.4	13,200	9,387	3,813	68.0
Bhiwani	1,056	756	300	86.8	779	564	215	69.9
Faridabad	942	488	454	89.4	744	373	371	72.9
Gurgaon	972	813	159	87.0	591	495	96	56.9
Jind	1,002	694	308	88.1	615	439	176	58.7
Kaithal	898	649	249	87.1	848	617	231	84.0
Mahendragarh	1,034	724	310	88.2	748	527	221	68.9
Panchkula	957	737	220	88.6	704	548	156	66.7
Panipat	917	570	347	87.1	611	415	196	61.1
Sirsa	913	680	233	86.7	617	465	152	61.3
Yamunanagar	1,036	632	404	91.7	650	412	238	59.7
Ambala	1,010	726	284	96.4	574	404	170	59.4
Fatehabad	998	715	283	88.5	762	551	211	73.1
Hisar	1,033	727	306	93.5	679	486	193	68.0
Jhajjar	1,003	722	281	92.8	731	526	205	80.9
Karnal	1,004	751	253	90.1	720	539	181	71.4
Kurukshetra	1,057	797	260	91.0	783	587	196	72.2
Rewari	1,029	746	283	86.8	724	503	221	70.7
Rohtak	919	626	293	92.5	581	386	195	65.3
Sonipat	1,016	754	262	86.5	739	550	189	71.5

Note: Table based on unweighted cases.

1.9 Basic Demographic Profile of the State

Before presenting the survey result, the basic demographic features of Haryana and its districts (as per census 2001) are presented here.

According to 2001 census, the total population of the state was 21.08 million and the growth rate during 1991-2001 was recorded as 2.47 percent, which is higher than that of India (1.93 percent). Haryana constitutes 2.1 percent of India's population. The sex ratio of Haryana is 861 females per thousand males, which is lower than that of India's sex ratio of 933. The levels of literacy among males and females above age 7 in the state are 79 percent and 56 percent respectively. The literacy rates in the state are higher than that in the country. There are 19 districts in the state with Chandigarh as its capital. The level of urbanization in the state is 29 percent, which is little higher than the level of urbanization in India. About one-fifth of the state population belongs to scheduled caste, while there was no scheduled tribe population in the state.

Haryana has an area of 44212 square km. Haryana is bounded on the east by Uttar Pradesh, Punjab on the West, Himachal Pradesh on the North and river Yamuna in the east. The Aravalli range, which stretches from Delhi to Gujarat, also acts as Haryana's south western boundary and runs through its Gurgaon region. The river Ghaggar provides a kind of boundary in the west of the state.

Faridabad is the most populated district with 2.2 million people. The urban population in Faridabad, Panchkula and Panipat exceeds 40 percent. In every district, with the exception of Panipat, Panchkula, Faridabad and Gurgaon the scheduled caste population is more than 16 percent, which is above the national average, there is no scheduled tribe population in the state. The literacy levels of both the males and females are above the national average. There is considerable difference in the literacy levels in the different districts. While the literacy rate in Ambala and Rewari is 75 percent, it is 58 percent in Fatehabad.

According to 2001 census the rural and urban breakup of the population shows that 71 percent of the population was enumerated in rural areas and 29 percent in urban areas. Keeping pace with the national average, Haryana has recorded increase in the decadal growth rate from 27.4 per cent in 1981-91 to 28.4 percent during 1991-2001. Among the districts, Panchkula with 51.2 percent has the highest decadal growth rate whereas Mahendragarh with 19.1 percent has the lowest decadal growth rate of total population during 1991-2001.

Percentage of Scheduled Caste population has experienced a marginal decline during 1991-2001 and the proportion of schedule caste population in total population of 2001 is 19.3 percent. Highest proportion of Schedule Caste population has been recorded in Fatehabad district (27.4 per cent) and Gurgaon has the lowest proportion of Schedule Caste population (11.3 percent). With a population density of 478 per sq. km., Haryana ranks 11th among the states and union territories in India and this figure is higher than the all India density of 325 persons per square km. Among the districts, Faridabad has the highest density (1020 person/sq. km.) and Fatehabad has the lowest (318 person/sq. km).

The sex ratio of the total population in the state has declined since 1991 Census from 865 to 861 per 1000 males. Mahendragarh has recorded the highest sex ratio (918) and Panchkula has the lowest (823) within the state. In all the districts except Mahendragarh, the sex ratio was below 900 females per 1000 males.

The literacy rate in the state has improved from 55.9 percent in 1991 to 67.9 percent in 2001 and it is higher than the national average of 64.8 percent. Among the districts, Ambala has the highest literacy rate of 75.3 percent. Fatehabad has the lowest literacy rate of 58 percent. The male literacy of the state is 78.5 percent and the female literacy rate is 55.7 percent. Both the rates have increased from 1991 census to 2001 census.

Table 1.3 BASIC DEMOGRAPHIC INDICATOR

Basic demographic indicator of India, state and districts, Census 2001 Haryana

India/state/district	Population (in thousand)	Percentage urban	Percentage decadal growth rate[1]	Sex ratio[2]	Percentage literate 7+		
					Male	Female	Persons
India	1028737	28.0	21.5	933	75.3	53.7	64.8
State	21144	28.9	28.4	861	78.5	55.7	67.9
Bhiwani	1425	19.0	22.5	879	81.19	53.0	67.4
Faridabad	2195	55.7	48.5	839	82.49	56.3	70.0
Gurgaon	1660	22.2	44.6	873	77.11	47.8	62.9
Jind	1190	20.3	21.4	852	74.69	48.5	62.1
Kaithal	946	19.4	21.0	853	69.81	47.3	67.7
Mahendragarh	813	13.5	19.1	918	85.31	54.1	69.9
Panchkula	468	44.5	51.2	823	82.74	65.7	74.0
Panipat	967	40.5	38.6	829	79.16	58.0	69.2
Sirsa	1117	26.3	23.0	882	70.93	49.9	60.6
Yamunanagar	1042	37.7	21.8	862	79.28	60.6	71.6
Ambala	1014	35.2	25.7	868	83.01	63.4	75.3
Fatehabad	806	17.6	24.8	884	68.71	46.5	58.0
Hisar	1230	25.9	27.1	851	77.62	52.1	65.9
Jhajjar	709	22.2	24.1	847	83.26	59.9	72.5
Karnal	1274	26.5	23.1	865	76.74	58.0	67.7
Kurukshetra	825	26.1	23.7	866	78.23	67.4	69.9
Rewari	765	17.8	25.2	899	89.04	60.8	75.2
Rohtak	940	35.1	21.0	847	84.29	62.6	73.7
Sonipat	1279	25.1	22.4	839	83.95	60.7	72.8

Note: Source: Primary Census Abstract, Series 20, Census of India, 2001. [1] 1991-2001, [2] Females per 1,000 males.

CHAPTER II

BACKGROUND CHARACTERISTICS OF HOUSEHOLD

This chapter provides a socio-economic and demographic profile of households interviewed in the District Level Household Survey-Reproductive and Child Health. Facilities and services such as Health, Education and Communication available in the representative sampled village are also presented here. The *de facto* producer of enumeration is adopted in order to include every individual staying in the sampled Primary Sampling Units (PSU), either a village or an urban area, the night before the survey. The objective of adopting the *de facto* method is to avoid duplication of persons who are in transit.

2.1 Age – Sex Structure

The age-sex distribution of sampled household population classified by residence is presented in Table 2.1. The percent distribution is based on sampled *de facto* population of 1,13,103 persons of whom 71 percent lived in the rural areas of Haryana. The state of Haryana depicts a young and growing population with 35 percent below the age of 15 years (Figure 2.1). There are more children below 15 years recorded in rural areas (36 percent) compared to those in urban areas (31 percent).

Figure 2.1
Age-Sex Pyramid

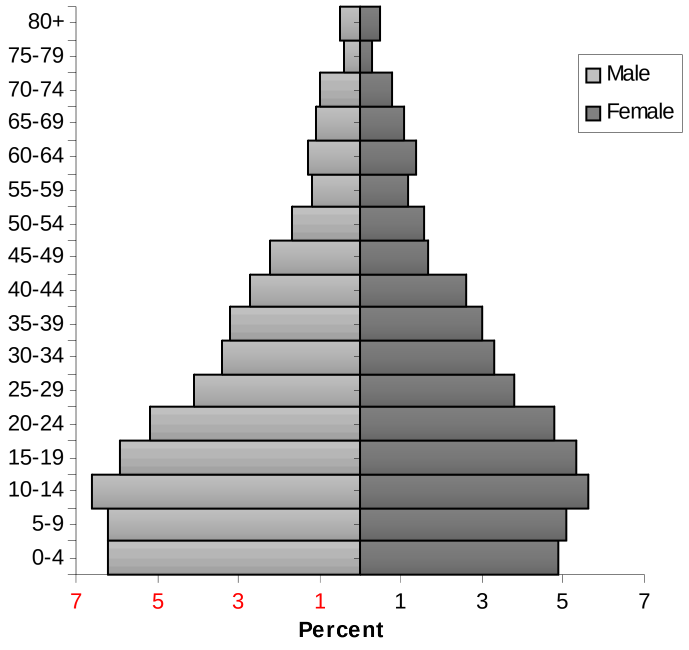

Haryana, DLHS-RCH, 2002-04

The overall sex ratio of 113 males per 100 females is recorded for the *de facto* population. The sex ratio is 113 both in rural and urban areas.

Table 2.1 HOUSEHOLD POPULATION BY AGE AND SEX

Percent distribution of the household population by age and by residence and sex, Haryana, 2002-04

Age	Total			Rural			Urban		
	Total	Male	Female	Total	Male	Female	Total	Male	Female
< 1	2.2	2.4	2.1	2.3	2.5	2.2	2.0	2.1	1.8
1-4	8.8	9.3	8.3	9.2	9.8	8.6	7.8	8.0	7.6
5-9	11.3	11.6	10.9	11.9	12.1	11.7	9.9	10.6	9.1
10-14	12.2	12.4	11.9	12.4	12.4	12.4	11.6	12.4	10.6
15-19	11.2	11.1	11.3	11.3	11.2	11.3	11.1	10.9	11.4
20-24	10.0	9.8	10.2	9.9	9.8	10.0	10.2	9.8	10.7
25-29	8.0	7.8	8.2	7.9	7.8	7.9	8.2	7.7	8.7
30-34	6.7	6.4	7.0	6.5	6.1	6.9	7.2	6.9	7.5
35-39	6.2	6.1	6.3	5.8	5.8	5.9	7.1	6.9	7.3
40-44	5.3	5.2	5.5	4.9	4.8	5.1	6.2	6.2	6.2
45-49	3.9	4.2	3.6	3.6	3.9	3.2	4.7	4.8	4.6
50-54	3.3	3.3	3.3	3.1	3.0	3.2	3.8	4.0	3.5
55-59	2.5	2.3	2.6	2.3	2.2	2.5	2.7	2.6	2.9
60-64	2.7	2.5	2.9	2.8	2.5	3.1	2.4	2.4	2.5
65-69	2.2	2.0	2.4	2.3	2.2	2.5	1.9	1.6	2.3
70-74	1.8	1.8	1.7	1.9	2.0	1.8	1.6	1.5	1.6
75-79	0.7	0.8	0.7	0.7	0.8	0.7	0.8	0.8	0.8
80+	1.0	1.0	1.0	1.1	1.1	1.1	0.8	0.8	0.9
Total percent	100.0	100.0	100.0	100.0	100.0	100.0	100.0	100.0	100.0
Number of persons	1,13,103	59,929	53,174	79,761	42,259	37,502	33,342	17,669	15,672
Sex ratio[1]	113	NA	NA	113	NA	NA	113	NA	NA

Note: Table is based on the *de facto* population, i.e. persons who stayed in the household the night before the interview (including both usual resident and visitors). NA: not Available.[1] Male per 100 females.

2.2 Household Characteristics

The percent distribution of 20,205 households surveyed in the state of Haryana by selected characteristics of the household head and the number of usual household members are shown in Table 2.2. This is based on *de jure*, the usual resident population. Ninety four percent of household heads are male; while only 6 percent are female-headed households. Nearly 69 percent of household heads are in the 30-59 years age group. The median age of household heads is 44 years for the state as a whole. About 11 percent of household heads are younger than 30 years and 20 percent are at least 60 years old. Majority of the household heads are Hindus (90 percent), 5 percent each are Sikh and Muslim. Hindus constitute a higher proportion of population in urban areas (92 percent) than in rural areas (89 percent). A total of 6 percent of rural households are Muslim compared to 2 percent of the urban households.

Table 2.2 HOUSEHOLD CHARACTERISTICS

Percent distribution of the household head by selected characteristics of the household head and household size, according to residence, Haryana, 2002-04

Characteristic	Total	Residence	
		Rural	Urban
**Sex of the household head**			
Male	93.7	94.1	92.8
Female	6.3	5.9	7.2
**Age of the household head**			
< 30	11.3	12.4	8.8
30-44	40.3	39.8	41.5
45-59	28.4	26.8	31.8
60+	20.0	21.0	17.8
Median age of the household head	44.2	44.0	44.7
**Religion of the household head**			
Hindu	90.1	89.1	92.3
Muslim	4.7	5.8	2.1
Christian	0.1	0.1	0.2
Sikh	4.9	4.9	4.9
Buddhist	0.0	0.0	0.0
**Caste/tribe of the household head**			
Scheduled caste	22.9	26.5	15.1
Scheduled tribe	0.6	0.6	0.4
Other backward class	29.5	31.6	24.9
Other #	47.0	41.2	59.4
Don't know	0.0	0.0	0.1
**Number of usual members**			
1	1.4	1.2	1.8
2	4.8	4.5	5.3
3	8.4	7.7	10.1
4	19.3	17.7	22.7
5	22.2	21.6	23.6
6	17.0	17.3	16.2
7	10.1	11.1	7.8
8	6.1	6.7	4.7
9+	10.8	12.1	7.9
Mean household size	5.4	5.6	5.1
Total percent	100.0	100.0	100.0
Number of households	20,205	13,832	6,373

Note: Table is based on the *de jure* population
Higher caste (Not belonging to a scheduled caste, a scheduled tribe and an other backward class)

Thirty percent of the households in Haryana belong to other backward classes, 23 percent to schedule caste, while the remaining 47 percent of the households are headed by other castes not under schedule caste, schedule tribe and other backward classes. Slightly more than one-fourth of the household head belong to schedule caste in rural areas and it is only 15 percent in urban areas. The overall state average household size is 5.4 persons. The rural-urban differential in average household size is 5.6 in rural areas and 5.1 in urban areas.

2.3 Educational Level

The educational background of Haryana presented in this section is based on *de facto* household population. Level of literacy and years of schooling, according to age, sex and residence are shown in Table 2.3.

Table 2.3 EDUCATIONAL LEVEL OF THE HOUSEHOLD POPULATION

Percent distribution of household population age 7 and above by literacy level and years of schooling, according to age , residence and sex, Haryana, 2002-04

Age	Non-literate	Literate but no schooling	Years of schooling				Missing	Total Percent	Number of persons
			1-5	6-8	9-10	11 or more			
**Total**									
Male									
7-9	13.4	0.0	85.3	0.9	0.0	0.0	0.3	100.0	4,091
10-14	5.3	0.0	47.2	41.1	6.1	0.0	0.4	100.0	7,456
15-19	6.2	0.0	9.3	25.8	32.0	26.6	0.0	100.0	6,676
20-29	11.0	0.0	8.9	21.2	24.1	34.9	0.0	100.0	10,562
30-39	20.4	0.0	10.9	19.3	25.3	24.0	0.0	100.0	7,471
40-49	26.0	0.0	10.8	17.8	28.1	17.4	0.0	100.0	5,610
50+	46.3	0.0	12.0	11.5	19.5	10.7	0.0	100.0	8,199
Total	18.6	0.0	21.9	20.9	20.4	18.2	0.1	100.0	50,065
Female									
7-9	16.1	0.1	82.8	0.7	0.0	0.0	0.3	100.0	3,416
10-14	9.8	0.0	43.0	40.7	6.1	0.0	0.5	100.0	6,316
15-19	14.9	0.0	12.1	23.2	25.6	24.0	0.0	100.0	6,012
20-29	29.8	0.0	12.3	17.9	17.1	22.8	0.0	100.0	9,781
30-39	55.3	0.0	13.4	10.3	10.9	10.0	0.0	100.0	7,093
40-49	63.1	0.0	11.7	8.2	9.3	7.7	0.0	100.0	4,820
50+	83.3	0.0	7.9	3.3	2.8	2.7	0.0	100.0	7,835
Total	40.8	0.0	21.2	15.8	11.1	11.0	0.1	100.0	45,273
Total									
7-9	14.6	0.0	84.2	0.8	0.0	0.0	0.3	100.0	7,507
10-14	7.3	0.0	45.3	40.9	6.1	0.0	0.4	100.0	13,773
15-19	10.3	0.0	10.7	24.6	29.0	25.4	0.0	100.0	12,688
20-29	20.0	0.0	10.5	19.6	20.7	29.1	0.0	100.0	20,343
30-39	37.4	0.0	12.1	14.9	18.3	17.2	0.0	100.0	14,564
40-49	43.1	0.0	11.2	13.3	19.4	12.9	0.0	100.0	10,430
50+	64.4	0.0	10.0	7.5	11.3	6.8	0.0	100.0	16,034
Total	29.1	0.0	21.6	18.4	16.0	14.8	0.1	100.0	95,338

Note: Table is based on *de facto* population.

Contd…

Table 2.3 indicates that, 29 percent of the population aged seven and above are non-literate. The proportion of non-literates is 41 percent for females compared to 19 percent for males. The proportion of non-literate is much higher among the older cohorts compared to the younger ones. For both males and females, going by expected trend, the level of literacy is higher in the younger population than in the older age groups with the exception of the youngest age group of 7-9 years (Figure 2.2).

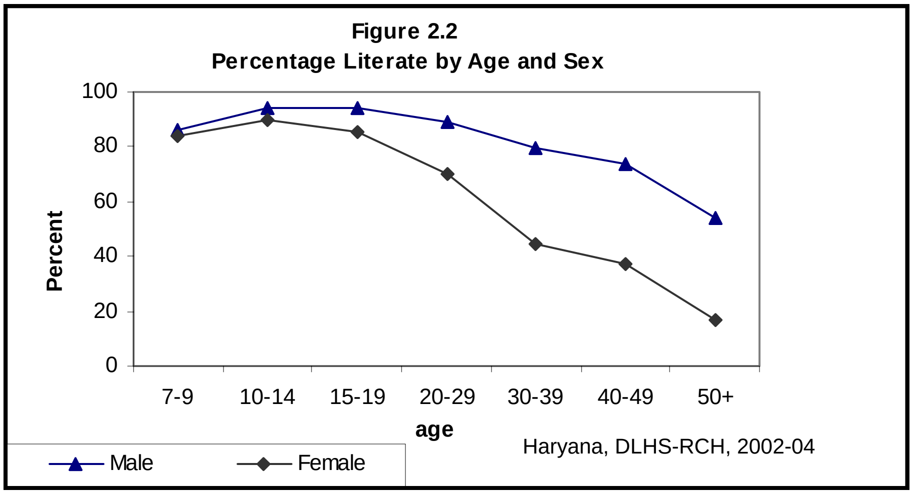

Table 2.3 EDUCATIONAL LEVEL OF THE HOUSEHOLD POPULATION

Percent distribution of household population age 7 and above by literacy level and years of schooling, according to age , residence and sex, Haryana, 2002-04

Age	Non-literate	Literate but no schooling	Years of schooling					Total Percent	Number of persons
			1-5	6-8	9-10	11 or more	Missing		
**RURAL**									
Male									
7-9	14.5	0.0	84.2	1.0	0.0	0.0	0.3	100.0	2,970
10-14	5.3	0.0	48.9	40.0	5.5	0.0	0.4	100.0	5,259
15-19	6.8	0.0	9.9	27.8	32.2	23.3	0.0	100.0	4,750
20-29	12.6	0.0	9.8	23.1	24.6	29.8	0.0	100.0	7,466
30-39	24.6	0.0	12.0	20.7	25.1	17.6	0.0	100.0	5,029
40-49	32.4	0.0	11.6	19.4	26.9	9.7	0.0	100.0	3,674
50+	55.7	0.0	11.7	11.4	15.9	5.3	0.0	100.0	5,792
Total	21.8	0.0	22.8	21.7	19.6	14.0	0.1	100.0	34,937
Female									
7-9	17.8	0.0	81.1	0.8	0.0	0.0	0.3	100.0	2,567
10-14	11.6	0.0	44.9	38.0	5.3	0.0	0.2	100.0	4,650
15-19	17.8	0.0	14.7	25.5	24.6	17.2	0.0	100.0	4,228
20-29	35.9	0.0	14.2	20.3	16.4	13.1	0.0	100.0	6,740
30-39	67.1	0.0	14.5	9.2	6.5	2.8	0.0	100.0	4,784
40-49	77.0	0.0	11.8	5.9	4.3	1.0	0.0	100.0	3,123
50+	92.5	0.0	5.3	1.2	0.7	0.3	0.0	100.0	5,562
Total	47.2	0.0	22.4	15.6	9.1	5.7	0.1	100.0	31,654
Total									
7-9	16.0	0.0	82.8	0.9	0.0	0.0	0.3	100.0	5,537
10-14	8.2	0.0	47.0	39.0	5.4	0.0	0.3	100.0	9,909
15-19	12.0	0.0	12.2	26.7	28.7	20.5	0.0	100.0	8,977
20-29	23.7	0.0	11.9	21.8	20.7	21.9	0.0	100.0	14,205
30-39	45.3	0.0	13.2	15.1	16.0	10.4	0.0	100.0	9,812
40-49	52.9	0.0	11.7	13.2	16.5	5.7	0.0	100.0	6,797
50+	73.7	0.0	8.5	6.4	8.5	2.8	0.0	100.0	11,353
Total	33.9	0.0	22.6	18.8	14.6	10.0	0.1	100.0	66,592

Contd...

Eighty five percent of males in this age group had 1-5 years of schooling. Eighty three percent of females of this age group have had education for 1-5 years. Lesser proportion of females are found in higher education of 9-10 years and 11 or more years (11 percent each) compared to the males having corresponding figures of 20 percent and 18 percent respectively. In Haryana not even a single person found to be literate without any formal schooling.

Table 2.3 EDUCATIONAL LEVEL OF THE HOUSEHOLD POPULATION

Percent distribution of household population age 7 and above by literacy level and years of schooling, according to age , residence and sex, Haryana, 2002-04

Age	Non-literate	Literate but no schooling	Years of schooling					Total Percent	Number of persons
			1-5	6-8	9-10	11 or more	Missing		
**URBAN**									
Male									
7-9	10.6	0.0	88.2	0.7	0.0	0.0	0.4	100.0	1,121
10-14	5.3	0.0	43.1	43.7	7.5	0.0	0.4	100.0	2,198
15-19	4.8	0.0	8.0	20.9	31.5	34.8	0.0	100.0	1,927
20-29	6.9	0.0	6.8	16.7	22.7	46.9	0.0	100.0	3,096
30-39	11.8	0.0	8.8	16.5	25.6	37.3	0.0	100.0	2,443
40-49	13.7	0.0	9.4	14.7	30.2	31.9	0.0	100.0	1,936
50+	23.8	0.0	12.8	11.7	27.9	23.8	0.0	100.0	2,407
Total	11.0	0.0	19.9	18.9	22.2	27.9	0.1	100.0	15,127
Female									
7-9	10.9	0.1	88.0	0.5	0.0	0.0	0.5	100.0	848
10-14	4.6	0.0	37.7	48.3	8.2	0.0	1.2	100.0	1,666
15-19	8.1	0.0	6.0	17.7	28.1	40.2	0.0	100.0	1,784
20-29	16.3	0.0	8.0	12.7	18.7	44.2	0.0	100.0	3,041
30-39	30.8	0.0	11.2	12.7	20.2	25.1	0.0	100.0	2,309
40-49	37.6	0.0	11.4	12.4	18.6	20.1	0.0	100.0	1,697
50+	60.7	0.0	14.3	8.4	7.8	8.8	0.0	100.0	2,273
Total	26.0	0.0	18.4	16.2	15.9	23.4	0.2	100.0	13,619
Total									
7-9	10.7	0.1	88.1	0.6	0.0	0.0	0.4	100.0	1,970
10-14	5.0	0.0	40.7	45.7	7.8	0.0	0.7	100.0	3,864
15-19	6.4	0.0	7.0	19.3	29.8	37.4	0.0	100.0	3,711
20-29	11.6	0.0	7.4	14.7	20.7	45.6	0.0	100.0	6,138
30-39	21.0	0.0	9.9	14.7	23.0	31.3	0.0	100.0	4,752
40-49	24.9	0.0	10.4	13.6	24.8	26.4	0.0	100.0	3,633
50+	41.7	0.0	13.5	10.1	18.1	16.5	0.0	100.0	4,680
Total	18.1	0.0	19.2	17.6	19.2	25.8	0.1	100.0	28,746

An examination of the educational attainment by place of residence revealed that the urban-rural differential was quite pronounced. In urban areas, only 18 percent of the total population is non-literate in comparison to 34 percent of the rural population. The numbers of non-literate females live in rural areas of Haryana accruing a share as high as 47 percent, while non-literate rural males is 22 percent. Prevalence of illiterate is much less in urban areas with figures of 26 percent and 11 percent non-literate females and males respectively. A contrasting feature of rural-urban difference in educational level is that in rural areas most people had 1-5 years of schooling (23 percent) and those who had 11 or more years of schooling was just 10 percent, whereas in urban areas a significant proportion of people (26 percent) had this level of education.

2.4 Marital Status of the Household Population

The DLHS, collected information on the marital status of all household members aged 10 years and above. Table 2.4 shows the percent distribution of household population by marital status distribution of *de facto* household population by age and sex. Ninety four percent in the age group 30-44 years, followed by 90 percent in the age group 45-59 years and 87 percent in the age group 25-29 years are currently married. The proportion of never married for both males and female is 35 percent in the state, and it is higher for males (41 percent) than for females (28 percent). The proportion of never married among males declines with increasing age and reaches the lowest by the time they are in the age group 45-59 years. A similar pattern has been observed in the case of females, with the lowest never married proportion for the age group 45-49 years. The proportions of divorced, separated or widowed are negligible and limited to the older ages. Forty five percent of women aged 60 years or above are widowed /divorced /separated. Among the *de facto* population aged 10 years and above, 56 percent of males and 63 percent of females are currently married.

Table 2.4 MARITAL STATUS OF THE HOUSEHOLD POPULATION

Percent distribution of the household population aged 10 years and above by marital status, according to age and sex, Haryana, 2002-04

Age	Marital status				Total Percent	Number of persons
	Never married	Currently married	Married, gaunna not performed	Widowed/ divorced/ Separated		
			Male			
10-14	99.6	0.3	0.0	0.1	100.0	7,456
15-19	94.8	3.5	1.7	0.1	100.0	6,676
20-24	61.4	36.8	1.3	0.5	100.0	5,884
25-29	19.7	78.9	0.2	1.2	100.0	4,678
30-44	3.4	94.6	0.0	1.9	100.0	10,572
45-59	1.3	94.6	0.0	4.2	100.0	5,858
60+	1.5	81.7	0.0	16.7	100.0	4,850
Total	40.9	55.7	0.5	3.0	100.0	45,974
			Female			
10-14	99.5	0.2	0.2	0.0	100.0	6,316
15-19	72.6	26.0	1.2	0.2	100.0	6,012
20-24	19.6	79.2	0.3	0.9	100.0	5,437
25-29	2.4	96.3	0.1	1.3	100.0	4,344
30-44	0.4	94.0	0.0	5.6	100.0	9,995
45-59	0.2	83.9	0.1	15.8	100.0	5,076
60+	0.3	54.2	0.1	45.4	100.0	4,678
Total	28.4	62.7	0.3	8.6	100.0	41,857
			Total			
10-14	99.6	0.2	0.1	0.0	100.0	13,773
15-19	84.3	14.2	1.4	0.1	100.0	12,688
20-24	41.3	57.2	0.8	0.7	100.0	11,321
25-29	11.4	87.3	0.1	1.3	100.0	9,022
30-44	1.9	94.3	0.0	3.7	100.0	20,566
45-59	0.8	89.6	0.0	9.6	100.0	10,934
60+	0.9	68.2	0.1	30.8	100.0	9,528
Total	34.9	59.1	0.4	5.6	100.0	87,831

Note: Table is based on *de facto* population

2.5 Marriage

Marriage in the household is an important event that reflects the socio-cultural practices of the communities surveyed in DLHS. This section outlines the marriages ceremonies during the three years period prior to the survey. Mean age at marriage by sex and percentage of total marriages which are below legal age at marriage, 21 years for boys and 18 years for girls by resident at the state and at district levels are shown in Table 2.5.

Table 2.5 MARRIAGE

Mean age at marriage and percentage of marriages below legal at marriage by sex and by districts, Haryana, 2002-04

Place of residence/ District	Mean age at marriage		Percentage of marriage below legal age at marriage	
	Boy	Girl	Boy (<21)	Girl (<18)
**State – Total**	22.7	19.0	29.0	27.8
**State – Rural**	22.1	18.3	33.2	33.7
**State – Urban**	24.2	20.7	17.7	11.9
**District**				
Ambala	24.4	20.9	12.2	1.6
Bhiwani	22.2	17.9	32.6	45.1
Faridabad	21.5	18.9	41.3	33.0
Fatehabad	22.5	19.5	28.0	23.6
Gurgaon	21.4	17.5	48.6	49.1
Hisar	23.1	18.5	25.3	34.5
Jhajjar	23.4	19.0	19.8	21.9
Jind	22.2	18.6	34.3	26.6
Kaithal	22.0	18.9	33.6	31.3
Karnal	22.7	19.9	25.7	15.6
Kurukshetra	23.0	20.7	21.0	6.0
Mahendragarh	21.8	17.8	36.5	42.5
Panchkula	24.1	20.9	15.8	11.9
Panipat	22.5	18.7	26.8	29.4
Rewari	22.9	19.4	23.2	16.5
Rohtak	23.8	19.0	17.7	26.8
Sirsa	22.6	19.2	33.8	23.7
Sonipat	23.6	18.8	21.8	25.8
Yamunanagar	23.3	20.2	20.7	14.5

Note: Table based on *de jure* population.
Reference period: - January 1st, 1999 to survey date for phase-1, and January 1st, 2001 to survey date for phase-2.

Mean age at marriage for boys and girls in urban areas of Haryana are 24 years and 21 years respectively. The corresponding figures in rural areas are 22 years and 18 years. On the whole, as far as Haryana is concerned, both boys and girls seem to oblige the legal age marriage, the average age at marriage being 23 years for boys and 19 years for girls. However, over one-fourth of the boys and girls got married below the corresponding specified legal age marriage. The proportion is much higher in the rural areas compared to the urban areas of the state.

When it comes to district level variation in mean age at marriage, it is highest (24 years) in Ambala, Panchkula, Rohtak and Sonipat for boys and 21 years for girls in Ambala, Kurukshetra and Panchkula. The lowest mean age at marriage for boys is 21 years recorded for the district of Gurgaon and for the girls; the lowest is 18 years in Bhiwani, Gurgaon, and Mahenderagarh.

It is also found that, the percentage of girls who were married below the legal age at marriage was the highest in Gurgaon (49 percent) and the lowest in Ambala (2 percent). In 10 out of 19 districts more than 25 percent girls were marrying below the legal age at marriage (see Map-1). In the case of boys, marriages below the legal age at marriage are the highest in Gurgaon district (49 percent) and lowest in Ambala (12 percent).

2.6 Morbidity Rates

The DLHS-RCH has collected information on the morbidity status relating to blindness, tuberculosis and malaria of the *de jure* members of the household. Table 2.6 provides prevalence rates.

Table 2.6 MORBIDITY RATES

Prevalence of blindness, tuberculosis, and malaria, according to place of residence, Haryana, 2002-04.

Morbidity	Total	Residence	
		Rural	Urban
Prevalence rate of blindness			
Male			
Partial	3,849	4,001	3,483
Complete	146	150	135
Night blindness	131	168	43
Female			
Partial	6,110	6,324	5,600
Complete	182	216	101
Night blindness	165	200	81
Persons			
Partial	4,906	5,085	4,478
Complete	163	181	119
Night blindness	147	183	61
Prevalence rate of tuberculosis			
Male	306	341	223
Female	250	275	191
Person	280	310	208
Prevalence rate of malaria[1]			
Male	345	394	227
Female	338	362	283
Person	342	379	253

Note: All the rates refer to *de jure* population.
Prevalence rate per 100, 000 population
Reference period: - January 1st, 1999 to survey date for phase-1, and January 1st, 2001 to survey date for Phase-2. [1] Last two weeks prior to the survey

Partial, Complete and Night Blindness

The overall prevalence of partial blindness is 4,906 per 100,000 populations in the state and is lower in urban areas (4,478 per 100,000) than in rural areas (5,085 per 100,000). It is more among females. The prevalence of complete blindness is 163 per 100,000 populations with a rural-urban differential of 181 against 119 per 100,000. Sex differential is also significant in complete blindness. The prevalence of night blindness due to vitamin A deficiency is 147 per 100,000 populations and is higher in rural areas (183) than in urban areas (61).

Tuberculosis

The prevalence of tuberculosis is 280 per 100,000 populations, with rural areas having a higher prevalence of 310 compared to 208 per 100,000 in urban areas. The prevalence of TB is higher among males (306 per 100,000) than among females (250 per 100,000).

Malaria

In the DLHS-RCH, household respondents were asked to state whether any member of their household suffered from malaria (characterized by recurrent fever with shivering) any time during the two weeks prior the survey. In the state of Haryana, 342 persons per 100,000 populations were reported to have suffered from malaria. Rural residents are more likely to suffer from malaria (379 per 100,000) than urban residents (253 per 100,000). The reported prevalence of malaria is higher for males than for females.

2.7 Morbidity Rates by Districts

Table 2.7 shows the prevalence of blindness, tuberculosis and malaria in the districts of Haryana. The prevalence of partial blindness varies considerably among the districts the lowest being 358 per 100,000 in Ambala and the highest, 7,722 per 100,000 in Gurgaon. The district with a prevalence rate below 1,000 per 100,000 is Ambala.

Table 2.7 MORBIDITY RATES BY DISTRICTS

Prevalence of blindness, tuberculosis, and malaria, by district, Haryana, 2002-04

District	Prevalence[1] of morbidity			
	Partial blindness	Complete blindness	Tuberculosis	Malaria[2]
Ambala	358	209	197	151
Bhiwani	7,681	154	226	198
Faridabad	4,376	174	256	305
Fatehabad	4,500	275	389	570
Gurgaon	7,722	139	345	146
Hisar	1,132	35	95	50
Jhajjar	6,684	93	162	218
Jind	3,944	115	291	404
Kaithal	7,656	368	456	603
Karnal	6,219	284	309	836
Kurukshetra	2,420	52	242	183
Mahendragarh	3,299	285	242	353
Panchkula	4,667	89	76	199
Panipat	7,378	144	358	182
Rewari	5,513	277	422	350
Rohtak	6,675	198	329	554
Sirsa	3,224	122	205	303
Sonipat	5,709	83	388	756
Yamunanagar	1,876	82	266	242
Haryana	4,906	163	280	342

Note: All the rates refer to *de jure* population.
[1] Prevalence rate per 100, 000 population
Reference period: - January 1st, 1999 to survey date for phase-1, and January 1st, 2001 to survey date for Phase-2. [2] Last two weeks prior to the survey

The prevalence rate of complete blindness ranges from 35 per 100,000 in Hisar to 368 per 100,000 in Kaithal. Inter-district variations are substantial for tuberculosis and malaria. The prevalence rate of tuberculosis is the highest in Kaithal district (456 per 100,000 population) and it is lowest in Panchkula (76 per 100,000). In the case of malaria, the prevalence rate is highest in Karnal (836 per 100,000) and lowest in Hisar (50 per 100,000).

2.8 Housing Characteristics

This section describes the availability of basic amenities in the state. Table 2.8 presents the percent distribution of households by selected housing characteristics. Ninety one percent of the households in Haryana have electricity connection and it is more in urban areas (98 percent) than in rural areas (88 percent).

As regards household source of drinking water around two-third (63 percent) of the households get drinking water through taps, while 28 percent drink water from hand pumps/ bore-wells and one percent drink water from wells. About 84 percent of households in urban areas get piped water for drinking, whereas in rural areas only 53 percent of the households have such provision.

When it comes to sanitation facility, only 23 percent of the households have flush toilets, while 24 percent have pit based toilets or latrines, one percent depend on shared toilets and nearly 51 percent of the households have no toilet facility at all. There is a large rural-urban difference; 69 percent of rural households have no toilet facility, compared to just 13 percent of urban households.

DLHS-RCH has also collected data on type of fuel used in the households for cooking. Thirty two percent of the households used liquid petroleum gas or electricity for cooking in Haryana. About 54 percent of households rely on firewoods, 2 percent on kerosene and 13 percent of households use other types of fuel for cooking. The use of liquid petroleum gas/electricity for cooking is reported more in urban areas (73 percent) and firewood or other sources for cooking are reported more in rural areas (70 percent).

There is considerable variation in the quality of housing. On the basis of building material, type of floor, walls and roof, households are categorised into *kachcha*, semi-*pucca* and *pucca*. Less than half of the households are living in semi-*pucca* houses, 45 percent in *pucca* houses and 8 percent in *kachcha* houses. Seventy nine percent of urban households live in *pucca* houses compared to 30 percent of rural households.

The possession of consumer durable goods is an indication of a household’s socio-economic status. Table 2.8 shows that almost all the households in the state own electric fan (90 percent), television (66 percent), bicycles (57 percent), radio/transistor (31 percent) and telephone (22 percent)

Table 2.8 HOUSING CHARACTERISTICS

Percent distribution of the household by housing characteristics and percentage of households owing selected durable goods, according to residence, Haryana, 2002-04

Housing characteristic	Total	Residence	
		Rural	Urban
**Electricity**			
Yes	91.2	88.2	97.8
No	8.8	11.8	2.2
**Source of drinking water**			
Tap inside	36.5	23.0	66.0
Tap shared public	26.1	29.8	18.0
Hand pump/ bore well	28.1	34.3	14.7
Well covered	1.0	1.4	0.1
Well uncovered	7.1	10.3	0.3
River	0.1	0.2	0.0
Pond	0.0	0.0	0.0
Spring	0.1	0.1	0.0
Other	0.9	0.9	0.8
**Sanitation facility**			
Own flush toilet	23.3	5.6	61.8
Own pit toilet / latrine	23.9	24.3	23.1
Shared toilet of any type	1.1	0.8	1.9
Public / community toilet	0.4	0.3	0.7
No toilet facility	51.3	69.1	12.6
**Main type of fuel used for cooking**			
Liquid petroleum gas/ electricity	31.9	13.0	73.0
Kerosene	1.7	0.8	3.8
Wood	53.5	69.6	18.7
Other	12.8	16.7	4.4
**Type of house**			
Kachcha	8.0	10.5	2.5
Semi - pucca	46.7	59.7	18.6
Pucca	45.3	29.9	78.9
**Household assets**			
Fan	90.1	86.6	97.5
Radio/transistor	31.0	28.7	35.9
Sewing machine	56.8	50.3	70.8
Television	65.7	56.5	85.8
Telephone	22.1	13.7	40.3
Bicycle	57.2	53.1	65.9
Motor cycle/ scooter	23.3	16.5	38.1
Car / Jeep	4.2	2.1	8.5
Tractor	5.7	7.8	1.1
**Standard of living index**			
Low	19.3	26.6	3.5
Medium	43.5	52.0	25.2
High	37.2	21.4	71.3
**Number of households**	20,205	13,832	6,373

Other durable goods found in the surveyed households are sewing machine (57 percent), motorcycle or scooter (23 percent). Car/jeep and tractor are owned by 4 percent and 6 percent of households in Haryana respectively. Ownership of most of the consumer durable items is more among the urban households than among the rural households. However, a higher proportion of households in rural areas than in urban areas own a tractor.

Considering household amenities, such as, source of drinking water, type of house, source of lighting, fuel for cooking, toilet facility and ownership of durable goods a composite measure, standard of living index (SLI) is made for classification of households. The standard of living index is calculated as by adding the following scores:

Source of drinking water: 3 for Tap (own), 2 for Tap (shared), 1 for hand pump and well, and 0 for other;

Type of house: 4 for *pucca*, 2 for semi-*pucca*, and 0 for *kachcha*;

Source of lighting: 2 for electricity, 1 for kerosene, and 0 for other;

Fuel for cooking: 2 for LPG gas/electricity, 1 for kerosene and 0 for other;

Toilet facility: 4 for own flush toilet, 2 for own pit toilet, 2 for shared toilet and 0 for no toilet;

Ownership for items: 4 each for car and tractor, 3 each for television, telephone and motorcycle/scooter, and 2 each for fan, radio/transistor, sewing machine and bicycle.

The total of the scores may vary from the lowest of a 0 to maximum of 40. On the basis of total score, households are divided into three categories as;

a) Low – if total score is less than or equal to 9,
b) Medium – if total score is greater than 9 but less than or equal to 19 and
c) High – if total score is greater than 19.

As per the standard of living index, nearly half of the households (44 percent) come under the medium standard of living category, 37 percent of households to high standard of living and 19 percent of the households to low standard of living.

The proportion of sample households with high standard of living is higher in urban areas than in rural areas and the proportion of households with a low and medium standard of living is much higher in rural households (79 percent) than in urban households (29 percent) in the state of Haryana.

2.9 Housing Characteristics by Districts

The 19 districts in Haryana are not uniform in terms of basic amenities and possession of consumer durables. Table 2.9 presents an inter-district comparison of housing characteristics. The percentage of households with electricity is lowest in the district Gurgaon (78 percent) and the household with electricity is highest in Ambala and Kurukshetra (98 percent). Ninety percent or more of households used piped water or water from a hand pump for drinking in most of the districts except for Bhiwani (73 percent), Gurgaon (79 percent), Jhajjar (81 percent), Jind (85 percent) and Rohtak (86 percent).

Largely the districts in Haryana have inadequate toilet facility, in 9 of the 19 districts less than half of the households have toilet facilities and it is the least in Gurgaon district (33 percent).

In Ambala and Faridabad districts the percentage of households using liquid petroleum gas/electricity for cooking is 45 percent and in the rest of the districts, it is relatively low ranging between 22 to 42 percent. The percentage of households living in *pucca* houses is quite low in all the districts of Haryana. In 12 of the19 districts, less than half of the households live in *pucca* houses. In Faridabad 68 percent of the households live in *pucca* houses.

Table 2.9 HOUSING CHARACTERISTICS BY DISTRICT

Selected housing characteristics by district, Haryana, 2002-04

Districts	Percentage of households				
	With electricity	With drinking water[1]	With toilet facility	Using Liquid petroleum gas/ electricity	Living in *pucca* house
Ambala	97.7	99.8	51.2	45.3	39.8
Bhiwani	93.2	73.3	45.7	27.6	51.5
Faridabad	90.5	97.6	60.8	45.2	68.4
Fatehabad	91.8	99.3	60.4	31.6	37.0
Gurgaon	77.5	79.1	33.2	23.2	43.2
Hisar	92.9	91.8	50.1	27.9	30.5
Jhajjar	92.1	80.8	38.7	24.3	49.5
Jind	93.0	84.4	34.0	24.9	41.1
Kaithal	92.7	95.9	36.3	21.6	32.3
Karnal	94.8	99.5	54.0	36.0	42.5
Kurukshetra	97.9	99.8	53.3	39.0	37.0
Mahendragarh	83.2	90.0	37.2	25.5	51.3
Panchkula	90.5	91.2	48.1	41.7	60.7
Panipat	96.0	99.2	52.5	41.2	49.9
Rewari	93.0	95.4	44.9	30.2	45.1
Rohtak	95.2	86.0	52.6	32.9	48.3
Sirsa	81.7	97.5	75.3	26.7	37.5
Sonipat	93.9	96.4	51.9	32.8	52.5
Yamunanagar	93.0	97.6	44.8	34.4	37.2
Haryana	91.2	91.7	48.7	31.9	45.3

Note: [1]That is piped or from a hand pump / bore well.

2.10 Iodization of Salt

Consumption of salt fortified with iodine is recommended to avoid miscarriages, brain disorders, cretinism and retarded psychomotor development. As per the Prevention of Food Adulteration Act, 1988, the minimum iodine content of edible salt is 30 parts per million (PPM) at the manufacturing level.

In the DLHS-RCH survey, each interviewer was provided with a test kit to measure the level of iodine content of salt consumed by the surveyed households. The test results (Table 2.10) are classified by degree of ionization of salt and categorised by background characteristics. It is observed that nearly 55 percent of households used salt that contained a minimum recommended 15 ppm or higher level of iodine content whereas 26 percent of households used salt that is not iodized at all and another 18 percent used salt, which was inadequately iodized.

In rural areas, 34 percent of households against 9 percent in urban areas used non-iodized salts. Percentage of households using inadequately iodized salt in rural areas is one and half times higher compared to that in urban areas. Number of households using non-iodized or inadequately iodized salt is closely associated with the educational level of the household head. Seventy one percent of households headed by persons who had more than 10 years of schooling reported the use of adequately iodized salts. Consumption of adequately iodised salt among households of other caste is 63 percent, followed by 51 percent each in other backward class and scheduled tribe households and among scheduled caste it is about 46 percent of households.

Table 2.10 IODIZATION OF SALT

Percent distribution of household heads by degree of Iodization of salt, according to selected background characteristics, Haryana, 2002-04

Background characteristic	Not Iodised	7ppm	15+ppm	Other[1]	Total percent	Number of households
**Place of Residence**						
Rural	34.3	19.6	45.2	0.9	100.0	13,832
Urban	9.0	12.8	77.4	0.8	100.0	6,373
**Education of the household heads**						
Non-literate	35.7	21.3	41.7	1.2	100.0	6,738
0-9@ years	28.5	18.3	52.5	0.7	100.0	6,444
10 and above	15.3	13.0	71.0	0.7	100.0	7,022
**Religion of household head**						
Hindu	26.3	17.1	55.7	0.9	100.0	18,206
Muslim	42.3	24.4	32.2	1.1	100.0	941
Sikh	12.5	18.2	68.5	0.8	100.0	995
Other	8.4	9.8	80.1	1.7	100.0	64
**Caste/tribe of the household head#**						
Scheduled caste	32.9	19.7	46.3	1.1	100.0	4,632
Scheduled tribe	25.1	21.2	51.1	2.6	100.0	115
Other backward class	29.6	18.9	50.5	1.0	100.0	5,961
Other	21.0	15.4	62.8	0.7	100.0	9,488
**Standard of living index**						
Low	47.0	22.0	29.7	1.3	100.0	3,903
Medium	30.4	19.9	48.8	0.9	100.0	8,793
High	10.7	12.2	76.4	0.7	100.0	7,509
Total	26.3	17.5	55.3	0.9	100.0	20,205

Note: Ppm: Parts per million. @ Literate persons with no years of schooling are also included. # Total number of cases may not add up to N due to do not know and missing cases. [1] Includes salt not at home, salt not tested, refused and missing cases.

Differential in the consumption of properly iodized salt is more pronounced when analysed by religion of the household head and standard of living index. Percentage of households using adequately iodized salt is only 32 percent among Muslim households, whereas the corresponding figures for Sikh and Hindu households are 69 percent and 56 percent respectively. Again, households with low standard of living are more likely to use non-iodized or inadequately iodized salt compared to households with medium or high standard of living index. While 47 percent of households with low standard of living used non-iodized salt, only 11 percent households with a high standard of living fall in this category. The number of households with a high standard of living using adequately iodized salt is more than twice of those with a low standard of living.

2.11 Iodization of Salt by Districts

Table 2.11 shows district level variation in the percent distribution of households by level of iodization of salt used in the households. Kaithal, Panchkula and Sonipat have the lowest proportion of households (12 percent) using non-iodized salt, whereas Bhiwani has the highest proportion of households (48 percent) using non-iodized salt. Percentage of households using inadequately iodized salt is the highest (31 percent) in Kaithal and Sirsa and the lowest in Ambala (8 percent). Around 55 percent of the households in the state used adequately iodized salt, the highest being in the district of Ambala (78 percent). In 12 out of 19 districts about half of the households were using adequately iodized salt (see Map-2).

Table 2.11 IDOIZATION OF SALT BY DISTRICT

Percent distribution of household heads by degree of idoization of salt by district, Haryana, 2002-04

District	Not iodised	7ppm	15+ppm	Other[1]
Ambala	13.7	7.5	78.2	0.7
Bhiwani	48.1	11.4	40.4	0.1
Faridabad	27.8	18.9	52.9	0.5
Fatehabad	27.3	23.1	49.1	0.5
Gurgaon	33.8	20.7	45.0	0.5
Hisar	41.7	10.4	47.8	0.2
Jhajjar	29.6	14.2	54.4	1.8
Jind	20.3	20.5	58.6	0.6
Kaithal	11.6	31.1	54.4	2.9
Karnal	25.5	12.1	59.9	2.5
Kurukshetra	20.7	9.5	68.7	1.1
Mahendragarh	41.7	21.2	36.7	0.4
Panchkula	12.2	22.4	64.2	1.2
Panipat	13.1	17.3	67.8	1.8
Rewari	30.0	20.0	49.4	0.6
Rohtak	22.7	8.6	67.9	0.9
Sirsa	19.6	30.8	49.2	0.5
Sonipat	12.3	20.5	66.5	0.7
Yamunanagar	19.9	19.0	60.0	1.1
Haryana	26.3	17.5	55.3	0.9

Note: Ppm: Parts per million. [1] Includes salt not at home, salt not tested, refused and missing cases

2.12 Availability of Facility and Services to the Rural Population

The DLHS-RCH collected information about surveyed village from knowledgeable persons such as, the '*Sarpanch*' or '*Pradhan*', (village head) or other village officials or other persons including 'teacher' in the villages on health and educational facilities and other services available in the village. One important aspect was on the distance of the village, if not available within the village, from various types of education facilities, including primary school, middle school, secondary school, higher secondary school, college, *Gurujee* scheme and '*Madarsa*'. Further information on the distance of the village, if not available within the village, from various types of health facility, including sub-centres, primary health centres (PHCs), community health centres/ Rural Hospitals (CHCs/RHs), Government dispensary, hospital, private clinic or hospitals and health facilities of Indian system of Medicine (ISM).

Table 2.12 gives the distance of surveyed villages from an education facility. The unit of analysis is usual residents of rural population. Most of the rural residents (98 percent) (the *de jure* rural population) in the state live in villages that have a primary school, 73 percent live in villages with middle school and half of the rural population live in villages with secondary schools and *Gurujee* scheme. Higher secondary schools are available for 25 percent of the rural population. Four percent of the rural population live in villages, which have *Madarassas*. Only 2 percent of the surveyed villages have a college. As regards the distribution of educational institutions within 5 kilometres distance from of the village, it can be seen that, 18 percent of the villages have middle school, 26 percent have secondary school, 28 percent have higher secondary school and 8 percent have a *'Madarassa'* within this distance. For 65 percent of the villages, the college is more than 10 kilometres away and *madarassa* are available at this distance for 11 percent of the villages.

Table 2.12 DISTANCE FROM THE NEAREST EDUCATION FACILITY

Percent distribution of rural household population by distance from the nearest education facility, Haryana, 2002-04

Education facility	Within village	Distance from the village < 5 km	5-9 km	10+ km	Don't know/ missing	Total percent
Primary School	98.1	1.7	0.2	0.0	0.0	100.0
Middle School	72.9	18.1	6.4	2.2	0.4	100.0
Secondary School	52.5	25.5	13.7	8.2	0.2	100.0
Higher Secondary School	24.5	28.1	24.3	22.5	0.5	100.0
College	2.2	9.7	22.4	65.2	0.5	100.0
Gurujee Scheme	1.6	8.0	2.9	13.0	74.5	100.0
Madarsa	4.3	8.4	4.6	11.3	71.3	100.0

Note: Table based on rural *de jure* population

Table 2.13 DISTANCE FROM THE NEARSET HEALTH FACILITY

Percent distribution of rural household population by distance from the nearest health facility, Haryana, 2002-04

Health facility	Within village	Distance from the village < 5 km	5-9 km	10+ km	Don't know/ missing	Total percent
		Rural household population				
Sub-centre	55.2	20.8	14.9	8.5	0.5	100.0
Primary health centre	23.5	19.0	31.9	24.7	0.9	100.0
Either sub-centre or PHC	60.9	20.7	12.0	6.2	0.2	100.0
Community health centre/ Referral hospital	2.8	11.4	31.4	53.1	1.3	100.0
Government dispensary	15.0	13.4	28.7	41.5	1.4	100.0
Government hospital	4.9	9.2	25.2	59.9	0.8	100.0
Private clinic	40.2	13.9	21.5	23.7	0.7	100.0
Private hospital	7.3	13.3	29.9	48.1	1.5	100.0
ISM health facility	8.8	10.2	26.6	51.2	3.2	100.0

Note: Table based on rural de jure population

Table 2.13 summarises the availability of health facilities within the surveyed villages and provides information on the distance between the villages and the nearest health facility. About 55 percent of the rural population live in villages with Sub-centres. Only 24 percent of the rural household population live in a village with a primary health centre, though the proportion of villages having facilities of either Sub-centre or primary health centre is 61 percent. The proportion of rural population with other health facilities are 3 percent for CHCs/RHs, 15 percent for Government dispensary, 5 percent for Government hospitals, 40 percent for private clinics, 7 percent for private hospitals and 9 percent for Indian System of Medicine.

**Table 2.14 AVAILABILITY OF SERVICES**<br>Percentage of rural residents living in villages that have selected services, Haryana, 2002-04	
Services	Percentage of rural residents
Anganwadi centre	99.7
Anganwadi worker	94.2
Private doctor	55.3
Visiting doctor	34.3
Homeopathic doctor	6.4
Village health guide	14.1
Trained birth attendant	50.2
Traditional healer	16.0
Dai	75.4
Note: Table based on rural de *jure* population	

The proportion of rural population located within a distance of 5 kilometres from health facilities are 21 percent for sub-centres, 19 percent for primary health centres, 11 percent for CHCs/RHs, 13 percent for a Government dispensary, 9 percent for Government hospitals, 14 percent for private clinic, 13 percent for private hospitals and 10 percent for ISM health facilities. Distance of particular health facilities is beyond 10 kilometres from surveyed villages in the case of Government hospitals (60 percent) and private hospitals, (48 percent).

Table 2.14 shows the proportion of rural residents in the state that live in the villages with various health services. All the rural residents live in villages that have an *anganwadi,* (a nursery school for children age 3-6 years) and at the same time 94 percent of rural households live in villages with *anganwadi* workers (*Anganwadi* workers provide integrated child development services) are available.

More than half of the rural residents live in villages that have a private doctor, 34 percent live in villages with a visiting doctor, 6 percent with a homoeopathy doctor, 14 percent with a village health guide, 50 percent with a trained birth attendant and 16 percent with a traditional healer. About three-fourth of the rural residents live in villages that have a *Dai* (*Dai* provides the services for the delivery).

2.13 Availability of Education Facility and Health Services by District

Table 2.15 shows the availability of education and health facilities for the rural population within the surveyed villages by districts in Haryana. All the rural population of Haryana have access to primary or middle schools except the districts of Gurgaon, Jhajjar, Jind, Karnal, Panchkula, and Yamunanagar. In the state of Haryana, 98 percent of the rural population live in villages having primary schools. Around 55 percent of the rural population in the state have sub-centres within the village, with the highest coverage of 88 percent in Hisar and the lowest of 27 percent of the population in Yamunanagar. Highest availability of PHCs within the village is found in Panipat (44 percent); while in Yamunanagar only 6 percent villages have PHCs.

Table 2.15 AVAILABILITY OF FACILITY AND SERVICES BY DISTRICT

Selected facility and services of rural household population within village by district, Haryana, 2002-04

Districts	Percentage of rural household population with						
	Primary or middle school	Sub-centre	PHCs	Any govern-ment health facility[1]	Doctor[2]	TBA[3]	Angan-wadi worker
Ambala	100.0	40.3	7.9	44.3	18.0	40.5	92.5
Bhiwani	100.0	54.8	42.7	57.9	88.3	63.7	98.3
Faridabad	100.0	65.2	22.5	65.2	85.4	37.9	94.6
Fatehabad	100.0	49.2	21.2	70.3	46.5	25.5	100.0
Gurgaon	98.6	47.1	19.7	57.4	92.8	54.8	92.3
Hisar	100.0	88.0	30.8	88.0	0.9	71.7	92.6
Jhajjar	91.5	53.4	14.6	64.6	65.3	43.4	96.7
Jind	96.1	42.4	27.7	50.2	76.0	32.8	93.0
Kaithal	100.0	70.9	13.1	76.3	90.1	58.4	96.5
Karnal	96.4	55.8	26.2	79.1	75.6	56.8	84.1
Kurukshetra	100.0	49.9	14.4	53.6	19.8	50.9	88.3
Mahendragarh	100.0	53.2	26.6	63.5	65.4	50.2	100.0
Panchkula	90.7	46.5	16.6	46.5	79.9	50.6	90.5
Panipat	100.0	72.4	43.6	81.5	97.4	84.8	95.4
Rewari	100.0	49.7	16.0	56.5	29.3	17.1	85.1
Rohtak	100.0	81.8	29.3	86.4	71.2	63.5	96.1
Sirsa	100.0	49.4	31.6	64.0	70.4	54.4	96.4
Sonipat	100.0	41.4	17.4	63.0	76.1	44.4	96.4
Yamunanagar	88.5	27.0	6.0	36.2	77.2	42.2	91.3
Haryana	98.4	54.9	23.5	64.0	66.4	50.2	93.8

Note: [1]Includes sub-center, primary health center, community health center or referral hospital, government hospital, and government dispensary within the village [2] Either private or visiting doctor [3]Trained birth attendant

Around 97 percent of the rural population are visited either by private or by visiting doctors in the surveyed villages of Panipat district, whereas less than one percent households can be classified in this category in Hisar district. Highest numbers of rural population (85 percent) are attended by trained birth assistants in Panipat, while only 17 percent of rural population, availed themselves of such a provision in Rewari. A visit by *anganwadi* workers to rural households is highest (100 percent) in Fatehabad and Mahendragarh and the lowest in Karnal (84 percent).

MAP-1
Percent Girl Marrying Below Legal Age at Marriage

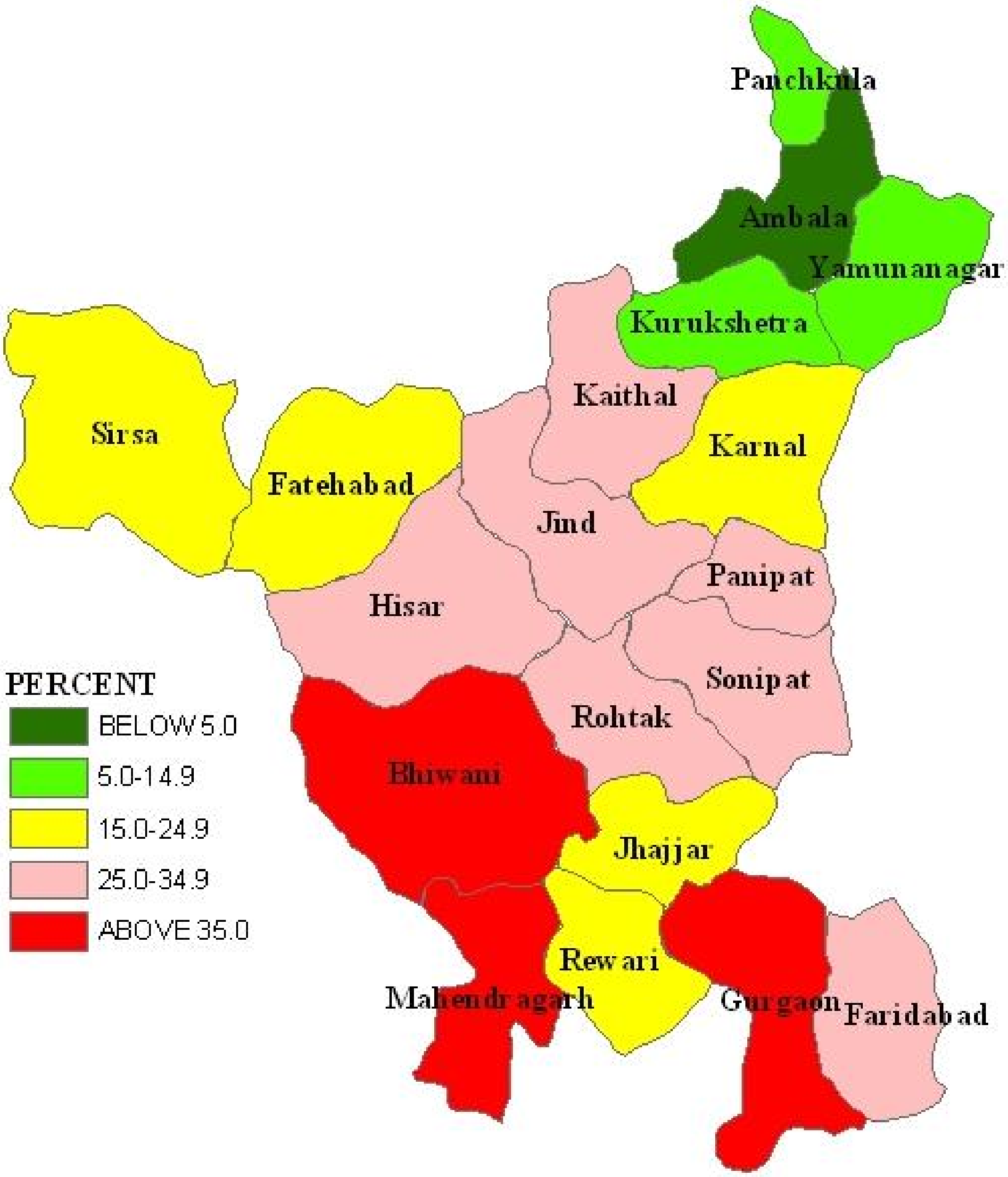

MAP-2
Percentage of Households Using Salt that Contains 15 ppm Level of Iodine

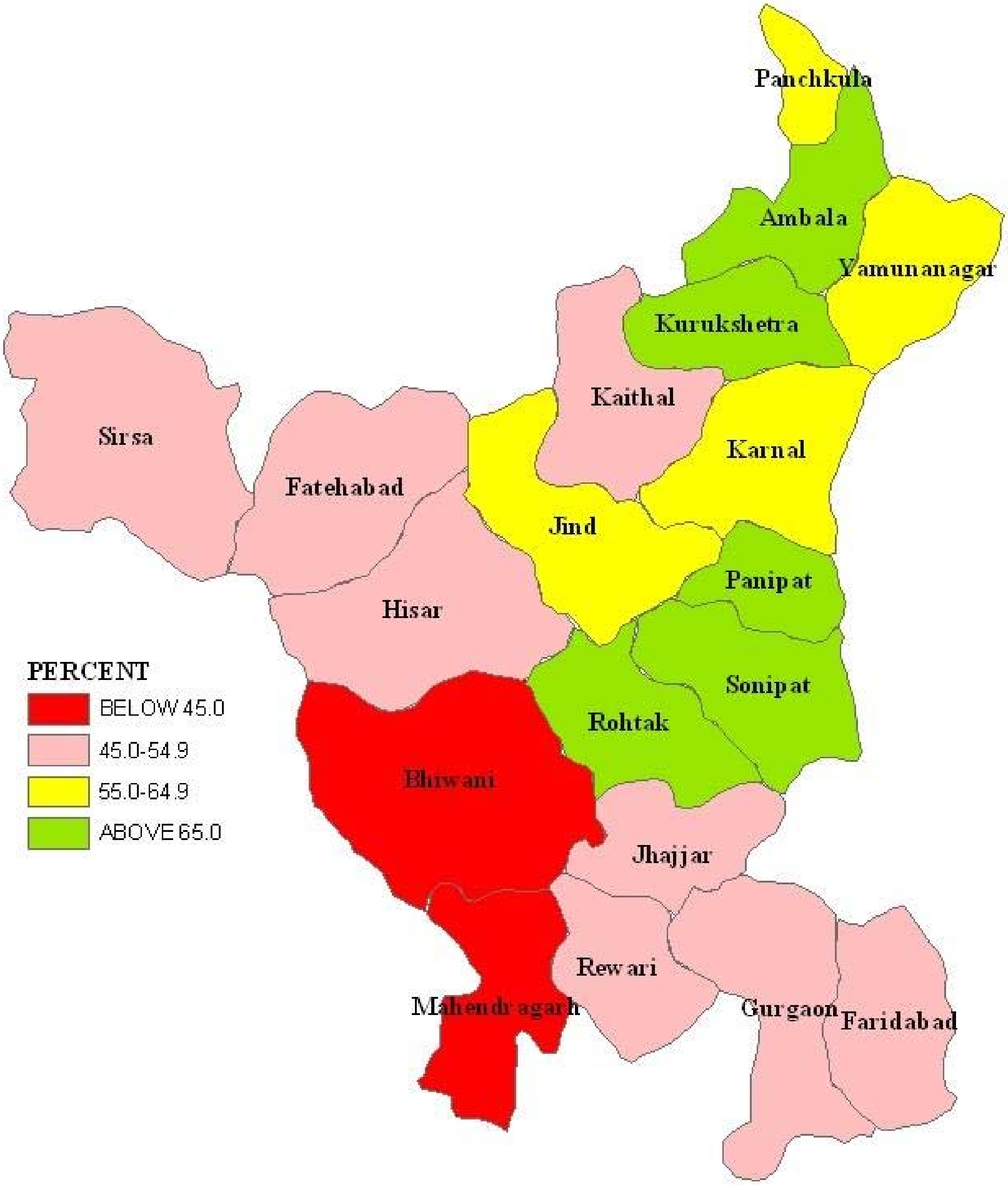

CHAPTER III

CHARACTERISTICS OF WOMEN, HUSBANDS AND FERTILITY

The Reproductive and Child Health (RCH) programme is targeted towards the underprivileged section of the population, particularly, women and children. The utilization of RCH services provided across the country depends to a large extent on the characteristics of women, their husbands and episodes of pregnancies, miscarriages, abortions, number of children born to them and survival status of children. Age of women, marital duration, educational attainment, social background and living standard are important factors, which influence reproductive and child health. With this in view, the DLHS-RCH data were collected on demographic characteristics, such as current age, age at consummation of marriage and number of pregnancies, live births and surviving children from eligible women respondents of selected representative households. Information regarding household background characteristics was collected using a separate household questionnaire that covered religion and caste of head of household, type of house, source of drinking water and possession of consumer durables. Fertility preference of women in terms of timing and desire for additional children in comparison to the number of living children provides information on the need for reproductive and child health services.

This chapter provides a comprehensive outline of distribution of currently married women by present age, age at consummation of marriage, duration of marriage, complete years of schooling, pregnancy episodes, children ever born and children surviving, along with social and economic characteristics of households the women represent.

3.1 Background Characteristics of Women

The percent distribution of currently married women in the reproductive age group 15-44 years by residence, religion and caste of head of household, economic standard of household and other demographic characteristics are shown in Table 3.1. A sample of 18,795 eligible women represents the state of Haryana in DLHS-RCH and more than two-third of these women are drawn from rural areas. About 62 percent of the currently married women are in the age range of 20-34 years and a similar age distribution is observed both for urban and rural areas. Age at consummation of marriage, particularly in rural areas is found to be low with as many as 53 percent of the women having cohabited before 18 years of age, while it is 32 percent in urban areas. Looking at the distribution of marital duration it is noted that about 38 percent of the women across the state are married for more than 15 years.

Among the sample 18,795 representative women in Haryana, Hindus constitute 90 percent. More Hindu women are found in urban areas (93 percent) than in rural areas (89 percent). The presence of women belonging to other religious groups is insignificant in proportional and absolute terms. Thirty one percent of the women belong to other backward class and 22 percent scheduled castes. Majority of the sample women (47 percent) belong to other caste other than scheduled caste/tribe and other backward class. In urban areas, there are more women belonging to other castes than in rural areas, while more women from scheduled caste and other backward classes are found in rural areas. There is a clear rural-urban differential in the educational attainment of women. For the state of Haryana, 45 percent of women are non-

literate and women of this category constitute 27 percent in urban areas while it is just 52 percent in rural areas.

Table 3.1 BACKGROUND CHARACTERISTICS OF ELIGIBLE WOMEN

Percent distribution of currently married women aged 15-44 by selected background characteristics, according to residence, Haryana, 2002-04

Background characteristic	Total	Residence	
		Rural	Urban
**Age group**			
15-19	8.6	9.9	5.2
20-24	22.1	23.7	18.3
25-29	21.2	20.7	22.3
30-34	19.0	18.6	20.0
35-39	15.8	14.6	18.7
40-44	13.4	12.5	15.4
**Age at consummation of marriage**			
Below 18 years	47.1	53.4	31.8
18 years & above	52.9	46.6	68.2
**Marital duration**			
0-4	23.4	23.7	22.6
5-9	19.7	19.9	19.2
10-14	19.3	19.0	20.1
15+	37.7	37.5	38.2
**Religion**			
Hindu	89.8	88.6	92.5
Muslim	5.0	6.1	2.1
Christian	0.1	0.1	0.2
Sikh	4.9	5.0	4.6
Buddhist	0.0	0.0	0.1
Jain	0.2	0.0	0.5
Zoroastrian	0.0	0.0	0.0
No religion	0.0	0.0	0.0
Other	0.0	0.0	0.0
**Caste/tribe**			
Scheduled caste	21.7	24.3	15.4
Scheduled tribe	0.6	0.6	0.4
Other backward class	31.1	32.8	26.8
Other #	46.6	42.2	57.3
Don't know	0.0	0.0	0.1
**Education (Years of schooling)**			
Non-literate	44.6	52.0	26.8
0-9@ years	30.8	32.3	27.3
10 years & above	24.5	15.7	45.9
**Husband's education (Years of schooling)**			
Non-literate	19.3	22.4	11.8
0-9@ years	33.6	35.8	28.3
10 years & above	46.9	41.7	59.8
Don't know	0.1	0.1	0.1
**Standard of living index**			
Low	16.5	22.1	3.0
Medium	43.8	52.0	23.8
High	39.7	25.9	73.2
Number of women	18,795	13,306	5,489

Note: Total includes 4 cases do not know information on caste, 9 cases missing information on women and 6 missing cases on husband education.
Higher caste Not belonging to a scheduled caste, scheduled tribe and an other backward class.
@ Literate persons with no year of schooling are included.

About 31 percent of women across the state have completed 0-9 years of schooling. Only a handful, 16 percent of rural women have completed 10 or more years of schooling compared to 46 percent for urban women. Men are more literate than their spouses. In Haryana, 19 percent of the husbands of eligible women are non-literate and the corresponding figures are 22 percent in rural areas and 12 percent in urban areas. The DLHS-RCH includes data on materials used for floor, walls and roofs of the housing structure along with status of possession of a list of durables and these are utilized to construct a composite index of household standard of living. Households are further classified as those with low, medium and high standard of living. Seventeen percent of women in the state live in low standard of living households and this is 22 percent in rural areas and 3 percent in urban areas. Less than half (44 percent) of the women across the state live in households categorised as medium standard of living. In rural areas, 52 percent of women belong to medium standard of living households and the corresponding figure is just 24 percent in urban areas.

3.2 Educational Level of Women

Table 3.2 provides details of educational level of eligible women in terms of classification by years of schooling, and selected background characteristics, such as, place of residence, religion, and caste and husbands' education. As regards distribution of non-literate women, it is observed that a lesser proportion of younger women below 30 years of age are non-literate compared to older women above 30 years. This age divide remains true even among literate women. A distinct pattern of educational attainment of women is that maximum of them attended schooling either for 1-5 years or 6-8 years and not many had 11 or more years of schooling. For the women in the age group 15-19 years, 18 percent of them had 1-5 years and 26 percent of them had 6-8 years of schooling, while only 8 percent had 11 or more years of schooling. Among the senior women in the age group 40-44 years, distribution by year of schooling varies with 11 percent, 9 percent, 10 percent and 8 percent of them having attended school for 1-5, 6-8, 9-10 and 11 or more years of schooling respectively.

There is a significant rural-urban differential in the level of education of women in Haryana. About 52 percent of rural eligible women are non-literate and 15 percent, 16 percent, 12 percent and 6 percent of the women have 1-5, 6-8, 9-10 and 11 or more years of schooling. The corresponding figures in urban areas are 27 percent non-literate and 11 percent, 15 percent, 21 percent and 27 percent respectively. More Muslim women (86 percent) are non-literate compared to Hindu women (43 percent), Sikh women (30 percent) and women belonging to other religious communities (17 percent). The proportion of Hindu women with 1-5 years of schooling is 14 percent and the same is 17 percent for Sikh women, 8 percent for Muslim women, and 10 percent for women from other religions. Among the literate Muslim women hardly 2 percent of them have 11 or more years of schooling, while 13 percent of literate Hindu women have attained this level of education.

The uneven level of educational attainment by caste can be noted from the recorded proportion of non-literate women among scheduled caste (62 percent), scheduled tribe (61 percent), other backward class (50 percent) and other caste (32 percent). The husband's education is an important characteristic, which has strong association with the education of eligible women. As many as 85 percent of women whose husbands are non-literate are also non-

literate, while only 14 percent of women whose husbands have 11 or more or years of schooling are non-literate. Thirty nine percent of literate women educated for 11 or more years of schooling have husbands who have the same level of education.

Table 3.2 LEVEL OF EDUCATION OF ELIGIBLE WOMEN

Percent distribution of currently married women aged 15-44 by years of schooling, according to selected background characteristics, Haryana, 2002-04

Background characteristic	Non-literate	Literate but no schooling	Years of schooling				Missing	Total percent	Number of women
			1-5 years	6-8 years	9-10 years	11 or more years			
**Age group**									
15-19	32.7	0.2	17.7	25.6	15.6	8.3	0.0	100.0	1,609
20-24	30.8	0.0	13.9	21.5	18.1	15.6	0.0	100.0	4,158
25-29	35.5	0.0	13.4	16.4	17.1	17.6	0.1	100.0	3,977
30-34	52.9	0.0	12.7	11.2	11.6	11.5	0.0	100.0	3,567
35-39	57.8	0.1	13.3	9.3	10.9	8.4	0.1	100.0	2,973
40-44	62.3	0.1	10.8	9.1	10.0	7.6	0.1	100.0	2,510
**Place of residence**									
Rural	52.0	0.1	14.6	15.5	11.5	6.3	0.1	100.0	13,306
Urban	26.8	0.0	10.5	14.5	20.9	27.2	0.0	100.0	5,489
**Religion**									
Hindu	43.2	0.0	13.5	15.7	14.6	12.9	0.1	100.0	16,871
Muslim	86.1	0.3	7.7	2.9	1.3	1.7	0.0	100.0	935
Sikh	29.9	0.0	17.3	20.2	19.6	13.0	0.0	100.0	922
Other	16.8	0.0	10.4	10.5	30.3	31.9	0.0	100.0	67
**Caste/tribe #**									
Scheduled caste	62.4	0.1	15.0	12.8	6.5	3.2	0.1	100.0	4,084
Scheduled tribe	61.3	0.0	9.8	10.7	9.0	9.2	0.0	100.0	104
Other backward class	50.3	0.0	14.3	16.0	12.2	7.0	0.1	100.0	5,839
Other	32.4	0.0	12.1	15.9	19.3	20.3	0.0	100.0	8,764
**Husband's education**									
Non-literate	84.9	0.1	9.1	4.3	1.2	0.4	0.0	100.0	3,632
1-5 years	65.1	0.1	19.0	11.0	3.5	1.0	0.3	100.0	1,865
6-8 years	47.5	0.1	19.3	21.6	9.2	2.3	0.1	100.0	3,614
9-10 years	33.4	0.0	14.6	20.1	22.8	9.1	0.0	100.0	5,098
11 or more years	14.2	0.0	8.5	15.2	23.6	38.5	0.0	100.0	4,552
Total	44.6	0.0	13.4	15.2	14.2	12.4	0.0	100.0	18,795

Note: # Total number may not add up to N due to don't know cases. Table includes 29 do not know cases on husband's education and 4 cases on caste were not shown separately. () Based on less than 50 unweighted cases. 5 cases not shown: Based on few cases.

3.3 Background Characteristics of Husbands of Eligible Women

In DLHS-RCH husbands of eligible women were also interviewed. The response rate for husbands is relatively low compared to that of eligible women. Selected background characteristics of husbands are shown in Table 3.3. Across the state of Haryana, husbands are mostly in the age group 25-34 years. Fewer husbands are below 25 years. In Haryana, 90 percent of the husbands are Hindus, 5 percent each of the husbands are Sikh and Muslim and presence of other religious groups is insignificant. Twenty two percent of husbands in the state belong to the scheduled caste and it is little more in rural areas (25 percent) than in urban areas (15 percent). Nearly 47 percent of the husbands belong to castes other than scheduled caste, scheduled tribe and other backward classes. In urban areas husbands from other castes constitute 58 percent,

while it is 42 percent rural areas. As regards educational characteristics of the husbands of surveyed eligible women, 36 percent of them have completed 0-9 years of schooling and the proportion of non-literate husband ranges from 12 percent in urban areas to 24 percent in rural areas, while the overall state figure is 20 percent.

Table 3.3 BACKGROUND CHARACTERISTICS OF MEN

Percent distribution of husband of eligible women by selected background characteristics, according to residence, Haryana, 2002-04

Background characteristic	Total	Residence	
		Rural	Urban
**Age group**			
< 25	11.8	13.4	7.8
25-34	38.1	38.9	36.2
35-44	35.1	33.1	40.0
45 +	15.0	14.5	16.1
**Religion**			
Hindu	89.8	88.8	92.2
Muslim	4.8	5.9	2.1
Christian	0.2	0.1	0.2
Sikh	5.1	5.1	4.9
Buddhist	0.0	0.0	0.1
Jain	0.1	0.0	0.4
Zoroastrian	0.0	0.0	0.0
No religion	0.0	0.0	0.0
Other	0.0	0.0	0.0
**Caste/tribe**			
Scheduled caste	22.2	25.0	15.2
Scheduled tribe	0.6	0.6	0.4
Other backward class	30.8	32.4	26.8
Other #	46.5	42.0	57.6
Don’t know	0.0	0.0	0.0
**Education (Years of schooling)**			
Non-literate	20.2	23.5	12.0
0-9@ years	36.0	38.4	30.2
10 years & above	43.7	38.0	57.9
**Standard of living index**			
Low	16.9	22.5	3.2
Medium	43.6	51.9	23.1
High	39.5	25.6	73.7
**Number of living children**			
0	10.9	11.1	10.4
1	14.1	13.6	15.3
2	28.9	27.5	32.3
3	24.6	24.2	25.6
4+	21.4	23.5	16.3
Number of Men	13,200	9,387	3,813

Note: # Higher caste Not belonging to a scheduled caste, scheduled tribe and an other backward class.
@ Literate persons with no year of schooling are included.

The proportion of husbands living in households classified as low, medium and high standard of living index are 17 percent, 44 percent and 40 percent respectively. In rural areas, 23 percent of the husbands live in low standard of living households compared to 3 percent in urban areas. This is complementary in the case of husbands living in high standard of living households, 74 percent in urban and 26 percent in rural. In terms of household standard of living

composition, those living in medium standard of living dominate in rural (52 percent) and in urban Haryana most husbands (74 percent) live in high standard of living households. Around 29 percent of husbands across the state reported to have two living children. More husbands in urban areas (15 percent) reported to have one living child, while more husbands in rural areas (24 percent) have four or more living children. Above 24 percent of the husbands of rural eligible women have more than three living children and it is 26 percent for husbands of urban eligible women.

3.4 Educational Level of Husbands of Eligible Women

Educational levels in categories of years of schooling classified by age, place of residence, religion and caste/tribe of husbands of eligible women are shown in Table 3.4. The distribution of non-literate husbands across age shows that it is marginally less for husbands below 25 years (12 percent) compared to the age groups 25-34 years (16 percent) and 24 percent for husbands in the age groups 35-44 years respectively. Among the literate husbands, irrespective of their age at the time of survey most of them have 1-8 years of schooling, 39 percent of those below 25 years and 27 percent of those above 45 years of age. As expected few of the younger husbands (23 percent) below 25 years have 11 or more years of schooling compared to 16 percent of those above 45 years. As in the case of eligible women, 48 percent of Muslim husbands are non-literate; while the corresponding non-literate husbands of Hindu and Sikh are 19 percent each. The proportions of husbands of Hindu, Muslim and Sikh who have 11 or more years of schooling constitute 23 percent, 7 percent and 19 percent respectively. Most of the literate Muslim husbands (16 percent) have completed 1-5 years of schooling and the corresponding numbers are 10 percent and 13 percent respectively for Hindu and Sikh husbands. Educational attainment of husbands of eligible women varies according to the caste/tribe they belong. There are more non-literate husbands belonging to scheduled caste (35 percent) followed by scheduled tribes husbands (25 percent). Among the scheduled caste and scheduled tribe husbands, 29 percent and 34 percent of them have 9 or more years of schooling. The literacy level of other backward classes is comparable with that of husbands from castes other than scheduled tribe, scheduled caste and other backward classes. Among the husbands belonging to other backward classes, 21 percent of them are non-literate and 45 percent of them have 9 or more years of schooling.

**Table 3.4 LEVEL OF EDUCATION OF MEN**									
Percent distribution of husbands of eligible women by years of schooling, according to selected background characteristics, Haryana, 2002-04									
Background characteristic	Non-literate	Literate but no schooling	Years of schooling				Missing	Total percent	Number of men
			1-5 years	6-8 years	9-10 years	11 or more years			
**Age group**									
< 25	11.7	0.2	11.6	27.1	26.2	23.2	0.1	100.0	1,556
25-34	15.9	0.0	10.2	19.7	26.3	27.8	0.0	100.0	5,034
35-44	24.0	0.1	11.0	19.2	27.8	18.0	0.0	100.0	4,632
45 +	28.9	0.1	11.7	15.0	28.3	16.0	0.0	100.0	1,978
**Place of residence**									
Rural	23.5	0.1	11.5	21.2	26.3	17.3	0.0	100.0	9,387
Urban	12.0	0.0	9.2	15.9	29.2	33.7	0.0	100.0	3,813
**Religion**									
Hindu	18.9	0.1	10.4	19.8	28.0	22.9	0.0	100.0	11,853
Muslim	47.9	0.4	15.9	17.1	11.5	7.3	0.0	100.0	637
Sikh	18.6	0.1	13.4	20.9	27.6	19.3	0.0	100.0	667
Other	(7.1)	(0.0)	(9.5)	(16.7)	(16.7)	(50.0)	(0.0)	(100.0)	41
**Caste/tribe #**									
Scheduled caste	35.4	0.0	14.5	20.9	18.2	10.9	0.0	100.0	2,925
Scheduled tribe	25.3	0.0	18.7	21.8	24.1	10.2	0.0	100.0	73
Other backward class	21.3	0.2	12.5	21.3	26.5	18.1	0.0	100.0	4,064
Other	12.1	0.0	7.9	18.0	31.8	30.1	0.0	100.0	6,137
Total	20.2	0.1	10.9	19.7	27.1	22.1	0.0	100.0	13,200

Note: # Total number may not add upto N due to don't know and missing cases. ( ) Based on less than 50 unweighted cases.

3.5 Children Ever Born and Surviving

In DLHS-RCH, currently married women in the age group of 15-44 years were asked about the children ever born alive and the number of children surviving. Table 3.5 shows mean children ever born and mean surviving children by selected background characteristics and sex of children. A look at the mean children ever born by age of the women reveals that older women had experience more average live births than younger women. On the average, women in the reproductive age group have given birth to more male children than female children and similar a sex differential is also noted when it comes to mean surviving children. Completed fertility, that is, mean children ever born to women in the age group 40-44 years is 4.1 for the state of Haryana and it comprises an average of 2.1 male children and 1.9 female children. Out of the 4.1 mean children ever born to women in the 40-44 year age group an average of 3.6 children survived. By sex of children, out of 2.1 mean numbers of males, 1.9 survived on the average and the corresponding mean number of females surviving was 1.7 out of 1.9.

Women with longer marital duration have higher mean children ever born. On the average, women who are married for 15 or more years have 3.9 children ever born and on the average 3.4 of them are surviving. There is a clear rural-urban divide in terms of mean children ever born with 2.8 children in rural areas and 2.5 children in urban areas. The mean children ever born to women who are Hindu, Muslim, Sikh and other religions are 2.6, 4.0, 2.3 and 2.5

respectively. The corresponding mean surviving children are 2.4, 3.5, 2.1 and 2.3 respectively for these religious groups. The average children ever born also vary by caste/tribe of the eligible women. For women belonging to scheduled caste and scheduled tribe, the mean children ever born are 3.0, other backward classes are 2.8 and other castes are 2.4. For all religious groups, the mean number of surviving children is slightly more than 2 shared almost by one surviving male and one surviving female children on the average.

Table 3.5 CHILDREN EVER BORN AND LIVING

Mean children ever born (CEB) and children surviving (CS) by selected background characteristics of currently married women aged 15-44 years, Haryana, 2002-04

Background characteristic	Mean children ever born			Mean children surviving			Number of women
	Total	Male	Female	Total	Male	Female	
**Age group (years)**							
15-19	0.5	0.3	0.2	0.5	0.3	0.2	1,609
20-24	1.5	0.8	0.7	1.4	0.7	0.7	4,158
25-29	2.6	1.4	1.2	2.4	1.3	1.1	3,977
30-34	3.3	1.8	1.5	3.0	1.6	1.3	3,568
35-39	3.7	2.0	1.7	3.3	1.8	1.5	2,973
40-44	4.1	2.1	1.9	3.6	1.9	1.7	2,510
**Marital duration**							
0-4	0.8	0.4	0.4	0.8	0.4	0.4	4,395
5-9	2.2	1.2	1.0	2.1	1.1	1.0	3,694
10-14	3.0	1.6	1.4	2.8	1.5	1.2	3,624
15+	3.9	2.1	1.8	3.4	1.9	1.6	7,082
**Residence**							
Rural	2.8	1.5	1.3	2.5	1.3	1.1	13,306
Urban	2.5	1.3	1.1	2.3	1.2	1.0	5,489
**Religion**							
Hindu	2.6	1.4	1.2	2.4	1.3	1.1	16,871
Muslim	4.0	2.1	1.9	3.5	1.9	1.6	935
Sikh	2.3	1.2	1.0	2.1	1.2	1.0	922
Other	2.5	1.5	1.0	2.3	1.4	0.9	67
**Caste/tribe #**							
Scheduled caste	3.0	1.6	1.4	2.6	1.4	1.2	4,084
Scheduled tribe	3.0	1.6	1.4	2.6	1.3	1.3	104
Other backward class	2.8	1.5	1.3	2.5	1.4	1.2	5,839
Other	2.4	1.3	1.1	2.2	1.2	1.0	8,764
**Education**							
Non-literate	3.4	1.8	1.6	3.0	1.6	1.4	8,388
0-9@ years	2.3	1.2	1.0	2.1	1.2	1.0	5,794
10 years & above	1.7	1.0	0.8	1.7	0.9	0.8	4,604
**Standard of living index**							
Low	3.4	1.8	1.6	3.0	1.6	1.4	3,099
Medium	2.7	1.4	1.3	2.5	1.3	1.1	8,229
High	2.3	1.3	1.0	2.1	1.2	1.0	7,467
All women	2.7	1.4	1.2	2.4	1.3	1.1	18,795

Note: # Total number may not add upto N due to don't know and missing cases. Table includes 9 women with missing information on education.
@ Literate women with no year of schooling are included.

The mean children ever born is higher for non-literate women (3.4) than women who have completed 0-9 years of schooling (2.3) and 10 or more years of schooling (1.7). The mean number of surviving children for women corresponding to these educational levels is 3.0, 2.1 and 1.7 respectively. Further the mean children ever born for women classified into low, medium and high standard of living by SLI are 3.4, 2.7 and 2.3 respectively. For the state of Haryana, the DLHS-RCH shows inverse association between mean children ever born and educational attainment of women and also the level of household economic comfort.

3.6 Completed Fertility by District

The level of completed fertility as measured by mean children, ever born to women of 40-44 years by districts in Haryana together with mean number of surviving children are shown in Table 3.6. On the average, women on the verge of completing reproductive period have given birth to 4.1 children in their reproductive life of which 3.6 children are surviving on the average. Completed fertility in Haryana varies from the low of 3.5 mean children ever born for Panchkula and Rewari to the highest of 6.2 children in Gurgaon district. Mean children ever born in all the districts of Haryana are 3 or more than 3 children. It is also true that in all the districts mean number of male children is more than the mean of female children born to women in the 40-44 year age group. Gurgaon (5.2) recorded highest mean number of surviving children. Looking at the absolute difference between mean children ever born and mean number of surviving children, it seems that infant and child mortality is quite high and varies among districts in Haryana.

Table 3.6 COMPLETED FERTILITY BY DISTRICT

Mean children ever born (CEB) and children surviving (CS) to currently married women aged 40-44 by district, Haryana, 2002-04

District	Mean children ever born			Mean children surviving		
	Total	Male	Female	Total	Male	Female
Ambala	3.8	1.9	1.9	3.4	1.7	1.7
Bhiwani	4.0	2.2	1.8	3.5	1.9	1.6
Faridabad	4.2	2.1	2.1	3.8	1.9	1.9
Fatehabad	3.7	2.0	1.6	3.3	1.8	1.5
Gurgaon	6.2	3.3	2.8	5.2	2.8	2.4
Hisar	3.9	2.1	1.7	3.3	1.8	1.5
Jhajjar	4.0	2.1	1.9	3.6	1.9	1.7
Jind	3.8	2.0	1.8	3.4	1.8	1.6
Kaithal	3.9	2.2	1.7	3.3	1.9	1.4
Karnal	4.3	2.2	2.0	3.7	1.9	1.8
Kurukshetra	3.7	2.0	1.7	3.2	1.8	1.4
Mahendragarh	4.1	2.1	2.0	3.6	1.9	1.7
Panchkulla	3.5	2.0	1.5	3.2	1.8	1.4
Panipat	4.6	2.3	2.3	4.0	2.1	1.9
Rewari	3.5	1.7	1.8	3.1	1.6	1.6
Rohtak	3.7	1.9	1.8	3.2	1.7	1.5
Sirsa	3.7	1.9	1.8	3.4	1.8	1.6
Sonipat	4.1	2.3	1.8	3.8	2.1	1.7
Yamunanagar	4.0	2.1	1.9	3.6	1.9	1.7
Haryana	4.1	2.1	1.9	3.6	1.9	1.7

3.7 Birth Order

Birth order distributions by selected background characteristics of women are provided in Table 3.7 and Figure 3.1. This distribution can be used as a measure of fertility in the absence of formal measures of fertility, such as, crude birth rate and total fertility rate.

Table 3.7 BIRTH ORDER

Percent distribution of births during three years preceding the survey by birth order by selected background characteristics, Haryana, 2002-04

Background characteristic	Birth order				Total percent	Number of births
	1	2	3	4+		
**Age of women**						
15-19	78.4	19.3	2.3	0.0	100.0	724
20-24	43.8	35.5	14.9	5.8	100.0	3,463
25-29	14.4	29.8	26.7	29.1	100.0	2,136
30-34	4.8	14.6	18.8	61.8	100.0	773
35-39	0.6	7.9	15.9	75.6	100.0	250
40-44	3.1	2.5	7.6	86.8	100.0	84
**Place of residence**						
Rural	31.8	27.7	17.2	23.3	100.0	5,497
Urban	35.4	32.1	18.0	14.6	100.0	1,933
Education (Years of schooling)						
Non-literate	20.8	21.2	18.8	39.2	100.0	2,979
0-9@ years	35.7	32.9	18.8	12.6	100.0	2,590
10 years & above	47.8	35.4	13.2	3.6	100.0	1,859
**Religion**						
Hindu	33.9	30.1	17.8	18.2	100.0	6,393
Muslim	16.2	14.4	15.3	54.0	100.0	692
Sikh	43.6	36.3	14.0	6.1	100.0	316
Other	(37.9)	(17.2)	(24.1)	(20.7)	(100.0)	29
**Caste/tribe #**						
Scheduled caste	28.7	26.6	18.8	25.9	100.0	1,888
Scheduled tribe	(30.4)	(34.8)	(15.2)	(19.6)	(100.0)	49
Other backward class	30.2	25.9	17.4	26.5	100.0	2,571
Other	37.6	32.8	16.6	13.0	100.0	2,921
**Standard of living index**						
Low	20.1	18.7	18.3	43.0	100.0	1,545
Medium	32.1	29.8	18.4	19.6	100.0	3,374
High	41.3	33.7	15.6	9.4	100.0	2,511
Total	32.7	28.8	17.4	21.0	100.0	7,430

Note: Total includes 2 births with missing information on mother's education.
\# Total number of births may not add upto N due to don't know and missing cases.
( ) Based on less than 50 un weighed cases.

For the state of Haryana, 33 percent of the births born in the three years period preceding the survey were of first order, 29 percent of second order and the remaining 38 percent were of order 3 and higher order births. By current age of eligible women, more than 50 percent of births to women in the age groups 30-34 years, 35-39 years and 40-44 years are 4 and higher order births. For women of 15-19 years, 78 percent births are of first order and 19 percent births are of second order. In the case of eligible women in urban areas 33 percent of the births are of 3 and higher whereas this order births constitute 41 percent for rural women indicating that higher order births are more concentrated in rural areas. Of the total births born to non-literate women,

58 percent are 3 and higher order births, followed by 31 percent for women with 0-9 years of schooling and 17 percent for women who had 10 or more years of schooling. In short, births born to non-literate women are of higher order whereas much lower order births occurred to women who completed 10 or more years of schooling. Looking at the religion differential in birth order distribution, it is observed that 69 percent of births born to Muslim women are 3 and higher order births. For Hindu and Sikh women, the 3 and higher order births constitute 36 percent and 20 percent respectively. The occurrence of births of order 3 and above is more among scheduled caste (45 percent) than among scheduled tribe (35 percent), other backward classes (44 percent) and other castes (30 percent) women. Incidence of births of order 3 and above for women classified by household standard of living index are 25 percent for high, 38 percent for medium and 61 percent for low living standard households women.

Figure 3.1
Birth Order3 & Above by Selected Background Characteristics

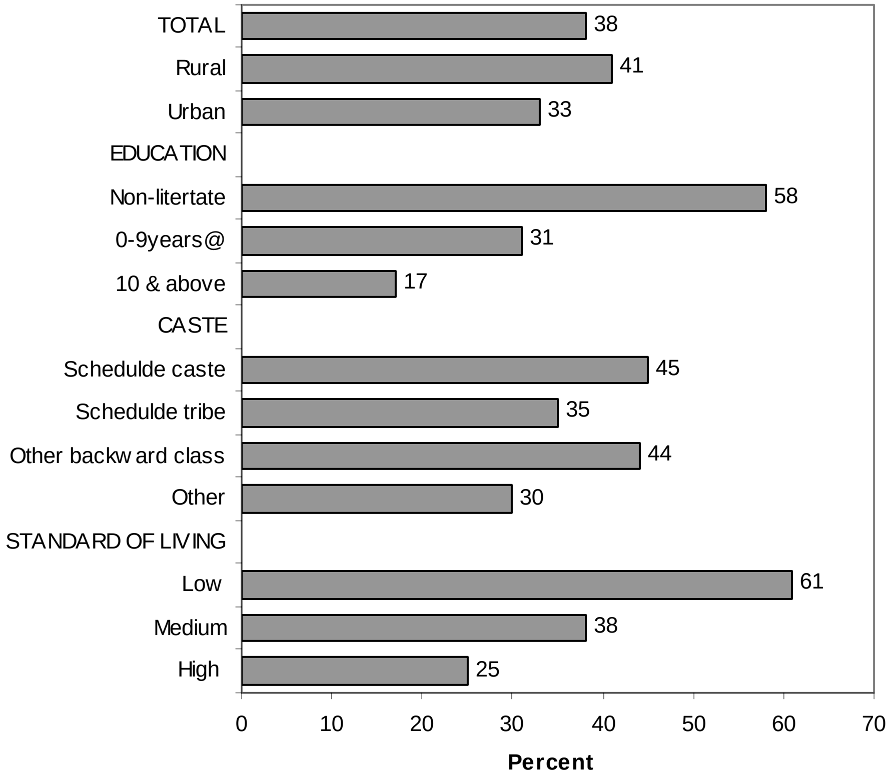

@ Literate women with no years of schooling are also included

Haryana, DLHS-RCH, 2002-04

3.8 Birth Order by District

Table 3.8 and Figure 3.2 shows the births order distribution by districts in Haryana. The proportion of births of order 3 and above ranges from the lowest of 24 percent in Panchkula to the highest of 56 percent in Gurgaon. The districts, which have lower proportion of births (less

than 30 percent) of order 3 and above, are Rohtak and Rewari (29 percent each), Kurukshetra (28 percent), Jhajjar (26 percent), Ambala (25percent) and Panchkula (24 percent) (The remaining districts fall between 35 percent to 56 percent.

Figure 3.2
Birth Order 3 & above by District

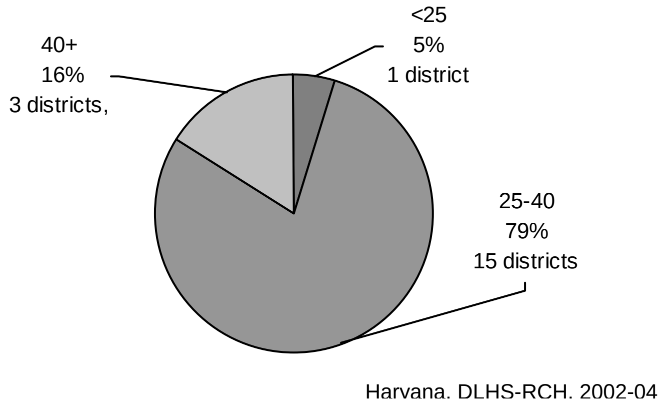

Table 3.8 BIRTH ORDER BY DISTRICT				
Percent distribution of births during three years preceding the survey by birth order, according to district, Haryana, 2002-04				
District	Birth order			
	1	2	3	4+
Ambala	43.2	32.2	16.6	7.9
Bhiwani	31.8	31.1	17.6	19.6
Faridabad	26.0	25.8	17.5	30.6
Fatehabad	33.1	29.3	17.7	20.0
Gurgaon	24.4	19.5	14.5	41.7
Hisar	38.4	26.8	20.2	14.5
Jhajjar	41.8	32.5	15.0	10.7
Jind	28.2	26.4	22.7	22.7
Kaithal	30.6	30.4	21.5	17.5
Karnal	32.8	31.1	17.2	18.8
Kurukshetra	43.0	29.5	13.6	14.0
Mahendragarh	34.0	31.1	18.6	16.4
Panchkula	39.1	36.7	15.9	8.3
Panipat	34.0	28.2	19.3	18.4
Rewari	35.2	36.1	16.2	12.6
Rohtak	35.7	35.5	16.6	12.2
Sirsa	31.7	32.1	15.9	20.3
Sonipat	36.4	28.1	19.7	15.7
Yamunanagar	32.2	28.0	17.7	22.0
Haryana	32.7	28.8	17.4	21.0

3.9 Fertility Preference

The distribution of currently married women desiring additional children and preferred sex of additional children by number of living children of the women is shown vividly in Table 3.9 and Figure 3.3. Out of the 1,351 women with no living child, 30 percent are currently pregnant and 4 percent are using spacing methods, while 60 percent want to have children within two years, 2 percent want to have children after two years, 1 percent is undecided about the timing of birth and less than one percent desired not to have any children. Among the currently married women, the desire for additional children dwindles down with increasing number of living children. As many as 30 percent of the women having one living child are using spacing methods, 28 percent of them want additional children within two years, 5 percent after two years, 2 percent are undecided about the timing of the next child, 4 percent of them want no more additional children and 2 percent are sterilized. Use of permanent as well as temporary means of contraception tends to be accelerated with number of living children. In the state of Haryana, out of the 18,795 surveyed representative women, 14 percent desired to have additional children within two years, 1 percent after two years, 10 percent want no more children, 10 percent are currently pregnant and 60 percent are using either terminal or temporary contraceptive methods. A total of 3,627 women want additional children irrespective of the number of living children. Out of 1351, women who have no living children and desire for additional children, 20 percent want a boy as the first child, 2 percent desired for girl, for 57 percent, the sex of the child is immaterial and 21 percent leave it to God. With increasing number of living children, male is the dominating preferred sex of the next child though a sizeable proportion of women desiring additional children expressed that the sex of the child was immaterial.

Figure 3.3
Fertility Preference

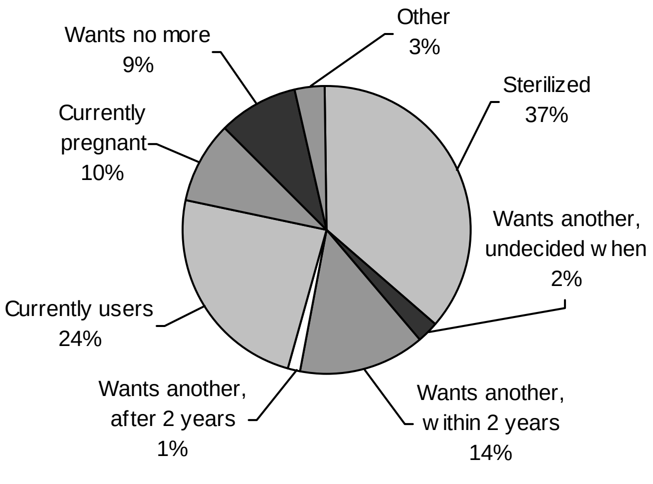

Haryana, DLHS-RCH, 2002-04

Table 3.9 FERTILITY PREFERENCE

Percent distribution of currently married women by desire for children, according to number of living children, Haryana, 2002-04

Desire for children	Number of living children 0	1	2	3	4+	Total
**Desire for additional child**						
Wants another soon[1]	59.6	27.7	7.5	3.7	3.1	14.1
Wants another later[2]	1.4	5.0	1.1	0.6	0.3	1.4
Want another, undecided when	2.4	7.9	1.7	1.1	1.1	2.3
Undecided	0.6	1.8	1.2	0.7	0.4	0.9
Up to God	0.6	0.5	0.4	0.2	1.2	0.6
Want no more	0.4	4.0	11.1	8.6	14.3	9.2
Sterilized	0.2	1.9	34.4	55.4	55.7	36.5
Currently users[3]	3.9	30.2	35.7	24.1	17.3	23.8
Currently pregnant	29.9	20.3	5.6	3.9	3.8	9.5
Declared infecund	0.8	0.6	1.2	1.7	2.7	1.6
Missing	0.2	0.2	0.1	0.0	0.1	0.1
Total percent	100.0	100.0	100.0	100.0	100.0	100.0
Number of women	2,090	2,643	4,776	4,214	5,072	18,795
Preferred sex of additional children						
Boy	20.3	39.3	55.8	66.9	60.8	38.6
Girl	1.8	10.6	6.6	6.3	1.1	5.6
Doesn’t matter	56.7	34.3	21.0	17.8	16.7	37.9
Upto God	21.0	15.8	16.5	9.0	21.5	17.8
Missing	0.2	0.0	0.1	0.0	0.0	0.1
Total percent	100.0	100.0	100.0	100.0	100.0	100.0
Number of women	1,351	1,133	570	261	309	3,625

Note: [1] Wants next births within 2 years. [2] Wants to delay next birth for 2 or more years. [3] Other than sterilization.

3.10 Pregnancy Outcomes

Table 3.10 shows distribution of pregnancy outcomes including live birth, stillbirth, induced abortion and spontaneous abortion by districts in Haryana. For the state as a whole, 90 percent of pregnancy ends in live births, 2 percent in induced abortions, 6 percent in spontaneous abortion and 1 percent in stillbirth. More pregnancies in rural areas end in live births (91 percent) than in urban areas (88 percent), while the incidence of induced abortion is more in urban areas (5 percent) than in rural areas (2 percent). The proportion of pregnancies ending in live births ranges from 84 percent in Karnal and to 96 percent in Kaithal. The incidence of stillbirth is highest in Karnal (3 percent) followed by Ambala and Bhiwani (2 percent). Induced abortion is highest in the district of Panchkula (6 percent). Spontaneous abortion is 3 percent each in Faridabad and Kaithal and highest in Hisar (9 percent).

Table 3.10 OUTCOMES OF PREGNANCY

Percent distribution of all pregnancies of currently married women aged 15-44 years by their outcomes three year preceding the survey currently married women, according to districts, Haryana, 2002-04

Districts	Live birth	Stillbirth	Induced abortion	Spontaneous abortion	Missing	Total percent
**State-Rural**	90.5	1.5	1.7	6.2	0.0	100.0
**State-Urban**	88.1	1.1	5.1	5.7	0.0	100.0
**State-Total**	89.9	1.4	2.6	6.1	0.0	100.0
Ambala	88.7	2.1	3.0	6.3	0.0	100.0
Bhiwani	89.2	2.0	1.6	7.2	0.0	100.0
Faridabad	93.4	1.0	2.8	2.8	0.0	100.0
Fatehabad	90.1	1.4	2.7	5.8	0.0	100.0
Gurgaon	89.5	1.2	2.2	7.2	0.0	100.0
Hisar	85.7	1.7	3.2	9.3	0.0	100.0
Jhajjar	92.0	1.3	2.2	4.5	0.0	100.0
Jind	87.5	1.3	2.0	9.2	0.0	100.0
Kaithal	96.0	0.9	0.4	2.8	0.0	100.0
Karnal	83.8	3.2	4.4	8.5	0.2	100.0
Kurukshetra	89.3	0.3	2.6	7.9	0.0	100.0
Mahendragarh	91.6	0.9	2.9	4.5	0.0	100.0
Panchkulla	88.6	1.1	6.2	4.2	0.0	100.0
Panipat	90.8	0.8	2.6	5.8	0.0	100.0
Rewari	89.7	1.7	2.2	6.4	0.0	100.0
Rohtak	90.2	1.2	2.6	6.0	0.0	100.0
Sirsa	94.1	1.0	0.9	3.7	0.3	100.0
Sonipat	92.4	0.2	1.6	5.8	0.0	100.0
Yamunanagar	87.8	1.7	4.5	6.0	0.0	100.0

CHAPTER V

CHILD CARE AND IMMUNIZATION

Child health services under the Reproductive and Child Health (RCH) programme include health education to mothers on breast-feeding and services for immunization, Vitamin A supplements and Iron prophylaxis, treatment of diarrhoea and Acute Respiratory Infections (ARIs). The District Level Household Survey (DLHS) covered all the currently married women whose last surviving child was born during the three years preceding the survey, and information on those breastfeeding currently and duration of breastfeeding. They were also asked about their awareness of diarrhoea management and danger signs of pneumonia and practices followed in case of episodes of diarrhoea and ARI among the children. Data on immunization, administering Vitamin A supplements and Iron prophylaxis was collected for the last two living children born after January 1, 1999/2001. This chapter presents an analysis of the data collected on the above aspects.

5.1 Breastfeeding

Educating mothers on correct breastfeeding practices and child nutrition is one of the components of the RCH programme. Infant feeding practices have significant effects on the health of both mothers and children. Mothers are affected through the influences of breastfeeding on the period of postpartum infertility, and hence on fertility levels and the length of birth intervals. These effects vary according to the duration and intensity of breastfeeding. Proper infant feeding, starting from the time of birth, is important for the physical and mental development of the child. Breastfeeding improves the nutritional status of young children and reduces morbidity and mortality. Breast milk not only provides important nutrients, but also protects the child against infection. The timing and type of supplementary foods introduced in an infant's diet have significant effects on the child's nutritional status.

As recommended by the World Health Organization (WHO), breastfeeding should be initiated immediately after birth and should be continued upto a minimum of six months. The WHO also suggests that the yellowish milk, known as colostrums, should be given to the baby because it provides protection against certain infections. Afterwards, it has to be supplemented with other semi-solid and solid foods at the proper time intervals.

Table 5.1 shows the breastfeeding practices among children born during the three years preceding the survey in Haryana. Although, the practice of breastfeeding is common in Haryana, the initiation of breastfeeding within two hours of the birth of the child is not always followed. Only seventeen percent of the children were breastfed within two hours of birth and 36 percent were breastfed within one day of birth (including those who were breastfed within two hours of birth), while 63 percent of children were breastfed after one day of birth. As shown in Figure 5.1, about 19 percent of the children were breastfed within one day of birth but after two hours of birth, 44 percent were breastfed after the first day of birth but before 3 days, and 19 percent children were put to the breast after three days. One percent of the children were never breastfed. Less than two-third of the women (62 percent) who gave birth to children during the three years preceding the survey squeezed the first milk from the breast before they began breastfeeding.

Not more than 23 percent of children in any socio-economic groups, shown in Table 5.1, were breastfed within two hours of birth. Twenty six percent of children from scheduled tribe were breastfed within two hours of birth and 30 percent of children from scheduled castes were breastfed within one day of birth. Women who reside in urban areas, women who have had high school education and above and women who live in households with a high standard of living are more likely to start breastfeeding their children early. A large proportion of children from rural areas (66 percent), Muslim children (81 percent), children from scheduled castes (69 percent), children of uneducated mothers (70 percent) and children from households with a low standard of living (75 percent) were put to the breast after one day of birth.

Table 5.1 INITIATION OF BREASTFEEDING

Percentage of children born during the three years preceding the survey who started breastfeeding within two hours of births, within one day of birth, and after one day of birth and percentage whose mother squeezed the first milk from her breast before breastfeeding by selected background characteristics, Haryana, 2002-04

Background characteristic	Percentage started breastfeeding			Percentage whose mother squeezed first milk from breast	Number of Children
	Within two hours of birth	Within one day of birth[1]	After one day of birth		
**Residence**					
Rural	16.0	33.1	65.5	62.3	4,409
Urban	20.8	43.6	54.5	62.8	1,633
**Mother's education**					
Non-literate	13.5	28.2	70.1	62.9	2,379
0-9@ years	16.7	36.3	62.4	63.7	2,078
10 and above	23.8	47.1	51.4	60.0	1,584
**Religion**					
Hindu	18.3	38.0	60.6	62.2	5,214
Muslim	6.8	18.4	80.7	63.4	544
Sikh	19.2	32.5	63.2	67.0	262
**Caste/tribe#**					
Scheduled caste	13.8	29.5	69.3	65.4	1,496
Scheduled tribe	26.4	39.2	58.3	47.2	38
Other backward class	15.6	34.4	64.0	63.8	2,087
Other	20.9	41.2	57.2	59.7	2,420
**Standard of living index**					
Low	11.6	23.6	75.2	58.8	1,250
Medium	15.9	34.3	64.4	63.4	2,669
High	22.5	45.3	52.8	63.3	2,123
Total	17.3	36.0	62.5	62.4	6,041

Note: Table based on youngest living child born during the three years preceding the survey
Table includes 22 children on other religion category who were not shown separately. [1] Includes children who started breastfeeding within two hours of births. @ Literate mother with no years of schooling are included.

The custom of squeezing the first milk from the breast before breastfeeding is widely practised in every group, but it is slightly higher among the mothers of scheduled caste children and children with Sikh religion. Children who live in households with a low standard of living are less likely than children in other households to have mothers who squeezed the first milk from the breast before breastfeeding. There is no Rural-Urban differential of the custom of squeezing the first milk from the breast before breastfeeding. Mothers of children born in the three years preceding the survey were asked whether the child had been fed breast milk exclusively and if so, what the duration was. Here it needs to be mentioned that, exclusive

breastfeeding includes breastfeeding the child without giving it anything including water. Results are shown in Table 5.2.

Figure 5.1
Initiation of Breastfeeding

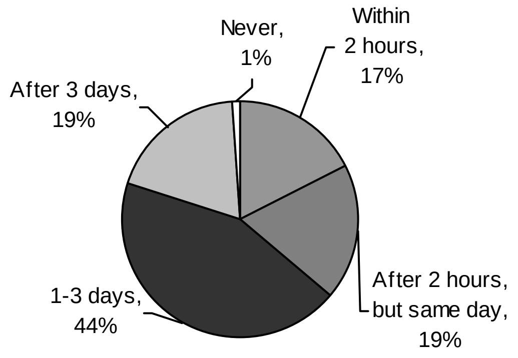

Haryana, DLHS-RCH, 2002-04

Table 5.2 EXCLUSIVE BREASTFEEDING BY CHILD'S AGE

Percentage of children under age3 years by exclusive breastfeeding, according to child's age in month, Haryana, 2002-04

Age in months	Status of exclusive breastfeeding			Number of children
	Exclusive breastfeeding	At least 4 months	At least 6 months	
<2	75.0	*	*	303
2-3	59.0	*	*	308
4-5	43.3	66.8	*	373
6-7	19.7	67.6	35.3	431
8-9	4.6	63.0	34.6	510
10-11	1.9	63.9	28.2	422
12-13	2.4	70.7	33.7	463
14-15	1.6	65.8	37.5	299
16-17	0.6	66.8	35.8	296
18-19	1.2	60.8	28.4	328
20-21	0.6	65.9	33.3	342
22-23	0.9	62.4	29.8	317
24-25	1.0	69.1	32.2	350
26-27	1.2	66.9	32.2	233
28-29	1.1	66.2	35.2	257
30-31	0.7	63.2	28.3	237
32-33	0.6	65.6	36.9	263
34-35	0.0	64.7	34.2	308
< 4 months	67.0	*	*	611
4-6 months	38.1	66.3	*	564
7-9 Months	7.4	65.0	34.0	751

Note: Table based on youngest living child born during the three years preceding the survey. *Percentage not shown: Based on very few cases.

In Haryana, only 67 percent of children under four months of age are exclusively breastfed. The percentage of infants exclusively breastfed drops steadily from 75 percent for children under 2 months of age to 43 percent for children who are 4-5 months old. About 38 percent of children in the age group 4-6 months were exclusively breastfed up to 4 months and 7 percent of children in the age group 7-9 months are exclusively breastfed upto 6 months.

5.1.1 Breastfeeding by Districts

Table 5.3 shows that in all the districts of Haryana, except Faridabad, Jhajjar, Panchkula and Rewari, not more than 25 percent of the children were put to the breast within two hours of birth. Only 8 percent of the children were breastfed within two hours of birth in Fatehabad and Yamunanagar districts. Less than half of the children were put to the breast after one day of birth in Faridabad, Jhajjar and Rewari districts. In 10 of the 19 districts, the mothers of more than 60 percent children squeezed the first milk before breastfeeding.

Table 5.3 BREASTFEEDING BY DISTRICT

Percentage of children under age 3 who started breastfeeding within two hours of births, within one day of birth and after one day of birth, percentage whose mother squeezed the first milk from her breast before breastfeeding and percentage of children who exclusively breastfeed by District, Haryana, 2002-04

District	Percentage started breastfeeding			Percentage whose mother squeezed first milk from breast	Exclusive breastfeeding[2]
	Within two hours of birth	Within one day of birth[1]	After one day of birth		
Ambala	9.1	20.8	76.4	82.7	21.6
Bhiwani	9.7	27.6	72.4	48.4	57.1
Faridabad	30.5	60.9	37.4	65.6	41.6
Fatehabad	8.2	24.0	75.7	68.2	1.7
Gurgaon	11.9	28.8	70.2	52.6	41.6
Hisar	10.0	20.7	76.3	79.3	14.8
Jhajjar	26.8	50.9	48.7	49.8	12.2
Jind	12.0	29.7	68.6	75.7	34.7
Kaithal	13.3	22.3	76.4	54.0	54.0
Karnal	19.8	39.5	58.4	69.5	13.8
Kurukshetra	11.8	28.7	68.6	86.2	10.7
Mahendragarh	22.3	45.4	52.7	60.5	64.1
Panchkula	32.7	49.1	50.9	74.9	35.5
Panipat	16.2	23.5	74.9	56.4	39.3
Rewari	27.6	57.5	40.4	43.0	7.2
Rohtak	24.0	45.6	54.4	45.1	9.4
Sirsa	21.3	34.1	63.4	55.1	74.4
Sonipat	17.9	42.6	57.2	56.1	25.3
Yamunanagar	7.6	23.1	74.0	79.9	32.0
Haryana	17.3	36.0	62.5	62.4	33.0

Note: Table based on youngest living child born during the three years preceding the survey.
[1]Includes children who started breastfeeding within two hours of births. [2] Based on youngest children age 6 months and older at the time of survey and breastfeed exclusively 6 months or more as mother reported.

There is a great deal of variation in the extent of exclusive breastfeeding for six months. It is highest in Sirsa (74 percent) and lowest in Fatehabad (2 percent).

5.2 Immunization of Children

The immunization of children against six serious but preventable diseases namely, tuberculosis, diphtheria, pertusis, poliomyelitis and measles is the main component of the child survival programme. As part of the National Health Policy, the National Immunization Programme is being implemented on a priority basis. The Government of India initiated the Expanded Programme on Immunization (EPI) in 1978 with the objective of reducing morbidity, mortality and disabilities among children from six diseases.

The Universal Immunization Programme (UIP) was introduced in 1985-86 with the objective of covering at least 85 percent of all infants against the six vaccine preventable diseases by 1990. This scheme has been introduced in every district of the country. The standard immunization schedule developed for the child immunization programme specifies the age at which each vaccine should be administrated and the number of doses to be given. Routine vaccinations received by infants and children are usually recorded on a vaccination card that is issued for the child.

In the first phase of Round II, all the women with last and last but one living child born after January 1, 1999 were asked whether the child/children had received the vaccination against polio, tuberculosis (BCG), diphtheria, whooping cough (pertusis), tetanus (DPT) and measles, and for the second phase, the reference period was from January 1, 2001. For Polio and DPT, further information on polio at birth and number of doses was asked. Children who received BCG, three doses of DPT and polio (excluding polio 0) and measles are considered to be fully vaccinated. Information on the source of immunization for last dose and in case where immunization was not given, the reason for not giving immunization was also compiled.

Table 5.4, Figures 5.2 and 5.3 present vaccination coverage rates for children in the age group 12-35 months. Only 59 percent of the children are fully vaccinated and around 12 percent have not received any routine vaccination. Coverage of each vaccination, except Polio 0, is much higher than the percentage fully vaccinated. BCG, DPT and Polio vaccine has each been given to more than three-fourths of children (Figure 5.3). Only 73 percent of the children have received three doses of DPT and 73 percent of the children received 3 drops of Polio and only 65 percent of the children have been vaccinated against measles. Moreover, not all children who begin the DPT and polio vaccination series go on to complete them. The differences between the percentage of children receiving the first and third doses is 11-percentage point for DPT and polio.

There has been deterioration in full vaccination coverage in Haryana since the time of Round I in 1998-99. These data indicate that despite the progress that has been made in immunization coverage for children in Haryana, coverage levels are still low and a large proportion of children who received some early vaccinations dropped out of the programme before receiving all of the recommended vaccinations.

Table 5.4 VACCINATION OF CHILDREN

Percentage of children age 12-35 months who received vaccination according to some selected background characteristics, Haryana, 2002-04

Background characteristic	Polio 0	BCG	DPT			Polio			Measles	Full[1] vaccination	No vaccination	Number of children
			1	2	3	1	2	3				
**Residence**												
Rural	28.5	82.5	83.5	78.8	71.6	83.0	78.3	70.8	63.5	56.7	12.4	1,638
Urban	55.7	86.1	85.5	82.9	78.6	84.7	81.8	77.9	70.0	66.3	10.3	577
**Sex of the child**												
Male	36.4	84.3	84.7	81.6	75.6	84.2	81.2	74.7	67.6	61.8	11.3	1,221
Female	34.6	82.4	83.1	77.6	70.7	82.7	76.9	70.1	62.2	56.1	12.7	993
**Birth order**												
1	45.9	89.5	89.8	86.2	81.4	88.9	85.1	80.3	71.9	67.3	7.4	775
2	41.5	86.7	88.2	83.8	76.6	87.6	83.7	76.1	69.3	61.0	7.3	611
3	27.9	83.1	84.7	80.6	73.5	84.3	79.5	71.9	70.1	63.6	13.0	373
4+	16.5	69.0	68.0	63.1	55.4	68.2	63.1	55.6	44.3	39.5	24.7	456
**Mother’s education**												
Non-literate	19.8	69.9	71.1	65.4	56.8	70.6	64.8	56.3	45.6	39.6	23.0	921
0-9@ years	31.9	89.4	89.7	86.0	80.4	89.5	85.4	79.4	72.2	65.3	6.3	742
10 years and above	67.0	98.0	97.9	95.8	91.6	96.8	95.1	90.9	88.6	83.7	0.8	551
**Religion**												
Hindu	37.2	86.9	87.9	84.0	77.7	87.3	83.3	76.9	68.6	62.9	8.6	1,903
Muslim	10.7	45.3	42.7	36.1	29.2	42.1	35.8	29.2	25.2	18.0	46.8	204
Sikh	55.0	94.4	93.5	88.9	80.3	94.7	89.7	78.9	80.8	71.2	2.2	96
**Caste/tribe#**												
Scheduled caste	24.9	84.2	85.4	81.1	76.0	84.9	80.3	75.1	66.1	60.0	10.1	560
Other backward class	27.6	76.4	75.4	71.7	64.9	74.9	71.5	64.8	56.9	51.6	19.4	770
Other	49.3	89.0	90.7	86.2	79.4	90.2	85.3	78.1	71.7	65.3	6.6	872
**Standard of living index**												
Low	12.8	62.5	63.2	55.2	45.7	62.4	54.7	45.3	37.9	30.6	29.2	458
Medium	28.4	85.1	85.5	81.6	74.6	85.3	81.0	73.3	64.2	57.9	9.9	1,018
High	59.7	94.0	94.9	92.7	89.0	94.0	92.1	88.7	83.5	78.9	3.9	738
Total	35.6	83.4	84.0	79.8	73.4	84.0	79.2	72.7	65.2	59.2	11.9	2,214

Note: Table includes only last and last but one living child born since 1.1.1999/1.1.2001.

@ Literate mothers with no years of schooling are included. # Total figure may not add to N due to do not know and missing cases.

[1] BCG, three injection of DPT, three doses of Polio (excluding Polio 0) and measles, Total includes 11 cases on other religion and 12 children in the schedule tribe category who were not shown separately

The data indicates that the coverage of each type of vaccine is more in urban areas than in rural areas. Fifty seven percent of the children in rural areas had received all the recommended vaccinations by the time of the survey, compared with 66 percent in urban areas. Differentials in rural-urban against polio 0 may be observed from the table. Fifty six percent of the children have received polio vaccine at the time of birth in urban areas whereas just half of it received the same in the rural areas.

Figure 5.2
Percentage of Children Age 12-23 Months who Have Received Specific Vaccinations, Haryana

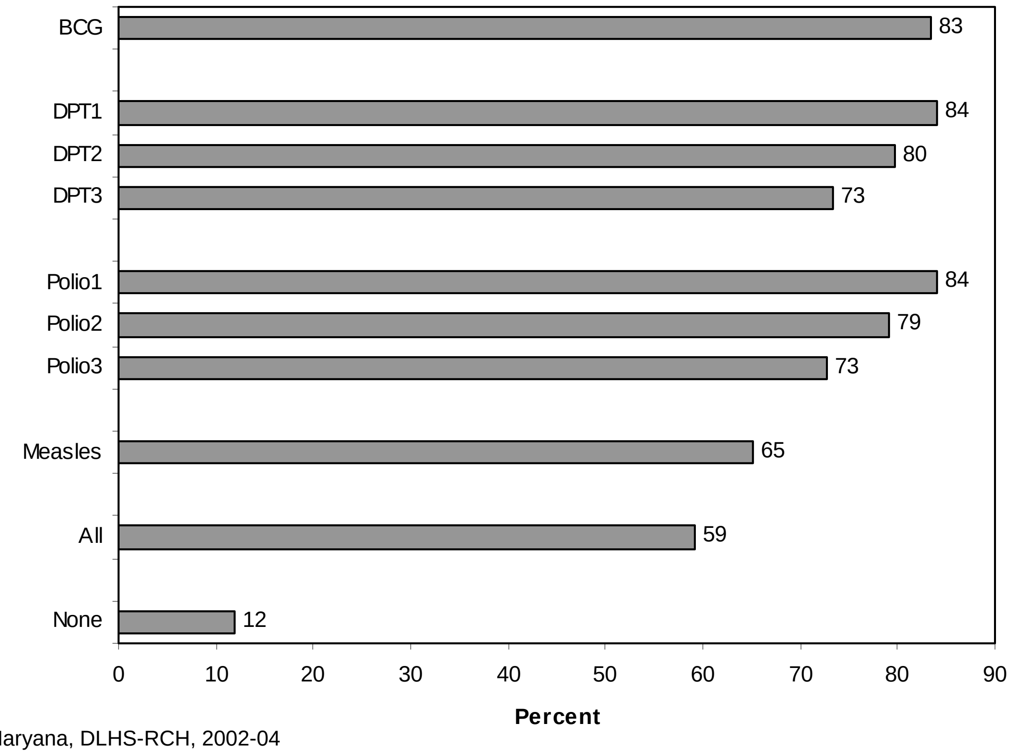

Haryana, DLHS-RCH, 2002-04

Male children (62 percent) are more likely than female children (56 percent) to be fully vaccinated. Male children are also much more likely than female children to have received most of the individual vaccinations. The relationship between vaccination coverage and birth order is consistently negative for almost all vaccinations. A large majority of first-order births occur to younger women who are more likely than older women to utilize child health care services. As with the use of child health care services, there is a positive relationship between mother's education and children's vaccination coverage. Only 40 percent children of non-literate mothers are fully vaccinated compared to 65 percent of children with mothers' education below high school and 84 percent of mothers who have at least completed high school. Sikh and Hindu children are much more likely than Muslim children to have received each of the recommended vaccinations. Children from other castes are more likely to have all the vaccinations. The standard of living index of the household has a strong positive relationship with vaccination

coverage. Seventy nine percent of children from households with a high standard of living are fully vaccinated, whereas only 31 percent of children are from households with a low standard of living.

Figure 5.3
Percentageof Children Age12-23 Months who Have Received All Vaccination, Haryana

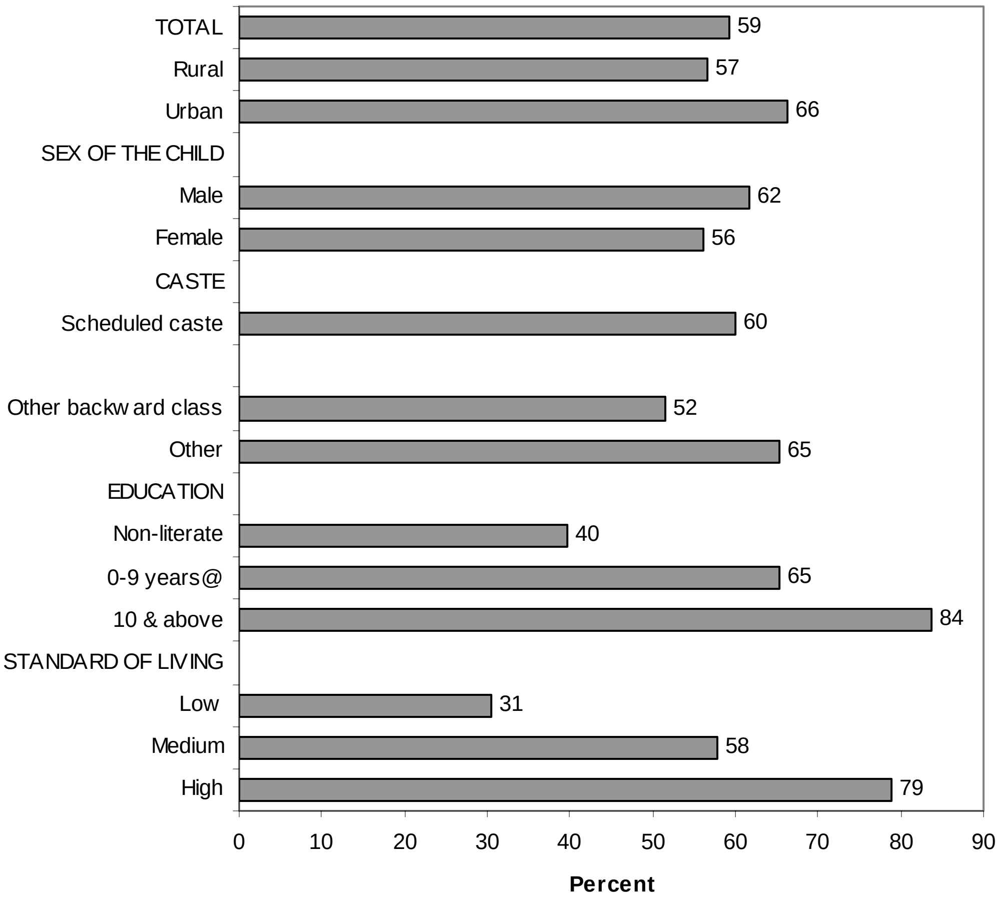

@ Literate mothers with no years of schooling are also included

Haryana, DLHS-RCH, 2002-04

Table 5.5 shows the percentage of children in the age group 12-23 months and 24-35 months with a vaccination card, and the percentage who received various vaccinations during the first year of life by current age of children and place of residence. The interviewer was shown this vaccination card.

The proportion of children fully vaccinated by age 12 months increased slightly from 59 percent for children in the age group 12-23 months to 66 percent for children in the age group 24-35 months. A rural-urban differential for the coverage of full vaccination is also observed. Fifty seven percent of children in the age group 12-23 months are fully vaccinated against 63 percent of children in the age group 24-35 months in rural areas, and this gap is much wider in urban areas (Figure 5.4). Sixty six percent of children in the age group 12-23 months have received all vaccinations in urban areas compared to 76 percent with children in the age group

24-35 months. Younger children aged 12-23 months are more likely to receive each type of vaccine except measles.

Table 5.5 CHILDHOOD VACCINATION RECEIVED BY 12 MONTHS OF AGE

Percentage of children age 12-23 months and 24-35 months with a vaccination card that shown to the interviewer and percentage who received specific vaccinations by 12 months of age according to residence, Haryana, 2002-04

Vaccination status	Total		Rural		Urban	
	12-23 months	24-35 months	12-23 months	24-35 months	12-23 months	24-35 months
Vaccination card shown to interviewer	25.3	17.6	23.2	14.5	31.0	26.2
Percentage vaccinated by 12 months of age						
Polio 0	35.6	33.5	28.5	26.3	55.7	53.6
BCG	83.4	83.2	82.5	82.3	86.1	85.6
**DPT injection**						
No DPT	15.5	14.0	16.0	14.4	13.9	12.8
1	4.2	2.7	4.7	3.2	2.6	1.3
2	6.4	5.1	7.2	6.2	4.3	2.1
3	73.4	77.9	71.6	75.9	78.6	83.5
Don't remember	0.5	0.4	0.4	0.4	0.7	0.5
**Polio doses**						
No Polio	15.8	14.2	16.0	14.7	14.6	12.8
1	4.2	2.6	4.7	3.3	2.9	0.9
2	6.6	5.5	7.6	6.8	3.9	2.1
3	72.8	77.2	70.9	74.9	77.9	83.5
Don't remember	0.6	0.4	0.6	0.4	0.7	0.6
Measles	65.2	72.9	63.5	70.8	70.0	79.0
Full [1] vaccination	59.2	66.3	56.7	62.7	66.3	76.3
No vaccination at all	11.9	11.9	12.4	11.8	10.3	12.2
Number of children	2,214	2,389	1,638	1,758	577	631

Note: Table includes only last and last but one living child born since 1.1.1999/1.1.2001

[1] BCG, three injection of DPT, three doses of Polio (excluding Polio 0) and measles

Figure 5.4
Child Vaccination by Age

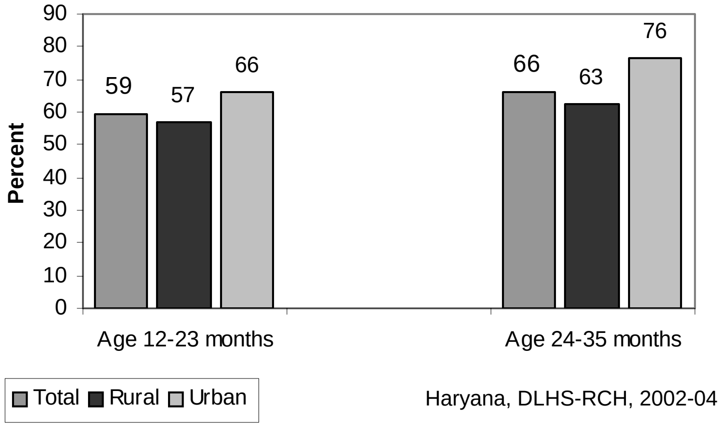

5.3 Source of Immunization

Table 5.6 gives the percent distribution of children under three years of age who have received any vaccination by the source of last vaccine, according to place of residence and availability of health facilities in the village. The sub-centre is the primary provider of childhood vaccinations in Haryana. Most of the children (66 percent) were immunized at the government health facilities and only 9 percent at private health facilities. Further, among the children immunized, 30 percent of them had received vaccination from the sub-centre, 21 percent from municipal hospital and 14 percent from community health centre or from primary health centre. The percentage of children receiving vaccination from the private sector is considerably lower in rural areas (4 percent) than in urban areas (21 percent). Even in urban areas, however, 68 percent of children received their vaccination from the government health facility. Children from those villages where health facilities are available are more likely to receive vaccination from the government health facility.

Table 5.6 SOURCE OF CHILDHOOD VACCINATION

Percent distribution of children under age 3 who have received any vaccination by source of last vaccination, according to place of residence and availability of health facilities in the village, Haryana, 2002-04

Source of vaccination	Total	Residence		Availability of health facility[1] in the village	
		Rural	Urban	No	Yes
**Government health sector**					
Government/municipal hospital	20.9	10.2	50.2	10.9	9.8
Community/primary health centre	13.6	14.9	10.1	8.7	18.3
Sub-centre	30.1	38.4	7.6	28.2	44.0
RCH/MCP camp	1.8	2.3	0.6	2.6	2.1
**Private health sector**					
Private hospital	4.5	2.1	11.2	2.3	2.0
Private doctor	4.3	2.2	9.9	2.6	2.0
ISM[2] health facility	0.9	0.6	1.6	0.4	0.7
Other	22.4	27.6	8.5	41.4	20.0
Do not remember	1.3	1.6	0.2	2.8	1.0
Missing	0.1	0.1	0.0	0.1	0.0
Total percent	100.0	100.0	100.0	100.0	100.0
Number of children	5,875	4,295	1,580	1,522	2,773

Note: Table includes last and last but one living children born in the three years preceding the survey
[1] Includes sub-centre, primary health centre, Community health centre or referral hospital, government hospital, and government dispensary within the village.
[2] Either government or private health facility of Indian System of Medicine

5.4 Reason for Not Immunizing the Children

Table 5.7 presents the percent distribution of children under the age of three years who did not receive any vaccination by reason as reported by the mother according to place of residence and availability of health facilities in the village. About 30 percent of the children did not receive any vaccination because the mothers of children were unaware of the need for immunization and 24 percent of children were not vaccinated, as the mothers feel that they were too young. The other reasons for not immunizing the children as reported by the mothers were fear of side effects (7 percent), place or time of vaccination was not known (13 percent), place or time of vaccination was inconvenient and ANM absent / vaccine not available (5 percent each),

no faith in vaccination (4 percent) and other reasons (3 percent). The percentage of children who did not receive any vaccinations is considerably lower in rural areas (29 percent) than in urban areas (33 percent), as they were unaware of the need for immunization as reported by their mothers. Children from those villages where health facilities are available are less likely to report that they were unaware of the need for immunization as compared to those villages where health facilities are not available. Where health facilities were available, place or time of vaccination was inconvenient, ANM absent/ vaccine not available; child too young, family problems and other reasons were reported more as reasons for not immunizing the children compared to the areas without having the same.

Table 5.7 REASON FOR NOT GIVING VACCINATION

Percent distribution of children under age 3 who did not receive any vaccination by reason reported by mother for not giving vaccination, according to place of residence and availability of health facilities in the village, Haryana, 2002-04

Reason	Total	Residence		Availability of health facility[1] in the village	
		Rural	Urban	No	Yes
Unaware of need for immunization	30.2	29.2	33.3	34.3	25.2
Place/time unknown	13.3	12.8	14.4	14.9	11.4
Place/time inconvenient	4.6	4.3	5.6	2.9	5.4
Fear of side effect	7.2	7.8	5.1	8.8	7.1
No faith	3.8	3.9	3.7	5.9	2.3
ANM absent/vaccine not	4.7	5.4	2.4	5.4	5.4
Long waiting time	0.6	0.1	2.0	0.0	0.2
Child too young	24.3	24.9	22.2	18.5	30.0
Family problems	8.3	8.6	7.5	7.4	9.8
Other	2.5	2.4	3.2	2.1	2.6
Total percent	100.0	100.0	100.0	100.0	100.0
Number of children	1,092	838	254	369	469

Note: Table includes last and last but one living children born in the three years preceding the survey
[1] Includes sub-centre, primary health centre, Community health centre or referral hospital, government hospital, and government dispensary within the village
[2] Includes mother too busy, family problems, including illness of mother, and illness of child

5.5 Vitamin A and IFA Supplements

Vitamin A deficiency is one of the most common nutritional deficiency disorders in the world, affecting more than 250 million children worldwide (Bolem et. al., 1997). The child survival programme also includes administration of five doses of Vitamin A for prevention of night blindness and distribution of IFA for iron supplement. In Round II, mothers of children born during the three years before the survey were asked whether their children had received a dose of Vitamin A and IFA tablets/syrup. Those who said that their children had received a dose of Vitamin A and IFA tablets/syrup were further asked how many doses were given. Table 5.8 shows the percentage of children in the age group 12-35 months who received at least one dose of Vitamin A and IFA tablets/syrup by selected background characteristics. In the state of Haryana as a whole, 38 percent of the children received at least one dose of Vitamin A and only 6 percent received IFA tablets/syrup. This indicates that a large number of children in Haryana did not receive Vitamin A supplements and very few children received IFA tablets/syrup supplementation.

Table 5.8 VITAMIN A AND IFA SUPPLEMENTATION FOR CHILDREN

Percentage of children age 12-35 months who have received at least one dose of Vitamin A and iron folic acid tablets / syrup, according to selected background characteristics, Haryana, 2002-04

Background characteristic	Percentage who received at least one dose of vitamin A	Percentage who received iron folic acid tablets/syrup	Number of children
**Age of the child**			
12-23 months	39.8	5.2	2,214
24-35 months	43.9	5.9	2,389
**Sex of the child**			
Male	42.2	5.7	2,549
Female	41.6	5.5	2,054
**Birth order**			
1	47.1	5.8	1,507
2	45.2	6.6	1,293
3	41.3	5.1	850
4+	29.8	4.3	953
**Residence**			
Rural	39.3	5.4	3,395
Urban	49.2	6.1	1,208
**Mother's education**			
Non-literate	27.4	3.8	1,865
0-9 years@	46.6	5.9	1,610
10 years and above	59.4	8.1	1,128
**Religion**			
Hindu	43.3	5.9	3,990
Muslim	17.9	3.1	407
Sikh	63.2	4.3	192
**Caste/tribe #**			
Scheduled caste	39.9	5.2	1,177
Scheduled tribe	(24.5)	(13.8)	32
Other backward class	36.1	5.0	1,598
Other	48.4	6.1	1,797
**Standard of living index**			
Low	24.5	2.8	975
Medium	40.4	6.2	2,073
High	54.8	6.5	1,555
**Availability of health facility in the village**[1]			
Yes	40.0	5.9	2,154
No	38.1	4.5	1,242
Total	38.3	5.6	4,603

Note: Table includes last and last but one living children born in the three years preceding the survey.
@ Literate mother with no years of schooling are also included here. # Total figure may not add to N due to do not know and missing cases. [1] Includes sub-centre, primary health centre, Community health centre or referral hospital, government hospital, and government dispensary within the village. Total includes 15 cases on other religion who were not shown separately. ( ): Based on less than 50 unweighted cases.

Children in the age group 24-35 months are more likely to receive at least one dose of Vitamin A and IFA tablets/syrup each than children in the age group 12-23 months. Male children are more likely to receive Vitamin A and IFA tablets/syrup than female children. Children living in urban areas, children whose mother completed high school and above, children living in households with a high standard of living and children living in those villages

where health facilities are available are more likely to receive a dose of Vitamin A and IFA tablets/syrup. Children of birth order 4 or above are much less likely than children of birth order 1, 2 or 3 to receive any dose of vitamin A and IFA tablets/syrup. Similarly, children from Schedule Tribes are less likely to receive at least one dose of Vitamin A and a dose of IFA tablets/syrup than other caste category.

Table 5.9 CHILDHOOD VACCINATION BY DSITRICT

Percentage of children age 12-35 months with a vaccination card that shown to the interviewer and percentage who received specific vaccinations by district, Haryana, 2002-04

District	Percentage vaccinated							Percentage received at least one does of Vitamin A[3]
	Polio 0	BCG	DPT3	Polio3	Measles	Full[1]	None	
Ambala	61.2	99.0	97.7	97.7	93.8	92.9	0.0	85.9
Bhiwani	31.8	80.4	65.9	62.3	54.0	47.8	15.0	29.6
Faridabad	31.5	75.4	60.4	62.0	61.1	53.7	21.6	29.0
Fatehabad	13.5	84.4	77.6	78.6	73.2	65.1	8.4	26.0
Gurgaon	21.5	59.9	46.7	46.7	39.0	32.9	33.9	21.5
Hisar	35.9	90.1	80.7	79.8	64.5	63.6	7.3	65.9
Jhajjar	41.9	90.7	81.7	79.8	73.7	68.0	5.4	25.3
Jind	27.9	87.0	74.2	75.0	68.2	62.4	8.6	53.5
Kaithal	59.0	73.2	60.6	58.4	52.4	41.4	12.8	33.1
Karnal	27.8	96.3	91.6	88.7	80.0	73.9	1.1	28.2
Kurukshetra	49.2	96.5	88.0	87.1	82.6	81.7	3.5	61.6
Mahendragarh	37.8	79.4	64.7	64.6	58.2	52.6	13.7	38.6
Panchkula	50.2	98.1	92.5	92.5	89.5	85.7	1.5	66.9
Panipat	51.9	81.1	70.1	70.1	69.8	63.8	12.8	38.2
Rewari	27.3	91.8	81.5	79.4	75.2	65.8	3.8	22.9
Rohtak	42.4	97.8	90.1	90.9	82.6	81.0	0.9	40.4
Sirsa	40.0	80.1	70.5	69.7	56.8	49.1	12.3	37.4
Sonipat	34.5	85.8	83.8	80.3	66.3	55.1	7.2	22.0
Yamunanagar	30.1	96.3	85.9	85.0	77.0	72.0	2.5	62.4
Haryana	34.5	83.3	75.7	75.0	69.2	62.9	11.9	38.3

Note: Table includes only last and last but one living child born since 1.1.1999/1.1.2001
[1] BCG, three injection of DPT, three doses of Polio (excluding Polio 0) and measles

5.6 Immunization Coverage by District

The coverage of vaccination rates for all vaccines for children in the age group 12-23 months in each district is presented in Table 5.9. There are inter-district differentials in the coverage for different vaccinations, and for children receiving all vaccinations and those that did not receive any vaccination at all. The percentage of children who are fully vaccinated ranges from 33 percent in Gurgaon to 93 percent in Ambala. In the districts Bhiwani (48 percent), Gurgaon (33 percent), Kaithal (41 percent) and Sirsa (49 percent), the coverage of full immunization is below 50 percent (see Map-5). Thirty four and 22 percent of children in Gurgaon and Faridabad districts respectively were not vaccinated at all. In most of the districts, fewer children have received the measles vaccine than any of the other vaccinations except Polio 0. The coverage of polio drops at the time of birth varies from the lowest in 14 percent in Fatehabad to the highest in Ambala (61 percent).

District wise variations in the percentage of children who received at least one dose of Vitamin A are also shown in Table 5.9. The percentage of children in the age group 12-35 months who received at least one dose of Vitamin ‘A’ supplements ranges from 22 percent in Gurgaon to 86 percent in Ambala. A total of 10 out of 19 districts stand out as having below the state average to receive at least one dose of Vitamin A.

5.7 Child Morbidity and Treatment

This section discusses the awareness, prevalence and treatment of diarrhoea and acute respiratory infection (ARI). Mothers of surviving children born during the three years preceding the survey were asked if their children suffered from cough and cold or diarrhoea during the two weeks preceding the survey, and if so, the type of treatment that had been given. Accuracy of all these measures is affected by the reliability of the mother’s recall of when the diseases occurred.

5.7.1 Awareness of Diarrhoea

Diarrhoea is a major killer disease of children under five years of age. Deaths from acute diarrhoea are mostly due to dehydration resulting from loss of water and electrolytes. An attempt was made to collect data on awareness of diarrhoea management and the practice followed during the episode of diarrhoea. This has been presented in Table 5.10.

In Haryana, 72 percent of the mothers with births three years preceding the survey were aware of what to do when a child had diarrhoea, as compared to 69 percent in Round I, and 30 percent were aware of ORS, which was three percent point down from Round I. Thirty three percent of the women were aware of salt and sugar solution. Some of the women also reported that they would continue normal food (8 percent), continue breastfeeding (4 percent) and give plenty of fluids (10 percent) and about 29 percent of women did not know what to give a child who had diarrhoea. As expected, knowledge of ORS is higher among urban women (44 percent) than rural women (24 percent) and among high school and above educated women (63 percent) as compared to non-literate women (9 percent). Women belonging to Schedule caste (22 percent) are less likely to know about ORS than women belonging to other caste groups (39 percent). Forty nine percent of women with children having a high standard of living know about ORS and it declines to 24 percent for women with a medium standard of living and 9 percent with a low standard of living. Knowledge of ORS is more among middle age groups and among younger women than among older women. Women from villages with availability of health facilities are equally aware of diarrhoea management than women from other villages.

Table 5.10 AWARENESS OF DIARRHOEA

Percentage of women who are aware of diarrhoea management, type of practice followed if child gets diarrhoea, and percentage of women whose child suffered[1] from diarrhoea by selected background characteristics, Haryana, 2002-04

Background Characteristic	Knowledge of diarrhoea manage-ment	Type of practices to be followed do if child gets diarrhoea*					Do not know	Number of women
		Give ORS	Salt and sugar solution	Continue normal food	Continue breastfeding	Give plenty of fluids		
**Age**								
15-24	71.5	28.6	31.3	6.9	3.6	8.7	28.7	3,489
25-34	73.1	32.6	36.7	9.4	4.7	12.6	27.3	2,790
35-44	63.0	16.7	25.0	4.5	2.4	8.6	37.5	351
**Residence**								
Rural	69.0	24.1	28.5	6.7	3.4	9.3	31.3	4,826
Urban	79.1	44.4	46.0	10.9	5.5	13.3	21.3	1,804
**Mother's education**								
Non-literate	60.1	8.9	16.7	5.0	2.7	5.7	40.3	2,619
0-9@ years	72.8	27.8	34.2	7.5	3.4	9.4	27.4	2,244
10 and above	87.6	62.8	56.5	12.4	6.7	18.5	12.6	1,765
**Religion**								
Hindu	72.6	31.6	35.6	8.5	4.1	10.8	27.7	5,751
Muslim	62.5	5.7	11.2	2.3	2.2	4.6	37.8	562
Sikh	71.1	33.8	27.4	5.8	4.4	11.4	29.9	295
**Caste/tribe#**								
Scheduled caste	68.8	22.4	27.6	6.2	3.3	8.1	31.5	1,631
Scheduled tribe	(65.1)	(31.0)	(35.7)	(7.1)	(4.8)	(26.2)	(31.0)	48
Other backward class	69.6	23.8	28.3	6.8	3.0	7.8	30.8	2,255
Other	75.4	38.9	40.6	9.7	5.4	13.5	24.9	2,695
**Standard of living index**								
Low	59.7	7.8	14.8	3.5	2.4	5.3	40.7	1,354
Medium	68.6	24.4	30.5	6.7	2.8	8.8	31.7	2,919
High	82.5	48.6	47.3	11.6	6.4	15.2	17.7	2,358
**Availability of health facility [2] in the village**								
Yes	69.1	26.1	29.3	6.8	3.5	9.5	31.1	3,057
No	68.7	20.7	27.1	6.5	3.4	8.9	31.6	1,769
Total	71.7	29.6	33.2	7.8	4.0	10.4	28.6	6,630

Note: Table based on women with living children born since 01.01.1999 for phase - I /01.01.2001 for phase - II.
[1]Last two weeks prior to survey.
@ Literate mother with no years of schooling are included. # Total figure may not add to N due to do not know and missing cases.
[2]Includes sub-centre, primary health canter, Community health centre or referral hospital, government hospital, and government dispensary within the village. Total includes 22 women on other religion and 2 women missing information on education who were not shown separately
( ) Based on less than 50 unweighted cases.

5.7.2 Treatment of Diarrhoea

During the two weeks before the survey, 18 percent of the women reported that their children suffered from diarrhoea (Table 5.11). Women, whose children had diarrhoea, were further asked about treatment with ORS, any other medical treatment and source of treatment. About 32 percent of the women mentioned that they gave ORS therapy and 78 percent of the women said that their child had been treated at health facility. Use of ORS for the treatment of childhood diarrhoea in Haryana is relatively high among urban women than among rural women.

It was observed that a relatively high proportion of women from those villages where health facilities are available within the village used ORS for the treatment of childhood diarrhoea.

Table 5.11 TREATMENT OF DIARRHOEA

Percentage of women who sought treatment whose child suffered from diarrhoea and by source of treatment, according to place of residence and availability of health facility in the village, Haryana, 2002-04

Sought treatment / source of treatment	Total	Residence		Availability of health fcaility[2] in the village	
		Rural	Urban	Yes	No
Percentage of women whose child suffered[1] from diarrhoea	18.1	18.0	18.6	19.1	16.0
Number of women	6,630	4,826	1,804	3,057	1,769
Percentage of women whose child suffered[1] from diarrhoea treated with ORS	32.1	29.1	40.1	29.4	28.4
Percentage of women whose child suffered[1] from diarrhoea sought treatment	78.3	76.0	84.2	77.4	73.1
Number of women	1,203	868	336	585	283
**Source of treatment**					
**Government health facility**					
Hospital/dispensary	4.2	4.7	3.3	5.5	2.9
UHC/UHP/UFWC	0.1	0.1	0.0	0.2	0.0
CHC/ Rural hospital	0.8	1.1	0.2	1.4	0.5
Primary health centre	2.6	3.6	0.2	3.5	3.8
Sub centre	1.2	1.8	0.0	2.3	0.6
**Private health facility**					
NGO/Trust hospital/clinic	1.0	0.9	1.1	1.2	0.4
Private hospital clinic	80.1	76.3	89.1	73.8	81.7
ISM[3] facility	6.4	6.1	7.3	6.3	5.6
Home remedy	3.9	5.0	1.5	5.6	3.6
Other	5.7	7.4	1.8	7.7	6.7
Percent distribution of women who seek treatment by					
Doctor	93.1	91.5	97.0	89.6	95.6
ANM/Nurse/LHV	3.1	4.2	0.5	5.6	1.2
Relative/friends	0.9	1.2	0.0	1.0	1.8
Chemist/medical shop	2.4	2.7	1.8	3.2	1.4
ISM	0.4	0.3	0.7	0.2	0.0
Missing	0.1	0.1	0.0	0.4	0.0
Total percent	100.0	100.0	100.0	100.0	100.0
Number of women	942	659	283	453	207

Note: Table based on women with living children born since 01.01.1999 for phase - I /01.01.2001 for phase - II.
[1] Last two weeks prior to survey. [2] Includes sub-centre, primary health centre, Community health centre or referral hospital, government hospital, and government dispensary within the village. [3] Either government or private health facility of Indian System of Medicine

Among those mothers whose children suffered from diarrhoea during the last two weeks before the survey and those women who consulted or obtained advice, about 81 percent of women visited private hospitals/clinics and 6 percent of women treated their children through the Indian System of Medicine.

5.7.3 Awareness of Pneumonia

Another major killer disease among infants and children is Acute Respiratory Infections (ARI) including pneumonia. Early diagnosis and treatment with antibiotics can prevent a large proportion of ARI/pneumonia deaths. An attempt was made to understand the awareness level of pneumonia, and the proportion of children who had suffered from pneumonia during the last two weeks before the survey and their health seeking behaviour. This is presented in Table 5.12. It was found that half of the women (50 percent) with births three years preceding the survey in Haryana were aware of danger signs of pneumonia. The figure was slightly down from 60 percent in Round I. A relatively high proportion of women in urban areas (53 percent) were aware of the danger signs of pneumonia as compared to women from rural areas (49 percent). Knowledge of danger signs of pneumonia is higher among 25-34 year old women of (55 percent), women from Muslim religion (54 percent), other castes category (52 percent), highly educated women (59 percent), women living in high standard of living household (56 percent) and women living in those villages without health facilities (51 percent).

Women, who were aware of the danger signs of pneumonia, were further asked about different types of signs of pneumonia. Most of the women mentioned about 'difficulty in breathing' (71 percent), 'chest in drawing' (63 percent), 'pain in chest and productive cough' (37 percent), 'wheezing / whistling' (29 percent), 'rapid breathing' (24 percent), 'conditions get worse than before' (17 percent), 'not able to drink or take a feed' (14 percent) and 'excessive drowsy and difficulty in keeping awake' (11 percent).

5.7.4 Treatment of Pneumonia

About 11 percent of women reported that their child had suffered from pneumonia during two weeks before the survey; the corresponding figures were 11 percent in rural areas and 10 percent in urban areas (Table 5.13). The incidence of pneumonia varies little with availability of health facilities in the villages.

Table 5.13 also shows that the percentage of women whose children suffered from ARI symptoms in the last two weeks before the survey who sought advice/treatment and taken to a health facility or provider. Seventy eight percent of women received some advice or treatment whose children were ill with ARI. This percentage is relatively low in rural areas (77 percent) than in urban areas (80 percent) and village without health facilities (73 percent) than village with health facility (80 percent).

Among them who got advice for children ill with ARI, 77 percent of women visited private hospital/clinic, and only 13 percent went to government hospital/dispensary, whereas 4 percent of them obtained treatment through Indian System of Medicine.

Table 5.12 AWARENESS OF PNEUMONIA

Percentage of women who are aware of danger signs of pneumonia by signs by selected background characteristics and availability of health facility in the village, Haryana, 2002-04

Background Characteristic	Percentage of women aware of danger signs of pneumonia	Number of women	Danger signs: Difficulty in breathing	Chest in-drawing	Not able to drink or take a feeding	Excessive drowsy and difficulty in keeping awake	Pain in chest and productive cough	Conditions get worse than before	Wheezing/ whistling	Rapid breathing	Number of women
**Age**											
15- 24	45.7	3,489	70.5	63.1	13.3	9.8	35.9	17.9	29.4	25.5	1,595
25-34	54.5	2,790	73.1	62.6	15.9	11.7	38.0	16.1	29.2	24.3	1,520
35-44	52.8	351	64.6	69.5	8.9	11.2	32.6	12.0	27.8	14.1	185
**Residence**											
Rural	48.5	4,826	70.2	62.5	13.2	9.9	36.1	17.1	29.6	23.3	2,341
Urban	53.1	1,804	74.2	64.9	16.8	12.8	38.2	15.8	28.3	26.7	959
**Mother's education**											
Non-literate	45.3	2,619	64.1	65.9	11.0	7.6	32.8	13.6	26.3	18.9	1,187
0-9@ years	47.9	2,244	73.6	59.3	13.9	10.2	36.5	16.6	31.6	25.7	1,074
10 and above	58.8	1,765	77.4	64.2	18.3	15.0	41.5	20.5	30.1	29.0	1,038
**Religion**											
Hindu	49.8	5,751	73.4	62.2	14.6	11.4	38.1	17.0	30.7	24.9	2,866
Muslim	54.0	562	44.7	79.4	8.0	4.3	20.9	12.5	14.2	14.2	302
Sikh	41.3	295	89.7	47.2	19.1	12.3	44.4	19.7	31.2	35.9	122
**Caste/tribe#**											
Scheduled caste	46.3	1,631	72.1	62.0	11.4	8.9	37.6	14.3	28.6	25.5	755
Scheduled tribe	(51.2)	48	(63.6)	(77.3)	(4.5)	(4.5)	(40.9)	(9.1)	(27.3)	(9.1)	26
Other backward class	49.2	2,255	64.9	65.7	10.9	8.4	31.5	16.6	26.5	21.3	1,109
Other	52.3	2,695	76.4	61.6	18.5	13.7	40.2	18.2	31.8	26.2	1,410
**Standard of living index**											
Low	43.8	1,354	59.2	66.4	7.7	6.7	27.1	12.4	25.4	18.8	593
Medium	47.5	2,919	72.4	62.4	13.5	9.2	38.7	17.4	29.5	23.0	1,387
High	56.0	2,358	75.8	62.7	17.9	14.2	38.9	18.0	30.6	28.1	1,320
**Availability of health facility [2] in the village**											
Yes	47.1	3,057	71.5	62.5	13.5	10.5	36.6	17.3	28.9	22.2	1,439
No	51.0	1,769	68.0	62.6	12.7	9.1	35.4	16.9	30.6	25.1	902
Total	49.8	6,630	71.4	63.2	14.2	10.8	36.7	16.7	29.2	24.3	3,300

Note: Table based on women with living children born since 01.01.1999 for phase - I /01.01.2001 for phase - II. [1] Last two weeks prior to survey.
@ Literate mother with no years of schooling are included. # Total figure may not add to N due to do not know and missing cases.
[2] Includes sub-centre, primary health centre, Community health centre or referral hospital, government hospital, and government dispensary within the village.
Total includes 2 women with missing information on education and 22 women on other religion who are not shown separately.
() Based on less than 50 unweighted cases.

Table 5.13 TREATMENT OF PNEUMONIA<br>Percentage of women who sought treatment whose child suffered[1] from cough and cold and source of treatment, according to place of residence and availability of health facility in the village, Haryana, 2002-04					
Sought treatment/ source of treatment	Total	Residence		Availability of health facility[2] in the village	
		Rural	Urban	Yes	No
Percentage of women whose child suffered from cough, cold and difficulty in breathing	10.8	11.3	9.6	11.5	10.9
Number of women	6,630	4,826	1,804	3,057	1,769
Percentage of women sought treatment whose child suffered from cough and cold	77.9	77.3	79.7	79.6	73.3
Number of women	717	544	172	352	193
**Source of treatment**					
**Government health facility**					
Hospital/dispensary	5.9	6.2	4.8	7.2	4.4
+UHC/UHP/UFWC	0.6	0.7	0.2	0.3	1.3
CHC/ Rural hospital	1.4	1.8	0.1	1.6	2.2
Primary health centre	4.7	5.7	1.4	6.6	4.0
Sub centre	0.3	0.4	0.0	0.3	0.6
**Private health facility**					
NGO/Trust hospital/clinic	0.7	0.6	0.7	0.7	0.6
Private hospital clinic	76.2	74.2	82.2	74.2	74.1
ISM[3] facility	4.2	5.4	0.7	4.2	7.6
Home remedy	3.8	2.9	6.4	2.9	2.9
Other	5.1	5.4	4.3	4.8	6.5
Percent distribution of women who seek treatment by					
Doctor	94.1	94.0	94.3	92.9	96.3
ANM/Nurse/LHV	1.6	2.1	0.0	2.0	2.3
Relative/friends	1.3	0.2	4.4	0.4	0.0
Chemist/medical shop	2.6	2.9	1.4	4.4	0.0
ISM practitioner	0.6	0.7	0.0	0.4	1.5
Total percent	100.0	100.0	100.0	100.0	100.0
Number of women	558	421	137	280	141

Note: Table based on women with living children born since 01.01.1999 for phase - I /01.01.2001 for phase - II.
[1]Last two weeks prior to survey. [2] Includes sub-centre, primary health centre, Community health centre or referral hospital, government hospital, and government dispensary within the village. [3] Either government or private health facility of Indian System of Medicine

5.7.5 Awareness of Diarrhoea, ORS and Pneumonia and Incidence of Diarrhoea and Pneumonia by District

Table 5.14 presents the knowledge of diarrhoea management, knowledge of ORS, and incidence of diarrhoea by district. Although knowledge of diarrhoea management is high in almost all districts but knowledge about ORS is low. Knowledge of ORS is also not common and it is lowest in Gurgaon (13 percent). The incidence of diarrhoea is 18 percent in the state as a whole and it varies from 6 percent each in Hisar and Fatehabad to 35 percent in Kaithal. Table 5.14 also shows differentials in the awareness of danger signs of pneumonia and incidence of pneumonia. In comparison to awareness about diarrhoea management, the awareness of danger

signs of pneumonia is quite low. It is the lowest in Bhiwani (28 percent) and highest in Karnal (79 percent). Incidence of ARI symptoms is comparatively low in most of the districts in Haryana. It is highest in Gurgaon and Hisar (26 percent) and lowest in Faridabad (2 percent).

Table 5.14 KNOWLEDGE OF DIARRHOEA MANAGEMENT AND PNEUMONIA BY DISTRICT

Percentage of women by awareness of diarrhoea management, ORS, danger signs of pneumonia and whose child had suffered from diarrhoea and pneumonia during last two weeks prior to survey by district, Haryana, 2002-04

District	Percentage of women aware of		Percentage of women whose child suffered[1] from diarrhoea	Percentage of women aware of danger signs of pneumonia	Percentage of women whose child suffered[1] from pneumonia
	Diarrhoea Management	ORS			
Ambala	85.3	28.7	12.7	44.4	5.1
Bhiwani	72.2	23.2	10.0	27.9	6.0
Faridabad	67.3	25.0	18.2	60.9	1.8
Fatehabad	92.8	28.0	(5.9)	40.0	3.1
Gurgaon	77.2	12.6	28.3	36.0	26.3
Hisar	65.9	29.1	(5.8)	55.8	25.9
Jhajjar	77.0	45.2	14.6	47.1	13.5
Jind	60.6	19.1	8.4	63.4	18.0
Kaithal	58.2	22.0	34.9	60.5	5.1
Karnal	84.3	43.3	28.1	79.2	7.2
Kurukshetra	64.1	39.3	15.8	33.9	11.9
Mahendragarh	57.4	24.8	13.6	30.9	4.9
Panchkula	75.8	38.4	12.2	43.2	9.3
Panipat	77.5	31.2	27.3	51.4	11.2
Rewari	91.1	42.6	11.8	55.9	10.8
Rohtak	83.6	50.9	16.6	36.3	13.3
Sirsa	47.8	24.3	20.3	77.6	11.0
Sonipat	81.9	51.6	27.4	52.8	10.9
Yamunanagar	54.7	24.3	14.5	51.4	6.8
Haryana	71.7	29.6	18.1	49.8	10.8

Note: Table based on women with last and last but one living children born since 01.01.1999 /01.01.2001.
[1]Last two weeks prior to survey. () base on less than 50 unweighted cases.

Under the RCH programme, the government health facilities are strengthened to provide treatment of ARI. However, the percentage of women who visited to a government health facility for treatment of their children sick with ARI symptoms was very low.

MAP-5
Percentage of Children (Age 12-23 Months) Who Have Received Full Vaccination

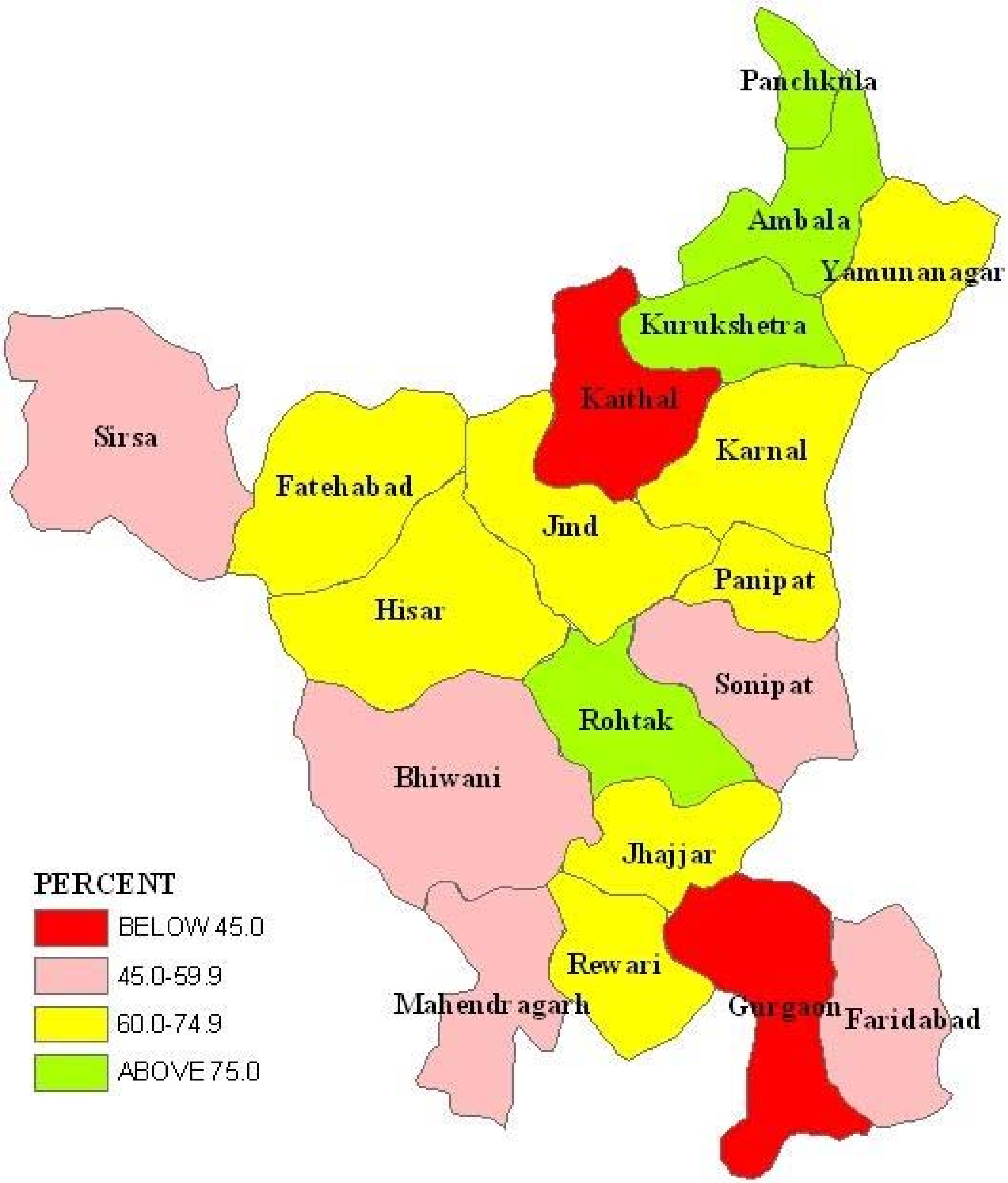

CHAPTER VI

FAMILY PLANNING

The Reproductive and Child Health Programme has been implemented with a new philosophy and direction to meet the health care needs of women and children. It envisages the provision of couples to control their fertility and have sexual relations free from the fear of pregnancy. Provision of free contraceptive services to all the needy couples is one of the components of the RCH programme. In DLHS-RCH a separate section on family planning was canvassed to all the eligible women to assess the knowledge and practice of various family planning methods. The information on source of currently adopted contraceptive method, source of supply of the method and health problems related to contraceptive use were collected from current users. The current non-users were asked about the past status of contraceptive use, reason for not using contraceptives currently and future intention to adopt a family planning method.

An attempt was made to understand why male methods of family planning especially that of vasectomy was not in common use. The husbands of sampled eligible women were asked about the contraceptive method they would recommend to a couple who was not desirous of any additional children. They were also asked about the reasons for not preferring male methods and their knowledge about the no-scalpel vasectomy. This chapter presents the results of data on contraceptive practices collected from both the sampled women and their husbands.

6.1 Knowledge of Family Planning Methods

Lack of knowledge of various contraceptive choices can be a major barrier to promotion and use of contraceptives among couples. In DLHS-RCH information on knowledge of contraceptives was obtained by asking a question, "Which are the family planning methods you know?" to each sampled eligible women. The knowledge of no-scalpel vasectomy was also asked to the husbands of eligible women. If the respondent did not recognise the name of the family planning method, he was given a brief description on how the particular method was to be used. The DLHS-RCH assesses the knowledge of female sterilisation, male sterilisation including NSV, IUD, Pills, condom and traditional methods along similar lines.

The extent of knowledge of contraceptive methods among currently married women for specific methods and selected background characteristics are shown in Table 6.1 and Figure 6.1. Knowledge of any method including any modern contraceptive method is almost universal in the state of Haryana. The knowledge of any method and any modern method do not vary much by residence. The knowledge of modern spacing method among currently married women is around 95 percent, and slightly higher among the women with an urban residence. There are large differentials in knowledge of all modern methods with respect to the aforesaid background characteristics. For instance, 63 percent of women from rural areas are aware about all modern methods compared to 79 percent of their urban counterparts.

Table 6.1 KNOWLEDGE OF CONTRACEPTIVE METHODS

Percentage of currently married women age 15-44 years who know any contraceptive method by specific method and selected background characteristics, Haryana, 2002-04

Contraceptive methods	Total	Residence		Availability of health facility in the village[3]	
		Rural	Urban	No	Yes
**Any method**	99.6	99.4	99.9	99.3	99.5
Any modern method	99.5	99.4	99.8	99.3	99.4
Any modern spacing method[1]	94.5	92.9	98.2	92.6	93.1
All modern methods[2]	67.3	62.5	78.7	61.2	63.3
**Female sterilization**	98.9	98.7	99.4	98.7	98.7
Tubectomy	71.6	69.6	76.6	70.2	69.3
Laparoscopy	48.0	43.7	58.4	43.2	43.9
**Male sterilization**	84.0	81.6	89.8	80.8	82.0
Vasectomy	50.2	46.5	59.0	47.1	46.2
No-scalpel vasectomy	34.2	29.1	46.6	26.8	30.3
**IUD/Loop**	85.5	82.4	93.0	81.0	83.2
**Pills**	91.4	89.2	96.9	88.8	89.4
Daily	74.1	69.6	84.9	68.3	70.3
Weekly	42.1	35.8	57.2	36.7	35.4
Condom/Nirodh	80.9	77.7	88.5	76.8	78.2
Sponge (today)	9.9	6.2	18.8	5.6	6.5
Injectables	28.8	27.5	31.9	26.3	28.1
Norplant	3.0	2.3	4.6	2.5	2.2
Contraceptive herbs	9.3	8.8	10.3	7.8	9.4
Any traditional method	46.7	45.6	49.5	43.1	46.9
Any other Indian system of medicinal contraceptives	0.9	0.6	1.7	0.5	0.7
**Number of women**	18,795	13,306	5,489	4,719	8,587

Note: [1] Include IUD, pills and condom. [2] Include Female sterilization, Male sterilization, IUD, pills and condom.
[3] Includes sub-centre, primary health centre, community health centre or referral hospital, government hospital, and government dispensary within the village.

Female sterilisation is the most widely known method of all contraceptive methods in Haryana followed by Pills. Overall, 99 percent of currently married women are aware of female sterilization and 84 percent knew about male sterilization. There is no rural - urban difference in knowledge of female sterilization but it is not the case of male sterilization. A sizable number of urban women (90 percent) know about male sterilization as compared to 82 percent of rural women. There are differentials in spacing methods such as IUD/Loop, Pill and condom users with respect to the background characteristics. The best-known spacing methods are Pills (91 percent) and condoms (81 percent) respectively. Eighty six percent of women know about the IUD/Loop. There is a large differential in knowledge of spacing methods by residence, only 78 percent of the rural women know condom compared to 89 percent of urban women. The modern spacing methods, Pill and IUD are known by 89 and 82 percent of rural women respectively while the corresponding figures in urban areas are 97 and 93 percent respectively of eligible women respondents. The knowledge of these spacing methods remains low as compared to knowledge of sterilization.

In Haryana, only 47 percent of the women are aware of a traditional method and less than one percent is also aware of other contraceptives of the Indian System of Medicine. It is also observed that women from villages with a health facility are slightly more aware about modern spacing methods.

Figure 6.1
Knowledge of Family Planning Method

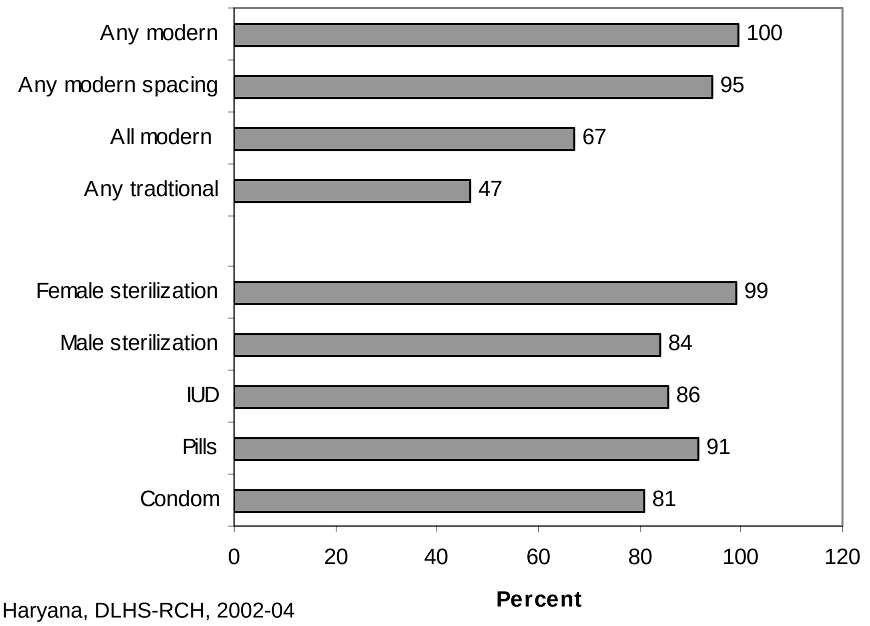

Haryana, DLHS-RCH, 2002-04

Table 6.2 KNOWLEDGE OF CONTRACEPTIVE METHODS BY DISTRICT

Percentage of currently married women age 15-44 years who know any contraceptive method by specific method and district, Haryana, 2002-04

Districts	Any method	Any modern[1] method	Any modern spacing[2] method	All modern[3] methods	Male sterilization	Female sterilization	IUD	Pill	Condom /Nirodh	Any traditional method
Ambala	100.0	100.0	99.9	89.2	95.6	100.0	96.9	98.5	96.7	58.6
Bhiwani	100.0	100.0	96.1	74.8	93.0	100.0	88.1	91.0	85.7	71.6
Faridabad	99.9	99.8	95.2	62.6	84.4	99.8	84.1	93.0	76.8	26.7
Fatehabad	99.9	99.9	95.8	63.8	76.4	99.9	90.3	93.3	81.8	48.9
Gurgaon	99.0	98.9	90.9	67.0	89.3	98.5	80.2	87.5	76.2	72.3
Hisar	100.0	100.0	99.5	87.2	95.7	100.0	95.0	97.4	95.8	50.2
Jhajjar	100.0	100.0	98.9	79.2	91.7	99.9	91.8	96.3	91.7	70.8
Jind	100.0	100.0	98.0	74.7	91.4	99.9	89.9	96.0	85.1	20.8
Kaithal	98.0	97.8	89.8	48.0	75.2	95.8	78.5	86.0	58.5	38.6
Karnal	99.8	99.8	98.1	70.0	86.1	99.5	84.7	94.4	88.3	68.4
Kurukshetra	100.0	100.0	99.8	89.8	95.1	100.0	97.0	99.1	97.1	66.3
Mahendragarh	99.6	99.6	86.3	47.6	75.9	99.1	76.4	83.0	63.0	18.3
Panchkula	100.0	100.0	98.0	79.6	92.6	100.0	90.0	96.2	91.7	29.6
Panipat	99.8	99.8	97.7	74.6	95.8	99.6	90.2	97.1	80.8	74.1
Rewari	99.9	99.9	96.9	64.4	75.4	99.5	88.5	93.8	86.9	43.7
Rohtak	100.0	100.0	99.0	76.7	88.4	99.9	93.4	95.6	90.1	72.4
Sirsa	97.0	96.9	73.0	29.7	50.3	92.4	59.2	67.9	52.8	11.2
Sonipat	99.3	98.8	90.1	44.5	59.9	97.0	76.3	83.8	69.6	22.8
Yamunanagar	99.8	99.8	96.6	65.0	83.5	99.1	86.3	95.6	79.5	28.5
Haryana	99.6	99.5	94.5	67.3	84.0	98.9	85.5	91.4	80.9	46.7

Note: [1] Includes female sterilization, male sterilization, IUD, pills and condom. [2] Includes IUD pills and condom. [3] Includes female sterilization & male sterilization & IUD & pills and condom

6.1.1 Knowledge of Family Planning Methods by District

Table 6.2 shows the knowledge of contraceptive methods by districts in Haryana. In all districts more than 97 percent of women know about any modern methods of contraception. A large differential is noticed in the knowledge of all modern methods by districts. The awareness ranges from 30 percent women in Sirsa to 90 percent in Kurukshetra district. There is not much variation in the knowledge of female sterilization, which is the lowest in Sirsa (92 percent) and the highest in Ambala, Bhiwani, Hisar, Kurukshetra and Panipat districts (100 percent). Knowledge about IUD/Loop and condom are 59 and 53 percent respectively in Sirsa district, whereas the same is around 97 percent for each method in Ambala and Kurukshetra districts. As for any traditional method, awareness is 74 percent in Panipat and the least in Sirsa (11 percent).

6.1.2 Knowledge of No-Scalpel Vasectomy (NSV)

Knowledge of no-scalpel vasectomy among the husbands of currently married women in the state of Haryana is shown in Table 6.3. One-half of the husbands (50 percent) know about the no-scalpel vasectomy. In rural areas, 47 percent of husbands know about NSV compared to 58 percent in urban areas. For women residing in villages with a health facility, 47 percent of their husbands are aware of No-scalpel vasectomy and it is the same, that is, 47 percent for those living in villages without health facilities. Among the husbands who know about NSV, 80 percent reported that NSV is simpler than a conventional family planning method, 59 percent feel that reported as NSV does not lead to any complication and 53 percent reported that NSV does not affect a man’s sexual performance. Only 53 percent of the husbands in villages with a health facility reported that, NSV does not affect sexual performance compared to 51 percent of husbands in villages without a health facility.

Table 6.3 KNOWLEDGE OF NO-SCALPEL VASECTOMY (NSV)

Husbands knowledge of NSV by residence and availability of health facility in the village, Haryana, 2002-04

Knowledge of NSV	Total	Residence		Availability of health facility in the village[1]	
		Rural	Urban	No	Yes
Percentage of husband who had knowledge about NSV	50.3	47.2	58.0	47.1	47.3
Number of husbands	13,200	9,387	3,813	3,330	6,057
Who know that NSV is simpler than conventional vasectomy	80.4	79.8	81.6	79.7	79.9
Who feel that NSV does not lead to any complication	59.0	58.4	60.3	57.3	59.0
Who feel that NSV does not affect man’s sexual performance	52.5	52.4	52.9	50.9	53.2
Number of husbands	6,643	4,433	2,210	1,569	2,864

Note: [1] Includes sub-centre, primary health centre, community health centre or referral hospital, government hospital, and government dispensary within the village.

6.1.3 Knowledge of No-Scalpel Vasectomy (NSV) by District

No-scalpel vasectomy awareness by districts in Haryana is provided in Table 6.4. The districts in which at least half of the husbands know about NSV are Ambala (58 percent), Bhiwani (50 percent), Faridabad (58 percent), Jind (55 percent) Karnal (52 percent), Kurukshetra (51 percent), Mahendragarh (60 percent), Panchkula (56 percent), Rewari (59 percent) and Yamunanagar (54 percent). Only 41 percent of the husbands in Panipat district know about the no-scalpel vasectomy. That NSV does not lead to any complications was reported by 68 percent of the husbands in Kurukshetra districts and only 44 percent in Ambala. The husbands who reported that the NSV does not affect a man's sexual performance were highest 65 percent in Kurukshetra and the lowest in Ambala (34 percent).

Table 6.4 NO-SCALPEL VASECTOMY BY DISTRICT

Percentage of husband of eligible women by knowledge of NSV by district, Haryana, 2002-04

Districts	Knowledge about NSV	NSV is simpler than conventional method	Who reported NSV does not lead to any complication	Who reported NSV does not affect man's sexual performance
Ambala	58.4	88.4	44.4	33.5
Bhiwani	50.1	76.2	57.6	52.7
Faridabad	58.4	81.3	58.3	54.9
Fatehabad	48.2	82.8	65.1	60.5
Gurgaon	44.8	72.2	49.4	46.1
Hisar	41.7	90.2	54.8	50.0
Jhajjar	48.5	81.2	65.2	50.8
Jind	54.5	75.2	65.2	53.0
Kaithal	45.5	79.2	61.7	55.0
Karnal	51.6	82.1	66.1	50.8
Kurukshetra	51.3	92.9	67.8	65.1
Mahendragarh	59.9	76.7	62.4	60.8
Panchkula	55.9	76.3	57.1	54.9
Panipat	41.3	68.6	53.1	45.6
Rewari	59.0	77.2	56.1	51.1
Rohtak	46.7	86.6	66.8	58.1
Sirsa	41.8	82.0	66.9	59.8
Sonipat	48.6	73.9	50.6	45.7
Yamunanagar	53.5	82.9	59.7	55.7
Haryana	50.3	80.4	59.0	52.5

6.2 Current use of Family Planning Methods

Table 6.5 and Figure 6.2 provide the information on current use of family planning methods for currently married women in Haryana. At the time of DLHS-RCH, 60 percent of currently married women were using some method of contraception, 2 percentage points up from Round I. Current contraceptive use is slightly higher in urban areas (64 percent) than in rural areas (59 percent). Use of modern method is reported by 54 percent of the women, the breakdown of which is 37 percent for permanent methods and 18 percent for spacing methods. Among the users of sterilization methods most prefer female sterilization, which invalidates the use of male sterilization (less than one percent).

Table 6.5 CONTRACEPTIVE PREVALENCE RATE

Percentage of currently married women age 15-44 years currently using any contraceptive method by selected background characteristics, Haryana, 2002-04

Method	Any method	Any modern[1] method	Any modern spacing method[2]	Any sterilization	Male sterilization	Female sterilization	IUD/ Loop	Pill	Condom / Nirodh	Any traditional method[3]	Rhythm/ periodic abstinence	Withdrawal	Number of women
**Residence**													
Rural	58.8	54.0	12.9	40.9	0.9	40.0	3.6	2.5	6.9	4.8	2.9	1.7	13,306
Urban	64.0	55.2	28.9	25.8	1.1	24.7	6.7	4.5	17.7	8.8	4.8	3.6	5,489
**Education**													
Non-literate	62.4	57.9	7.8	50.0	1.1	48.9	2.0	2.0	3.8	4.5	2.7	1.4	8,388
0-9@ years	57.2	51.0	17.6	33.1	1.0	32.1	4.2	3.6	9.9	6.2	3.6	2.4	5,794
10 years & above	60.5	52.1	35.4	16.1	0.6	15.5	9.4	4.4	21.7	8.3	4.5	3.6	4,604
**Religion**													
Hindu	62.0	56.1	17.4	38.5	1.0	37.5	4.4	3.0	9.9	5.9	3.5	2.2	16,871
Muslim	24.4	20.0	10.9	8.9	0.5	8.4	1.8	2.2	6.9	4.4	2.7	1.6	935
Sikh	65.0	57.4	27.3	29.7	1.2	28.5	8.4	4.1	14.7	7.5	3.5	3.8	922
Other	70.2	59.8	36.9	22.1	0.0	22.1	9.8	4.3	22.8	10.4	4.2	2.6	67
**Caste/tribe#**													
Scheduled caste	54.4	48.9	12.0	36.8	0.9	35.9	1.9	2.5	7.6	5.6	3.7	1.5	4,084
Scheduled tribe	50.4	46.6	13.5	33.0	0.5	32.5	2.8	1.9	8.9	3.8	2.0	1.8	104
Other backward class	57.4	52.2	14.2	37.7	0.9	36.8	3.1	2.9	8.2	5.3	3.0	2.0	5,839
Other	65.1	58.5	22.5	35.6	1.0	34.6	6.6	3.4	12.4	6.6	3.7	2.8	8,764
**Standard of living index**													
Low	49.4	45.7	5.8	39.8	0.7	39.1	1.5	1.5	2.9	3.7	2.6	0.9	3,099
Medium	59.2	53.9	12.2	41.5	0.8	40.7	3.1	2.5	6.6	5.3	3.3	1.9	8,229
High	66.1	58.5	28.4	29.7	1.3	28.4	7.3	4.3	16.8	7.6	4.0	3.2	7,467
Availability of health facility in the village[4]													
No	57.8	53.2	12.9	40.1	0.8	39.2	3.4	2.9	6.6	4.6	2.9	1.6	4,719
Yes	59.3	54.5	12.9	41.4	0.9	40.5	3.7	2.3	7.0	4.8	2.9	1.7	8,587
Total	60.3	54.4	17.6	36.5	0.9	35.6	4.5	3.1	10.0	6.0	3.5	2.3	18,795

Note:[1] Include Female sterilization, Male sterilization, IUD, Pills and Condom. [2] Include IUD, Pills and Condom. [3] Include Rhythm/Periodic abstinence, Withdrawal and Other traditional method. @ Literate women with no years of schooling are also included. #Total figure may not add to N due to don't know and missing cases. [4] Includes sub-centre, primary health centre, community health centre or referral hospital, government hospital, and government dispensary within the village.

The use of traditional methods is reported by 6 percent of the women of whom 2 percent are using withdrawal and 4 percent follow the rhythm or periodic abstinence practice. The rural-urban differential is visible in the case of traditional methods, where 9 percent of the urban women are using this means of contraception compared to 5 percent of the rural women.

Figure 6.2
Practice of Family Planning Methods

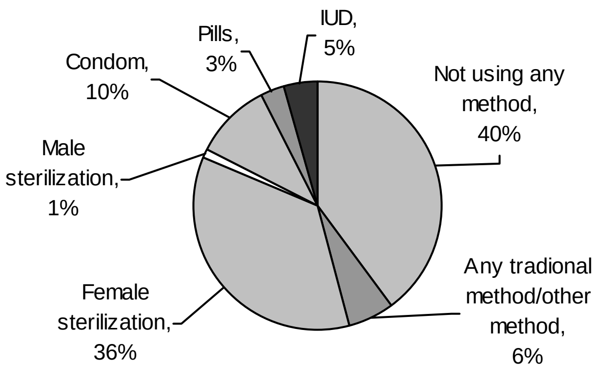

Note: Total percent may add more than 100.0 due to rounding

Haryana, DLHS-RCH, 2002-04

Current use of contraception is high among women of other castes (65 percent) than among Scheduled tribe women (50 percent). It is surprising that current use of contraception is high among non-literate women (62 percent) than the literate women who have less than 10 years of schooling (57 percent) and the women who have 10 or more years of schooling (61 percent). Similarly, current contraceptive use varies positively with respect to the standard of living of the women, increasing the prevalence rate from 49 percent to 66 percent for women from the lowest to the highest standard of living households. The availability of the health facility in the village is not so an important factor in motivating eligible women to use contraceptives. Fifty nine percent of the women living in villages with a health facility are currently under contraception and this is higher than the women from villages deprived of a health facility (58 percent). The current use of the traditional method is also higher among women with a higher education level and with a high standard of living than their counterparts not on par with these categories of women.

6.2.1 Current Use of Family Planning Methods by District

Table 6.6 presents a picture of current contraceptive use in the districts of Haryana. The contraceptive use is a couple concepts as family planning methods can be used either by women or by their husbands. In all the districts, the current use of contraception exceeds 42 percent of eligible women (see Map-6). The state figure of current spacing methods use is 18 percent and it ranges from 10 percent in Mahendragarh district to 25 percent in Kurukshetra. The variation in contraceptive prevalence at district level is basically due to the variation in the use of spacing methods while modern contraceptive uses do not show much variation across districts.

Table 6.6 CONTRACEPTIVE PREVALENCE RATES BY DISTRICT

Percentage of currently married women age 15-44 years currently using any contraceptive method by district, Haryana, 2002-04

Districts	Any method	Any modern[1] method	Any modern spacing[2] method	Male sterilization	Female sterilization	IUD	Pill	Condom/ Nirodh	Any traditional[3] method
Ambala	70.8	58.6	24.3	1.2	33.0	4.6	2.9	16.8	12.1
Bhiwani	60.3	56.6	11.5	1.4	43.2	3.1	2.1	6.4	3.6
Faridabad	52.7	47.9	19.0	0.7	27.8	5.9	3.7	9.4	4.7
Fatehabad	67.3	58.1	22.0	0.3	35.7	4.9	3.2	13.9	9.2
Gurgaon	41.6	36.4	11.6	0.8	23.6	2.9	2.3	6.3	5.2
Hisar	63.8	57.0	12.2	0.2	44.5	4.1	1.3	6.8	6.8
Jhajjar	64.3	57.2	17.7	0.4	38.6	5.4	2.4	10.0	7.2
Jind	57.8	55.4	15.2	0.7	39.2	6.9	2.8	5.4	2.4
Kaithal	59.4	58.2	17.6	1.0	39.4	5.4	4.2	8.0	1.2
Karnal	60.5	53.5	21.8	1.8	29.5	2.2	4.0	15.6	7.0
Kurukshetra	68.4	59.1	24.7	1.5	32.8	5.9	3.8	15.0	9.3
Mahendragarh	62.4	57.8	10.0	1.6	46.0	1.3	2.9	5.7	4.6
Panchkula	69.9	65.3	23.4	1.4	40.4	4.2	5.1	14.1	4.6
Panipat	54.0	45.3	19.9	0.4	24.7	5.9	3.5	10.6	8.7
Rewari	69.1	61.4	15.7	1.2	44.4	2.7	2.8	10.2	7.6
Rohtak	63.2	53.7	20.7	0.7	31.9	7.3	2.3	11.2	9.5
Sirsa	63.3	60.6	16.1	0.7	43.4	2.7	2.4	11.0	2.6
Sonipat	62.1	57.0	23.9	1.1	31.8	7.3	2.6	14.0	5.0
Yamunanagar	62.1	55.3	18.1	1.0	36.1	2.3	4.9	10.9	6.8
Haryana	60.3	54.4	17.6	0.9	35.6	4.5	3.1	10.0	6.0

Note: [1]Include Female sterilization, Male sterilization, IUD, Pills and Condom. [2] Include IUD, Pills and Condom. [3]Include Rhythm/ Periodic abstinence, Withdrawal and Other traditional method.

The pattern of use of contraceptive methods in Haryana is different from the general existing pattern in India. The contraceptive prevalence rate for traditional methods is 6 percent in the state. The use of oral Pills is only one percent in the district of Hisar and 5 percent in Panchkula and Yamunanagar .The district in which the use of condom is highest is Ambala (17 percent).

6.2.2 Current Use and Ever Use of Family Planning Methods by Women

Table 6.7 provides information on current contraceptive use and ever use of contraception by age and number of surviving children, living sons and daughters. Not using any method of contraception among currently married women in the 35-39 years age group is 18 percent and this attains a peak of 91 percent in the age group 15-19 years. A different age pattern of contraceptive use is also observed both in case of modern and traditional methods. The use of traditional method is 8 percent for the women aged 40 – 44 and 7 percent in 35-39 years and 6 percent for the women aged 30-34 and 25-29 years and it is least (3 percent) for the women in younger age groups 15-19 years. The use of modern methods ranges from 6 percent for women in the age group 15-19 years to 75 percent for women in the age group 35-39 years.

Table 6.7 USE OF CONTRACEPTION BY WOMEN

Percentage of currently married women in 15-44 years by current use and ever use of contraception according to selected demographic characteristics, Haryana, 2002-04

Demographic Characteristic	Percentage of women/husbands using				Percentage of women/ husbands by contraceptive status		Number of women
	Any modern method[1]	Any traditional method[2]	Any method	Not using any method	Ever used	Never used	
**Age-group**							
15-19	6.2	3.0	9.2	90.8	12.8	87.2	1,609
20-24	28.6	4.9	33.5	66.5	39.7	60.3	4,158
25-29	60.0	6.2	66.2	33.8	72.8	27.2	3,977
30-34	72.1	6.0	78.2	21.8	81.6	18.3	3,568
35-39	75.2	6.7	81.9	18.1	85.0	15.0	2,973
40-44	69.0	8.2	77.3	22.7	82.1	17.9	2,510
**Surviving children**							
0	2.9	1.1	4.0	96.0	7.2	92.8	2,217
1	26.6	6.9	33.5	66.5	41.9	58.1	2,824
2	63.5	7.4	70.9	29.1	76.4	23.6	5,392
3 or more	71.5	6.0	77.5	22.5	81.2	18.8	8,361
**Surviving sons**							
0	12.6	3.6	16.2	83.8	23.2	76.8	4,296
1	53.9	8.9	62.8	37.2	68.4	31.5	6,777
2 or more	78.0	4.7	82.6	17.4	85.7	14.3	7,721
**Surviving**							
**daughters**	41.5	4.1	45.6	54.4	49.8	50.2	6,653
0	62.8	6.5	69.3	30.7	74.5	25.5	6,590
1	59.8	7.5	67.3	32.7	72.5	27.5	5,552
2 or more							
	54.4	6.0	60.3	39.7	65.2	34.8	18,795
**All women**							

Note: [1] Include Female sterilization, Male sterilization, IUD, Pills and Condom. [2] Include Rhythm/Periodic abstinence, withdrawal and Other traditional method.

It is crucial to understand the association between the number of living children and contraceptive use. The contraceptive use is high among the women who have three or more surviving children invariably of methods in Haryana. The use of any method of contraception is 83 percent for the women who have two or more sons and is higher than the women who have two or more daughters (67 percent). The same trend can be observed in the case of use of any modern method which is 78 percent for the women who have two or more surviving sons and it is higher than the women who have two or more daughters (60 percent).

6.2.3 Current Use and Ever Use of Family Planning Methods as Reported by Husbands

Information pertaining to current use of family planning methods among the husbands of currently married women in Haryana by age and number of surviving children, sons and daughters are given in Table 6.8**.** The current use of any method of contraception among the husbands (aged below 25 years) of currently married women is 22 percent and it gradually picks up with the age of husband, to a peak of 80 percent in the age group 35-44 years. Similar age patterns of contraceptive use are observed in the case of modern methods. Among the husbands in the age group, 45 years and above the use of traditional methods is 7 percent and it is least 5 percent among the husbands in the younger age group of below 25 years and in the age group 25-

34 years. The use of modern methods ranges from 17 percent for husbands below 25 years of age to 73 percent for the husbands in the age group 35-44 years.

Table 6.8 USE OF CONTRACEPTION BY MEN

Percentage of husband of currently married women by current use and ever use of contraception by selected demographic variables, Haryana, 2002-04.

Demographic Characteristics	Percentage of husbands/women using				Number of men
	Any modern method[1]	Any traditionalmethod[2]	Any method	Not using any method	
**Age-group**					
<25	17.2	4.7	21.9	78.1	1,556
25-34	49.9	5.0	54.9	45.1	5,034
35-44	73.2	6.3	79.6	20.4	4,632
45+	71.0	6.9	77.9	22.1	1,978
**Surviving children**					
0	3.9	1.2	5.1	94.9	1,443
1	29.6	7.7	37.3	62.7	1,867
2	65.5	6.9	72.4	27.6	3,818
3 or more	73.6	5.4	79.0	21.0	6,073
**Surviving sons**					
0	14.1	4.0	18.0	82.0	2,843
1	57.7	8.1	65.8	34.2	4,740
2 or more	79.1	4.6	83.6	16.4	5,617
**Surviving daughters**					
0	44.6	4.4	48.9	51.0	4,523
1	65.2	6.3	71.5	28.5	4,729
2 or more	62.7	6.5	69.3	30.7	3,949
All men	57.4	5.7	63.1	36.9	13,200

Note: [1] Include Female sterilization, Male sterilization, IUD, Pills and Condom. [2] Include Rhythm/Periodic abstinence, Withdrawal and Other traditional method.

6.3 Reasons for Not Using Male Methods

The DLHS-RCH asked husbands of currently married women about the contraceptive methods that he or his wife was using currently. The husbands who were not using male methods were further asked the reasons for it. Table 6.9 provides information about reasons for not using male contraceptive methods in Haryana. Among all the husbands interviewed, 70 percent reported about female methods. Reporting of female methods is higher in rural areas (77 percent) than in urban areas (55 percent). The reasons cited for not preferring the male methods are greater popularity of female methods (56 percent), fear of weakness (42 percent), fear of operation (4 percent), fear of method failure (3 percent) and lack of sexual pleasure (2 percent). Less than one percent reported fear of impotency as one of the reasons for not using male methods. However, there is not much rural-urban differential in the reasons for not using male methods, except in the case of fear of weakness. The expression for fear of weakness is higher in rural areas (44 percent) than in urban areas (33 percent). Popularity of female methods as a reason for not using male methods of contraception is more in urban areas (60 percent) than in rural areas (55 percent).

Table 6.9 REASONS FOR NOT USING MALE METHODS

Percentage of husbands with their choice of family planning methods and reasons for not accepting male methods according to residence, Haryana, 2002-04

Female method users and reason for not accepting male methods	Total	Residence	
		Rural	Urban
Percentage of husband who have reported female methods	70.2	76.6	55.4
Number of men	8,330	5,803	2,527
Reasons for not accepting male methods*			
Fear of impotency	0.7	0.7	0.7
Lack of sexual pleasure	1.7	1.6	2.0
Fear of method failure	2.5	2.3	3.2
Fear of operation	3.8	3.8	3.7
Fear of weakness	41.5	44.3	32.8
Female methods are more popular	55.8	54.7	59.5
Other	8.7	8.4	9.8
Number of men	5,848	4,448	1,400

Note: * Percentages may add to more than 100.0 because multiple responses could be recorded.

6.4 Source of Contraceptive Methods

To asses the various sources of contraceptive methods, DLHS-RCH collected information on source of obtaining methods. Table 6.10 and Figure 6.3 show the percent distribution of current users of modern contraceptives by source of contraceptives. Family planning methods and services in Haryana are provided primarily through a network of government hospitals. The services are also provided by private hospitals and clinics, as well as non-governmental organisations (NGOs). Modern spacing methods like IUD, Pill and condom are available through both the government and private sectors. Government/municipal hospitals are the main source for female sterilization (69 percent) followed by community health centres or primary health centres (12 percent), family planning camps or RCH camp (8 percent) and private hospital (10 percent). Among the IUD users, 36 percent reported the source as private hospital, 32 percent reported the source as government/municipal hospital and 10 percent from the community health centres. It is found that the chemist is the main source for Pills (64 percent) and condom (82 percent).

TABLE 6.10 SOURCE OF MODERN CONTRACEPTIVE METHODS

Percent distribution of current users of modern contraceptive methods by method and source of supply, Haryana, 2002-04

Source	Contraceptive method					All modern methods[1]
	Female sterilization	Male sterilization	IUD/ Loop	Pills	Condom/ Nirodh	
**Government medical centre**	89.0	93.9	50.8	22.9	10.2	67.5
Government/Municipal hospital	68.5	80.0	31.7	6.1	3.2	50.0
CHC/PHC	11.7	8.7	10.3	4.6	1.9	9.3
Sub-centre	0.2	0.0	6.2	8.7	3.2	1.7
Government doctor	0.2	0.6	0.6	0.4	0.0	0.2
Government nurse/ ANM	0.0	0.0	1.2	2.4	1.0	0.4
Family planning/RCH camp	8.4	4.5	0.4	0.2	0.0	5.6
Out reach/MCP clinic in village	0.0	0.0	0.1	0.3	0.2	0.1
Mobile clinic	0.0	0.0	0.3	0.2	0.6	0.2
**Private medical centre**	10.5	0.8	47.7	5.6	2.2	11.6
Private hospital	9.6	0.8	35.7	2.2	0.9	9.6
Private doctor	0.7	0.0	8.7	2.2	1.0	1.5
Private nurse	0.1	0.0	3.3	1.2	0.3	0.5
Chemist	NA	NA	NA	64.4	81.9	18.8
Other	0.4	2.8	1.2	5.6	2.3	1.2
Do not know	0.1	2.3	0.2	1.5	3.4	0.8
Missing	0.0	0.2	0.0	0.0	0.0	0.0
Total percent	100.0	100.0	100.0	100.0	100.0	100.0
Number of users	6,683	177	845	575	1,886	10,166

Note: [1] Includes female sterilization, male sterilization, IUD, Pills or condom. CHC: Community health centre, PHC: Primary health centre. NA: Not applicable.

Figure 6.3
Source of Family Planning Among Current Users of Modern Contraceptive Methods

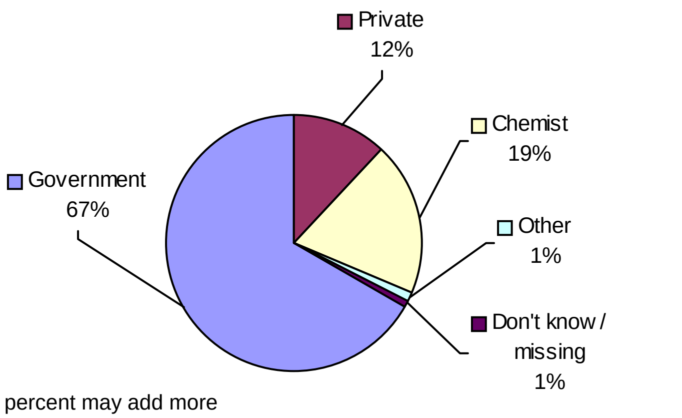

Note: Total percent may add more than 100.0 due to rounding

Haryana, DLHS-RCH, 2002-04

6.5 Problems with Current Use of Contraceptive Methods

Women who were using a modern contraceptive method were asked if they had experienced any problems related with the current methods they are using. Table 6.11 shows the percentage of current contraceptive users who reported specific health problems, treatment seeking behaviour and their satisfaction about the method. The analysis of the method specific problems reveals that 11 percent of the sterilized women have problem with the contraceptive methods in use. The most common problems experienced by sterilized women are body ache or backache (47 percent), weakness or inability to work (28 percent), white discharge (22 percent), excessive bleeding (19 percent), dizziness (17 percent), irregular periods (14 percent) and cramps (12 percent). With regard to the modern spacing methods, 8 and 9 percent of women had problems in using Pills and IUD respectively. The most common problems of Pill users were dizziness and irregular periods (31 percent each), weakness or inability to work and cramps (29 percent each), white discharge and body ache or backache (24 percent each), nausea or vomiting (12 percent).

Table 6.11 HEALTH PROBLEMS WITH CURRENT USE OF CONTRACEPTION

Percentage of women informed about side effects, had side effects with the method by use of method, Haryana, 2002-04

Health problems/side effect	Type of method		
	Female sterilizations	IUD/loop	Pill
Women who were informed about all the available methods	21.6	0.0	0.0
Women who were informed about the side effects before adoption of the method	34.9	39.2	27.3
Women who had side effect/health problem due to use of contraceptive method	10.9	9.1	8.4
Number of current users	6,683	845	575
**Type of health problems/side effects[1]**			
Weakness/inability to work	28.1	13.6	28.6
Body ache/ backache	46.6	30.3	23.8
Cramps	12.3	5.5	28.6
Weight gain	11.3	10.1	4.8
Dizziness	17.1	13.1	31.0
Nausea/vomiting	6.4	1.3	11.9
Breast tenderness	3.2	0.7	0.0
Irregular periods	14.1	23.5	31.0
Excessive bleeding	18.5	39.5	14.3
Spotting	3.3	0.3	0.0
White discharge	21.6	21.5	23.8
Other	39.6	47.1	57.1
Number of users with side effects	728	77	48

Note: [1] Percentages may add to more than 100.0 because multiple problems could be recorded.
( ) Based on less than 50 unweighted cases.

6.6 Treatment for Health Problems with Current Use of Contraception

The study of respondents who sought treatment for contraceptive related health problems reveals that 64 percent of the sterilized women sought treatment and 36 percent in the case of Pills. Regarding the satisfaction about the method, 96 percent of the sterilized women reported satisfaction with sterilization. In the case of spacing methods, 96 percent of women using Pills and 96 percent of women using IUD were satisfied with the respective methods.

Those women who had sought treatment for contraceptive use related problems, majority of them have taken treatment from private hospitals/clinics in case of IUD. For female sterilization related health problems, 56 percent had taken treatment from government hospitals/dispensaries, 37 percent from private hospitals/clinics and 7 percent from Indian System of Medicine health facilities. Private hospital/clinic is the source of treatment for 50 percent of women who had health problem in using IUD.

Table 6.12 FOLLOW-UP VISIT AND SOUGHT TREATMENT FOR HEALTH PROBLEMS WITH CURRENT USE OF CONTRACEPTION

Percentage of women who had follow-up visit, satisfied with current method and sought treatment with side effect with the method by use of method, Haryana, 2002-04

Health problems/side effect	Type of method		
	Female sterilizations	IUD/loop	Pill
Women who had follow up visit by health worker after adoption of method	41.7	9.3	7.4
Women who are satisfied with method of current use	95.9	96.3	95.7
Number of current users	6,683	845	575
Women who sought treatment for the health problem	64.0	57.6	(35.7)
Number of women with side effects	728	77	48
**Source of treatments**			
**Government health facility**			
Government hospital/dispensary	41.5	31.2	*
UHC/UHP/UFWC	1.4	2.0	*
CHC/Rural hospital	3.5	4.8	*
PHC	6.6	4.3	*
Sub-centre	2.4	3.3	*
Out reach/MCP clinic in village	0.3	2.3	*
**Private health facility**			
NGO/trust hospital clinic	0.3	1.5	*
Private hospital/clinic	36.5	48.3	*
ISM health facility[1]	6.7	2.0	*
Chemist/Medical shop	6.7	4.2	*
Home remedy	1.4	0.0	*
Other	2.4	0.0	*
Number of women with side effects	466	44	21

Note: [1]Either government or Private. () Based on les than 50 unweighted cases. *Percentage not shown: Based on very few cases.

6.7 Advice to Non-Users and their Future Intention to Use Contraception

Information about non-users who were advised by the ANM/Health worker to adopt contraceptives and their future intention to use by preferred method according to their background characteristics are presented in Table 6.13. In DLHS-RCH currently married women who were not using any method of contraception, were asked about advice given by ANM/Health worker for adoption of any contraceptive method. It is evident that only 6 percent of the women were advised by ANM/Health worker to adopt any family planning method in Haryana. Among rural women, 7 percent were advised by ANM/Health worker to adopt any method and it is higher than the urban women (6 percent) who were advised so.

Table 6.13 ADVICE ON CONTRACEPTIVE USE

Percentage of current non-users* who were advised by the ANM/health worker to use contraception by suggested method according to place of residence and availability of health facility in the village, Haryana, 2002-04

Advise/future intension to use	Total	Residence		Availability of health facility in the village[1]	
		Rural	Urban	No	Yes
Percentage of current non-users advised by ANM/health worker to use of contraceptive method	6.4	6.5	5.9	6.5	6.5
Number of non-users	7,159	5,303	1,856	1,920	3,383
Percent distribution of women who were advised by method					
Female sterilization	53.2	56.6	42.5	62.7	53.2
Male sterilization	3.4	3.8	2.4	3.6	3.9
IUD/loop	16.0	15.9	16.4	12.8	17.7
Pill	15.4	14.3	18.9	11.4	16.0
Condom/Nirodh	10.9	8.3	19.1	8.3	8.3
Rhythmic /periodic abstinence	0.4	0.5	0.0	0.6	0.4
Withdrawal	0.3	0.3	0.0	0.0	0.5
Other	0.4	0.3	0.7	0.8	0.0
Total percent	100.0	100.0	100.0	100.0	100.0
Number of non-users	456	346	110	125	221

Note: * Exclude women in menopause or those who have undergone hysterectomy.
[1]Includes sub-centre, primary health centre, community health centre or referral hospital, government hospital, and government dispensary within the village.

The recommended contraceptive methods by ANM/health worker are dominated by female sterilization (53 percent) and IUD/loop (16 percent). Only (15 percent) were advised to adopt Pill and Condom/Nirodh (11 percent) as spacing methods. Male sterilization has been advised to 3 percent. This pattern of advice also emerges irrespective of residence and availability of health facility in the village.

6.7.1 Future Intentions

Among the non-users, 33 percent of women have expressed their intention to use any method of contraception in the future. The intention to use any method of contraception is higher in rural areas (34 percent) than in urban areas (31 percent).

Among the women who intended to use permanent methods of contraception, 70 percent preferred female sterilization whereas less than one percent of the women preferred male sterilization. In case of temporary methods, the preferred methods by women are IUD (7 percent), oral pills (8 percent) followed by condom (6 percent), withdrawal method preferred by less than one percent, rhythm/periodic abstinence method preferred by one percent.

More than two-fifth (43 percent) of the husbands intended to use contraception in the future, among them 44 percent belong to rural areas and 42 percent from urban areas. Method wise choice in intention to use contraception is dominated female sterilization being reported by 72 percent, followed by condom (13 percent).

Table 6.14 FUTURE INTENTION TO USE

Percentage of current non-users* who were intended to use contraception in future by preferred method according to place of residence, Haryana, 2002-04

Future intention to use/method	Women			Husband		
	Total	Rural	Urban	Total	Rural	Urban
Percentage of respondents who intend to use contraceptive in future	33.0	33.8	30.7	43.2	43.6	41.9
Number of non-users	7,159	5,303	1,856	4,763	3,523	1,240
Percent distribution of non-user who were preferred to use family methods by preferred method						
Female sterilization	69.6	71.9	62.1	71.8	72.7	69.0
Male sterilization	0.7	0.8	0.7	5.3	5.8	3.7
IUD/copper-T/ Loop	6.6	5.4	10.1	3.7	3.1	5.5
Oral pills	8.3	9.6	4.3	3.1	3.7	1.3
Condom/Nirodh	5.7	4.0	11.2	12.8	11.3	17.1
Rhythm/periodic abstinence	0.9	0.9	1.1	0.3	0.3	0.3
Withdrawal	0.3	0.0	1.3	0.3	0.2	0.4
Other	7.1	6.8	7.8	2.6	2.7	2.4
Missing	0.8	0.6	1.4	0.2	0.1	0.3
Total percent	100.0	100.0	100.0	100.0	100.0	100.0
Number of non-users	2,358	1,789	569	2,049	1,530	519

Note: * Exclude women who are in menopause or those who have undergone hysterectomy.

6.7.2 Future Intention to Use Among Women by Number of Living Children

Currently married women who were not using any contraceptive method at the time of survey were asked about their intentions to use a method in the future. Those women who intended to use contraceptives in the future were further asked about preferred methods. This type of information aids the managers and programmers to identify the potential groups of future users and to provide the type of contraceptives that are likely to be in demand. Table 6.15 provides the information on intention to use contraception in future according to number of living children and residence background in Haryana. Among the current non-users, around 12 percent of the women intended to use contraception within the next twelve months. Only 6 percent of women wanted to use within one to two years whereas 14 percent reported their intention to use contraceptives after two years. About 43 percent are not sure of their intention to use, whereas 23 percent reported no intention to use. The intention of using contraception is high among the women who have two or more living children compared to the women who have either one or no living children. Sixty percent of the women who have no living children reported that they are yet to decide about the use of contraceptives.

Table 6.15 FUTURE USE OF CONTRACEPTION BY NUMBER OF LIVING CHILDREN

Percent distribution of currently married women* who were not currently using any contraceptive method by intention to use in the future, according to number of living children and residence, Haryana, 2002-04

Intention to use in the future	Number of living children					Total
	0	1	2	3	4+	
			**Total**			
Intends to use in next 12 months	2.8	9.9	17.7	20.1	23.0	12.2
One to two years	2.4	8.0	8.0	10.9	5.7	6.4
More than two years	14.9	18.7	15.1	10.1	6.6	14.3
Does not intend to use	19.7	17.8	23.2	28.4	39.6	23.4
Not yet decided	60.1	45.6	36.0	30.5	25.0	43.6
Total percent	100.0	100.0	100.0	100.0	100.0	100.0
Number of women	2,110	1,858	1,492	783	915	7,159
			**Rural**			
Intends to use in next 12 months	3.3	10.1	17.3	19.8	22.3	12.4
One to two years	2.3	8.1	8.9	11.7	6.0	6.7
More than two years	15.2	19.3	15.5	11.6	6.9	14.7
Does not intend to use	20.5	17.8	21.1	26.4	41.6	23.6
Not yet decided	58.8	44.7	37.1	30.5	23.1	42.6
Total percent	100.0	100.0	100.0	100.0	100.0	100.0
Number of women	1,539	1,324	1,107	574	758	5,303
			**Urban**			
Intends to use in next 12 months	1.5	9.7	19.1	20.8	26.4	11.8
One to two years	2.9	7.6	5.4	8.5	4.4	5.5
More than two years	14.3	17.2	13.8	5.8	5.2	13.3
Does not intend to use	17.8	17.7	29.0	34.2	29.9	23.0
Not yet decided	63.4	47.8	32.6	30.7	34.0	46.4
Missing	0.1	0.0	0.0	0.0	0.0	0.0
Total percent	100.0	100.0	100.0	100.0	100.0	100.0
Number of women	572	534	385	208	156	1,856

Note: * Exclude women who are in menopause or those who have undergone hysterectomy.

6.8 Reasons for Discontinuation and Non-Use of Contraception

Currently married non-pregnant women who were not using any contraceptive method at the time of survey were categorised as past users and never users according to their contraceptive experience. In DLHS-RCH, women who had discontinued contraceptive use were asked about the main reason for discontinuation. The survey also asked women who had never used contraceptives about the main reason for not doing so. Table 6.16 shows the main reason for not using contraceptives among both the past never users and current non-users. Among the past users, around 52 percent of the women mentioned that they discontinued the use because they had wanted child, method failed/became pregnant (8 percent) weakness/inability to work (6 percent), irregular periods, excessive bleeding and method was inconvenient (3 percent each) and other reasons (16 percent). For urban women 8 percent have reported method failure/become pregnant due to discontinuation. In urban areas, 21 percent of women reported as other reason for discontinuing the use and where as the same is 13 percent among rural women.

Table 6.16 REASONS FOR DISCONTINUATION OF CONTRACEPTION

Percent distribution of women who were past users (current non-users) by reason for discontinuation of the contraceptive method according to place of residence, Haryana, 2002-04

Reasons	Total	Place of residence	
		Rural	Urban
**Reason for discontinuation**			
Wanted child	51.6	52.6	49.9
Method failed/became pregnant	8.1	8.1	8.0
Supply not available	0.7	0.9	0.4
Difficult to get method	0.8	1.0	0.6
Weakness/inability to work	5.7	6.8	3.7
Body ache/ Backache	2.9	2.6	3.4
Cramps	0.0	0.0	0.0
Weight gain	0.3	0.1	0.8
Dizziness	1.5	1.8	0.9
Nausea/vomiting	0.9	1.3	0.2
Breast tenderness	0.4	0.5	0.3
Irregular periods	3.3	2.8	4.1
Excessive bleeding	3.1	2.9	3.5
Spotting	0.2	0.1	0.2
White discharge	1.0	1.4	0.2
Lack of pleasure	0.4	0.7	0.0
Method was inconvenient	2.9	3.1	2.5
Other	16.0	12.8	21.3
Missing	0.3	0.4	0.1
Total percent	100.0	100.0	100.0
Number of past users	913	577	337

6.8.1 Reasons for Not Using Contraceptive Methods

DLHS asked women and husbands who are currently not using any contraception and main reasons why they were not currently using a method. The reported main reasons for not using contraceptives are health does not permit (14 percent), opposed to family planning (12 percent), difficult to become pregnant (8 percent), against the religion (6 percent), lack of knowledge about family planning methods and afraid of sterilization (2 percent each). About 47 percent of the women reported other reasons for not using contraception. As far as rural-urban differentials are concerned, a little variation is observed in the reasons for not using any contraceptive.

Table 6.17 REASON FOR NOT USING CONTRACEPTIVE METHOD						
Percentage of current non-users who were currently not using contraceptive method by reason according to place of residence, Haryana, 2002-04						
Reason	Women			Husband*		
	Total	Rural	Urban	Total	Rural	Urban
Lack of Knowledge about FP method	2.0	2.5	0.9	2.3	3.0	0.4
Against the Religion	6.0	8.0	1.0	5.2	6.1	2.7
Opposed to family planning	11.7	12.0	10.7	6.9	7.0	6.7
Not like existing method	2.7	2.1	4.3	5.7	5.4	6.5
Afraid of sterilization	2.3	2.7	1.4	1.2	1.4	0.7
Can not work after sterilization	0.6	0.5	0.9	1.0	0.6	2.2
Worry about side effects	1.3	1.5	0.7	2.0	2.6	0.6
Costs too much	1.0	0.5	2.2	2.1	2.0	2.6
Health does not permit	13.8	12.9	16.2	25.2	23.2	30.4
Hard/inconvenient to get method	1.1	1.3	0.6	1.4	1.6	0.9
Inconvenient to use method	2.0	2.2	1.4	1.7	1.5	2.0
Difficult to become pregnant	7.9	7.5	8.8	8.2	8.1	8.6
Wife is pregnant[1]	-	-	-	0.3	0.4	0.0
Other	47.0	45.7	50.5	36.6	37.0	35.4
Missing	0.5	0.6	0.3	0.3	0.3	0.4
Total percent	100.0	100.0	100.0	100.0	100.0	100.0
Number of current non-users	2,771	2,004	767	1,466	1,068	398

Note: [1] Not applicable for women. * Excluding not decided cases on timing of next child.

6.9 Unmet Need for Family Planning Services

Unmet need for family planning is one of the indicators to assess the effectiveness of the family planning programme. Policy makers and family planning programme planners use this to know the demand for family planning services/supplies. Unmet need is defined in this report separately for limiting and spacing. Unmet need for spacing includes the proportion of currently married women who are neither in menopause nor had hysterectomy nor are currently pregnant and who want more children after two years or later and are currently not using any family planning method. The women who are not sure about whether and when to have next child, are also included in unmet need for spacing. The women who are not sure about the timing of the next child are also included in the unmet need for spacing. Unmet need for limiting includes the proportion of currently married women who are neither in menopause nor had hysterectomy nor are currently pregnant and do not want any more children but are currently not using any family planning method. Total unmet need refers to the totality of unmet for limiting and spacing. Table 6.18 provides the information about unmet need for limiting and spacing in Haryana by background characteristics.

The unmet need is high for women aged 20-24 years, mainly for spacing rather than for limiting. Unmet need is also relatively high for women below 20 years (18 percent). Among the older women of age 25-29 years, 14 percent have unmet need, and mostly for limiting. Among the women age 30 years and above, unmet need is mostly for limiting. The rural women have high unmet need (15 percent) than the urban women (14 percent). The unmet need for family planning is somehow equal among the non-literate women (16 percent), women with 0-9 years of schooling (14 percent) and women with 10 or more years of schooling (13 percent). Sikh and other women have lesser unmet need for family planning (13 percent) compared to the Muslim women (36 percent). Unmet need for family planning is equal for Scheduled tribe and Scheduled

caste (17 percent each), followed by other backward class (16 percent) and other caste (13 percent) women.

Table 6.18 UNMET NEED FOR FAMILY PLANNING SERVICES

Percentage of currently married women with unmet need for family planning services by selected background characteristics, Haryana, 2002-04

Background Characteristic	Unmet need for FP			Number of women
	Spacing[1]	Limiting[2]	Total	
**Age**				
15-19	15.8	2.6	18.4	1,609
20-24	11.7	8.6	20.2	4,158
25-29	4.2	10.1	14.3	3,977
30-34	2.2	9.3	11.5	3,568
35-39	1.3	10.0	11.3	2,973
40-44	0.3	12.0	12.3	2,510
**Residence**				
Rural	5.8	9.2	15.0	13,306
Urban	4.7	9.3	13.9	5,489
**Education**				
Illiterate	5.2	11.0	16.2	8,388
0-9 @ years	5.7	8.0	13.7	5,794
10 years and above	5.7	7.5	13.1	4,604
**Religion**				
Hindu	5.0	8.7	13.6	16,871
Muslim	15.4	20.3	35.8	935
Sikh	4.7	8.2	12.8	922
Others	7.8	5.0	12.8	67
**Caste/tribe#**				
Scheduled caste	6.0	10.7	16.6	4,084
Scheduled tribe	2.7	14.0	16.7	104
Other backward class	6.7	9.6	16.3	5,839
Others	4.4	8.2	12.6	8,764
**Number of living children**				
0	4.8	0.4	5.2	2,217
1	16.9	4.2	21.1	2,824
2	4.2	11.5	15.8	5,392
3	2.3	9.3	11.5	4,438
4+	2.9	14.6	17.5	3,923
**Standard of living Index**				
Low	7.9	13.0	20.9	3,099
Medium	5.5	9.0	14.5	8,229
High	4.5	7.9	12.3	7,467
All women	5.5	9.2	14.7	18,795

Note: [1]Unmet need for spacing includes the proportion of currently married women who are neither in menopause or had hysterectomy nor are currently pregnant and who want more children after two years or later and are currently not using any family planning method. The women who are not sure about whether and when to have next child are also included in unmet need for spacing. [2]Unmet need for limiting includes the proportion of currently married women who are neither in menopause or had hysterectomy nor are currently pregnant and do not want any more children but are currently not using any family planning method.

Total unmet need refers to unmet for limiting and spacing.

@ Literate women with no years of schooling are also included. # The total figure may not add to N due to do not know and missing cases.

Women in low standard of living have high (21 percent) unmet need than the women of medium (15 percent) and high standard of living (13 percent). Unmet need is much higher for the women with one living child (21 percent) than women with no children (5 percent). Among the women with no children or one child the unmet need is mainly for spacing, whereas for women with two children or more unmet need is exclusively for limiting.

6.9.1 Unmet Need for Family Planning Services by Districts

Table 6.19 provides the information about unmet need for limiting, spacing and total by district. The unmet need for family planning services for state is 15 percent and it ranges from 6 percent in Ambala to 27 percent in Gurgaon. In 7 out of 19 districts unmet need for family planning is more than state average. Unmet need for limiting was found lowest in Ambala (4 percent) and highest in Gurgaon (14 percent). Similarly, unmet need for spacing was lowest to two percent in Ambala, Kurukshetra and Yamunanagar to 13 percent in Gurgaon. It may also observe that in all the districts of Haryana unmet need for limiting was more than spacing.

Table 6.19 UNMET NEED BY DISTRICT

Percentage of currently married women with unmet need by district, Haryana, 2002-04

Districts	Unmet need for		
	Spacing	Limiting	Total
Ambala	2.2	4.2	6.4
Bhiwani	5.0	8.3	13.3
Faridabad	9.0	12.2	21.2
Fatehabad	3.9	5.4	9.3
Gurgaon	12.3	14.3	26.5
Hisar	3.3	5.3	8.6
Jhajjar	4.6	6.2	10.8
Jind	2.9	9.5	12.4
Kaithal	8.1	8.8	16.9
Karnal	5.3	9.9	15.2
Kurukshetra	1.8	7.7	9.6
Mahendragarh	5.2	8.7	14.0
Panchkula	4.4	6.2	10.6
Panipat	7.4	12.0	19.4
Rewari	3.2	8.1	11.4
Rohtak	5.4	6.7	12.2
Sirsa	5.3	10.8	16.1
Sonipat	5.4	11.6	17.1
Yamunanagar	2.2	9.8	12.1
Haryana	5.5	9.2	14.7

MAP-6
Current Use of Any Family Planning Method

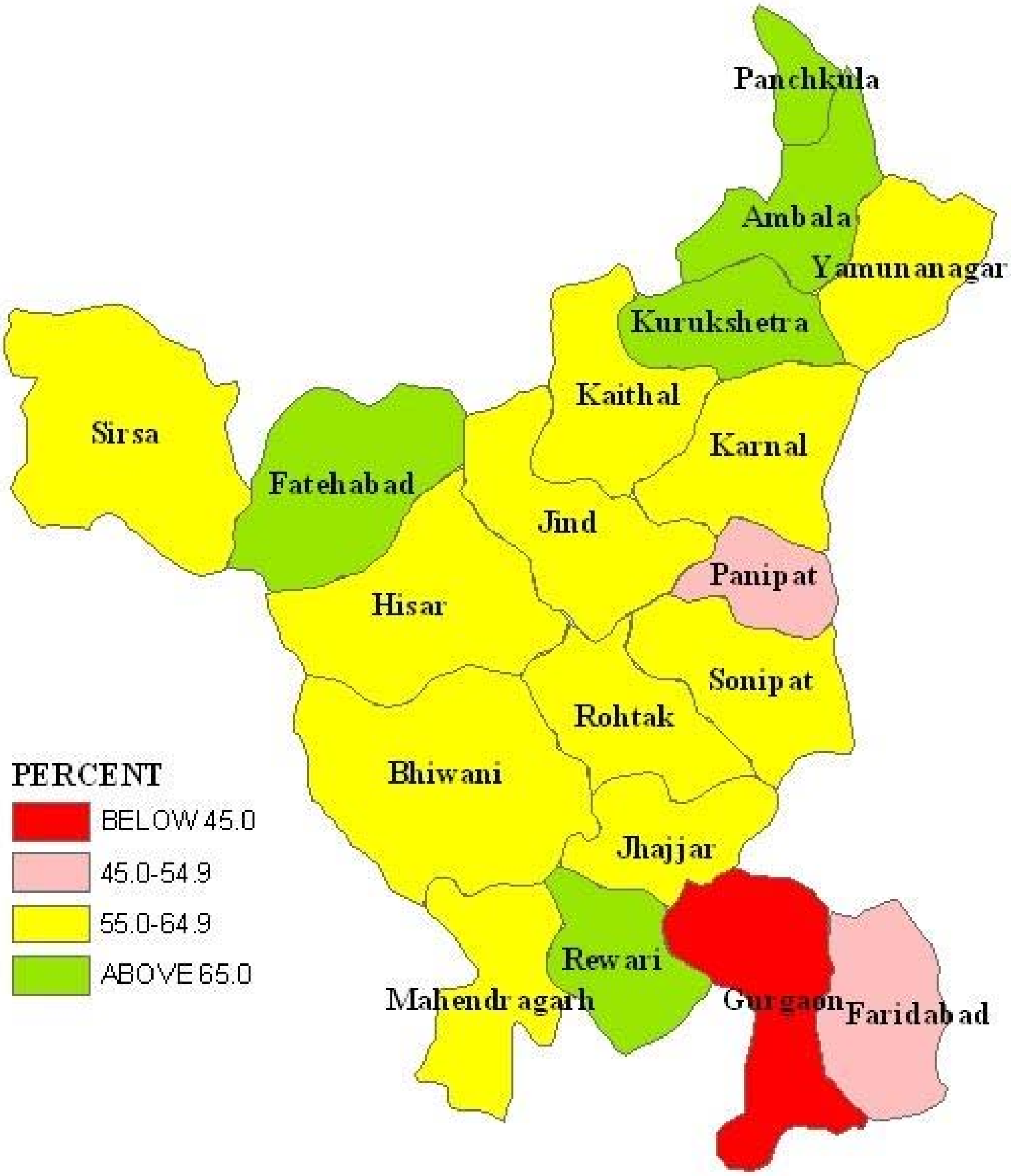

CHAPTER VII

ACCESSIBILITY AND PERCEPTION ABOUT GOVERNMENT HEALTH FACILITIES

The government health facilities at all the levels provide various RCH services. Auxiliary Nurse Midwife (ANM), family planning worker or male health worker play a key role in delivering the services to the community. Health workers are expected to make regular visits to all the households in their assigned area. During these contacts, the health workers are supposed to monitor various aspects of the health of women and children, provide information related to health and family planning, counsel and motivate to adopt appropriate health and family planning practices and deliver other selected services. These contacts are also important as they enhance the creditability of services and establish necessary rapport with the clients. In order to assess the extent of utilisation of government health facilities by all eligible women and to find out whether ANM/health workers reach the households for providing RCH services, a separate section in the women's questionnaire was canvassed to all the eligible women. This chapter deals with the accessibility and the opinion of women about the services provided by the government health workers. The quality of care offered by the government health programme as perceived by currently married women is also presented.

7.1 Home Visit by Health Workers

Table 7.1 shows the percentage of currently married women visited by health workers at home during the three months prior to the survey. Three percent of the women in Haryana reported that the health worker visited them at their residence at least once in last three months preceding the survey. Younger women seemed more likely to report a home visit than older women. Four percent of women in the age group 15-24 years reported at least one home visit compared to only 2 percent of women in the age group 35 years and older. The percentage of women in Haryana receiving home visits is higher in rural areas (4 percent) than in urban areas (2 percent). More Muslim and Sikh and Other women (4 percent each) reported home visits than Hindu (3 percent). More Scheduled tribe women (9 percent) reported home visits than Scheduled caste (3 percent).

Women who reported a home visit during the three months preceding the survey were asked who visited their household during the past three months and whether they were satisfied with the kind of services/advice received, and the time spent by these health workers. Among women who received services at home, 80 percent received services from ANM/LHV, 14 percent from the doctor and 11 percent from male health worker. There were rural-urban differentials by visit of households by health worker. Eighty one percent of women who received services at home were satisfied with the time spent with them and 92 percent of women were satisfied with the services or advice given to them.

Table 7.1 HOME VISIT BY HEALTH WORKER

Percentage of women who had home visit by a doctor, ANM/LHV, or male health worker in the 3 months preceding the survey, among women who had home visit, satisfied with time spent by health workers and with services provided by selected background characteristics, Haryana, 2002-04

Background characteristic	Percentage with home visit	Number of women	Home visit by[1]			Percentage of women satisfied with		Number of women
			Doctor	ANM / LHV	Male health worker	Amount of time	Services/ advices	
**Age**								
15.24	4.0	5,767	10.3	81.0	13.3	78.4	91.5	230
25-34	3.2	7,545	14.5	80.7	10.3	84.4	93.3	239
35-44	2.0	5,483	20.5	76.2	9.4	78.6	87.7	108
**Residence**								
Rural	3.6	13,306	13.0	82.7	9.3	80.9	92.1	485
Urban	1.7	5,489	18.8	65.1	22.2	80.9	88.3	92
**Education**								
Non-literate	2.5	8,388	13.2	82.0	7.8	77.9	89.7	213
0-9@ years	3.4	5,794	13.5	78.2	12.7	81.5	92.9	199
10 and above	3.6	4,604	15.3	79.3	14.2	84.2	92.2	165
**Religion**								
Hindu	3.0	16,871	13.8	80.2	10.9	82.0	91.5	501
Muslim	3.6	935	(12.9)	(80.6)	(16.1)	(71.0)	(87.1)	34
Sikh	4.3	922	(12.8)	(80.9)	(8.5)	(80.9)	(95.7)	39
Other	4.3	67	*	*	*	*	*	3
**Caste/tribe#**								
Scheduled caste	2.7	4,084	13.7	80.8	11.0	84.9	96.6	111
Scheduled tribe	8.6	104	*	*	*	*	*	9
Other backward class	3.5	5,839	12.4	79.4	11.9	80.4	91.4	202
Other	2.9	8,764	15.5	80.4	10.7	79.3	89.0	254
**Standard of living index**								
Low	3.1	3,099	12.5	79.6	13.2	79.5	91.4	95
Medium	3.1	8,229	12.4	82.2	10.4	82.9	94.8	255
High	3.0	7,467	16.2	77.6	11.6	79.3	87.8	227
Availability of health facility[2] in the village								
Yes	4.1	8,574	13.2	82.7	9.9	81.9	91.1	350
No	2.8	4,732	12.5	82.9	7.7	78.4	94.6	135
Total	3.1	18,795	13.9	79.9	11.3	80.9	91.5	577

Note: Total includes 9 and 4 women with missing information on education and do not know were not shown separately.
[1] Percentage add to more than 100.0 due to multiple responses. @ Literate mother with no years of schooling are included.
Total number may not add to N due to do not know and missing cases.
[2] Includes sub-center, primary health center, Community health center or referral hospital, government hospital, and government dispensary within the village. ( ) : Based on les than 50 unweighted cases. *Percentage not shown: Based on very few cases.

The proportion of women who were satisfied with the amount of time spent and advices provided by health workers varied across various background characteristics. Eighty four percent of women in the age group 25-34 years reported satisfaction with the time spent by health workers as compared to 79 percent of women aged 35 years and older. Urban and rural women (81 percent) were equally satisfied with the time spent by health workers during home visits. Women residing in the village with availability of health facility are slightly more satisfied with the time spent than women from those villages where health facilities are not available.

7.2 Home Visit by Health Workers by District

In 7 out of 19 districts in Haryana, health workers visited 2 or less than 2 percent of the women at home (Table 7.2 and Figure 7.1). In districts like Karnal and Panchkula 7-9 percent of the women were visited by health workers. Among women who were visited by health worker at home, about 48 percent of them approached by ANM/LHV in Rohtak and 98 percent of the women in Sirsa. Approached by male worker at home is lowest in Mahendragarh, Panchkula, Sirsa and Yamunanagar (nil), followed by Gurgaon (4 percent) district and highest in Jhajjar (33 percent) district and the percentage of women visited by doctor at home was below the state average of 14 percent in 12 of the 19 districts.

In the districts of Hisar, Kurukshetra and Yamunanagar all the women said that the worker had spent enough time with them. On the other hand, more than three-fourth of the women in all the districts and all the women in Ambala, Fatehabad, Hisar, Jind, Kurukshetra and Mahendragarh reported satisfaction with services/advices given by health workers.

Figure 7.1
Distribution of Districts by Home Visit by Health Worker

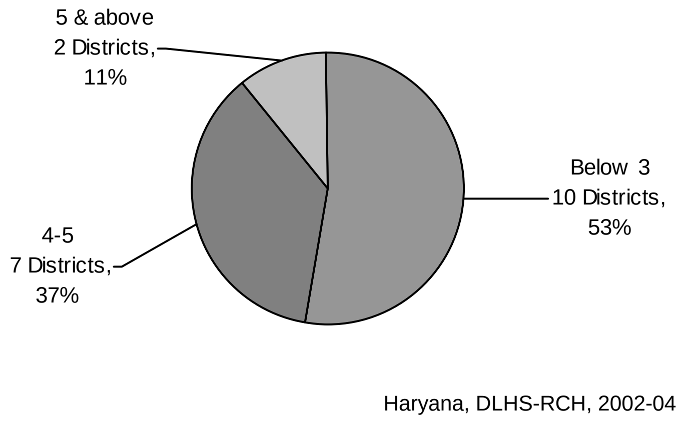

Table 7.2 HOME VISIT BY HEALTH WORKER BY DISTRICT

Percentage of women who had home visit by a doctor, ANM/LHV, or male health worker in the 3 months preceding the survey, among women who had home visit, satisfied with time spent by health workers and with services provided by district, Haryana, 2002-04

District	Percentage with home visit	Home visit by[1]			Percentage of women satisfied with	
		Doctor	ANM / LHV	Male health worker	Time spent	Service
Ambala	2.4	(38.1)	(57.8)	(9.2)	(96.0)	(100.0)
Bhiwani	1.3	(9.0)	(82.2)	(8.8)	(84.6)	(85.4)
Faridabad	4.1	3.5	94.3	4.5	90.6	90.5
Fatehabad	4.0	11.5	70.2	23.1	73.5	100.0
Gurgaon	2.9	6.5	90.0	3.5	74.6	95.7
Hisar	1.4	(0.0)	(90.5)	(9.5)	(100.0)	(100.0)
Jhajjar	2.5	4.4	74.9	32.5	63.4	92.1
Jind	2.4	(10.5)	(82.2)	(7.3)	(96.4)	(100.0)
Kaithal	2.9	19.8	73.1	13.9	76.9	71.2
Karnal	6.9	24.8	72.5	16.2	75.3	93.9
Kurukshetra	1.9	(0.0)	(95.6)	(4.4)	(100.0)	(100.0)
Mahendragarh	1.5	(31.0)	(69.0)	(0.0)	(90.2)	(100.0)
Panchkula	8.5	3.4	96.6	0.0	97.4	97.4
Panipat	3.4	14.1	78.0	10.9	80.5	90.6
Rewari	3.5	8.0	79.7	23.6	70.1	95.3
Rohtak	4.0	44.3	48.1	20.9	72.4	95.4
Sirsa	4.4	2.3	97.7	0.0	83.0	81.2
Sonipat	3.2	23.3	66.6	19.8	47.4	71.7
Yamunanagar	0.7	(12.9)	(87.1)	(0.0)	(100.0)	(85.0)
Haryana	3.2	14.0	79.9	11.3	81.5	92.2

Note: [1] Percentage adds to more than 100.0 due to multiple responses. () Based on less number of cases.

7.3 Matters Discussed during Home visits or Visits to Health Facilities

Women who were visited at home by a family planning worker, as well as those who visited government health facility or other health facility during the three months preceding the survey were asked about the different topics discussed with the workers during any of these visits. Table 7.3 shows the percentage of women who discussed the health and family planning or any health related matters to the health workers during home visits or visits to a health facility during the past three months. There are 341 pregnant woman or women with children born during the reference period and other women includes 195 current users and 41 current non-users, who were visited by health workers at home.

The major focus of discussion during home visits was immunization (35 percent) and family planning (28 percent). In addition, discussions were also made on antenatal care (11 percent), delivery care (4 percent) and postpartum care (7 percent) and child care (13 percent) as well as about treatment of other health problems (7 percent). Discussions about family planning were mentioned more often by current users of contraception and by pregnant women or women with child born after reference period than current non- users. As expected, pregnant women or women with child born after reference period were much more likely than other women to report that they discussed childcare, immunization, antenatal care, postpartum care, and breastfeeding. A higher proportion of current contraceptive users and current non-

users discussed disease prevention, treatment of health problems and other health related matters during home visit by health workers past three months preceding the survey.

Table 7.3 MATTER DISCUSSED DURING CONTACT WITH A HEALTH WORKER

Percentage of women who were visited by health worker in the three months preceding the survey, and percentage of women who visited health facility, and the percentage of women[1] who discussed specific topics with the health worker, Haryana, 2002-04

Topic discussed	Pregnant women or women with children after reference period[2]	Other women		Total
		Current contraceptive users	Current nonusers	
**During home visit**				
Family planning	27.8	28.6	25.2	27.9
Breastfeeding	(4.3)	(1.4)	(0.0)	(3.2)
Supplementary feeding	(7.7)	(4.2)	(2.3)	(6.6)
Immunization	43.4	21.8	22.6	34.6
Nutrition	(3.7)	(3.7)	(2.3)	(6.1)
Diseases prevention	8.2	36.5	20.5	18.7
Treatment of health problem	7.8	20.7	24.8	13.3
Antenatal care	15.3	4.4	0.0	10.5
Delivery care	(6.6)	(0.5)	(0.0)	(4.3)
Postpartum care	(6.6)	(1.4)	(0.0)	(7.0)
Childcare	12.6	17.7	6.4	13.9
Sanitation / cleanliness	(4.6)	(11.1)	(7.0)	(8.3)
Oral rehydration	(1.7)	(1.9)	(4.7)	(20)
Other	6.5	12.5	10.5	8.8
Number of women	341	195	41	577
**During visit to health facility**				
Family planning	13.0	14.1	6.5	12.7
Breastfeeding	(0.6)	(0.2)	(0.0)	(0.4)
Supplementary feeding	(1.3)	(0.2)	(1.1)	(0.9)
Immunization	33.6	1.1	.6	21.2
Nutrition	2.7	5.7	6.8	4.0
Diseases prevention	13.1	42.7	35.9	23.6
Treatment of health problem	20.3	45.7	42.4	29.6
Antenatal care	25.5	0.6	0.9	16.1
Delivery care	4.7	0.1	0.8	3.0
Postpartum care	(2.3)	(0.9)	(0.0)	(1.7)
Childcare	7.0	7.0	5.2	6.8
Sanitation / cleanliness	(1.6)	(1.4)	(1.6)	(1.5)
Oral rehydration	(0.2)	(0.3)	(0.0)	(0.2)
Other	2.8	4.8	15.7	4.6
Number of women	1,139	514	179	1,832

Note: Percentage add to more than 100.0 due to multiple responses.
[1] Women who visited private health facility are not included. [2] Reference period for phase I, January 1st 1999 and for phase II, January 1st 2001
(): Based on les than 50 unweighted cases

The topics discussed most often during visits to health facility by women were treatment of health problems (30 percent), diseases prevention (24 percent), immunization (21 percent) and antenatal care (16 percent). Only 13 percent women reported that they discussed family planning during the visit. A few pregnant women or women with children born after reference period also discussed about delivery care, postpartum care, breastfeeding, nutrition, oral re-hydration and breastfeeding during visit to health facility. A higher proportion of current users and non-users discussed on treatment of health problems, disease prevention, and other health related problems than pregnant women with children after reference period during visit to health facility in three months prior to survey.

7.4 Visit to Health Facility

Table 7.4 presents the percentage of currently married women who needed to visit health facility and visited the health facility by residence and availability of health facility in the village. Around 39 percent of women needed to visit health facility but did not visit in comparison with 31 percent of women who needed to visit health facility and visited in past three months of the survey. The proportion of such women was higher in urban areas (35 percent) than in rural areas (30 percent). Among them who visited any health facility, two-third of the women reported that they had visited a private hospital/ dispensary, (61 percent in rural areas and 74 percent in urban areas).

Table 7.4 VISIT TO HEALTH FACILITY

Percentage of women who need to visit health facility and visited, and percent distribution of women visited health facility by type of health facility and according to place of residence and availability of health facilities in the village, Haryana, 2002-04

Health facility	Total	Residence		Availability of health facility[1] in the village	
		Rural	Urban	No	Yes
Percentage of women who needed to visit health facility and not visited	38.9	39.9	36.5	41.5	38.9
Percentage of women who needed to visit health facility and visited	31.1	29.5	34.8	27.7	30.5
Number of women	18,795	13,306	5,489	4,732	8,574
**Government health facility**					
Hospital / CHC / FRU /RH	16.5	15.3	18.9	16.3	14.8
Dispensary	1.6	1.8	1.0	1.6	1.9
Primary health center	4.5	6.1	1.1	3.3	7.6
Sub-center	5.8	8.1	1.0	5.7	9.2
**Private health facility**					
Hospital	50.1	44.8	60.9	47.7	43.3
Dispensary	15.4	16.5	13.1	16.0	16.7
ISM[2] hospital/dispensary	3.5	3.7	2.9	3.9	3.7
Other	2.8	3.7	1.1	5.5	2.8
Total percent	100.0	100.0	100.0	100.0	100.0
Number of women	5,841	3,932	1,909	1,313	2,619

Note: CHC: Community health center, FRU: First referral unit, RH: Referral Hospital

[1] Includes sub-center, primary health center, Community health center or referral hospital, government hospital, and government dispensary within the village. [2] Either government or private health facility of Indian System of Medicine.

Only twenty eight percent of the women visited a government health facility, of which 17 percent visited hospital/CHC/FRU/RH, 6 percent visited sub-centres, 5 percent visited to primary health centre and only 2 percent visited government dispensary. Only four percent of the women reported that they visited Indian system of medicine hospital/ dispensary either government or private. There are not many differences in visit to any health facility according to availability of health facility in the village in the past three months of the survey.

7.5 Visit to Health Facility by District

Table 7.5 presents the percentage of currently married women who needed to visit health facility and visited the health facility by districts. Seventeen percent of currently married women in Ambala and Fatehabad and 59 percent in Bhiwani needed to visit a health facility, but they did not visit. Out of 19, in 3 districts i.e. Ambala, Karnal and Kurukshetra, more than half of the women visited health facility for their health problems In Sirsa only 14 percent of women visited health facility when needed. Among them who visited health facility, less than a quarter women visited government health facility in three districts and in all districts more than half of the women visited private health facility in past three months of the survey.

Table 7.5 VISIT TO HEALTH FACILITY BY DISTRICT

Percentage of women who needed to visit health facility, but not visited and percentage of women who visited health facility by type of health facility by district, Haryana, 2002-04

Districts	Percentage of women who need to visit health facility, but not visited	Percentage of women who need to visit health facility and visited	Percentage of women who visited to	
			Government health facility	Private health facility
Ambala	17.3	52.3	24.9	75.1
Bhiwani	59.0	27.0	27.3	71.0
Faridabad	57.6	27.2	22.7	74.4
Fatehabad	17.3	27.6	44.3	53.9
Gurgaon	48.9	25.8	26.1	70.4
Hisar	33.8	41.9	27.4	72.6
Jhajjar	30.3	37.9	28.4	71.1
Jind	41.3	34.8	28.2	61.3
Kaithal	42.3	23.6	30.6	68.6
Karnal	24.6	50.1	18.2	75.1
Kurukshetra	27.5	55.1	20.7	79.1
Mahendragarh	55.5	15.9	45.3	50.2
Panchkula	44.4	44.2	43.9	49.1
Panipat	44.5	24.0	26.0	74.0
Rewari	28.5	18.9	33.7	62.8
Rohtak	24.9	43.0	39.3	59.8
Sirsa	55.4	13.6	42.2	55.6
Sonipat	22.3	15.0	37.6	61.3
Yamunanagar	27.6	28.7	26.5	67.8
Haryana	38.9	31.1	28.6	68.6

7.6 Client's Perception of Quality of Government Health Services

Utilization of services is an essential indicator reflecting the quality of services. Better quality of services would have a higher utilization rate, which is very important from the policy point of view. Unless clients are satisfied with the services provided by the government, efforts made by the government will be wasted. In order to assess the utilization of government health facilities, a question was asked whether they had visited any health facility for their health problem during past three months to the survey. Those who visited the government health facility were asked their perceptions about quality of services, (personal manner like courtesy, respect, sensitivity, and friendliness of the physician and staff, technical

skills and quality like thoroughness, carefulness, and competence and waiting time for receiving the services) and same is presented in Table 7.6. Women in general perceived that the quality of services, personal manner as well technical skills and quality of physician, ANM/nurse and other staff were good. Majority of the respondents perceived that personal manner (courtesy, respect, sensitivity, and friendliness) and technical skills (thoroughness, carefulness, and competence) of the physician, nurses and other staff were good, a few respondents mentioned that personnel manner of doctor (7 percent), nurse and other staff including paramedical staff (5 percent each) were excellent.

Table 7.6 QUALITY OF GOVERNMENT HEALTH FACILITY

Percentage of women who visited government health facility and rated quality and availability of services during most recent visit to a government health facility in the three months proceeding the survey, Haryana, 2002-04

Quality indicator	Poor	Good	Excellent
The convenience of the health facility location	4.2	91.9	3.8
Length[1] of time spend towards waiting	11.4	77.9	10.6
Personal manner[2] of the physician[5]	2.3	91.1	6.6
The technical skills and quality[3] of the physician[5]	2.4	91.1	6.5
Personal manner[2] of nurse	3.0	92.0	5.0
The technical skills and quality[3] of nurse	3.1	91.5	5.5
Personal manner of other staff[5]	4.0	91.5	4.5
The technical skills and quality of other[4] staff	3.5	85.9	3.5
The explanation of what was done to her	3.1	91.1	5.8
Medical, surgical and diagnostic equipment	8.4	80.8	4.9
General comfort	5.8	87.8	6.4

Note: [1] Poor indicate long waiting time, good indicate average waiting time, and excellent indicate short waiting time [2]Courtesy, respect, sensitivity, friendliness. [3]Thoroughness, carefulness, competence. [4] Including paramedical staff. [5]Includes hospital/community health center/ first referral unit/ referral hospital, dispensary, and primary health center last visit made by women

7.7 Reason for not visiting Government Health Centre

Women who visited the private health centre were asked the main reason for not visiting the government health centre and the results are presented in Table 7.7. Twenty five percent of the currently married women reported poor quality of service as one of the reasons for not visiting the government health centre for their health problems; urban and rural women equally report this reason. About 17 percent reported that they did not feel necessity to visit the government health centre due to inconvenient location of the centre (17 percent in rural and 18 urban areas). Other reasons for not visiting government health centres were: time is not suited (13 percent), heavy rush (10 percent), doctor/ health workers do not examine properly (11 percent), medicine rarely/not given or of bad quality (14 percent), non-availability or rare availability of doctors/ health workers (3 percent).

Table 7.7 REASON FOR NOT PREFERRING GOVERNMENT HEALTH FACILITY

Percent distribution of women visited private health facility by reason for not visiting government health facility and according to residence and availability of health facilities in the village, Haryana, 2002-04

Reason	Total	Residence		Availability of health facility[1] in the village	
		Rural	Urban	No	Yes
Not conveniently located	16.9	16.5	17.5	20.0	14.6
Time is not suited	13.2	13.3	13.0	13.6	13.1
Poor quality of services	24.8	24.9	24.7	23.7	25.5
Heavy rush	10.4	8.4	13.9	8.2	8.5
Non/rare-availability of doctors/health workers	3.0	3.3	2.6	3.3	3.3
Doctors/health workers do not examine properly	10.7	10.9	10.5	11.0	10.8
Medicine not/rarely given or of bad quality	14.2	16.6	10.1	14.5	17.8
Doctors/paramedical staff does not behave properly	(0.6)	(0.6)	(0.7)	(0.4)	(0.7)
Services are charged	1.3	1.5	0.9	(1.3)	(1.5)
Referred by government doctor	(0.8)	(0.6)	(1.0)	(0.4)	(0.8)
Other	4.1	3.5	5.2	3.4	3.5
Total percent	100.0	100.0	100.0	100.0	100.0
Number of women	4,009	2,545	1,463	885	1,659

Note: [1] Includes sub-center, primary health center, Community health center or referral hospital, government hospital, and government dispensary within the village

( ) Based on les than 50 unweighted cases.

7.8 Family Planning Information and Advice Received

Women who are currently not using any contraceptive method were asked whether they were ever advised by ANM or family planning health worker to adopt family planning method advised during any of the contact. Six percent of currently non-users said that they had advices or discussion on method of family planning with ANM or family planning health worker (Table 7.8). The most frequently discussed method was female sterilization (53 percent), IUD (16 percent) and Pills (15 percent). 11 percent of women received advice to adopt condom as a contraceptive method. Discussions about traditional method, such as rhythm method advised to (10 percent) women, while below one percent of women advised for withdrawal method. There is no much variation by types of residence in terms of family planning information and advice received.

7.9 Availability of Pills and Condom

To explore difficulties faced in the procurement of condoms and pills, current users of these methods were asked that they had been able to get their supply whenever needed. The results are presented in Table 7.9. Only 3 percent of condom and pill users reported that they had a problem in getting these methods. Higher proportion of rural women (5 percent) than urban women (1 percent) had problems in getting a supply of these methods.

Table 7.8 ADVISE TO ADOPT FAMILY PLANNING METHOD

Percentage of current non-users who reported ever advised to adopt family planning method by method of family planning by ANM/health worker, according to residence, Haryana, 2002-04

Method	Total	Rural	Urban
Percentage of non-users who were advised to adopt family planning method	6.4	6.5	5.9
Number of women	7,159	5,303	1,856
**Method**			
Female sterilization	53.2	56.6	42.5
Male sterilization	3.4	3.8	2.4
IUD	16.0	15.9	16.4
Pills	15.4	14.3	18.9
Condom	10.9	8.3	19.1
Rhythm	10.3	0.5	0.0
Withdrawal	0.2	0.3	0.0
Other	0.4	0.4	0.7
Total percent	100.0	100.0	100.0
Number of women	456	346	110

Note: Total includes 5 cases missing on advice to adopt family planning method.

Table 7.9 AVAILABILITY OF REGULAR SUPPLY OF CONDOMS/PILLS

Percentage of current condom or pill users who ever had a problem getting a supply of condoms/pills by residence, Haryana, 2002-04

Method/residence	Percentage who had a problem getting supply	Number of users
**Condom**		
Rural	4.8	329
Urban	0.8	245
Total	3.1	575
**Pills**		
Rural	2.8	916
Urban	2.9	970
Total	2.9	1,886

7.10 Quality of Care of Family Planning Services

Several aspects regarding quality of care of family planning services were also investigated. Current user of a sterilization was asked whether the person or centre where sterilization had been performed, informed her about other alternative methods of family planning; and further it was asked whether she was told by a ANM or health worker about possible side effects of the modern method at the time she accepted the method; whether she received any follow-up care after accepting the method. Tables 7.10 and 7.11 present the results of this investigation.

Around 22 percent of sterilized women reported that ANM or health worker informed them about alternative methods that they could use (Table 7.10) before adopting sterilization. Around 22 percent of sterilized women received such information by an ANM or health worker in the government health facilities compared to around 23 percent of women who were sterilized in private health facilities and 16 percent of women received this information in the family planning or RCH camp or out reach/ MCH clinic in village at the time of accepting the sterilization.

Table 7.10 INFORMATION OF OTHER MODERN METHOD BEFORE STERILIZATION

Percentage of current users of sterilization who were informed about other modern method by the source where they get sterilized, according to the source of sterilization and residence, Haryana, 2002-04

Source of sterilization	Total	Rural	Urban	Number of users
Government health facility	22.0	21.8	23.1	5,543
Family planning or RCH camp/ village session	16.4	15.3	23.8	569
Private health facility	22.9	22.0	24.8	702
Other	(48.0)	(32.0)	(70.0)	34
Total	21.8	21.3	23.8	6,860

Note: Total includes 1 and 12 women who said that they sterilized at mobile clinic, and by chemist, and who do not know including missing information of place/source of sterilization, are not shown separately

( ) Based on less than 50 unweighted cases.

Table 7.11 INFORMATION ON SIDE EFFECT AND FOLLOW-UP FOR CURRENT METHOD

Percentage of current users of modern contraceptive methods who were told about side effects or other problems of current method by a health worker or ANM/Nurse at the time of accepting the method and percentage who received follow-up services after accepting the method by current method and residence, Haryana, 2002-04

Information/follow-up	Total	Rural	Urban
**Told about side effects**			
Sterilization	34.9	35.4	32.7
Other modern method	20.5	23.2	17.6
Any modern method	30.2	32.5	24.7
**Received follow-up**			
Sterilization	41.1	45.0	26.2
Other modern method	5.4	6.5	4.2
Any modern method	29.5	35.8	14.6

Another important fact of informed contraceptive choice is being fully informed about any side effects and any other problems associated with the method. In Haryana, only 30 percent of users of any modern method were informed about possible side effects or health problems associated with their current method. Thirty five percent of acceptors of sterilization in rural area and 33 percent in urban area reported that they were informed about side effects. Among users of modern method other than sterilization, 23 percent of rural users and 18 percent of urban users were informed about side effects. It is clear from the result that ANM or health workers in Haryana are not providing sufficient information to couples who need to make an informed choice about contraceptive methods. The situation with respect to follow-up services is also not encouraging. Follow-up services among sterilization users are slightly higher than user of modern methods. About forty five percent of sterilization users in rural

area and about 26 percent in urban area reported that they received follow-up services by ANM or health worker. Only 5 percent of the users of other modern method received follow-up services. In all, only 36 percent of the users of any modern method in rural area and only 15 percent in urban areas received follow-up services.

7.11 Quality of Care Indicators for Contraceptive Users by District

Table 7.12 shows inter-district variations in the percentage of users of sterilization who were told about alternative methods before adopting sterilization and about side effects or other problems related to the current method or users of modern contraceptive methods, and the percentage of users who received follow-up services.

Table 7.12 QUALITY OF CARE INDICATORS FOR CONTRACEPTIVE USERS BY DISTRICT

Among currently married women who are current users of modern contraceptive methods, quality of care indicators related to the use of their current contraceptive method by district, Haryana, 2002-04

District	Percentage informed about other methods before getting sterilization[1]	Percentage told about side effects or other problems with method[2]		Percentage who received follow –up[2]		Percentage nonuser told ever had advised to adopt contraceptive method
		Sterilization	Other modern method	Sterilization	Other modern method	
Ambala	31.6	80.5	33.4	62.5	4.5	5.8
Bhiwani	25.3	29.6	27.2	43.9	2.3	10.6
Faridabad	12.1	13.2	14.6	23.4	4.0	4.7
Fatehabad	31.6	58.2	21.0	40.5	5.0	5.7
Gurgaon	18.8	35.0	21.1	55.3	4.2	7.6
Hisar	27.5	85.4	39.2	44.0	3.8	3.7
Jhajjar	22.2	13.1	18.9	31.4	0.9	2.1
Jind	9.0	16.1	17.7	38.6	7.3	2.8
Kaithal	16.3	21.3	22.8	37.8	5.2	4.9
Karnal	20.2	14.0	13.5	35.9	8.1	7.6
Kurukshetra	39.9	74.7	23.3	57.6	7.4	8.3
Mahendragarh	9.6	10.9	2.5	20.5	0.0	5.7
Panchkula	9.4	18.0	21.0	48.4	8.4	9.8
Panipat	26.8	40.4	20.7	51.9	7.7	9.2
Rewari	41.5	37.9	23.3	55.2	3.7	10.3
Rohtak	13.2	17.9	17.2	37.1	11.3	4.0
Sirsa	8.1	22.4	13.4	39.1	6.4	5.7
Sonipat	29.7	20.7	19.2	24.9	2.3	9.3
Yamunanagar	30.6	46.1	16.6	56.2	5.6	7.0
Haryana	21.8	34.9	20.5	41.1	5.4	6.4

Note: [1] At the time of accepting the current method. [2] By a health worker or ANM/Nurse after accepting the current method.

The percentage of sterilization-users who were told about alternate method is lowest in Sirsa (8 percent) and it is highest in Rewari (42 percent). There are also large inter-district variations in the percentage of sterilization-users and users of modern contraceptive methods who were told about the possible side effect. In case of sterilization, the proportion varied from a low of 11 percent in Mahendragarh to a high of 85 percent in Hisar. For other modern contraceptive methods, about 39 percent in Hisar and a minimum of 3 percent users in Mahendragarh district were told about the side effects of the method. Follow-up services are

slightly better for acceptors of sterilization than for other modern methods in most of the districts of Haryana. Table 7.12 also shows district wise variation in the percentage of currently non-users who were ever advised to adopt contraceptive methods, which varies from a low 2 percent in Jhajjar to a high of 11 percent in Bhiwani.

Overall, the quality of care for family planning and health services is far from satisfactory in many of the district of Haryana; almost all districts need to work much more to improve their health and family planning services, particularly services that are provided by the government sector.

7.12 Quality of Care of Maternal Health Care

Information on few other aspects of quality of care in terms of maternal care was also collected. Women with last live/still births during three years preceding the survey were asked whether the Doctor/ANM/health worker advised you to go to health facility for delivery when they were pregnant, and received any follow-up care after delivering the baby within 2 weeks of delivery and received follow care at least one visit within six weeks of delivery. The same information is presented in Table 7.13.

Table 7.13 ADVISED TO HAVE DELIVERY AT HEALTH FACILITY AND FOLLOW-UP SERVICES FOR POSTPARTUM CHECK-UP

Percentage of women* who were advised to have delivery at health facility by doctor/ health worker and percentage who receive follow-up services within 2 weeks and within 6 weeks of delivery by ANM, according to residence, Haryana, 2002-04

Advise/follow-up service	Total	Rural	Urban
Percentage of women who were advised to have delivery at health facility	26.9	22.2	39.6
Percentage of women who were visited within 2 weeks of delivery	8.2	9.1	5.6
Percentage of women who were visited at least once within 6 weeks of delivery	8.6	9.7	5.8
Number of women	6,674	4,877	1,797

Note: * Women who had live birth/still birth after 1.1.1999/2001
Total includes 3 missing cases in Percentage of women who were advised to have delivery at health facility.

More than one-fourth of the women with last live/still births during three years preceding the survey reported that they were advised by doctor or health worker to have delivery in health facility. Women from urban areas (40 percent) were more likely than rural areas (22 percent) to get advised to deliver their child at health facility.

In district wise variation, the percentage varies from as low as 15 percent in Gurgaon to as high as 52 percent in Ambala (Table 7.14). In 13 of the 19 districts, less than one-third women were advised for deliver their child in health facility.

Table 7.14 QUALITY OF CARE INDICATORS FOR MATERNAL CARE

Among currently married women* who are given live/still birth three years preceding the survey, quality of care indicators related to delivery care by district, Haryana, 2002-04

District	Percentage of women		
	Advised to have delivery at health facility by doctor/ health worker	Visited within 2 weeks of delivery by ANM	Visited at least one within 6 weeks of delivery by ANM
Ambala	51.6	5.0	5.3
Bhiwani	21.9	3.5	3.7
Faridabad	21.7	6.9	6.9
Fatehabad	32.1	14.7	15.7
Gurgaon	14.9	5.5	5.5
Hisar	25.7	2.3	4.6
Jhajjar	36.1	6.7	6.8
Jind	26.6	6.8	6.8
Kaithal	19.8	11.7	11.7
Karnal	33.3	15.7	16.3
Kurukshetra	35.7	5.5	6.1
Mahendragarh	21.2	7.5	8.4
Panchkula	35.4	10.7	10.7
Panipat	26.7	11.5	11.5
Rewari	31.7	10.7	10.4
Rohtak	27.7	6.5	7.8
Sirsa	21.0	14.0	15.3
Sonipat	28.9	9.4	10.1
Yamunanagar	34.4	11.8	11.8
Haryana	26.9	8.2	8.6

Note: * Women who had live birth/still birth after 1.1.1999/2001

Eight percent of the women reported that they were visited by an ANM within two weeks of delivery; such visit was only 6 percent in urban areas and 9 percent in rural areas. Only 10 percent of the women in rural area and 6 percent in urban areas received at least one follow-up service within six weeks of delivery. Not more than one-sixth women were received postpartum check-up within 2 weeks of delivery in any district of Haryana and the proportion of women who had at least one postpartum check-up within six weeks of delivery varied from a low of 4 percent in Bhiwani to high of 16 percent each in Fatehabad and Karnal (Table 7.14).

CHAPTER IV

MATERNAL HEALTH CARE

Provisions of maternal health care services to ensure safe motherhood is one of the major components of the Reproductive and Child Health (RCH) programme. The RCH programme services for antenatal care, includes at least three antenatal care visits, iron prophylaxis for pregnant and lactating women, at least one dose of tetanus toxoid vaccine, detection and treatment of anaemia in mothers, and management and referral of high-risk pregnancies, natal care, that is encouragement of safe delivery, post-natal care, and management of unwanted pregnancies. In rural areas, the government delivers reproductive health and other health services through its network of Sub-Centres (SCs), Primary Health Centres (PHCs) and other health facilities. In addition, pregnant women and children can get services from private maternity homes, hospitals, private practitioners, and in some case non-governmental organisations (NGOs) and trust hospitals. In urban areas, reproductive health services are available mainly through government or municipal hospitals, Urban Health Posts (UHPs), Urban Family Welfare Centres (UFWCs), hospitals and nursing homes operated by NGOs, and private nursing and maternity homes.

The National Population Policy (NPP), 2000 adopted by the Government of India (Ministry of Health and Family Welfare, 2000) reiterates the Government's commitments to the safe motherhood programme within the wider context of reproductive health. Among the national socio-demographic goals for 2010 specified by the policy, several goals pertain to safe motherhood, that 80 percent of all deliveries should take place in institutions by 2010, hundred percent deliveries should be attended by trained personnel and the maternal mortality ratio should be reduced to a level below 100 per 100,000 live births. Empowering women for improved health and nutrition is one of the 12 strategic themes identified in the policy to be pursued either as stand-alone programmes or as intersectoral programmes.

In DLHS-RCH Phase-I, to all the eligible women who had their last pregnancy after January 1, 1999 a separate section on the status of maternal health and utilisation of maternal health care services was canvassed. In Phase-II, the same section was canvassed to all the eligible women who had their last pregnancy after January 1, 2001. The women whose last pregnancy terminated into live/still birth were asked about the details of antenatal, natal and post-natal care they received; pregnancy, delivery and post-delivery complications they suffered from and the treatment seeking behaviour in case of complications. Women whose last pregnancy terminated into abortion, either spontaneous or induced, were asked about the utilisation of safe abortion services and the post-abortion complications they experienced. This chapter presents information on antenatal, natal and postnatal care received by women whose last pregnancy had terminated during the three years preceding the survey as live birth or as stillbirth.

4.1 Antenatal Check-Ups

Women who had given a birth during the three years preceding the survey were asked whether they had gone for antenatal check-ups outside the home, and if they had, what type of service provider had given them the check-ups. They were also asked whether any health worker had visited them at home to provide antenatal check-ups. Table 4.1 and Figure 4.1 present the percentage of women who had given birth during the three years preceding the survey, and information regarding the antenatal check-ups they had by source of antenatal check-ups according to some selected background characteristics. Results show that nine out of every ten women received antenatal check-ups during the three years preceding the survey. Forty two percent of women received antenatal check-ups from doctors and 47 percent from ANM/Nurse/LHV. Only 3 percent women received antenatal check-ups at the doorstep from the ANMs or health worker.

Figure 4.1
Source of Antenatal care

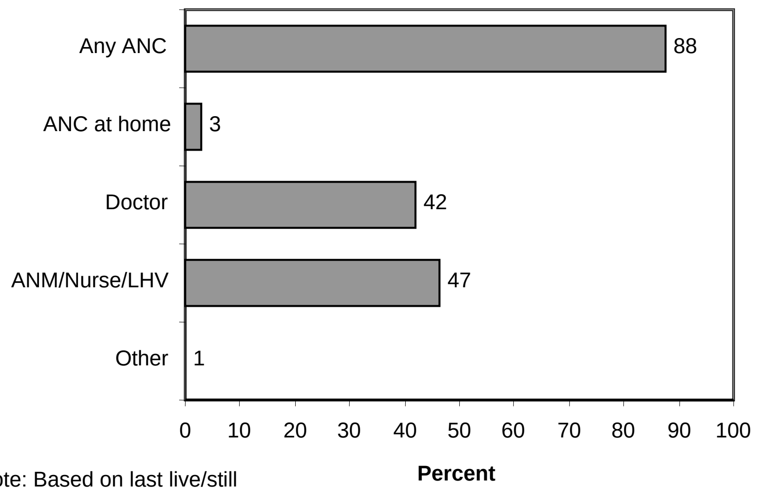

Note: Based on last live/still birth, born during three years preceding the survey

Haryana, DLHS-RCH, 2002-04

Antenatal check-ups are more common among younger women age below 35 years than among older women, and it is more common among those women who had given their first birth. The percentage of women who received antenatal check-up was higher in urban areas (93 percent) than in rural areas (86 percent) and the percentage of women who received antenatal check-ups from doctors is also higher in urban areas (65 percent) than in rural areas (33 percent) and on the other hand 53 percent of rural women received antenatal check-ups from auxiliary nurse midwife, nurse or LHVs, the same for women in urban areas is 29 percent. Seventy seven percent of non-literate women received antenatal check-ups; nearly all women (98 percent) who had completed high school received antenatal check-ups for their last pregnancy that terminated into births (either live or still birth) during the three years preceding the survey.

Table 4.1 ANTENATAL CHECK-UP

Percentage of women* who received any antenatal check-up (ANC) during pregnancy by source of antenatal provider, according to selected background characteristics, Haryana, 2002-04

Background Characteristics	Any[1] antenatal check-up	Antenatal check-up only at home by ANM	Health personnel providing ANC[2]				
			Doctor	ANM/ Nurse/ LHV	Other health professional	Other[3]	
**Age group**							
Less than 20 years	85.6	3.5	32.5	54.6	0.0	0.0	599
20-34 years	89.0	2.9	43.5	46.3	0.3	0.5	5,713
35 years & above	70.0	2.5	32.8	36.5	0.0	0.4	361
**Children ever born**							
1	92.6	2.0	53.8	41.3	0.1	0.3	1,921
2	91.7	3.4	45.0	48.1	0.3	0.4	1,961
3	89.0	2.5	39.1	51.1	0.1	0.6	1,247
4+	74.9	3.8	25.1	47.0	0.5	0.8	1,516
**Residence**							
Rural	85.7	3.0	33.3	53.0	0.2	0.4	4,877
Urban	93.1	2.5	65.3	28.8	0.4	0.6	1,797
**Education**							
Non-literate	76.7	4.4	23.1	50.6	0.4	0.5	2,672
0-9 @ years	92.4	2.1	42.4	52.4	0.2	0.5	2,243
10 years & above	98.3	1.5	70.1	32.8	0.1	0.5	1,758
**Religion**							
Hindu	90.7	2.8	43.7	48.3	0.2	0.5	5,796
Muslim	53.2	3.3	14.9	35.5	0.3	0.0	565
Sikh	92.8	3.2	58.4	31.3	0.4	0.5	290
**Caste/tribe#**							
Scheduled caste	88.6	3.2	31.8	55.8	0.4	0.6	1,650
Scheduled tribe	(95.3)	(9.3)	(30.2)	(55.8)	(0.0)	(0.0)	49
Other backward class	80.9	3.0	36.9	44.2	0.1	0.5	2,265
Other	92.6	2.6	52.6	42.3	0.3	0.4	2,709
**Standard of living index**							
Low	70.4	4.6	15.0	52.1	0.1	0.7	1,359
Medium	88.8	2.7	36.8	53.4	0.4	0.3	2,971
High	96.2	2.2	64.1	34.4	0.2	0.6	2,344
**Availability of health facility[4] in the village**							
No	83.1	3.3	31.4	51.4	0.2	0.5	1,788
Yes	87.1	2.9	34.5	53.9	0.2	0.4	3,089
Total	87.6	2.9	41.9	46.5	0.3	0.5	6,674

Note: * Women who had their last live/still birth since 1-1-1999/1-1-2001.
Note: Total includes 29 women with zero parity and 2 women missing information on education who were not shown separately. Total includes 23 women in other religion who were not shown separately.
[1] Antenatal check-ups either at home or outside from home at health facility.
[2] Antenatal check-ups outside home and percentage add more than 100.0 due to multiple responses
[3] Other also includes trained and untrained *dai* .
Total figure may not add to N due to do not know and missing cases. @ Literate women with no years of schooling are also included. [4] Includes sub-centre, primary health centre, community health centre or referral hospital, government hospital, and government dispensary within the village.
() Based on less than 50 unweighted cases.

The proportion of women who received antenatal check-ups from a doctor, increased steadily with the level of education and the standard of living index. Twenty three percent non-literate women as compared to 70 percent having education of more than 10 years received ANC from doctors. Similarly, 15 percent women belonging to households with a low standard of living against 64 percent of that from a high standard of living fall in this category. The proportion of Sikh women who received antenatal check-ups from doctors (58 percent) was higher than that of Hindu women (44 percent) and Muslim women (15 percent). Fifty three percent of women from the 'other castes' category received antenatal check-ups from doctors, while it was 32 percent for scheduled caste women and 30 percent for scheduled tribe women, and for women from other backward classes, it was 37 percent. Women from scheduled castes and tribes were more likely to receive antenatal check-ups from auxiliary nurse midwives, or LHVs (56 percent), while it was 44 percent among other backward class women and 42 percent of women from the 'other' castes category.

4.2 Antenatal Check-Ups at Health Facility

DLHS-RCH asked women who had a birth during the three years preceding the survey whether women had received antenatal check-ups, and if they had, from where they had availed such services.

Table 4.2 shows the percentage of women who had received antenatal check-ups during pregnancy by place. During pregnancy, women received antenatal check-ups from multiple sources such as, health workers providing ANC at home, Government health facility, private health facility, and at Indian System of medicine etc. Women who received antenatal check-ups both at home and outside the home are categorised as having received care outside the home. Around 46 percent of women received antenatal check-ups at Government health facility, including 8 percent through primary health centre and 19 percent through sub-centre, and 27 percent at a private health facility. Other than this, 4 percent of women reported that they had received antenatal check-ups at private Indian system of medicine. As mentioned above women availed antenatal check-ups from multiple sources. Women who were visited by an ANM might have also visited government and/ or private health facilities including Indian system of medicine.

Younger women were more likely to receive antenatal-check-ups at government health facilities (51 percent) than older women 46 percent for age 20-34 and 36 percent for age 35 and above. Forty eight percent women from rural areas availed government health facilities for antenatal check-ups that were higher than women in urban areas (42 percent) and a high proportion of women (44 percent) from urban areas availed private health facilities for antenatal check-ups than women from rural areas (20 percent). It may be mentioned that about one-fourth of the women from rural areas and younger women aged below 20 years (25 percent each) received antenatal check-ups at sub-centre. This indicates that the services are not reaching the target population, particularly through the public sector. A comparatively high proportion of women who received antenatal check-ups at Government health facilities are literate (0-9 years), Hindu, scheduled caste, living in households with a medium standard of living and women from those villages where health facilities are available.

Table 4.2 PLACE OF ANTENATAL CHECK-UP

Percentage of women* who received any antenatal check-ups (ANC) during pregnancy by source and place of antenatal check-ups, according to selected background characteristics, Haryana, 2002-04.

Background characteristic	Antenatal check-up only at home	Place of antenatal check-ups[1]							Number of Women
		Government[2] health facility	Private health facility	PHC	SC	ISM[4] facility		Other	
						Govt.	Private		
**Age group**									
Less than 20 years	3.5	50.8	19.4	7.7	25.4	0.0	4.1	15.1	599
20-34 years	2.9	46.2	27.8	8.4	18.3	0.1	4.0	12.6	5,713
35 years & above	2.5	35.5	18.9	9.2	14.2	0.4	4.5	15.0	361
**Children ever born**									
1	2.0	45.5	15.4	6.5	14.9	0.1	5.7	9.4	1,921
2	3.4	50.3	35.9	9.3	18.2	0.2	3.5	11.6	1,961
3	2.5	47.8	27.6	8.4	21.6	0.1	3.3	14.8	1,247
4+	3.8	39.6	24.9	10.0	22.6	0.1	3.1	19.1	1,516
**Residence**									
Rural	3.0	47.6	20.3	10.6	25.1	0.1	3.5	16.6	4,877
Urban	2.5	41.8	43.6	3.0	3.0	0.2	5.4	3.9	1,797
**Education**									
Non-literate	4.4	43.0	13.7	9.4	25.2	0.1	2.7	18.3	2,672
0-9 @ years	2.1	52.5	25.9	8.8	21.7	0.2	3.4	13.3	2,243
10 years & above	1.5	42.5	47.0	6.8	7.9	0.1	6.4	6.4	1,758
**Religion**									
Hindu	2.8	49.2	26.9	9.0	19.0	0.1	4.0	12.5	5,796
Muslim	3.3	20.9	12.3	1.6	22.6	0.4	3.5	23.7	565
Sikh	3.2	34.5	45.1	3.9	10.3	0.0	6.4	8.6	290
**Caste/tribe#**									
Scheduled caste	3.2	53.9	16.7	11.7	25.9	0.0	2.1	16.1	1,650
Scheduled tribe	(9.3)	(48.8)	(27.9)	(8.1)	(13.5)	(0.0)	(2.7)	(13.5)	49
Other backward class	3.0	42.1	23.8	6.8	19.8	0.1	3.6	14.1	2,265
Other	2.6	44.5	35.0	7.6	13.8	0.2	5.6	10.2	2,709
**Standard of living index**									
Low	4.6	39.1	8.1	10.8	29.5	0.1	2.2	25.5	1,359
Medium	2.7	53.3	20.5	10.1	24.1	0.1	3.0	13.9	2,971
High	2.2	40.9	45.0	5.4	8.1	0.2	6.0	6.7	2,344
**Availability of health facility[5] in the village**									
No	3.2	36.2	21.2	5.9	17.6	0.1	3.6	25.1	1,788
Yes	2.9	54.3	19.8	13.1	29.2	0.1	3.5	11.9	3,089
Total	2.9	46.1	26.6	8.4	18.7	0.1	4.1	12.9	6,674

Note: * Women who had their last live/still birth since 1-1-1999/1-1-2001.
Note: Total includes 29 women with zero parity, 2 women with missing information on education who were not shown separately. Total includes 23 women in other religion who were not shown separately.
Total figure may not add to N due to do not know and missing cases.
@ Literate women with no years of schooling are also included.
[1]Antenatal check-ups outside home and percentage add more than 100.0 due to multiple responses. [2] Includes sub-centre, primary health centre, community health centre or rural hospital, urban health centre/ urban health post/ urban family welfare centre, government hospital or dispensary. [3] Includes Private hospital/clinic or non-governmental hospital/ trust hospital or clinic. [4] Indian system of medicine. [5] Includes sub-centre, primary health centre, community health centre or referral hospital, government hospital, and government dispensary within the village.
() Based on less than 50 unweighted cases.

4.3 Antenatal Check-Ups by District

Table 4.3 indicates the antenatal coverage in Haryana that ranges from the highest of cent percent in Ambala to the lowest of 67 percent in Gurgaon. In majority of the districts, except Faridabad, Gurgaon, Kaithal, Panipat, Sirsa and Sonipat 90 or more than 90 percent of women got some kind of antenatal check-ups for their last births during the three years preceding the survey. Antenatal check-up received from doctor is very low in Gurgaon (22 percent) and it is highest in Ambala and Kurukshetra (71 percent each). In 8 out of 19 districts half or more than half of the women received antenatal check-ups by ANM/Nurse/LHV.

The extent of utilisation of government health facilities for antenatal check-ups was higher than that of private health facilities. The range of antenatal check-ups coverage through government facilities was highest in Rohtak (81 percent) to the lowest of 28 percent in Gurgaon and only in one district, Ambala, about half of the women visited private health facility. In Haryana, 9 percent pregnant women in Panipat and Kurukshetra districts availed the Indian system of medicine (either government or private) for an antenatal check-up. In 6 out of 17 districts, more than 5 percent of women availed such services through the Indian system of medicine.

Table 4.3 ANTENATAL CHECK-UPS BY DISTRICT

Percentage of women* who received any antenatal care (ANC), by source and place of antenatal check-ups by district, Haryana, 2002-04

District	Any[1] antenatal check-up	Antenatal check-up only at home by ANM	Health personnel providing ANC		Place of antenatal check-ups		
			Doctor	ANM/ Nurse	Government [2] health facility	Private[3] health facility	ISM[4] facility
Ambala	99.5	0.0	71.0	28.3	38.1	47.4	6.2
Bhiwani	93.8	0.5	32.9	61.7	40.5	20.8	6.5
Faridabad	81.2	3.2	34.4	47.4	42.8	26.6	0.3
Fatehabad	91.8	1.6	49.5	43.0	39.8	32.6	1.1
Gurgaon	67.2	4.1	21.9	42.0	28.4	17.4	2.2
Hisar	95.2	0.2	49.5	45.4	64.6	28.8	0.8
Jhajjar	95.8	0.5	60.8	47.5	70.3	27.7	4.3
Jind	90.8	1.8	28.9	69.9	35.4	23.7	0.7
Kaithal	80.2	8.8	27.1	44.1	43.8	21.2	6.7
Karnal	94.4	1.5	50.3	53.9	58.0	31.1	4.2
Kurukshetra	97.7	0.0	71.0	26.7	50.8	36.6	8.8
Mahendragarh	89.6	1.9	34.0	56.1	61.9	14.6	1.8
Panchkula	97.8	0.5	50.0	61.1	70.0	17.2	0.4
Panipat	88.3	6.7	32.7	49.5	41.4	25.2	9.3
Rewari	89.5	0.3	60.4	29.6	35.7	38.8	2.3
Rohtak	98.1	0.0	64.7	52.6	80.6	26.9	4.9
Sirsa	78.6	15.5	30.2	32.0	32.8	23.6	3.1
Sonipat	84.8	1.3	57.9	26.2	44.7	27.5	5.4
Yamunanagar	91.9	0.6	39.0	56.1	46.6	32.4	2.9
Haryana	87.6	2.9	41.9	46.5	46.1	26.6	3.5

Note: * Women who had last live/still birth during three years preceding the survey. [1] Antenatal check-ups either at home or health facility. [2] Includes sub-centre, primary health centre, community health centre or rural hospital, urban health centre/ urban health post/ urban family welfare centre, government hospital or dispensary. [3] Includes Private hospital/clinic or non-governmental hospital/ trust hospital or clinic. [4] Either government or private Indian system of medicine.

4.4 Components of Antenatal Check-ups

Women who received any kind of antenatal check-ups were asked whether they received each of the several components of antenatal check-ups at least once during their pregnancy. Table 4.4 presents the percentage of women who received specific components of check-ups by residence. Except for X-rays (which are not recommended as a standard component of antenatal care), all of the measurements and tests are part of essential obstetric care or are required for monitoring high-risk pregnancies.

Sixty percent of women had an abdominal examination as the part of the antenatal check-ups and 49 percent had their blood tested. Other common components of antenatal check-ups were urine test and blood pressure checked (45 percent) each, the measurement weight (43 percent) and height (9 percent), internal examination (22 percent) and breast examination (10 percent). About 27 percent of women had a sonogram or ultrasound, 3 percent had an X-ray and only 1 percent of women reported that they had amniocentesis test. All of these measurements or procedures were performed more often during antenatal check-ups in urban areas than in rural areas.

The type of advice received by women who had antenatal check-ups for last live/still births during three years preceding the survey is also presented in Table 4.4. Advice on diet was given to 72 percent of urban women as compared to 57 percent of rural women and 61 percent in general. Twenty percent of the women received advice on danger signs of pregnancy and delivery care (21 percent). Women were less likely to receive advice on breastfeeding and on newborn care (12 percent each). Advice on family planning was given to 5 percent of rural women and 10 percent of urban women.

Table 4.4 COMPONENTS OF ANTENATAL CHECK-UPS

Percentage of women* who received an antenatal check-up by specific components of antenatal check-up, according to residence, Haryana, 2002-04

Components of antenatal check-ups	Total	Rural	Urban
**Antenatal measurements/tests**			
Weight measured	42.9	35.5	61.6
Height measured	8.8	5.3	17.6
Blood pressure checked	45.0	36.3	66.9
Blood tested	49.3	40.8	70.6
Urine tested	45.0	35.7	68.4
Abdomen examined	59.5	54.8	71.1
Internal examined	22.4	17.1	35.7
Breast examined	10.0	7.3	17.0
X-ray	3.4	2.6	5.6
Sonography /ultrasound	26.7	21.6	39.6
Amniocentesis	1.2	0.9	2.2
**Antenatal advice**			
Diet	61.2	56.8	72.4
Danger signs of pregnancy	20.3	17.3	27.7
Delivery care	20.6	17.9	27.2
Breast feeding	12.2	9.9	17.9
New born care	12.1	9.9	17.8
Family planning	6.3	4.9	9.7
Number of women who received any antenatal check-up	5,849	4,177	1,672

Note: * Women who had their last live/still birth since 1-1-1999/1-1-2001

4.5 Antenatal Care Services

In India, the Reproductive and Child Health Programme includes all pregnant women should be registered in the first 12-16 weeks (Ministry of Health and Family Welfare, 1997). Accordingly the first antenatal check-ups should take place at latest during the first trimester of the pregnancy. It also includes the provision of at least three antenatal care visits, of at least one tetanus toxoid injection and supplementary iron in the form of IFA tablets daily for 100 days. To assess whether the women had received all the care during pregnancy, information was collected regarding number of antenatal visits, timing of the first visit, received tetanus toxoid injection and supplement iron folic acid tablets. The results are presented in Table 4.5. In Haryana, 49 percent of the women received at least three antenatal check-ups and 30 percent had four or more check-ups. At least three antenatal check-ups were received by 64 percent of women in urban areas compared with 43 percent of women in rural areas. Number of visits for antenatal care varies by education, children ever born, religion, caste and standard of living index. Thirty two percent of non-literate, 50 percent literate women (educated below high school) and 72 percent of women who had 10 or more years of schooling visited for minimum three antenatal care. Parity of women is negatively associated with antenatal check-ups. Three-fifth of women with parity one received three antenatal check-ups compared to less one-third of the women with parity 4 and above.

Table 4.5 ANTENATAL CARE

Percent distribution of women who had live/still births during three years preceding the survey by number of antenatal check-ups, the stage of pregnancy at the time of first check-up, the number of tetanus toxoid injections received and were given iron folic acid (IFA) tablets/syrup during pregnancy, and percentage who received full antenatal check-ups by some selected background characteristics, Haryana, 2002-04

Antenatal care indicators	Total	Residence		Education			Children ever born			
		Rural	Urban	Non-literate	0-9@ years	10 years & above	1	2	3	4+
**Number of ANC visits**										
No visit	12.3	14.3	6.9	23.1	7.6	1.7	7.4	8.1	11.0	25.0
1	7.3	8.2	5.1	9.8	7.1	3.9	5.4	7.5	8.3	8.8
2	31.8	34.9	23.6	35.3	35.2	22.3	25.8	32.3	35.6	35.5
3	18.4	19.3	15.9	18.2	21.2	15.1	19.3	18.7	19.1	16.2
4+	30.1	23.3	48.5	13.4	28.9	57.0	42.1	33.1	26.0	14.5
Missing	0.1	0.1	0.1	0.2	0.0	0.0	0.0	0.2	0.0	0.1
Stage of pregnancy at the time of the first antenatal check-up										
No antenatal check-up	12.3	14.3	6.9	23.1	7.6	1.7	7.4	8.1	11.0	25.0
First trimester	45.0	41.4	54.8	32.2	47.8	60.9	53.3	48.7	42.7	31.7
Second trimester	36.3	37.5	33.2	36.1	38.9	33.4	34.6	37.2	40.0	34.0
Third trimester	6.3	6.8	5.0	8.4	5.7	4.0	4.7	5.8	6.3	9.2
Missing	0.1	0.1	0.1	0.2	0.0	0.0	0.0	0.2	0.0	0.1
**Women who received TT**										
No TT	13.8	15.7	8.7	26.0	8.3	2.2	7.9	9.8	12.2	27.8
1	8.3	9.0	6.4	10.1	8.3	5.4	6.1	9.2	10.0	8.4
2+	77.5	75.0	84.3	63.4	83.1	91.8	85.7	80.5	77.2	63.6
Do not remember/missing	0.4	0.3	0.6	0.4	0.3	0.5	0.3	0.6	0.6	0.2
**Women who received IFA tablets/syrup**										
No IFA/syrup	31.5	31.8	30.6	43.8	25.9	19.8	24.7	29.0	32.2	42.8
Received but not consumed	6.6	7.4	4.3	6.8	7.6	4.9	6.3	6.8	8.3	5.3
Consumed one IFA per day	37.9	37.6	38.6	30.1	41.7	44.8	42.5	40.2	36.3	30.3
Received 100+ IFA tablets/syrup	17.1	15.6	21.4	10.1	15.4	30.1	22.1	19.6	15.0	9.5
Percentage of women who received full [1] antenatal check-ups	11.8	9.5	17.8	5.0	10.2	24.0	16.9	13.3	9.6	5.0
Number of women	6,674	4,877	1,797	2,672	2,243	1,758	1,921	1,961	1,247	1,516

Note: Total includes 29 women with zero parity and 2 women with missing information on education who were not shown separately.
@ Literate women with no years of schooling are also included.
[1] At least three visits for antenatal check-ups, at least one TT injection received and was given adequate amount of IFA tablets/syrup.

Contd…

Table 4.5 ANTENATAL CARE (contd…)

Percent distribution of women who had live/still births during three years preceding the survey by number of antenatal check-ups, the stage of pregnancy at the time of first check-up, the number of tetanus toxoid injections received and iron and were given iron folic acid (IFA) tablets/syrup during pregnancy, and percentage who received full antenatal check-ups by some selected background characteristics, Haryana, 2002-04

Antenatal care indicators	Religion			Caste#				Standard of living index			Availability of health facility[2] in the village	
	Hindu	Muslim	Sikh	Scheduled caste	Scheduled tribe	Other backward class	Other	Low	Medium	High	No	Yes
**Number of ANC visits**												
No visit	9.3	46.1	7.2	11.4	(4.7)	19.0	7.3	29.4	11.1	3.8	16.9	12.7
1	7.4	8.1	4.3	8.0	(4.7)	7.9	6.6	8.3	9.1	4.5	7.9	8.3
2	33.2	22.6	24.9	39.6	(46.5)	29.7	28.6	36.4	35.9	24.0	34.1	35.3
3	19.3	11.6	13.7	20.8	(23.3)	18.2	17.0	16.8	20.2	17.0	18.9	19.6
4+	30.9	10.8	49.9	20.2	(20.9)	25.0	40.4	9.0	23.6	50.6	22.2	24.0
Missing	0.0	0.8	0.0	0.0	(0.0)	0.1	0.1	0.2	0.1	0.0	0.0	0.1
Stage of pregnancy at the time of the first antenatal check-up												
No antenatal check-up	9.3	46.1	7.2	11.4	(4.7)	19.0	7.3	29.4	11.1	3.8	16.9	12.7
First trimester	46.8	18.1	59.0	42.1	(51.2)	37.5	52.9	29.1	41.6	58.5	38.9	42.8
Second trimester	37.6	27.1	29.8	39.7	(30.2)	36.8	33.9	33.5	39.9	33.4	38.2	37.0
Third trimester	6.3	8.0	4.0	6.7	(14.0)	6.6	5.7	7.8	7.4	4.2	5.9	7.3
Missing	0.0	0.8	0.0	0.0	(0.0)	0.1	0.1	0.2	0.1	0.0	0.0	0.1
**Women who received TT**												
No TT	10.6	50.7	7.9	13.3	(11.6)	20.4	8.7	32.0	12.6	4.8	18.0	14.3
1	8.4	8.1	5.6	9.4	(9.3)	8.1	7.7	9.3	9.8	5.7	9.2	8.8
2+	80.7	41.1	85.1	77.1	(79.1)	71.3	82.9	58.5	77.3	88.8	72.5	76.4
Do not remember/missing	0.4	0.2	1.4	0.3	(0.0)	0.2	0.6	0.2	0.3	0.7	0.2	0.4
**Women who received IFA tablets/syrup**												
No IFA/syrup	28.7	60.7	29.4	29.0	(25.6)	35.6	29.7	46.5	30.2	24.4	36.5	29.1
Received but not consumed	6.9	5.3	2.7	7.8	(2.3)	6.9	5.7	5.8	8.0	5.2	6.6	7.9
Consumed one IFA per day	39.1	21.2	45.3	39.9	(41.9)	36.5	37.6	31.2	37.4	42.3	36.4	38.3
Received 100+ IFA tablets/syrup	18.0	6.7	20.0	13.7	(18.6)	14.8	21.2	8.5	14.6	25.4	13.4	16.8
Percentage of women who received full [1] antenatal check-ups	12.2	5.2	14.6	7.7	(11.6)	9.4	16.2	3.6	8.9	20.1	8.3	10.3
Number of women	5,796	565	290	1,650	49	2,265	2,709	1,359	971	2,344	1,793	3,084

Note: Total includes 23 women in other religion who were not shown separately.# Total figure may not add to N due to don't know and missing cases.

[1] At least three visits for antenatal check-ups, at least one TT injection received and was given adequate amount of IFA tablets/syrup.

[2] Includes sub-center, primary health center, community health center or referral hospital, government hospital, and government dispensary within the village, () Based on less than 50 unweighted cases.

Hindu women (50 percent) were more likely to have at least three visits for antenatal check-ups than Muslim women (22 percent). Coverage is substantially lower for women from scheduled caste (41 percent) than to women of other than scheduled caste (43-57 percent). Having three or more antenatal visits also increased with the standard of living-26 percent for women with a low standard of living, 44 percent for women with a medium standard of living and 68 percent for women with a high standard of living. Availability of health facility in the village makes a little difference to the minimum three visits for antenatal check-ups.

Data on timing of first antenatal check-ups shows that about half of the women (45 percent) received their first antenatal check-up in the first trimester of pregnancy and another 36 percent received their first check-up in the second trimester and 6 percent of women received their first check-up in the third trimester. A relatively high proportion of women in the urban areas (55 percent) as compared to those in rural areas (41 percent) had a check-up in the first trimester of pregnancy. The first antenatal check-up in the first trimester has steadily increased with education. Thirty two percent of non-literate women had undergone their first antenatal check-up in the first trimester and 61 percent of women who had 10 or more years of schooling received their first antenatal check-up in the first trimester. More than half of the women (53 percent) with parity-1 were visited in first trimester and only one-third (32 percent) women with parity- four and above had undergone antenatal check-up in first trimester. Muslim women were less likely to go for first antenatal check-up in first trimester of their pregnancy as compared to Hindu and women of other religion, and one-third (38 percent) of other backward class women were visited in first trimester for first antenatal check-ups compared with 51 percent to scheduled tribe women, 42 percent of schedule caste women and 53 percent women from ‘other’ caste category. Twenty nine percent women with low standard of living, 42 percent with medium standard of living and 59 percent of women with high standard of living respectively had undergone their first antenatal check-up in the first trimester of their pregnancy period

Nutritional deficiencies in women are often exacerbated during pregnancy because of the additional nutrient requirements of foetal growth; therefore a pregnant woman needs six times more iron than a non-pregnant woman. The information on receiving iron folic acid tablets/syrup during pregnancy is also collected. Table 4.5 shows that women in Haryana received IFA supplements for two-thirds (68 percent) of the last birth during three years preceding the survey. The coverage of IFA tablets is relatively high in urban areas (69 percent) than in rural areas (68 percent). IFA coverage is much low for non-literate women, women with low standard of living, scheduled caste-tribe women, and women of higher parity. IFA coverage is also lower among Muslim women (39 percent) than Hindu (64 percent) and Sikh (68 percent) women. Again, during pregnancy in the last three years preceding the survey, only 17 percent of women received 100 or more IFA, 16 percent in rural areas and 21 percent in urban areas. Intake of 100 or more IFA is positively associated with education and standard of living index and negatively associated with parity. Women from schedule tribe received more 100 or more IFA than their counterparts. Such a large difference in receiving IFA or intake of 100 or more IFA tablets/syrup is not found while analysing the situation by availability of health facility in the village.

For the last live birth or stillbirth during the three years preceding the survey, women were asked whether they were given tetanus toxoid injection to prevent them and their baby from getting tetanus. Table 4.5 shows that 78 percent of the women received two or more tetanus toxoid injections. Coverage of two or more TT injection is slightly higher in urban areas (84 percent) than that in rural areas (75 percent). The coverage of at least one tetanus toxoid injection for Sikh women (91 percent) is more than that for Muslim women (49 percent) and Hindu women (89 percent). Coverage of at least one tetanus toxoid injection is more for' other' caste category women (91 percent) than other backward classes (79 percent). Non-literate women received at least one tetanus toxoid injection for 74 percent of their last birth, whereas literate women with 9 years of schooling received at least one tetanus toxoid injection for 91 percent, and women who had completed 10 years or more of schooling received at least one tetanus toxoid injection for 97 percent of their last birth. Ninety five percent of women with a high standard of living received at least one tetanus toxoid injection, and 68-87 percent women with low or medium standard of living received at least one tetanus toxoid injection for their last live/still birth. The coverage varies inversely by parity. At least one tetanus toxoid injection was received by 92 percent women of Parity-1 compared with 72 percent of Parity 4 and above.

Figure 4.2
Full Antenatal care by background Characteristic

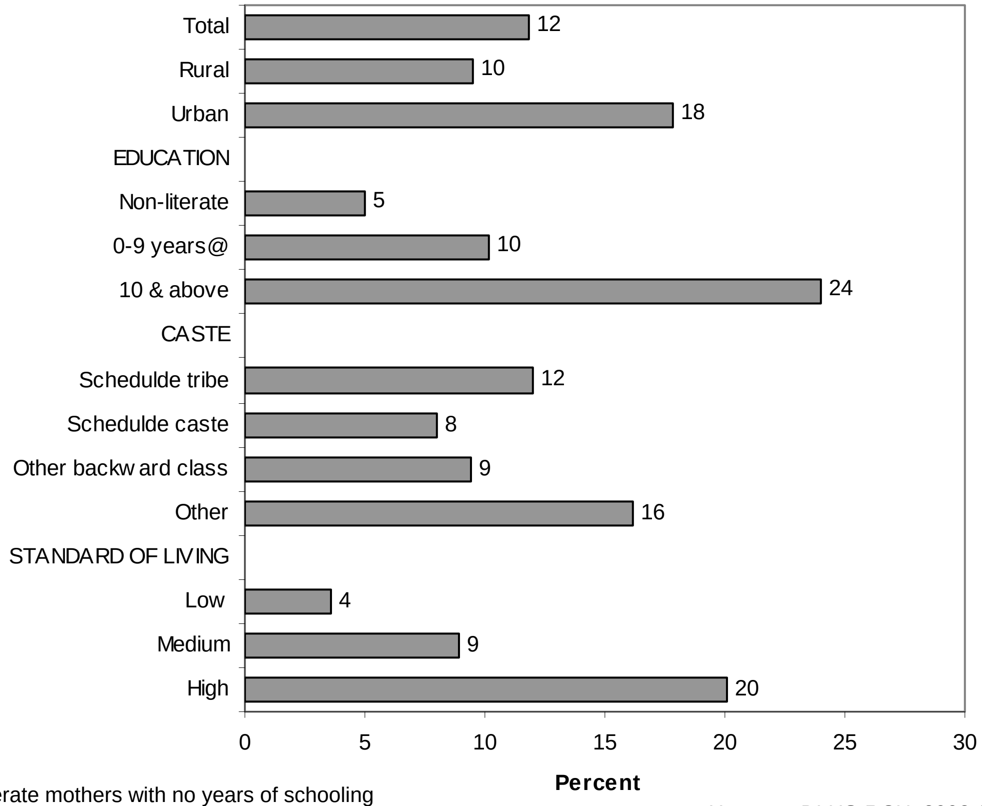

@ Literate mothers with no years of schooling are also included

Haryana, DLHS-RCH, 2002-04

The percentage of women who received full antenatal care (that is, at least three antenatal check-ups, and at least one tetanus toxoid injection and supplementary iron in the form of IFA tablets daily for 100 days as recommended by the RCH programme,) has been presented in Figure 4.2. Only 12 percent of women in Haryana received full antenatal care. Coverage of full antenatal care is low for non-literate women, women with higher parity, Muslim women, women from scheduled caste, women with a low standard of living and women from those villages where health facilities are not available. Full antenatal coverage was also lower in rural areas (10 percent) than in urban areas (18 percent).

4.6 Antenatal Care Indicator by District

Table 4.6 shows the percentage of women who had given live/still birth during the three years preceding the survey who received different types of antenatal care; (the percentage who received antenatal check-up in the first trimester of pregnancy, the percentage who received at least three antenatal check-ups, the percentage who received at least one tetanus toxoid injection, the percentage given 100 or more iron folic acid tablets/syrup, and the percentage who received full antenatal care services) by district.

Table 4.6 ANTENATAL CARE INDICATORS BY DISTRICT

Percentage of women* who received different type of antenatal care by district, Haryana, 2002-04

District	Percentage that received an antenatal check-up in the first trimester of pregnancy	Percentage that received three or more antenatal check-ups	Percentage that received at least one tetanus toxoid injection	Percentage that received adequate amount of IFA[1]	Percentage that received full[2] antenatal check-ups
Ambala	62.5	80.5	98.9	20.7	19.2
Bhiwani	43.2	47.6	92.2	20.5	15.6
Faridabad	36.3	43.9	80.1	10.3	6.5
Fatehabad	41.3	43.5	88.0	10.2	7.7
Gurgaon	28.0	34.2	62.4	13.4	10.0
Hisar	61.2	57.6	92.1	18.0	13.9
Jhajjar	42.8	55.4	92.7	22.8	15.4
Jind	51.5	41.4	88.0	14.7	6.6
Kaithal	46.5	40.4	73.7	23.9	13.1
Karnal	47.3	56.3	90.8	21.1	13.2
Kurukshetra	61.7	74.0	97.0	23.3	19.5
Mahendragarh	34.2	35.9	90.7	11.0	7.1
Panchkula	51.1	68.0	96.1	27.7	20.8
Panipat	50.4	46.7	84.4	17.1	13.3
Rewari	44.6	47.2	94.6	19.9	12.7
Rohtak	60.5	60.1	97.5	26.8	19.0
Sirsa	40.9	34.5	78.7	14.8	7.2
Sonipat	46.0	46.2	87.1	14.3	8.8
Yamunanagar	37.3	52.2	89.7	17.8	12.2
Haryana	45.0	48.6	85.8	17.1	11.8

Note: * Women who had their last live/still birth since 1-1-1999/1-1-2001

[1] 100 or more iron folic acid tablets including syrup

[2] At least three visits for antenatal check-ups, at least one TT injection received and adequate amount of IFA

The utilisation of antenatal care services differs from district to district. In 7 out of 19 districts more than half the women received their first antenatal check-up in the first trimester of pregnancy. The percentage of women who received at least three visits for antenatal check-ups ranges from 34 percent in Gurgaon to 81 percent in Ambala. (see Map-3). There has been good coverage of tetanus toxoid injection in the all districts, ranging from 62 to 99 percent, but on the other hand, performance regarding receipt of 100 or more IFA is poor. In all the districts, the value ranges from 10 to 28 percent and it is lowest in Faridabad and Fatehabad. The percentage of women who received full antenatal care ranges from 7 percent in Faridabad, Jind, Mahendragarh and Sirsa to 21 percent in Panchkula. In 7, out of 19 of districts coverage rate of full antenatal care is below than that of the state average (12 percent).

4.7 Pregnancy Complications and Treatment

Complications during pregnancy may affect both women's health and the outcome of the pregnancy adversely. Early detection of complications during pregnancy and their management are important components of the safe motherhood programme. In the survey, all the eligible women who had given last live or still birth during the three years preceding the survey were asked if at any time during the pregnancy, they had experienced any of the following pregnancy-related problems such as swelling of hands and feet, paleness, visual disturbance, vaginal bleeding, convulsions, weak or no movement of foetus, abnormal position of foetus, and other problems. All the information is based on women's self-reporting which is presented in Table 4.7 and Figure 4.3.

Figure 4.3
Percentage of Women with pregriancy Complication and by symptoms

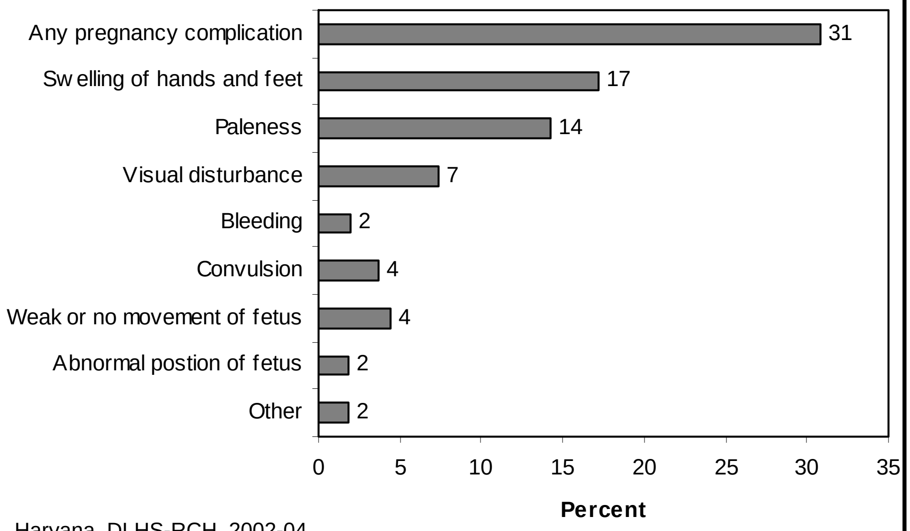

Haryana, DLHS-RCH, 2002-04

Table 4.7 PREGNANCY COMPLICATIONS

Percentage of women who had live/still births during three years preceding the survey by pregnancy complication and type of complication during pregnancy by some selected background characteristics, Haryana, 2002-04

Background characteristic	Percentage of women with any pregnancy complication	Type of pregnancy complication;								Number of women
		Swelling of hands and feet	Paleness	Visual disturbances	Bleeding	Convulsion	Weak or no movement of foetus	Abnormal position of foetus	Other	
**Age group (years)**										
15-19	32.1	16.4	16.0	6.8	1.1	2.9	5.5	2.2	1.6	599
20-24	30.0	16.0	13.9	7.5	1.9	3.4	4.8	1.9	2.2	2,901
25-29	31.6	19.0	14.4	6.5	2.4	3.8	4.1	1.9	1.4	2,035
30-34	31.3	17.9	13.4	8.6	2.1	4.4	3.6	2.4	3.2	777
35-39	31.4	17.9	15.7	9.8	3.4	5.7	4.4	1.2	0.5	271
40-44	20.8	11.6	11.8	7.3	0.0	1.6	1.6	1.5	0.0	90
**Children ever born**										
1	32.6	20.6	12.3	4.4	1.8	2.1	4.6	2.4	1.7	1,921
2	29.5	14.8	13.8	7.0	1.9	3.6	4.4	1.7	2.2	1,961
3	30.7	15.9	16.2	9.0	2.7	4.1	4.4	1.5	1.8	1,247
4+	29.4	16.7	15.4	10.4	1.8	5.3	4.0	1.7	1.8	1,516
**Residence**										
Rural	29.5	15.0	14.2	8.1	2.0	3.8	4.5	1.7	1.9	4,877
Urban	34.2	23.1	14.3	5.5	2.2	3.2	4.2	2.4	1.9	1,797
**Standard of living index**										
Low	27.4	14.4	13.3	9.5	1.4	3.8	3.7	0.9	1.9	1,359
Medium	30.3	15.4	15.3	8.0	2.6	3.9	5.0	2.0	1.6	2,971
High	33.2	21.1	13.5	5.3	1.7	3.2	4.2	2.4	2.3	2,344
**Received any ANC**										
Yes	31.6	17.5	14.8	7.3	2.1	3.7	4.6	2.0	2.1	5,849
No	25.1	15.0	10.7	8.1	1.6	3.1	3.1	1.1	0.9	819
Total	30.8	17.2	14.2	7.4	2.0	3.7	4.4	1.9	1.9	6,674

Note: Total include 29 women with zero parity, 5 with missing information on whether received any ANC who were not shown separately. @ Literate women with no years of schooling are also included.

About 31 percent of the women experienced at least one pregnancy related problem. The proportion was higher among urban women (34 percent) than among rural women (30 percent). Women with higher parity face at least one pregnancy related problem less than women with low parity do. This proportion is relatively high among women who had received some kind of antenatal care during the pregnancy. Thirty two percent of women who had an antenatal check-up reported that they had experienced at least one problem during their pregnancy while 25 percent of women did not receive any antenatal check-up during their pregnancy fall in this category. The major problems reported were ‘swelling of hand and feet’ (17 percent), ‘paleness’ (14 percent), and ‘visual disturbance’ (7 percent). Only 4 percent reported ‘weak or no movement of foetus’ and ‘convulsions’ whereas ‘vaginal bleeding’ and ‘abnormal position of foetus’ reported by 2 percent. Other problems related to pregnancy were reported by 2 percent of women. Swelling of hands and feet is more common among older women and women with high a standard of living. The percentage of women who were more anaemic belonging to the age group 15-19 years and 35-39 years, women with a medium standard of living and women who received any kind of antenatal care during the pregnancy. Anaemia, visual disturbance and convulsion increased steadily with increase of parity, whereas women with parity-1 reported weak or no movement of foetus and abnormal position of foetus more. The younger women (15-19 years of age) were more likely to report vaginal bleeding and weak or no movement of foetus as pregnancy complications.

Women who reported at least one pregnancy related complication were asked whether they had consulted someone or had sought treatment for their problem and also the source of treatment. Table 4.8 shows the percentage of women who had pregnancy complications who obtained advice or had sought treatment by source of treatment according to residence and availability of health facility in the village. Forty nine percent of women reported that they had obtained advice or consulted someone for their problem. The proportion was higher among urban women (57 percent) than among rural women (45 percent) and 47 percent of women sought treatment from those villages where health facility was available as compared to 43 percent of women with non-availability of health facility within the village.

Among women who sought treatment for pregnancy complications, 34 percent visited a government health facility along with primary health centre and sub-centre (4 percent each). Three-fifth of them visited a private health facility and 4 percent had gone to a facility with the Indian system of medicine while another 3 percent obtained advice from another health facility. The proportion of women who visited a private health facility is higher in urban areas (68 percent) than in rural areas (57 percent). Among women who sought treatment, 81 percent went to a doctor and 16 percent to an auxiliary nurse midwife or nurse or LHV and another 2 percent to someone else. Seventy eight percent of these women in rural areas and 88 percent in urban areas were examined by a doctor; whereas ANM/Nurse/LHV examined 20 percent women in rural areas and 11 percent in urban areas.

TABLE 4.8 TREATMENT FOR PREGNANCY COMPLICATIONS

Percentage of women* who had any pregnancy complication, sought treatment and source of treatment according to residence and availability of health facility in the village, Haryana, 2002-04

Treatment and source	Total	Residence		Availability of health facility[5] in the village	
		Rural	Urban	No	Yes
Percentage of women sought treatment who had any pregnancy complication	48.8	45.4	56.8	43.1	46.7
Number of women	1,002	653	349	221	432
Percentage sought treatment at health facility					
Government health facility[1]	34.4	37.0	29.4	32.7	39.1
Primary health centre	4.4	6.0	1.3	3.1	7.5
Sub centre	3.6	5.4	0.2	4.7	5.8
Private health facility[2]	60.5	56.6	68.0	59.7	54.8
ISM[3] facility	4.0	4.9	2.4	6.0	4.4
Other	3.0	3.9	1.5	5.1	3.2
Percent distribution of women who obtained treatment from					
Doctor	81.3	77.9	87.6	78.1	77.8
ANM/nurse/midwife/LHV	16.4	19.5	10.6	19.6	19.4
Other[4]	1.8	2.1	1.2	2.3	2.1
Missing	0.5	0.5	0.6	0.0	0.7
Total percent	100.0	100.0	100.0	100.0	100.0
Number of women	1,002	653	349	221	432

Note: [1]Include municipal hospital, dispensary, urban health centre/urban health post/urban family welfare centre, community health centre/rural hospital, primary health centre and sub centre

[2] Include private hospital/clinic and non-governmental organization/ trust hospital

[3] Either government or private Indian system of medicine

[4] Other include Dai trained or untrained, other health professional and ISM practitioner

[5] Includes sub-centre, primary health centre, community health centre or referral hospital, government hospital, and government dispensary within the village

4.8 Delivery Care

4.8.1 Place of Delivery

One of the important thrusts of the Reproductive and Child Health Programme is to encourage deliveries under proper hygienic conditions under the supervision of trained health professionals. The provision of delivery services in the government health institutions is one of the components of the RCH programme. For each live/still birth during three years preceding the survey, DLHS-RCH asked the women where (place) their children were born, who assisted during the deliveries in case of home deliveries, characteristics of delivery and any problems that occurred during the delivery. Table 4.9 and Figure 4.4 present the place of delivery. About 11 percent of the birth took place in government health institutions, 25 percent in private health institutions and slightly less than two-third of the births (65 percent) took place at home. More than half of the deliveries in urban areas and 27 percent of the deliveries in rural areas took place in health institutions. Deliveries in health facilities in Haryana were 35 percent.

Table 4.9 PLACE OF DELIVERY

Percent distribution of women who had given live/still births during three years preceding the survey, by place of delivery, according to selected background characteristics, Haryana, 2002-04

Background characteristics	Health institutions		Home	Other	Missing	Total percent	Number of women
	Public	Private					
**Age group (in years)**							
Below 20	10.6	17.8	71.5	0.0	0.0	100.0	599
20-34	10.6	25.7	63.6	0.0	0.0	100.0	5,713
35 and above	9.7	17.7	72.6	0.0	0.0	100.0	361
**Children ever born**							
1	14.1	36.4	49.4	0.0	0.1	100.0	1,921
2	12.6	25.8	61.6	0.0	0.0	100.0	1,961
3	7.9	20.3	71.8	0.0	0.0	100.0	1,247
4+	5.4	11.3	83.3	0.0	0.0	100.0	1,516
**Residence**							
Rural	8.5	18.8	72.7	0.0	0.0	100.0	4,877
Urban	16.1	40.3	43.6	0.0	0.0	100.0	1,797
**Education**							
Non-literate	6.4	10.4	83.2	0.0	0.0	100.0	2,672
0-9@ years	11.5	22.0	66.4	0.0	0.1	100.0	2,243
10 years & above	15.6	49.3	35.1	0.0	0.0	100.0	1,758
**Religion**							
Hindu	11.3	25.0	63.7	0.0	0.0	100.0	5,796
Muslim	2.1	7.7	90.3	0.0	0.0	100.0	565
Sikh	13.3	47.7	39.0	0.0	0.0	100.0	290
**Caste#**							
Scheduled caste	8.3	13.4	78.3	0.0	0.0	100.0	1,650
Scheduled tribe	(14.0)	(18.6)	(67.4)	(0.0)	(0.0)	100.0	49
Other backward class	8.6	20.1	71.3	0.0	0.0	100.0	2,265
Other	13.5	35.2	51.2	0.0	0.1	100.0	2,709
**Standard of living index**							
Low	4.4	7.4	88.2	0.0	0.0	100.0	1,359
Medium	9.7	16.5	73.7	0.0	0.1	100.0	2,971
High	15.2	44.8	40.0	0.0	0.0	100.0	2,344
**Number of antenatal check-ups**							
No check-up	2.1	6.9	91.0	0.0	0.0	100.0	819
1	7.0	14.1	78.9	0.0	0.0	100.0	490
2	8.2	16.4	75.3	0.0	0.1	100.0	2,124
3	11.3	19.6	69.1	0.0	0.0	100.0	1,227
4+	17.0	46.0	37.0	0.0	0.0	100.0	2,008
**Delivery characteristics**							
Normal	9.2	20.0	70.7	0.0	0.0	100.0	6,036
Caesarean	21.2	73.0	5.8	0.0	0.0	100.0	447
Assisted	27.3	55.0	17.7	0.0	0.0	100.0	190
**Availability of health facility[1] in the village**							
No	9.0	18.7	72.1	0.0	0.1	100.0	1,788
Yes	8.2	18.8	73.0	0.0	0.0	100.0	3,089
Total	10.6	24.6	64.8	0.0	0.0	100.0	6,674

Note: Total includes 29 women with zero parity, 2 cases with missing information on education and 5 on number of ANC visits who were not shown separately. Total includes 23 women in other religion who were not shown separately.# Total figure may not add to N due to do not know and missing cases. @ Literate women with no years of schooling are also included.[1] Includes sub-centre, primary health centre, community health centre or referral hospital, government hospital, and government dispensary within the village, () Based on less than 50 unweighted cases.

The proportion of births occurring in health institutions is higher for young women under 35 years (28-36 percent) than for women aged 35 years and above (27 percent). Institutional deliveries, particularly in private health facilities, increase sharply with education and the standard of living. Less than one-fifth (17 percent) of the births to non-literate women and 65 percent births to literate women who had completed at least 10 or more years of schooling took place at health institutions. Women with a high standard of living were more

likely to give birth in health institutions than women with a low standard of living (Figure 4.4). The proportion of institutional deliveries decreases as parity increases from parity one (51 percent) to parity four and above (17 percent). Institutional delivery is much lower for Muslim women (10 percent) than for Sikh (61 percent) and Hindu women (36 percent). Only 33 percent births of women from scheduled-tribes are institutional deliveries as compared to 22 percent of births to women from scheduled-castes, 29 percent to other backward classes and 49 percent of births to women from the ‘other’ caste category. Institutional deliveries are more common among women who had four or more antenatal check-ups (63 percent) than among who had fewer antenatal check-ups (21-31 percent). Institutional deliveries are least prevalent among births to women who did not receive any antenatal check-ups (9 percent). As expected, most of the births occurred through caesarean section (94 percent) and 82 percent of assisted deliveries took place at health institutions. At the same time, 6 percent of caesarean deliveries and 18 percent of assisted deliveries took place at home. Twenty seven percent of births took place at health institutions in the village with availability of health facility compared to 28 percent of births from villages without any health facility.

4.8.2 Assistance During Home Delivery

Table 4.10 shows distribution of assistance during home delivery by selected background characteristics. Generally, assistance during delivery can be provided by medical staff (doctors, ANM/nurse/LHV, TBA, un-trained *dai*), and relatives/friends. If more than one type of attendant assisted during the delivery, then only the most qualified person is considered. In the last three years only two percent of deliveries were attended by doctors, 10 percent by ANM or nurse or LHV, 25 percent by trained birth attendants, 60 percent attended by untrained dais, only three percent of home deliveries were attended by relatives and friends (Figure 4.4). Overall, health professionals attended 37 percent of deliveries that took place at home. The percentage of births (home delivery) attended by health professionals do not differ much between women age. About 11-13 percent of births attended by health professional for women age below 20 and 20-34 years and only 9 percent of births for women age 35 and above were attended by health professionals. In rural areas, 33 percent of births were attended by health professionals as compared to 58 percent of that in urban areas. The percentages of births attended by health professionals were decreased steadily with increasing with parity of women.

Births to literate women who had completed 10 or more years of schooling which were attended by health professionals is more than two times higher than those of non-literate women. More than one-third (38 percent) of home deliveries to women with a medium standard of living and 25 percent of deliveries to women with a low standard of living were attended by health professionals. Home deliveries are more likely to be attended by health professionals among Sikh women (55 percent) than among Muslim women (18 percent). Only 37 percent of births to women from scheduled castes, 41 percent to scheduled tribes, 32 percent to other backward classes and 44 percent to women belonging to ‘other castes’ category were attended by health professionals. Twenty two percent of home deliveries to women who did not have any antenatal check-ups were attended by health professionals compared to 55 percent of home deliveries to women who had four or more antenatal check-ups. About 37 percent of home deliveries that were normal were attended by health professionals, which differ substantially to births by either caesarean section or assisted (56-89 percent), but the result should be interpreted with caution due to the small number of cases.

Twenty nine percent home deliveries were attended by health professionals in villages with non-availability of a health facility and 35 percent in villages with availability of a health facility.

Table 4.10 ASSISTANCE DURING HOME DELIVERY AND SAFE DELIVERY

Percent distribution of women who had given live/still births during three years preceding the survey, by assistance during home delivery, and percentage of safe delivery, according to selected background characteristics, Haryana, 2002-04

Background characteristics	Attendant assisting during home delivery[1]						Number of women	Percentage of safe delivery
	Doctor	ANM/ Nurse/LHV	TBA	Untrained *dai*	Relative/ friends	None		
**Age group (in years)**								
Below 20	2.7	7.8	25.3	60.7	3.4	0.0	429	36.0
20-34	2.3	10.7	25.3	59.0	2.7	0.0	3,636	44.6
35 and above	1.2	8.0	18.2	67.9	4.7	0.0	262	34.0
**Children ever born**								
1	3.0	13.2	26.6	54.5	2.6	0.0	950	58.5
2	2.6	11.2	26.8	57.1	2.2	0.0	1,208	46.9
3	2.8	9.4	25.5	58.7	3.6	0.1	896	36.9
4+	1.0	7.6	21.4	66.7	3.2	0.0	1,263	23.9
**Residence**								
Rural	2.2	7.8	22.7	64.2	3.0	0.0	3,544	34.6
Urban	2.5	21.2	34.7	39.2	2.4	0.0	783	66.7
**Education**		6.8						
Non-literate	1.4		20.6	67.3	3.8	0.0	2,222	23.7
0-9@ years	2.8	12.2	28.0	55.1	1.8	0.0	1,489	43.5
10 years & above	4.0	17.7	32.6	43.3	2.2	0.1	616	72.5
**Religion**								
Hindu	2.5	10.7	26.2	58.0	2.6	0.0	3,691	44.7
Muslim	0.7	4.1	13.6	76.0	5.7	0.0	510	14.0
Sikh	2.5	21.5	29.9	44.4	1.7	0.0	113	70.4
Other	*	*	*	*	*	*	13	54.1
**Caste#**								
Scheduled caste	1.9	8.4	26.6	61.0	2.1	0.0	1,291	29.8
Scheduled tribe	(0.0)	(10.3)	(31.0)	(58.6)	(0.0)	(0.0)	33	(10.3)
Other backward class	1.7	8.5	21.8	65.0	3.0	0.0	1,614	35.9
Other	3.4	14.0	26.7	52.3	3.6	0.1	1,388	57.6
**Standard of living index**								
Low	1.6	4.7	18.2	71.8	3.7	0.0	1,199	17.3
Medium	2.6	10.0	25.4	58.9	3.0	0.0	2,190	35.6
High	2.3	17.8	32.1	46.2	1.5	0.0	938	68.0
**Number of antenatal check-ups**								
No check-up	1.5	4.6	15.4	73.5	5.0	0.0	745	14.6
1	1.7	8.4	21.9	64.8	3.2	0.0	387	29.1
2	1.5	9.2	26.0	60.5	2.7	0.0	1,600	32.6
3	2.8	10.5	26.4	58.3	2.0	0.0	848	40.1
4+	4.4	18.9	31.9	42.9	1.9	0.0	742	71.6
**Delivery characteristics**								
Normal	1.9	10.1	25.0	60.2	2.8	0.0	4,268	37.7
Caesarean	(16.7)	(27.8)	(11.1)	(33.3)	(11.1)	(0.0)	26	(44.4)
Assisted	(41.7)	(27.8)	(19.4)	(11.1)	(0.0)	(0.0)	34	(69.4)
**Availability of health facility[3] in the village**								
No	2.2	6.5	20.7	67.0	3.6	0.0	1,290	34.1
Yes	2.3	8.6	23.9	62.6	2.6	0.0	2,254	34.9
Total	2.3	10.2	24.9	59.7	2.9	0.0	4,327	43.2

Note: Total includes 11 women with zero parity and 5 women with missing information on number of ANC visits who were not shown separately. * Percentage not shown: Based on very few cases.
@ Literate women with no years of schooling are also included.
# Total figure may not add to N due to do not know and missing cases.
[1] If the respondent mentioned more than one attendant, only the most qualified attendant is shown.
[2] Either institutional delivery or home delivery assisted by doctor/ANM/Nurse/LHV.
[3] Includes sub-centre, primary health centre, community health centre or referral hospital, government hospital, and government dispensary within the village.
( ) Based on less than 50 unweighted cases. *Percentage not shown: Based on very few cases.

Figure 4.4

Place of Delivery and Assistance During Delivery

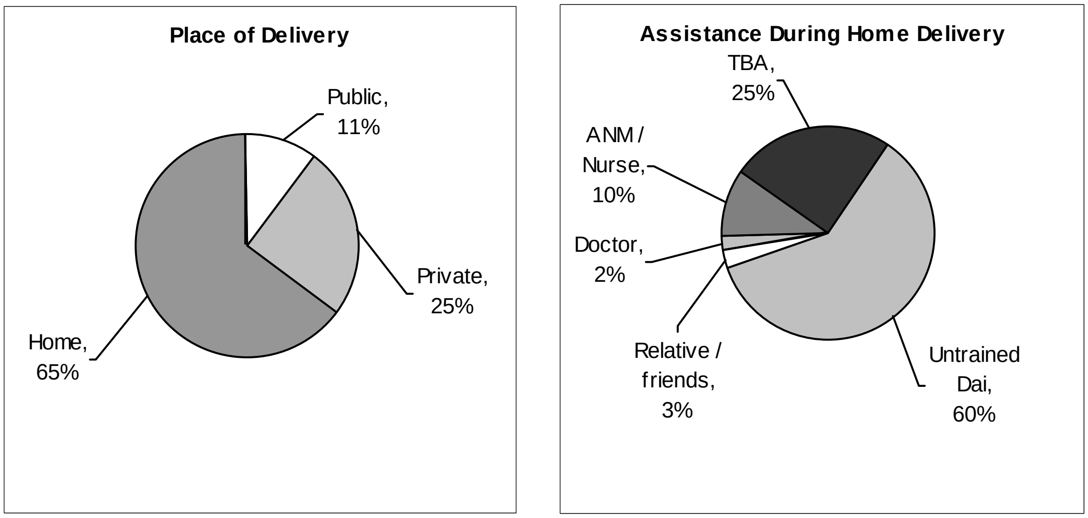

Note: Percentage may add more than 100.0 due to rounding

Haryana, DLHS-RCH, 2002-04

4.8.3 Delivery Assisted by Skilled Persons

The extent of safe deliveries varied substantially by background characteristics of women (Table 4.10 and Figure 4.5). Less than half of the births (43 percent) were safe in Haryana. In urban areas two-third (67 percent) of the deliveries were safe as against more than one-third (35 percent) in rural areas. About 36-45 percent of the deliveries were safe for younger women aged below 35 than to elderly women (34 percent). The proportion of safe deliveries was much lower among Muslim women (14 percent) than among Sikh women (70 percent and women from other religions (45-54 percent). Only 30 percent and10 percent of births to women from scheduled-castes and scheduled-tribes respectively were safe deliveries, compared to 36 percent to women from other backward classes and 58 percent of births to women from ‘other castes’ category. Proportion of safe deliveries decreases as parity rises from 1 (59 percent) to 4 and above (24 percent). Safe deliveries were least prevalent among women who did not receive any antenatal check-ups (15 percent) and it is most prevalent among women who had four or more antenatal check-ups (72 percent). The proportion of safe deliveries increased sizeably with women’s education and standard of living. Only twenty four percent of non-literate women had safe deliveries whereas its prevalence is 73 percent among women who had completed 10 or more years of schooling. Women with a high standard of living had 68 percent safe deliveries compared to 36 percent of women with a medium standard of living and 17 percent with a low standard of living. As compared to women who had caesarean and assisted deliveries (44-69 percent), only 38 percent of women with normal deliveries are safe deliveries. The proportion of safe deliveries was slightly higher in villages with a health facility than to women from those villages were health facilities are not available.

Figure 4.5
Delivery Assisted by Skilled Person by Background Characteristics

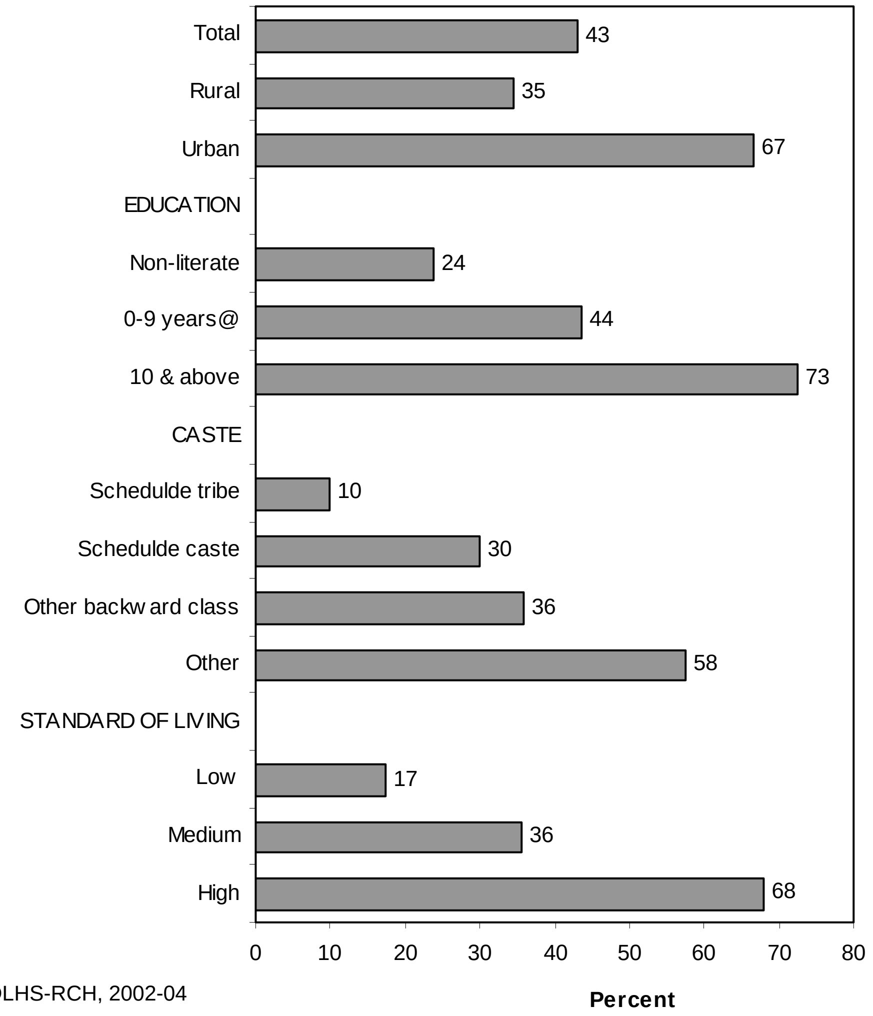

Haryana, DLHS-RCH, 2002-04

4.9 Reasons for Not Going to Health Institutions for Delivery

Table 4.11 shows the percentage distribution of women who did not deliver in health institutions in the three years preceding the survey. The main reason for not going to health institutions has been presented according to residence and availability of health facility in the village. About two-third of the women (60 percent) stated that it was not necessary to deliver in health institutions. It is surprising to see that a higher proportion of urban women (66 percent) than rural women (59 percent) felt this way. Also, 60 percent of women stated that it was not necessary to deliver in health institutions when their villages were equipped with health facilities, when compared to 57 percent of women from villages where a health facility is not available. Only 5 percent of the women felt that it was not customary to deliver in health institutions. Other factors contributing for not going to health institutions for delivery were, ‘better care at home’ (16 percent), ‘it costs too much’ (8 percent), ‘no transportation’ or

‘health facility is too far’ (2 percent), ‘no time to go’ (4 percent) and ‘family did not allow’ (2 percent). About two percent reported lack of knowledge regarding the delivery facilities. A few of the women (less than1 percent) did not opt for institutional delivery due to poor quality of services. The corresponding figures were same in rural and urban areas. It is also needed to mention here that 3 percent of women from villages with a health facility reported lack of knowledge as a reason for not having delivery at home.

Table 4.11 REASONS FOR NOT GOING TO HEALTH INSTITUTIONS FOR DELIVERY

Percent distribution of women who had given last live/still birth at home during three years preceding the survey by the main reason for not going to health institution for delivery, according to residence and availability of health facility in the village, Haryana, 2002-04

Reason	Total	Residence		Availability of health facility[1] in the village	
		Rural	Urban	No	Yes
Not Necessary	60.1	58.7	66.2	57.0	59.8
Not customary	5.3	5.8	3.0	7.3	4.9
Cost too much	8.4	8.7	6.9	9.4	8.3
Health facility too far/ No transport	1.9	2.0	1.6	2.2	1.9
Poor quality service	0.3	0.2	0.7	0.2	0.2
No time to go	4.0	3.9	4.4	3.4	4.1
Family did not allow	1.7	1.8	1.2	2.3	1.5
Better care at home	15.7	16.1	13.9	16.0	16.2
Lack of knowledge	2.2	2.3	1.8	2.0	2.5
Other	0.4	0.4	0.3	0.2	0.6
Total percent	100.0	100.0	100.0	100.0	100.0
**Number of women**	4,327	3,544	783	1,290	2,254

Note: [1] Includes sub-centre, primary health centre, community health centre or referral hospital, Government hospital, and government dispensary within the village.

4.10 Delivery Characteristics by District

Table 4.12 shows the delivery characteristics by district; institutional delivery (delivery in government or private health institutions), home delivery and attendant assistance during home delivery for last live/still births to women during the three years preceding the survey. The proportion of institutional delivery is lowest in Gurgaon (20 percent) and followed by Jind (27 percent) and it is highest in Ambala (62 percent).

Table 4.12 DELIVERY CHARACTERISTICS BY DISTRICT

Place of delivery, assistance during home deliveries, and percentage of safe deliveries by district, Haryana, 2002-04

Districts	Percentage of women who had institutional delivery	Percentage of women who had delivery at home	Home delivery assisted by skilled[1] persons	Percentage of safe[2] delivery
Ambala	61.8	38.2	19.1	69.1
Bhiwani	36.3	63.7	7.5	41.1
Faridabad	31.2	68.8	10.5	38.5
Fatehabad	37.9	62.1	21.7	51.4
Gurgaon	20.4	79.6	3.4	23.1
Hisar	32.4	67.3	11.5	40.1
Jhajjar	46.2	53.8	17.1	55.4
Jind	27.1	72.9	11.4	35.4
Kaithal	28.4	71.6	17.2	40.8
Karnal	33.8	66.2	10.2	40.5
Kurukshetra	50.3	49.7	17.7	59.1
Mahendragarh	36.8	63.2	18.9	48.7
Panchkula	45.1	54.9	15.3	53.5
Panipat	33.9	66.1	18.5	46.1
Rewari	45.9	54.1	20.0	56.7
Rohtak	41.9	57.9	9.4	47.3
Sirsa	33.3	66.7	21.7	47.7
Sonipat	41.1	58.9	15.2	50.0
Yamunanagar	31.6	68.4	10.3	38.7
Haryana	35.1	64.8	12.5	43.2

Note: *Table includes last live/still birth since 1-1-1999/1-1-2001.

[1] Includes Doctor/ANM/Nurse. [2] Either institutional delivery or home delivery assisted by skilled person.

Compared to delivery in a government health facility, deliveries in a private health facility are more common in Haryana. A little more than one-third (35 percent) of births are institutional delivery in the state, but in all the districts, except Ambala, more than half of the births took place at home. The extent of safe deliveries also varies by district, in 8 of 19 districts, the proportion of safe deliveries are below state average, it ranges from 23 percent in Gurgaon to 69 percent in Ambala. The proportion of safe deliveries is less than 40 percent in four districts i.e. Faridabad, Gurgaon, Jind and Yamunanagar (see Map-4).

4.11 Complications During Delivery

Complications during delivery include ‘premature labour’, ‘obstructed labour’, ‘prolonged labour (more than 12 hours)’, ‘breech presentations’, ‘excessive bleeding during delivery’ and ‘other problems’ at the time of delivery reported by women during the three years preceding the survey. One-fifth of the women experienced at least one problem during delivery (Table 4.13 and Figure 4.6). The proportion of delivery complications is higher among urban women (22 percent) than rural among women (20 percent). Younger women below the age of 20 years, and women with low parity 1-2 reported more at least one delivery related problem than older women aged 35 years and above and women with higher parity. This proportion is relatively high among women who had received some kind of antenatal care during their pregnancy. Fifteen percent of women who had not had any antenatal check-up reported that they experienced at least one problem during their pregnancy when compared to 16-27 percent of women who had received some kind of antenatal check-up. Among women who

had assisted or caesarean delivery, 66-76 percent reported experiencing such problems and only 15 percent women with normal deliveries cited complications during delivery. A higher proportion of women who delivered in health institutions (34-35 percent) faced at least one delivery complication compared to those who delivered at home (12 percent).

Table 4.13 DELIVERY COMPLICATIONS

Percentage of women who had given last live/still births during three years preceding the survey by delivery complication, according to selected background characteristics, Haryana, 2002-04

Background characteristics	Any delivery complica-tion	Type of delivery complication;						Number of women
		Pre-mature labour	Excessive bleeding	Pro-longed labour	Obstructed labour	Breech presnta-tion	Other	
**Age group (in years)**								
Below 20	23.3	5.8	4.3	11.3	8.0	2.4	0.8	599
20-34	20.0	3.8	3.5	9.9	5.2	3.2	1.3	5,713
35 and above	17.9	1.7	1.8	13.6	4.9	5.3	0.0	361
**Children ever born**								
1	27.2	5.4	4.5	12.3	8.7	4.7	1.3	1,921
2	18.5	4.3	3.2	8.7	4.7	2.5	1.4	1,961
3	16.7	2.7	2.9	8.9	4.6	2.9	1.2	1,247
4+	15.7	1.9	3.0	10.2	2.8	2.2	0.8	1,516
**Residence**								
Rural	19.5	3.4	3.8	10.6	5.0	3.0	1.3	4,877
Urban	22.1	5.2	2.8	9.1	6.7	3.9	1.0	1,797
**Number of antenatal check-ups**								
No check-up	15.4	1.9	2.7	11.1	3.7	1.6	0.8	819
1	20.6	4.6	3.5	9.9	7.5	3.9	1.1	490
2	16.2	3.2	2.9	9.0	3.9	2.2	0.6	2,124
3	18.1	3.1	3.3	10.6	4.9	2.4	1.1	1,227
4+	27.4	5.6	4.7	11.0	7.6	5.3	2.1	2,008
**Delivery characteristics**								
Normal	14.6	3.4	2.9	8.5	2.6	1.5	0.4	6,036
Caesarean	76.4	7.8	8.9	28.6	32.0	23.0	10.3	447
Assisted	65.6	8.4	11.1	22.2	33.4	10.8	4.0	190
Place of delivery								
Government sector	33.5	6.9	4.9	16.4	11.7	6.0	2.6	705
Private sector	35.2	8.2	5.3	13.4	11.5	7.2	2.8	1,640
Home	12.3	1.7	2.6	7.9	2.1	1.2	0.4	4,327
Total	20.2	3.9	3.5	10.2	5.4	3.2	1.2	6,674

Note: Total includes 29 women with zero parity, 5 with missing information on number of ANC visits and 2 on place of delivery who were not shown separately. Total includes one woman in other place of delivery who is not shown separately.

The major problems reported were 'prolonged labour' (10 percent), 'obstructed labour' (5 percent), 'premature labour' and 'excessive bleeding (4 percent each). Only 3 percent reported 'breech presentation', and one percent reported 'other' problems related to delivery. Premature labour, prolonged labour, obstructed labour and breech presentation are more common among younger women and women with low parity. Urban women were more likely to report delivery complications such as premature labour, obstructed labour and breech presentations; whereas prolonged labour and excessive bleeding are more prevalent among rural women. Prolonged labour, obstructed labour and other health problems related to

delivery were more among women whose last delivery was assisted with instruments and breech presentation was more likely among those who had a caesarean than by women with normal delivery during the three years preceding the survey. Women whose recent delivery was performed in medical institutions were more likely to report premature labour, excessive bleeding, prolonged labour, breech presentation and obstructed labour compared with place of delivery other than medical institutions.

Figure 4.6
Percentage of Women with Delivery Complication and By Symptoms

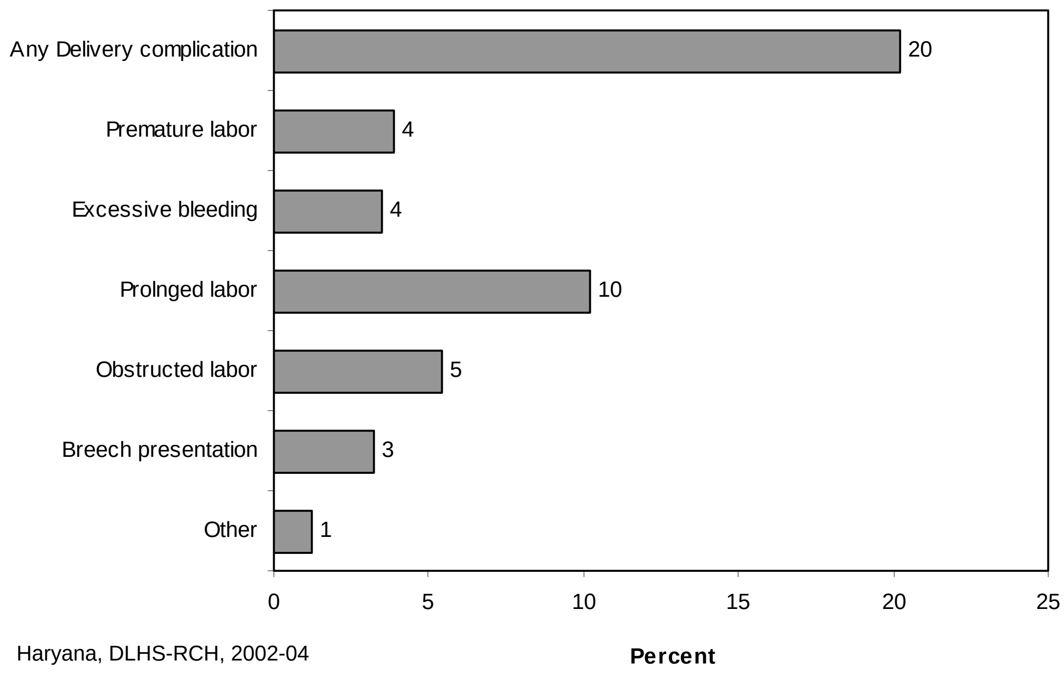

Haryana, DLHS-RCH, 2002-04

4.12 Post Delivery Complications and Treatment

Table 4.14 and Figure 4.7 present information about women who faced complications after delivery according to some selected background characteristics. The incidence of post delivery complications judged by any of the following during the first six-weeks of delivery- ‘high fever’, ‘lower abdominal pain’, ‘foul smelling vaginal discharge’, ‘excessive bleeding’, ‘convulsion’, ‘severe headache’, and ‘other’ problems. Twenty four percent of women reported that they faced any of the problems during the first six weeks after their delivery. The proportion of women who cited at least one post delivery complication is higher in rural areas (26 percent) than in urban areas (20 percent). Younger women below 20 years and women with higher parity 4 and over are more prone to report at least one post delivery related problem.

Table 4.14 POST DELIVERY COMPLICATIONS									
Percentage of women who had given last live/still births during three years preceding the survey by post delivery complication, according to selected background characteristics, Haryana, 2002-04									
Background characteristics	Any post delivery complica-tion	Type of post delivery complication;							Number of women
		High fever	Lower abdo-minal pain	Foul smelling vaginal discharge	Ex-cessive bleeding	Con-vulsion	Severe head-ache	Other	
**Age**									
Below 20	26.3	11.7	12.1	4.9	4.6	3.0	9.9	1.8	599
20-34	23.7	9.9	12.2	4.3	3.2	2.5	9.9	1.4	5,713
35 and above	24.9	13.5	12.2	5.4	3.6	3.0	13.2	1.2	361
**Children ever born**									
1	22.6	9.0	10.9	3.9	3.7	2.0	7.4	1.7	1,921
2	21.5	8.6	10.6	4.0	3.2	2.2	9.2	1.4	1,961
3	26.3	11.9	14.9	5.1	3.0	3.1	11.1	1.4	1,247
4+	26.6	12.6	13.4	5.0	3.2	3.3	13.6	1.2	1,516
**Residence**									
Rural	25.6	11.3	12.8	4.9	3.5	3.0	11.1	1.4	4,877
Urban	19.7	7.5	10.5	3.1	2.9	1.5	7.3	1.5	1,797
**Delivery characteristics**									
Normal	23.3	10.1	11.8	4.2	3.0	2.4	10.0	1.4	6,036
Caesarean	31.0	11.7	17.3	6.4	5.5	4.5	11.6	2.2	447
Assisted	29.8	12.9	13.6	7.3	9.0	3.9	10.2	1.3	190
**Place of delivery**									
Government sector	22.7	8.3	12.0	5.0	5.3	2.3	9.1	0.9	705
Private sector	23.4	8.7	10.9	4.0	3.9	2.6	8.7	1.8	1,640
Home	24.5	11.2	12.7	4.5	2.8	2.6	10.8	1.4	4,327
**Assistance during home delivery**									
Doctor	26.0	15.0	13.7	5.0	4.8	5.4	8.1	5.8	98
ANM/Nurse/LHV	23.9	11.2	12.7	5.3	2.4	3.0	6.8	2.1	443
TBA	24.2	8.9	12.5	5.4	3.4	2.7	10.9	2.2	1,076
Untrained *dai*	24.4	12.0	12.6	3.7	2.3	2.4	11.4	0.9	2,584
Relative/friends	28.2	12.6	16.1	10.0	9.2	2.8	13.1	0.0	125
Total	24.0	10.3	12.2	4.4	3.3	2.6	10.1	1.5	6,674

Note: Total includes 29 women with zero parity and 2 on place of delivery who were not shown separately. Total includes one woman in other place of delivery and one assisted by none during home delivery who were not shown separately.

Women reported lower abdominal pain (12 percent), high fever and severe headache (10 percent each), foul smelling vaginal discharge (4 percent), excessive vaginal bleeding and convulsion (3 percent each). Less than two percent of women reported other problems. Rural-urban differences in some of the symptoms of postpartum complication are large. All the postpartum complications, except excessive vaginal bleeding, are more prevalent among women aged 35 years and above than among women below 35 years. The symptoms of postpartum complications were increasing steadily with increased parity. There are minimal differences in the likelihood of having different symptoms in the postpartum period by place of delivery. Women who had the last delivery at home were more likely to have high fever, lower abdominal pain, convulsion, severe headache and other postpartum problems during the first six weeks of delivery.

Figure 4.7
Percentage of Women with Post Delivery Complication and By Symptoms

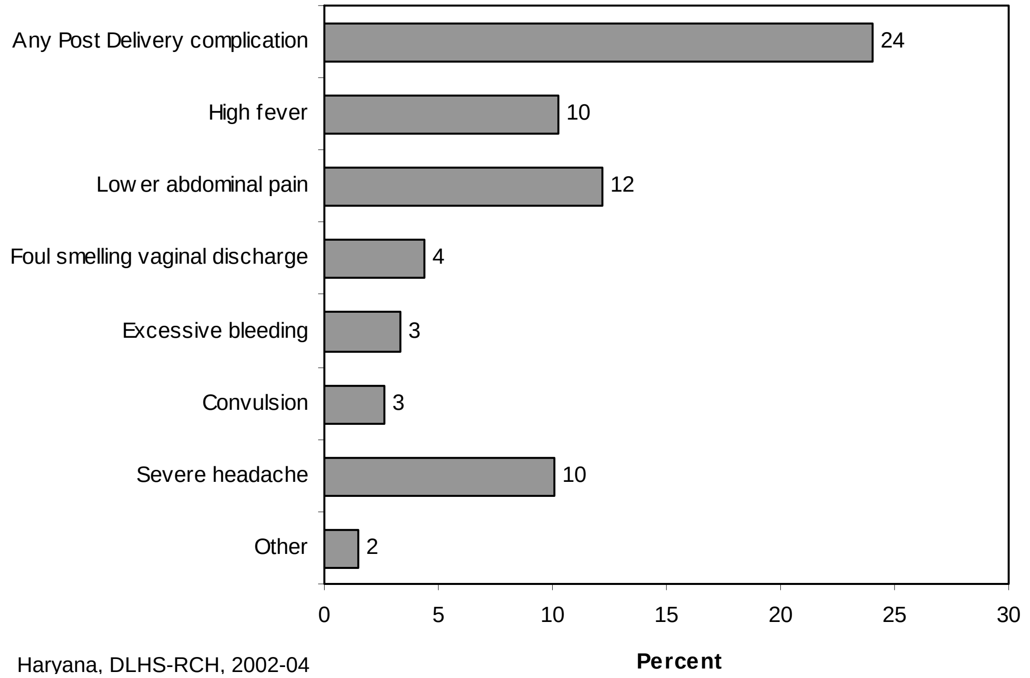

Women who reported at least one complication during the postpartum period were asked, whether they had consulted or sought treatment for their problems and also the source of treatment. Table 4.15 shows the percentage of women who had post delivery complications and who sought treatment by source of treatment according to residence and availability of health facility in the village. More than half of the women (54 percent) reported that they had obtained advice or had consulted someone for their problems. The proportions of urban and rural women were 61 percent and 52 percent respectively and 53 percent of women sought treatment from those villages where health facility was available as compared to 50 percent of women who did not have a health facility within the village.

Table 4.15 TREATMENT FOR POST DELIVERY COMPLICATIONS

Percentage of women who had last live/still births during three years preceding the survey and who had any post delivery complication, sought treatment for the problems, and source of treatment according to residence and availability of health facility in the village, Haryana, 2002-04

Treatment and source	Total	Residence		Availability of health facility[5] in the village	
		Rural	Urban	No	Yes
Percentage of women sought treatment who had any post delivery complication	54.0	52.1	60.5	50.0	53.3
Number of women	865	650	214	217	433
Percentage sought treatment at health facility					
Government health facility[1]	19.7	20.3	18.1	17.7	21.5
Primary health centre	2.8	3.5	0.9	1.5	4.5
Sub centre	1.4	1.9	0.0	2.2	1.7
Private health facility[2]	66.5	66.1	67.7	67.6	65.3
ISM[3] facility	6.8	5.7	10.0	5.6	5.7
Other	7.9	8.9	5.2	10.5	8.0
Percent distribution of women who obtained treatment from					
Doctor	82.7	82.6	83.2	82.6	82.6
ANM/nurse/midwife/LHV	9.7	9.6	9.8	8.7	10.1
Other health professionals[4]	1.8	1.9	1.5	3.6	1.1
Other	3.6	4.0	2.2	4.0	4.1
Missing	2.2	1.8	3.3	1.1	2.2
Total percent	100.0	100.0	100.0	100.0	100.0
Number of women	865	650	214	217	433

Note: [1]Include municipal hospital, dispensary, urban health centre/urban health post/urban family welfare centre, community health centre/rural hospital, primary health centre and sub centre.
[2]Include private hospital/clinic and non-governmental organization/ trust hospital.
[3]Either government or private Indian system of medicine.
[4]Other health professionals include Dai (trained or untrained), relative/friends and ISM practitioner.
[5]Includes sub-centre, primary health centre, community health centre or referral hospital, government hospital and government dispensary within the village.

Among women who sought treatment for complications in the postpartum period, only 20 percent visited a government health facility along with primary health centre (3 percent) and sub-centre (1 percent). More than two-third of the women visited a private health facility and only 7 percent went to a facility with the Indian system of medicine (either government or private) and other health facilities. The proportion of women who visited a government health facility is almost equal in rural and urban areas. Among women who sought treatment, 83 percent preferred to go to a doctor and 10 percent visited an auxiliary nurse midwife or nurse or LHV, 2 percent went to other health professionals and 4 percent went to some one else. Eighty percent of these women in rural and urban areas went to a doctor, whereas a visit to an ANM/nurse/LHV was 10 percent each in rural and urban areas. There are no differences by availability of health facilities and non-availability of health facilities in the village.

4.13 Obstetric Morbidity by District

The extent of health problems/ complications women suffer during pregnancy, delivery and post delivery period indicates the state of obstetric morbidity. Table 4.16 presents the incidence of pregnancy, delivery and post-delivery complications and treatment seeking behaviour in case of pregnancy and post delivery complications by district. As mentioned earlier, in the state, 31 percent 20 and 24 percent of the women experienced pregnancy, delivery and post-delivery complications respectively. About 49 percent of the women sought treatment for pregnancy complications and 54 percent for post delivery complications. In every district, a minimum of one-sixth of the women experienced at least one of the symptoms of pregnancy complications.

Table 4.16 PREGNANCY, DELIVERY AND POST DELIVERY COMPLICATIONS

Extent of pregnancy, delivery and post delivery complications and treatment seeking behaviour by districts, Haryana, 2002-04

	Percentage of women[1]				
	Who had complication during pregnancy	Sought [2] treatment for pregnancy complication	Who had delivery complication	Who had post delivery complication	Sought [3] treatment for post delivery complication
Ambala	26.3	73.1	22.5	15.3	84.0
Bhiwani	25.9	39.1	15.6	17.4	52.6
Faridabad	23.3	49.4	11.9	14.5	57.9
Fatehabad	33.5	52.7	11.8	21.0	70.7
Gurgaon	35.3	34.4	29.8	42.3	37.6
Hisar	17.8	54.5	9.2	11.5	82.2
Jhajjar	40.4	51.9	20.7	26.0	56.4
Jind	20.4	54.5	14.0	15.0	64.1
Kaithal	40.1	52.2	26.2	43.5	42.1
Karnal	48.1	50.2	42.1	45.3	56.7
Kurukshetra	22.5	52.8	18.7	15.2	79.8
Mahendragarh	21.0	50.4	10.3	15.5	38.6
Panchkula	24.0	60.9	22.0	12.8	64.7
Panipat	33.4	44.1	27.0	35.3	45.3
Rewari	43.9	53.8	19.3	17.1	57.2
Rohtak	39.8	53.4	16.2	22.1	62.1
Sirsa	22.7	60.6	15.8	18.4	51.5
Sonipat	43.6	44.5	27.9	31.0	54.2
Yamunanagar	25.4	40.6	16.7	18.5	71.8
Haryana	30.8	48.8	20.2	24.0	54.0

Note: [1] Women who had last live/still birth during three years preceding the survey. [2] Women who reported at least one complication of pregnancy. [3] Women who reported at least one post delivery complication.

In a few districts like Rewari and Sonipat (44 percent each) and Karnal (48 percent), the incidence of pregnancy complications is higher than other districts. The incidence of pregnancy complication is higher than that of delivery and post delivery complications. The percentage of women who experienced at least one type of delivery complication ranges from 9 percent in Hisar to 42 percent in Karnal and incidence of post delivery complication varies from 12 percent in Hisar to 45 percent in Karnal. The incidence of all three types of complications seems to be linked with one another in varying proportions.

In most of the districts of Haryana about half of the women received some kind of antenatal care. In spite of a large proportion of women having contact with a doctor or any other health workers during the antenatal period, in 6 of 19 districts less than 50 percent of the women sought treatment for pregnancy complication. Similarly, among women who experienced at least one symptoms of postpartum complication, the proportion seeking treatment also varies across the districts, ranging from 38 percent in Gurgaon to 84 percent in Ambala.

MAP-3

Percentage of Women Received Three or More Antenatal Checkups

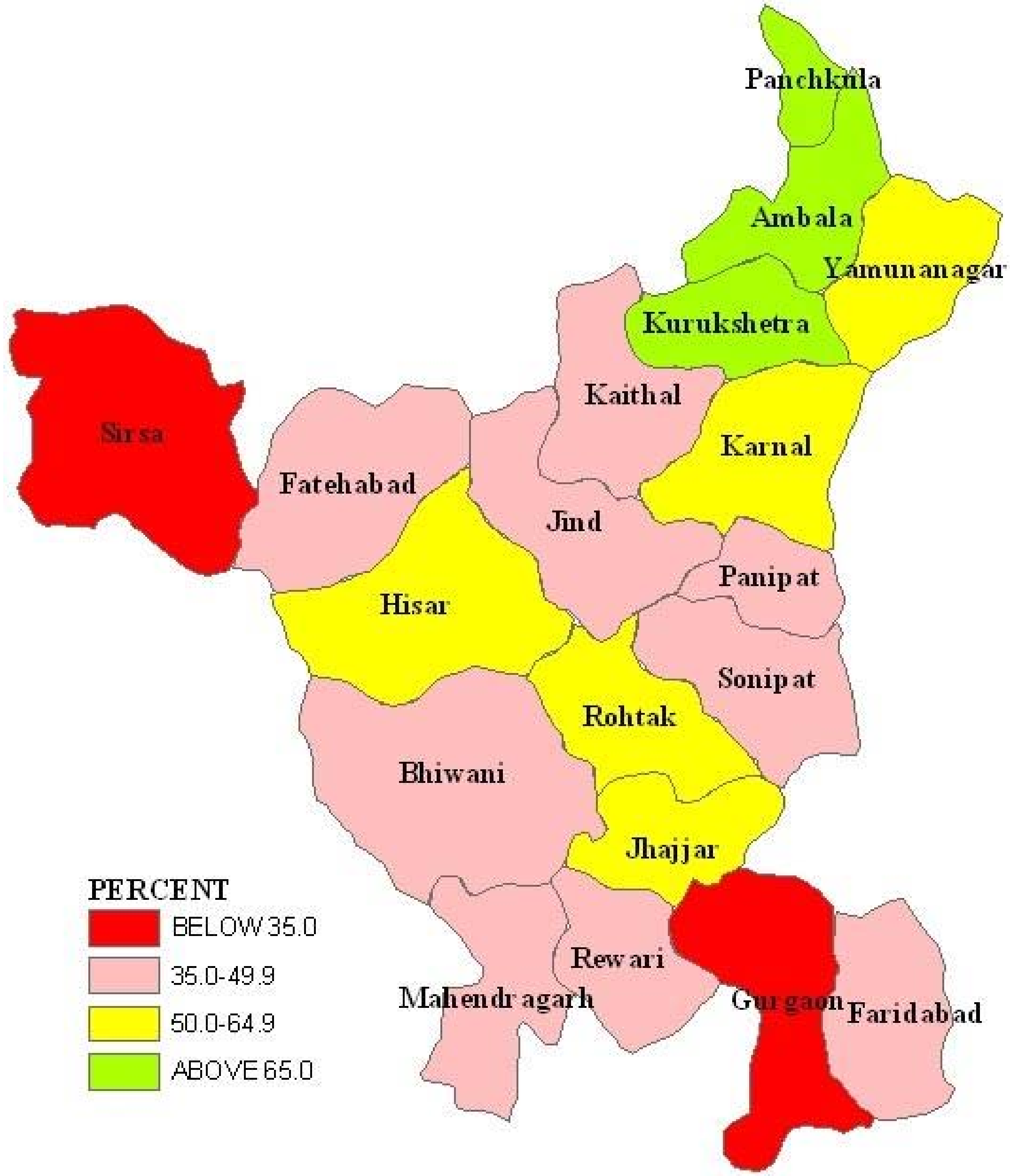

MAP-4

Percentage of Delivery Attended by Skilled Person

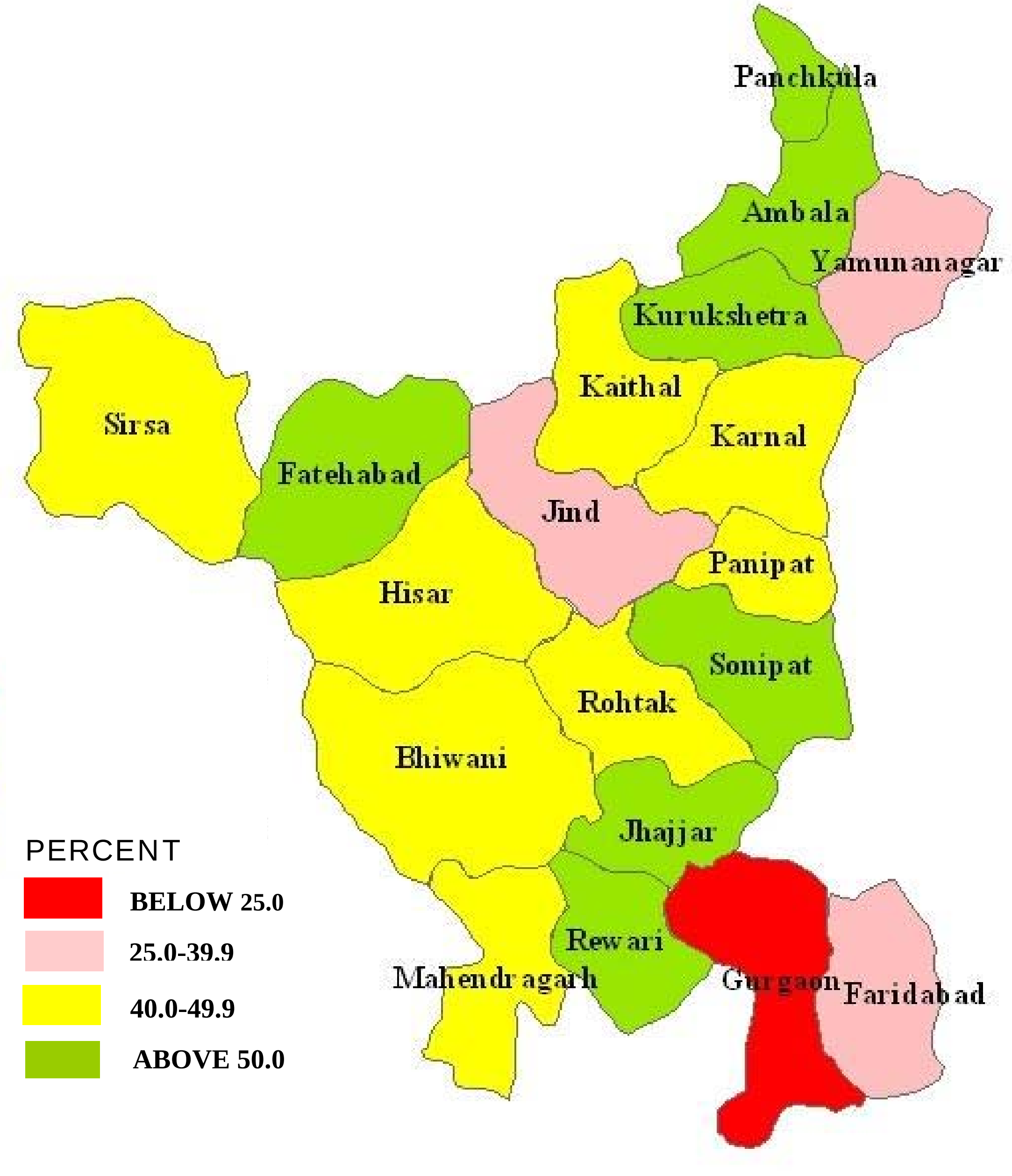

CHAPTER VIII

REPRODUCTIVE HEALTH PROBLEMS AND AWARENESS OF RTIs/STIs AND HIV/AIDS

One of the important components of the Reproductive and Child Health Programme is to have a healthy sexual life without any fear of contracting disease. With this approach the RCH programme places a lot of emphasis on promoting and encouraging healthy sexual behaviour among couples through various Information, Education and Communication (IEC) activities. Health workers are also expected to educate women and men about Reproductive Tract Infections (RTIs) and Sexually Transmitted Infections (STIs) and motivate those people with RTI/STI problems to seek medical help. The DLHS-RCH has made an attempt to collect information on awareness and prevalence of RTI/STI. Apart from this, information on knowledge of HIV/AIDS, source of information and way of avoiding AIDS were also collected.

8.1 Awareness of RTI/STI

An attempt was made to asses whether couples were aware of RTI/STI. Currently married women and their husbands were asked about their awareness of RTI/STI, and if they were aware, they were further questioned about the source of information and mode of transmission of the disease.

Table 8.1 shows the percentage of women aware of RTI/STI by background characteristics. Fifty one percent of the women in Haryana were aware of RTI/STI. The proportion of women who were aware of RTI/STI is much higher in urban areas (57 percent) than in rural areas (48 percent) Figure 8.1. Awareness of RTI/STI is much lower among younger women, non-literate women, women from Muslim religion, scheduled tribe women and women from households with a low standard of living. Awareness of RTI/STI increases from 41 percent among non-literate women to 68 percent among women who have completed 10 or more years of schooling. The standard of living index shows a positive relationship with awareness of RTI/STI, ranging from 36 percent among women with a low standard of living to 61 percent among women with a high standard of living.

Those women who had heard of RTI/STI were further asked about the source of information of RTI/STI, which is presented in Table 8.1. Most of the women (78 percent) reported that they received information of RTI/STI from friends or relatives. Other sources of information of RTI/STI as reported by women were television (21 percent), newspaper or books or magazines (17 percent), slogans or posters or pamphlets or wall hoardings and radio (5 percent each). Only 7 percent of women received this information from doctors and 4 percent from health workers and about 10 percent of the women reported that they had heard of RTI/STI from another source.

Table 8.2 shows the percentage of husbands of currently married women who heard of RTI/STI by specific source of information according to some selected background characteristics. In Haryana, the percentage of men who heard of RTI/STI is higher than that of women (Figure 8.3). Fifty four percent of the men heard of RTI/STI. Men from urban areas and younger men were relatively more aware of RTI/STI. Men who belong to scheduled castes are less likely to report awareness of RTI/STI. The level of awareness of RTI/STI increases with an

increase in education level and standard of living. Twenty five percent of non-literate men were aware of RTI/STI as compared to 72 percent of men who had completed 10 or more years of schooling. Thirty seven percent of men from households with a low standard of living were aware of RTI/STI as compared to 65 percent of men with a high standard of living.

Figure 8.1
Awareness of RTI/STI by Sex According to Residence

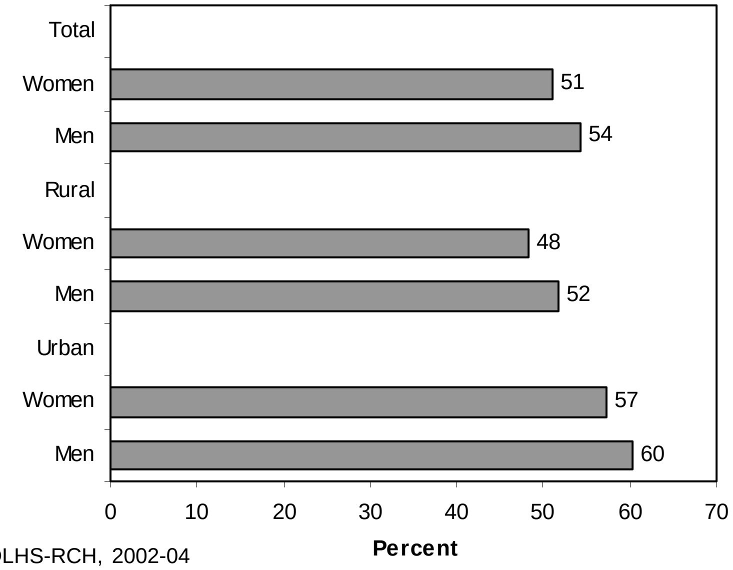

Haryana, DLHS-RCH, 2002-04

Relatives or friends are the most prominent source of RTI/STI for men in Haryana. Sixty one percent of men who knew about RTI/STI received information from relatives or friends. Other important sources of information about RTI/STI are the television (54 percent), followed by newspaper or books or magazines (41 percent), slogans or posters or pamphlets or wall hoardings (29 percent) and radio (18 percent). Twenty three percent of the men received this information from a doctor, 13 percent from community meetings, 6 percent from health workers and 2 percent mentioned that they had received information about RTI/STI from schoolteachers. About 4 percent of the men reported that they heard of RTI/STI from other sources. Relatives or friends are the most important source of information of RTI/STI in all the groups. Men from rural areas, non-literate men, Muslim men, Men from scheduled-caste, men with a low standard of living and younger men are more prone to receive information from relatives or friends. Electronic media such as ‘television’ is also an important source of information of RTI/STI for men who are from urban areas and belong to Hindu religion as well other castes category. The differences in the knowledge of RTI/STI from television as a source of information by educational level and standard of living household are quite visible. Only 30 percent of non-literate men had heard of RTI/STI from television, which increased to 64 percent for men who have completed 10 or more years of schooling.

Table 8.1 SOURCE OF KNOWLEDGE ABOUT RTI/STI AMONG WOMEN

Percentage of currently married women aged 15 - 44 who have heard about RTI/STI and among women who have heard about RTI/STI, percentage who received information from specific sources by selected background characteristics, Haryana, 2002-04.

Background characteristics	Percentage who have heard about RTI/STI	Number of Women	Among those who have heard about RTI/STI, percentage who received information from.										Number of women who have heard about RTI/STI
			Radio	Tele-vision	Newspaper/ Books/ Magazines	Slogan/ Pamphlets/ Posters/ Wall Hoardings	Doctor	Health worker	School teacher	Community Meeting	Relative/ Friends	Others	
**Age group (years)**													
15-19	37.8	1,609	5.7	21.0	16.1	3.1	4.1	1.9	2.5	6.4	79.3	6.9	608
20-24	48.9	4,158	5.1	23.7	18.1	6.7	5.7	3.0	2.8	5.8	75.5	9.4	2,032
25-29	53.6	3,977	4.4	23.9	19.2	5.6	7.4	3.6	2.6	4.9	77.0	10.9	2,130
30-34	52.5	3,568	4.7	20.0	17.7	5.6	8.0	4.3	3.1	7.4	76.6	10.9	1,873
35-39	52.5	2,973	4.4	20.5	16.7	4.2	7.4	4.0	3.3	7.1	79.2	10.7	1,562
40-44	54.7	2,510	3.0	16.0	13.4	3.7	5.3	3.0	3.6	3.9	84.4	10.0	1,374
**Residence**													
Rural	48.3	13,306	3.9	16.7	11.8	3.5	5.8	3.8	3.0	6.0	81.5	10.8	6,430
Urban	57.4	5,489	5.8	30.3	28.3	8.5	8.4	2.9	3.0	5.6	71.4	8.9	3,149
**Education**													
Non-literate	40.5	8,388	1.9	8.5	4.8	0.5	3.7	2.4	3.1	5.6	87.4	11.6	3,400
0-9@ years	52.9	5,794	4.5	18.8	13.1	3.0	5.3	3.1	2.7	6.0	80.7	10.6	3,062
10 and above	67.6	4,604	7.3	37.5	35.0	12.4	11.2	5.0	3.2	6.1	65.6	8.2	3,113
**Religion**													
Hindu	51.8	16,871	4.6	21.6	17.6	5.3	6.8	3.5	3.0	6.3	77.6	9.5	8,744
Muslim	21.9	935	1.0	8.7	5.1	1.0	4.4	2.3	4.5	.9	87.2	26.4	205
Sikh	64.1	922	2.5	18.1	14.7	4.5	5.2	2.6	1.8	1.3	84.8	14.2	591
Other	58.5	67	(20.0)	(40.9)	(43.2)	(18.2)	(11.4)	(6.8)	(2.3)	(9.1)	(65.9)	(9.1)	39
**Caste/tribe[#]**													
Scheduled caste	49.2	4,084	2.7	14.5	9.1	2.4	4.7	3.0	2.2	5.9	83.6	10.4	2,009
Scheduled tribe	43.6	104	(12.2)	(30.6)	(26.5)	(6.1)	(8.2)	(6.1)	(2.0)	(12.2)	(77.6)	(14.3)	45
Other backward class	48.8	5,839	3.7	18.7	13.8	4.4	5.6	3.6	3.8	5.9	80.0	10.7	2,852
Other	53.3	8,764	5.7	25.5	22.7	6.9	8.1	3.6	2.8	5.9	74.7	9.7	4,671
**Standard of living index**													
Low	35.8	3,099	1.7	6.5	4.9	1.7	3.8	2.6	4.4	6.7	85.6	12.2	1,108
Medium	47.9	8,229	3.5	15.3	10.1	2.8	5.0	3.5	2.7	6.3	82.7	10.5	3,943
High	60.6	7,467	6.1	29.9	26.5	8.1	8.7	3.7	2.9	5.3	72.5	9.4	4,527
Total	51.0	18,795	4.5	21.2	17.3	5.2	6.7	3.5	3.0	5.9	78.2	10.2	9,579

Note: Total includes 9 cases missing information on education, and 4 woman do not category in caste
( ) Based on less than 50 unweighted cases.
@ Literate women with no year of schooling are also included.

Table 8.2 SOURCE OF KNOWLEDGE ABOUT RTI/STI AMONG MEN

Percentage of husband of eligible women who have heard about RTI/STI and among men who have heard about RTI/STI, percentage who received information from specific sources by selected background characteristics, State Name, 2002-04.

Background characteristics	Percentage who have heard about RTI/STI	Number of Men	Among those who have heard about RTI/STI, percentage who received information from.										Number of men who have heard about RTI/STI
			Radio	Television	Newspaper / Books/ Magazines	Slogan/ Pamphlets/ Posters/ Wall Hoardings	Doctor	Health worker	School teacher	Community Meeting	Relative/ Friends	Others	
**Age group (years)**													
< 25	52.9	1,556	17.0	57.7	41.6	33.6	20.9	5.5	4.2	11.5	61.2	1.8	823
25-34	57.6	5,034	18.9	55.1	42.5	29.7	22.7	6.1	2.1	12.2	61.4	3.5	2,902
35-44	52.5	4,632	18.5	53.0	39.4	26.0	24.1	6.3	2.1	13.1	58.6	3.6	2,431
45+	50.6	1,978	17.9	49.1	41.6	27.6	21.8	6.5	1.6	12.6	61.8	4.8	1,001
**Residence**													
Rural	51.7	9,387	20.3	49.9	36.2	26.7	21.8	6.6	2.4	13.3	62.3	3.4	4,852
Urban	60.4	3,813	14.4	62.2	51.8	32.7	24.9	5.3	2.0	10.6	56.5	3.6	2,305
**Education**													
Non-literate	25.1	2,667	11.8	29.5	4.5	6.1	16.1	4.2	0.9	15.7	71.3	6.7	669
0-9@ years	48.9	4,759	14.9	42.9	24.1	19.4	20.8	4.9	1.1	11.4	65.8	3.5	2,325
10 and above	72.1	5,773	21.4	64.0	56.6	37.4	25.0	7.2					4,162
**Religion**									3.2	12.5	55.7	3.0	
Hindu	54.7	11,853	18.5	55.0	42.2	29.8	22.8	6.6	2.4	12.7	59.6	3.5	6,487
Muslim	38.8	637	20.9	32.3	30.4	19.8	28.4	1.6	1.3	11.3	77.6	4.4	247
Sikh	58.9	667	14.3	46.8	30.0	14.1	19.1	2.6	0.4	9.8	64.4	3.7	393
Other	(68.1)	43	(23.5)	(73.4)	(71.1)	(47.7)	(22.7)	(0.0)	(0.0)	(9.4)	(47.7)	(3.1)	29
**Caste/tribe**													
Scheduled caste	47.1	2,925	15.5	43.2	28.7	20.6	19.3	5.1	1.5	12.1	63.7	3.9	1,376
Scheduled tribe	53.9	73	(27.2)	(68.7)	(34.0)	(39.3)	(11.4)	(5.3)	(0.0)	(3.7)	(60.9)	(4.6)	40
Other backward class	51.5	4,064	19.2	50.4	36.6	25.0	23.6	6.2	2.5	11.3	62.7	3.9	2,092
Other	59.4	6,137	19.0	59.8	48.7	33.6	23.8	6.6	2.5	13.4	58.0	3.1	3,648
**Standard of living index**													
Low	36.9	2,237	17.7	30.8	22.5	19.0	23.4	5.3	1.6	15.4	71.1	4.5	825
Medium	51.0	5,750	19.8	50.1	34.6	26.1	20.8	6.7	2.2	13.1	62.5	3.4	2,932
High	65.2	5,214	17.4	62.8	51.4	33.1	24.4	5.9	2.5	11.2	56.2	3.4	3,400
Total	54.2	13,200	18.4	53.9	41.2	28.6	22.8	6.2	2.3	12.5	60.5	3.5	7,157

Note: Table includes 30 cases missing information on aware of RTI/STI. Total includes 14 cases with missing information on education and 341 cases in do not know category of caste are not shown separately. @ Literate men with no year of schooling are also included. ( ) Based on less than 50 unweighted cases.

8.1.1 Knowledge of Mode of Transmission of RTI/STI

Women who were aware of RTI/STI were asked about the mode of transmission. This is presented in Table 8.3. Among women who reported knowledge of RTI/STI, 46 percent of them did not know anything further about the mode of transmission of this disease. This proportion is higher among rural women, young women, non-literate women and women from Muslim religion, women from scheduled-tribes and women coming from households with low standard of living. Forty nine percent of rural women do not know about the mode of transmission of RTI/STI compared to 41 percent of urban women. Lack of personnel hygiene and heterosexual intercourse were mentioned by 27 percent and 16 percent of women respectively as mode of transmission of RTI/STI. Only 3 percent of women reported homosexual intercourse and 20 percent reported other modes of transmission of RTI/STI.

Table 8.3 SOURCE OF KNOWLEDGE ABOUT MODE OF TRANSMISSION OF RTI/STI AMONG WOMEN

Percentage of currently married women aged 15-44 who have heard of RTI/STI, knowledge of mode of transmission by selected background characteristics, Haryana, 2002-04

Background characteristic	Percentage by knowledge of mode of transmission				Do not know	Number of women who have heard about RTI/STI
	Homosexual intercourse	Heterosexual intercourse	Lack of personnel hygiene	Other		
**Age**						
15-19	3.6	15.5	25.9	13.5	53.6	608
20-24	2.3	17.4	29.2	18.1	46.1	2,032
25-29	2.8	16.4	28.3	20.5	44.7	2,130
30-34	3.7	17.0	28.4	19.7	45.7	1,873
35-39	2.4	15.1	25.9	23.2	44.9	1,562
40-44	2.7	13.4	21.8	24.3	48.1	1,374
**Residence**						
Rural	2.0	13.1	24.6	20.9	49.1	6,430
Urban	4.6	21.9	31.9	19.3	40.5	3,149
**Education**						
Non-literate	1.6	8.1	17.4	22.2	55.9	3,400
0-9@ years	2.5	12.8	26.1	21.6	47.0	3,062
10 years and above	4.5	27.9	38.3	17.1	35.1	3,113
**Religion**						
Hindu	3.0	16.4	27.5	19.7	46.3	8,744
Muslim	0.0	9.6	9.3	16.4	69.3	205
Sikh	1.3	12.8	25.7	30.4	39.7	591
Other	(11.8)	(22.2)	(39.6)	(26.3)	(20.0)	39
**Caste/tribe**						
Scheduled caste	1.7	11.8	22.3	21.9	50.4	2,009
Scheduled tribe	(1.8)	(26.1)	(29.6)	(7.5)	(47.7)	45
Other backward class	2.4	14.4	25.6	21.0	48.2	2,852
Other	3.7	18.7	29.9	19.5	43.3	4,671
Standard of living index						
Low	1.2	8.2	18.3	16.0	61.7	1,108
Medium	2.4	12.3	22.8	21.2	50.6	3,943
High	3.6	21.2	32.9	20.7	38.7	4,527
Total	2.9	16.0	27.0	20.4	46.3	9,579

Note: Total includes 3 cases missing information on education and 1 woman with do not category in caste. @ Literate women with no year of schooling are also included. ( ) Base on less than 50 unweighted cases.

Table 8.4 presents the knowledge of mode of transmission of RTI/STI among men. Among men who had heard of RTI/STI, 12 percent of them mentioned that they did not know any thing about the mode of transmission of this disease. The percentage of men who did not know about the mode of transmission is higher among non-literate men, Muslim men, men from scheduled tribes and men from households with a low standard of living. Among the men who new the modes of transmission of RTI/STI, 68 percent mentioned heterosexual intercourse, 36 percent reported lack of personnel hygiene and only 5 percent mentioned homosexual intercourse and 14 percent reported other modes of transmission.

Table 8.4 SOURCE OF KNOWLEDGE ABOUT MODE OF TRANSMISSION OF RTI/STI AMONG MEN

Percentage of husbands of currently married women who have heard of RTI/STI , knowledge of mode of transmission by selected background characteristics, Haryana, 2002-04

Background characteristic	Percentage by knowledge of mode of transmission				Do not know	Number of men who have heard about RTI/STI
	Homosexual intercourse	Heterosexual intercourse	Lack of personnel hygiene	Other		
**Age**						
<25	4.5	67.1	33.0	12.7	14.1	823
25-34	4.3	68.6	35.7	14.4	11.4	2,902
35-44	4.3	67.9	36.1	14.0	12.1	2,431
45+	6.6	63.8	38.8	16.8	11.0	1,001
**Residence**	4.8	65.2	33.3	15.6	13.3	4,852
Rural	4.4	72.3	41.4	11.9	8.9	2,305
Urban						
**Education**						
Non-literate	3.8	45.8	22.1	19.3	25.9	669
0-9@ years	3.5	62.5	29.7	15.4	15.6	2,325
10 years and above	5.5	73.8	41.6	13.0	7.6	4,162
**Religion**						
Hindu	4.9	68.4	36.3	14.0	11.4	6,487
Muslim	2.2	56.5	17.4	15.9	27.7	247
Sikh	3.0	59.7	40.9	20.0	10.4	393
Other	(2.4)	(75.4)	(34.6)	(21.2)	(2.7)	29
**Caste/tribe**						
Scheduled caste	4.4	59.8	32.8	18.1	14.6	1,376
Scheduled tribe	(5.5)	(75.2)	(18.6)	(4.7)	(16.5)	40
Other backward class	3.9	66.3	33.2	15.0	12.5	2,092
Other	5.2	71.0	38.9	12.8	10.5	3,648
Standard of living index						
Low	3.8	57.3	25.7	14.3	21.2	825
Medium	4.5	65.4	33.5	15.6	13.3	2,932
High	5.0	71.9	40.5	13.4	8.4	3,400
Total	4.7	67.5	35.9	14.4	11.9	7,157

Note: Total includes 1 case with missing information on education and 1 men with do not know in caste are not shown separately. @ Literate men with no years of schooling are also included. ( ) Base on less than 50 unweighted cases.

8.2 Prevalence of RTI/STI

In DLHS-RCH, information was collected on the common symptoms of reproductive tract infections and sexually transmitted infections from women and their husbands, and information on menstruation related problems in the three months immediately preceding the survey.

The prevalence of reproductive tract infections and sexually transmitted tract infections is judged by their symptoms. All the respondents were told about symptoms of RTI/STI, and were asked whether they had any of them. In case of the presence of at least one symptom, they were further asked whether they sought treatment for such problems, and if they had sought treatment, details regarding the source of treatment also recorded. The topic of RTI/STI is quite sensitive. The culture of silence prevents people from discussing such topics in front of others. In spite of intensive training of the investigators, the respondent might have hesitated in reporting the symptoms of RTI/STI. What gets reported in the survey though may not have given the exact prevalence, but may have given the lower limit for it.

Table 8.5 and Figure 8.2 show that about one-third of currently married women (31 percent) reported at least one reproductive health problem. The problems reported by women were 'low backache' (14 percent), 'pain in lower abdomen' (9 percent), 'some mass coming out of vagina' (8 percent), 'itching over vulva' and frequent / painful passage of urine (7 percent) and 'fever' and 'painful sexual intercourse (5 percent each). Very few women reported 'boils/ ulcers/ warts around vulva', 'bleeding after sexual intercourse' and 'swelling in the groin'. The prevalence of reproductive health problems is common among rural and urban women.

Table 8.5 SYMPTOMS OF RTI/STI AMONG WOMEN

Percentage of currently married women aged 15-44 who reported any symptoms RTI/STI and specific symptoms during three months prior to survey, according to residence, Haryana, 2002-04

Symptoms	Total	Residence	
		Rural	Urban
Percentage of women reported any RTI/STI symptoms			
Symptoms	30.9	32.3	27.4
Itching over vulva	7.1	7.5	6.4
Boils/ ulcers/ warts around vulva	2.2	2.3	1.9
Pain in lower abdomen not related to menses	9.1	9.8	7.3
Low backache	13.7	14.3	12.3
Pain during sexual intercourse 1`	4.9	5.4	3.7
Bleeding after sexual intercourse	0.8	0.9	0.6
Swelling in the groin	3.4	3.7	2.7
Frequent / painful passage of urine	6.9	7.5	5.4
Fever	5.0	5.1	4.7
Some mass coming out of vagina	8.0	8.5	6.8
Any involuntary escape of urine while coughing or sneezing	2.4	2.5	2.0
Swelling / lump in breast	0.8	1.0	0.4
Number of women	18,795	13,306	5,489

Figure 8.2
Symptomps of RTI/STI Among Women

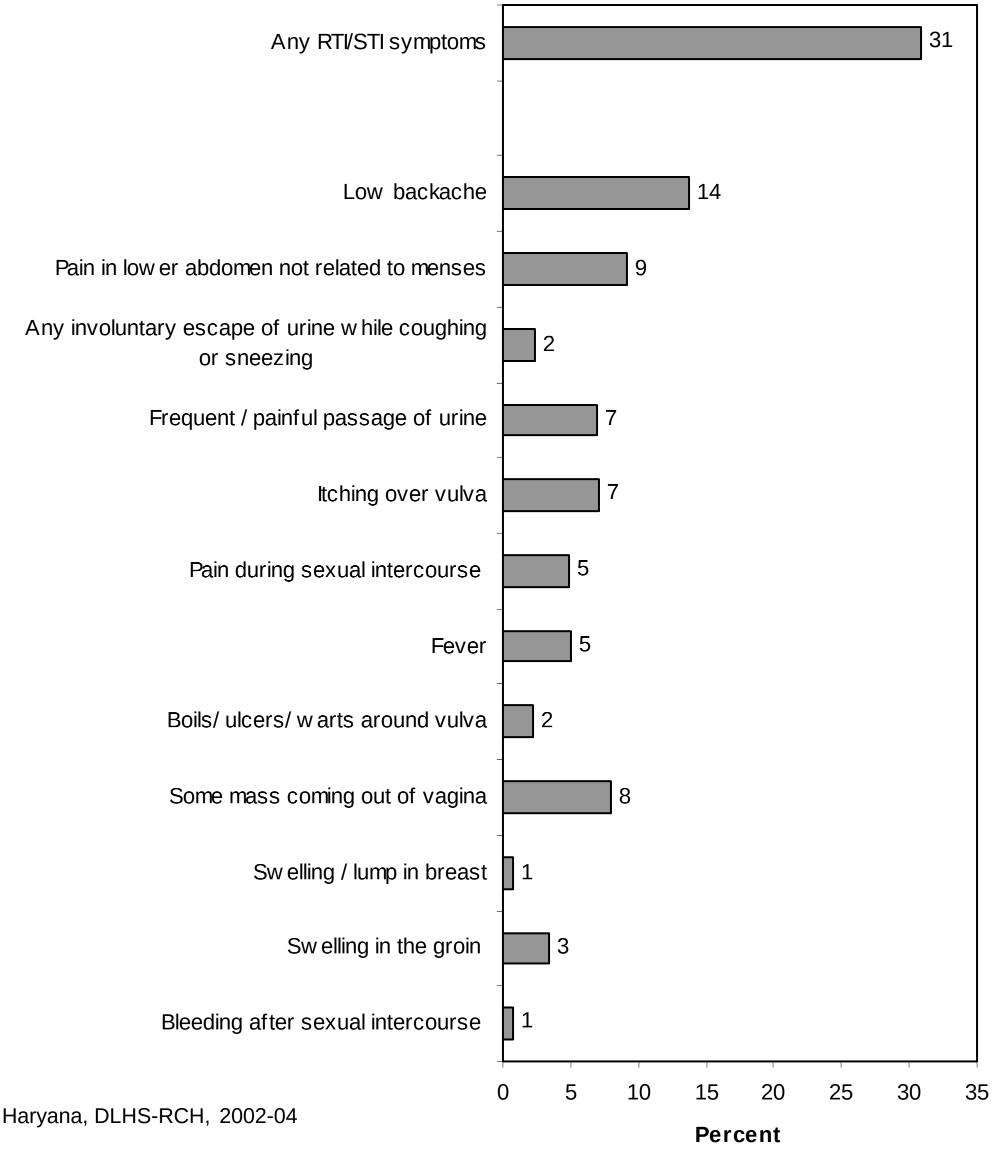

Table 8.6 and Figure 8.3 show the prevalence of reproductive health problems among husbands of currently married women. The prevalence of RTI/STI among men was judged by the reporting of symptoms. Six percent of men reported experiencing at least one symptom of reproductive health problem in the last three months preceding the survey. The prevalence of reproductive health problems is higher among rural men (7 percent) than urban men (5 percent). The specific problem of reproductive health experienced by men is ‘difficulty / pain while urinating or very frequent urination (3 percent), ‘discharge from penis’, and ‘itching / irritation around genital’ and ‘sore / rash / redness on genitals or anal area’ (2 percent each) and ‘sore / rash / redness on genitals or anal area’ and Swelling in the groin (1 percent each).

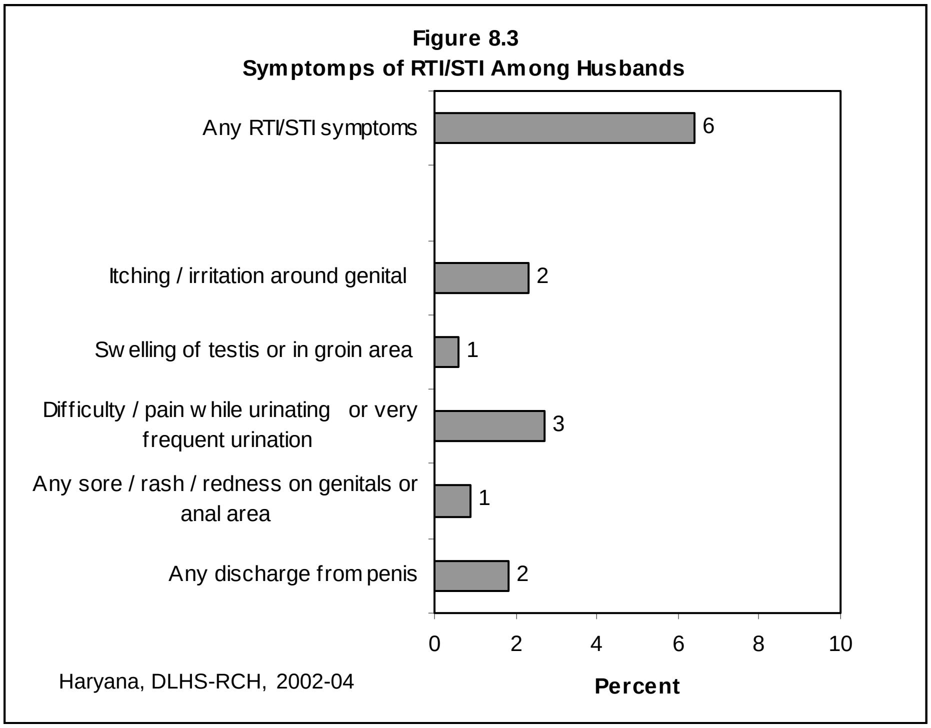

Among men who reported reproductive health problems, 43 percent of them sought treatment. There is not much rural-urban differential in seeking treatment for reproductive health problems. Among them only 22 percent visited a government health facility, including a primary health centre (5 percent) and sub-centre (2 percent) and 47 percent visited a private health facility. Seven percent of men were treated by the Indian system of medicine, 23 percent obtained treatment from a chemist or medical shop and 6 percent of the men reported that they were treated at other sources. A relatively higher proportion of men from rural areas utilised the government health facility and chemist or medical shop for treatment; utilisation of the Indian system of medicine is much higher among urban men (9 percent) than among rural men (6 percent). A large proportion of men saw a doctor (70 percent), 60 percent in urban areas and 72 percent in rural areas. Three percent of men were seen by relative or friends and 2 percent by an ISM practitioner. Four percent of the men used home remedies and 18 percent of the men went to a chemist. Another 5 percent of the men obtained treatment from other sources.

Table 8.6 SYMPTOMS OF RTI/STI AMONG MEN

Percentage of husbands of currently married women who reported any symptoms RTI/STI and specific symptoms during three months prior to survey and sought treatment for RTI/STI by source of treatment, according to residence, Haryana, 2002-04			
Symptoms and treatment	Total	Residence	
		Rural	Urban
Percentage of men reported any RTI/STI symptoms	6.4	6.9	5.0
Symptoms			
Any discharge from penis	1.8	2.0	1.3
Any sore / rash / redness on genitals or anal area	0.9	1.1	0.5
Difficulty / pain while urinating or very frequent urination	2.7	3.1	1.8
Swelling of testis or in groin area	0.6	0.6	0.5
Itching / irritation around genital	2.3	2.3	2.0
Number of Men	13,200	9,387	3,813
Percentage of men sought treatment for any RTI/STI[1]	42.8	43.6	40.0
Number of Men	838	646	192
Percentage sought treatment at health facility[2]			
Government health facility[3]	22.0	22.5	20.2
Primary health centre	4.8	6.1	0.0
Sub centre	2.4	3.0	0.0
Private health facility[4]	46.7	48.9	38.6
ISM[5] facility	6.9	6.3	8.9
Chemist/ medical shop	23.0	20.1	33.7
Other	6.4	7.0	4.2
Percentage obtained treatment from[2]			
Doctor	69.7	72.3	60.2
Male health worker	2.0	2.5	0.0
Traditional healer	1.2	1.6	0.0
Relative/friends	2.8	3.2	1.4
ISM practitioner	2.1	2.1	1.8
Home remedy	3.7	3.7	3.5
Chemist medical shop	18.3	14.3	32.7
Other	4.8	5.7	1.6
Number of men	359	282	77

Note: [1] Based on men with any symptoms of RTI/STI. [2] Percentage may add more than 100.0 due to multiple responses. [3]Includes Government municipal hospital, dispensary, UHC/ UHP /UWFC, CHC/ rural hospital, Primary health centre, sub-centre. [4] Includes private hospital/ clinic, non-governmental / trust hospital/clinic[5]. Either government or private hospital/clinic of Indian system of medicine.

The DLHS-RCH also collected information from currently married women on symptoms of RTIs, that is, on abnormal vaginal discharge, texture, colour and odour of discharge in the three months immediately preceding the survey. The prevalence of reproductive health problems among currently married women is estimated from women’s experiences. Table 8.7 shows the asymptotic prevalence of vaginal discharge related problems among currently married women in Haryana during the three months preceding the survey according to residence. Seventeen percent of the women reported problems related to vaginal discharge. The prevalence of vaginal discharge problem is more among rural (17 percent) than urban women (15 percent).

Table 8.7 ABNORMAL VAGINAL DISCHARGE

Percentage of currently married women aged 15-44 who reported had abnormal vaginal discharge during three months prior to survey and percentage who sought treatment and source of treatment according to residence, Haryana, 2002-04

	Total	Residence	
		Rural	Urban
Percentage of women reported abnormal vaginal discharge	16.7	17.3	15.3
Number of Women	18,795	13,306	5,489
Percentage of women sought treatment for vaginal discharge[1]	37.9	37.1	40.3
Number of Women	3,145	2,307	838
**Percentage sought treatment at health facility** [2]			
Government health facility[3]	27.9	29.6	23.5
Primary health centre	3.7	4.8	0.8
Sub centre	2.5	3.2	0.8
Private health facility[4]	58.4	56.1	64.1
ISM[5] facility	5.9	5.2	7.5
Home remedy	8.3	8.9	6.8
Other	7.5	7.6	7.1
**Percent distribution of women who obtained treatment from** [2]			
Doctor	73.2	71.2	78.1
ANM/nurse/midwife/LHV	13.0	14.5	9.2
Other health professionals[6]	7.9	8.0	7.6
Other	5.7	6.0	4.8
Missing	0.2	0.2	0.2
Total percent	100.0	100.0	100.0
Number of women	1,193	856	337

Note: Total include 6 woman missing information on abnormal vaginal discharge.
[1]Based on women who reported having vaginal discharge. [2] Based on women who sought treatment for vaginal discharge. [3] Includes Government municipal hospital, dispensary, UHC/ UHP /UWFC, CHC/ rural hospital, Primary health centre, sub-centre and out reach/ MCP clinic in village. [4] Includes private hospital/ clinic, non-governmental / trust hospital/clinic, chemist/ medical shop. [5] Either government or private hospital/clinic of Indian system of medicine. [6] Includes *dai* (trained or untrained), relative or friends and chemist/ medical shop.

Among the women who had reported symptoms of vaginal discharge, 38 percent went for treatments, higher percentage (40 percent) from urban areas compared to their rural counterparts (37 percent). A considerable proportion (58 percent) visited private health facilities, followed by government health facility (28 percent). Only 6 percent went to ISM facility, 4 percent to Primary health centre, 3 percent to Sub-centre; while 8 percent took home remedies or visited other places for treatment. The proportion of women who visited a private health facility is higher in urban areas (64 percent) than in rural areas (56 percent), and the proportion of women who visited a facility rendering the Indian system of medicine, is also higher in urban areas (8 percent) than in rural areas (5 percent). A significantly higher proportion (73 percent) of women in the state of Haryana obtained treatment from doctors for their problems. Around 13 percent women were treated by ANM/Nurse/Midwife /LHV and 8 percent by other health professionals.

8.3 Menstruation Related Problems

Table 8.8 shows the percentage of women who had menstruation problems and who sought treatment during the three months preceding the survey. The Table shows that around 16 percent women in Haryana had menstruation problems and the figures are 17 percent and 15 percent in the rural and urban areas respectively.

Table 8.8 MENSTRUATION RELATED PROBLEMS

Percentage of currently married women aged 15-44 who had any menstruation related problem during three months prior to survey and percentage who sought treatment and source of treatment according to residence, Haryana, 2002-04

Symptoms and treatment	Total	Residence	
		Rural	Urban
Percentage of women with any menstruation related problem	16.0	16.6	14.8
Symptoms[1]			
No period	2.5	2.5	2.7
Painful period	40.7	42.6	36.0
Frequent or short period	17.2	17.6	16.1
Delayed period	14.8	14.2	16.5
Prolonged bleeding	9.1	10.0	6.7
Excessive bleeding	20.7	21.6	18.3
Continuous bleeding	5.7	6.4	3.9
Scanty bleeding	23.6	22.2	27.2
Inter-menstrual bleeding	6.2	6.7	5.0
Percentage of women sought treatment who had any menstruation related problems	33.3	31.3	38.8
Percentage sought treatment at health facility[6]			
Government health facility[2]	28.5	29.8	25.8
Primary health centre	(3.2)	(3.9)	(1.9)
Sub centre	(3.0)	(4.1)	(0.7)
Private health facility[3]	62.1	60.2	66.0
ISM[4] facility	9.5	10.0	8.3
Other	(3.4)	(3.7)	(2.6)
Percentage of women obtained treatment from[6]			
Doctor	77.0	74.9	81.3
ANM/nurse/midwife/LHV	19.1	21.0	14.9
Other health professionals[5]	(5.7)	(6.4)	(4.5)
Other	(3.4)	(3.2)	(3.7)
Number of women who are currently menstruating	805	545	260

Note: [1] Based on women who reported any menstruated related problems.
[2] Includes Government municipal hospital, dispensary, UHC/UHP/UWFC, CHC/ rural hospital, Primary health centre, sub-centre and out reach/ MCP clinic in village. [3] Includes private hospital/ clinic, non-governmental / trust hospital/clinic, chemist/ medical shop. [4]Either government or private hospital/clinic of Indian system of medicine, [6] Includes *dai* (trained or untrained), relative or friends and chemist/ medical shop. [6] Multiple responses.
( ) Based on less than 50 cases.
Note: Total includes 2 women missing information on each symptoms and sought treatment for menstruation related problems

Among the women who had reported menstrual problems in Haryana, 41, 24, 21, 17 and 15 percent reported painful periods, scanty bleeding, excessive bleeding, frequent or short period and delayed periods as symptoms respectively. The magnitude of these symptoms is more or less the same among urban as well as rural women. Painful periods and scanty bleeding are the main menstrual problems prevalent in Haryana. Among the women who had menstrual problems, 33 percent sought treatment in the state and the figures for urban and rural areas are 39

percent and 31 percent respectively. The private health facility and government health facility are the main sources of treatment for menstrual problems. Around 62 percent of women sought treatment at private facility and 29 percent sought treatment at government health facility. Ten percent of the women were treated at ISM facility. Most of the women went to a doctor for treatment (77 percent). The figures for urban and rural areas are 81 and 75 percent respectively.

8.4 Prevalence of RTI/STI by District

Table 8.9 presents the prevalence of RTIs/STIs among currently married women and their husbands by districts. The reported symptoms of RTIs/STIs among women are lowest in Ambala and Faridabad (15 percent each) and highest in Karnal (55 percent). The problems related to abnormal vaginal discharge ranges from 6 percent in Fatehabad to 31 percent in Gurgaon.

Table 8.9 REPRODUCTIVE HEALTH CARE INDICATORS BY DISTRICT

Percentage of currently married women and their husbands who reported reproductive health problems and percentage who sought treatment for the problems by district, Haryana, 2002-04

District	Percentage of women			Percentage of men	
	With any symptoms of RTI/STI	Reported any abnormal vaginal discharge	Sought treatment for abnormal vaginal discharge	With any symptoms of RTI/STI	Sought treatment for RTI/STI problems
Ambala	14.8	9.4	43.3	1.7	(44.5)
Bhiwani	30.2	18.8	42.8	5.6	37.7
Faridabad	14.6	16.2	36.4	5.1	26.9
Fatehabad	20.2	6.3	45.7	4.8	52.0
Gurgaon	47.1	31.4	35.7	9.9	42.3
Hisar	21.6	14.4	43.4	1.7	(63.2)
Jhajjar	40.4	22.0	39.7	4.2	31.5
Jind	20.7	14.7	43.1	5.7	50.9
Kaithal	48.8	16.7	37.5	13.5	37.6
Karnal	54.8	25.2	43.6	13.0	54.9
Kurukshetra	29.6	11.0	44.5	1.6	(38.2)
Mahendragarh	21.2	19.2	25.4	3.3	53.2
Panchkula	17.0	13.1	37.1	4.0	41.9
Panipat	41.4	24.6	30.1	9.6	40.7
Rewari	28.6	9.6	43.6	4.8	39.2
Rohtak	41.1	20.5	30.4	4.1	(34.9)
Sirsa	22.1	7.0	39.6	7.7	45.7
Sonipat	40.0	10.3	38.3	8.1	35.4
Yamunanagar	32.3	19.7	40.7	9.3	51.8
Haryana	30.7	16.3	38.1	6.2	43.0

Note: ( ) Based on less number of cases.

In comparison to women, fewer men from all districts of Haryana reported symptoms of RTIs/STIs. Men from Ambala, Hisar and Kurukshetra (2 percent) reported the lowest prevalence of symptoms of RTIs/STIs and men from Kaithal (14 percent) reported the highest prevalence.

The percentage of women who have sought treatment for RTIs (abnormal vaginal discharge) ranges from 25 percent in Mahendragarh to 46 percent in Fatehabad; for men who have sought treatment; it ranges from 32 percent in Jhajjar to 63 percent in Hisar.

8.5 HIV/AIDS

Acquired Immune Deficiency Syndrome (AIDS) is an illness caused by the Human Immunodeficiency Virus (HIV), which weakens the immune system and leads to death through secondary infection such as tuberculosis or pneumonia. The virus is generally transmitted through sexual contact, through the placenta of HIV-infected women to their children, or through contact with contaminated needle (injections) or blood. Prevalence of HIV and AIDS has been on the rise for more than a decade in India and has reached alarming proportions in recent years. To prevent HIV transmission, the government has been making various efforts.

DLHS-RCH has collected information on the general state of awareness of HIV/AIDS, its transmission, its prevention and common misconceptions about HIV/AIDS. All the currently married women in the age group 15-44, and their husbands were first asked if they had ever heard of an illness called HIV/AIDS. Respondents who had heard of HIV/AIDS were further asked about their source of information, mode of transmission, and correct knowledge of HIV/AIDS transfusion.

8.5.1 Knowledge of HIV/AIDS

Table 8.10 shows the percentage of women who had heard about HIV/AIDS by some selected background characteristics. Fifty five percent of currently married women in Haryana have heard of HIV/AIDS, which is lower than RCH Round – I. In Round-I only 76 percent of currently married women were aware of HIV/AIDS.

Knowledge of HIV/AIDS is lower among rural women, non-literate women and Muslim women, women from scheduled castes, women from households with a low standard of living and younger women. Seventy two percent of urban women had heard about HIV/AIDS compared to only 48 percent of rural women. Knowledge of HIV/AIDS steadily increased with increase in educational level and household standard of living. About one-fourth of non-literate women (26 percent) had heard of HIV/AIDS against 94 percent of women who had completed 10 or more years of schooling. Similarly a little less than one-fifth of the women (18 percent) with a low standard of living had heard of HIV/AIDS against 77 percent of women with a high standard of living. About half of the women from different age groups have knowledge of HIV/AIDS. Muslim women (16 percent) were less aware of HIV/AIDS compared to women from Sikh (66 percent), Hindu (56 percent) and ‘other’ religions (76 percent). Women from ‘other caste’ category were more knowledgeable about of HIV/AIDS (65 percent) than women belonging to scheduled tribe and other backward classes (49 percent each) and scheduled-caste women (42 percent).

The government has been using mass media, such as television, radio, and newspaper extensively to increase awareness among the general public about HIV/AIDS and its prevention. Table 8.10 shows the percentage of currently married women who were aware of HIV/AIDS from different sources. The most prominent source of information about HIV/AIDS is television. About 86 percent of women reported that television was their source of information about HIV/AIDS, followed by relatives or friends (36 percent), newspapers, books or magazines (29 percent), slogans or pamphlets, posters or wall hoardings (22 percent) and radio (14 percent). Nine percent of the women reported that a doctor had informed them about HIV/AIDS and 5 percent of the women received information of HIV/AIDS from a health worker.

Table 8.11 shows the percentage of husbands of currently married women who had heard about HIV/AIDS. In Haryana, the proportion of men who had heard about HIV/AIDS is much higher than that of women. Eighty six percent of men had heard of HIV/AIDS as compared to 55 percent of women (Figure 8.4).

Figure 8.4
Awarness of HIV/AIDS by Sex According to Residence

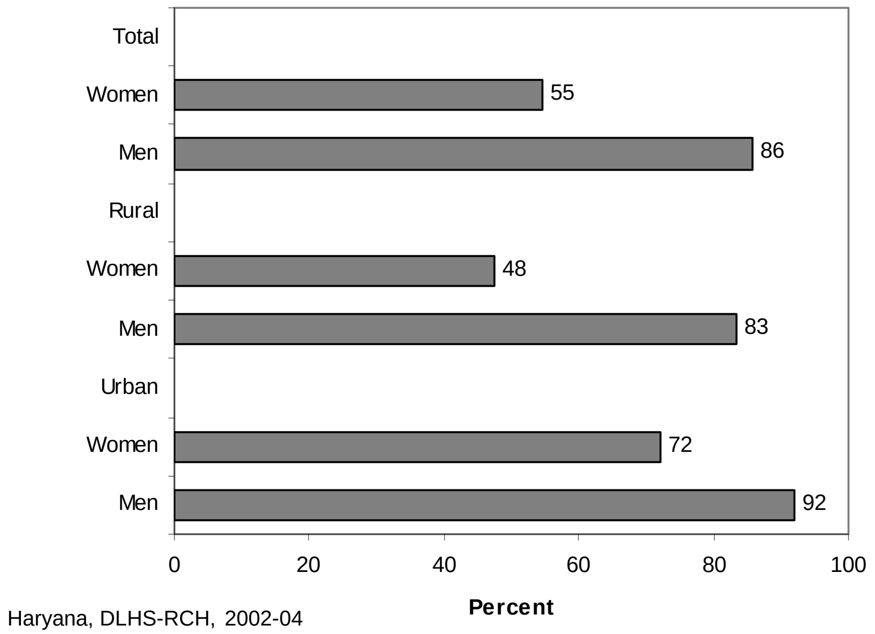

About 92 percent of urban men had heard about HIV/AIDS as compared to 83 percent of rural men. Knowledge of HIV/AIDS varies by men's age and it is higher for the age below 25 years. Awareness of HIV/AIDS is much lower among non-literate men, Muslim men, men from scheduled tribes and men who belong to households with a low standard of living. Less than three-fifth of non-literate men had heard of HIV/AIDS and it increased up to 87 percent for literate men and up to 98 percent of men who had completed 10 or more years of schooling and it is positively related to standard of living.

Table 8.11 also shows the percentage of husbands of currently married women who were aware of HIV/AIDS by different sources. As reported by the men of Haryana, the most prominent source of information of HIV/AIDS was television (79 percent), followed by relatives or friends (54 percent). Other important sources of HIV/AIDS are the radio (25 percent) newspapers, books or magazines (46 percent) and slogans or pamphlets, posters or wall hoardings (43 percent). Twenty one percent of men reported that a doctor had informed them about HIV/AIDS and 6 percent men had received information of HIV/AIDS from a health worker.

Table 8.10 SOURCE OF KNOWLEDGE ABOUT HIV/AIDS AMONG WOMEN

Percentage of currently married women aged 15 - 44 who have heard about HIV/AIDS and among women who have heard about HIV/AIDS, percentage who received information from specific sources by selected background characteristics, Haryana, 2002-04.

Background characteristic	Percentage who have heard about HIV/AIDS	Number of Women	Among those who have heard about HIV/AIDS, percentage who received information from.										Number of women who have heard about HIV/AIDS
			Radio	Television	Newspaper/ Books/ Magazines	Slogan/ Pamphlets/ Posters/ Wall Hoardings	Doctor	Health worker	School teacher	Community Meeting	Relative/ Friends	Others	
**Age group (years)**													
15-19	49.1	1,609	15.5	82.2	26.4	18.6	5.5	3.9	4.2	3.6	33.4	2.3	790
20-24	62.2	4,158	14.4	87.4	27.6	22.0	8.0	4.5	2.5	4.5	35.7	1.6	2,586
25-29	62.3	3,977	14.5	87.4	31.5	24.6	9.5	4.9	1.5	5.7	36.8	1.6	2,478
30-34	52.4	3,568	14.4	85.7	28.9	21.4	9.8	6.2	1.4	7.0	35.8	1.6	1,867
35-39	48.8	2,973	12.0	83.5	26.4	19.7	9.4	4.4	1.2	5.0	38.0	2.0	1,451
40-44	44.3	2,510	12.2	83.9	28.4	21.3	7.9	5.3	2.3	5.5	37.5	0.9	1,112
**Residence**													
Rural	47.6	13,306	14.8	80.7	20.0	16.5	7.4	5.6	1.7	5.8	40.5	1.9	6,327
Urban	72.1	5,489	12.5	93.9	42.3	30.5	10.7	3.9	2.4	4.6	29.7	1.2	3,955
**Education**													
Non-literate	26.2	8,388	9.9	70.1	3.3	2.0	4.0	3.8	0.5	5.6	45.5	2.1	2,198
0-9@ years	64.6	5,794	12.5	84.6	16.5	15.8	6.2	3.7	1.0	5.1	36.2	1.9	3,743
10 and above	94.3	4,604	17.3	94.7	51.9	37.2	13.2	6.6	3.6	5.4	31.8	1.2	4,341
**Religion**													
Hindu	56.2	16,871	14.2	85.5	28.6	21.9	8.7	5.0	2.0	5.5	36.2	1.6	9,481
Muslim	15.6	935	12.0	71.7	16.1	8.6	6.2	0.5	0.5	3.8	43.8	6.2	146
Sikh	65.7	922	8.9	91.6	28.2	22.4	8.0	4.4	1.4	3.2	35.6	0.8	605
Other	76.0	67	25.6	98.3	61.3	51.0	13.1	9.1	9.1	7.3	36.2	0.0	51
**Caste/tribe**													
Scheduled caste	41.9	4,084	12.5	81.8	16.2	12.8	7.4	4.7	1.5	5.1	35.4	2.2	1,712
Scheduled tribe	49.4	104	20.5	86.6	24.4	29.8	20.9	8.1	1.9	1.1	30.9	0.0	52
Other backward class	48.7	5,839	13.0	84.1	24.1	18.5	7.8	5.9	1.5	5.8	38.3	1.8	2,844
Other	64.7	8,764	14.8	87.8	34.6	26.2	9.4	4.5	2.4	5.2	35.6	1.4	5,674
**Standard of living index**													
Low	17.9	3,099	16.5	51.5	10.9	9.9	8.6	5.8	1.3	7.1	53.3	3.3	556
Medium	48.1	8,229	13.1	80.1	15.7	13.4	6.0	4.8	1.8	6.0	38.8	1.6	3,958
High	77.3	7,467	14.3	93.0	39.1	28.8	10.5	5.0	2.2	4.7	33.0	1.5	5,769
Total	54.7	18,795	13.9	85.8	28.6	21.9	8.7	4.9	2.0	5.4	36.3	1.6	10,283

Note: Total includes 9 cases with missing information on education 4 women with do not category in caste are not shown separately. @ Literate women with no year of schooling are also included.

Table 8.11 SOURCE OF KNOWLEDGE ABOUT HIV/AIDS AMONG MEN

Percentage of husband of currently married women who have heard about RTI/STI and among men who have heard about RTI/STI, percentage who received information from specific sources by selected background characteristics, Haryana, 2002-04.

Background characteristic	Percentage who have heard about HIV/AIDS	Number of men	Among those who have heard about HIV/AIDS, percentage who received information from.										Number of men who have heard about HIV/AIDS
			Radio	Televi-sion	Newspaper/ Books/ Magazines	Slogan/ Pamphlets / Posters/ Wall Hoardings	Doctor	Health worker	School teacher	Community Meeting	Relative/ Friends	Others	
**Age group (years)**													
< 25	89.8	1,556	25.1	81.4	45.1	43.3	19.9	5.5	3.9	12.3	56.6	2.4	1,398
25-34	89.4	5,034	25.8	81.6	47.7	44.2	21.5	6.2	2.7	13.2	55.0	2.5	4,500
35-44	83.8	4,632	24.3	78.1	45.6	40.7	21.2	6.4	1.9	13.2	52.1	2.6	3,883
45+	78.3	1,978	24.3	73.4	46.1	42.0	21.2	6.5	2.1	12.7	52.8	1.8	1,548
**Residence**													
Rural	83.3	9,387	27.2	74.8	40.8	39.0	20.2	6.1	2.7	14.4	56.0	2.5	7,817
Urban	92.1	3,813	20.0	89.2	59.0	50.7	23.3	6.4	2.0	9.9	49.3	2.3	3,512
**Education**													
Non-literate	57.0	2,667	16.1	54.9	4.5	7.7	13.1	4.4	0.9	15.3	63.4	1.8	1,519
0-9@ years	87.1	4,759	20.9	73.4	31.5	33.3	17.6	4.8	1.2	12.2	56.2	2.3	4,147
10 and above	98.1	5,773	30.4	90.1	68.6	58.8	26.0	7.8	3.8	13.0	49.7	2.7	5,661
**Religion**													
Hindu	86.8	11,853	25.3	80.2	47.1	43.5	21.3	6.5	2.6	13.3	53.8	2.4	10,289
Muslim	62.6	637	27.1	52.7	33.5	34.2	21.8	3.3	1.3	13.6	59.3	2.1	399
Sikh	90.3	667	18.7	80.9	42.2	32.7	17.7	2.8	1.3	7.8	52.4	3.0	602
Other	(92.0)	43	(18.5)	(81.6)	(66.9)	(56.3)	(26.7)	(11.4)	(7.4)	(12.9)	(63.4)	(1.6)	40
**Caste/tribe**													
Scheduled caste	80.5	2,925	21.9	70.7	32.0	31.3	17.0	5.3	1.6	12.7	57.7	2.2	2,353
Scheduled tribe	75.3	73	30.5	91.9	40.0	46.1	20.3	5.0	3.3	12.3	55.6	7.8	55
Other backward class	82.9	4,064	25.3	77.2	43.1	39.9	21.2	6.2	2.5	12.5	53.9	2.6	3,369
Other	90.4	6,137	26.0	84.0	54.6	49.0	23.0	6.6	2.8	13.5	52.3	2.3	5,550
**Standard of living index**													
Low	65.0	2,237	23.2	48.0	24.4	28.5	19.9	6.5	2.0	17.1	63.3	3.1	1,454
Medium	84.8	5,750	25.4	76.8	37.4	37.0	18.9	5.4	2.1	12.5	55.7	2.4	4,874
High	95.9	5,214	25.1	90.8	61.6	52.1	23.8	7.0	2.9	12.3	49.4	2.2	5,001
Total	85.8	13,200	25.0	79.3	46.4	42.6	21.2	6.2	2.5	13.0	53.9	2.4	11,329

Note: Table includes 6 cases missing information on knowledge of HIV/AIDS. Total includes 14 case missing information on education and 341 cases about do not know in caste category are not shown separately. @ Literate men with no year of schooling are also included. ( ) Based on less than 50 unweighted cases.

About 13 percent reported that they were informed through community meetings and 3 percent received such information from a school teacher. The information on awareness of HIV/AIDS through mass media, such as television and newspapers, and books or magazines, was received more by younger men, urban men, and men from other religions and 'other castes' category, with at least 10 years of schooling, and men from households with a high standard of living.

8.5.2Knowledge of Mode of Transmission about HIV/AIDS

Women who were aware of HIV/AIDS were asked about the mode of transmission and this is presented in Table 8.12. Among women who reported awareness of HIV/AIDS, 14 percent of them did not know about the mode of transmission.

Table 8.12 SOURCE OF KNOWLEDGE ABOUT MODE OF TRANSMISSION OF HIV/AIDS AMONG WOMEN
Percentage currently married women aged 15-44 who have heard of HIV/AIDS , knowledge of mode of transmission by selected background characteristics, Haryana, 2002-04

Background characteristic	Percentage by knowledge of mode of transmission						Do not know	Number of women who have heard of HIV/AIDS
	Homo sexual intercourse	Hetero sexual intercourse	Needles/ blade/ skin puncture	Mother to child	Transfusion of infected blood	Other		
**Age**								
15-19	5.5	76.3	35.3	13.2	24.8	1.4	19.0	790
20-24	7.7	83.4	47.5	17.7	34.6	2.1	11.9	2,586
25-29	7.5	81.8	45.5	17.5	35.5	1.7	13.0	2,478
30-34	9.5	80.4	44.3	16.2	33.8	1.8	13.7	1,867
35-39	7.9	79.0	36.3	13.5	27.8	1.1	16.2	1,451
40-44	7.7	78.7	36.2	12.8	26.3	0.9	16.7	1,112
**Residence**								
Rural	6.2	80.9	39.5	13.8	28.8	1.8	14.7	6,327
Urban	10.4	80.7	47.8	19.4	37.2	1.3	13.3	3,955
**Education**								
Non-literate	5.9	72.4	21.9	5.8	14.0	0.9	22.8	2,198
0-9@ years	6.3	77.9	35.2	11.2	24.6	1.6	16.7	3,743
10 years and above	10.1	87.5	59.7	25.1	47.6	2.0	7.6	4,341
**Religion**								
Hindu	8.0	80.5	42.5	16.0	31.9	1.6	14.3	9,481
Muslim	8.9	71.7	23.5	6.5	19.4	2.0	23.0	146
Sikh	3.9	87.3	48.2	15.7	36.0	1.4	9.3	605
Other	17.5	77.8	60.5	29.5	53.3	2.4	13.9	51
**Caste/tribe**								
Scheduled caste	6.2	79.6	33.8	11.1	25.1	0.7	16.1	1,712
Scheduled tribe	14.7	67.6	41.4	20.0	30.7	0.0	19.3	52
Other backward class	6.6	81.3	41.2	14.1	29.5	2.0	14.0	2,844
Other	8.9	81.0	46.1	18.3	35.5	1.7	13.6	5,674
Standard of living index								
Low	5.2	70.7	22.2	9.7	15.3	1.6	24.3	556
Medium	6.5	79.2	35.3	11.7	25.6	1.4	15.7	3,958
High	9.0	82.8	49.7	19.5	38.1	1.8	12.1	5,769
Total	7.8	80.8	42.7	15.9	32.1	1.6	14.2	10,283

Note: One woman with do not category in caste are not shown separately. @ Literate women with no year of schooling are also included.

This proportion is relatively high among rural women, non-literate women, and Muslim women, women from scheduled tribes and women with a medium standard of living. Fifteen percent of the rural women do not know about the mode of transmission of HIV/AIDS compared to 13 percent of urban women.

Among women who reported different ways of transmission of HIV/AIDS, a large proportion (81 percent) mentioned heterosexual intercourse as a mode of transmission. All the socio-economic groups reported that heterosexual intercourse was the main mode of transmission of HIV/AIDS. Other modes reported by women were transmission through needle or blade or skin puncture (43 percent), transfusion of infected blood (32 percent), mother to child, if pregnancy occurs during a stage of HIV (16 percent); only 8 percent of the women mentioned that homosexual intercourse could also be a mode of transmission. Only two percent stated that there were other ways of transmission of HIV/AIDS.

Table 8.13 SOURCE OF KNOWLEDGE ABOUT MODE OF TRANSMISSION OF HIV/AIDS AMONG MEN

Percentage of husbands of currently married women who have heard of HIV/AIDS , knowledge of mode of transmission by selected background characteristics, Haryana, 2002-04

Background characteristic	Percentage by knowledge of mode of transmission						Do not know	Number of men who have heard of HIV/AIDS
	Homo-sexual intercourse	Hetero-sexual intercourse	Needles/ blade/ skin puncture	Mother to child	Transfusion of infected blood	Other		
**Age**								
<25	5.4	91.2	48.8	9.3	34.5	2.6	5.6	1,398
25-34	5.0	91.4	51.1	11.2	38.1	2.3	5.7	4,500
35-44	4.8	91.4	44.5	9.1	31.9	2.7	6.3	3,883
45+	4.7	93.0	41.9	9.8	32.1	1.9	5.2	1,548
**Residence**								
Rural	5.1	90.7	45.4	8.8	32.0	2.3	6.3	7,817
Urban	4.5	93.7	51.4	13.0	40.7	2.6	4.8	3,512
**Education**								
Non-literate	3.2	81.8	17.1	1.7	8.9	2.0	14.9	1,519
0-9@ years	3.5	90.2	34.8	4.9	22.9	2.3	7.3	4,147
10 years and above	6.4	95.3	64.5	16.1	50.3	2.6	2.3	5,661
**Religion**								
Hindu	5.1	91.9	48.0	10.2	35.3	2.5	5.5	10,289
Muslim	2.4	81.8	27.7	5.2	18.3	2.2	17.0	399
Sikh	3.2	93.3	46.9	10.0	36.0	1.8	4.1	602
Other	(5.5)	(92.0)	(54.4)	(24.3)	(40.9)	(0.0)	(5.3)	40
**Caste/tribe**								
Scheduled caste	4.8	89.6	36.0	7.5	23.9	2.0	7.3	2,353
Scheduled tribe	5.3	86.5	45.6	6.5	23.3	3.5	9.6	55
Other backward class	5.2	90.0	45.8	8.2	32.5	2.9	6.8	3,369
Other	4.8	93.5	53.0	12.4	40.8	2.3	4.6	5,550
**Standard of living index**								
Low								
Medium	4.2	82.6	23.5	3.4	14.9	2.6	13.4	1,454
High	4.5	91.4	42.7	7.5	28.6	2.3	6.2	4,874
	5.5	94.5	58.7	14.5	46.4	2.5	3.2	5,001
Total								
	4.9	91.6	47.3	10.1	34.7	2.4	5.8	11,329

Note: Total includes 5 case missing information on education and 229 cases about do not know in caste category are not shown separately. @ Literate men with no year of schooling are also included. ( ) Based on less than 50 unweighted cases.

Table 8.13 presents the knowledge about mode of transmission of HIV/AIDS among men. Six percent of the men who had heard about HIV/AIDS mentioned that they do not know the mode of transmission. The percentage of men not knowing the mode of transmission is higher among rural men, non-literate men, Muslim men, scheduled tribe and men from households with a low standard of living. Among who reported ways of transmission of HIV/AIDS, 92 percent of them mentioned heterosexual intercourse as a mode of transmission. All the groups reported that heterosexual intercourse was the main mode of transmission of HIV/AIDS. Other modes reported by men are transmission through needle or blade or skin puncture (47 percent), transfusion of infected blood (35 percent), mother to child, if pregnancy occurs during a stage of HIV (10 percent) and only 5 percent of men mentioned that homosexual intercourse could also be a mode of transmission of HIV/AIDS. Only two percent stated that there were other ways of transmission of HIV/AIDS.

8.5.3 How to avoid HIV/AIDS

All the respondents, male and female, were asked about how to prevent HIV/AIDS. The percentage of women who said that HIV/AIDS could be avoided by various ways has been presented in Table 8.14 by some selected background characteristics.

Among women who reported about awareness of HIV/AIDS, about 19 percent of them did not know how to avoid becoming infected by HIV/AIDS. This percentage is higher among rural women than among urban women. The percentage of women who did not know of any way to avoid infection decreases with increasing levels of education and household standard of living. Twenty eight percent of non-literate women reported that they did not know of any way to avoid infection as compared to 11 percent of women who had completed ten or more years of schooling. Similarly, 30 percent of women with low a standard of living stated that they did not know of any way to avoid infection as compared to 16 percent of women with a high standard of living. The percentage of women who did not know ways to avoid infection is also high among Muslim women and scheduled tribe women.

Among women who mentioned ways to avoid HIV/AIDS, most of the women (75 percent) said, “sex with only one partner is the way to avoid it”. Other ways to prevent HIV/AIDS mentioned by women were sterilizing needles and syringe before injecting’ (38 percent), ‘checking blood prior to transfusion’ (34 percent),‘using a condom correctly during each sexual intercourse’ (33 percent) and 10 percent of the women reported that the pregnancy should be avoided if couples were infected by HIV/AIDS. All the specific ways to avoid becoming infected by HIV/AIDS reported by women are proportionally higher in urban areas, among Sikh women, women who belong to ‘other castes’ category, women with a high level of education and women with a high standard of living.

Table 8.15 shows the percentage of men who reported that HIV/AIDS could be avoided by some selected background characteristics. Among men who are aware of HIV/AIDS, 6 percent of them did not know of any method to avoid infection, compared to 19 percent women in the state.

In Haryana a higher proportion of men reported that 'sex with only one partner' is the way to avoid HIV/AIDS, most of the men (89 percent) also reported the same, and this was the most commonly reported way to avoid HIV/AIDS in all the groups.

Table 8.14 KNOWLEDGE ABOUT AVOIDANCE OF HIV/AIDS AMONG WOMEN

Among currently married women aged 15-44 who have heard about HIV/AIDS, the percentage of women reported HIV/AIDS can be avoided in specific ways by selected background characteristics, Haryana, 2002-04

Background characteristic	Percentage reported HIV/AIDS can be avoided by:						Do not know to avoid HIV / AIDS	Number of women
	Sex With Only one partner	Using condoms correctly during each sexual intercourse	Checking blood prior to transfusion	Sterilizing needles and syringes for injection	Avoiding pregnancy when having HIV/AIDS	Other		
**Age**								
15-19	69.9	24.1	27.5	31.3	6.0	1.6	24.4	790
20-24	77.1	35.8	36.9	42.4	10.9	1.5	16.6	2,586
25-29	76.0	37.0	36.3	40.8	11.3	1.5	17.3	2,478
30-34	75.2	34.1	35.1	38.9	11.0	2.4	18.7	1,868
35-39	72.6	27.4	28.8	31.9	7.8	2.3	21.0	1,451
40-44	73.4	28.4	28.7	32.5	8.0	1.3	21.2	1,112
**Residence**								
Rural	74.0	29.1	30.0	34.4	7.6	1.6	19.7	6,327
Urban	76.5	38.9	39.6	43.6	13.6	2.0	17.5	3,955
**Education**								
Non-literate	68.2	14.2	14.8	17.9	2.7	1.0	28.3	2,198
0-9@ years	71.6	26.0	26.3	30.3	5.7	1.7	22.2	3,743
10 years and above	81.2	48.2	49.7	54.8	17.1	2.2	11.2	4,341
**Religion**								
Hindu	74.5	32.8	33.5	37.7	9.9	1.8	19.1	9,481
Muslim	69.7	19.7	20.4	19.5	4.0	2.3	26.6	146
Sikh	82.9	36.8	38.1	45.9	10.3	1.0	12.9	605
Other	76.8	47.3	46.5	52.5	17.1	7.8	17.7	51
**Caste/tribe**								
Scheduled caste	74.3	25.1	26.4	29.3	5.6	2.0	20.5	1,712
Scheduled tribe	70.4	36.2	19.5	31.1	7.6	.0	22.5	52
Other backward class	76.0	30.9	31.7	37.0	8.4	1.5	18.6	2,844
Other	74.6	36.1	37.0	41.2	11.9	1.9	18.5	5,674
**Standard of living index**								
Low	66.6	14.8	17.8	19.7	3.6	1.3	29.6	556
Medium	73.1	25.3	27.1	29.6	6.4	1.5	21.0	3,958
High	77.0	39.8	39.7	45.5	12.9	2.0	16.4	5,769
Total	74.9	32.9	33.7	38.0	9.9	1.8	18.9	10,283

Note: One woman with do not category in caste are not shown separately. @ Literate women with no year of schooling are also included.

Other ways to prevent by HIV/AIDS mentioned by men are 'using a condom correctly during each sexual intercourse' (48 percent), 'sterilizing needles and syringe before injecting' (44 percent), and 'checking blood prior to transfusion' (36 percent). All the specific ways to avoid becoming infected by HIV/AIDS reported by men are proportionally higher in urban areas than in rural areas, and among men who belong to 'other caste' category, men with a high level of education and men with a high standard of living. 'Other caste' category men were more likely to report using a condom correctly during each sexual intercourse.

Table 8.15 KNOWLEDGE ABOUT AVOIDANCE OF HIV/AIDS AMONG MEN

Among husbands of currently married women who have heard about HIV/AIDS, the percentage of men reported HIV/AIDS can be avoided in specific ways by selected background characteristics, Haryana, 2002-04

Background characteristic	Percentage reported HIV/AIDS can be avoided by:						Do not know to avoid HIV / AIDS	Number of men
	Sex with only one partner	Using condoms correctly during each sexual intercourse	Checking blood prior to transfusion	Sterilizing needles and syringes for injection	Avoiding pregnancy when having HIV/AIDS	Other		
**Age**								
<25	87.8	50.3	35.2	46.7	6.0	2.6	6.6	1,398
25-34	88.7	52.3	39.5	47.3	7.5	2.8	5.6	4,500
35-44	88.7	45.1	33.5	41.0	5.9	2.8	6.6	3,883
45+	90.8	39.5	33.4	39.6	6.2	3.5	6.1	1,548
**Residence**								
Rural	88.1	45.5	33.5	42.0	6.0	2.8	6.7	7,817
Urban	90.4	53.0	41.6	48.4	7.8	2.9	5.0	3,512
**Education**								
Non-literate	80.7	21.6	8.7	14.3	1.8	3.3	15.0	1,519
0-9@ years	87.0	38.3	23.2	31.7	3.3	3.2	8.1	4,147
10 years and above	92.5	61.8	52.8	61.0	10.3	2.5	2.3	5,661
**Religion**								
Hindu	89.0	48.7	36.5	44.7	6.6	2.9	5.9	10,289
Muslim	81.8	29.4	20.8	24.7	4.5	3.4	14.9	399
Sikh	90.8	44.3	37.6	44.2	7.5	2.5	4.2	602
Other	(91.7)	(56.1)	(43.6)	(54.4)	(12.4)	(3.0)	(5.3)	40
**Caste/tribe**								
Scheduled caste	87.1	37.7	25.4	32.7	4.9	3.4	7.6	2,353
Scheduled tribe	85.4	41.8	30.3	42.2	4.5	2.1	13.2	55
Other backward class	88.7	44.7	33.9	42.5	5.9	3.2	6.4	3,369
Other	89.8	54.1	41.9	49.7	7.8	2.5	5.3	5,550
Standard of living index								
Low	81.7	29.6	15.5	21.2	2.0	4.2	13.4	1,454
Medium	88.3	42.2	29.8	38.9	5.1	2.9	6.8	4,874
High	91.5	58.6	48.1	55.6	9.4	2.4	3.4	5,001
Total	88.9	47.8	36.1	44.0	6.6	2.9	6.1	11,329

Note: Total includes 2 cases with missing information on education and 2 men with do not know in caste are not shown separately.
@ Literate men with no years of schooling are also included. ( ) Base on less than 50 unweighted cases.

8.5.4 Misconception about HIV/AIDS

People generally have misconceptions about the ways of transmission of HIV/AIDS, such as 'shaking hands with a person having AIDS', hugging and kissing with them, sharing their clothes or sharing eating utensils, stepping on urine/stool, through insect bites, for example, being bitten by mosquitoes, fleas and bedbugs. All these questions were asked to the respondents who had heard of HIV/AIDS.

Table 8.16 shows the percentage of women with misconceptions about spreading HIV/AIDS through specific ways by selected background characteristics. Being bitten by mosquitoes, fleas or bedbugs is commonly reported as the way of getting HIV/AIDS infection by women in all the groups and this percentage is higher among rural areas (18 percent) than in urban areas (15 percent). Non-literate women, women from households with a low standard of living, Muslim

women and women from scheduled tribe mentioned this method of transmission more often. Other misconceptions about the spreading of HIV/AIDS were ‘stepping on urine/stool’ (12 percent), ‘sharing eating utensils’ (15 percent), ‘kissing’ (14percent), ‘sharing clothes’ (14 percent), ‘hugging’ and ‘shaking hands’ (11 percent each). The percentage of all these misconceptions is also higher among women who belong to scheduled castes, among Muslim women, non-literate women and women with a low standard of living.

Table 8.16 MISCONCEPTION ABOUT TRANSMISSION OF HIV/AIDS AMONG WOMEN

Among currently married women aged 15-44 who have heard about HIV/AIDS, the percentage of women having misconception about the transmission of HIV/AIDS by selected background characteristics, Haryana, 2002-04

Background characteristic	Percentage having misconception about the transmission of HIV/AIDS							Number of women
	Shaking hands	Hugging	Kissing	Sharing clothes	Sharing eating utensils	Stepping on Urine/ stool	Mosquito, flea, or bedbugs biting	
**Residence**								
Rural	11.4	11.8	14.5	15.5	16.1	12.6	17.8	6,327
Urban	10.0	10.2	12.9	12.7	13.4	10.3	15.4	3,955
**Education**								
Non-literate	14.7	15.6	18.4	20.1	19.3	15.6	19.9	2,198
0-9@ years	11.7	12.2	14.8	15.8	16.8	13.0	18.1	3,743
10 years and above	8.1	8.1	10.8	10.4	11.4	8.7	14.3	4,341
**Religion**								
Hindu	10.8	11.1	13.7	14.4	14.9	11.6	16.6	9,481
Muslim	15.1	15.3	16.5	15.4	17.0	16.2	24.0	146
Sikh	11.1	12.6	16.6	15.6	16.7	12.9	20.2	605
Other	4.8	6.4	9.6	8.5	9.6	6.4	13.8	51
**Caste/tribe**								
Scheduled caste	11.8	11.9	14.9	15.1	15.5	12.6	18.0	1,712
Scheduled tribe	7.3	6.6	15.5	9.1	8.8	3.3	20.0	52
Other backward class	10.4	11.0	14.0	13.8	14.8	11.5	16.5	2,844
Other	10.8	11.1	13.5	14.5	15.0	11.7	16.7	5,674
Standard of living index								
Low	15.2	15.3	17.8	21.2	20.4	16.1	19.5	56
Medium	12.1	12.3	15.2	16.1	16.5	13.3	19.0	3,958
High	9.5	10.0	12.6	12.6	13.5	10.3	15.2	5,769
Total	10.8	11.2	13.9	14.4	15.0	11.7	16.9	10,283

Note: one woman with do not category in caste is not shown separately. @ Literate women with no year of schooling are also included.

Table 8.17 presents the percentage of men with misconceptions about the spreading of HIV/AIDS through specific ways by selected background characteristics. Again, just like the women, men in all the groups reported that HIV/AIDS is transmitted through insect bites, mosquitoes, through flea or bedbugs. 29 percent of the men in Haryana felt so. The percentage who reported that HIV/AIDS could be transmitted through the biting by mosquitoes or flees or bedbugs was higher among rural men (30 percent) than among urban men (26 percent). Non-literate men, men from households with a low and medium standard of living, Muslim and Sikh men and scheduled tribe men are of the impression that HIV/AIDS spreads when one is bitten by mosquitoes, fleas or bedbugs. Other misconceptions about the spread of HIV/AIDS are ‘sharing eating utensils’ (23 percent), ‘kissing’ (23 percent each), ‘stepping on urine/stool’ (15 percent) ‘sharing clothes’ (21 percent), ‘hugging’ (15 percent), and ‘shaking hands’ (12 percent). All the misconceptions reported by men are relatively higher than those reported by women.

Table 8.17 MISCONCEPTION ABOUT TRANSMISSION OF HIV/AIDS AMONG MEN

Among husbands currently married women who have heard about HIV/AIDS, the percentage of men having misconception about the transmission of HIV/AIDS by selected background characteristics, Haryana, 2002-04

Background characteristic	Percentage having misconception about the transmission of HIV/AIDS							Number of men
	Shaking hands	Hugging	Kissing	Sharing clothes	Sharing eating utensils	Stepping on Urine/ stool	Mosquito, flea, or bedbugs biting	
**Residence**								
Rural	12.7	15.7	24.3	22.8	24.9	16.1	29.7	7,817
Urban	10.9	12.3	19.8	17.4	18.0	12.1	25.7	3,512
**Education**								
Non-literate	20.4	25.9	38.8	37.7	40.6	26.4	35.9	1,519
0-9@ years	14.5	17.5	27.8	26.4	28.3	18.5	32.5	4,147
10 years and above	8.2	9.5	15.1	12.9	14.0	9.1	23.5	5,661
**Religion**								
Hindu	11.6	14.1	22.4	20.4	22.0	14.5	28.4	10,289
Muslim	22.4	28.0	35.4	37.4	40.4	20.0	29.6	399
Sikh	13.6	15.2	24.5	22.7	25.9	16.5	29.5	602
Other	(14.1)	(11.2)	(21.6)	(21.6)	(18.7)	(16.1)	(18.2)	40
**Caste/tribe**								
Scheduled caste	15.0	18.5	29.0	27.3	30.2	19.9	32.8	2,353
Scheduled tribe	17.7	21.2	27.4	22.2	28.6	9.2	36.5	55
Other backward class	12.8	15.4	24.1	22.2	24.5	15.2	29.6	3,369
Other	10.5	12.4	19.6	17.8	18.6	12.6	25.8	5,550
Standard of living index								
Low	20.4	25.9	36.4	34.7	38.1	23.9	32.0	1,454
Medium	13.3	16.0	25.4	23.8	26.3	16.8	31.6	4,874
High	8.6	10.0	16.7	14.6	15.0	10.3	24.4	5,001
Total	12.1	14.6	22.9	21.1	22.8	14.8	28.5	11,329

Note: One woman with do not category in caste is not shown separately. @ Literate women with no year of schooling are also included. ( ) Base on less than 50 unweighted cases.

8.5.5 Knowledge of Curability of HIV/AIDS

Table 8.18 shows the percentage distribution of currently married women and their husbands who have heard about HIV/AIDS by knowledge of curability of the same, according to some selected background characteristics. Around 25 percent women and 20 percent men have the notion that HIV/AIDS is curable, whereas 68 percent men and 51 percent women replied that the disease is not curable. Twenty four percent women and 13 percent men do not have any idea regarding the curability of the disease. It can be safely asserted from the figures that women having high level of education, belonging to Sikh religion and from households of high standard of living are showing better performance as far as the knowledge of curability of HIV/AIDS is concerned.

Table 8.18 KNOWLEDGE OF CURABILITY ABOUT HIV/AIDS

Among currently married women and their husband, who have heard about HIV/AIDS, Percent distribution of respondents by knowledge of curability about HIV/AIDS, according to some selected background characteristics, Haryana, 2002-04

Background characteristic	Percent distribution of women			Number of women	Percent distribution of men			Number of men
	Yes	No	Do not know		Yes	No	Do not know	
**Residence**								
Rural	22.6	53.0	24.4	6,327	19.6	66.5	13.9	7,817
Urban	29.9	46.9	23.2	3,955	19.3	70.1	10.6	3,512
**Education**								
Non-literate	19.5	44.2	36.3	2,198	23.8	47.2	29.0	1,519
0-9@ years	25.4	48.0	26.6	3,743	21.3	62.6	16.1	4,147
10 years and above	28.4	56.3	15.3	4,341	17.1	76.7	6.1	5,661
**Religion**								
Hindu	25.4	50.6	24.1	9,481	19.4	67.9	12.7	10,289
Muslim	17.9	43.2	39.0	146	25.0	54.3	20.7	399
Sikh	26.7	54.6	18.7	605	18.8	70.4	10.8	602
Other	38.0	46.0	15.9	51	(14.6)	(70.2)	(15.2)	40
**Caste/tribe**								
Scheduled caste	22.7	47.5	29.7	1,712	22.5	60.8	16.6	2,353
Scheduled tribe	21.4	53.6	25.0	52	26.5	63.5	10.0	55
Other backward class	23.9	51.8	24.3	2,844	19.9	65.7	14.4	3,369
Other	27.0	51.0	22.0	5,674	18.0	71.7	10.4	5,550
Standard of living index								
Low	18.7	41.6	39.6	556	23.9	53.2	22.9	1,454
Medium	22.8	50.1	27.1	3,958	20.6	64.8	14.7	4,874
High	27.8	51.9	20.3	5,769	17.3	74.5	8.1	5,001
Total	25.4	50.7	23.9	10,283	19.5	67.6	12.9	11,329

Note: Total Includes One woman and one man in do not know in caste category are not shown separately.
@ Literate persons with no year of schooling are also included. ( ) Based on less than 50 unweighted cases.

8.6 Awareness of RTI/STI and HIV/AIDS by District

Table 8.19 shows the percentage distribution of currently married women were and their husbands who are aware of RTI/STI and HIV/AIDS by districts.

According to DLHS, 51 percent and 55 percent of women ware aware of RTI/STI and HIV/AIDS respectively and the corresponding figures for husbands of eligible women are 54 and 86 percent respectively. The awareness of HIV/AIDS among men is higher than that among women by 31 percentage points and the awareness of RTI/STI among women is higher than that among men by three percentage points.

In the districts of Ambala, Fatehabad, Hisar, Jind, Karnal, Kurukshetra, Rewari, Sonipat and Yamunanagar women are more aware of RTI/STI than men. The highest level of awareness about RTI/STI among women was reported in Kurukshetra (93 percent), followed by Hisar (92 percent) to the lowest in Gurgaon (16 percent). Among men the highest level of awareness of RTI/STI was reported in Yamunanagar (78 percent) and to the lowest in Karnal (38 percent).

The proportion of husbands of eligible women for currently married women ages 15-44 who are aware of HIV/AIDS in the districts of state Haryana is also presented Table 8.19.

Among women the awareness about HIV/AIDS ranges from the highest of 76 percent in Ambala to the lowest of 31 percent in Sirsa. A high level of awareness of HIV/AIDS among men exceeding 90 percent was reported in Ambala, Hisar, Kurukshetra, Rewari, Rohtak and Yamunanagar.

Table 8.19 AWARENESS OF RTI/STI AND HIV/AIDS BY DISTRICT

Percentage of currently married women and their husbands aware of RTI/STI and HIV/AIDS by district, Haryana, 2002-04

District	Percentage of women		Percentage of men	
	Aware of RTI/STI	Aware of HIV/AIDS	Aware of RTI/STI	Aware of HIV/AIDS
Ambala	91.0	75.5	65.8	95.3
Bhiwani	14.5	46.8	59.2	85.0
Faridabad	35.7	45.7	47.1	82.2
Fatehabad	71.3	60.3	61.9	83.0
Gurgaon	16.1	35.4	56.2	77.4
Hisar	92.3	55.1	46.3	90.3
Jhajjar	51.7	66.8	56.5	88.4
Jind	72.1	50.7	47.3	83.3
Kaithal	47.6	47.5	67.2	83.5
Karnal	44.5	64.3	37.8	84.8
Kurukshetra	93.4	75.4	49.6	97.7
Mahendragarh	34.0	40.3	52.8	83.7
Panchkula	47.3	59.8	38.1	87.0
Panipat	43.7	53.1	61.2	82.2
Rewari	70.1	67.1	63.5	92.5
Rohtak	48.4	69.7	66.4	92.5
Sirsa	22.2	31.4	51.2	76.1
Sonipat	33.5	69.3	42.2	87.5
Yamunanagar	87.5	63.2	77.7	90.5
Haryana	51.0	54.7	54.2	85.8

Appendix - A

Sampling Error Estimation

The accuracy of programme indicators such as contraceptive prevalence rate, unmet need and institutional delivery, antenatal coverage etc. estimated from DLHS-RCH can be assessed in terms of stability of the estimated indicators as measured by the standard errors. Standard errors reflect only the appropriateness and suitability of sampling design adopted for RCH survey. However, the accuracy of estimated programme indicator are also affected to a great extent by non-sampling errors arising from lack of proper operationalisation and non-response cases, and is inherent in large scale surveys. The estimation producers of District Level Reproductive & Child Health survey takes into consideration design appropriateness and non-response rates. DLHS-RCH estimator of a programme indicators is design as

$$r = \frac{\sum_h \sum_j \sum_i w_{hji} y_{hji}}{\sum_h \sum_j \sum_i w_{hji} x_{hji}} = \frac{y}{x} \quad \ldots\ldots (1)$$

where the cell (h, j, i) stands for i^{th} observational unit in j^{th} primary sampling unit (PSU) in h^{th} stratum, basically rural-urban areas of a district are taken as strata. W_{hij} is the sampling weight of $(h, j, i)^{th}$ cell inflated by response rates. The variables y and x denote the main and the auxiliary characteristics required for computation of proportion or ratios.

The equation for estimation of variance of programme indicator (r) is obtained after Taylor series linearisation as

$$\text{var}(r) = \frac{1}{x^2}[\text{var}(y) + r^2 \text{var}(x) - 2r \,\text{cov}(y, x)] \quad \ldots\ldots (2)$$

$$\text{var}(y) = \sum_h \frac{n_h}{n_h - 1}\left[\sum_j \sum_i (w_{hji} y_{hij})^2 - \frac{\left(\sum_j \sum_i w_{hji} y_{hji}\right)^2}{n_h} \right] \quad \ldots\ldots (3)$$

$$\text{cov}(y, x) = \sum_h \frac{n_h}{n_h - 1}\left[\sum_j \sum_i w_{hji}^2 y_{hji} x_{hji} - \frac{(\sum_j \sum_i w_{hji} y_{hji})(\sum_j \sum_i w_{hji} x_{hji})}{n_h} \right] \quad \ldots\ldots (4)$$

and n_h is the number of sampled PSUs representing rural or urban areas of a district.

List of Selected Programme Variables for Sampling Errors, RCH 2002-04

Variable	Estimate	Base Population
CPR (Any Method)	Proportion	Currently married women age 15-44 years
Unmet Need	Proportion	Currently married women age 15-44 years
Any ANC	Proportion	Last live/still births in the past three years
ANC3+	Proportion	Last live/still births in the past three years
Institutional Delivery	Proportion	Last live/still births in the past three years
Safe Delivery	Proportion	Last live/still births in the past three years
BCG	Proportion	Children age 12-23 months
Measles	Proportion	Children age 12-23 months
BO3+	Proportion	Currently married women age 15-44 years with births in past three years

Sampling errors, Haryana, 2002-04								
Variables	Estimate (R)	Sampling error (SE)	Number of cases		Design Effect	Relative Error (%)	95% Conf. Interval	
			Unweighted	Weighted			R-1.96 SE	R+1.96 SE
**Contraceptive Prevalence Rate (Currently Married Women age 15-44)**								
Total	0.603	0.004	18,796	18,795	1.289	0.7	0.595	0.611
Rural	0.588	0.005	13,307	13,306	1.158	0.8	0.579	0.597
Urban	0.640	0.008	5,489	5,489	1.625	1.3	0.624	0.656
**Unmet Need (Currently Married Women age 15-44)**								
Total	0.1468	0.0030	18,796	18,794	1.353	2.0	0.141	0.153
Rural	0.1499	0.0034	13,307	13,306	1.194	2.3	0.143	0.157
Urban	0.1393	0.0062	5,489	5,488	1.767	4.5	0.127	0.151
**Received Any Antenatal Check up (last live/still birth of past 3 years)**								
Total	0.876	0.005	6,479	6,673	1.429	0.6	0.867	0.886
Rural	0.857	0.006	4,741	4,877	1.297	0.7	0.845	0.868
Urban	0.931	0.009	1,738	1,796	2.120	0.9	0.914	0.948
**Received 3+ Antenatal Check up (last live/still birth of past 3 years)**								
Total	0.486	0.007	6,479	6,673	1.301	1.4	0.472	0.499
Rural	0.427	0.008	4,741	4,877	1.159	1.8	0.412	0.442
Urban	0.644	0.015	1,738	1,796	1.724	2.3	0.615	0.673
**Institutional Delivery (last live/still birth of past 3 years)**								
Total	0.351	0.007	6,479	6,674	1.285	1.9	0.338	0.364
Rural	0.273	0.007	4,741	4,877	1.106	2.5	0.260	0.286
Urban	0.564	0.015	1,738	1,797	0.594	1.700	0.534	0.594
**Safe Delivery (last live/still birth of past 3 years)**								
Total	0.43	0.01	6,479	6,674	1.290	1.6	0.419	0.446
Rural	0.35	0.01	4,741	4,877	1.123	2.1	0.332	0.360
Urban	0.67	0.01	1,738	1,797	1.811	2.2	0.638	0.697
**Received BCG Vaccination (last and last but one living children, age 12-23 months)**								
Total	0.834	0.010	2,128	2,213	1.419	1.2	0.816	0.853
Rural	0.825	0.011	1,571	1,637	1.256	1.3	0.804	0.846
Urban	0.861	0.021	557	576	1.987	2.4	0.820	0.901
**Received Measles (last and last but one living children, age 12-23 months)**								
Total	0.652	0.012	2,128	2,213	1.351	1.8	0.628	0.675
Rural	0.635	0.013	1,571	1,637	1.193	2.1	0.609	0.661
Urban	0.700	0.027	557	576	1.874	3.8	0.648	0.753
**Birth order 3+ (birth in last three years)**								
Total	0.384	0.007	7,172	7,430	1.322	1.7	0.372	0.397
Rural	0.405	0.007	5,305	5,497	1.202	1.8	0.391	0.419
Urban	0.326	0.014	1,867	1933	1.689	4.2	0.299	0.353

Sampling errors, Haryana, 2002-04							
District	Estimate (R)	Sampling error (SE)	Number of cases		Relative Error (%)	95% Conf. Interval	
			Unweighted	Weighted		R-1.96 SE	R+1.96 SE
**Contraceptive Prevalence Rate (Currently Married Women age 15-44)**							
Ambala	0.708	0.015	1,010	1,010	2.1	0.679	0.737
Bhiwani	0.603	0.015	1,056	1,056	2.5	0.572	0.633
Faridabad	0.673	0.016	998	998	2.4	0.642	0.703
Fatehabad	0.527	0.017	942	943	3.2	0.494	0.560
Gurgaon	0.416	0.017	972	972	4.1	0.382	0.450
Hisar	0.638	0.016	1,033	1,033	2.5	0.606	0.670
Jhajjar	0.643	0.016	1,003	1,003	2.5	0.612	0.674
Jind	0.578	0.016	1,002	1,002	2.8	0.547	0.609
Kaithal	0.594	0.017	898	898	2.9	0.560	0.627
Karnal	0.605	0.016	1,004	1,004	2.6	0.573	0.637
Kurukshetra	0.684	0.015	1,057	1,057	2.2	0.655	0.713
Mahendragarh	0.624	0.015	1,034	1,034	2.4	0.594	0.654
Panchkula	0.699	0.016	957	957	2.3	0.668	0.731
Panipat	0.540	0.017	917	917	3.1	0.507	0.574
Rewari	0.691	0.015	1,029	1,029	2.2	0.662	0.719
Rohtak	0.632	0.016	919	919	2.5	0.600	0.664
Sirsa	0.633	0.016	913	913	2.5	0.602	0.665
Sonipat	0.621	0.016	1,016	1,016	2.6	0.590	0.652
Yamunanagar	0.621	0.016	1,036	1,036	2.6	0.590	0.652

Sampling errors, Haryana, 2002-04							
District	Estimate (R)	Sampling error (SE)	Number of cases		Relative Error (%)	95% Conf. Interval	
			Unweighted	Weighted		R-1.96 SE	R+1.96 SE
**Unmet Need (Currently Married Women age 15-44)**							
Ambala	0.064	0.008	1,010	1,010	12.5	0.049	0.080
Bhiwani	0.133	0.011	1,056	1,056	8.3	0.112	0.154
Faridabad	0.093	0.010	998	998	10.8	0.074	0.112
Fatehabad	0.212	0.014	942	943	6.6	0.184	0.239
Gurgaon	0.265	0.015	972	972	5.7	0.235	0.295
Hisar	0.086	0.010	1,033	1,033	11.6	0.068	0.105
Jhajjar	0.108	0.011	1,003	1,003	10.2	0.087	0.129
Jind	0.124	0.011	1,002	1,002	8.9	0.103	0.145
Kaithal	0.169	0.013	898	898	7.7	0.143	0.195
Karnal	0.152	0.012	1,004	1,004	7.9	0.129	0.175
Kurukshetra	0.096	0.010	1,057	1,057	10.4	0.077	0.115
Mahendragarh	0.140	0.011	1,034	1,034	7.9	0.118	0.161
Panchkula	0.106	0.011	957	957	10.4	0.086	0.127
Panipat	0.194	0.014	917	916	7.2	0.167	0.221
Rewari	0.114	0.010	1,029	1,029	8.8	0.094	0.133
Rohtak	0.122	0.011	919	919	9.0	0.100	0.143
Sirsa	0.161	0.012	913	913	7.5	0.137	0.185
Sonipat	0.171	0.012	1,016	1,016	7.0	0.147	0.194
Yamunanagar	0.121	0.011	1,036	1,036	9.1	0.099	0.142

Sampling errors, Haryana, 2002-04							
District	Estimate (R)	Sampling error (SE)	Number of cases		Relative Error (%)	95% Conf. Interval	
			Unweighted	Weighted		R-1.96 SE	R+1.96 SE
**Received Any Antenatal Check up (last live/still birth of past 3 years)**							
Ambala	0.995	0.004	325	327	0.4	0.987	1.000
Bhiwani	0.938	0.013	365	365	1.4	0.913	0.963
Faridabad	0.918	0.016	329	328	1.7	0.887	0.950
Fatehabad	0.812	0.021	393	400	2.6	0.771	0.853
Gurgaon	0.672	0.022	503	502	3.3	0.629	0.716
Hisar	0.952	0.013	318	318	1.4	0.926	0.977
Jhajjar	0.958	0.011	327	330	1.1	0.936	0.979
Jind	0.908	0.016	333	336	1.8	0.876	0.940
Kaithal	0.802	0.024	300	305	3.0	0.755	0.850
Karnal	0.944	0.013	366	376	1.4	0.919	0.968
Kurukshetra	0.977	0.008	319	323	0.8	0.960	0.993
Mahendragarh	0.896	0.018	317	312	2.0	0.861	0.930
Panchkula	0.978	0.008	325	305	0.8	0.962	0.994
Panipat	0.883	0.017	347	345	1.9	0.849	0.917
Rewari	0.895	0.018	318	316	2.0	0.860	0.930
Rohtak	0.981	0.008	320	319	0.8	0.965	0.996
Sirsa	0.786	0.024	312	311	3.1	0.740	0.833
Sonipat	0.848	0.020	340	347	2.4	0.808	0.887
Yamunanagar	0.919	0.017	322	326	1.8	0.886	0.951

Sampling errors, Haryana, 2002-04							
District	Estimate (R)	Sampling error (SE)	Number of cases		Relative Error (%)	95% Conf. Interval	
			Unweighted	Weighted		R-1.96 SE	R+1.96 SE
**Received 3+ Antenatal Check up (last live/still birth of past 3 years)**							
Ambala	0.805	0.023	325	326	2.9	0.760	0.849
Bhiwani	0.476	0.027	365	365	5.7	0.424	0.528
Faridabad	0.435	0.029	329	329	6.7	0.379	0.491
Fatehabad	0.439	0.026	393	400	5.9	0.389	0.490
Gurgaon	0.342	0.023	503	502	6.7	0.297	0.387
Hisar	0.576	0.030	318	318	5.2	0.516	0.635
Jhajjar	0.554	0.029	327	330	5.2	0.499	0.610
Jind	0.414	0.027	333	336	6.5	0.360	0.468
Kaithal	0.404	0.029	300	304	7.2	0.346	0.461
Karnal	0.563	0.027	366	377	4.8	0.511	0.616
Kurukshetra	0.740	0.026	319	324	3.5	0.690	0.790
Mahendragarh	0.359	0.028	317	313	7.8	0.304	0.413
Panchkula	0.680	0.028	325	306	4.1	0.624	0.735
Panipat	0.467	0.028	347	344	6.0	0.413	0.521
Rewari	0.472	0.029	318	316	6.1	0.416	0.528
Rohtak	0.601	0.028	320	318	4.7	0.546	0.656
Sirsa	0.345	0.027	312	312	7.8	0.292	0.398
Sonipat	0.462	0.028	340	346	6.1	0.407	0.517
Yamunanagar	0.522	0.029	322	327	5.6	0.465	0.578

Sampling errors, Haryana, 2002-04							
District	Estimate (R)	Sampling error (SE)	Number of cases		Relative Error (%)	95% Conf. Interval	
			Unweighted	Weighted		R-1.96 SE	R+1.96 SE
**Institutional Delivery (last live/still birth of past 3 years)**							
Ambala	0.618	0.028	325	326	4.5	0.564	0.672
Bhiwani	0.363	0.026	365	364	7.2	0.313	0.413
Faridabad	0.379	0.028	329	328	7.4	0.324	0.434
Fatehabad	0.312	0.024	393	399	7.7	0.266	0.359
Gurgaon	0.204	0.019	503	502	9.3	0.166	0.242
Hisar	0.324	0.029	318	318	9.0	0.267	0.381
Jhajjar	0.462	0.029	327	329	6.3	0.406	0.519
Jind	0.271	0.025	333	336	9.2	0.222	0.319
Kaithal	0.284	0.027	300	306	9.5	0.232	0.337
Karnal	0.338	0.026	366	376	7.7	0.287	0.388
Kurukshetra	0.503	0.029	319	323	5.8	0.446	0.560
Mahendragarh	0.368	0.028	317	312	7.6	0.313	0.422
Panchkula	0.451	0.032	325	305	7.1	0.389	0.513
Panipat	0.339	0.026	347	345	7.7	0.288	0.390
Rewari	0.459	0.028	318	316	6.1	0.403	0.515
Rohtak	0.419	0.029	320	319	6.9	0.363	0.475
Sirsa	0.333	0.027	312	311	8.1	0.280	0.386
Sonipat	0.411	0.028	340	346	6.8	0.357	0.465
Yamunanagar	0.316	0.027	322	326	8.5	0.263	0.368

Sampling errors, Haryana, 2002-04							
District	Estimate (R)	Sampling error (SE)	Number of cases		Relative Error (%)	95% Conf. Interval	
			Unweighted	Weighted		R-1.96 SE	R+1.96 SE
**Safe Delivery (last live/still birth of past 3 years)**							
Ambala	0.691	0.026	325	326	3.8	0.639	0.742
Bhiwani	0.411	0.026	365	364	6.3	0.360	0.462
Faridabad	0.514	0.029	329	327	5.6	0.457	0.570
Fatehabad	0.385	0.025	393	400	6.5	0.335	0.434
Gurgaon	0.231	0.020	503	503	8.7	0.191	0.271
Hisar	0.401	0.030	318	318	7.5	0.342	0.460
Jhajjar	0.554	0.028	327	330	5.1	0.498	0.610
Jind	0.354	0.027	333	336	7.6	0.302	0.406
Kaithal	0.408	0.029	300	305	7.1	0.351	0.465
Karnal	0.405	0.027	366	376	6.7	0.353	0.458
Kurukshetra	0.591	0.028	319	322	4.7	0.535	0.646
Mahendragarh	0.487	0.029	317	312	6.0	0.430	0.543
Panchkula	0.535	0.031	325	307	5.8	0.474	0.595
Panipat	0.461	0.027	347	347	5.9	0.407	0.515
Rewari	0.567	0.028	318	316	4.9	0.511	0.623
Rohtak	0.473	0.029	320	319	6.1	0.416	0.529
Sirsa	0.477	0.029	312	312	6.1	0.421	0.533
Sonipat	0.500	0.028	340	346	5.6	0.445	0.555
Yamunanagar	0.387	0.028	322	326	7.2	0.332	0.442

Sampling errors, Haryana, 2002-04							
District	Estimate (R)	Sampling error (SE)	Number of cases		Relative Error (%)	95% Conf. Interval	
			Unweighted	Weighted		R-1.96 SE	R+1.96 SE
**Received BCG Vaccination (last and last but one living children, age 12-23 months)**							
Ambala	0.990	0.010	97	98	1.0	0.971	1.009
Bhiwani	0.804	0.035	118	115	4.4	0.735	0.873
Faridabad	0.754	0.041	111	118	5.4	0.674	0.834
Fatehabad	0.844	0.039	95	98	4.6	0.768	0.921
Gurgaon	0.599	0.039	167	166	6.5	0.523	0.675
Hisar	0.901	0.038	93	94	4.3	0.826	0.977
Jhajjar	0.907	0.030	90	91	3.3	0.848	0.966
Jind	0.870	0.031	107	106	3.5	0.810	0.930
Kaithal	0.732	0.046	97	102	6.3	0.641	0.822
Karnal	0.963	0.017	114	120	1.8	0.930	0.996
Kurukshetra	0.965	0.017	94	98	1.8	0.931	0.999
Mahendragarh	0.794	0.044	78	79	5.6	0.707	0.880
Panchkula	0.981	0.011	105	98	1.1	0.959	1.003
Panipat	0.811	0.041	91	93	5.0	0.731	0.890
Rewari	0.918	0.029	89	88	3.1	0.862	0.974
Rohtak	0.978	0.013	101	100	1.3	0.953	1.004
Sirsa	0.801	0.039	102	103	4.9	0.724	0.878
Sonipat	0.858	0.035	113	117	4.0	0.790	0.926
Yamunanagar	0.963	0.021	90	90	2.2	0.921	1.004

Sampling errors, Haryana, 2002-04							
District	Estimate (R)	Sampling error (SE)	Number of cases		Relative Error (%)	95% Conf. Interval	
			Unweighted	Weighted		R-1.96 SE	R+1.96 SE
**Received Measles (last and last but one living children, age 12-23 months)**							
Ambala	0.938	0.025	97	98	2.7	0.889	0.988
Bhiwani	0.540	0.045	118	115	8.4	0.451	0.628
Faridabad	0.611	0.046	111	118	7.5	0.521	0.701
Fatehabad	0.732	0.046	95	98	6.3	0.642	0.822
Gurgaon	0.390	0.039	167	166	10.0	0.314	0.467
Hisar	0.645	0.054	93	94	8.4	0.539	0.752
Jhajjar	0.737	0.046	90	91	6.3	0.646	0.828
Jind	0.682	0.043	107	106	6.3	0.597	0.767
Kaithal	0.524	0.051	97	102	9.8	0.423	0.625
Karnal	0.800	0.038	114	120	4.7	0.726	0.874
Kurukshetra	0.826	0.039	94	98	4.7	0.750	0.901
Mahendragarh	0.582	0.055	78	79	9.4	0.474	0.689
Panchkula	0.895	0.035	105	98	3.9	0.827	0.964
Panipat	0.698	0.047	91	93	6.8	0.606	0.791
Rewari	0.752	0.046	89	88	6.2	0.661	0.843
Rohtak	0.826	0.036	101	100	4.3	0.757	0.896
Sirsa	0.568	0.049	102	103	8.6	0.473	0.664
Sonipat	0.663	0.045	113	117	6.7	0.575	0.750
Yamunanagar	0.770	0.045	90	90	5.8	0.682	0.858

Sampling errors, Haryana, 2002-04							
District	Estimate (R)	Sampling error (SE)	Number of cases		Relative Error (%)	95% Conf. Interval	
			Unweighted	Weighted		R-1.96 SE	R+1.96 SE
**Birth order 3+ (birth in last three years)**							
Ambala	0.245	0.024	354	357	9.8	0.199	0.291
Bhiwani	0.372	0.024	427	428	6.5	0.325	0.419
Faridabad	0.376	0.026	371	366	6.9	0.324	0.428
Fatehabad	0.482	0.024	457	470	5.0	0.434	0.529
Gurgaon	0.561	0.022	573	579	3.9	0.518	0.605
Hisar	0.347	0.028	339	332	8.1	0.292	0.403
Jhajjar	0.257	0.024	352	356	9.3	0.211	0.304
Jind	0.454	0.027	363	364	5.9	0.402	0.506
Kaithal	0.390	0.028	320	328	7.2	0.335	0.445
Karnal	0.360	0.025	396	415	6.9	0.311	0.410
Kurukshetra	0.276	0.024	369	376	8.7	0.229	0.323
Mahendragarh	0.350	0.026	346	342	7.4	0.298	0.401
Panchkula	0.242	0.025	342	321	10.3	0.193	0.291
Panipat	0.378	0.026	377	376	6.9	0.327	0.428
Rewari	0.288	0.025	339	333	8.7	0.238	0.337
Rohtak	0.288	0.024	363	358	8.3	0.240	0.336
Sirsa	0.362	0.026	345	346	7.2	0.311	0.413
Sonipat	0.355	0.025	378	381	7.0	0.305	0.405
Yamunanagar	0.397	0.027	361	365	6.8	0.345	0.450

Appendix - B

DLHS-RCH Staff, Haryana

Indian Institute of Health Management Research, Jaipur

Project Coordinator	Dhirendra Kumar, Ph.D.<br>Associate Professor
Senior Research Officer	J.P. Singh
Research Officers	Laxman Sharma<br>Jatinder Bir Singh<br>Vani Verma
Project Associate	Surajit Chakraborty
Trainee Research Officers	E.J. James<br>Ubeida Sulthana
Secretarial Assistant	N.K. Jacob

International Institute for Population Sciences, Mumbai

Project Coordinators	Dr. F. Ram<br>Dr. B. Paswan<br>Dr. L. Ladu Singh
Senior Research Officers	Mr. Rajiv Ranjan<br>Mr. K.C.Lakhara<br>Mr. Nizamuddin Khan

Research Officers

Mr. M. Nagavara Prasad
Mr. Akash N. Wankhede
Mr. Uttam J Sonkamble
Mr. Ashok Kumar
Ms. Jigna Thacker
Ms. Baishali Goswami
Ms. Sancheeta Ghosh
Ms. Kirti Mishra
Ms. Sucharita Pujari
Ms. Preeti Chauhan
Mrs. Santhi N.S.
Ms. Sanjeeta Gupta
Ms. Reshmi R.S.
Ms. Rinki Saha
Mr. Arnendu Kumar Jha
Mr. Atanu Ghosh
Mr. Manas Pradhan
Mr. Suhas Narkhede
Mr. Pramod Kumar Gupta
Mr. Bipul Hazarika
Dr. Manoj Alagarajan
Dr. Kalyan Saha
Dr. N Anbazhaham
Dr. Saithya Susaman
Mr. Manoj Kumar
Mr. Dibya L Mohanta
Mr. Mohan Tiwari
Mr. Battala Madhusudana
Mr. Bardanwala S.I.
Mr. Jiten Kumar Singh
Mr. Manoranjan Barik
Mr. Laxmi Prasad Sonwani
Mr. Nimakwala M. I.

Accounts and Administrative staff

Mr. Sunil Adavede (Sr. Accountant)
Mr. Jeba Kumar (Data Entry Operator)
Ms. Pratima P.Zore (Data Entry Operator)
Ms. Preeti S. Kharat (Data Entry Operator)
Ms. Sayali Shivalkar (Data Entry Operator)
Mrs. Seema V. Zagade (Office Assistant)
Mrs. Deepa J. Nair (Office Assistant)
Mr. Chandra D. Singh (Office Boy)
Mr. Ravindra P. Gawade (Office Boy)
Mr. Sanjay P. Kadam (Office Boy)

LIST OF CONTRIBUTORS

Dr. Dhirendra Kumar, Associate Professor, Indian Institute of health Management Research, 1, Prabhu Dayal Marg, Near Airport, Sanganer, Jaipur-302 011

Mr. J.P. Singh, Senior Research Officer, Indian Institute of health Management Research, 1, Prabhu Dayal Marg, Near Airport, Sanganer, Jaipur-302 011

Mr. Laxman Sharma, Research Officer, Indian Institute of health Management Research, 1, Prabhu Dayal Marg, Near Airport, Sanganer, Jaipur-302 011

Mr. Jatinder Bir Singh, Research Officer, Indian Institute of health Management Research, 1, Prabhu Dayal Marg, Near Airport, Sanganer, Jaipur-302 011

Dr. Anil Singh Jha, Research Officer, Indian Institute of health Management Research, 1, Prabhu Dayal Marg, Near Airport, Sanganer, Jaipur-302 011

Mr. B.Gowtam Ghosh, Trainee Research Officer, Indian Institute of health Management Research, 1, Prabhu Dayal Marg, Near Airport, Sanganer, Jaipur-302 011

Dr. F. Ram, Professor & Head, Department of Fertility Studies, International Institute for Population Sciences, Govandi Station Road, Deonar, Mumbai-400 088.

Dr. B. Paswan, Reader, Department of Population Policy and Programme, International Institute for Population Sciences, Govandi Station Road, Deonar, Mumbai-400 088.

Dr. L. Ladu Singh, Professor & Head, Department of Mathematical Demography and Statistics, International Institute for Population Sciences, Govandi Station Road, Deonar, Mumbai-400 088.

Mr. Uttam J. Sonkamble, Research Officer, DLHS-RCH, International Institute for Population Sciences, Govandi Station Road, Deonar, Mumbai-400 088.

Mr. Akash Wankhede, Research Officer, DLHS-RCH, International Institute for Population Sciences, Govandi Station Road, Deonar, Mumbai - 400 088.
