# Supplementary material for: Effectiveness of Multiple-Strategy Community Intervention in Reducing Geographical, Socioeconomic and Gender Based Inequalities in Maternal and Child Health Outcomes in Haryana, India
Source: PLoS One. 2016 Mar 22;11(3):e0150537. doi: 10.1371/journal.pone.0150537 (PMC4803212; doi:10.1371/journal.pone.0150537)
Supplement: S2 File — Haryana Report. Round 3. 2007–08. (PDF) [file pone.0150537.s006.pdf]

District Level Household and Facility Survey

2007-08

Haryana

International Institute for Population Sciences
(*Deemed University*)
Mumbai-400088

सत्यमेव जयते
Government of India
Ministry of Health and Family Welfare
New Delhi-110011

2010

Suggested citation:- International Institute for Population Sciences (IIPS), 2010. *District Level Household and Facility Survey (DLHS-3), 2007-08: India. Haryana*: Mumbai: IIPS.

For additional information, please contact:
Director/Project Coordinator (DLHS-3)
International Institute for Population Sciences
Govandi Station Road, Deonar
Mumbai - 400 088 (India)
Telephone: 022-2556 3254/5, 022-4237 2465, 42372411, 42372400
Fax: 022-25563257, 25555895
Email: rchpro@iips.net, director@iips.net
Website: http://www.rchiips.org
http://www.iipsindia.org

Additional Director General (Stat.)
Ministry of Health and Family Welfare
Government of India
Nirman Bhavan
New Delhi 110 011
Telephone: 011 - 23061334
Fax: 011 - 23061334
Email: adg-mohfw@nic.in

Chief Director (Stat.)
Ministry of Health and Family Welfare
Government of India
Nirman Bhavan
New Delhi 110 011
Telephone: 011 - 23062699
Fax: 011 - 23062699
Email: cdstat@nic.in
Website: http: //www.mohfw.nic.in

DLHS-3 Project Coordinators

Chander Shekhar
F. Ram
L. Ladusingh
B. Paswan
Sayeed Unisa
Rajiva Prasad
T.V. Sekher

Research Staff

Akash Wankhede
B.Amenla Nuken

Sumoni Mukherjee
Prakash Malin
S. Priyokumar Meitei
L. Priyananda Singh
Rajesh Kr. Rai
Ranjan Kr. Prusty
Namrata Mondal
Arpita Das
Mamta
Amrita Gupta
Prakash Chand D. Meher

<u>IMPORTANT INSTRUCTIONS TO READERS</u>:

This report is based on data collected from 21,406 households from Haryana during 2007-08. From these households, 21,484 ever-married women aged 15-49 years and 5,031 unmarried women aged 15-24 years were interviewed. Most of the tables and analysis presented in the report is based on ever-married women aged 15-49 years. However, for the purpose of comparison with DLHS-2 (2002-04) and the Fact Sheet of DLHS-3, we also provided some indicators based on currently married women aged 15-44 years in selected tables. We request the readers to keep this distinction in mind while using and comparing the DLHS-3 indicators with other surveys.

For more information, visit DLHS website: http://www.rchiips.org

CONTENTS

	Pages
Background and Objectives of the Survey……………………………………….…...	1
Survey Design, Sample Size and Design Weight…………………………………..	2
Implementation and Quality Control…………………..……………………..…..	4
Survey Instruments…..……………………………..………………………….	4
Household and Village Background...……………………………………………...	6
Characteristics of Women and Fertility………………………………...................	8
Maternal Health Care……………………………………………………………….	10
Child Health Care and Immunization………………………………………………	14
Family Planning and Contraceptive Use…………………………………………...	17
Reproductive Health and Awareness of RTIs/STIs and HIV/AIDS………………….	21
Infertility and Childlessness………………………………………………………...	24
Family Life Education among Unmarried Women…………………………………...	24
Reproductive Health and Awareness of Contraceptives, RTIs/STIs and HIV/AIDS among Unmarried Women…………………………………………………………….	26
Health Facilities-Availability and Quality…………………………………………	27

TABLES

APPENDIX

Sampling Errors for Selected Indicators…………………………………………… 171

LIST OF TABLES

		Page
Table 1.1	Number of households, ever married women & unmarried women interviewed………..…………	33
Table 1.2	Basic demographic indicators…………………………………………………………………	34
Table 2.1	Household population by age and sex…………………………………………………..……….	37
Table 2.2	Marital status of the household population…………………………………………….………..	38
Table 2.3	Age at marriage………………………………………………………………………..………	39
Table 2.4	Educational level of the household population……………………………………….….……	40
Table 2.5	Currently attending school…………………………………………………………..............	43
Table 2.6	Reasons for dropping out of school…………………………………………………….………..	43
Table 2.7	Household characteristics………………………………………………………….……….	44
Table 2.8	Housing characteristics and assets……………………………………………………….……...	45
Table 2.9	Housing characteristics by districts…………………………………………………................	46
Table 2.10	Distance from the nearest educational facility……………………………………….………..	47
Table 2.11	Distance from the nearest health facility…………………………………………………….…...	47
Table 2.12	Availability of facility and health personnel by districts……………………………………..….	48
Table 2.13	Knowledge about government health programmes………………………………………………...	49
Table 3.1	Background characteristics of ever married women……………………………………………..	53
Table 3.2	Level of education of ever married women………………………………… ………………..	54
Table 3.3	Birth order………………………………………………………………………………………..	55
Table 3.4	Birth order distribution by districts…………………………………………………..….............	56
Table 3.5	Children ever born…………………………………………………………………... …….…...	57
Table 3.6	Fertility preferences………………………………………………………………………………...	58
Table 3.7	Outcomes of pregnancy ……………………………………………………………….………	59
Table 3.8	Outcome of pregnancy by districts ……………………………………………………………....	60
Table 4.1	Place of antenatal check-up……………………………………………………………….……...	63
Table 4.2	Place of antenatal care by districts………………………………………………………….………	64
Table 4.3	Components of antenatal check-up…………………………………………………..……..	65
Table 4.4	Women received advice during antenatal care…………………………………..……….……...	66
Table 4.5 (A)	Antenatal care: ANC visits and time of first ANC check-up……………………. …….….…….	67
Table 4.5 (B)	Antenatal care: TT, IFA and ANC………………………………………………………….………	68
Table 4.6	Antenatal care indicators and complications by districts……………………………….…….	69
Table 4.7	Place of delivery and assistance ………………..	70
Table 4.8	Mode of transportation used for delivery and arrangement of transportation……………. ……….	71
Table 4.9	Place of delivery and assistance by districts……………………………………………….…….	72
Table 4.10	Reasons for not going to health institutions for delivery………………………. ……................	73
Table 4.11	Delivery complications……………………………………………………………..………	74
Table 4.12	Post delivery complications…………………………………………………………..……….	75
Table 4.13	Any check-up after delivery ……………………………………………………..……………	76

LIST OF TABLES

		Page
Table 4.14	Complications during pregnancy, delivery and post-delivery period ………….……….……….	77
Table 4.15	Complications during pregnancy, delivery and post-delivery period by districts………….……….	78
Table 4.16	Knowledge of danger sign of new born…………………………………………………..………	79
Table 5.1	Timing and place of early childhood check-up by background characteristics……………………	83
Table 5.2	Initiation of breastfeeding by background characteristics …………......……………………..……….	84
Table 5.3	Breastfeeding and weaning status by children's age………………………………………………..	85
Table 5.4	Exclusive breastfeeding by background characteristics…………………………………………….	85
Table 5.5	Breastfeeding by districts…………………………………………………………………………	86
Table 5.6	Vaccination of children by background characteristics ………………………………..................	87
Table 5.7	Childhood vaccination by districts……………………………………………………………….	88
Table 5.8	Place of childhood vaccination by background characteristics ………………………......……...	89
Table 5.9	Vitamin-A and Hepatitis-B supplementation for children by background characteristics ……….	90
Table 5.10	Knowledge regarding diarrhoea management by background characteristics …………….............	91
Table 5.11	Treatment of diarrhoea by background characteristics ………………………………………...	92
Table 5.12	Knowledge and treatment of acute respiratory infection (ARI) by background characteristics ……	93
Table 5.13	Knowledge of ORS and acute respiratory infection (ARI) by districts…………………..…………	94
Table 6.1	Awareness of contraceptive methods by place of residence...………………………………………	97
Table 6.2	Awareness of contraceptive methods by background characteristics……………………………….	98
Table 6.3	Awareness of contraceptive methods by districts………………………………………………...	99
Table 6.4	Ever use of contraceptive methods……………………………………………………..………...	100
Table 6.5 (A)	Current use of contraceptive methods……………………………………………..…..……..	101
Table 6.5 (B)	Duration of use of spacing methods…………………………………………………….…….……..	103
Table 6.6	Age at the time of sterilization……………………………………………………………………	104
Table 6.7	Contraceptive prevalence rate by districts………………………………………………………..	105
Table 6.8	Sources of modern contraceptive methods……………………………………………………….	106
Table 6.9	Cash benefits received after sterilization………………………………………………….………...	107
Table 6.10	Health problems with current use of contraception and treatment received……………………..…..	108
Table 6.11	Reasons for discontinuation of contraception……………………………………….………...	109
Table 6.12	Future intention to use……………………………………………………………………………….	110
Table 6.13	Advice on contraceptive use…………………………………………………………….…………..	111
Table 6.14	Reasons for not using modern contraceptive method among rhythm and withdrawal method users………………………………………………..…………………………......………	112
Table 6.15	Unmet need for family planning services…………………………………………………………...	113
Table 6.16	Unmet need for family planning services by districts……………………………….……………	114
Table 7.1	Menstruation related problems by background characteristics……………………………........	117
Table 7.2	Source of knowledge about RTI/STI by background characteristics …………….........................	119
Table 7.3	Knowledge of mode of transmission of RTI/STI by background characteristics ……………..........	121
Table 7.4	Symptoms of RTI/STI by background characteristics ……………………………………………...	122
Table 7.5	Discussed about RTI/STI problems with husband and sought treatment by background characteristics …………………………..	124

LIST OF TABLES Page

Table 7.6	RTI/STI indicators by districts	125
Table 7.7	Knowledge of HIV/AIDS by background characteristics	126
Table 7.8	Knowledge about mode of transmission of HIV/AIDS by background characteristics	128
Table 7.9	Knowledge of HIV/AIDS prevention methods by background characteristics	129
Table 7.10	Misconception about transmission of HIV/AIDS by background characteristics	130
Table 7.11	Knowledge about the place where HIV/AIDS test can be done by background characteristics	131
Table 7.12	Undergone HIV/AIDS test by background characteristics	133
Table 7.13	HIV/AIDS indicators by districts	134
Table 7.14	Ever had infertility problem by background characteristics	135
Table 7.15	Childlessness and infertility by background characteristics	136
Table 7.16	Treatment for infertility by background characteristics	137
Table 7.17	Infertility problem and sought treatment by districts	138
Table 8.1	Background characteristics of unmarried women	141
Table 8.2	At what age and standard family life education should be introduced	142
Table 8.3	Sources of family life education	143
Table 8.4	Ever received family life education by sources	144
Table 8.5	Knowledge of legal age at marriage and reported ideal age at marriage for boys and girls	145
Table 8.6	Current status of menstruation and experienced menstruation related problems during last three months and reported problems	146
Table 8.7	Practices during menstrual period	147
Table 8.8	Knowledge of contraceptive methods	148
Table 8.9	Sources from where to get pill and condom	149
Table 8.10	Discussion about family planning method by source of information	150
Table 8.11	Knowledge of RTI and STI by sources	151
Table 8.12	Knowledge of RTI/STI transmission	152
Table 8.13	Knowledge of HIV/AIDS by sources	153
Table 8.14	Knowledge of HIV/AIDS transmission	154
Table 8.15	Misconception of HIV/AIDS	155
Table 8.16	Knowledge about how to avoid or reduce the chances of infecting HIV/AIDS	156
Table 8.17	Knowledge where to get tested for HIV/AIDS and sources	157
Table 8.18	Knowledge of some selected statements	158
Table 8.19	Awareness of Reproductive Health Issues	159
Table 9.1	Average population covered by health facility by districts	163
Table 9.2	Percentage of villages having Sub-Centre within villages & ANM available at Sub-Centre and staying in Sub-Centre quarter by districts	163
Table 9.3	Status of infrastructure at Sub-Centre functioning in government building by districts	164
Table 9.4	Number of Sub-Centres having adequately equipped and essential drugs by districts	164
Table 9.5	Number of Sub-Centres having different activities by districts	165
Table 9.6	Available human resources at Primary Health Centres by districts	165

LIST OF TABLES

		Page
Table 9.7	Available infrastructure at Primary Health Centres by districts	166
Table 9.8	Specific health facilities available at Primary Health Centres by districts	166
Table 9.9	Number of Primary Health Centres having different activities by districts	167
Table 9.10	Human resources available at Community Health Centres by districts	167
Table 9.11	Specific health care facilities available at Community Health Centres by districts	168
Table 9.12	Number of Community Health Centres having different activities by districts	168

LIST OF FIGURES

Figure 1	Proportion of households by wealth quintile	7
Figure 2	Age-sex pyramid	7
Figure 3	Percentage literate by age and sex	8
Figure 4	Mean children ever-born by district	9
Figure 5	Fertility preferences of currently married women	10
Figure 6	Any ANC by background characteristics	11
Figure 7	Progress in institutional delivery	12
Figure 8	Change in full immunization coverage of children	15
Figure 9	Percent currently married women using contraceptive methods	18
Figure 10	Progress in contraceptive prevalence rate	18
Figure 11	Change in unmet need for contraception	20
Figure 12	Contraceptive prevalence rate and unmet need by district	21
Figure 13	Heard about RTIs/STIs by background characteristics	22
Figure 14	Knowledge about mode of transmission of HIV/AIDS	23
Figure 15	Knowledge about minimum legal age at marriage of boys and girls by background characteristics	25

LIST OF MAPS

Map 1	Full ante-natal check-up by districts	12
Map 2	Institutional delivery by districts	13
Map 3	Full immunization coverage of children aged 12-23 months by districts	16
Map 4	Contraceptive prevalence rate for any method by districts	19

Preface and Acknowledgements

The District Level Household and Facility Survey (DLHS-3) is a nationwide survey covering 601 districts from 34 states and union territories of India. This is the third round of the district level household survey which was conducted during December 2007 to December 2008. The survey was funded by the Union Ministry of Health and Family Welfare, United Nations Population Fund (UNFPA) and United Nations Children's Fund (UNICEF).

We are very grateful to the Ministry of Health & Family Welfare, Government of India for designating the International Institute for Population Sciences (IIPS) as the nodal agency for the DLHS-3 Project and providing an opportunity to work closely with the health and programme officials. In particular, we would like thank Ms. K. Sujatha Rao, Secretary-Ministry of Health and Family Welfare (MoHFW), Government of India for her advice, suggestions and support. We also thank Shri Naresh Dayal, former Secretary-Ministry of Health and Family Welfare (MoHFW), Government of India for the advice and valuable support extended to the project. Our special thanks to Smt. Madhu Bala, the Additional Director General, Dr. Rattan Chand, the Chief Director and Shri. Rajesh Bhatia, the Director-Statistics Division, Ministry of Health and Family Welfare, Government of India for their active involvement and suggestions. We are also thankful to Dr. V.K. Malhotra and Shri S.K. Das, former Additional Director Generals, Shri Partha Chattopadhyay, former Chief Director, Shri K. D. Maiti, former Director and Ms. Rashmi Verma, former Deputy Director-Statistics Division, MoHFW, Government of India for the co-operation and support at various stages of this project.

We are grateful to late Dr. P. N. Mari Bhat, former Director, IIPS and Dr. S. Lahiri, former Officiating Director, IIPS for their keen interest and guidance in the initial stages of the project. We acknowledge the contributions of the ORG Centre for Social Research, New Delhi the regional agency for the field implementation of DLHS-3 in Haryana state. The monitoring of the field survey was done independently by National Institute of Health and Family Welfare, New Delhi.

Our thanks to the members of Technical Advisory Committee (TAC) of DLHS-3 and especially to its Chairman, Dr. P. M. Kulkarni, Professor, Jawaharlal Nehru University, New Delhi. We also thank Dr. N.K. Singh for guiding the software development and CSPro training for the project staff. We gratefully acknowledge the immense contributions of DLHS-3 project team at IIPS in developing survey instruments, training field staff, monitoring field work, data processing, preparation of district and state level fact sheets, and drafting the reports.

Finally, special thanks to all respondents who spared their valuable time and cooperated with us by providing the required information.

DLHS-3 Coordinators
International Institute for Population Sciences

BACKGROUND AND OBJECTIVES OF THE SURVEY

The National Rural Health Mission (2005-2012) was launched by the Government of India (GoI) in 2005-06 to provide effective health care to rural population in the country with special focus on states which have poor health outcomes and inadequate public health infrastructure and manpower. The primary focus of the mission is to improve access of rural people, especially women and children, to equitable and affordable primary health care. The main goal of NRHM is to reduce infant mortality rate (IMR) and maternal mortality ratio (MMR) by promoting newborn care, immunization, antenatal care, institutional delivery and post-partum care.

The National Rural Health Mission (NRHM) foundation is built on community involvement in drawing a village health plan under the auspices of Village Health & Sanitation Committee (VHSC), making rural primary health care services accountable to the community and giving authority to the District Health Mission for implementation of inter-sectoral District Health Plan including drinking water, sanitation, hygiene and nutrition. The interface between the community and the public health system at the village level is entrusted to a female Accredited Social Health Activist (ASHA), a health volunteer receiving performance based compensation for promotion of universal immunization, referral and escort services for reproductive & child health (RCH), construction of household toilets, and other health care delivery programmes. To promote institutional delivery, cash incentive programme under Janani Suraksha Yojana (JSY) is made an integral component of NRHM.

The third round of the District Level Household and Facility Survey (DLHS-3) carried out during 2007-08 was designed to collect data at district level on various aspects of health care utilization for RCH, accessibility of health facilities, assess the effectiveness of ASHA and JSY in promoting RCH care, to assess health facility capacity and preparedness in terms of infrastructure. The integration of facility survey with the household survey was done with a view to link the RCH care outcomes to health facility accessibility, availability of medical & paramedical manpower and other village infrastructure. The broad objective of DLHS-3 is to provide RCH outcome indicators at the district level in order to monitor and provide corrective measures to the NRHM. The other important objective is to assess the contribution of decentralization of primary health care at the district level and below by way of involving village health committees under the Panchayats in the implementation of health care programmes.

The main focus and objectives of DLHS-3 is to provide RCH indicators covering the following aspects:

- Coverage of antenatal check up and immunization services
- Institutional/safe deliveries
- JSY Beneficiaries
- Contraceptive prevalence rates
- ASHA’s involvement
- Unmet need for family planning
- Awareness about RTIs/STIs and HIV/AIDS
- Family life education among unmarried adolescent girls
- Health facility and infrastructure

The District Level Household and Facility Survey, 2007-08 (DLHS-3) is the third in the series of district level household surveys. The first one was conducted in 1998-99 followed by the second in 2002-04. For all the three DLHS, the Ministry of Health and Family Welfare (MoHFW), Government of India (GoI) designated the International Institute for Population Sciences (IIPS), Mumbai, as the Nodal Agency responsible for the development of survey design, instruments, data entry and tabulation software, training, supervision of field work, analysis and report writing. The sources of funds for DLHS-3 are the MoHFW, GoI and United Nations Population Fund (UNFPA) and United Nations Children's Fund (UNICEF).

A Technical Advisory Committee (TAC) constituted by the MoHFW, GoI guided the designing, implementation, progress, tabulation, basis of selection of RCH indicators and consistency issues.

SURVEY DESIGN, SAMPLE SIZE AND DESIGN WEIGHT

A multi-stage stratified systematic sampling design was adopted for DLHS-3. In each district, 50 primary sampling units (PSUs) which were census villages in rural areas and census enumeration blocks (CEBs) in urban areas. In rural areas, villages were selected by probability proportional to size (PPS) systematic sampling and in the second stage households were selected by systematic sampling. For urban areas, first wards were selected by PPS systematic sampling, in the second stage CEBs by PPS sampling and households in the third stage by systematic sampling. The Census of India, 2001 was the sampling frame for DLHS-3. All villages and urban wards in a district were stratified by household size into three strata of less than 50, 50-300 and 300+ households, percent of SC/ST population into two strata- below and above 20 percent and implicitly by three alternating order of female literacy. These variables used for stratification are from the 2001 Census. The number of households representing a district is either 1000 or 1200 or 1500 considering the levels of immunization, antenatal check up and institutional delivery as given by DLHS-2 plus 10 percent over sampling to cushion for non-response. The PSUs are allocated to rural and urban areas of each district proportionally to the actual rural-urban population ratio and within the rural-urban domains. The PSUs are further distributed proportionately to the different sub-strata of combinations of household size, percent of SC/ST population and levels of female literacy. To make a proper rural PSU, selected villages with less than 50 households were linked with another contiguous village and selection probability is adjusted accordingly. Selected villages with more than 300 households were further divided into two or more segments and one or more segments were selected so as to have standard size PSUs. The numbers of households drawn from PSUs of districts represented by 1000, 1200 and 1500 households are 22, 27 and 33 households respectively. All ever-married women aged 15-49 years and adolescent's aged 15-24 years from the sampled households are the respondents for questions on RCH and family life education, while any adult household member is the respondent for household related questions in DLHS-3.

Sampling weight for household, ever married women and unmarried women were generated for each district. These design weights were used for computations of district level demographic and RCH indicators. The selection probabilities f^i_1, f^i_2 and f^i_3 at different stages of randomization pertaining to the ith PSU of a district were the main inputs for generation of design weight. These selection probabilities are defined as follows:

f_1^i = Probability of selection of ith rural PSU in a district

$$= \frac{(n_r * H_i)}{H}$$

Where, n_r is the number of rural PSUs selected from a district, H_i refers to the number of household in the ith psu and $H = \sum Hi$, total number of rural households in a district.

f_2^i = Probability of selecting segment (s) from segmented PSU (in case the ith selected PSU is segmented)

= (Number of households in the selected segment) / (number of households in the PSU)

The value of f_2^i is to be equal to one for un-segmented PSU.

f_3^i = probability of selecting a household from the total listed households of a PSU or in segment(s) of a PSU

$$= \frac{HS_i}{HL_i}$$

Where HL_i is the number of households listed in i[th] PSU in a district and HS_i the number of households per PSU assigned for the ith PSU is either 22 or 27or 33 depending on whether a district is represented by 1000 or 1200 or 1500 households.

For urban PSUs, f_1^i is computed as the ratio of urban population of the selected PSU to the total urban population of the district.

The probability of selecting a household from the district works out as:

$$f^i = (f_1^i * f_2^i * f_3^i)$$

The non-normalized household weight for the ith PSU of the district is, $w^i = \frac{1}{f^i * HRi}$, where HR_i is the household response rate of the ith sampled PSU, assumed to be 10 % but actual response rates are used here. The normalized weight used in the generation of district indicators as

$$n_i^d = \frac{\sum_i n_i}{\sum_i n_i * w^i} * w^i$$, i= 1, 2, 3……………, 50.

Where n_i is the number of households interviewed in the ith PSU. The weight for women is computed in the similar manner after multiplication of expression for f^i by the corresponding

response rate. State weights for households, women and husbands are further derived from the district weights n_i^d for the ith psu in d[th] district using external control so that sample results do not deviate from the corresponding information about the population.

Let, $n_s = \sum_i n_i^d$ and $N_{sc} = \sum_i N_i^d$, denote the number of households in the sample and census of a particular state, then state level households weights work out as:

$n_i^s = n_i^d * \dfrac{\left(N_i^d / N_{sc} \right)}{\left(n_i^d / n_s \right)}$, where n_i^d represents household sample in i[th] district, n_s is the total sample in the state. These households' weights are computed for rural-urban areas separately. Considering sample and census currently married women age between 15-49 years and married males above 15 years for specified state by districts and rural-urban residence, state level women weights are obtained for estimation of state level indicators.

IMPLEMENTATION AND QUALITY CONTROL

Actual field operation of DLHS-3 in different states and union territories were implemented by Regional Agencies (RA) selected by the MoHFW through a competitive bidding process, ORG Centre for Social Research, New Delhi was designated as RA for implementation of DLHS-3 in Haryana. Data from the selected PSUs were collected by a team of five persons consisting of one Supervisor, one field Editor and three Female Investigators who are graduates at least. A minimum of two days visit to each sampled PSU is followed to ensure 100 percent coverage of selected households, ever married women and unmarried women. Independent team of Health Investigators, mostly paramedics were entrusted the work of carrying out the accompanying facility survey. A strict quality check protocol was put in place by the Nodal Agency by way of spot and back checks by an independent team. The Quality team comprised of two females and one male investigator and was headed by a Research Officer from the monitoring agency. One Research Officer from IIPS was stationed in each state throughout the period of the field work for supervising the survey operations.

SURVEY INSTRUMENTS

The main instrument for collection of data in DLHS-3 was a set of structured questionnaires, namely, household, ever married woman, unmarried woman and village questionnaires. Sub-Centre, Primary Health Centre (PHC), Community Health Centre (CHC) and District Hospital (DH) questionnaires were used to conduct the facility survey. All household level questionnaires were bilingual, with questions in regional languages and in English.

Household Questionnaire

The household questionnaire lists all usual residents in each sample household including visitors who had stayed the night before the interview. For individual household member information on age, sex, marital status, relationship to the head of the household and education were collected. Marriages and deaths of members of household were also recorded. Efforts were

made to get information about maternal deaths. Information was also collected on the main source of drinking water, type of toilet facility, source of lighting, type of cooking fuel, religion and caste of household head and ownership of durable goods in the household. The other information collected relates to awareness of government programmes.

Ever Married Women's Questionnaire

The respondents for the ever married women's questionnaire were ever married women age 15-49 years living in the sampled households. Details on age, age at marriage and place of birth, educational attainment, number of biological children ever born and surviving by sex were collected. Accounts of antenatal check-up, experience of pregnancy related complications, place of delivery, delivery attendant and post-partum care, together with history of contraceptive use, sex preference of children and fertility intentions were recorded. For the recent births, immunizations status of children was collected either from the vaccination card or by asking the mother about the status of immunization of the child. The other information collected includes knowledge and awareness about RTIs/STIs and HIV/AIDS by source and treatment seeking behaviour for RTIs/STIs.

Unmarried Women's Questionnaire

Information that was collected from unmarried women age 15-24 years (those under 18 years with consent from the parents) included knowledge of family life education, awareness about legal age at marriage, awareness about contraception, menstruation related problems, and knowledge of RTIs/STIs and HIV/AIDS by source of information.

Village Questionnaire

This questionnaire was designed to collect information on availability and accessibility of education, health, transport and communication facilities at village level. Functioning of village health committees and utilization of untied funds were additionally collected from the sampled villages of DLHS.

Facility Survey Questionnaires

In the facility survey the information collected at the Sub-Centre level was on the availability of human resources, physical infrastructure, equipments and essential drugs and RCH service provided during the one month preceding the survey. Additional information collected at Primary health centre (PHC) level was availability of Lady Medical Officer, functional Labour Room, Operation Theatre, number of beds, drug storage facilities, waiting room for OPD, availability of RCH related equipments, essential drugs and essential laboratory testing facilities. Information that was collected for Community health centre (CHC) included status of in-position clinical, supporting and Para-medical staff, availability of specialists trained for NSV (Non Scalpel Vasectomy), emergency obstetric, medically terminated pregnancy (MTP), new born care, treatment of RTIs/STIs, IMNCI, ECG etc. Physical infrastructures of CHC such as, water supply, electricity, communication, waste disposal facilities, OT, Labour Room and availability of residential quarters for doctors were also collected in the facility survey. (The questionnaires are available at DLHS-3 website: www.rchiips.org).

HOUSEHOLD AND VILLAGE BACKGROUND

DLHS-3 Coverage and Response Rate

DLHS-3 surveyed a total of 21,406 households, 21,484 ever-married women and 5,031 unmarried women in Haryana. The response rates are 94.7, 89.9 and 85.4 percent for households, ever-married women and unmarried women respectively. The lowest response rates for household and for ever-married women are found in Panchkula (89.4 and 86.3 percent) and for unmarried women in Jhajjar-79.4 percent (Table 1.1). As far as the demographic indicators of Haryana are concerned, from 2001 census it can be noted that the overall sex ratio is 861 females per 1,000 males. Except in Mahendragarh (918 females per 1000 males) all other districts have sex ratio under 900 which is lower than the national average. Sex ratio is lowest in Panchkula (823 females per 1000 males) (Table 1.2).

Village Infrastructure

As regards the accessibility of health facilities to the sampled villages, 46.6 percent of villages have Sub-Centres within the village itself and as many as 90.6 percent of the villages are within 5 km. distance from a Sub-Centre. In Haryana, 12.9 percent of the villages have a government dispensary within the village and 12.6 percent have Primary Health Centres (PHC). One noticeable feature of Haryana villages is that 35.5 percent of them have private clinics within the villages (Table 2.11). Little more than one-fourths (25.8 percent) of the rural population are treated by doctors and this varies from 5.1 percent in Jhajjar to 54.1 percent in Kurukshetra and Karnal. There were no doctors in the 20 villages of Faridabad. Almost all the villages (98.2 percent) in Haryana have an *Anganwadi* worker (Table 2.12), according to this survey.

Household Characteristics

DLHS-3 surveyed a total of 1,17,052 persons (Table 2.1) from 21,406 households (Table 2.8) in Haryana covering all the twenty districts of which 72.9 percent are in rural areas and the remaining (27.1 percent) in urban areas. In Haryana, 87.1 percent of household heads are Hindus, 6.4 percent are Muslims and 6.3 percent are Sikhs. About 8.5 percent of households headed are by females. The average household size in the state is 5.5 persons and there is marginal difference in rural and urban areas. Twenty-four percent of household heads belong to scheduled castes, less than one percent (0.4 percent) belongs to scheduled tribes; 34.7 percent belongs to other backward classes and 40.6 percent to others. The median age of household head is 45 years (Table 2.7).

Almost all households (92.4 Percent) in Haryana have electricity connection, 96 percent household have improved source of water, 60.1 percent of the households have access to tap water for drinking, 46.8 percent of households have provision for flush toilet, 26.3 percent of households use LPG for cooking, more than half of the houses (58 percent) are pucca houses and 57.9 percent households have at least three rooms (Table 2.8). For the state of Haryana, 18.6 percent of households have BPL (below poverty line) cards and it varies from a low of 9.5 percent in Faridabad to a high of 27.8 percent in Jind district (Table 2.9).

Household Wealth Index

Combining household amenities, assets and durables, a wealth index is computed at the national level and divided into quintiles. Households are categorized from the poorest to the richest groups corresponding to the lowest to the highest quintiles at the national level. Based on national cut-off points, in Haryana lesser than two percent (1.7 percent) of the households are in the lowest wealth quintile and about two-fifths (37.9 percent) households are in the highest wealth quintile. In rural areas, only 12 percent households are in the lowest or in the second wealth quintile, and in urban, areas more than two-thirds of the households (67.9 percent) are in the highest wealth quintile, as shown in figure 1.

FIGURE 1
PROPORTION OF HOUSEHOLDS BY WEALTH QUINTILE

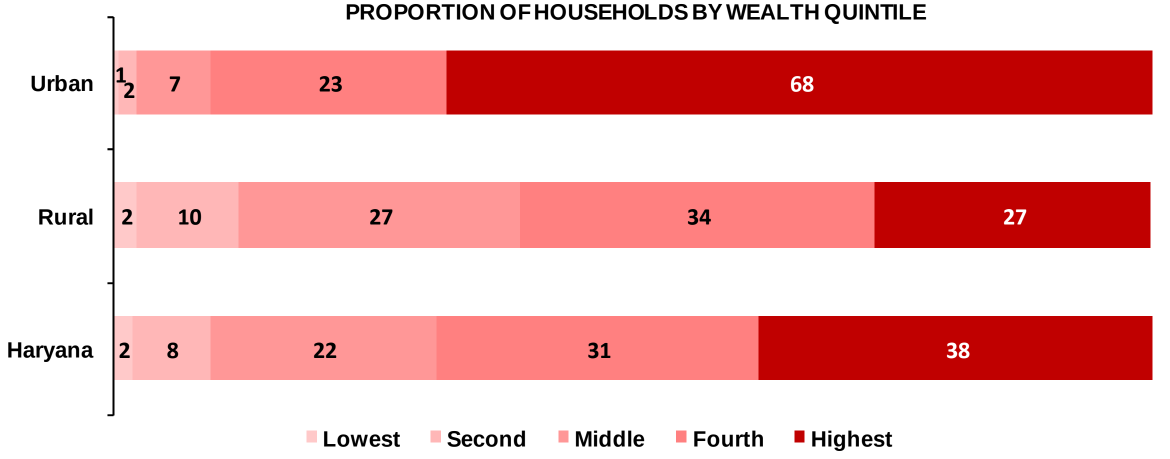

Age-Sex Composition

FIGURE 2
AGE-SEX PYRAMID

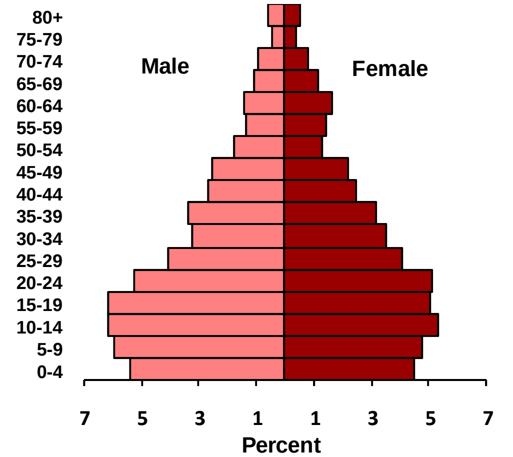

The overall sex ratio in Haryana is 907 females per 1000 males. The age-sex pyramid (figure 2) depicts a scenario of declining fertility with shrinking base of 32.1 percent of the total population below 15 years and indicates a gradually aging population with 6 percent of the population above the age of 65 years. The remaining 62 percent of the population is in the 15-64 years age group. There are more children (33.1 percent) in rural areas than urban areas (29.3 percent) (Table 2.1).

The mean age at marriage of boys and girls in Haryana are 22.7 years and 19.7 years respectively. In Haryana 27.4 percent boys and 15.9 percent girls were married before attaining the minimum legal age for marriage. Boys and girls getting married below legal age is highest in Mewat (62.5 percent boys and 43.2 percent girls) and lowest in Ambala (14.4 percent boys and 2.9 percent girls) (Table 2.3).

Literacy by Age and Sex

Twelve percent of the population 7-9 years are non-literate and non-literacy is 3.4 percent point higher for females. In the older age groups (10-14 and 15-19 years) non-literacy is higher (3.8 and 6.8 percent point respectively) for females, as shown in figure 3. There are noticeable gender gaps in rural areas in the age groups 10-14 (3.5 percent for male and 8 percent for female) and 15-19 years (5.1 percent for male and 12.7 percent for female) (Table 2.4). The information on main reasons for dropping out of school was also collected in DLHS-3. For girls below 18 years as many as 22.5 percent stated that they were not interested in studies followed by 17.2 percent said that cost too much, 15.6 percent were required for household work, further education not necessary (8.5 percent) and school too far (8 percent) as the main reasons for dropping out of school. The reported main reason for boys for dropping out of school are not interested in studies (38.4 percent), required for outside work (11.5 percent), cost too much (11.4 percent), required for household work (11.3 percent), for taking care of siblings (9.2 percent) and further education is not necessary (7 percent) (Table 2.6).

FIGURE 3
PERCENTAGE LITERATE BY AGE AND SEX

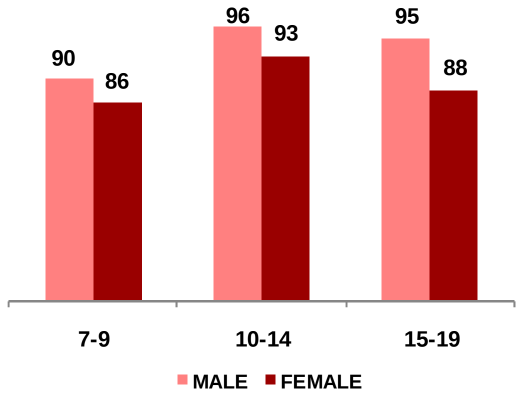

CHARACTERISTICS OF WOMEN AND FERTILITY

Age at consummation of marriage is at least 18 years for 62.8 percent of ever married sampled women aged between 15-49 years irrespective of residence. Proportion women non-literate is more in rural areas (46.8 percent) than in urban areas (26.5 percent) of Haryana. In urban areas, the proportion of husbands non-literate (10.9 percent) was lesser than women (26.5 percent). In Haryana, 24.2 percent of married women and 45.9 percent married men have at least 10 years of education. About half (47.1 percent) of ever-married women were married for 15 years or more, 20 percent women were with less than 5 years of marital duration and other one-thirds (33 percent) of ever married women were almost equally distributed in the categories of 5-9 and 10-14 years marital duration (about 17 percent in each category). In Haryana, only 1.3 percent of women belong to households in the lowest wealth quintile. In rural areas, women belonging to lowest and second lowest wealth quintiles are 1.6 and 7.6 percent respectively, and in urban areas, these are 0.3 and 1.2 percent respectively (Table 3.1).

Mean Children Ever Born by Districts

Mean Children Ever Born (MCEB) to ever-married women aged between 15-49 years is found to be 2.7 with small differential by residence (2.8 in rural and 2.5 in urban areas), while it is 3.6 for non-literates and 1.7 for women with at least 10 years of education. The completed fertility measured in terms of average children ever born to ever married women aged between 40-49 years is 3.9 (Table 3.5). District wise distribution of MCEB is depicted in the figure 4.

Out of the births to ever married women during the three years period preceding the survey, 34 percent were of third or higher order births and the corresponding figures were 56.6 and 12 percents for non-literate and for the ever-married women with 10 or more years of schooling respectively (Table 3.3). The births of third and higher order were more among the ever-married women who live in rural areas (36.1 percent), women belongs to households in lowest wealth quintile (66.3 percent) and those belonging to scheduled tribe category (52.1 percent) and less to ever-married women in urban areas (27.1 percent), those belonging to other castes (24.2 percent), those belongs to highest wealth quintile (22.2 percent) and women from richest households (17.4 percent). Births of third and higher order were highest in Bhiwani (36.8 percent) and lowest in Ambala (21.7 percent) (Table 3.4).

FIGURE 4
MEAN CHILDREN EVER BORN BY DISTRICT

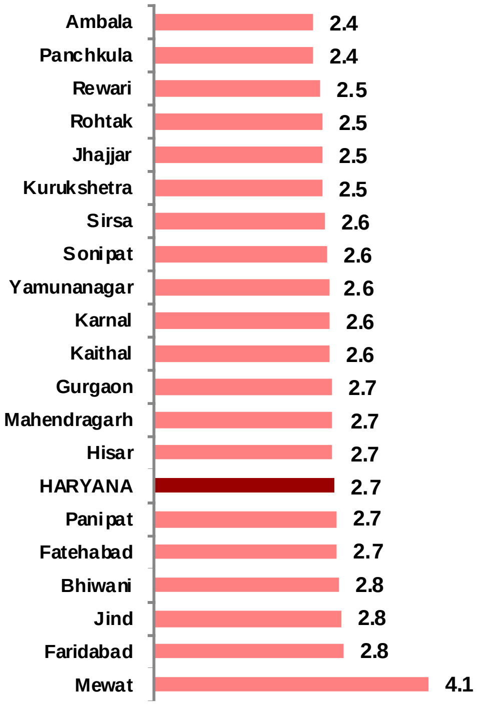

Fertility Intentions and Preferences of Currently Married Women

Fertility intensions of currently married women in terms of desire for additional child and timing to have the desired additional child (among those with no living children) was 53.1 percent who want a child soon within the next two years and 33.7 percent who want a child 2 or more years later. Among the currently married women with one living child, the proportion women wanting an additional child soon (within 2 years) and later were 20.3 and 37.9 percents respectively. Among Currently married women with two surviving children, 45.4 percent did not want any child and 42.4 percent were sterilized. As many as 32.4 percent of currently married women want no more children, 10.5 percent want a child soon, 1.8 percent are undecided and 39.7 percent have undergone sterilization (figure5). Among the currently married women with no living children but want a child, more than half of the currently married women (57.7 percent) reported that sex of the child does not matter, 25.2 percent say it is up to God, 12.8 and 4.3 percent want a boy and a girl child respectively. With increasing number of living children, longing for a boy among the currently married women who want an additional child, becomes more and more magnified from 37.5 to 80.9 percent for women with one and three living children respectively and desire for boy child had decreased to 55.7 percent among the currently married women with four or more living children (Table 3.6).

FIGURE 5
FERTILITY PREFERENCES OF CURRENTLY MARRIED WOMEN

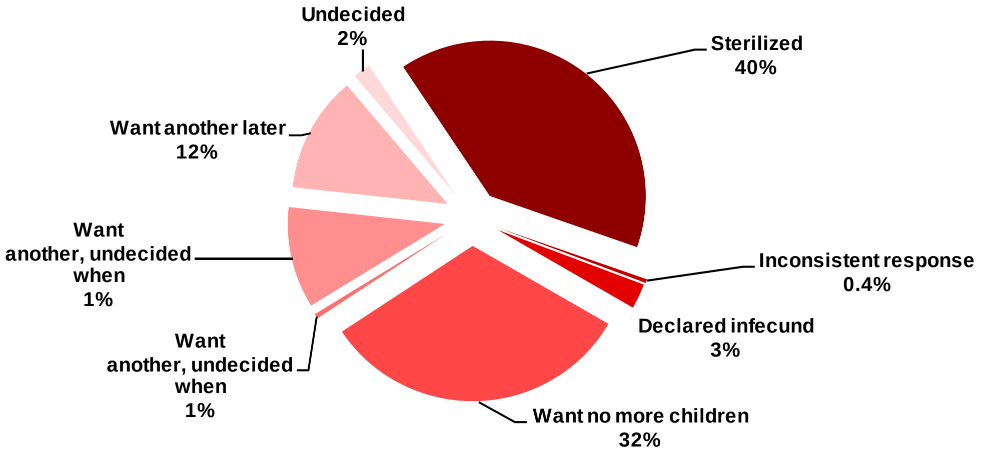

More than four-fifths (84.5 percent) of pregnancies which occurred during the three years period preceding the survey to currently married women aged between 15-49 years are found to be live births. In Haryana, 10.7 percent of the pregnancies in the three years period preceding the survey resulted in spontaneous abortion and this varies from 8.4 percent in Ambala to 15.2 percent in Rewari (Table 3.7).

MATERNAL HEALTH CARE

Maternal health care package of antenatal care is the main programme of NRHM to strengthen RCH care. ANC provided by a doctor, an ANM or other health professional comprises of physical checks, checking the position and the growth of foetus and giving TT injection at periodic intervals during the time of pregnancy. At least three check-ups are expected to complete the course of ANC to safeguard women from pregnancy related complications. Institutional delivery and post-natal care in a health facility is promoted in NRHM through the Janani Suraksha Yojana (JSY) to prevent maternal deaths.

Any ANC by Selected Background Characteristics

Among women who had their last live/still birth in the three years period preceding the survey, 87.2 percent received at least one antenatal check, 45.7 percent from government health facility, 45.8 percent from private health facility and 3.8 percent from community-based services (Table 4.1).

A distinctive feature is that any ANC is low 71.9 percent among non-literates as against 98.4 percent among women educated for 10 or more years; rural-urban gap is 8.6 percent point, with 93.7 percent among urban residents as depicted in figure 6.

The coverage of ANC was highest in Rohtak district (96.6 percent) and lowest in Mewat (54.2 percent). In all districts of Haryana, women availed ANC service from either a government or private health facility or from both facilities. For women in Panchkula district, 67.6 percent had ANC from a government facility and 40.4 percent received it from a private facility, while the corresponding government and private health facility utilization for ANC in Yamunanagar were 28.2 and 60.2 percent respectively (Table 4.2).

FIGURE 6
ANY ANC BY BACKGROUND CHARACTERISTICS

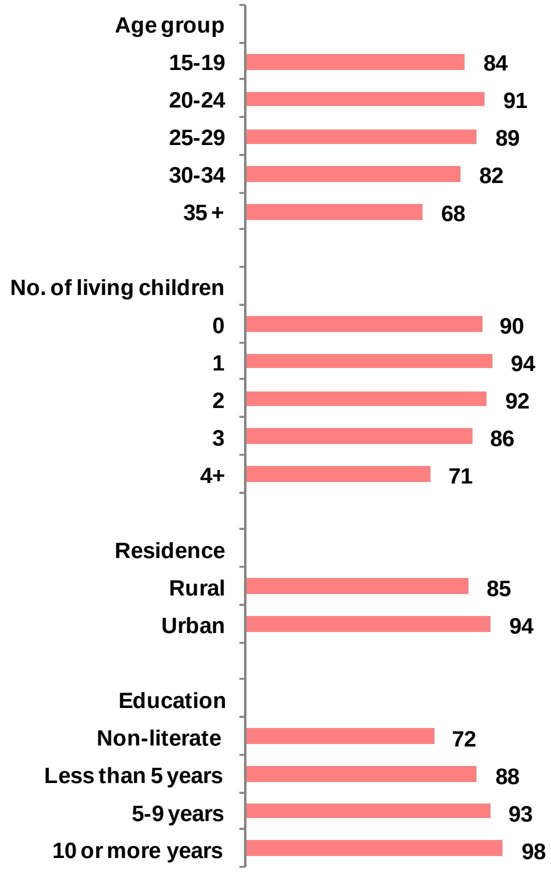

All check-ups and examinations recommended for ANC are not availed by women who had ANC during pregnancy. The proportion of women who have weight and height measured, blood pressure checked, blood and urine tested, abdomen and breast examined are 47.6, 9, 42.7, 50.2, 47.1, 58 and 18 percent respectively (Table 4.3). Women went for sonography/ultra sound test (42.1 percent), it is high in urban areas (55.7 percent), among women with 10 or more years of schooling (73.4 percent) and women belonging to the households in the highest wealth quintile (68.3 percent) (Table 4.3).

The proportion of women who received at least three ANC, among them 51.8 percent and 55 percent women had received first ANC in the first trimester, 85.9 percent women had received at least one TT injection and 13.2 percent of the women received full ANC in Haryana (Table 4.6). In Mewat, a lowest 1.9 percent and in Gurgaon and Rohtak districts a highest 27.5 percent of women have received full ANC as shown in the Map 1. The proportion of women who consumed 100 IFA tablets/syrup was 29 percent (Table 4.6).

MAP 1
FULL ANTE-NATAL CHECKS BY DISTRICTS

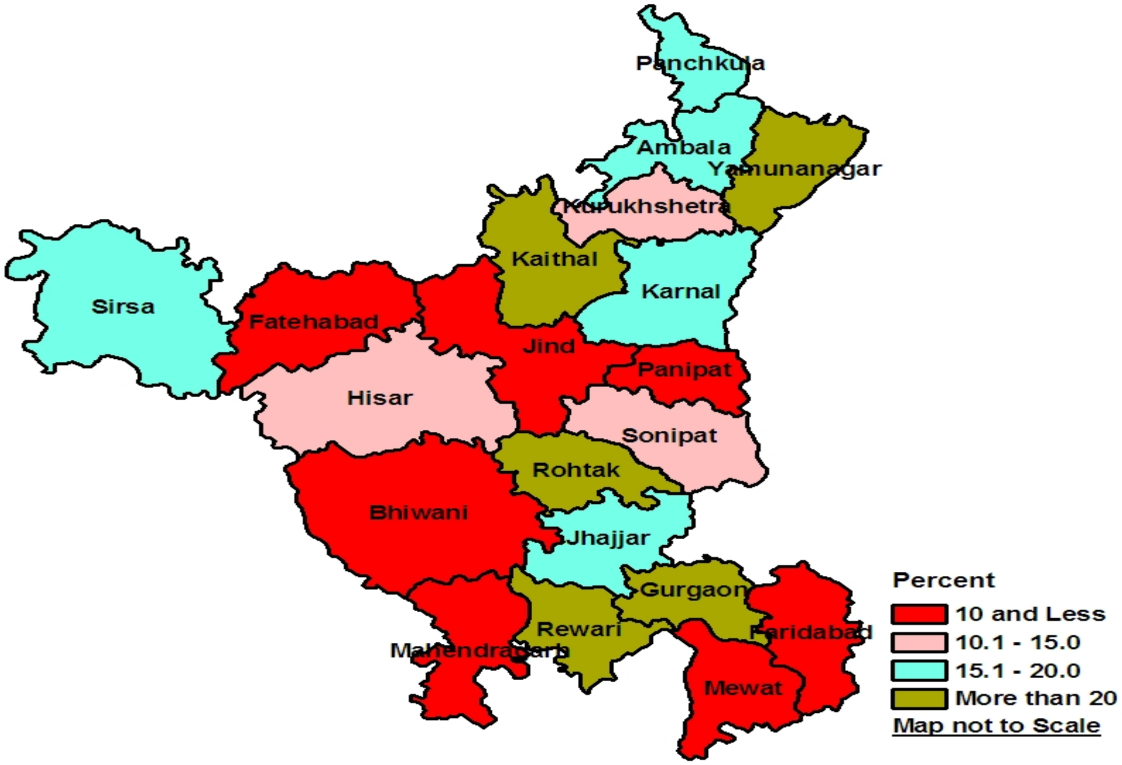

Institutional Delivery

In Haryana, the institutional delivery had increased from 26 percent in DLHS-1 (1998-99) to 35 percent in DLHS-2 (2002-04) and 47 percent in DLHS-3 (2007-08) as presented in figure 7. Forty-seven percent of deliveries since Jan 1, 2004, which results either in still or live births were done in health facilities, either public or private. The percentage of institutional delivery ranges from 14.8 percent in Mewat to 65 percent in Rewari as presented in the Map 2. Percentage of safe delivery is 73.5 percent in Rewari and 16.3 percent in Mewat districts and in Sonipat, Ambala, Mahendragarh, Kurukshetra, Panchkula and Sirsa it ranges from 61 to 69.5 percent. In Hisar, Gurgaon, Karnal, Kaithal, Jhajjar, Fatehabad, Yamunanagar and Rohtak districts of Haryana safe delivery were more than 50 percent (Table 4.9). In Haryana, 4.6 percent of the deliveries which took place in institutions after January 1, 2004 have been provided with JSY financial assistance. The mean cost of delivery at government health facility is lower (Rs.3,483) compared to private health facility (Rs.7,523). More than half (50.7 percent) of the women, who had institutional delivery used an ambulance or jeep or car as the mode of transport and mean cost of transportation was Rs.299 (Table 4.8). Women who had home

FIGURE 7
PROGRESS IN INSTITUTIONAL DELIVERY

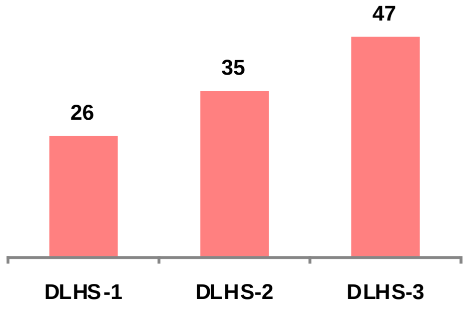

delivery, 45.7, 25.3, 22.7 and 14.2 reported institutional delivery was not necessary, better care at home, cost too much, and no time to go respectively as reasons for not opting for delivery in a health facility (Table 4.10).

MAP 2
INSTITUTIONAL DELIVERY BY DISTRICTS

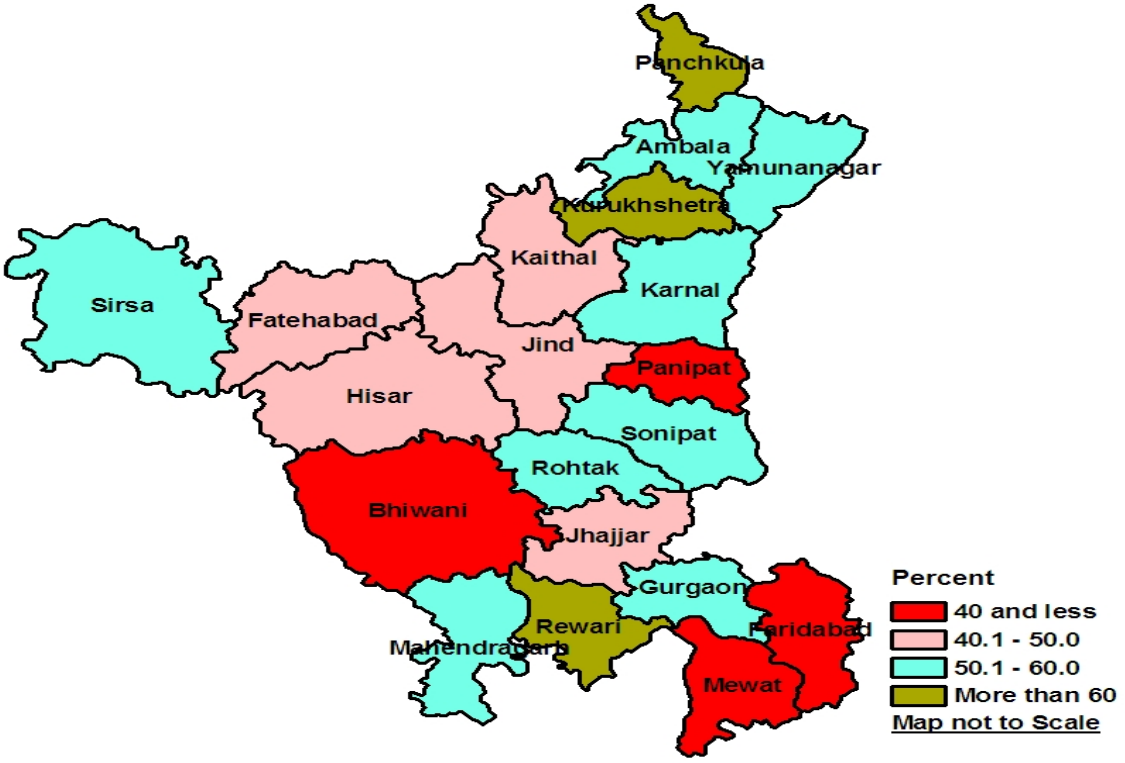

Complications during Pregnancy, Delivery and Post-delivery Period

Women who either do not take ANC or take an incomplete course of ANC are exposed to the risk of maternal death. In Haryana, as much as 56.5 percent of women who had still /live births since Jan 1, 2004, had some complications during pregnancy. This varies from 71 percent in Mewat to 42.9 percent in Sonipat. Only in five out of 20 districts in Haryana, less than 50 percent women had pregnancy complications. Among the women, who had complications during pregnancy 60 percent of them sought the treatment.

More than half (55.8 percent) of women in Haryana had faced at least one delivery complication. The main cause of delivery complications were obstructed labour (80.9 percent), premature labour (29.6 percent), prolonged labour (24.5 percent) experienced by women who had still or live births in the three year period preceding the survey (Table 4.11). Delivery complications were lowest in Rewari (37.9 percent) and highest in Mahendragarh (84.1 percent). In Kaithal, Kurukshetra, Yamunanagar, Fatehabad, Sirsa, Bhiwani, Jind and Mewat delivery complications ranges from 50.4 to 83.8 percent (Table 4.15).
More than one-fourth (28.3 percent) of women in Haryana had post-delivery complications. The major problems during post-delivery period were high fever (56.1 percent), followed by lower

abdominal pain (44.6 percent) (Table 4.12). Post-delivery complications were lowest in Mahendragarh (17.4 percent) and highest in Yamunanagar (39.8 percent). Among the women who had post-delivery complications, 88.7 percent had sought treatment in Yamunanagar and 58.3 percent in Gurgaon. Only in four out of 20 districts women who had post-delivery complications more than 80 percent of them sought treatment (Table 4.15).

CHILD HEALTH CARE AND IMMUNIZATION

To promote child survival and prevent infant mortality, NRHM envisages new born care, breastfeeding and food supplementation at the right time and a complete package of immunization for children. About half (49.5 percent) of newborn during the three years period preceding the survey were examined within 24 hours of birth. More newborns to women of urban residents (57.6 percent), newborn to women educated upto 10 or more years (70.1 percent) and belonging to other castes (60.2 percent) have received care within 24 hours compared to others. About one-fourth (22.9 percent) of the women in Haryana who had delivered in the three years period preceding the survey availed newborn check-up within 24 hours from government facility. Women who availed newborn cares from a private health facility constitute 54.8 percent as compared to newborn care availed from home (19.2 percent) and others (3 percent) (Table 5.1).

More than three-fourths (80.8 percent) of children under 3 years, born after January 1, 2004 were fed with colostrum. Women who are from urban areas (83.5 percent), more than 10 years of education (87.6 percent), other caste groups (84.5 percent) and belonging to the highest wealth quintile households (85.3 percent) were more likely to give colostrum to their children than their counterparts who live in rural areas (79.9 percent), non-literates (73.6 percent), belong to scheduled tribes (67.4 percent) and from lowest wealth quintile households (72.3 percent) (Table 5.2). There is a visible variation across districts. In the districts of Sonipat, Ambala, Faridabad, Hisar and Jhajjar (90.6-95.9 percent), Yamunanagar, Gurgaon, Mahendragarh, Rewari, Panipat, Panchkula, Karnal and Rohtak (80.1-86.8 percent) and Bhiwani, Jind, Kaithal, Fatehabad, Sirsa, Mewat and Kurukshetra (63.4-75.1 percent) of children being fed with colostrum (Table 5.5).

Less than one-fifth (16.5 percent) of women had initiated breastfeeding within one hour of birth of the child. More than half of the children (55.4 percent) started breastfeeding within 24 hours of birth (Table 5.2). The initiation of breastfeeding within one hour of birth was least practiced among women in Mewat (7.5 percent) and most widely practiced in Rewari (33.3 percent). Fifty-five percent women initiate breastfeeding within 24 hours of birth of their children, ranging from 29.2 percent in Mewat to 74.8 percent in Rewari (Table 5.5).

Median duration of exclusive breastfeeding of the youngest surviving child was 1.2 months (Table 5.3). For those children who had started food supplementation while still breastfeeding, the median age in months at the time of other fluids, semi-solid food and solid food supplementation were 1.3 months, 7 months and 11.7 months respectively. The proportion of youngest surviving child who had exclusively breastfed for 6 months was 9.4 percent (Table 5.4).

Immunization Coverage of Children Aged 12-23 Months

In DLHS-3 immunization course of children aged 12-23 months has been recorded either from vaccination card or by questioning the mothers in case the card was not available. The vaccination data from children aged 12-23 months who received specific vaccine, 38.8 percent was recorded from the vaccination card in Haryana. More than half (59.6 percent) of children aged 12-23 months received full immunization comprising BCG, three doses of DPT, three doses of Polio (excluding Polio 0) and measles. Only 1.9 percent of children have not received any vaccine (Table 5.6).

Full immunization coverage would have been well above 80 percent, if immunization against DPT did not drop down to 15.6 percent point for first (84.6 percent) to third (69 percent) dose and had vaccination against polio not dropped 29.2 percent point for first (97 percent) to third (67.8 percent) dose. The coverage of measles vaccine (69 percent) also (17.5 percent point) lower than the coverage of BCG vaccine (86.5 percent). The key to improvement in full immunization coverage is to monitor drop out at all stages of vaccination before completion of full course of immunization. Higher coverage of full vaccination is observed with boys (62.5 percent), the urban residents (70.9 percent), births of first order (68.5 percent) and children born to women educated up to 10 or more years (81.4 percent), children belong to women from other caste groups (69.5 percent) and children from households in the highest wealth quintile (74.4 percent) and it was lower for the girl children (56 percent), children have rural residence (55.9 percent), births of order four and above (31.7 percent), children of non-literate mother (34.9 percent), children whose mothers belong to scheduled tribe (43.8 percent) and children belong to households in lowest wealth quintile (28.5 percent) (Table 5.6). District-wise variation in coverage of full Immunization is depicted in the Map 3.

The coverage of full immunization was decreased from DLHS-1 to DLHS-2 (66 to 59 percent) and has increased only one percent point in DLHS-3 as depicted in figure 8. The coverage of full immunization of children is below 50 percent in 2 districts of Haryana, Mewat (11 percent) and Faridabad (46.4 percent) and it is more than 70 percent in Yamunanagar (70 percent), Gurgaon (70.5 percent), Kaithal (72.5 percent), Sonipa (73 percent), Karnal (75.2 percent), Rohtak (75.7 percent), Panchkula (78.1 percent) and Ambala (79.1 percent) (Table 5.7). With regard to the place of vaccination, children received it from a Sub-Centre (12.9 percent) and Primary Health centre (PHC) (8.4 percent), 85.2 percent from other government health facility and 9.2 percent from private health facility (Table 5.8).

FIGURE 8
CHANGE IN FULL IMMUNIZATION COVERAGE OF CHILDREN

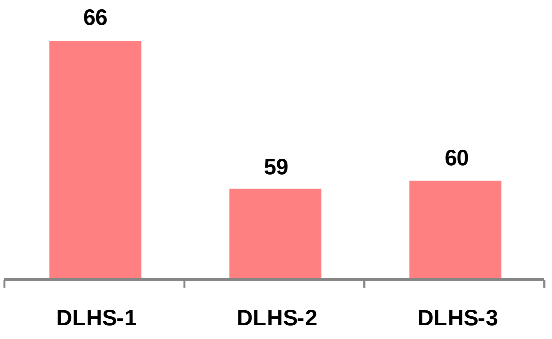

MAP 3
FULL IMMUNIZATION COVERAGE OF CHILDREN AGED 12-23 MONTHS BY DISTICTS

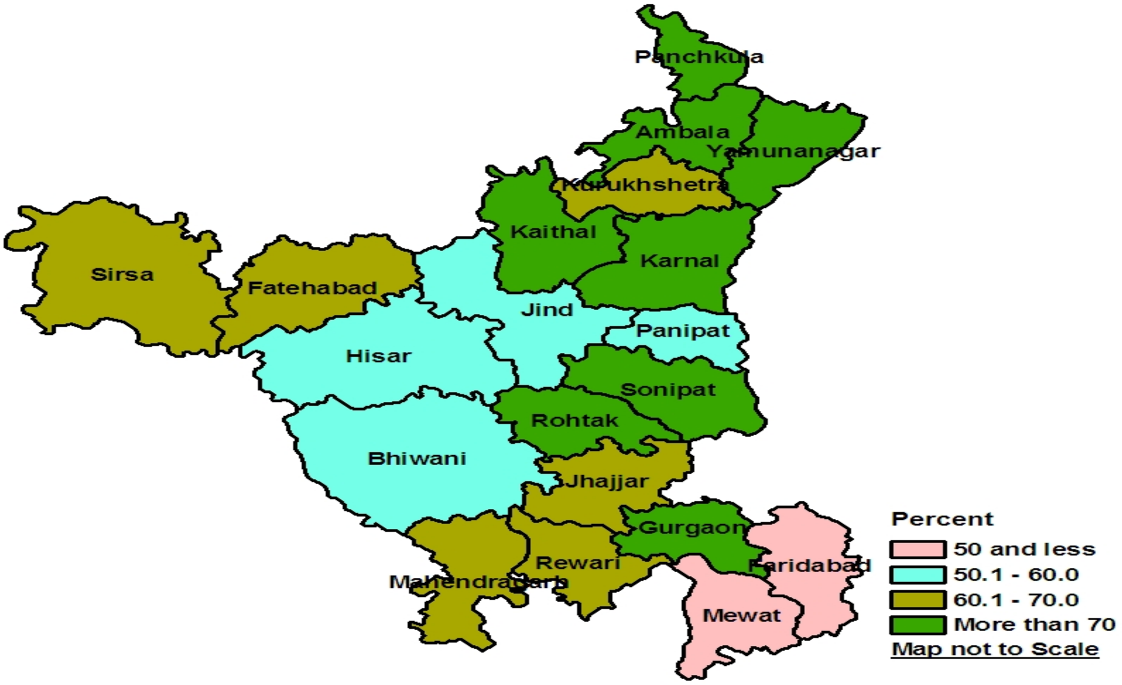

In Haryana, among children aged 12-35 months, about half of them (49.4 percent) had received at least one dose of Vitamin-A and only nine percent of children had received 3-5 doses of Vitamin-A supplementation (Table 5.9). Children from urban residence (56.6 percent), richest households (61.1 percent), other caste groups (56.8 Percent), mother's education is 10 or more years (68.4 percent), lower birth order (58.8 percent) are more likely to receive at least one dose of Vitamin-A than children from rural residence (47 percent), lowest wealth quintile households (9.5 percent), scheduled tribes (36.8 percent), non-literate mothers (31.4 percent) and children of four or more birth order (30.1 percent). In Mewat, only 7.9 percent and in Karnal 78.4 percent children aged 12-23 months received at least one dose of Vitamin-A (Table 5.7).

About one-third (32.9 percent) of children had Hepatitis-B vaccination. In Haryana, children living in urban areas (47.6 percent), lower birth order (40.6 percent), mothers having 10 or more years of education (56 percent), those belonging to other caste groups (41.9 percent) and from highest wealth quintile households (50.3 percent) are more likely to receive Hepatitis-B vaccine than children living in rural areas (28.1 percent), four or more birth order (14.5 percent), non-literate mothers (15 percent), those belonging to scheduled tribes (26.4 percent) and from lowest wealth quintile households (7.9 percent).

Diarrhoea and Acute Respiratory Infection (ARI) Management

DLHS-3 collected information on knowledge of diarrhoea and ARI management from women respondents as part of assessment of child care knowledge. More than three-fourth (79 percent) of women have knowledge of diarrhoea management (Table 5.10). The common practice followed by women for treatment of children who had diarrhoea was to give salt and sugar solution (53.9 percent), ORS (43.3 percent), plenty of fluids (24.6 percent) and continue normal

food (18.1 percent) (Table 5.10). Among the seventeen percent children who suffered from diarrhoea, 81.7 percent had sought advice/treatment and 31.7 percent among them were treated by ORS (Table 5.11). Only nine percent children who had suffered from diarrhoea are treated in a government health facility and 86.5 percent in private health facility (Table 5.11). In Sirsa 7.5 percent children suffered from diarrhoea in last two weeks prior to the survey and among them 87.8 percent had sought advice/treatment and in Karnal 33.4 percent children suffered from diarrhoea and 88.3 percent of them sought advice/treatment (Table 5.13).

More than three-fourths (76.9 percent) of women are aware about danger signs of ARI in Haryana. Sixty-two percent, 61.8, 49.5 and 38.3 percent of women know that difficulty in breathing, pain in chest and productive cough, wheezing/whistling and rapid breathings are the danger signs of ARI respectively (Table 5.12). The prevalence of ARI among children in Haryana is 8.3 percent. Eighty-eight percent of the children who suffered from ARI or fever had sought advice/treatment mostly at a private health facility (86.2 percent) (Table 5.12).

The prevalence of ARI among children varies from 2.8 percent in Mahendragarh district to 14.9 percent in Kurukshetra. The treatment seeking for ARI or fever ranges from 82.4 percent in Mahendragarh and Hisar to 98.4 percent in Panchkula (Table 5.13).

FAMILY PLANNING AND CONTRACEPTIVE USE

To achieve population stabilization and to encourage healthy married life, NRHM promotes contraceptive use on voluntary basis through a comprehensive package of improved accessibility and incentive programme. There is near universal awareness of sterilization for limiting and IUD, Pills and Condom for spacing of children among the ever married and currently married women in Haryana (Table 6.1). Female condom is least known among currently women with just 6.2 percent being aware of this contraceptive method. Emergency contraceptive pills, injectables, withdrawal and rhythm methods were known to 24.3, 96.1, 71.5, 51.6 and 69.4 percent of currently married women. Similar pattern of knowledge and in awareness of different contraceptives are also found in all the districts of Haryana (Table 6.3).

Among currently married women aged 15-49 years, the most popular method that they ever used is female sterilization (38.6 percent) followed by Rhythm (23 percent), Pill (10.6 percent) and IUD (10.3 percent). Condom/*nirodh* and withdrawal are also ever used by 21.9 and 10.2 percent currently married women's husbands. About 60 percent of women between 30-49 years are sterilized and the female sterilizations are more among rural women (42.1 percent) and non-literate (51.3 percent) women. The urban women (28 percent) and women educated for at least 10 years (16.6 percent) are less likely to use female sterilization (Table 6.4).

Among the currently married women, the proportion using any modern method is 55.7 percent, 62.9 percent of currently married women used either modern or traditional methods (figure 9). Oral pills and IUD are being used by only 2.1 and 3.5 percent of ever married women. Female sterilization is predominant among the contraceptive methods being used by 38.5 percent of currently married women aged between 15-49 years and popular male oriented spacing or temporary method like condom/*nirodh* is being used by 9.8 percent of husbands of currently married women (Table 6.5A).

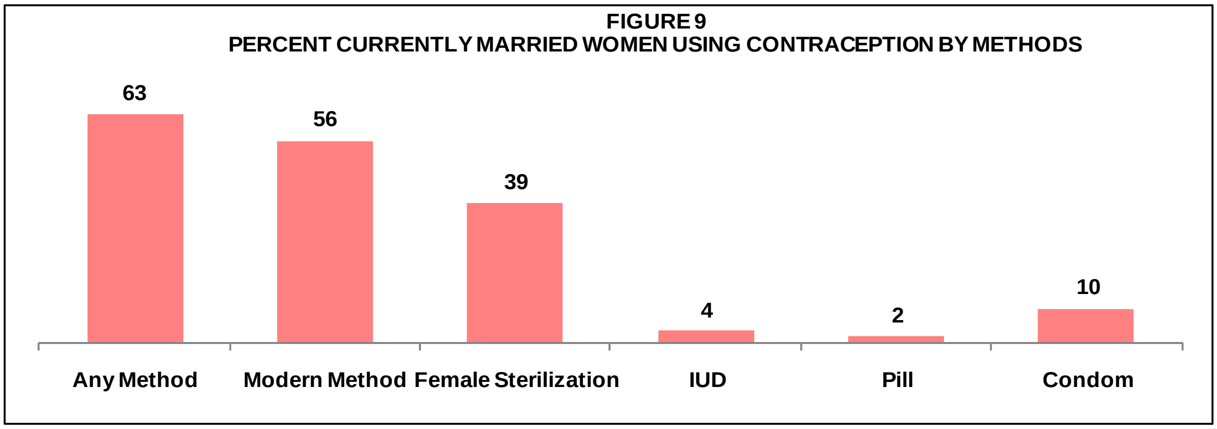

Currently married women who are in the senior age group (35-49 age group, more than 70 percent), women belongs to other castes (68.3 percent) and women from richest households (66.8 percent) are more likely to use any contraceptive method than women in the young age groups (15-24 years 12.5 to 33.5 percent), belonging to scheduled tribe (58 percent) and women from poorest households (37.5 percent). Female sterilization, regardless of family size, is more among currently married women who have one or more living son compared to those with no living son. Women in the urban areas, with more than 10 years of education, belongs to other castes and from richest households are less likely to use female sterilization and more likely to use IUD, Pill and Condom/*nirodh* (Table 6.5 A).

Among the currently married women using IUD as a spacing method, the proportion continuing IUD for less than 2, 2-3 and more than 4 years are 45, 34.8, and 20.2 percent respectively. Oral pill users continuing for more than 6 months constitute 68.2 percent of the total users and 71.5 percent of condom users are continuing with the same method for longer than 6 months (Table 6.5B). About one-thirds (32.3 percent) of women between 20-24 years, 42.1 percent of women between 25-29 years and 18.5 percent of women between 30-34 years have been found to have undergone female sterilization at the time of survey. Mean age of women at the time of sterilization is 26.6 years (Table 6.6).

Contraceptive Prevalence Rate for Any Method

The contraceptive prevalence rate for any method had changed by 2 percent points from 58 to 60 percent in DLHS-1 to DLHS-2 and then to 63 percent in DLHS-3 (figure 10). Contraceptive prevalence rate (CPR) for any method is below 30 percent is the lowest in Mewat (27.9 percent), CPR for any method ranges from 54.4-71.3 percent in all other districts (Table 6.7), depicted spatially in Map 4. The prevalence of female sterilization is more than 50 percent in Hisar, Bhiwani and Mahendragarh, Jind, Fatehabad, Sirsa, Jhajjar and Rewari are the other districts where female sterilization ranges between 40-50 percent and in Mewat it is only 12.7 percent. The use of condom is least (2.9 percent) in Mewat and highest in Panchkula (18.9 percent).

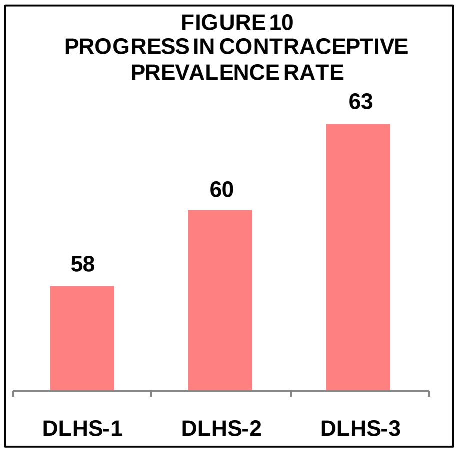

MAP 4
CONTRACEPTIVE PREVALENCE RATE BY ANY METHOD BY DISTRICTS

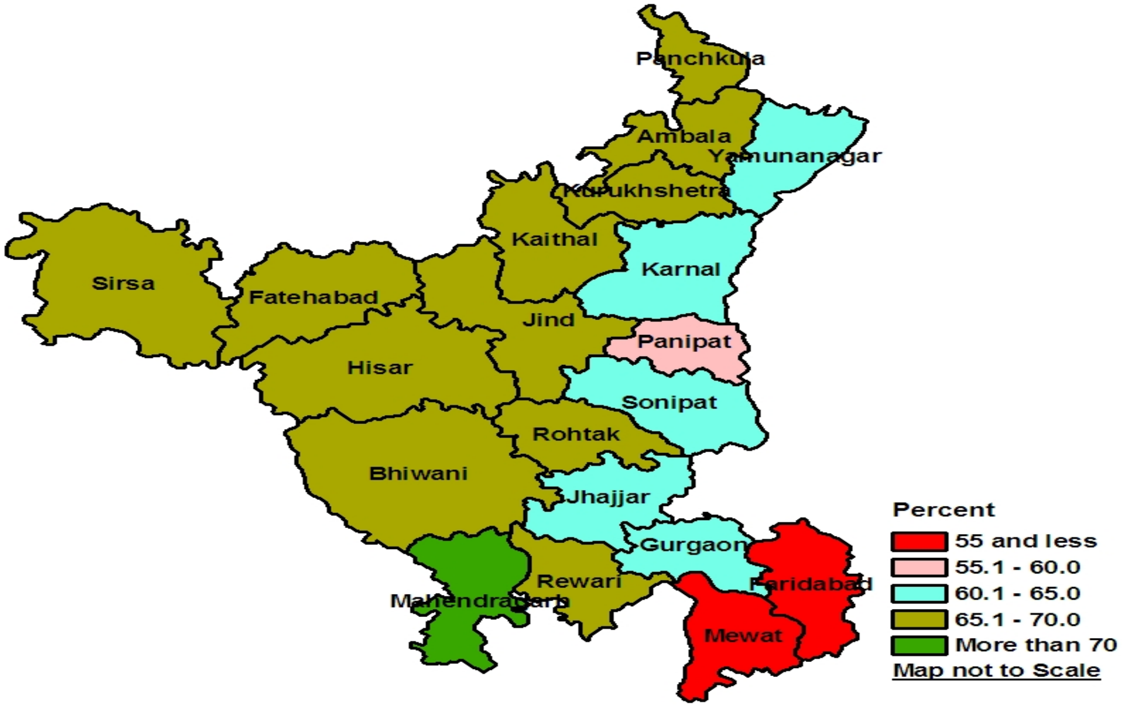

The contrast in the source of terminal and temporary methods of contraceptives is found in the state, 88.1 percent of sterilization has been done in a government health facility and only 18.7 percent have availed of government health facility services for spacing methods (Table 6.8). The high and low utilization rate of government health facility for limiting and spacing methods is true for all the districts of Haryana. In Haryana two-thirds (66.7 percent) of sterilized women and wives of men who had undergone sterilization three years preceding the survey got monetary compensation for sterilization and as much as 93.7 percent of them at the time of discharge. In Hisar, the lowest proportion 42.1 percent and highest (78.8 percent) in Rewari received cash benefits for sterilization (Table 6.9).

In Haryana, 31.1, 39.7 and 25.8 percent of sterilized women, users of IUD and Pills were informed about the side-effects before the adoption and 12.1, 11 and 8.4 percent of women using the aforesaid methods have experienced side-effects or health problems. The main health problems/side effects faced by women who have undergone sterilization were body/back ache (32.6 percent), abdominal pain (32.4 percent), weakness / inability to work (16.9 percent), irregular periods (16.4 percent) and excessive bleeding (13.2 percent). For women using IUD, the main problems were excessive bleeding (36.9 percent), abdominal pain (32.6 percent), body/back ache (22.2 percent), irregular periods (22 percent) and weakness/inability to work (10.4 percent). The proportion of Pill users with health problems/side-effects are irregular periods (28.1 percent), dizziness (25.6 percent), body/back ache (15 percent), followed by weakness/inability to work (12.8 percent), weight gain (12.7 percent) and abdominal pain (12.2 percent) (Table 6.10).

Among the currently married women who have discontinued contraception the main reason cited is related to fertility (69.1 percent), while 9.9 percent mentioned side effects and 20.9 percent cited other reasons For the younger women between 15-29 years the reasons for discontinuation of contraception are mostly fertility related and it is also true for women with no living children or only one child (Table 6.11). Seventeen percent of currently married women aged between 15-49 years not using any contraceptive intends to adopt limiting and 4.3 percent intend to use spacing method in future. Among the women who intended to adopt either limiting or spacing methods in future, 22.5 percent want to use it within 12 months and 9.2 percent want to use it after 12 months, 68.4 percent women are still undecided about the timing for adopting any family planning method (Table 6.12).

Unmet need for contraception

The total unmet need for contraception either for spacing or limiting is 15.4 percent. Currently married women physiologically fertile for conceiving and who want more children after two or more years are considered to have unmet need of contraception for spacing. In Haryana, 5.2 and 10.2 percent of currently married women have unmet need for spacing and limiting respectively. Unmet need for spacing is 18.7 percent for women with one living child and 4.5-22.4 percent of women under 25 years have unmet need for spacing (Table 6.15). The Unmet need for contraception during DLHS-2 to DLHS-3 is continuing at the same level of 15 percent as shown in the figure 11.

FIGURE 11
CHANGE IN UNMET NEED
FOR CONTRACEPTION

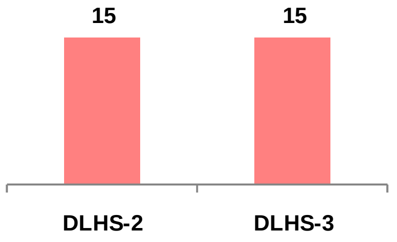

Contraceptive Prevalence Rate and Unmet Need by Districts

Unmet need is 35.4 percent in Mewat, the highest amongst the districts and the unmet need is the lowest (10.9 percent) in Sirsa. Among currently married women the unmet need for spacing was lowest (2.7 percent) in Kurukshetra and highest (13.3 percent) was in Mewat (Table 6.16). District-wise distribution of contraceptive prevalence rate and unmet need for spacing and limiting are depicted in figure 12.

On the other hand, currently married women who still are physiologically fertile for conceiving but do not want more children are categorized as having unmet need for limiting and 10.2 percent women in Haryana have unmet need for limiting. The unmet need of contraception for limiting is highest (21.9 percent) in Mewat and lowest (7.3 percent) in Hisar (Table 6.16). It can be noted that unmet need for contraception is low for districts with higher contraceptive prevalence rates.

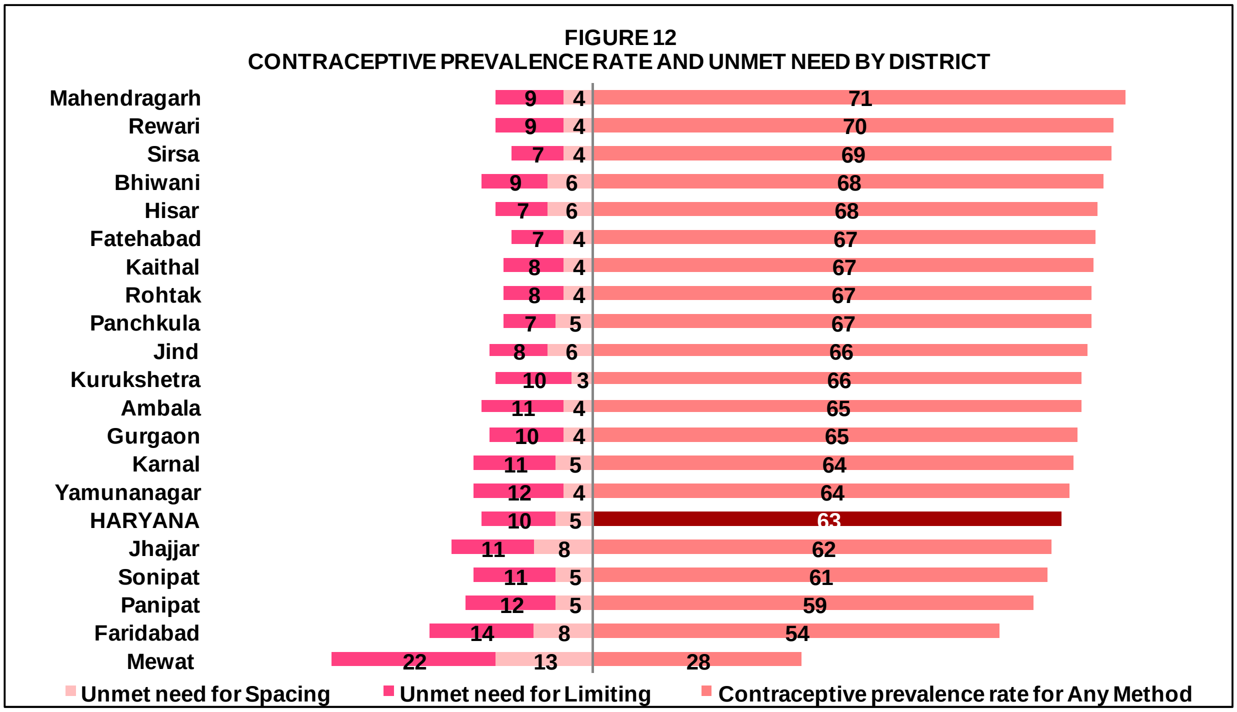

REPRODUCTIVE HEALTH AND AWARENESS OF RTIs / STIs AND HIV/AIDS

RTIs/STIs

An integrated agenda of NRHM is to promote awareness and knowledge on RTIs/STIs and HIV/AIDS and to make health facilities accessible for checking and treatment seeking to ensure healthy sexual life, free from fatal infection. One of the responsibilities of health personnel is to provide correct knowledge of reproductive tract infections (RTIs)/sexually transmitted infections (STIs), HIV/AIDS and to encourage checking and treatment.

Among ever-married women aged between 15-49 years, 18 percent have experienced one or the other menstruation related problems, largely painful periods (51.5 percent), irregular periods (30.5 percent), scanty bleeding (13 percent), prolonged bleeding (10.8 percent), blood clots/excessive bleeding (10.2 percent) and frequent or short periods (8.1 percent) are major problems reported by women in Haryana. The problem is high among the women in older age groups before 18 years at consummation of marriage, non-literate or less educated women, non-literate or less educated husband and it is invariant with the residence and wealth index of the household of the women (Table 7.1).

In Haryana, 39 percent of ever married women have heard about RTIs/STIs and it is more often heard by urban women (46.9 percent), recently married (marital duration 0-4), women (44.8 percent), women with more than 10 years education (62.2 percent) and women married more educated husbands (48.7 percent) than women living in rural areas (36.4 percent), non-literate (24.8 percent) and those married to non-literate husbands (25.1 percent) (Table 7.2) and also shown in figure13. Women have heard about RTIs/STIs from multiple sources like relatives/friends (53.9 percent), television (50.6 percent), print media (News papers /books

/magazines /slogans /pamphlets /posters) (21 percent), health personnel (11.6 percent) and radio (6.9 percent) (Table 7.2). Among those who have heard about RTIs/STIs, 70.8, 10.1, 9.7, 8.1, 5.7 and 4.6 percent have reported unsafe sex with persons who have many partners, unsafe sex with homosexuals, unsafe sex with sex worker, unsafe delivery, unsafe abortion, unsafe IUD insertion, respectively as the reasons for transmission of RTIs/STIs (Table 7.3).

FIGURE 13
HEARD ABOUT RTIs/STIs BY BACKGROUND CHARACTERISTICS

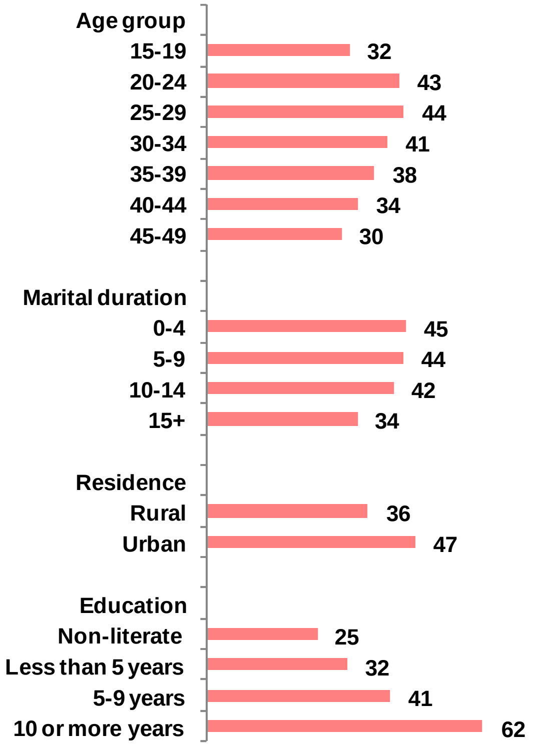

Sixteen percent of ever married women aged 15-49 years has reported having symptoms of RTIs/STIs and 14.3 percent have experienced abnormal vaginal discharge. Among the women who had RTIs/STIs symptoms, 0.2-3.5 percent of women have itching or irritation over vulva, boils/ulcers/warts around vulva, pain in lower abdomen not related to period, swelling in the groin, painful blister like lesions, pain during sexual act, spotting after sexual act (Table 7.4). More than three-fourths (80.8 percent) of the women discussed RTIs/STIs related problems with their husbands and among the women having RTIs/STIs symptoms, 52.6 percent have taken treatments, mainly from private doctors (71.2 percent) and also from a government health facility (19.1 percent) (Table 7.5).

Mewat and Panchkula are the two extreme districts as far as ever married women who have heard about RTIs/STIs are concerned (27.0 and 58.8 percent having heard of it). In the districts of Ambala, Hisar, Jhajjar, Jind, Sonipat, Rewari, Panipat, Yamunanagar, Karnal and Kurukshetra 40.7-54.2 percent of ever married women have heard about RTIs/STIs (Table 7.6). Women who have reported having abnormal vaginal discharge and any symptom of RTIs/STIs in Mewat are 27 and 24.3 percent respectively and in Panchkula it is 12.9 and 20.3 percent respectively. In Jind (41.3 percent) and Yamunanagar (69.1 percent) are two extreme districts women sought treatment for any RTIs/STIs problem (Table 7.6).

HIV/AIDS

Among the ever married women aged between 15-49 years 64.4 percent have heard of HIV/AIDS and have heard about it mostly from Television (81.5 percent), relatives/friends (37.1 percent), print media (news papers/ books/magazines/pamphlets/posters) (29.8 percent), Radio (14.8 percent). Eleven percent has heard about it from health personnel and 5.4 percent from their husband. Unlike in the case of RTIs/STIs, school/adult education program (1.2 percent) and leaders/community meeting (1.4 percent) are not a major source of knowledge for HIV/AIDS (Table 7.7). The main mode of transmission of HIV/AIDS reported by women are unsafe sex with persons having many partners (80.7 percent), transfusion of infected blood (41.2 percent), unprotected sex with an HIV/AIDS infected person (25.8 percent), mother to child (13.5

percent), unsafe sex with sex worker (11.1 percent) and unsafe sex with homosexuals (3.3 percent) (Table 7.8) as presented in figure14. There is no significant variation in the knowledge regarding the mode of transmission of HIV/AIDS by the background of women (Table 7.8).

In Mewat district, women have least heard about HIV/AIDS (18.9 percent) and majority women (86 percent) in Punchkula have heard of HIV/AIDS (Table 7.13). Among women who have heard of HIV/AIDS and reported that transmission of HIV/AIDS can be prevented by having sex with one partner or avoiding sex with homosexuals constitute (71.4 percent), avoiding getting infected through blood (safe blood transmission or avoiding sex with persons who inject drugs, use of tested blood, use only new/sterilized needles, avoid IV drip and avoid razors/blades) comprises of 44.4 percent and using condom correctly in each sexual act makes up 34.2 percent (Table 7.9). The right knowledge that HIV/AIDS transmission can be prevented by using condom is least in Faridabad (16.3 percent) and highest in Ambala (57.4 percent) (Table 7.13).

The most common misconception about transmission of HIV/AIDS among the ever married women is that one can get HIV/AIDS from mosquito, flea or bug bites as reported by 17.3 percent of them. This misconception is more among rural, non-literate and Muslim women. The proportion of women who have the misconception that HIV/AIDS can be transmitted by shaking hand and hugging, sharing clothes, sharing food and stepping on others urine/stool are 8.9, 9, 11.3,12.3 and 10 percent respectively (Table 7.10). Regardless of background characteristics, about three-fourths (71 percent) of ever married women know the place where HIV/AIDS can be tested (Table 7.11). More than 70 percent women in Ambala, Kurukshetra, Bhiwani, Sirsa, Jind, Karnal, Mahendragarh, Rewari, Yamunanagar, Kaithal, Panipat, Panchkula and Sonipat know

FIGURE 14
KNOWLEDGE ABOUT MODE OF TRANSMISSION OF HIV/AIDS

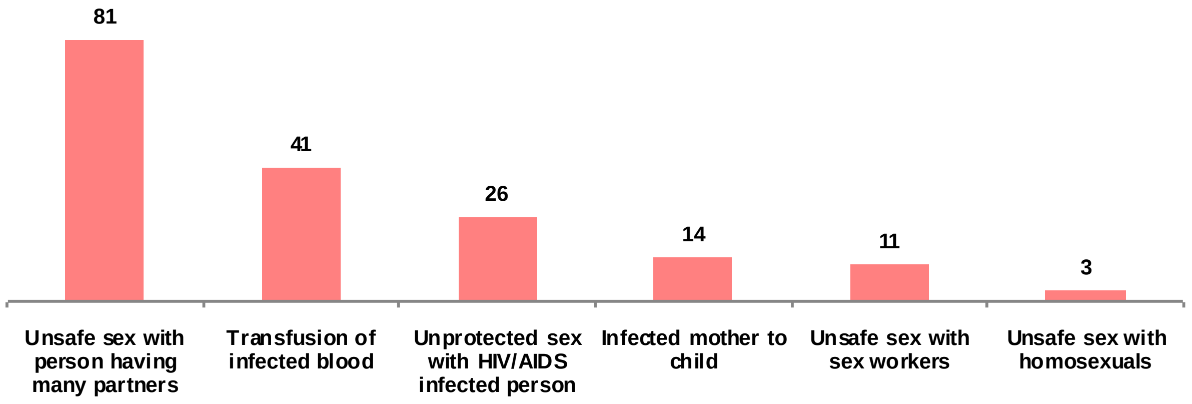

the place for testing and 42.7- 68.8 percent of women in Fatehabad, Hisar, Faridabad, Jhajjar, Gurgaon, Mewat and Rohtak are aware of the same (Table 7.13). Most women (54.3 percent) reported HIV/AIDS test can be done in a government hospital/dispensary followed by 33.8 percent reporting private hospital/clinic as the place for testing HIV/AIDS (Table 7.11). Just about 5 percent of ever-married women have undergone HIV/AIDS test, among them 56.7 percent have done it more than a year ago and 43.3 percent during the last one year period (Table 7.12).

INFERTILITY AND CHILDLESSNESS

Eleven percent of ever married women aged 15-49 years in Haryana have infertility problem, among them 8.5 and 2.6 percent had primary and secondary infertility respectively. More than three-fourths (76.7 percent) of ever married women reported to have experienced problems in conceiving for the first time; 12.1 percent had problems in conceiving after still/live birth and 9.9 percent after undergoing induced abortion (Table 7.14). In Haryana, 18 percent women have menstruation related problems. In Yamunanagar it is highest (25.7 percent), and in Fatehabad it is lowest (10.5 percent). Ambala, Panchkula, Panipat and Mahendragarh are the other districts where more than 20 percent of women have menstruation related problem. One in every ten ever married women have infertility problem including primary and secondary infertility, in all districts of Haryana (Table 7.17).

Among currently married women aged 20-49 years, who have been married for at least five years, 2 percent were childless and among them 1.6 percent had problem in conceiving. Among the ever married women between 40-49 years, 0.9 percent of them are childless (Table 7.15). among currently married women aged 20-49 years, childlessness is high among the women with 10 or more years of education (2.2 percent), 10 or more years of education of husband (2.2 percent) and among those who belong to second wealth quintile households (3.5 percent) and low among non-literate women (1.8 percent), women whose husbands are non-literate or less educated (1.9 percent), and women from poorest households (1 percent).

Women who had primary and secondary infertility among them 89.4 and 86.1 percent had sought treatment respectively (Table 7.16). More than 90 percent woman who has an infertility problem has taken treatment in Hisar, Gurgaon, Panchkula, Kurukshetra, Panipat, Rohtak, Sonipat, Ambala, Faridabad and Yamunanagar (Table 7.17).

FAMILY LIFE EDUCATION AMONG UNMARRIED WOMEN

The objective of family life education is providing knowledge about the physiological features of women and men as they grow up and make them understand the norms of marriage and reproductive health of women. In DLHS-3, right knowledge and source of information about RTIs/STIs, HIV/AIDS, Family Planning, Family Life Education, Marriage and Reproductive Health were collected from unmarried women aged between 15-24 years.

In all 5,031 unmarried women were interviewed in Haryana, of which 78.8 percent were between 15-19 years and the remaining (21.2 percent) were between 20-24 years. In rural areas, unmarried women in the later age group is 16.2 percent as against 33.5 percent in urban areas. About half (47.5 percent) of the unmarried women in rural areas have 10 or more years of education and among urban unmarried women, 73.6 percent had more than 10 years of education Highest proportion of unmarried women age 15-24 years belong to other castes in rural and urban areas (39.3 and 48.6 percent respectively). In urban areas more than three-fourths (76.5 percent) of unmarried women were from the richest wealth quintile households (Table 8.1).

Little more than three-fourths (75.8 percent) of women were aware of Family Life Education. Awareness about Family Life Education among women aged 15-19 years is below the state average among those who live in rural areas, have lower level of education; belonging to schedule caste and other backward classes, and women from households belong to lowest wealth quintile. There is a significant gap between the level of awareness and perception of importance of Family Life Education among the unmarried women in Haryana by selected background characteristics. 37.4 percent of women were of the opinion that Family Life Education should be given by age 15-17 years. As many as 46.4 percent of unmarried women felt it is important to provide Family Life Education after reaching tenth standard (Table 8.2).

FIGURE 15
KNOWLEDGE ABOUT MINIMUM LEGAL AGE AT MARRIAGE OF BOYS AND GIRLS BY BACKGROUND CHARACTERISTICS

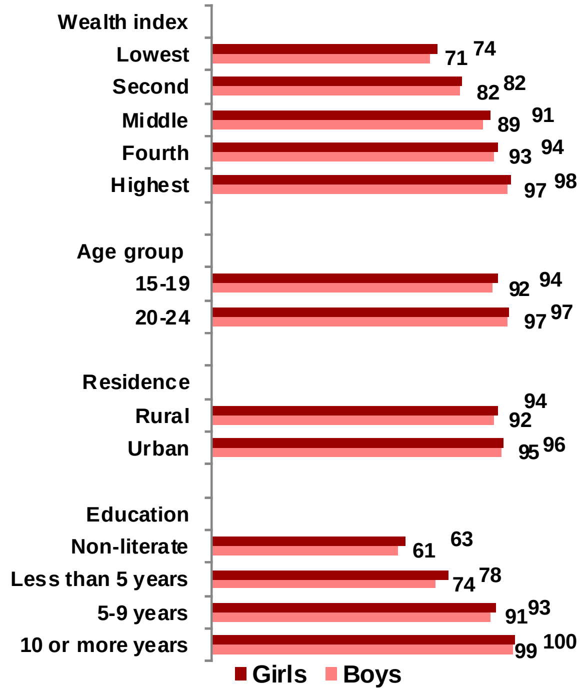

As regards the opinion on ideal persons who should impart Family Life Education, 83.7 percent of unmarried women felt that parents should provide family life education, while 58.5 percent women were of the view that it should be provided by brother/sister/sister in law. Forty-nine percent had viewed that it should be provided by teacher/school/college and 26.3 percent were of the view that it should be provided by friend/peers. Only 4.2 percent of unmarried women mentioned that healthcare provider/sex education experts were ideal persons to impart such education (Table 8.3).

Little more than two-fifths (44.8 percent) of the unmarried women in Haryana had received family life education. The main source of education is school/college (85.2 percent), followed by other sources (16 percent), 4.7 percent from youth club, 3 percent from government programme/camp and 2 percent had received Family Life Education from a NGO programme/camp (Table 8.4).

It has been observed that around 9 in every 10 unmarried women (between 15-24 years) have the knowledge of minimum legal age of marriage for boys and girls (93.1 percent among boys and 94.5 percent among girls) aged. Eleven percent reported ideal age of marriage for boys as less than 21 years while the remaining 89 percent reported more than 21 years. On the other hand, almost all women (98.5 percent) in the age group of 15-24 years reported that the ideal age of marriage for girls is 18 years and above (Table 8.5).

REPRODUCTIVE HEALTH AND AWARENESS OF CONTRACEPTIVES, RTIs/STIs AND HIV/AIDS AMONG UNMARRIED WOMEN

In DLHS-3, the status of menstruation and menstruation related problems experienced by unmarried women in the three months preceding the survey were collected. About one in every six women (15.8 percent) had menstruation related problems during the last three months preceding the survey. Eighty-one percent unmarried women had painful periods, 11.6 percent had irregular periods. Some (2.3 to 5.9 percent) women had problems like absence of menstruation, frequent or short menstrual periods, prolonged bleeding, scanty bleeding or blood clots/excessive bleeding (Table 8.6).

The practices followed during menstruation are important from the consideration of RTI and personal hygiene. Currently menstruating women were asked about the practices followed during the menstrual period. About three-fourths (73.7 percent) of the currently menstruating unmarried women used clothes, 36.6 percent use sanitary napkins and 4 percent used locally prepared napkins (Table 8.7).

Education on contraceptive means and methods is an integral component of family life education, besides the knowledge acquired from the mass media. It is imperative to assess the knowledge of contraceptive means and methods among the unmarried women. About 80.4 percent of unmarried women between 15–24 years had the knowledge of male sterilization and 94.7 percent had knowledge of female sterilization. As regards spacing methods, 86 percent had knowledge of pills, 79.4 percent had knowledge of condom/*nirodh* and 63 percent had knowledge of IUD. About one-third (30.2 percent) of unmarried women had knowledge of emergency contraception and about half (48.9 percent) have knowledge about Injectables (Table 8.8). Information was sought from the unmarried women about the places from where one can get pill and condom. More than two-thirds (68.2 percent) women were of the opinion that pill and condom can be obtained from government health facilities and another 58.4 percent said it can be had from private sources too (Table 8.9).

The unmarried women were further asked about their involvement in discussion on family planning. It has been found that 13.7 percent women had ever discussed about family planning with anyone. Nine percent had discussed with parents, 33.2 percent had discussed with brother/sister/sister-in-law, 73.3 percent with friends/peers, and more than 13.7 percent had discussed with teacher/school/college (Table 8.10).

More than one-third (36.4 percent) of unmarried women had heard about RTIs/STIs, majority (61.7 percent) of unmarried women knew about RTIs/STIs from television, 47.2 percent from print media (newspaper/books/magazines), 28.9 percent from relatives/friends, 18.6 percent from adult education programme/school teacher and 10.3 percent heard about from radio. The other sources of information are cinema, health personnel, partner, and religious/political leader/community meetings/exhibition/*mela* (Table 8.11).

Three-fourths (75.2 percent) of women mentioned that RTIs/STIs can be transmitted through unsafe sex with persons who have many partners, 12.5 percent were of the opinion that it can be transmitted through unsafe sex with homosexuals. Eight, 5.3 and 4.1 percent of unmarried

women were of the view that RTIs/STIs can be transmitted through unsafe delivery, unsafe abortion and unsafe IUD insertion (Table 8.12).

More than 80 percent of unmarried women had ever heard of HIV/AIDS. Among them 85.4 percent women knew about HIV/AIDS from television, 54.3 percent from print media, 17.6 percent from radio, 1.7 percent from cinema and 5.5 percent from health personnel. Among them about 72.4 percent said that HIV/AIDS can be transmitted through unsafe sex with person who have many partners, 60.5 percent said that through transfusion of blood, 26.9 percent said through unprotected sex with HIV/AIDS infected person, 21.3 percent said that from infected mother to child, 7.9 percent said that unsafe sex with sex workers and 5.1 percent said unsxafe sex with homosexuals (Table 8.14).

There are still many misconceptions about transmission of HIV/AIDS. Eighteen percent unmarried women were of the view that one can get HIV/AIDS by mosquito/flea or bedbugs bites, eleven percent were of the view that one can get HIV/AIDS by kissing, sharing cloths, sharing food and stepping on urine/stools of someone who has AIDS (Table 8.15). These women were further asked about how to avoid or reduce the chances of getting HIV/AIDS. About two-third (61.6 percent) of unmarried women feel that it can be avoided by avoiding sex with person who inject drugs, 58 percent feel they could avoid by limit number of sexual partner and avoid sex with sex workers, 35.9 percent feel use of condom correctly during each sexual act may reduce chances of infecting HIV/AIDS. 11.1 percent women feel that abstain from sex also may reduce the chances of infection of HIV/AIDS and 9.7 percent said avoid pregnancy when infected with HIV/AIDS (Table 8.16).

In Haryana, 75.9 percent women were aware of the place where HIV/AIDS could be tested. They knew that it can be done at the government/private hospitals (89.1 and 34 percent in government and private hospital respectively), CHC/PHC/Sub-Centre (14 percent) and VCTC/ICTC clinics (0.6 percent) (Table 8.17). Seventy-one percent of unmarried women knew that it was possible to know the sex of the baby before the baby was born by a medical test, 3.3 percent also knew that pregnancy cannot occur after kissing or hugging. Nearly nineteen percent were of the opinion that a woman was most likely to get pregnant if she had sexual intercourse half way between her periods (Table 8.18).

HEALTH FACILITIES - AVAILABILITY AND QUALITY

Health Facility Survey was conducted as a companion survey of the household survey in DLHS-3. It includes Sub-Centres, Primary Health Centres (PHCs) and Community Health Centres (CHC) which are catering to the RCH services of sampled villages. The basic objective of facility survey is to collect data on health personnel, availability of drugs/medicines, equipments, basic RCH care amenities, communication means and infrastructure at the level of Sub-Centre, PHC and CHC, in order to assess the adequacy of RCH services in rural areas. The average sampled rural population served per Sub-Centre, PHC and CHC in Haryana are 7,585, 41,500, and 1,67,422 respectively (Table 9.1). In all 721 villages were surveyed in DLHS-3 and the RCH services of these sampled villages were catered by 625 Sub-Centres, 263 PHCs and 84 CHCs.

About half (46.6 percent) of sampled villages have Sub-Centres within the villages, while the corresponding proportion is 9.7 and 81.1 percent in Yamunanagar and Hisar respectively. More than 90 percent (92.3 percent) of sampled Sub-Centres have an ANM/Female Health Worker (FHW) in position and 31.4 percent of sampled Sub-Centre an ANM/FHW residing in government quarter available at Sub-Centre. Out of sampled Sub-Centres in Kurukshetra, Panipat, Rohtak and Mewat districts none of the ANMs reside in Sub-Centre quarter, whereas in Jhajjar and Faridabad in all sampled Sub-Centre ANMs universally stay in the same (Table 9.2). Out of the 343 sampled Sub-Centres in public building only 69 (20.1 percent) Sub-Centres have regular electricity. In 204 (59.5 percent) of the Sub-Centres located in government buildings have labour rooms and out of this only 174 (85.3 percent) of the labour rooms are currently in use. There is toilet facility in 211 (61.5 percent) of the sampled Sub-Centres located in public buildings and about three-fourths (74.9 percent) of Sub-Centres housed in government buildings have provision for water (Table 9.3).

In order to assess the adequacy of the equipments in the sampled Sub-Centres, the Sub-Centres having 60 percent of essential equipments for RCH services are categorized as adequately equipped Sub-Centres, otherwise treated inadequately equipped. A similar categorization of Sub-Centres having adequate stock of essential drugs for RCH services is also followed. It is being noted that 594 (95 percent) of the sampled Sub-Centres in Haryana are adequately equipped and in Panchkula, Ambala, Kurukshetra, Sonipat, Fatehabad, Sirsa, Rohtak, Rewari and Gurgaon where hundred percent adequacy of equipments in the sampled Sub-Centres observed. On the other hand, 182 (29.1 percent) of the sampled Sub-Centres have adequate stock/supply of essential drugs for RCH services. In five districts- Yamunanagar, Sirsa, Faridabad, Hisar and Ambala - the sampled Sub-Centres have 52 to 73.9 percent adequacy of essential drugs (Table 9.4). Citizen’s charter is displayed in 46.4 percent of the sampled Sub-Centres. The proportion of sampled Sub-Centres facilitated by Village Health & Sanitation Committee (VHSC) and those that received untied funds is 91.2 and 95 percent respectively (Table 9.5).

There are 202 (76.8 percent) PHCs where Medical Officers (MO) are serving. In Ambala, Kurukshetra, Jhajjar and Rewai all the PHCs have medical officers in position. There is no Lady Medical Officer (LMO) in the sampled PHC of Sirsa. There are LMOs in position in 81 (30.8 percent) of the 263 sampled PHCs, only in 4 (1.5 percent) AYUSH doctors in position and in 246 (93.5 percent) of the sampled PHCs, Pharmacists are in position, at the time of the survey (Table 9.6).

More than two-fifths (43 percent) of the 263 sampled PHCs have residential quarters available for MO. Among the 263 sampled PHCs 103 (39.2 percent) PHCs were functioning 24 hours. About two-thirds 169 (64.3 percent) of the sampled PHCs catering to the sampled villages have at least 4 beds and such facilities are available in all the districts. Hundred and ten (41.8 percent) sampled PHCs have regular power supply and only 37 (14.1 percent) have functional vehicles in place (Table 9.7).

Newborn care equipments are available in 65 (24.7 percent) of the sampled PHCs, 159 (60.5 percent) have functional OTs and 48 (46.6 percent) provide referral services for delivery. About Eighty-five percent of the PHCs in Haryana have at least 60 percent of essential drugs and more than 65 percent of the PHCs have cold storage systems. All the districts in Haryana have

conducted at least 10 deliveries in the last one month, except in four districts Jhajjar, Mahendragarh, Rewari and Faridabad and in the state 40 PHCs (38.8 percent) among 263 PHCs have conducted at least 10 deliveries in the last one month (Table 9.8). Citizen’s Charter displayed, Rogi Kalyan Samitis (RKS) and Untied funds have been constituted in 172 (65.4 percent), 198 (75.3 percent) and 236 (89.7 percent) of the sampled 263 PHCs. Untied funds have been utilized by 230 of the 263 sampled PHCs. In Hisar, Yamunanagar, Jhajjar, Panchkula and Ambala have more than 80 percent of Citizen’s Charter displayed, Jind and Mahendragarh have hundred percent of RKS and Sonipat, Gurgaon, Sirsa, Ambala and Kurukshetra have received hundred percent of untied fund (Table 9.9).

The distribution of 84 sampled CHCs among the districts in Haryana is uneven with Hisar having 8 of them and no CHCs in Ambala, Kaithal and bhiwani districts (Table 9.10). Among 84 CHCs, only 11 CHCs (13.1 percent) have gynaecologists in position and in Panchkula, Kurukshetra, Panipat, Fatehabad, Sirsa, Rohtak, Jhajjar, Rewari, Gurgaon and Faridabad no gynaecologist in position among the sampled CHCs. The proportion of CHCs which have a Pediatrician, Anesthetist and Health Manager are 13.1, 10.7 and 9.5 percent respectively (Table 9.10). In Kurukshetra, Panipat, Sonipat, Fatehabad, Rohtak, Jhajjar, Rewari, Faridabad and Mewat the sampled CHCs which provide RCH services to the sampled villages have no blood storage facility (Table 9.11). For the state as a whole, only 7 (18.9 percent) of the sampled 84 CHCs have blood storage facility. All the sampled CHCs in Panchkula are designated as FRUs and overall 44.1 percent of the sampled CHCs are designated as FRUs. Out of the 84 sampled CHCs, 51 (60.7 percent), 23 (62.2 percent) and 18 (21.4 percent) have functional OTs, newborn care facilities and low birth weight (LBW) management facilities. Out of the sampled 84 CHCs, in 86.9 percent of CHCs, RKS have been constituted and 97.3 percent of the constituted RKS are monitored regularly (Table 9.12).

TABLES

RESPONSE RATES AND DEMOGRAPHIC INDICATORS

TABLE 1.1 NUMBER OF HOUSEHOLDS, EVER-MARRIED WOMEN & UNMARRIED WOMEN INTERVIEWED

Number of households, ever-married women and unmarried women interviewed by district, Haryana, 2007-08

	Number of households interviewed				Number of ever-married women interviewed				Number of unmarried women interviewed			
State/district	Total	Rural	Urban	Response rate	Total	Rural	Urban	Response rate	Total	Rural	Urban	Response rate
Haryana	21,406	15,615	5,791	94.7	21,484	16,154	5,330	89.9	5,031	3,585	1,446	85.4
Panchkula	991	586	405	89.4	951	630	321	86.3	255	159	96	81.5
Ambala	1,037	670	367	94.0	980	669	311	88.9	336	208	128	86.8
Yamunanagar	1,064	674	390	98.7	1,067	718	349	92.4	349	215	134	89.0
Kurukshetra	1,036	774	262	94.0	1,002	767	235	90.9	276	207	69	84.7
Kaithal	1,036	833	203	93.8	1,097	890	207	91.0	260	205	55	88.7
Karnal	1,054	788	266	95.2	1,051	803	248	90.8	299	226	73	84.9
Panipat	1,049	637	412	94.3	1,026	669	357	88.4	241	150	91	86.4
Sonipat	1,062	787	275	95.4	1,048	803	245	92.0	250	173	77	88.3
Jind	1,056	850	206	95.7	1,070	868	202	87.0	267	205	62	87.3
Fatehabad	1,087	892	195	97.3	1,073	889	184	91.7	280	232	48	83.6
Sirsa	1,053	781	272	95.5	1,096	825	271	91.4	295	219	76	87.8
Hisar	1,050	783	267	95.5	1,010	773	237	88.8	214	149	65	84.9
Bhiwani	1,073	888	185	96.9	1,112	930	182	88.7	209	165	44	86.7
Rohtak	1,043	676	367	94.1	1,056	706	350	89.0	228	126	102	86.0
Jhajjar	1,033	816	217	93.8	1,022	817	205	87.9	200	154	46	79.4
Mahendragarh	1,061	914	147	95.5	1,117	972	145	89.1	190	156	34	81.9
Rewari	1,039	851	188	93.9	1,117	931	186	91.6	248	205	43	87.3
Gurgaon	1,279	825	454	94.7	1,265	830	435	93.9	236	153	83	82.2
Faridabad	1,024	415	609	92.7	994	437	557	89.8	204	104	100	87.9
Mewat	1,279	1175	104	93.8	1,331	1,228	103	87.8	194	174	20	80.5

Note: Table based on unweighted cases.

TABLE 1.2 BASIC DEMOGRAPHIC INDICATORS

Basic demographic indicators of Haryana and its districts, Census 2001, India

State/district	Population (in thousands)	Percentage urban	Percentage decadal growth rate[2]	Sex ratio[3]	Percentage literate 7+		
					Male	Female	Total
Haryana	21,144	28.9	28.4	861	78.5	55.7	67.9
Panchkula	468	44.5	51.2	823	82.7	65.7	74.0
Ambala	1,014	35.2	25.7	868	83.0	63.4	75.3
Yamunanagar	1,042	37.7	21.8	862	79.3	60.6	71.6
Kurukshetra	825	26.1	23.7	866	78.2	67.4	69.9
Kaithal	946	19.4	21.0	853	69.8	47.3	67.7
Karnal	1,274	26.5	23.1	865	76.7	58.0	67.7
Panipat	967	40.5	38.6	829	79.2	58.0	69.2
Sonipat	1,279	25.1	22.4	839	84.0	60.7	72.8
Jind	1,190	20.3	21.4	852	74.7	48.5	62.1
Fatehabad	806	17.6	24.8	884	68.7	46.5	58.0
Sirsa	1,117	26.3	23.0	882	70.9	49.9	60.6
Hisar	1,230	25.9	27.1	851	77.6	52.1	65.9
Bhiwani	1,425	19.0	22.5	879	81.2	53.0	67.4
Rohtak	940	35.1	21.0	847	84.3	62.6	73.7
Jhajjar	709	22.2	24.1	847	83.3	59.9	72.5
Mahendragarh	813	13.5	19.1	918	85.3	54.1	69.9
Rewari	765	17.8	25.2	899	89.0	60.8	75.2
Gurgaon	1,660	22.2	44.6	873	77.1	47.8	62.9
Faridabad	2,195	55.7	48.5	839	82.5	56.3	70.0
Mewat	-	-	-	-	-	-	-

Source: Primary Census Abstract, Series 20, Census of India, 2001.
[1] 1991-2001
[2] Females per 1,000 males.

BACKGROUND CHARACTERISTICS OF HOUSEHOLDS

TABLE 2.1 HOUSEHOLD POPULATION BY AGE AND SEX

Percent distribution of the household population by age, residence and sex, Haryana, 2007-08

	Total			Rural			Urban		
Age	Total	Male	Female	Total	Male	Female	Total	Male	Female
<1	2.1	2.1	2.0	2.2	2.2	2.1	1.9	2.0	1.8
1-4	7.8	8.2	7.5	7.9	8.4	7.5	7.5	7.8	7.3
5-9	10.7	11.3	10.1	11.1	11.8	10.4	9.6	10.0	9.2
10-14	11.5	11.7	11.3	11.9	12.1	11.7	10.3	10.6	10.1
15-19	11.2	11.8	10.6	11.4	11.9	11.0	10.5	11.4	9.5
20-24	10.4	10.1	10.7	10.2	9.9	10.5	11.0	10.6	11.4
25-29	8.2	7.8	8.6	7.9	7.5	8.4	9.0	8.7	9.3
30-34	6.8	6.2	7.4	6.6	6.0	7.2	7.3	6.9	7.8
35-39	6.5	6.4	6.6	6.4	6.3	6.5	6.8	6.7	7.0
40-44	5.2	5.1	5.3	5.0	5.0	5.1	5.7	5.5	5.8
45-49	4.7	4.8	4.6	4.5	4.6	4.4	5.3	5.4	5.2
50-54	3.1	3.4	2.7	2.9	3.2	2.5	3.7	4.0	3.3
55-59	2.8	2.5	3.0	2.6	2.4	2.9	3.1	3.0	3.3
60-64	3.1	2.8	3.4	3.2	2.9	3.5	2.8	2.4	3.3
65-69	2.2	2.0	2.4	2.2	2.0	2.5	2.1	2.0	2.2
70-74	1.8	1.8	1.8	1.8	1.9	1.8	1.6	1.5	1.7
75-79	0.8	0.9	0.8	0.9	0.9	0.8	0.8	0.8	0.8
80+	1.2	1.2	1.2	1.2	1.3	1.2	0.9	0.8	1.0
Below 15	32.2	33.3	30.9	33.1	34.5	31.7	29.3	30.4	28.4
Number of persons**	1,17,052	61,386	55,666	87,695	45,875	41,820	29,357	15,511	13,846
Sex ratio[1]	907	na	na	912	na	na	893	na	na

Note: Table is based on the *de facto* population, i.e. persons who stayed in the household the night before the interview (including both usual residents and visitors).
na = Not applicable.
** Unweighted cases.
[1] Females per 1000 males.

TABLE 2.2 MARITAL STATUS OF THE HOUSEHOLD POPULATION

Percent distribution of the household population (age10 years and above) by marital status, age and sex, Haryana, 2007-08

Age	Marital status: Never married	Currently married	Married, *gauna* not performed	Widowed/ divorced/ separated	Total percent	Number of persons**
**Total**						
10-14	99.5	0.2	0.2	0.0	100.0	13,452
15-19	87.7	11.0	1.1	0.1	100.0	13,124
20-24	43.2	55.8	0.4	0.5	100.0	12,135
25-29	12.0	86.7	0.1	1.3	100.0	9,573
30-44	2.2	94.3	0.0	3.5	100.0	21,605
45-59	1.1	89.0	0.0	9.9	100.0	12,348
60+	1.0	67.6	0.0	31.4	100.0	10,603
Total	34.5	59.3	0.3	5.9	100.0	92,840
**Male**						
10-14	99.6	0.2	0.2	0.0	100.0	7,178
15-19	95.8	3.1	1.0	0.0	100.0	7,218
20-24	64.0	34.9	0.7	0.3	100.0	6,175
25-29	21.1	78.0	0.1	0.8	100.0	4,772
30-44	4.0	94.4	0.0	1.6	100.0	10,878
45-59	1.8	93.9	0.0	4.3	100.0	6,592
60+	1.8	81.5	0.0	16.7	100.0	5,276
Total	40.9	55.9	0.3	2.9	100.0	48,089
**Female**						
10-14	99.4	0.2	0.3	0.1	100.0	6,274
15-19	77.8	20.7	1.2	0.3	100.0	5,906
20-24	21.7	77.4	0.1	0.8	100.0	5,960
25-29	2.9	95.3	0.0	1.8	100.0	4,801
30-44	0.4	94.2	0.0	5.5	100.0	10,727
45-59	0.2	83.3	0.0	16.4	100.0	5,756
60+	0.2	53.9	0.0	45.9	100.0	5,327
Total	27.5	63.0	0.2	9.2	100.0	44,751

** Unweighted cases.

TABLE 2.3 AGE AT MARRIAGE

Mean age at marriage and percentage of marriages below legally prescribed minimum age at marriage by sex, residence and districts, Haryana, 2007-08

Place of residence/district	Mean age at marriage		Percentage of marriages below legal age at marriage		Currently married women aged 20-24 who were married before age 18
	Boys	Girls	Boys (<21 years)	Girls (<18 years)	
**District**					
Panchkula	24.3	21.9	17.3	3.4	16.6
Ambala	24.7	21.7	14.4	2.9	6.0
Yamunanagar	23.2	21.3	16.6	3.8	10.0
Kurukshetra	23.4	21.2	20.4	4.4	9.7
Kaithal	22.9	20.1	25.5	12.8	22.5
Karnal	23.0	20.1	23.4	10.7	23.9
Panipat	22.4	20.0	27.2	8.9	28.8
Sonipat	23.3	20.0	19.6	4.4	21.0
Jind	23.4	19.0	19.7	20.2	39.8
Fatehabad	22.9	19.5	23.4	17.2	24.0
Sirsa	22.6	20.2	31.0	10.1	21.9
Hisar	22.6	18.8	31.8	25.2	34.3
Bhiwani	22.0	18.9	31.7	20.7	34.7
Rohtak	23.3	19.7	21.4	9.6	31.3
Jhajjar	23.0	19.4	21.4	14.5	26.0
Mahendragarh	22.3	18.3	24.1	33.2	42.6
Rewari	22.4	19.7	22.2	14.2	25.8
Gurgaon	22.5	19.1	32.8	22.4	29.9
Faridabad	23.0	20.0	23.0	15.3	37.4
Mewat	20.0	17.6	62.5	43.2	55.5
Rural	22.3	19.2	30.7	18.5	29.2
Urban	23.9	21.1	17.3	8.2	24.0
**Haryana**	22.7	19.7	27.4	15.9	28.0

Reference period: January 1st, 2004 to survey date.

TABLE 2.4 EDUCATIONAL LEVEL OF THE HOUSEHOLD POPULATION

Percent distribution of household population age 7 years and above by literacy levels and years of schooling, age, residence and sex, Haryana, 2007-08

Age	Non-literate	Years of schooling among those who are literate: Less than 5	6 - 8	9 - 10	11 or more	Missing	Total percent	Number of persons**
**Total**								
7-9	11.8	99.0	0.8	0.0	0.0	0.1	100.0	7,400
10-14	5.4	44.8	46.6	8.3	0.2	0.0	100.0	13,452
15-19	8.2	10.5	26.1	31.3	32.1	0.0	100.0	13,124
20-29	15.3	13.3	24.1	22.6	39.9	0.1	100.0	21,708
30-39	31.9	19.4	24.8	26.6	28.9	0.4	100.0	15,541
40-49	43.1	21.8	26.7	30.4	20.5	0.6	100.0	11,571
50+	61.7	26.9	22.2	30.7	19.2	1.1	100.0	17,444
Total	26.6	28.8	26.4	21.6	23.0	0.2	100.0	1,00,240
**Male**								
7-9	10.3	98.9	0.9	0.0	0.0	0.1	100.0	4,137
10-14	3.6	46.1	45.7	8.0	0.2	0.0	100.0	7,178
15-19	5.1	9.2	25.9	32.9	31.9	0.0	100.0	7,218
20-29	7.9	10.3	22.5	23.2	43.8	0.1	100.0	10,947
30-39	16.6	14.3	23.7	29.1	32.6	0.3	100.0	7,745
40-49	24.6	16.0	27.9	34.0	21.7	0.4	100.0	6,065
50+	43.2	21.3	22.9	34.8	20.1	0.9	100.0	8,936
Total	16.4	25.8	25.6	23.7	24.6	0.2	100.0	52,226
**Female**								
7-9	13.7	99.1	0.8	0.0	0.0	0.1	100.0	3,263
10-14	7.4	43.4	47.8	8.6	0.2	0.0	100.0	6,274
15-19	11.9	12.1	26.4	29.2	32.2	0.1	100.0	5,906
20-29	22.7	16.9	26.0	21.8	35.2	0.1	100.0	10,761
30-39	47.0	27.2	26.4	22.7	23.1	0.5	100.0	7,796
40-49	63.5	35.1	24.1	22.0	17.7	1.1	100.0	5,506
50+	81.1	44.5	20.0	17.7	16.2	1.5	100.0	8,508
Total	37.7	33.1	27.5	18.4	20.7	0.3	100.0	48,014

Contd……

TABLE 2.4 EDUCATIONAL LEVEL OF THE HOUSEHOLD POPULATION—***continued***

Percent distribution of household population age 7 years and above by literacy levels and years of schooling, age, residence and sex, Haryana, 2007-08

Age	Non-literate	Years of schooling among those who are literate: Less than 5	6 - 8	9 - 10	11 or more	Missing	Total percent	Number of persons**
**Rural**								
7-9	12.4	98.9	0.9	0.0	0.0	0.1	100.0	5,744
10-14	5.6	45.5	46.4	7.9	0.2	0.0	100.0	10,427
15-19	8.6	11.0	28.1	31.9	28.9	0.0	100.0	10,035
20-29	17.0	14.9	26.0	23.6	35.3	0.1	100.0	15,833
30-39	36.8	22.5	28.0	27.3	21.8	0.4	100.0	11,380
40-49	49.7	26.4	30.7	29.9	12.5	0.7	100.0	8,351
50+	69.7	31.1	24.8	31.3	11.4	1.4	100.0	13,036
Total	29.8	31.2	28.3	21.4	18.8	0.3	100.0	74,806
**Male**								
7-9	10.4	98.9	0.9	0.0	0.0	0.1	100.0	3,220
10-14	3.5	46.4	45.5	7.9	0.2	0.0	100.0	5,543
15-19	5.1	9.2	27.2	33.6	30.0	0.0	100.0	5,451
20-29	8.6	10.8	23.4	23.9	41.8	0.1	100.0	7,945
30-39	19.0	15.6	26.2	30.4	27.4	0.4	100.0	5,630
40-49	28.4	18.2	32.1	34.7	14.7	0.4	100.0	4,373
50+	50.8	25.0	25.6	35.4	12.7	1.2	100.0	6,688
Total	18.5	27.5	27.2	23.8	21.3	0.2	100.0	38,850
**Female**								
7-9	15.0	98.9	0.9	0.1	0.0	0.1	100.0	2,524
10-14	8.0	44.4	47.4	8.0	0.2	0.0	100.0	4,884
15-19	12.7	13.4	29.3	29.7	27.5	0.1	100.0	4,584
20-29	25.5	20.0	29.3	23.3	27.3	0.1	100.0	7,888
30-39	54.3	34.5	31.0	21.9	12.1	0.5	100.0	5,750
40-49	73.1	50.3	26.6	15.7	5.8	1.6	100.0	3,978
50+	89.8	61.7	20.8	10.2	4.7	2.6	100.0	6,348
Total	42.0	36.8	30.1	17.9	15.0	0.3	100.0	35,956

Contd......

TABLE 2.4 EDUCATIONAL LEVEL OF THE HOUSEHOLD POPULATION—*continued*

Percent distribution of household population age 7 years and above by literacy levels and years of schooling, age, residence and sex, Haryana, 2007-08

Age	Non-literate	Years of schooling among those who are literate: Less than 5	6 - 8	9 - 10	11 or more	Missing	Total percent	Number of persons**
**Urban**								
7-9	9.6	99.3	0.5	0.0	0.1	0.1	100.0	1,656
10-14	4.7	42.5	47.5	9.7	0.3	0.0	100.0	3,025
15-19	6.8	8.6	19.8	29.5	42.1	0.0	100.0	3,089
20-29	10.5	9.3	19.2	19.9	51.4	0.1	100.0	5,875
30-39	18.3	12.7	18.0	25.2	43.8	0.2	100.0	4,161
40-49	25.9	13.8	19.8	31.3	34.7	0.5	100.0	3,220
50+	38.0	20.8	18.3	29.8	30.4	0.6	100.0	4,408
Total	17.3	22.7	21.6	21.8	33.6	0.2	100.0	25,434
**Male**								
7-9	10.0	99.0	0.7	0.0	0.1	0.1	100.0	917
10-14	4.2	44.9	46.3	8.6	0.2	0.0	100.0	1,635
15-19	5.2	9.3	21.9	31.0	37.8	0.0	100.0	1,767
20-29	6.0	9.2	20.0	21.6	49.1	0.2	100.0	3,002
30-39	10.2	11.2	17.7	26.0	44.9	0.1	100.0	2,115
40-49	14.7	11.3	18.8	32.6	36.9	0.3	100.0	1,692
50+	20.8	14.4	17.7	33.6	33.8	0.5	100.0	2,248
Total	10.2	21.4	21.5	23.5	33.4	0.2	100.0	13,376
**Female**								
7-9	9.1	99.7	0.3	0.0	0.0	0.0	100.0	739
10-14	5.3	39.7	49.0	10.9	0.4	0.0	100.0	1,390
15-19	8.9	7.7	16.9	27.5	47.9	0.0	100.0	1,322
20-29	15.2	9.4	18.3	18.0	54.2	0.1	100.0	2,873
30-39	26.7	14.6	18.4	24.2	42.5	0.4	100.0	2,046
40-49	38.4	17.7	21.2	29.2	31.2	0.6	100.0	1,528
50+	55.8	32.9	19.4	22.9	24.1	0.7	100.0	2,160
Total	25.1	24.5	21.7	19.6	33.9	0.2	100.0	12,058

Note: Years of schooling is applicable to those who are literate.
** Unweighted cases.

TABLE 2.5 CURRENTLY ATTENDING SCHOOL

Percentage of household population (age less than 18 years) attending school/college , Haryana, 2007-08

	Total			Male			Female		
Age (years)	Rural	Urban	Total	Rural	Urban	Total	Rural	Urban	Total
6-10	99.2	99.6	99.3	99.5	99.7	99.5	98.9	99.4	99.0
11-13	94.8	96.1	95.1	96.2	96.1	96.2	93.2	96.2	93.9
14-17	77.2	82.1	78.3	81.6	81.4	81.5	71.5	83.2	74.0
6-11	99.0	99.4	99.1	99.4	99.3	99.4	98.6	99.5	98.8
12-17	83.2	86.9	84.0	86.4	86.1	86.4	79.3	87.9	81.2
Total	91.5	93.5	92.0	93.3	93.1	93.2	89.4	94.0	90.4

TABLE 2.6 REASONS FOR DROPPING OUT OF SCHOOL

Percent distribution of household population age less than 18 years who dropped out of school by main reasons, Haryana, 2007-08

	Total		Rural		Urban	
Reasons	Male	Female	Male	Female	Male	Female
School too far	1.2	8.0	1.5	8.7	0.0	3.6
Transport not available	0.3	0.4	0.3	0.5	0.0	0.0
Further education not necessary	7.0	8.5	5.9	8.4	10.9	9.5
Required for household work	11.3	15.6	11.3	16.0	11.2	13.7
Required for work on family/business	3.3	1.9	3.4	1.8	3.2	2.6
Required for outside work	11.5	2.9	10.5	2.4	14.8	6.1
Cost too much	11.4	17.2	11.4	16.3	11.7	22.9
No proper school	0.0	3.1	0.0	3.6	0.0	0.0
Not safe to send girls	0.1	1.7	0.1	1.7	0.0	1.6
For taking care of siblings	9.2	6.0	9.5	5.9	8.3	6.9
Not interested in studies	38.4	22.5	38.9	22.3	36.7	24.2
Repeated failures	2.3	3.1	2.3	3.2	2.1	2.7
Got married	0.0	3.4	0.0	3.4	0.0	3.2
Others	4.1	5.6	5.0	6.0	1.0	3.1
Total percent	100.0	100.0	100.0	100.0	100.0	100.0
Number of persons**	1,196	1,351	919	1,160	277	191

**Unweighted cases.

TABLE 2.7 HOUSEHOLD CHARACTERISTICS

Percent distribution of the households by selected characteristics of the household head, household size and residence, Haryana, 2007-08

Characteristics	Total	Residence	
		Rural	Urban
**Sex**			
Male	91.5	91.6	91.3
Female	8.5	8.4	8.7
**Age (years)**			
< 30	10.8	10.7	11.1
30-44	37.4	38.1	35.6
45-59	30.1	28.9	33.5
60+	21.7	22.3	19.8
**Median age**	45.0	45.0	45.0
**Religion**			
Hindu	87.1	85.5	91.5
Muslim	6.4	7.7	3.1
Christian	0.1	0.1	0.2
Sikh	6.3	6.8	5.0
Buddhist	0.0	0.0	0.0
Jain	0.0	0.0	0.2
Others	0.0	0.0	0.0
**Castes/tribes**			
Scheduled castes	24.3	25.9	20.0
Scheduled tribes	0.4	0.4	0.4
Other backward classes	34.7	36.5	29.8
Others	40.6	37.2	49.8
**Number of usual members**			
1	2.3	1.8	3.7
2	6.1	5.7	7.0
3	9.4	8.8	11.0
4	19.8	18.9	22.4
5	21.1	21.0	21.4
6	15.7	16.0	14.8
7	9.5	10.2	7.7
8	5.7	6.3	4.2
9+	10.4	11.4	7.8
Total percent	100	100	100
Mean household size	5.5	5.6	5.1
Number of households**	21,406	15,615	5,791

Note: Total figure may not add to 100 percent due to ‘multiple responses’.
**Unweighted cases.

TABLE 2.8 HOUSING CHARACTERISTICS AND ASSETS

Percent distribution of the households by housing characteristics and selected durable goods, by residence, Haryana 2007-08

Housing characteristics	Total	Residence	
		Rural	Urban
**Electricity**			
Having electricity connection	92.4	90.5	97.5
**Source of drinking water**			
**Improved source**	96.0	94.7	99.5
Piped water into dwelling/yard/plot	48.7	40.3	71.1
Public tap/standpipe	11.4	12.9	7.3
Tube Well or borehole	12.6	14.4	7.7
Other improved	23.4	27.0	13.5
**Non improved sources**	4.0	5.3	0.5
**Sanitation facility**			
**Improved sanitation**	50.7	39.0	82.1
Flush to sewer/septic/pit	46.8	34.4	79.9
Pit with slab	3.3	3.9	1.7
Pit ventilated improved	0.5	0.6	0.4
Other	0.1	0.1	0.1
**Not improved**	49.3	61.0	17.9
Flush not to sewer/septic/pit/twin pit	1.3	0.7	2.7
Pit without slab	4.3	5.5	0.9
Dry toilet	0.0	0.0	0.1
No toilet/open space	43.7	54.7	14.1
**Fuel used for cooking**			
Liquefied Petroleum Gas (LPG)	26.3	11.0	67.8
Electricity	0.2	0.1	0.3
Kerosene	0.8	0.4	1.9
Wood	10.9	11.9	8.1
Others	61.8	76.6	21.9
**Type of house**			
Kachha	4.9	5.8	2.7
Semi - pucca	37.1	45.6	14.2
Pucca	58.0	48.7	83.1
**Number of rooms**			
1	21.2	21.3	21.1
2	20.9	22.3	17.2
3+	57.9	56.5	61.7
**Household assets**			
Fan	86.4	83.6	93.8
Radio/transistor	21.6	21.1	22.9
Sewing machine	47.6	44.3	56.8
Television	60.6	53.3	80.4
Telephone	55.9	50.2	71.3
Bicycle	52.0	51.1	54.6
Motor cycle/ scooter	27.3	23.0	39.0
Car / Jeep	5.5	3.6	10.5
Tractor	6.9	8.8	1.6
**Wealth Index**			
Lowest	1.7	2.2	0.5
Second	7.6	9.8	1.6
Middle	21.7	27.1	7.2
Fourth	31.1	34.1	22.8
Highest	37.9	26.7	67.9
Number of households**	21,406	15,615	5,791

**Unweighted cases.

TABLE 2.9 HOUSING CHARACTERISTICS BY DISTRICT

Percentage of household with selected characteristics by district, Haryana, 2007-08

District	With electricity	With drinking water[1]	With toilet facility[2]	Using Liquefied Petroleum Gas	Living in *pucca* house	Having BPL card
Panchkula	94.2	98.0	63.1	48.0	74.5	11.3
Ambala	94.9	99.7	56.4	39.7	62.8	12.1
Yamunanagar	93.9	99.5	49.6	32.5	63.9	16.5
Kurukshetra	94.4	99.6	64.6	31.8	63.0	18.2
Kaithal	94.5	99.3	56.8	20.4	51.1	21.6
Karnal	95.8	98.5	65.7	29.9	62.0	17.2
Panipat	96.5	99.8	76.6	35.8	63.7	14.3
Sonipat	95.6	95.8	59.5	30.1	64.0	12.0
Jind	96.2	91.1	44.9	14.7	38.7	27.8
Fatehabad	87.6	97.2	79.6	21.9	50.7	24.5
Sirsa	85.6	99.5	95.0	26.3	47.3	20.9
Hisar	88.9	89.9	57.5	23.6	54.7	19.8
Bhiwani	91.8	87.1	46.3	17.3	52.7	19.1
Rohtak	97.5	94.1	61.3	34.5	69.7	15.5
Jhajjar	93.9	91.5	51.8	23.0	59.3	14.2
Mahendragarh	89.3	98.4	37.0	15.2	51.7	16.4
Rewari	94.3	95.2	46.0	26.6	67.1	20.7
Gurgaon	95.2	99.6	65.5	49.3	75.6	12.1
Faridabad	90.9	99.5	59.2	46.1	61.9	9.5
Mewat	78.8	88.3	16.2	4.5	34.6	24.7
Haryana	92.4	96.0	56.3	26.3	58.0	18.6

Note: [1] Includes piped into dwelling piped to yard/plot, public tap/stand pipe/hand pump/tube well/bore well/well covered/spring tanker, cart with small tank and bottled water.

[2] Household having access to toilet facility = improved source of sanitation + flush not to sewer/septic/pit/twin pit + pit without slab + dry toilet.

TABLE 2.10 DISTANCE FROM THE NEAREST EDUCATIONAL FACILITY

Percent distribution of sampled villages by distance from the nearest educational facility, Haryana, 2007-08

Educational facility	Within village	Distance from the village[1] < 5 km	5-9 km	10+ km	Total percent
Primary school	98.6	1.2	0.0	0.1	100.0
Middle school	77.4	18.9	3.2	0.6	100.0
Secondary school	51.3	32.9	13.5	2.4	100.0
Higher secondary school	31.5	34.4	22.1	12.1	100.0
College	3.6	9.6	23.3	63.5	100.0
Madarasa	4.9	7.6	7.5	80.0	100.0
Number of villages**	721				

Note: Table based on unweighted cases.
[1] Distance from the village is calculated for only those villages which do not have the facility within the village.

TABLE 2.11 DISTANCE FROM THE NEAREST HEALTH FACILITY

Percent distribution of sampled villages by distance from the nearest health facility, Haryana, 2007-08

Health facility	Within village	Distance from the village[1] Within 3km	Within 5km	Within 10km	More than 10km
Sub-Centre	46.6	77.0	90.6	99.3	0.7
Primary Health Centre	12.6	31.3	50.1	82.3	17.8
Community Health Centre	2.9	11.4	21.5	50.9	49.1
District/Govt. Hospital	1.8	3.9	6.8	18.4	81.6
Government Dispensary	12.9	29.4	41.7	63.1	36.9
Private Clinic	35.5	51.0	62.6	82.0	18.0
Private Hospital	4.0	13.6	25.9	56.0	44.0
AYUSH health facility[2]	9.0	19.0	30.0	52.1	47.9

[1] including facilities within village.

[2] AYUSH- Ayurveda, Yoga and Naturopathy, Unani, siddha and Homeopathy.

TABLE 2.12 AVAILABILITY OF FACILITY AND HEALTH PERSONNEL BY DISTRICT

Percentage of availabilities of facility and health personnel of villages by district, Haryana, 2007-08

District	Percentage of villages with**										
	Primary or middle school	Sub-centre	PHCs	Any government health facility[1]	Doctor	ASHA	Anganwadi workers	JSY beneficiary	VHSC	Aware of Untied fund[2]	Number of villages
Panchkula	100.0	21.4	7.1	28.6	42.9	78.6	96.4	78.6	14.3	32.1	28
Ambala	96.9	31.3	6.3	37.5	34.4	78.1	100.0	50.0	15.6	65.6	32
Yamunanagar	96.8	9.7	6.5	12.9	29.0	51.6	96.8	48.4	9.7	45.2	31
Kurukshetra	100.0	43.2	8.1	43.2	54.1	75.7	97.3	54.1	21.6	21.6	37
Kaithal	100.0	60.0	20.0	60.0	35.0	92.5	100.0	72.5	22.5	30.0	40
Karnal	100.0	32.4	18.9	40.5	54.1	83.8	100.0	70.3	13.5	40.5	37
Panipat	100.0	50.0	16.7	53.3	46.7	90.0	100.0	50.0	50.0	40.0	30
Sonipat	100.0	62.2	10.8	64.9	45.9	86.5	100.0	73.0	13.5	18.9	37
Jind	97.5	57.5	17.5	60.0	27.5	87.5	97.5	82.5	20.0	60.0	40
Fatehabad	100.0	68.3	9.8	68.3	12.2	92.7	100.0	85.4	29.3	61.0	41
Sirsa	100.0	48.6	13.5	56.8	21.6	91.9	97.3	86.5	45.9	81.1	37
Hisar	100.0	81.1	21.6	81.1	16.2	86.5	97.3	91.9	37.8	37.8	37
Bhiwani	97.6	56.1	22.0	56.1	12.2	61.0	100.0	63.4	2.4	22.0	41
Rohtak	100.0	78.1	21.9	78.1	18.8	81.3	100.0	65.6	6.3	34.4	32
Jhajjar	100.0	71.8	20.5	71.8	5.1	97.4	100.0	97.4	28.2	33.3	39
Mahendragarh	100.0	30.2	7.0	30.2	11.6	79.1	97.7	62.8	20.9	16.3	43
Rewari	100.0	41.5	4.9	43.9	19.5	90.2	97.6	65.9	19.5	36.6	41
Gurgaon	100.0	25.0	0.0	25.0	21.9	90.6	93.8	46.9	6.3	43.8	32
Faridabad	100.0	25.0	10.0	25.0	0.0	90.0	100.0	65.0	0.0	25.0	20
Mewat	95.7	19.6	6.5	26.1	13.0	47.8	93.5	45.7	15.2	19.6	46
Haryana	99.2	46.6	12.6	49.1	25.8	81.3	98.2	68.2	20.1	38.0	721

** Facilities as reported by village pradhan/up pradhan/any other panchayat member/teacher/gram sevak/aganwadi worker. [1] Includes Sub-Centre, Primary Health Centre (including Block PHC), Community Health Centre or referral hospital, government hospital, and government dispensary within the village. [2] This information was collected from Sarpanch/Pradhan, PRI member, Gram Sevak, Village Secretary/officer or any other official at village level.VHSC = Village Health and Sanitation Committee.

Note: Table is based on unweighted cases.

TABLE 2.13 KNOWLEDGE ABOUT GOVERNMENT HEALTH PROGRAMMES

Percentage of ever married women (age 15-49 years) who heard about Government health programmes and among them, who received information from specific sources, Haryana, 2007-08.

Health programmes	Women aware about programmes	Sources of knowledge: T.V.	Radio	News paper /books, etc.	Drama/Song/ Dance, etc.	Exhibition/ Mela	Group meeting/ Pro-grammes	Doctor/ANM/AWW/ ASHA	Friends/ relatives	Others	Number of women**
Antenatal care	95.7	50.1	7.4	14.8	0.1	0.2	2.2	68.3	63.3	0.5	20,571
Institutional Delivery	84.5	50.7	7.5	14.0	0.1	0.2	2.3	60.1	63.7	0.4	18,167
Breastfeeding	89.8	57.8	9.2	17.7	0.2	0.2	2.6	55.8	61.5	0.7	19,309
Immunization	99.1	55.5	9.1	17.1	0.2	0.2	2.8	79.2	59.9	0.6	21,301
Family Planning	98.6	60.6	10.4	21.1	0.2	0.3	2.5	58.0	66.2	1.2	21,188
Personal hygiene	92.8	60.0	9.5	18.7	0.2	0.2	1.8	35.9	59.8	1.4	19,931
Sanitation/Safe drinking water	94.4	61.9	10.2	18.1	0.2	0.2	2.0	40.8	57.8	1.1	20,286
Prevention of Sex Selection	89.4	62.4	9.2	20.4	0.3	0.3	2.4	43.6	62.4	1.4	19,249
Awareness about Government health programmes (based on response from household questionnaire)											Number of Household**
DOTS (Tuberculosis)	39.6	74.6	15.1	36.6	0.3	0.4	2.0	31.5	28.8	2.2	8,461
Leprosy eradication	34.0	80.0	16.6	42.3	0.4	0.5	2.4	24.6	24.5	1.9	7,272
Malaria/Dengue/Chikun Guinea	84.9	59.4	14.0	27.8	0.2	0.2	1.9	47.2	44.5	1.1	18,179
Prevention of sex selection	84.5	63.8	14.1	36.0	0.7	0.4	2.4	33.1	52.5	2.6	18,095

**Unweighted cases.

CHARACTERISTICS OF WOMEN AND FERTILITY

TABLE 3.1 BACKGROUND CHARACTERISTICS OF EVERMARRIED WOMEN

Percent distribution of ever married women aged 15-49 years according to selected background characteristics, and place of residence, Haryana, 2007-08

Background characteristics	Place of residence		
	Total	Rural	Urban
**Age group**			
15-19	5.8	6.6	3.5
20-24	18.7	19.3	17.0
25-29	18.8	18.5	19.6
30-34	18.2	18.0	19.0
35-39	15.6	15.5	15.9
40-44	13.1	12.8	14.0
45-49	9.7	9.2	11.0
**Age at consummation of marriage**			
Below 18 years	37.2	40.0	28.7
18 years & above	62.8	60.0	71.3
**Marital duration**			
Less than 5 years	20.0	19.9	20.1
5-9 years	16.7	16.5	17.2
10-14 years	16.3	15.9	17.2
15 or more years	47.1	47.6	45.5
**Woman's education**			
Non-literate[a]	41.8	46.8	26.5
Less than 5 years	4.3	4.6	3.3
5-9 years	29.7	30.5	27.1
10 or more years	24.2	18.1	43.0
Non-literate[a †]	38.8	43.4	24.9
10 or more years[†]	25.9	19.9	44.5
**Husband's education**			
Non-literate[a]	18.1	20.4	10.9
Less than 5 years	3.6	3.6	3.6
5-9 years	32.2	33.8	27.2
10 or more years	45.9	41.9	58.1
**Religion**			
Hindu	86.6	84.8	92.2
Muslim	7.2	8.7	2.7
Sikh	6.0	6.4	4.7
Others	0.1	0.1	0.3
**Castes/tribes**			
Scheduled castes	22.8	23.4	21.2
Scheduled tribes	0.4	0.4	0.4
Other backward classes	36.1	37.6	31.5
Others	40.7	38.6	46.9
**Wealth index**			
Lowest	1.3	1.6	0.3
Second	6.0	7.6	1.2
Middle	19.7	24.2	6.0
Fourth	31.2	34.5	20.9
Highest	41.8	32.0	71.5
**Number of women (15-49)****	21,484	16,154	5,330
**Number of women (15-44)**** [†]	18,575	14,009	4,566

**Unweighted cases.
[a] Literates but did not attend school, are also included.
[†] Represents figures for currently married women aged 15-44 years.

TABLE 3.2 LEVEL OF EDUCATION OF EVER MARRIED WOMEN

Percent distribution of ever married women aged 15-49 years according to selected background characteristics and years of schooling, Haryana, 2007-08

Background characteristics	Non-literate	Literate but no schooling	Years of schooling 0 -5 years	6-8 years	9-10 years	11 or more years	Total percent	Number of women**
**Age group**								
15-19	32.7	0.3	13.6	26.7	15.0	11.5	100.0	1,236
20-24	26.4	0.5	11.2	22.2	19.5	19.9	100.0	4,012
25-29	31.8	0.4	11.5	21.3	15.7	19.1	100.0	4,032
30-34	44.0	0.7	12.5	14.7	14.4	13.4	100.0	3,918
35-39	56.6	0.9	12.5	11.8	9.4	8.6	100.0	3,364
40-44	65.5	0.7	10.6	9.2	7.9	5.9	100.0	2,830
45-49	66.8	1.1	9.8	8.0	6.9	6.8	100.0	2,093
**Place of residence**								
Rural	50.2	0.6	12.4	16.3	11.7	8.5	100.0	16,154
Urban	28.4	0.7	9.3	16.0	18.0	27.5	100.0	5,330
**Husband's education**								
Non-literate[a]	85.6	1.0	7.5	4.3	1.0	0.2	100.0	3,856
Less than 5 years	69.4	1.3	12.8	12.9	2.3	0.8	100.0	765
5-9 years	51.7	0.7	16.9	20.0	8.1	2.3	100.0	6,896
10 or more years	21.7	0.4	9.5	18.5	22.7	27.0	100.0	9,909
**Religion**								
Hindu	42.0	0.7	11.9	17.1	14.0	14.2	100.0	18,725
Muslim	84.8	0.8	6.2	3.6	2.0	1.0	100.0	1,419
Sikh	36.1	0.5	14.0	19.0	15.9	14.1	100.0	1,315
Others	23.5	0.0	3.9	11.7	38.8	22.1	100.0	26
**Castes/tribes**								
Scheduled castes	60.0	0.9	12.3	14.6	7.9	4.2	100.0	4,924
Scheduled tribes	55.8	0.0	7.1	21.1	8.1	7.9	100.0	87
Other backward classes	49.2	0.7	11.9	16.5	11.8	9.4	100.0	7,684
Others	32.1	0.5	11.1	16.8	17.6	21.7	100.0	8,790
**Wealth index**								
Lowest	87.3	1.2	4.6	2.6	0.7	0.4	100.0	258
Second	82.9	1.1	7.8	5.8	1.2	0.5	100.0	1,270
Middle	71.1	0.6	11.0	11.7	4.1	1.2	100.0	4,213
Fourth	50.9	0.7	14.9	18.0	10.3	5.0	100.0	6,731
Highest	21.0	0.5	10.3	18.9	22.0	27.2	100.0	9,012
Haryana	44.8	0.7	11.6	16.2	13.3	13.2	100.0	21,484

Note: Total includes women with missing information on husband's education, who are not shown separately.
** Unweighted cases.
[a] Literates but did not attend school, are also included.

TABLE 3.3 BIRTH ORDER

Percent distribution of births[#] among ever married women aged 15-49 years according to selected background characteristics and birth order, Haryana, 2007-08

Background characteristics	Distribution of births[†]	Birth order 1	2	3	4+	2 & above[†]	Total	Number of births**
**Age group**								
15-19	5.0	84.4	13.9	1.7	0.0	16.1	100.0	454
20-24	42.8	49.9	34.7	11.9	3.6	47.4	100.0	3,206
25-29	33.4	20.2	33.0	23.8	23.0	77.4	100.0	2,041
30-34	13.4	6.8	18.9	20.0	54.4	92.2	100.0	748
35-39	4.2	4.4	5.4	8.2	82.0	95.8	100.0	211
40-44	1.2	3.1	3.3	1.3	92.3	97.4	100.0	58
45-49	na	*	*	*	*	na	100.0	8
**Place of residence**								
Rural	76.2	35.5	28.3	15.7	20.4	65.6	100.0	5,132
Urban	23.8	39.1	33.9	14.7	12.4	61.0	100.0	1,594
**Education**								
Non-literate[a]	36.6	21.6	21.7	18.5	38.1	78.6	100.0	2,306
Less than 5 years	4.6	27.0	29.5	22.0	21.5	70.6	100.0	305
5-9 years	31.8	38.5	34.2	17.0	10.3	61.5	100.0	2,179
10 or more years	27.1	53.7	34.3	8.9	3.1	48.0	100.0	1,936
**Religion**								
Hindu	80.9	38.7	31.6	16.2	13.5	62.4	100.0	5,501
Muslim	14.8	21.2	16.9	13.5	48.4	78.9	100.0	910
Sikh	4.3	44.4	36.3	9.4	9.8	55.7	100.0	308
Others	0.1	*	*	*	*	66.7	100.0	7
**Caste/tribes**								
Scheduled castes	25.4	31.9	27.3	19.7	21.1	68.9	100.0	1,737
Scheduled tribes	0.6	31.8	16.1	24.8	27.3	70.2	100.0	41
Other backward classes	40.9	33.6	27.5	15.2	23.8	67.3	100.0	2,701
Others	33.1	43.4	34.4	12.4	9.8	57.7	100.0	2,247
**Wealth index**								
Lowest	2.3	15.9	17.8	14.2	52.1	83.9	100.0	141
Second	7.8	20.3	17.1	19.1	43.5	79.9	100.0	483
Middle	22.6	24.8	24.3	19.4	31.5	75.2	100.0	1,465
Fourth	31.3	36.7	30.8	16.9	15.5	64.0	100.0	2,153
Highest	36.0	47.5	35.1	11.2	6.2	53.6	100.0	2,484
	100.0	-	-	-	-	-	-	-
**Haryana**	9,595	36.4	29.6	15.5	18.5	64.5	100.0	6,726

[#] Women who had their last live/still birth since 01-01-2004.
* Percentage not shown; based on less than 10 unweighted cases.
** Unweighted cases.
[a] Literates but did not attend school, are also included.
[†] Represents figures for currently married women aged 15-44 years.

TABLE 3.4 BIRTH ORDER DISTRIBUTION BY DISTRICT

Percent distribution of births[#] to ever married women aged 15-49 years by birth order and district, Haryana, 2007-08

District	Birth order 1	2	3	4+	Number of births**
Panchkula	38.7	36.3	14.1	10.9	296
Ambala	45.3	33.0	14.5	7.2	226
Yamunanagar	46.4	25.7	17.9	10.0	289
Kurukshetra	39.8	35.7	14.8	9.7	282
Kaithal	37.0	34.6	15.1	13.4	336
Karnal	42.4	32.4	15.1	10.1	332
Panipat	35.5	28.3	17.6	18.7	360
Sonipat	46.2	30.4	12.2	11.3	307
Jind	35.0	30.1	20.3	14.5	302
Fatehabad	37.9	32.8	15.0	14.2	320
Sirsa	40.5	33.0	13.7	12.9	287
Hisar	39.7	32.1	14.4	13.8	265
Bhiwani	32.0	31.2	21.2	15.6	318
Rohtak	39.2	32.8	16.1	11.9	317
Jhajjar	42.7	30.6	14.6	12.1	271
Mahendragarh	37.7	36.7	12.5	13.2	273
Rewari	38.2	35.1	13.5	13.2	308
Gurgaon	34.4	30.9	17.8	16.8	431
Faridabad	37.8	30.3	12.9	19.0	380
Mewat	19.2	15.8	15.9	49.1	826
Haryana	36.4	29.6	15.5	18.5	6,726

[#] Women who had their last live/still birth since 01-01-2004.
** Unweighted cases.

TABLE 3.5 CHILDREN EVER BORN

Mean children ever born (MCEB) according to selected background characteristics of ever married women aged 15-49 years and 40-49 years, Haryana, 2007-08

Background characteristics	Mean children ever born to women age 15-49 years				Mean children ever born to women age 40-49 years				Mean children ever born to currently married women aged 40-44 years
	Total	Males	Females	Number of women**	Total	Males	Females	Number of women**	
**Age group**									
15-19	0.42	0.22	0.19	1,236	na	na	na	na	na
20-24	1.32	0.70	0.62	4,012	na	na	na	na	na
25-29	2.35	1.27	1.08	4,032	na	na	na	na	na
30-34	3.07	1.68	1.39	3,918	na	na	na	na	na
35-39	3.49	1.89	1.60	3,364	na	na	na	na	na
40-44	3.83	2.08	1.76	2,830	3.83	2.08	1.76	2,830	3.84
45-49	4.10	2.18	1.91	2,093	4.10	2.18	1.91	2,093	na
**Residence**									
Rural	2.80	1.50	1.29	16,155	4.11	2.18	1.92	3,568	4.00
Urban	2.52	1.39	1.13	5,330	3.51	1.95	1.56	1,355	3.41
**Education**									
Non-literate[a]	3.59	1.91	1.68	8,928	4.37	2.33	2.04	3,136	4.33
Less than 5 years	2.80	1.51	1.29	925	3.82	2.12	1.70	164	3.53
5-9 years	2.32	1.26	1.06	6,384	3.44	1.88	1.56	981	3.26
10 or more years	1.71	0.97	0.75	5,248	2.65	1.47	1.18	642	2.55
**Religion**									
Hindu	2.65	1.44	1.21	1,8725	3.82	2.06	1.76	4,313	3.69
Muslim	3.99	2.07	1.91	1,419	6.97	3.66	3.32	240	6.94
Sikh	2.32	1.30	1.03	1,315	3.19	1.78	1.42	363	3.01
Others	2.60	1.20	1.40	26	*	*	*	7	2.50
**Castes/tribes**									
Scheduled castes	3.05	1.62	1.43	4,924	4.58	2.42	2.15	1,072	4.40
Scheduled tribes	3.26	1.65	1.61	87	(4.61)	(2.67)	(1.94)	18	4.90
Other backward classes	2.86	1.54	1.32	7,684	4.25	2.29	1.96	1,677	4.18
Others	2.42	1.33	1.09	8,790	3.38	1.83	1.55	2,156	3.28
**Wealth index**									
Lowest	4.00	1.93	2.07	258	6.02	2.45	3.57	52	5.73
Second	3.67	1.92	1.75	1,270	5.21	2.65	2.55	254	5.20
Middle	3.32	1.74	1.58	4,213	4.71	2.48	2.23	907	4.53
Fourth	2.75	1.48	1.27	6,731	4.13	2.20	1.93	1,433	4.03
Highest	2.26	1.26	0.99	9,012	3.32	1.86	1.46	2,277	3.17
Haryana	2.73	1.47	1.25	21,485	3.94	2.12	1.82	4,923	3.84

Note: Total figure may not add to N, sample total, due to ‘don’t know’ or ‘missing cases’.
na = Not applicable.
( ) Based on 10-24 unweighted cases.
* Percentage not shown; based on less than 10 unweighted cases.
** Unweighted cases.
[a] Literates but not attend school, are also included.

TABLE 3.6 FERTILITY PREFERENCES

Percent distribution of currently married women aged 15-49 years by desire for additional child, by number of surviving children, Haryana, 2007-08

	Number of surviving children					
Desire for children	0	1	2	3	4+	Total
**Desire for additional/next child**						
Want another soon[1]	53.1	20.3	3.3	2.0	1.4	10.5
Want another later[2]	33.7	37.9	5.5	2.5	2.6	12.1
Want another, undecided when	1.2	1.3	0.3	0.2	0.2	0.5
Undecided	6.4	4.6	0.8	0.5	0.4	1.8
Want no more	2.3	28.2	45.4	29.9	34.1	32.4
Sterilized[3]	0.0	4.8	42.4	62.4	56.4	39.7
Declared infecund	2.1	1.6	2.1	2.2	4.9	2.6
Inconsistent response	1.2	1.2	0.2	0.2	0.0	0.4
Total percent	100.0	100.0	100.0	100.0	100.0	100.0
Number of women**	2,161	3,038	6,400	4,706	4,089	20,394
Wanting no more children (15-44) †	2.3	29.3	79.5	80.8	82.0	53.4
Number of women (15-44)** †	2,143	2,845	3,484	1,602	1,478	11,552
**Preferred sex of additional/ next child**						
Boy	12.8	37.5	71.3	80.9	55.7	34.4
Girl	4.3	18.2	6.4	4.7	3.8	9.9
Doesn't matter	57.7	30.4	13.5	8.3	26.8	38.2
Up to God	25.2	13.9	8.8	6.1	13.7	17.5
Total percent	100.0	100.0	100.0	100.0	100.0	100.0
Number of women[4]**	1,928	1,842	584	223	164	4,741

** Unweighted cases.
[1] Want next birth within 2 years.
[2] Want to delay next birth for 2 or more years.
[3] Includes both female and male sterilization.
[4] Includes women who want another/next child.
† Represents figures for currently married women aged 15-44 years and It excludes sterilised cases.

TABLE 3.7 OUTCOMES OF PREGNANCY

Percent distribution of all pregnancies of currently married women[#] aged 15-49 years by outcomes according to background characteristics, Haryana, 2007-08

Background characteristics	Live birth	Still birth	Induced abortion	Spontaneous abortion	Total percent	Number of pregnancies**
**Age group**						
15-19	75.6	1.5	3.1	19.7	100.0	600
20-24	84.7	1.8	2.6	10.9	100.0	3,761
25-29	86.5	1.9	3.2	8.4	100.0	2,336
30-34	85.6	1.9	3.6	8.9	100.0	868
35-39	79.6	1.8	5.0	13.6	100.0	263
40-44	93.8	0.0	1.5	4.7	100.0	62
45-49	(66.7)	(8.3)	(8.3)	(16.7)	100.0	12
**Sex-composition of living children [1]**						
One son only	84.9	2.0	3.3	9.8	100.0	1,320
One daughter only	83.0	1.9	2.2	12.9	100.0	1,147
One son, one daughter only	92.8	0.6	2.3	4.2	100.0	1,392
Two sons only	90.3	0.8	2.9	6.0	100.0	681
Three sons only	97.0	1.0	0.9	1.2	100.0	100
Three daughters only	89.6	0.6	1.7	8.1	100.0	169
Four and above	91.0	2.1	1.9	5.0	100.0	1,160
**Woman's education**						
Non-literate[a]	87.6	2.1	1.9	8.4	100.0	2,623
Less than 5 years	85.3	1.9	3.0	9.9	100.0	352
5-9 years	82.0	2.0	3.4	12.6	100.0	2,631
10 or more years	83.7	1.2	3.8	11.2	100.0	2,296
**Husband's education**						
Non-literate[a]	87.5	2.1	2.2	8.1	100.0	1,209
Less than 5 years	86.2	2.5	1.6	9.7	100.0	310
5-9 years	84.9	2.0	2.8	10.4	100.0	2,678
10 or more years	83.1	1.5	3.6	11.8	100.0	3,692
**Religion**						
Hindu	83.8	1.6	3.3	11.2	100.0	6,511
Muslim	88.1	2.8	0.7	8.5	100.0	1,027
Sikh	85.2	1.9	5.1	7.8	100.0	357
Others	*	*	*	*	100.0	7
**Castes/tribes**						
Scheduled castes	83.2	2.2	2.8	11.8	100.0	2,070
Scheduled tribes	97.3	0.0	0.0	2.7	100.0	42
Other backward classes	85.6	2.1	2.9	9.4	100.0	3,135
Others	84.0	1.1	3.4	11.5	100.0	2,655
**Wealth index**						
Lowest	88.0	3.7	1.0	7.3	100.0	160
Second	85.9	3.5	1.9	8.7	100.0	554
Middle	86.6	2.1	2.1	9.2	100.0	1,684
Fourth	83.8	1.8	3.0	11.3	100.0	2,545
Highest	83.4	1.2	3.8	11.5	100.0	2,959
Haryana	84.5	1.8	3.0	10.7	100.0	7,902

Note: Total includes women with missing information on husband's education, who are not shown separately.
( ) Based on 10-24 unweighted cases.
[#] Women who had their pregnancies since 01-01-2004.
* Percentage not shown; based on less than 10 unweighted cases.
** Unweighted cases.
[a] Literates but did not attend school, are also included.
[1] 475 cases with no child were excluded.

TABLE 3.8 OUTCOMES OF PREGNANCY BY DISTRICTS

Percent distribution of all pregnancies of currently married women# aged 15-49 years by outcomes by districts, Haryana, 2007-08

District	Live birth	Still birth	Induced abortion	Spontaneous abortion	Total percent	Number of pregnancies**
Panchkula	84.2	1.8	3.8	10.2	100.0	339
Ambala	82.4	1.5	7.7	8.4	100.0	265
Yamunanagar	85.6	2.7	2.1	9.6	100.0	328
Kurukshetra	86.9	1.7	2.3	9.0	100.0	319
Kaithal	85.8	0.4	3.8	10.0	100.0	393
Karnal	85.9	2.1	2.6	9.4	100.0	386
Panipat	84.3	3.5	3.6	8.6	100.0	430
Sonipat	84.0	0.8	4.3	10.9	100.0	365
Jind	85.2	0.9	1.9	12.1	100.0	347
Fatehabad	83.5	2.1	2.7	11.7	100.0	386
Sirsa	83.9	1.9	3.9	10.3	100.0	342
Hisar	83.3	0.5	1.5	14.7	100.0	316
Bhiwani	86.7	0.6	2.6	10.1	100.0	363
Rohtak	80.8	1.2	6.0	12.1	100.0	395
Jhajjar	81.8	1.9	3.0	13.3	100.0	327
Mahendragarh	85.3	0.8	2.5	11.4	100.0	310
Rewari	78.7	2.1	4.0	15.2	100.0	386
Gurgaon	81.9	1.0	4.3	12.8	100.0	529
Faridabad	84.1	2.4	3.6	9.9	100.0	450
Mewat	88.7	1.9	0.8	8.5	100.0	926
Haryana	84.5	1.8	3.0	10.7	100.0	7,902

Women who had their pregnancies since 01-01-2004.
** Unweighted cases.

MATERNAL HEALTH CARE

TABLE 4.1 PLACE OF ANTENATAL CHECK-UP

Percentage of women (aged 15-49) [#] who received any antenatal check-up (ANC) during pregnancy by source and place of antenatal check-ups, according to selected background characteristics, Haryana, 2007-08

Background characteristics	Any antenatal check-up[1]	Place of antenatal check-up[b] Government health facility[2]	Private health facility[3]	Community based services[4]	Number of women[**]
**Age group**					
15-19	83.9	48.3	42.4	2.7	395
20-24	91.4	47.4	47.4	4.1	2,768
25-29	88.5	44.7	47.4	3.5	2,251
30-34	82.3	43.7	42.8	3.1	966
35 +	67.8	39.2	33.2	6.2	404
**No. of living children**					
0	90.1	48.2	54.3	3.7	29
1	94.3	44.6	57.9	2.9	2,014
2	92.2	45.6	48.7	3.6	2,163
3	86.4	50.4	36.8	3.5	1,183
4+	70.6	43.0	26.6	6.0	1,395
**Residence**					
Rural	85.1	44.4	41.1	3.8	5,103
Urban	93.7	49.0	58.9	3.6	1,681
**Education**					
Non-literate[a]	71.9	44.9	26.3	6.3	2,293
Less than five years	88.4	51.9	25.4	2.9	295
5-9 years	93.4	50.5	43.6	2.8	2,153
10 or more years	98.4	40.6	67.2	2.7	2,043
**Religion**					
Hindu	91.9	46.9	47.1	3.0	5,638
Muslim	54.5	37.0	23.3	12.1	804
Sikh	95.6	39.3	59.7	3.4	337
Other	*	*	*	*	5
**Castes/tribes**					
Scheduled castes	87.9	53.2	30.7	3.7	1,696
Scheduled tribes	87.9	40.5	33.9	5.9	40
Other backward classes	80.1	44.5	43.9	4.7	2,624
Others	94.7	42.0	57.6	2.9	2,424
**Wealth index**					
Lowest	55.8	36.3	18.0	8.6	129
Second	68.5	47.5	17.9	8.6	481
Middle	77.3	46.6	25.0	4.7	1,444
Fourth	88.4	49.9	38.5	3.2	2,090
Highest	97.0	42.2	64.9	2.9	2,639
**Haryana (15-49)**	87.2	45.7	45.8	3.8	6,784
**Haryana (15-44)**[†]	87.3	45.7	45.8	3.8	6,768

[#] Women who had their last live/still birth since 01-01-2004.
* Percentage not shown, based on less than 10 unweighted cases.
** Unweighted cases.
[1] Antenatal check-up done outside home or at home.
[2] Includes sub-centre, primary health centre, community health centre or rural hospital, urban health centre/ urban health post/ urban family welfare centre, government hospital or dispensary.
[3] Includes private hospital/clinic.
[4] Includes non-governmental hospital/ trust hospital or clinic, own home , parents home, other home and other.
[a] Literates but did not attend school, are also included.
[b] Among those who had received any ANC. Total figure may not add to 100 percent due to multiple responses, 'do not know' and 'missing cases'.
† Represents figure for currently married women aged 15-44 years.

TABLE 4.2 ANTENATAL CARE BY DISTRICT

Percentage of women (aged 15-49) [#] who received any antenatal check-up (ANC) during pregnancy by source and place of antenatal check-ups by districts, Haryana, 2007-08

District	Place of antenatal check-up[a]			Any ANC	Number of women**
	Government[1] health facility	Private[2] health facility	Community[3] based services		
Panchkula	67.6	40.4	0.4	91.9	296
Ambala	37.1	56.3	4.5	84.3	260
Yamunanagar	28.2	60.2	3.9	90.8	296
Kurukshetra	34.3	60.8	4.9	92.0	288
Kaithal	50.9	45.8	5.3	95.1	342
Karnal	39.8	57.5	2.9	90.3	333
Panipat	34.7	63.9	1.5	87.2	368
Sonipat	37.4	51.7	2.4	95.5	331
Jind	50.7	42.2	0.9	93.2	312
Fatehabad	64.8	28.9	4.0	91.0	338
Sirsa	48.8	43.2	3.6	92.8	277
Hisar	55.3	42.9	5.3	92.9	287
Bhiwani	49.0	30.5	1.4	91.0	331
Rohtak	57.4	33.6	2.2	96.6	306
Jhajjar	63.2	43.6	2.9	92.1	298
Mahendragarh	51.9	37.1	1.8	95.2	286
Rewari	47.1	58.4	1.4	96.2	300
Gurgaon	41.5	53.8	2.3	94.1	434
Faridabad	40.3	49.7	6.9	81.5	379
Mewat	33.9	21.1	12.7	54.2	722
**Haryana (15-49)**	45.7	45.8	3.8	87.2	6,784
**Haryana (15-44)**[†]	45.7	45.8	3.8	87.3	6,768

[#] Women who had their last live/still birth since 01-01-2004.
** Unweighted cases.
[1] Includes sub-centre, primary health centre, community health centre or rural hospital, urban health centre/ urban health post/ urban family welfare centre, government hospital or dispensary.
[2] Includes Private hospital/clinic,
[3] Includes non-governmental hospital/ trust hospital or clinic, own home , parents home, other home and other.
[a] Total figure may not add to 100 percent due to ‘do not know’ and ‘missing cases’.
† Represents figure for currently married women aged 15-44 years.

TABLE 4.3 COMPONENTS OF ANTENATAL CHECK-UP

Percentage of women (aged 15-49) # who received specific components of antenatal check-up according to selected background characteristics, Haryana, 2007-08

Background characteristics	Weight measured	Height measured	Blood pressure checked	Blood tested	Urine tested	Abdomen examined	Breast examined	Sonography /ultrasound	Number of women**
**Age group**									
15-19	42.9	5.8	34.8	47.1	42.0	54.9	14.5	36.7	395
20-24	49.5	8.5	46.0	54.2	51.3	61.6	18.7	47.8	2,768
25-29	50.3	10.5	44.7	53.0	49.4	61.3	19.4	43.5	2,251
30-34	45.5	10.3	41.1	45.0	42.3	52.2	18.5	34.8	966
35+	29.6	4.6	21.9	23.4	23.4	32.4	8.8	19.9	404
**No. of living children**									
0	49.0	9.9	51.9	59.9	59.5	65.8	20.8	44.6	29
1	59.2	12.5	57.3	67.0	63.6	70.9	25.4	62.3	2,014
2	54.8	10.5	48.8	56.7	53.4	65.4	21.0	47.6	2,163
3	41.8	7.9	37.2	43.2	39.8	53.5	12.5	31.7	1,183
4+	25.2	2.8	17.5	22.4	20.6	32.3	7.7	14.1	1,395
**Residence**									
Rural	42.6	6.8	36.8	44.9	42.5	53.1	15.1	37.6	5,103
Urban	62.8	15.7	60.6	66.3	61.3	72.7	27.1	55.7	1,681
**Education**									
Non- literate[a]	23.2	2.5	17.0	23.9	23.2	34.4	6.9	16.2	2,293
Less than five years	32.9	5.5	22.9	31.6	30.8	47.4	9.6	23.1	295
5-9 years	50.4	8.3	45.7	54.9	51.5	63.2	18.0	43.6	2,153
10 or more years	75.1	17.9	72.3	78.3	72.6	81.3	32.2	73.4	2,043
**Religion**									
Hindu	52.5	9.9	46.9	54.9	51.6	63.0	19.8	46.3	5,638
Muslim	12.7	2.3	9.9	13.2	13.4	20.8	5.0	8.9	804
Sikh	56.3	11.3	59.3	69.0	61.9	72.1	23.8	59.9	337
Others	*	*	*	*	*	*	*	*	5
**Castes/tribes**									
Scheduled Castes	40.3	7.3	32.1	41.6	38.5	51.6	13.6	29.7	1,696
Scheduled Tribes	48.3	14.6	32.2	44.9	55.8	63.3	14.9	34.6	40
Backward Classes	41.2	7.0	36.3	42.6	42.2	50.3	15.0	36.5	2,624
Others	59.7	12.4	57.5	64.7	58.5	70.8	24.6	57.2	2,424
**Wealth index**									
Lowest	10.9	3.0	5.1	12.6	11.8	20.2	1.5	6.6	129
Second	17.8	1.6	12.0	17.9	17.3	29.1	5.1	9.5	481
Middle	28.5	4.2	20.1	26.9	26.2	38.8	8.6	18.1	1,444
Fourth	41.7	6.4	35.4	45.6	44.6	55.6	13.6	36.0	2,090
Highest	70.4	15.5	68.9	74.8	68.2	77.9	30.1	68.3	2,639
**Haryana (15-49)**	47.6	9.0	42.7	50.2	47.1	58.0	18.0	42.1	6,784
**Haryana (15-44)**†	47.7	9.0	42.8	50.3	47.2	58.1	18.1	42.2	6,768

Note: Percentage may not add to 100.0 due to multiple responses.
# Women who had their last live/still birth since 01-01-2004.
* Percentage not shown, based on less than 10 unweighted cases.
** Unweighted cases.
[a] Literates but did not attend school, are also included.
† Represents figure for currently married women aged 15-44 years.

TABLE 4.4 WOMEN RECEIVED ADVICE DURING ANTENATAL CARE

Percentage of women (aged 15-49) [#] who received advice on different components, according to selected background characteristics, Haryana, 2007-08

Background characteristics	Breast feeding	Keep baby warm	Cleanliness	Spacing	Limiting	Nutrition	Institutional delivery	Number of women**
**Age group**								
15-19	41.2	28.7	27.3	20.2	18.2	45.0	38.8	395
20-24	45.7	31.8	34.3	26.7	25.4	49.3	40.7	2,768
25-29	48.3	34.5	35.6	29.1	28.9	51.3	41.4	2,251
30-34	44.9	31.4	34.0	28.1	30.6	46.4	39.0	966
35+	35.0	24.4	24.5	21.1	24.3	38.6	30.2	404
**No. of living children**								
0	43.0	32.1	24.6	17.1	17.1	31.8	40.1	29
1	51.9	36.5	39.4	31.3	28.4	53.8	46.6	2,014
2	49.5	35.7	37.3	30.6	30.9	52.0	43.4	2,163
3	42.7	29.1	30.0	21.8	23.3	46.7	36.1	1,183
4+	29.8	20.0	20.8	17.9	19.7	36.0	25.7	1,395
**Residence**								
Rural	42.3	29.4	31.2	24.9	24.5	46.0	37.3	5,103
Urban	55.1	39.8	41.3	33.0	33.3	56.6	47.9	1,681
**Education**								
Non-literate[a]	29.7	19.0	19.4	13.6	15.4	31.8	22.7	2,293
Less than 5 years	36.4	24.1	18.6	14.2	15.2	38.1	32.9	295
5-9 years	46.2	31.5	33.6	26.1	25.2	50.2	40.7	2,153
10 or more year	60.0	45.0	48.4	41.2	39.6	63.3	55.2	2,043
**Religion**								
Hindu	47.0	33.2	35.0	27.8	27.6	50.1	41.6	5,638
Muslim	27.0	16.8	15.9	13.6	13.9	31.0	20.1	804
Sikh	52.8	39.5	42.7	35.8	33.3	55.8	45.6	337
Others	*	*	*	*	*	*	*	5
**Castes/tribes**								
Scheduled castes	40.9	25.4	28.9	22.3	22.7	43.9	35.6	1,696
Scheduled Tribes	54.4	30.2	40.6	30.7	27.5	39.3	33.7	40
Other backward classes	43.7	30.4	30.8	24.0	23.9	45.9	36.4	2,624
Others	50.5	38.2	39.8	33.0	32.2	54.9	46.6	2,424
**Wealth index**								
Lowest	18.6	7.9	9.2	5.4	10.6	16.7	14.2	129
Second	24.8	14.2	15.8	11.5	14.3	29.1	18.1	481
Middle	34.9	21.8	22.0	16.7	17.8	37.1	26.7	1,444
Fourth	41.9	28.2	30.2	24.1	23.0	44.8	35.3	2,090
Highest	56.8	42.7	44.9	36.5	35.7	60.5	53.2	2,639
**Haryana**	45.7	32.1	33.8	27.1	26.8	48.8	40.1	6,784

[#] Women who had their last live/still birth since 01-01-2004.
* Percentage not shown, based on less than 10 unweighted cases.
** Unweighted cases.
[a] Literates but did not attend school, are also included.

TABLE 4.5 (A) ANTENATAL CARE: ANC VISITS AND TIME OF FIRST ANC CHECK-UP

Percent distribution of women (aged 15-49) # by the number of antenatal check-up and the stage of pregnancy at the time of first check-up during pregnancy according to selected background characteristics, Haryana, 2007-08

	Number of ANC visits				Stage of pregnancy at the time of the first antenatal check-up			
Background characteristics	No visit	1	2	3+	First trimester	Second trimester	Third trimester	Number of women**
**Age group**								
15-19	16.7	8.1	24.6	50.7	48.5	28.9	5.7	395
20-24	9.2	6.7	28.5	55.6	59.1	29.7	2.5	2,768
25-29	11.9	6.6	29.2	52.3	57.1	28.5	2.3	2,251
30-34	18.5	6.0	26.3	49.1	49.8	29.6	2.2	966
35+	32.7	6.6	29.6	31.1	35.3	26.4	5.3	404
**No. of living children**								
0	9.9	3.3	26.8	60.0	69.6	20.6	0.0	29
1	6.2	4.8	22.7	66.3	65.3	26.3	2.5	2,014
2	8.6	6.6	27.6	57.2	60.5	29.2	2.1	2,163
3	14.1	7.3	32.2	46.4	51.9	31.4	2.8	1,183
4+	29.8	8.9	33.7	27.6	34.7	30.9	4.3	1,395
**Residence**								
Rural	15.4	7.5	30.0	47.1	51.9	29.8	3.1	5,103
Urban	7.0	4.1	22.9	66.0	64.4	26.8	1.9	1,681
**Education**								
Non literate[a]	28.4	9.7	32.6	29.3	37.0	29.8	4.5	2,293
Less than 5 years	12.6	9.7	38.5	39.3	49.3	35.7	2.8	295
5-9 years	7.1	6.2	31.1	55.7	58.6	32.1	2.7	2,153
10 or more years	2.6	3.1	18.7	75.6	73.0	24.0	1.0	2,043
**Religion**								
Hindu	8.8	6.1	28.8	56.3	59.4	29.7	2.4	5,638
Muslim	45.8	11.0	25.6	17.6	23.3	25.3	5.5	804
Sikh	4.4	3.9	25.3	66.4	65.4	28.4	1.8	337
Others	*	*	*	*	*	*	*	5
**Castes/tribes**								
Scheduled castes	12.8	8.1	33.7	45.4	51.7	32.7	3.1	1,696
Scheduled tribes	12.1	11.9	22.8	53.2	53.3	32.2	2.4	40
Other backward classes	20.4	7.8	26.8	45.0	48.8	27.8	3.1	2,624
Others	5.9	4.2	26.1	63.8	64.2	27.8	2.2	2,424
**Wealth index**								
Lowest	44.2	5.5	33.9	16.3	22.6	29.4	3.8	129
Second	32.0	12.8	29.3	25.9	34.0	29.4	4.9	481
Middle	23.0	9.5	34.7	32.8	40.4	31.9	4.3	1,444
Fourth	12.1	7.4	32.8	47.6	55.0	30.4	2.8	2,090
Highest	3.8	3.3	20.5	72.4	68.8	26.3	1.5	2,639
**Haryana (15-49)**	13.3	6.6	28.3	51.8	55.0	29.1	2.8	6,784
**Haryana (15-44)†**	13.2	6.6	28.3	51.9	55.1	29.1	2.8	6,768

Note: Percentage may not add to 100.0 due to multiple responses, do not know or missing cases.
# Women who had their last live/still birth since 01-01-2004.
* Percentage not shown, based on less than 10 unweighted cases.
** Unweighted cases.
[a] Literates but did not attend school, are also included.
† Represents figure for currently married women aged 15-44 years.

TABLE 4.5 (B) ANTENATAL CARE: TT, IFA AND ANC

Percent distribution of women (aged 15-49) [#] by the number of tetanus toxoid (TT) injections and iron folic acid (IFA) tablets/syrup received during pregnancy, and the percentage who received full antenatal check-up (ANC) according to selected background characteristics, Haryana, 2007-08

	Women who received TT			Women who received IFA tablets/syrup			
Background characteristics	No TT	1	2+	No IFA/ syrup	Consumed IFA for at least 3 months	Full ANC[b]	Number of women**
**Age group**							
15-19	16.9	7.6	75.5	18.5	11.3	9.1	395
20-24	9.8	6.5	83.4	17.3	16.3	12.2	2,768
25-29	12.8	6.6	80.5	18.3	19.5	15.5	2,251
30-34	18.9	5.4	75.4	18.2	18.6	14.1	966
35+	34.0	5.6	60.4	16.9	14.1	9.3	404
**No. of living children**							
0	9.9	6.6	83.5	23.3	22.1	13.6	29
1	6.8	3.9	89.1	17.3	22.9	18.8	2,014
2	8.8	7.5	83.6	17.1	20.1	15.5	2,163
3	15.3	7.3	77.1	19.0	13.8	9.8	1,183
4+	31.0	7.5	61.4	18.5	7.8	4.9	1,395
**Residence**							
Rural	16.0	6.9	76.9	17.3	14.0	10.2	5,103
Urban	8.0	4.6	87.0	19.3	27.2	22.6	1,681
**Education**							
Non literate[a]	30.3	7.9	61.7	20.8	6.2	4.2	2,293
Less than 5 years	12.6	10.0	77.4	22.0	7.5	4.6	295
5-9 years	7.7	6.9	85.1	19.0	15.0	10.8	2,153
10 or more years	2.0	3.5	94.3	12.5	33.9	27.6	2,043
**Religion**							
Hindu	9.3	6.1	84.5	16.8	19.3	14.9	5,638
Muslim	47.2	9.2	43.6	21.7	2.6	1.2	804
Sikh	5.4	4.4	89.9	24.9	20.6	17.0	337
Others	*	*	*	*	*	*	5
**Castes/tribes**							
Scheduled castes	13.9	7.5	78.5	16.3	13.9	10.3	1,696
Scheduled tribes	14.4	4.9	80.7	17.3	16.0	13.5	40
Other backward classes	21.1	7.3	71.4	18.2	14.0	10.3	2,624
Others	6.2	4.7	88.9	18.5	23.2	18.5	2,424
**Wealth index**							
Lowest	45.1	6.4	48.5	18.0	2.2	1.4	129
Second	33.7	9.9	56.2	18.8	5.8	3.4	481
Middle	24.6	7.8	67.6	18.8	7.9	5.0	1,444
Fourth	12.9	7.2	79.7	19.1	13.8	9.2	2,090
Highest	3.7	4.3	91.8	16.1	28.1	23.5	2,639
**Haryana (15-49)**	14.0	6.4	79.4	17.8	17.2	13.2	6,784
**Haryana (15-44)**[†]	13.9	6.5	79.6	17.8	17.3	13.3	6,768

Note: Percentage may not add to 100.0 due to multiple responses, do not know or missing cases.
[#] Women who had their last live/still birth since 01-01-2004.
* Percentage not shown, based on less than 10 unweighted cases.
** Unweighted cases.
[a] Literates but did not attend school, are also included.
[b] At least three visits for antenatal check-up, at least one TT injection received and 100+ IFA tablets/ syrup consumed.
† Represents figure for currently married women aged 15-44 years.

TABLE 4.6 ANTENATAL CARE INDICATORS AND COMPLICATIONS BY DISTRICT

Percentage of women (aged 15-49) # who received different types of antenatal care (ANC) by districts, Haryana, 2007-08

District	Antenatal check-up in the first trimester of pregnancy	Three or more antenatal check-up	At least one tetanus toxoid injection	100+ IFA tablets/ syrup[1]	Full[2] antenatal check-up	Any complications	Number of women**
Panchkula	68.2	72.3	90.0	27.9	19.1	59.7	296
Ambala	59.5	57.7	83.0	37.7	17.4	62.2	260
Yamunanagar	72.5	60.8	90.7	31.6	20.8	70.1	296
Kurukshetra	65.9	61.5	90.7	24.3	11.8	53.9	288
Kaithal	65.3	64.0	93.1	29.5	21.1	57.0	342
Karnal	61.9	58.9	88.2	28.1	16.6	51.8	333
Panipat	65.3	51.1	84.6	20.7	6.2	57.0	368
Sonipat	69.6	57.7	95.4	23.1	14.9	42.9	331
Jind	58.0	55.4	92.3	17.5	9.7	58.3	312
Fatehabad	51.2	41.7	88.9	22.8	9.5	52.0	338
Sirsa	58.1	56.0	92.1	29.2	17.8	45.3	277
Hisar	56.0	44.3	90.6	18.7	10.4	45.8	287
Bhiwani	48.9	48.3	90.8	22.2	8.9	49.5	331
Rohtak	66.7	60.1	96.1	35.7	27.5	57.9	306
Jhajjar	54.5	59.7	91.0	26.3	16.2	44.6	298
Mahendragarh	55.0	53.1	94.9	17.2	9.1	57.6	286
Rewari	68.4	70.0	95.9	30.3	20.6	53.1	300
Gurgaon	56.1	60.1	93.1	38.1	27.5	59.7	434
Faridabad	45.5	43.8	78.9	29.1	9.3	50.1	379
Mewat	17.4	17.0	52.7	48.8	1.9	71.0	722
**Haryana (15-49)**	55.0	51.8	85.9	29.0	13.2	56.5	6,784
**Haryana (15-44)**†	55.1	51.9	86.1	29.0	13.3	56.6	6,768

Women who had their last live/still birth since 01-01-2004.
** Unweighted cases.
[1] 100 or more iron folic acid tablets including syrup.
[2] At least three visits for antenatal check-up, at least one TT injection received and 100+ IFA tablets/ syrup consumed.
† Represents figure for currently married women aged 15-44 years.

TABLE 4.7 PLACE OF DELIVERY AND ASSISTANCE

Percent distribution of women (aged 15-49) [#] according to place of delivery, assistance during home deliveries, and safe deliveries according to background characteristics, Haryana, 2007-08

Background characteristics	Percentage of women who had institutional delivery	Percentage of women who had delivery at home	Home delivery assisted by skilled persons[1]	Percentage of safe delivery[2]	Number of women**
**Age group**					
15-19	47.1	52.5	6.7	53.8	395
20-24	49.5	50.0	7.7	57.2	2,768
25-29	47.1	52.4	5.6	52.7	2,251
30-34	45.6	54.0	5.3	50.9	966
35+	30.9	68.8	3.8	34.7	404
**No. of living children**					
0	67.6	32.4	3.4	71.0	29
1	64.8	34.8	6.5	71.3	2,014
2	51.9	47.9	6.3	58.2	2,163
3	36.9	62.3	7.8	44.7	1,183
4+	22.1	77.5	4.9	27.0	1,395
**Residence**					
Rural	42.1	57.5	5.7	47.8	5,103
Urban	61.5	38.0	8.1	69.6	1,681
**Education**					
Non literate[a]	24.0	75.3	5.6	29.6	2,293
Less than 5 years	39.1	60.6	6.4	45.5	295
5-9 years	46.4	53.2	7.0	53.4	2,153
10 or more years	75.0	24.7	6.6	81.6	2,043
**Religion**					
Hindu	50.1	49.5	7.1	57.2	5,638
Muslim	15.2	84.1	2.2	17.4	804
Sikh	76.4	23.6	4.8	81.2	337
Others	*	*	*	*	5
**Castes/tribes**					
Scheduled castes	36.8	63.0	7.3	44.1	1,696
Scheduled tribes	33.1	66.9	7.6	40.7	40
Other backward classes	40.4	58.9	5.6	46.0	2,624
Others	61.2	38.4	6.6	67.8	2,424
**Wealth index**					
Lowest	14.8	83.8	0.7	15.5	129
Second	18.4	81.1	4.1	22.5	481
Middle	26.3	73.2	5.9	32.2	1,444
Fourth	39.8	59.7	7.5	47.3	2,090
Highest	70.9	28.6	6.4	77.3	2,639
**Haryana (15-49)**	46.8	52.7	6.4	53.2	6,784
**Haryana (15-44)**[†]	46.9	52.6	6.5	53.4	6,768

Note: Percentage of women who had institutional and home delivery may not add to 100.0, as some deliveries took place on the way to the institute, working place, other place etc.
[#] Women who had their last live/still birth since 01-01-2004.
* Percentage not shown, based on less than 10 unweighted cases.
** Unweighted cases.
[1] Includes Doctor/ANM/Nurse.
[2] Either institutional delivery or home delivery assisted by skilled person.
[a] Literates but did not attend school, are also included.
† Represents figure for currently married women aged 15-44 years.

TABLE 4.8 MODE OF TRANSPORTATION USED FOR DELIVERY AND ARRANGEMENT OF TRANSPORTATION

Percent distribution of women (aged 15-49) [#] who had institutional delivery, according to the transportation used to reach the health facility for delivery and transportation arrangement made according to selected background characteristics, Haryana,2007-08

Background characteristics	Mode of transportation used to reach the health facility for delivery: Ambulance Jeep/ car	Motor cycle/ scooter	Others[1]	Mean Transport cost (Rupees)	Mean Delivery cost (Rupees): Govt.	Private	Govt. financial assistance for delivery care (JSY)[c]	Number of women**
**Age group**								
15-19	56.6	9.8	33.6	363	2,159	6,672	5.4	188
20-24	52.9	13.8	33.3	315	3,263	7,428	5.8	1,386
25-29	49.7	16.0	34.4	278	4,167	8,000	3.5	1,080
30-34	47.9	15.9	36.2	281	3,300	7,268	2.6	449
35+	37.4	14.1	48.5	240	2,791	6,625	2.1	130
**No. of living children**								
0	(40.0)	(20.0)	(40.0)	(417)	(3,216)	(5,380)	(0.0)	20
1	55.4	14.0	30.6	340	4,007	8,708	5.3	1,320
2	50.5	16.3	33.2	264	2,877	6,821	6.4	1,132
3	43.6	13.4	43.0	271	3,255	6,982	3.3	442
4+	42.8	12.5	44.7	281	3,633	5,857	1.9	319
**Residence**								
Rural	56.5	13.7	29.8	338	3,287	7,183	4.5	2,195
Urban	38.7	16.5	44.8	189	3,973	8,241	5.0	1,038
**Education**								
Non literate[a]	43.5	9.6	46.9	262	2,279	5,393	3.2	566
Less than 5	42.6	9.5	47.9	234	1,584	5,101	6.6	114
5-9 years	47.0	13.6	39.4	310	3,500	7,353	6.2	1,009
10 or more years	56.4	17.5	26.1	313	4,381	8,400	4.5	1,544
**Religion**								
Hindu	49.8	14.5	35.7	302	3,397	7,555	5.2	2,844
Muslim	53.1	11.6	35.3	307	2,763	5,830	1.8	125
Sikh	59.0	17.9	23.1	256	5,445	7,820	3.7	262
Other	*	*	*	*	*	*	*	2
**Castes/tribes**								
Scheduled Castes	40.0	9.0	51.1	242	2,365	6,125	8.6	633
Scheduled Tribes	(15.4)	(46.2)	(38.5)	(68)	(4,000)	(5,502)	(4.0)	13
Other backward classes	48.3	16.3	35.4	306	3,167	7,318	3.3	1,090
Others	57.3	15.5	27.1	323	4,542	8,122	3.2	1,497
**Wealth index**								
Lowest	(45.0)	(10.0)	(45.0)	(240)	(3,757)	(3,831)	(2.2)	20
Second	45.1	5.4	49.5	346	3,569	5,897	3.1	92
Middle	45.7	8.9	45.4	289	1,728	6,025	5.0	387
Fourth	48.5	11.2	40.3	330	2,886	6,494	5.4	849
Highest	53.0	17.8	29.2	281	4,528	8,284	4.3	1,885
**Haryana (15-49)**	50.7	14.6	34.7	299	3,483	7,523	4.6	3,233
**Haryana (15-44)**[†]	50.9	14.6	34.5	299	3,483	7,523	4.7	3,199

( ) Based on 10-24 unweighted cases.
[#] Women who had their last live/still birth since 01-01-2004.
* Percentage not shown, based on less than 10 unweighted cases.
** Unweighted cases.
[1] Includes bus/train, tempo/auto/tractor, animal drawn cart, foot march.
[a] Literates but did not attend school, are also included.
[b] Total figure may not add to 100 percent due to don't know or missing cases.
[c] Percentage women who got JSY assistance.
† Represents figure for currently married women aged 15-44 years.

TABLE 4.9 PLACE OF DELIVERY AND ASSISTANCE BY DISTRICT

Percent distribution of women (aged 15-49) [#] according to place of delivery, assistance during home deliveries, and safe deliveries by districts, Haryana, 2007-08

District	Percentage of women who had institutional delivery	Percentage of women who had delivery at home	Home delivery assisted by skilled persons[1]	Percentage of safe delivery[2]	Number of women**
Panchkula	64.3	35.4	3.5	67.8	296
Ambala	55.4	43.7	7.5	62.9	260
Yamunanagar	52.3	47.5	6.1	58.4	296
Kurukshetra	64.2	35.5	3.6	67.8	288
Kaithal	48.0	52.0	9.4	57.4	342
Karnal	51.3	47.8	5.8	57.1	333
Panipat	39.0	60.4	9.4	48.4	368
Sonipat	53.7	45.4	7.3	61.0	331
Jind	42.1	56.9	6.2	48.3	312
Fatehabad	48.6	51.1	9.5	58.1	338
Sirsa	53.5	46.4	16.0	69.5	277
Hisar	48.6	50.7	5.9	54.5	287
Bhiwani	35.7	64.6	9.1	44.8	331
Rohtak	52.8	46.4	6.1	58.9	306
Jhajjar	48.0	51.6	9.9	57.9	298
Mahendragarh	56.8	43.1	8.3	65.1	286
Rewari	65.0	35.1	8.5	73.5	300
Gurgaon	52.3	47.5	4.5	56.8	434
Faridabad	39.1	60.6	3.6	42.7	379
Mewat	14.8	84.5	1.5	16.3	722
**Haryana (15-49)**	46.8	52.7	6.4	53.2	6,784
**Haryana (15-44)**[†]	46.9	52.6	6.5	53.4	6,768

Note: Percentage of women who had institutional and home delivery may not add to 100.0, as some deliveries took place on the way to the institute, working place, other place etc.
[#] Women who had their last live/still birth since 01-01-2004.
** Unweighted cases.
[1] Includes Doctor/ANM/Nurse.
[2] Either institutional delivery or home delivery assisted by skilled person.
† Represents figure for currently married women aged 15-44 years.

TABLE 4.10 REASONS FOR NOT GOING TO HEALTH INSTITUTIONS FOR DELIVERY

Percent distribution of women (aged 15-49) # according to main reasons for not going to health institution for delivery, according to selected background characteristics, Haryana, 2007-08

Background characteristics	Reason										Number of women**
	Cost too much	Poor quality service	Too far/ No transport	No time to go	Not necessary	Not Custo mary	Better care at home	Family did not allow	Lack of know ledge	Other	
**Age group**											
15-19	18.7	1.4	0.7	12.6	52.9	3.9	24.1	8.8	0.9	1.4	207
20-24	20.3	3.2	1.4	14.1	44.7	4.6	27.8	5.0	1.3	2.2	1,390
25-29	22.1	3.8	2.3	15.4	46.1	4.4	23.8	4.5	1.5	1.2	1,189
30-34	26.9	3.6	1.8	14.6	45.7	4.0	25.0	5.3	1.9	2.2	528
35+	32.3	4.5	2.6	9.6	44.2	5.8	20.4	5.6	1.7	2.7	279
**No. of living children**											
0	*	*	*	*	*	*	*	*	*	*	9
1	16.5	2.4	1.1	15.3	46.1	4.3	28.8	7.2	1.5	2.1	705
2	20.1	3.2	1.2	17.1	45.0	4.2	26.1	4.9	1.0	1.9	1,047
3	23.1	3.1	2.8	14.1	46.1	3.9	23.6	4.9	1.0	2.6	747
4+	29.0	4.6	2.1	10.8	46.0	5.2	23.5	4.3	2.0	1.3	1,085
**Residence**											
Rural	23.4	3.6	1.9	13.0	46.4	4.3	25.4	5.1	1.4	1.6	2,938
Urban	19.5	3.0	1.2	19.7	42.5	5.4	24.6	5.3	1.4	3.2	655
**Education**											
Non literate[a]	28.1	4.2	2.2	10.6	46.5	5.2	23.4	4.9	2.0	1.5	1,731
Less than 5 years	27.3	4.6	0.4	12.4	43.8	0.5	23.9	3.9	0.5	3.3	185
5-9 years	18.8	3.1	1.7	17.1	46.8	3.5	25.9	5.2	1.2	1.9	1,169
10 or more years	11.0	1.4	1.3	20.6	41.0	5.9	31.0	6.5	0.4	2.8	508
**Religion**											
Hindu	21.0	3.5	1.5	15.5	44.8	4.2	25.4	4.9	1.1	2.4	2,830
Muslim	28.7	3.5	3.1	8.4	47.7	6.0	25.9	6.2	3.0	0.3	678
Sikh	26.3	1.2	0.0	20.5	60.4	1.2	15.8	3.6	0.0	0.0	83
Others	*	*	*	*	*	*	*	*	*	*	2
**Castes/tribes**											
Scheduled castes	23.2	3.4	2.2	15.4	45.1	4.1	21.5	5.2	1.2	2.6	1,085
Scheduled tribes	11.8	0.0	0.0	18.8	37.0	7.6	18.9	14.2	0.0	3.6	27
Other backward classes	25.6	3.8	2.2	11.7	46.6	4.5	26.2	5.2	1.8	1.3	1,536
Others	17.7	3.1	0.8	16.8	45.3	4.9	28.2	4.8	1.2	2.0	945
**Wealth index**											
Lowest	37.6	6.8	3.0	5.0	45.9	6.2	21.9	4.5	2.7	1.0	108
Second	28.3	4.9	3.1	9.5	44.2	5.1	25.0	6.4	1.5	1.9	394
Middle	28.8	3.8	2.0	12.4	46.2	3.5	21.9	3.9	2.2	1.5	1,061
Fourth	20.3	3.3	1.7	14.2	45.9	4.9	24.6	6.3	1.2	2.1	1,263
Highest	12.8	1.9	1.0	20.4	45.6	4.8	31.9	4.4	0.6	2.2	766
**Haryana**	22.7	3.5	1.8	14.2	45.7	4.5	25.3	5.2	1.4	1.9	3,593

# Women who had their last live/still birth since 01-01-2004.
* Percentage not shown, based on less than 10 unweighted cases.
** Unweighted cases.
[a] Literates but did not attend school, are also included.
[b] Total figure may not add to 100 percent due to ' Multiple responses', 'don't know' or 'missing cases'.

TABLE 4.11 DELIVERY COMPLICATIONS

Percentage of women (aged 15-49) # who had complications during delivery and type of complications, according to selected background characteristics, Haryana, 2007-08

Background characteristics	Any delivery complication	Type of delivery complications							Number of women**
		Premature labour	Excessive bleeding	Prolonged labour	obstructed labour	Breech presentation	Convulsion /high BP	Other	
**Age group**									
15-19	62.7	27.8	5.2	27.9	80.1	7.7	2.7	2.3	395
20-24	57.0	29.0	4.4	25.4	80.0	6.6	2.5	1.5	2,768
25-29	53.1	31.4	4.9	24.9	81.3	5.6	3.6	1.2	2,251
30-34	54.4	30.2	5.3	22.9	80.8	7.7	5.0	0.9	966
35+	59.3	25.5	4.8	16.6	85.7	5.1	2.8	0.9	404
**No. of living children**									
0	62.9	63.2	15.4	29.1	54.8	10.0	0.0	10.5	29
1	58.7	29.2	5.0	30.7	76.8	7.7	3.6	1.8	2,014
2	52.6	28.9	4.4	22.5	81.0	7.0	3.3	1.5	2,163
3	51.0	30.2	5.5	22.7	81.9	5.6	3.1	0.8	1,183
4+	60.5	30.0	4.1	19.9	86.1	4.5	2.9	0.7	1,395
**Residence**									
Rural	58.0	30.2	4.8	23.6	81.8	6.2	3.1	1.3	5,103
Urban	49.2	27.7	4.6	27.8	77.5	7.4	3.7	1.6	1,681
**Number of ANC Visits**									
No visit	62.5	33.8	3.5	21.1	85.2	2.7	2.1	0.3	863
1	55.9	37.3	5.5	28.6	78.6	5.5	2.0	1.6	448
2	56.6	25.9	3.8	23.3	83.4	4.7	1.6	0.8	1,928
3+	53.6	29.5	5.6	25.6	78.4	8.7	4.7	2.0	3,545
**Delivery**									
Normal	54.4	29.4	4.1	21.6	84.0	4.1	2.4	0.9	5,933
Caesarean	65.1	31.3	8.9	39.5	60.2	21.0	7.6	4.3	666
By Instrument or Assisted	67.9	28.9	8.4	47.3	73.0	15.9	8.5	3.0	185
**Place of delivery**									
Government facility	57.3	30.7	5.1	32.6	79.2	7.5	3.7	1.8	1,053
Private facility	58.1	30.5	6.4	29.7	73.8	10.5	5.0	2.3	2,149
Home	54.1	28.8	3.6	18.7	85.9	3.5	2.0	0.6	3,551
Other	48.7	19.4	5.4	13.1	81.4	6.5	0.0	0.0	31
**Haryana**	55.8	29.6	4.8	24.5	80.9	6.4	3.2	1.4	6,784

Note: Total figure may not add to 100 percent due to ‘multiple responses’, ‘don’t know’ or ‘missing cases’.
Women who had their last live/still birth since 01-01-2004.
** Unweighted cases.

TABLE 4.12 POST-DELIVERY COMPLICATIONS

Percentage of women (aged 15-49) # who had post delivery complications and type of complications, according to selected background characteristics, Haryana, 2007-08

Background characteristics	Any post delivery complication	Type of post-delivery complication: High fever	Lower abdominal pain	Foul smelling vaginal discharge	Excessive bleeding	Other	Number of women**
**Age group**							
15-19	32.9	59.3	47.5	17.2	15.3	40.8	395
20-24	28.7	54.9	42.7	11.7	15.2	45.3	2,768
25-29	27.1	55.4	46.1	13.5	17.4	43.6	2,251
30-34	27.7	56.6	46.6	11.1	14.3	43.2	966
35+	29.8	63.1	41.4	10.5	9.0	57.5	404
**No. of living children**							
0	46.0	62.9	36.1	14.3	14.2	30.2	29
1	28.2	54.3	42.9	13.2	17.0	41.6	2,014
2	25.7	50.5	45.9	13.3	17.1	41.2	2,163
3	27.9	55.9	45.8	10.6	14.1	47.1	1,183
4+	32.5	65.0	44.4	11.9	12.2	52.5	1,395
**Residence**							
Rural	29.4	58.1	44.2	11.8	13.9	47.0	5,103
Urban	25.2	49.2	45.9	14.9	20.6	37.9	1,681
**Delivery**							
Normal	26.7	57.8	43.4	12.9	14.8	45.8	5,933
Caesarean	39.4	47.5	53.1	10.1	15.4	41.3	666
By Instrument or Assisted	40.3	51.6	38.7	12.5	27.3	40.0	185
**Place of delivery**							
Government facility	27.7	45.8	46.7	11.3	16.5	40.3	1,053
Private facility	27.6	50.8	44.7	13.3	17.8	42.7	2,149
Home	29.0	61.9	43.9	12.3	13.8	47.5	3,551
Others	19.5	70.3	46.0	16.1	0.0	54.0	31
**Who conducted the last delivery**							
Doctor	21.4	31.5	61.3	12.0	11.1	42.9	65
ANM/Nurse/Midwife/LHV	26.8	58.1	49.5	8.5	19.3	49.3	386
Other Health Personal	*	*	*	*	*	*	1
Dai	29.4	63.0	43.6	12.3	13.1	47.5	2,862
Relatives/Friends	26.9	60.6	37.7	19.9	14.9	48.4	247
Others	(53.3)	(87.5)	(25.0)	(0.0)	(0.0)	(25.0)	15
No One	*	*	*	*	*	*	6
**Haryana**	28.3	56.1	44.6	12.5	15.4	45.0	6,784

( ) Based on 10-24 unweighted cases.
# Women who had their last live/still birth since 01-01-2004.
* Percentage not shown, based on less than 10 unweighted cases.
** Unweighted cases.

TABLE 4.13 ANY CHECK-UP AFTER DELIVERY

Percentage of women (aged 15-49) [#] received any check-up after delivery, according to background characteristics, Haryana, 2007-08

Background characteristics	Check up within 2 weeks after delivery	Check up within 48 hours after delivery	Number of women**
**Age group**			
15-19	48.3	48.3	395
20-24	51.3	50.5	2,768
25-29	49.7	49.0	2,251
30-34	50.0	49.4	966
35+	35.5	35.6	404
**No. of living children**			
0	56.7	57.6	29
1	62.0	61.1	2,014
2	53.1	52.5	2,163
3	42.4	41.5	1,183
4+	32.1	31.9	1,395
**Residence**			
Rural	46.4	45.8	5,103
Urban	58.7	57.9	1,681
**Education**			
Non literate[a]	33.4	33.0	2,293
Less than 5 years	41.9	41.8	295
5-9 years	47.7	47.2	2,153
10 or more years	71.0	69.9	2,043
**Religion**			
Hindu	50.4	49.7	5,638
Muslim	35.2	34.9	804
Sikh	71.0	70.1	337
Others	*	*	5
**Castes/tribes**			
Scheduled castes	40.1	39.6	1,696
Scheduled tribes	38.1	38.4	40
Other backward classes	46.6	46.0	2,624
Others	59.4	58.6	2,424
**Wealth index**			
Lowest	27.3	25.7	129
Second	31.6	31.6	481
Middle	34.9	34.7	1,444
Fourth	43.5	42.7	2,090
Highest	66.8	66.0	2,639
**Haryana (15-49)**	49.4	48.8	6,784
**Haryana (15-44)**[†]	49.5	48.9	6,768

[#] Women who had their last live/still birth since 01-01-2004.
* Percentage not shown, based on less than 10 unweighted cases.
** Unweighted cases.
[a] Literates but did not attend school, are also included.
† Represents figure for currently married women aged 15-44 years.

TABLE 4.14 COMPLICATIONS DURING PREGNANCY, DELIVERY AND POST-DELIVERY PERIOD

Percentage of women (aged 15-49) # who had pregnancy, delivery and post-delivery complications and sought treatment for the problem, according to background characteristics, Haryana, 2007-08

Background characteristics	Who had complication during pregnancy	Sought treatment for pregnancy complication[1]	Who had delivery complication	Who had post delivery complication	Sought treatment for post- delivery complication[2]	Number of women**
**Age group**						
15-19	59.5	51.8	62.7	32.9	65.5	395
20-24	56.2	62.3	57.0	28.7	70.8	2,768
25-29	55.4	62.6	53.1	27.1	72.9	2,251
30-34	57.1	57.0	54.4	27.7	74.2	966
35+	60.5	49.7	59.3	29.8	65.6	404
**No. of living children**						
0	59.8	72.0	62.9	46.0	78.5	29
1	57.2	65.8	58.7	28.2	72.3	2,014
2	54.1	64.1	52.6	25.7	73.3	2,163
3	53.4	55.8	51.0	27.9	69.5	1,183
4+	61.5	50.4	60.5	32.5	68.5	1,395
**Residence**						
Rural	56.0	58.1	58.0	29.4	70.7	5,103
Urban	58.1	66.0	49.2	25.2	73.3	1,681
**Education**						
Non literate[a]	56.4	47.6	59.8	30.3	67.2	2,293
Less than five years	60.3	57.1	55.4	34.6	65.0	295
5-9 years	56.1	63.9	55.5	29.9	70.2	2,153
10 or more years	56.6	71.2	51.6	23.5	80.1	2,043
**Religion**						
Hindu	54.5	62.8	52.7	26.8	71.4	5,638
Muslim	70.7	42.4	77.9	37.2	68.0	804
Sikh	52.6	77.0	49.0	30.4	79.4	337
Others	*	*	*	*	*	5
**Castes/tribes**						
Scheduled castes	56.7	59.3	53.3	30.4	70.5	1,696
Scheduled tribes	66.6	55.5	41.5	34.7	85.8	40
Other backward classes	59.8	55.6	61.0	29.2	69.7	2,624
Others	52.5	66.6	52.1	25.8	73.6	2,424
**Wealth index**						
Lowest	68.3	35.3	70.5	33.9	58.0	129
Second	59.3	42.3	62.7	33.3	60.5	481
Middle	55.5	48.4	59.7	32.6	66.5	1,444
Fourth	56.6	59.6	55.7	28.3	73.4	2,090
Highest	55.8	72.3	51.7	24.8	76.5	2,639
**Haryana**	56.5	60.1	55.8	28.3	71.3	6,784

[#] Women who had their last live/still birth since 01-01-2004.
* Percentage not shown, based on less than 10 unweighted cases.
** Unweighted cases.
[1] Women who reported at least one complication during pregnancy.
[2] Women who reported at least one post delivery complication.
[a] Literates but did not attend school, are also included.

TABLE 4.15 COMPLICATIONS DURING PREGNANCY, DELIVERY AND POST DELIVERY PERIOD BY DISTRICTS

Percentage of women (aged 15-49) [#] who had pregnancy, delivery, post-delivery complications and treatment seeking behaviour by districts, Haryana, 2007-08

District	Percentage of women[1]					Number of women**
	Who had complication during pregnancy	Sought treatment for pregnancy complication[2]	Who had delivery complication	Who had post-delivery complication	Sought treatment for post- delivery complication[3]	
Panchkula	59.7	67.0	49.0	29.1	82.3	296
Ambala	62.2	66.3	44.3	39.1	74.8	260
Yamunanagar	70.1	72.5	56.2	39.8	88.7	296
Kurukshetra	53.9	74.7	51.0	39.3	76.4	288
Kaithal	57.0	64.7	50.4	31.1	81.6	342
Karnal	51.8	74.6	48.0	37.5	75.8	333
Panipat	57.0	61.0	38.0	29.9	78.2	368
Sonipat	42.9	63.1	38.2	22.6	81.1	331
Jind	58.3	51.1	80.0	21.3	76.6	312
Fatehabad	52.0	65.2	59.1	29.9	58.7	338
Sirsa	45.3	67.2	61.5	26.0	68.6	277
Hisar	45.8	61.9	44.9	19.4	63.3	287
Bhiwani	49.5	53.3	78.1	18.7	65.4	331
Rohtak	57.9	55.2	46.0	27.8	61.1	306
Jhajjar	44.6	63.5	44.5	25.7	76.7	298
Mahendragarh	57.6	62.0	84.1	17.4	66.2	286
Rewari	53.1	69.9	37.9	18.7	67.9	300
Gurgaon	59.7	56.2	47.2	23.1	58.3	434
Faridabad	50.1	61.1	41.7	31.7	72.5	379
Mewat	71.0	40.5	83.8	35.1	64.3	722
**Haryana**	56.5	60.1	55.8	28.3	71.3	6,784

[#] Women who had their last live/still birth since 01-01-2004.
** Unweighted cases.
[1] Women who had last live/still birth during three years preceding the survey.
[2] Women who reported at least one complication of pregnancy.
[3] Women who reported at least one post delivery complication.

TABLE 4.16 KNOWLEDGE OF DANGER SIGNS OF NEW BORN

Percentage of women (aged 15-49) [#] who had knowledge of the danger signs of new born, according to selected background characteristics, Haryana, 2007-08

Background characteristic	Blue tongue & lips	Difficulty in breathing	Cold/ hot to touch	Develop yellow staining on palm and soles	Abnormal movement	Poor sucking of breast	Baby did not cry	Number of women**
**Age group**								
15-19	39.3	43.0	37.3	24.6	26.9	51.3	47.3	395
20-24	41.4	47.8	38.5	26.0	29.0	53.3	53.2	2,768
25-29	43.2	49.2	38.3	28.6	30.3	57.1	54.3	2,251
30-34	40.5	47.1	37.5	27.0	29.9	54.2	51.0	966
35+	42.9	51.1	32.6	24.4	26.2	50.7	52.8	404
**No. of living children**								
0	22.7	23.3	13.2	10.1	18.2	46.4	48.5	29
1	43.3	48.7	40.3	28.4	30.6	55.6	55.6	2,014
2	43.8	51.1	39.5	29.6	32.6	56.4	54.3	2,163
3	42.3	48.0	37.7	26.2	28.5	54.4	51.0	1,183
4+	36.8	43.3	32.7	21.4	23.1	49.8	48.6	1,395
**Residence**								
Rural	39.9	47.0	37.1	25.0	27.1	54.4	52.2	5,103
Urban	47.7	51.4	40.2	32.3	35.7	54.4	54.8	1,681
**Education**								
Non literatea	35.4	42.1	32.0	20.3	21.5	48.1	47.4	2,293
Less than 5 years	39.6	45.7	37.2	19.5	21.7	56.4	51.5	295
9-10 years	41.4	46.7	36.8	25.6	29.7	54.5	52.0	2,153
10 or more years	50.0	56.8	46.0	36.8	38.9	61.3	60.4	2,043
**Religion**								
Hindu	41.9	47.9	37.3	27.1	29.8	54.2	53.0	5,638
Muslim	40.3	48.1	40.5	22.3	22.9	54.6	51.7	804
Sikh	44.8	52.0	40.0	34.3	37.4	57.8	54.5	337
Others	*	*	*	*	*	*	*	5
**Castes/tribes**								
Scheduled castes	37.4	42.4	33.6	23.2	25.5	49.1	46.9	1,696
Scheduled tribes	46.1	41.9	29.9	25.1	25.1	48.3	33.5	40
Other backward classes	40.8	48.0	37.5	26.1	27.9	55.2	54.0	2,624
Others	46.0	52.3	41.4	30.2	33.4	57.3	56.2	2,424
**Wealth index**								
Lowest	31.4	36.8	30.3	13.2	13.8	40.2	44.1	129
Second	28.9	36.4	31.4	17.3	18.3	44.6	41.5	481
Middle	38.1	44.2	33.1	20.8	21.7	49.0	48.9	1,444
Fourth	39.2	46.1	35.7	24.5	27.7	52.8	51.9	2,090
Highest	48.9	54.6	43.9	34.6	37.5	61.3	58.5	2,639
**Haryana**	41.8	48.1	37.9	26.8	29.2	54.4	52.9	6,784

[#] Women who had their last live/still birth since 01-01-2004.
* Percentage not shown, based on less than 10 unweighted cases.
** Unweighted cases.
[a] Literates but did not attend school, are also included.

CHILD HEALTH CARE AND IMMUNIZATION

TABLE 5.1 TIMING AND PLACE OF EARLY CHILDHOOD CHECK-UP BY BACKGROUND CHARACTERISTICS

Percentage of children aged under 3 years received check-up and place of check-up according to selected background characteristics, Haryana, 2007-08

Background characteristics	Children received check-up within 24 hours of birth	Number of children**	Percentage among those who received check-up[b]				Number of children**
			Government[1]	Private[2]	Home[3]	Others	
**Age group**							
15-19	49.0	412	25.1	51.7	20.8	2.5	196
20-24	50.7	2,997	24.7	54.0	17.9	3.4	1,460
25-29	49.9	1,903	20.9	58.4	18.4	2.3	914
30-34	50.1	705	22.2	52.9	21.4	3.5	345
35-39	36.6	198	12.5	47.5	34.4	5.6	70
40-44	25.9	55	(7.1)	(50.0)	(42.9)	(0.0)	14
45-49	*	8	*	*	*	*	1
**Residence**							
Rural	47.0	4,780	22.4	50.9	23.2	3.5	2,169
Urban	57.6	1,498	24.1	65.2	8.8	1.9	831
**Mother's education**							
Non-literate[a]	32.3	2,108	18.9	34.1	42.6	4.4	661
Less than 5 years	46.4	290	24.1	44.2	23.0	8.7	129
5-9 years	49.4	2,051	28.9	49.6	17.0	4.5	969
10 or more years	70.1	1,829	20.2	71.7	7.6	0.5	1,241
**Religion**							
Hindu	51.0	5,162	24.7	57.7	14.4	3.2	2,535
Muslim	33.7	823	10.8	18.5	67.1	3.6	267
Sikh	67.6	287	17.7	74.8	7.0	0.5	192
Others	*	6	*	*	*	*	6
**Castes/Tribes**							
Scheduled Castes	41.1	1,626	29.0	41.8	23.1	6.1	646
Scheduled Tribes	36.8	39	(23.1)	(69.2)	(7.7)	(0.0)	14
Other Backward Classes	46.2	2,502	21.8	48.3	26.7	3.2	1,110
Others	60.2	2,110	20.7	67.3	10.6	1.4	1,229
**Wealth index**							
Lowest	31.7	128	4.3	21.9	73.8	0.0	39
Second	32.9	442	16.1	29.1	47.0	7.9	142
Middle	35.1	1,364	21.3	35.2	37.5	6.0	462
Fourth	43.6	1,996	26.6	48.2	21.7	3.5	837
Highest	67.1	2,348	22.5	67.8	8.1	1.5	1,520
**Haryana**	49.5	6,278	22.9	54.8	19.2	3.0	3,000

Note: Table based on youngest living child born since 01.01.2004.
( ) Based on 10-24 unweighted cases.
* Percentage not shown, based on less than 10 unweighted cases.
** Unweighted cases.
[1] Includes government hospital or dispensary, urban health centre/urban health post/ urban family welfare centre, community health centre or rural hospital, primary health centre, sub-centre, ICDS and Govt. AYUSH hospital/clinic.
[2] Includes non-governmental hospital/ trust hospital or clinic, private hospital/clinic and private AYUSH hospital /clinic.
[3] Includes Doctor and ANM/Nurse.
[4] Children who received check-up within 24 hours of birth.
[a] Literate but did not attend school, are also included.
[b] Total figure may not add to 100 percent due to 'do not know' or 'missing cases'.

TABLE 5.2 INITIATION OF BREASTFEEDING BY BACKGROUND CHARACTERISTICS

Percentage of children aged under 3 years whose mothers started breastfeeding within one hour of birth, within 24 hours of birth and after 24 hours of birth, according to selected background characteristics, Haryana, 2007-08

Background characteristics	Children received colostrum/*Khees*[b]	Percentage of women started breastfeeding			Number of children**
		Within one hour of birth	Within 24 hours of birth[1]	After 24 hours of birth	
**Age group**					
15-19	72.4	12.0	47.2	52.8	412
20-24	81.6	17.8	56.7	43.3	2,997
25-29	82.8	17.2	57.8	42.2	1,903
30-34	79.8	14.3	55.7	44.3	705
35-39	74.5	10.5	36.9	63.1	198
40-44	72.8	10.6	34.9	65.1	55
45-49	*	*	*	*	8
**Residence**					
Rural	79.9	16.0	53.8	46.2	4,780
Urban	83.5	18.2	60.4	39.6	1,498
**Mother's education**					
Non-literate[a]	73.6	11.1	40.8	59.2	2,108
Less than 5 years	81.3	14.9	51.4	48.6	290
5-9 years	82.3	18.1	61.2	38.8	2,051
10 or more years	87.6	21.4	67.0	33.0	1,829
**Religion**					
Hindu	82.2	18.2	60.2	39.8	5,162
Muslim	74.3	7.3	27.8	72.2	823
Sikh	76.2	15.6	57.5	42.5	287
Others	*	*	*	*	6
**Castes/Tribes**					
Scheduled Castes	78.9	18.2	55.8	44.2	1,626
Scheduled Tribes	67.4	15.1	50.8	49.2	39
Other Backward Classes	79.2	13.8	49.7	50.3	2,502
Others	84.5	18.5	62.0	38.0	2,110
**Wealth index**					
Lowest	72.3	10.2	33.4	66.6	128
Second	75.6	11.2	39.0	61.0	442
Middle	75.2	13.8	48.8	51.2	1,364
Fourth	81.1	16.4	53.7	46.3	1,996
Highest	85.3	19.6	65.2	34.8	2,348
**Haryana (15-49)**	80.8	16.5	55.4	44.6	6,278
**Haryana (15-44)**[†]	80.8	17.4	55.5	44.5	6,225

Note: Table based on youngest living child born since 01.01.2004.
* Percentage not shown, based on less than 10 unweighted cases.
** Unweighted cases.
[1] Includes children whose mother started breastfeeding within one hour of birth.
[a] Literate but did not attend school, are also included.
[b] Yellowish thick milk secretion during the first few days after child birth.
[†] Represents figures for children of currently married women aged 15-44 years.

TABLE 5.3 BREASTFEEDING AND WEANING STATUS BY CHILDREN'S AGE

Percentage of children aged under 3 years having exclusive breastfeeding and weaning status, Haryana, 2007-08

Age in months	Exclusive breastfeeding	Weaning status[1] Other fluids	Semisolid food	Solid food	Solid/semi-solid food	Number of children**
<2	19.1	48.5	0.7	1.5	2.2	267
2-3	6.9	83.7	2.9	0.4	3.3	273
4-5	3.4	67.4	18.4	2.8	21.2	322
6-8	0.6	25.0	46.6	21.9	68.5	616
9-11	0.2	4.0	33.2	51.6	84.8	621
12-17	0.4	2.0	7.1	72.7	79.8	916
18-23	0.5	6.2	3.3	59.7	63.0	1,189
24-35	0.5	9.9	5.7	46.8	52.5	2,074
6-9	0.5	19.7	45.4	28.7	73.4	849
6-35[2]	5.7	8.8	13.2	51.7	64.9	5,416
6-9 (15-44)[†]	0.5	19.5	45.5	28.1	74.1	844
6-35[2] (15-44)[†]	5.7	8.7	13.2	51.9	65.1	5,366
Median duration (months)	1.19	1.26	7.03	11.70	9.36	6,278

Note: Table based on youngest living child born since 01.01.2004. ** Unweighted cases.
[1] Based on those children who had breastfeeding with other fluids, semi solid food and solid food.
[2] Children aged 6-35 months breastfed for at least 6 months.
[†] Represents figures for children of currently married women aged 15-44 years.

TABLE 5.4 EXCLUSIVE BREASTFEEDING BY BACKGROUND CHARACTERISTICS

Percentage of children aged 0-5 months who received exclusive breastfeeding according to selected background characteristics, Haryana, 2007-08

Background characteristics	Exclusive breastfeeding 0-5 months	Number of children**
**Age group**		
15-19	8.1	105
20-24	8.4	451
25-29	10.7	209
30-34	15.1	70
35-39	(7.8)	23
40-44	*	4
45-49	nc	0
**Residence**		
Rural	9.3	669
Urban	9.7	193
**Mother's education**		
Non-literate[a]	6.0	300
Less than 5 years	13.5	39
5-9 years	10.4	268
10 or more years	11.9	255
**Religion**		
Hindu	11.1	671
Muslim	3.0	146
Sikh	6.8	44
Others	*	6
**Castes/Tribes[b]**		
Scheduled Castes	12.3	211
Scheduled Tribes	*	6
Other Backward Classes	7.7	373
Other	9.8	272
**Wealth index**		
Lowest	(0.0)	19
Second	7.0	81
Middle	9.3	178
Fourth	10.9	273
Highest	9.3	311
**Haryana (15-49)**	9.4	862
**Haryana (15-44)[†]**	9.4	859

Note: Table based on youngest living child born since 01.01.2004.
nc= Not calculated because there are no cases.( ) Based on 10-24 unweighted cases.
* Percentage not shown, based on less than 10 unweighted cases.** Unweighted cases.
[a] Literate but did not attend school, are also included. [b] Total figure may not add to N, sample total, due to 'don't know' or 'missing cases'.[†] Represents figures for children of currently married women aged 15-44 years.

TABLE 5.5 BREASTFEEDING BY DISTRICTS

Percentage of children aged under 3 years whose mothers started breastfeeding within one hour of birth, within 24 hours of birth, and after 24 hours of birth by districts, Haryana, 2007-08

Districts	Children received colostrum/*Khees*[a]	Percentage started breastfeeding			Number of children**
		Within one hour of birth	Within 24 hours of birth[1]	After 24 hours of birth	
Panchkula	86.0	19.7	70.5	29.5	276
Ambala	91.5	19.0	69.3	30.7	217
Yamunanagar	80.1	8.5	57.1	42.9	274
Kurukshetra	75.1	18.8	67.9	32.1	271
Kaithal	67.9	18.5	58.1	41.9	308
Karnal	86.6	7.8	54.6	45.4	319
Panipat	85.3	12.3	52.8	47.2	335
Sonipat	90.6	8.0	63.3	36.7	285
Jind	65.2	12.1	37.7	62.3	273
Fatehabad	68.9	10.8	40.3	59.7	303
Sirsa	71.6	27.5	57.2	42.8	264
Hisar	94.7	23.0	63.4	36.6	250
Bhiwani	63.4	24.1	47.6	52.4	306
Rohtak	86.8	20.3	70.9	29.1	295
Jhajjar	95.9	15.7	49.9	50.1	252
Mahendragarh	83.2	23.3	61.0	39.0	260
Rewari	85.0	33.3	74.8	25.2	291
Gurgaon	82.8	17.6	67.8	32.2	402
Faridabad	93.8	10.9	53.1	46.9	354
Mewat	73.2	7.5	29.2	70.8	743
**Haryana (15-49)**	80.8	16.5	55.4	44.6	6,278
**Haryana (15-44)**[†]	80.8	17.4	55.5	44.5	6,225

Note: Table based on youngest living child born since 01.01.2004.
** Unweighted cases.
[1] Includes children whose mother started breastfeeding within one hour of birth.
[a] Yellowish thick milk secretion during the first few days after child birth.
[†] Represents figures for children of currently married women aged 15-44 years.

TABLE 5.6 VACCINATION OF CHILDREN BY BACKGROUND CHARACTERISTICS

Percentage of children aged 12-23 months who received specific vaccination according to selected background characteristics, Haryana, 2007-08

Background characteristics	BCG	DPT 1	DPT 2	DPT 3	Polio 0	Polio 1	Polio 2	Polio 3	Measles	Full vaccination[1]	No vaccination	Vaccination card seen	Number of children**
**Residence**													
Rural	85.0	82.9	77.9	66.2	31.5	96.9	81.3	65.0	66.3	55.9	1.8	35.3	1,593
Urban	91.0	89.8	84.1	77.6	17.8	97.5	84.7	76.2	77.2	70.9	1.9	49.2	532
**Sex of the child**													
Male	87.8	85.9	81.1	71.7	27.5	97.2	83.1	70.2	70.9	62.5	1.7	40.0	1,178
Female	84.9	83.1	77.3	65.8	28.8	96.7	81.0	64.7	66.7	56.0	2.1	37.2	947
**Birth order**													
1	91.7	91.0	86.5	77.6	20.8	97.7	87.8	76.5	76.6	68.5	1.0	47.2	749
2	92.0	91.2	86.6	76.9	23.9	98.3	88.0	75.3	75.9	66.4	0.9	42.1	631
3	86.0	83.3	77.1	67.7	29.0	95.9	81.4	67.1	67.2	57.5	3.2	36.8	316
4+	68.9	62.8	55.7	40.2	48.1	94.4	62.0	38.5	43.8	31.7	3.8	18.2	337
**Mother's education**													
Non-literate[a]	71.7	65.1	57.2	43.5	42.4	94.1	64.9	42.7	45.8	34.9	4.6	20.9	685
Less than 5 years	78.1	81.8	74.0	59.4	35.9	94.3	77.4	56.8	55.2	45.0	2.8	30.2	105
5-9 years	92.3	92.5	87.4	74.9	26.1	98.8	88.1	73.7	77.3	66.0	0.3	41.9	651
10 or more years	97.6	97.7	95.6	91.3	13.8	98.7	95.1	89.9	87.3	81.4	0.4	55.7	684
**Religion**													
Hindu	91.8	91.7	87.1	76.5	23.6	97.9	87.8	75.1	75.7	65.9	1.2	42.8	1,767
Muslim	53.1	40.5	30.5	20.3	58.5	91.6	45.5	20.3	25.6	17.2	6.3	10.5	274
Sikh	97.8	96.6	96.6	90.5	9.8	97.5	97.5	89.0	89.1	84.1	0.0	59.2	82
Others	*	*	*	*	*	*	*	*	*	*	*	*	2
**Castes/Tribes**													
Scheduled Castes	88.4	88.8	82.4	69.4	29.3	97.5	84.2	68.0	68.0	56.6	1.7	38.0	537
Scheduled Tribes	(87.5)	(70.5)	(70.5)	(56.3)	(18.8)	(87.5)	(75.0)	(50.0)	(50.0)	(43.8)	(11.9)	(37.5)	16
Other Backward Classes	80.5	75.0	69.4	60.7	32.1	96.1	74.7	59.8	61.5	53.1	2.7	35.3	826
Others	92.0	92.7	88.8	78.6	22.7	97.9	89.2	77.1	78.9	69.5	0.8	43.4	746
**Wealth index**													
Lowest	51.8	47.2	47.2	31.4	65.5	90.4	50.8	31.4	32.0	28.5	9.6	9.6	31
Second	66.1	66.4	54.9	43.9	48.8	98.1	59.7	42.1	40.6	30.0	1.0	21.1	100
Middle	76.6	70.4	62.2	47.3	39.0	94.8	71.2	46.8	48.5	36.3	3.9	22.2	383
Fourth	85.8	83.1	78.2	66.7	31.6	96.3	80.5	65.6	66.6	57.3	2.4	34.2	620
Highest	94.1	94.4	90.6	83.0	18.1	98.4	90.9	81.4	82.9	74.4	0.6	51.0	991
**Haryana (15-49)**	86.5	84.6	79.4	69.0	28.1	97.0	82.2	67.8	69.0	59.6	1.9	38.8	2,125
**Haryana (15-44)**[†]	86.5	84.7	79.5	69.1	28.0	97.0	82.2	67.9	69.0	59.6	1.9	38.9	2,111

Note: Table based on youngest living child born since 01.01.2004.
( ) Based on 10-24 unweighted cases.
* Percentage not shown, based on less than 10 unweighted cases. .
** Unweighted cases.
[1] BCG, three injections of DPT, three doses of Polio (excluding Polio "0") and measles.
[a] Literate but did not attend school, are also included.
[†] Represents figures for children of currently married women aged 15-44 years.

TABLE 5.7 CHILDHOOD VACCINATION BY DISTRICTS

Percentage of children aged 12-23 months received specific vaccination and Vitamin-A supplementation by districts, Haryana, 2007-08

Districts	Percentage immunized								Percentage received at least one dose of Vitamin-A[2]	Number of children**
	Vaccination card seen	BCG	DPT3	Polio 0	Polio 3	Measles	Full[1]	None		
Panchkula	61.1	94.9	88.7	9.5	90.2	82.8	78.1	0.0	73.9	99
Ambala	61.1	95.2	82.0	1.7	86.2	91.9	79.1	4.8	65.9	73
Yamunanagar	55.8	97.7	82.1	1.4	79.6	85.7	70.0	2.3	61.5	101
Kurukshetra	54.8	93.8	77.9	6.6	74.3	82.7	67.8	3.8	67.2	107
Kaithal	54.9	91.9	83.5	18.1	83.5	73.7	72.5	1.3	56.3	113
Karnal	52.0	98.1	89.9	4.4	81.8	87.1	75.2	1.9	78.4	110
Panipat	22.7	83.8	70.7	4.9	63.4	71.8	57.0	11.4	54.9	114
Sonipat	45.7	95.6	81.6	11.3	78.8	83.9	73.0	2.9	70.2	94
Jind	37.4	92.5	66.4	42.2	66.4	67.3	55.4	0.8	57.1	91
Fatehabad	35.3	86.8	72.5	31.1	70.9	76.2	62.8	0.0	45.0	82
Sirsa	48.8	94.1	69.5	17.4	71.1	76.2	61.3	0.0	59.6	83
Hisar	47.5	93.7	69.6	21.1	69.6	65.7	55.8	2.6	44.6	88
Bhiwani	24.1	88.5	70.8	45.1	68.5	71.8	58.4	0.0	44.5	100
Rohtak	35.8	95.1	86.7	29.7	86.7	80.6	75.7	0.0	46.8	102
Jhajjar	43.1	90.6	81.2	33.1	77.7	69.6	64.8	0.0	42.8	87
Mahendragarh	42.2	92.1	79.4	31.3	79.5	71.9	67.7	0.9	52.4	94
Rewari	39.4	94.8	82.2	31.1	80.6	74.3	67.3	0.0	54.1	99
Gurgaon	37.9	90.7	74.7	29.6	74.7	76.5	70.5	0.0	49.5	134
Faridabad	36.5	79.2	54.8	37.0	54.8	57.0	46.4	0.0	28.7	114
Mewat	4.9	48.5	14.0	71.7	14.0	20.3	11.0	4.8	7.9	240
**Haryana (15-49)**	38.8	86.5	69.0	28.1	67.8	69.0	59.6	1.9	49.4	2,125
**Haryana (15-44)**[†]	38.9	86.5	69.1	28.0	67.9	69.0	59.6	1.9	46.3[#]	2,111

Note. Table based on youngest living child born since 01.01.2004.
** Unweighted cases.
[1] BCG, three injections of DPT, three doses of Polio (excluding Polio 0) and measles.
[2] Children aged 12-35 months.
[#] Children aged 9-35 months.
[†] Represents figures for children of currently married women aged 15-44 years.

TABLE 5.8 PLACE OF CHILDHOOD VACCINATION BY BACKGROUND CHARACTERISTICS

Percent of children aged 3 years received vaccination by place of vaccination, according to selected background characteristics, Haryana, 2007-08

	Place of vaccination					
	Government health sector					
Background characteristics	Sub-Centre	Primary Health centre	Other government health facility	Private health sector[1]	Others	Number of children**
**Residence**						
Rural	15.5	7.9	86.3	5.7	2.6	4,591
Urban	4.4	9.7	81.8	20.2	2.7	1,452
**Sex of the child**						
Male	13.5	8.6	84.6	9.7	2.8	3,326
Female	12.1	8.1	86.0	8.6	2.4	2,717
**Birth order**						
1	13.2	8.6	83.0	12.8	2.2	2,141
2	13.5	9.9	83.1	10.0	1.9	1,779
3	13.8	7.4	86.9	5.5	3.2	914
4+	10.3	5.7	91.7	4.4	3.8	991
**Mother's education**						
Non-literate[a]	11.9	6.6	89.2	2.8	4.5	1,944
Less than 5 years	19.0	7.1	84.1	2.7	1.1	271
5-9 years	14.9	8.9	85.7	5.3	2.2	1,999
10 or more years	10.9	9.8	80.4	21.5	1.2	1,829
**Religion**						
Hindu	13.6	9.0	84.5	9.7	2.3	5,017
Muslim	6.4	3.8	95.9	2.1	5.2	736
Sikh	19.6	10.2	65.3	21.7	0.7	285
Others	*	*	*	*	*	5
**Castes/Tribes**						
Scheduled Castes	15.6	8.8	84.4	3.5	2.7	1,550
Scheduled Tribes	2.9	2.9	94.1	3.1	3.0	32
Other Backward Classes	10.3	8.0	87.5	7.4	2.8	2,380
Others	14.1	8.5	82.9	15.6	2.3	2,080
**Wealth index**						
Lowest	12.5	5.1	89.9	1.4	6.7	77
Second	11.6	7.0	92.7	2.9	5.3	299
Middle	13.2	8.0	88.9	1.5	3.1	1,030
Fourth	15.2	8.1	86.3	3.2	3.1	1,793
Highest	11.5	8.9	82.1	16.7	1.7	2,844
**Haryana**	12.9	8.4	85.2	9.2	2.6	6,043

Note: Table based on youngest living child born since 01.01.2004.
* Percentage not shown, based on less than 10 unweighted cases.
** Unweighted cases.
[1] Includes non-governmental hospital/ trust hospital or clinic, private hospital and private doctor/clinic.
[a] Literate but did not attend school, are also included.

TABLE 5.9 VITAMIN-A AND HEPATITIS-B SUPPLEMENTATION FOR CHILDREN BY BACKGROUND CHARACTERISTICS

Percentage of children aged 12-35 months received at least one dose of Vitamin-A and Hepatitis-B injection, according to selected background characteristics, Haryana, 2007-08

Background characteristics	Children who received at least one dose of Vitamin-A	Children who received 3-5 doses of Vitamin-A	Children who received Hepatitis-B injection	Number of children**
**Age of the child**				
12-23 months	50.0	5.5	31.9	2,125
24-35 months	48.7	13.3	34.0	2,080
**Residence**				
Rural	47.0	9.2	28.1	3,159
Urban	56.6	9.8	47.6	1,046
**Sex of the child**				
Male	50.9	9.5	35.5	2,310
Female	47.5	9.1	29.8	1,895
**Birth order**				
1	58.8	9.0	40.6	1,401
2	53.5	10.4	38.3	1,240
3	45.2	7.7	28.8	634
4+	30.1	8.8	14.5	717
**Mother's education**				
Non-literate[a]	31.4	9.2	15.0	1,418
Less than 5 years	39.2	5.2	16.6	196
5-9 years	53.1	9.2	33.9	1,361
10 or more years	68.4	9.9	56.0	1,230
**Religion**				
Hindu	54.1	8.9	36.0	3,495
Muslim	17.2	16.5	8.4	533
Sikh	65.1	9.7	55.9	173
Others	*	*	*	4
**Castes/Tribes**				
Scheduled Castes	48.3	9.5	26.3	1,076
Scheduled Tribes	36.8	36.1	26.4	29
Other Backward Classes	44.1	9.6	29.6	1,667
Others	56.8	8.6	41.9	1,432
**Wealth index**				
Lowest	9.5	0.0	7.9	62
Second	25.6	6.2	12.3	211
Middle	33.6	9.2	15.3	751
Fourth	47.3	9.5	22.1	1,258
Highest	61.1	9.5	50.3	1,923
**Haryana (15-49)**	49.4	9.3	32.9	4,205
**Haryana (15-44)**[#†]	46.3	9.0	31.2	4,834

Note: Table based on youngest living child born since 01.01.2004.
** Percentage not shown, based on less than 10 unweighted cases.
** Unweighted cases.
[a] Literate but did not attend school, are also included.
[#] Children aged 9-35 months.
[†] Represents figures for children of currently married women aged 15-44 years.

TABLE 5.10 KNOWLEDGE REGARDING DIARRHOEA MANAGEMENT BY BACKGROUND CHARACTERISTICS

Percentage of women who are aware of diarrhoea management according to selected background characteristics, Haryana, 2007-08

Background characteristics	Knowledge of diarrhoea management	Type of practices followed if child gets diarrhoea[1]						Number of women**
		Give ORS	Salt and sugar solution	Continue normal food	Continue breast-feeding	Give plenty of fluids	Others	
**Age group**								
15-19	61.2	49.9	47.9	15.4	3.2	23.1	31.8	1,235
20-24	76.9	56.2	49.8	15.8	4.1	21.9	32.1	4,012
25-29	82.6	51.3	55.3	18.3	3.2	23.7	35.8	4,032
30-34	82.0	41.8	55.7	18.5	2.4	26.0	40.6	3,918
35-39	80.3	35.7	56.2	20.0	2.3	26.3	42.1	3,364
40-44	79.1	31.3	54.5	18.7	2.8	25.3	45.9	2,830
45-49	78.9	31.2	53.8	18.2	2.7	25.7	43.0	2,093
**Residence**								
Rural	76.7	37.7	50.6	18.3	3.2	24.3	41.8	16,154
Urban	86.0	58.5	62.9	17.4	2.5	25.4	30.9	5,330
**Mother's education**								
Non-literate[a]	70.4	19.1	42.5	20.4	2.9	24.8	48.4	8,928
Less than 5 years	75.4	32.4	52.1	19.1	2.7	27.3	41.2	925
5-9 years	80.7	45.6	56.2	16.9	2.5	24.5	38.6	6,384
10 or more years	92.5	74.2	66.7	16.1	3.7	24.0	26.4	5,248
**Religion**								
Hindu	80.1	44.7	55.0	17.3	2.9	24.4	38.8	18,724
Muslim	68.5	15.3	41.0	27.8	3.7	31.5	40.6	1,419
Sikh	76.2	51.8	52.2	18.9	2.8	19.9	38.1	1,315
Others	84.7	71.6	56.3	28.9	0.0	38.1	35.5	26
**Castes/Tribes**								
Scheduled Castes	72.4	36.3	49.6	18.0	2.9	23.0	42.1	4,924
Scheduled Tribes	85.0	45.6	34.7	14.4	1.3	12.0	52.2	87
Other Backward Classes	78.6	39.0	53.1	20.1	3.0	27.0	38.5	7,684
Others	83.1	50.2	56.9	16.5	3.0	23.5	37.5	8,787
**Wealth index**								
Lowest	68.0	11.0	36.8	22.2	3.6	24.4	50.4	258
Second	68.6	17.4	37.2	17.9	2.4	20.9	52.0	1,270
Middle	71.2	23.1	42.6	19.0	3.1	25.0	47.3	4,213
Fourth	76.3	36.3	49.9	19.4	2.8	25.0	41.8	6,730
Highest	86.5	59.5	63.3	16.8	3.1	24.6	31.9	9,012
**Haryana**	79.0	43.3	53.9	18.1	3.0	24.6	38.9	21,484

Note: Table based on women with youngest living children born since 01.01.2004.
** Unweighted cases.
[1] Among women aware of diarrhoea management.
[a] Literate but did not attend school, are also included.

TABLE 5.11 TREATMENT OF DIARRHOEA BY BACKGROUND CHARACTERISTICS

Percentage of children suffered from diarrhoea and sought advice/treatment according to selected background characteristics, Haryana, 2007-08

Background characteristics	Children suffered from diarrhoea[1]	Number of children**	Given ORS	Children sought advice/treatment	Source of treatment: Government health facility[2]	Source of treatment: Private health facility[3]	Source of treatment: Other	Number of children**
**Age group**								
Less than 25	17.2	4,290	33.8	82.2	8.3	88.4	4.8	602
25-29	15.6	3,035	31.2	80.1	8.2	88.1	5.8	376
30-34	16.4	1,199	29.8	85.1	10.3	80.0	10.3	166
35-39	16.6	365	23.7	75.6	20.1	77.7	4.6	46
40-49	18.5	122	11.9	85.6	(5.3)	(78.9)	(21.1)	19
**Residence**								
Rural	17.0	6,835	28.3	81.4	8.6	86.7	6.4	930
Urban	15.0	2,176	43.9	82.8	10.2	85.9	5.5	279
**Mother's education**								
Non-literate[a]	17.9	3,132	17.1	78.4	7.4	85.1	8.8	434
Less than 5 years	20.7	410	25.3	75.7	6.1	92.5	1.5	63
5-9 years	16.7	2,899	35.7	83.8	10.2	86.7	5.3	401
10 or more years	14.0	2,570	51.3	85.7	10.3	87.2	4.3	311
**Religion**								
Hindu	15.4	7,427	35.9	83.6	9.5	86.4	5.8	954
Muslim	23.8	1,165	12.7	73.4	8.4	84.4	9.2	204
Sikh	13.6	412	49.7	91.1	1.9	98.1	0.0	50
Others	*	7	*	*	*	*	*	2
**Castes/Tribes** [b]								
Scheduled Castes	16.3	2,293	30.1	84.7	11.1	85.2	6.0	317
Schedule Tribes	17.2	50	22.7	89.2	*	*	*	8
Other Backward Classes	18.5	3,576	24.8	78.0	7.1	86.6	7.6	510
Others	14.4	3,091	43.8	84.8	9.2	88.4	4.2	374
**Wealth index**								
Lowest	22.0	131	10.3	74.2	(9.5)	(81.0)	(9.5)	21
Second	22.0	482	13.0	71.6	5.5	90.5	6.8	75
Middle	17.5	1,588	20.4	78.0	11.8	81.3	8.5	214
Fourth	17.6	2,718	27.7	82.1	8.9	85.7	6.9	388
Highest	14.6	4,091	44.7	85.4	8.1	89.3	4.3	510
**Haryana (15-49)**	16.5	9,011	31.7	81.7	9.0	86.5	6.2	1,209
**Haryana (15-44)**[†]	16.6	8,905	31.7	82.0	8.9	86.5	6.2	1,200

Note: Table based on women with youngest living children born since 01.01.2004.
( ) Based on 10-24 unweighted cases.
* Percentage not shown, based on less than 10 unweighted cases.
** Unweighted cases.
[1] Last two weeks prior to survey.
[2] Includes government hospital or dispensary, urban health centre/ urban health post/urban family welfare centre, community health centre or rural hospital, primary health centre, sub-centre, ICDS and Govt. AYUSH hospital/clinic.
[3] Includes non-governmental hospital/trust hospital or clinic, private hospital/clinic and private AYUSH hospital/clinic.
[a] Literate but did not attend school, are also included.
[b] Total figure may not add to sample total, due to 'don't know' or 'missing cases'.
[†] Represents figures for children of currently married women aged 15-44 years.

TABLE 5.12 KNOWLEDGE AND TREATMENT OF ACUTE RESPIRATORY INFECTION (ARI) BY BACKGROUND CHARACTERISTICS

Percentage of women who are aware of danger signs of ARI and whose children suffer from ARI and sought advice/treatment according to selected background characteristics, Haryana, 2007-08

Background characteristics	Women aware of danger signs of ARI	Number of women**	Danger signs of Acute Respiratory Infection (ARI)[1]: Difficulty in breathing	Pain in chest and productive cough	Wheezing/ whistling	Rapid breathing	Other signs[2]	Children suffered from ARI[3]	Children sought advice/treatment[4]	Source of treatment[5]: Government health facility[6]	Private health facility[7]	Others	Number of children**
**Age group**													
15-19	54.0	1,235	55.8	58.3	46.6	38.7	37.0	7.7	85.5	9.4	83.0	7.5	435
20-24	69.2	4,012	61.7	59.6	47.8	36.2	43.5	8.8	89.5	8.8	85.3	5.9	3,855
25-29	78.6	4,032	61.6	62.1	50.0	38.4	43.1	7.7	89.9	3.9	90.3	5.7	3,035
30-34	80.3	3,918	63.9	62.4	48.7	37.6	41.0	8.3	88.0	6.2	85.2	8.6	1,199
35-39	80.7	3,364	62.1	62.9	50.0	39.6	40.1	9.6	77.9	20.8	73.6	5.7	365
40-44	81.9	2,830	62.9	62.6	51.8	40.0	37.8	9.3	66.7	7.1	85.7	7.1	102
45-49	83.0	2,093	63.7	62.0	49.9	38.1	40.0	(4.3)	(100.0)	(0.0)	(66.7)	(33.3)	20
**Residence**													
Rural	76.8	16,154	62.6	61.9	49.4	38.5	39.1	8.8	87.3	7.3	85.2	7.4	6,835
Urban	77.3	5,330	61.5	61.5	49.8	37.6	46.4	6.8	91.5	7.6	89.8	2.7	2,176
**Mother's education**													
Non-literate[a]	75.9	8,928	60.1	60.7	50.1	39.4	36.2	8.7	84.7	6.6	83.9	9.5	3,132
Less than 5 years	75.6	925	61.1	65.1	51.4	40.4	41.9	12.8	81.7	5.3	91.2	3.5	410
5-9 years	75.7	6,384	63.2	62.3	49.8	38.4	42.1	9.1	90.7	8.1	86.3	5.7	2,899
10 or more years	80.4	5,248	65.0	62.3	47.9	35.8	47.2	6.3	92.2	8.5	88.1	3.4	2,570
**Religion**													
Hindu	77.1	18,724	63.0	62.0	49.2	37.9	41.5	8.1	90.3	7.8	86.2	5.9	7,427
Muslim	81.1	1,419	54.5	58.8	56.1	47.9	31.4	9.6	78.4	5.0	84.4	10.6	1,165
Sikh	69.9	1,315	62.4	62.4	45.0	29.9	46.2	8.2	92.3	9.1	90.9	0.0	412
Other	60.7	26	66.7	60.0	73.3	40.0	33.3	*	*	*	*	*	7
**Castes/Tribes**													
Scheduled Castes	72.8	4,924	59.0	60.8	47.8	38.5	40.0	10.1	91.4	7.8	84.4	7.8	2,293
Scheduled Tribes	76.9	87	64.1	74.4	54.0	32.0	30.8	19.2	61.5	25.0	75.0	0.0	50
Other Backward Classes	77.3	7,684	61.8	61.9	50.7	38.8	39.0	8.3	85.3	5.7	87.8	6.4	3,576
Others	78.9	8,787	64.3	62.0	49.2	37.7	43.2	7.0	90.2	8.8	85.9	5.3	3,091
**Wealth index**													
Lowest	77.9	258	58.0	52.9	56.5	48.0	27.2	9.3	53.6	6.7	93.3	0.0	131
Second	73.6	1,270	57.7	55.9	49.5	42.3	34.8	7.3	78.7	2.9	84.3	12.9	482
Middle	75.5	4,213	59.6	60.9	49.7	40.3	37.0	9.8	88.2	10.3	79.4	10.3	1,588
Fourth	75.4	6,730	62.0	63.3	50.3	38.4	40.3	9.4	86.0	6.7	87.4	5.9	2,718
Highest	79.2	9,012	64.5	62.1	48.6	36.4	44.4	7.1	93.9	7.2	88.6	4.2	4,091
**Haryana (15-49)**	76.9	21,484	62.3	61.8	49.5	38.3	41.0	8.3	88.1	7.4	86.2	6.4	9,011
**Haryana (15-44)**[†]	76.3	18,575	62.2	61.7	49.4	38.3	41.2	8.3	88.1	7.2	86.3	6.5	8,905

Note: Table based on women with youngest living children born since 01.01.2004.

*Percentage not shown, based on less than 10 unweighted cases. ** Unweighted cases. ( ) Based on 10-24 unweighted cases.

[1] Among women who are aware of any danger signs of ARI. [2] Includes not able to drink or take a feed, excessive drowsy and difficulty to keep awake, running nose and others. [3] Last two weeks prior to survey. [4] Among children with ARI or fever in last two weeks who sought advice/treatment. [5] Among children who sought advice/treatment. [6] Includes government hospital or dispensary, urban health centre/urban health post/urban family welfare centre, community health centre or rural hospital, primary health centre, sub-centre, ICDS and Govt. AYUSH hospital/clinic. [7] Includes non-governmental hospital/trust hospital or clinic, private hospital/clinic and private AYUSH hospital/clinic. [a] Literate but did not attend school are also included. [†] Represents figures for children of currently married women aged 15-44 years.

TABLE 5.13 KNOWLEDGE OF ORS AND ACUTE RESPIRATORY INFECTION (ARI) BY DISTRICTS

Percentage of women by awareness of ORS and percentage of children suffered from diarrhoea and ARI and sought advice/treatment by districts, Haryana, 2007-08

Districts	Women aware of ORS	Children suffered from diarrhoea[1]	Children sought advice/treatment	Children suffered from ARI[1]	Children sought advice/treatment[2]	Number of children**
Panchkula	56.0	15.7	97.5	9.8	98.4	388
Ambala	50.8	15.1	74.0	13.8	91.2	311
Yamunanagar	42.5	26.8	88.1	13.1	94.8	374
Kurukshetra	43.2	22.8	79.5	14.9	82.7	363
Kaithal	52.8	12.1	98.3	7.6	96.0	454
Karnal	39.4	33.4	88.3	14.6	91.5	431
Panipat	37.8	22.1	85.6	9.2	87.0	483
Sonipat	44.0	13.5	83.7	7.3	94.2	429
Jind	40.5	14.7	88.3	7.0	88.1	413
Fatehabad	29.2	9.3	81.4	10.1	89.7	423
Sirsa	55.7	7.5	87.8	5.6	88.5	357
Hisar	38.7	11.7	79.7	5.6	82.4	370
Bhiwani	37.8	12.1	81.3	4.1	95.2	445
Rohtak	59.1	11.5	74.5	8.2	88.4	429
Jhajjar	42.7	17.1	95.0	5.0	79.3	380
Mahendragarh	42.3	13.9	76.7	2.8	82.4	378
Rewari	63.5	9.5	86.5	4.9	97.1	402
Gurgaon	61.1	8.9	77.1	8.5	94.7	592
Faridabad	33.5	19.8	80.6	4.1	84.6	531
Mewat	16.6	23.0	74.8	9.1	75.9	1,058
**Haryana (15-49)**	43.3	16.5	83.3	8.3	88.1	9,011
**Haryana (15-44)**[†]	45.0	16.6	82.0	8.3	88.1	8,905

Note; Table based on women with youngest living child born since 01.01.2004.
** Unweighted cases.
[1] Last two weeks prior to survey.
[2] Among children with ARI or fever in last two weeks who sought advice/ treatment.
[†] Represents figures for children of currently married women aged 15-44 years.

FAMILY PLANNING

TABLE 6.1 AWARENESS OF CONTRACEPTIVE METHODS

Percentage of ever married and currently married women aged 15-49 years who are aware of specific contraceptive method by place of residence, Haryana, 2007-08

	Ever married women			Currently married women		
Contraceptive methods	Total	Rural	Urban	Total	Rural	Urban
Any method	99.9	99.9	100.0	100.0	99.9	100.0
Any modern method[1]	99.9	99.9	100.0	99.9	99.9	100.0
Female sterilization	99.8	99.7	99.9	99.8	99.7	99.9
Male sterilization	95.8	95.0	98.1	95.9	95.2	98.1
Intra Uterine Device	93.0	91.6	97.2	93.3	91.9	97.3
Pill	95.9	95.0	98.8	96.1	95.2	98.8
Emergency contraceptive pill	23.7	18.7	38.9	24.3	19.1	39.7
Injectables	71.0	69.1	76.9	71.5	69.6	77.3
Condom/Nirodh	93.2	91.7	97.6	93.5	92.1	97.8
Female condom	6.0	4.6	10.2	6.2	4.7	10.5
Rhythm method	68.8	67.8	72.0	69.4	68.3	72.5
Withdrawal method	50.8	48.9	56.7	51.6	49.6	57.4
Others	0.6	0.6	0.4	0.6	0.6	0.4
Number of women**	21,484	16,154	5,330	20,394	15,306	5,088

** Unweighted cases.
[1]Includes female sterilization, male sterilization, Intra Uterine Device, pill, condom, female condom, Emergency Contraceptive Pill and Injectables

TABLE 6.2 AWARENESS OF CONTRACEPTIVE METHODS

Percentage of currently married women aged 15-49 years who are aware of specific contraceptive method according to selected background characteristics, Haryana, 2007-08

Background characteristics	Any method	Any modern method	Male sterilization	Female sterilization	IUD	Pill	ECP	Injectables	Condom/*nirodh*	Female condom	Rhythm method	Withdrawal method	Other	Number of women**
**Age group**														
15-24	99.9	99.9	94.5	99.5	91.0	96.1	26.9	71.1	94.2	6.8	64.8	47.2	0.4	5,116
25-29	99.9	99.9	96.7	99.8	95.6	97.2	31.6	76.1	96.1	7.6	71.9	56.0	0.8	3,936
30-34	100.0	100.0	96.0	99.9	95.5	96.9	25.0	73.3	94.9	6.3	72.4	55.3	0.8	3,787
35-39	100.0	100.0	96.2	99.9	93.8	95.9	20.0	70.3	93.1	5.5	70.1	52.5	0.5	3,167
40-49	100.0	100.0	96.4	100.0	91.5	94.5	16.9	67.3	89.3	4.4	69.3	48.8	0.5	4,388
**No. of living children**														
0	99.8	99.7	92.6	99.1	86.6	94.3	27.1	65.5	91.8	8.0	58.2	39.5	0.4	2,161
1	100.0	100.0	96.0	99.8	94.4	97.5	35.2	73.6	96.3	10.1	69.4	54.5	0.4	3,038
2	100.0	100.0	96.8	99.9	95.6	97.5	30.6	75.0	95.8	7.5	73.4	56.4	0.7	6,400
3	99.9	99.9	96.2	99.8	94.5	96.2	19.4	70.9	93.4	4.7	68.8	52.2	0.5	4,706
4+	100.0	100.0	95.7	100.0	90.8	93.8	10.4	68.6	88.9	1.8	69.5	47.5	0.8	4,089
**Residence**														
Rural	99.9	99.9	95.2	99.7	91.9	95.2	19.1	69.6	92.1	4.7	68.3	49.6	0.6	15,306
Urban	100.0	100.0	98.1	99.9	97.3	98.8	39.7	77.3	97.8	10.5	72.5	57.4	0.4	5,088
**Education**														
Non-literate[a]	99.9	99.9	93.5	99.7	88.0	92.3	8.9	63.5	87.2	1.9	65.4	43.9	0.5	8,310
Less than five years	100.0	100.0	95.8	100.0	91.7	96.2	12.6	72.3	93.4	3.4	65.3	50.7	0.5	869
5-9 years	100.0	100.0	96.9	99.8	95.9	98.3	23.1	74.6	97.1	5.2	68.9	52.7	0.5	6,111
10 or more years	100.0	100.0	98.6	99.9	99.1	99.8	53.1	81.0	99.6	14.8	77.2	63.0	0.8	5,104
**Religion**														
Hindu	100.0	100.0	96.4	99.8	94.0	96.7	25.2	72.0	94.4	6.2	69.3	51.6	0.6	17,769
Muslim	99.9	99.7	92.1	99.6	82.6	87.7	8.1	70.3	81.3	1.6	75.0	50.5	0.7	1,369
Sikh	99.9	99.9	92.9	99.5	95.9	97.7	29.5	67.2	96.3	10.4	63.6	52.4	0.7	1,231
Others	100.0	100.0	100.0	100.0	96.8	100.0	55.9	69.3	100.0	22.2	68.5	49.6	0.0	25
**Castes/tribes**														
Scheduled castes	99.9	99.9	94.7	99.6	90.3	95.0	15.7	64.8	91.8	3.1	63.5	44.7	0.8	4,651
Scheduled tribes	98.8	98.8	95.3	98.8	92.9	97.7	28.5	70.3	94.0	9.5	57.9	46.0	0.0	83
Other backward classes	100.0	99.9	95.3	99.8	91.4	95.0	21.0	72.1	92.1	5.2	72.0	54.0	0.6	7,344
Others	100.0	100.0	97.2	99.9	96.6	97.7	32.0	74.8	95.7	8.7	70.5	53.3	0.5	8,316
**Wealth index**														
Lowest	100.0	100.0	88.5	100.0	79.8	85.5	4.6	63.9	77.0	0.9	66.7	40.2	0.4	236
Second	99.9	99.9	90.7	99.7	81.0	86.8	5.7	56.3	80.5	1.3	61.9	41.1	0.7	1,185
Middle	99.9	99.8	93.7	99.7	87.8	93.2	9.7	64.8	88.0	2.3	64.3	43.2	0.5	3,930
Fourth	99.9	99.9	95.7	99.7	93.2	96.2	16.9	69.8	93.7	4.0	68.9	49.2	0.6	6,386
Highest	100.0	100.0	98.0	99.9	97.9	99.1	39.5	78.3	98.2	10.4	73.2	58.9	0.6	8,656
Haryana	100.0	99.9	95.9	99.8	93.3	96.1	24.3	71.5	93.5	6.2	69.4	51.6	0.6	20,394

Note: IUD = Intra Uterine Device; ECP = Emergency contraceptive pill.
** Unweighted cases.
[a] Literates but did not attend school, are also included.

TABLE 6.3 AWARENESS OF CONTRACEPTIVE METHODS BY DISTRICT

Percentage of currently married women aged 15-49 years who are aware of specific contraceptive method by district, Haryana, 2007-08

District	Any method	Any modern method	Male sterili-zation	Female sterili-zation	IUD	Pill	ECP	Inject-ables	Condom/ *nirodh*	Female condom	Rhythm method	With-drawal method	Other	Number of women**
Panchkula	100.0	99.9	98.2	99.9	96.8	98.8	42.2	70.7	97.8	11.8	69.3	59.5	1.9	909
Ambala	99.5	99.4	85.2	97.5	86.7	93.7	35.7	55.9	93.7	11.5	52.0	34.8	0.5	946
Yamunanagar	99.9	99.9	95.2	99.5	89.5	95.1	35.9	60.7	94.6	8.1	63.2	49.4	0.3	1,019
Kurukshetra	99.8	99.8	91.5	99.6	93.2	97.6	35.9	65.3	95.1	13.5	54.4	44.4	3.0	949
Kaithal	100.0	100.0	95.9	100.0	96.8	98.6	19.2	64.6	96.4	4.5	59.7	54.1	0.2	1,039
Karnal	100.0	100.0	98.8	100.0	95.1	98.2	33.0	72.6	96.9	11.7	58.0	50.4	1.9	971
Panipat	100.0	100.0	98.9	100.0	95.4	99.0	33.0	76.5	97.6	13.3	69.7	53.6	0.5	980
Sonipat	100.0	100.0	99.7	100.0	96.5	98.2	38.8	79.3	97.3	11.9	78.8	57.8	0.4	998
Jind	100.0	100.0	96.8	100.0	91.1	93.3	20.0	61.6	85.9	4.8	84.6	60.3	0.3	1,008
Fatehabad	100.0	100.0	94.1	100.0	96.2	97.1	11.7	69.7	97.6	2.2	73.3	51.6	0.3	1,000
Sirsa	100.0	100.0	95.4	99.7	93.9	96.2	19.8	66.0	96.7	4.3	60.0	51.6	0.1	1,040
Hisar	100.0	100.0	93.8	100.0	93.4	95.4	25.1	78.9	90.1	3.4	60.3	40.0	0.6	950
Bhiwani	100.0	100.0	94.9	100.0	86.6	91.2	20.3	64.7	87.0	5.9	82.2	56.1	0.2	1,048
Rohtak	100.0	100.0	98.8	100.0	98.5	99.4	24.0	79.2	99.2	5.2	82.0	65.3	0.1	981
Jhajjar	100.0	100.0	95.8	100.0	94.1	99.0	20.9	75.6	94.9	3.0	56.1	31.4	0.3	964
Mahendragarh	100.0	100.0	99.3	100.0	90.5	90.7	20.3	78.9	89.4	5.0	87.4	65.3	0.3	1,057
Rewari	100.0	100.0	98.1	100.0	97.8	99.2	20.8	77.8	97.1	3.2	66.6	57.1	0.1	1,076
Gurgaon	100.0	100.0	98.9	100.0	97.7	99.1	25.9	81.9	99.1	6.0	84.7	62.2	0.2	1,212
Faridabad	100.0	100.0	97.0	99.8	96.9	97.7	23.3	82.5	94.4	4.8	53.4	35.8	1.0	960
Mewat	100.0	99.8	93.0	99.8	82.2	88.0	7.3	77.2	80.9	0.9	80.4	56.9	0.3	1,287
Haryana	100.0	99.9	95.9	99.8	93.3	96.1	24.3	71.5	93.5	6.2	69.4	51.6	0.6	20,394

Note: IUD = Intra Uterine Device; ECP = Emergency contraceptive pill.
** Unweighted cases.

TABLE 6.4 EVER USE OF CONTRACEPTIVE METHOD

Percentage of currently married women aged 15-49 years who ever used specific contraceptive method according to selected background characteristics, Haryana, 2007-08

Background characteristics	Any method	Any modern method	Male sterili-zation	Female sterili-zation	IUD	Pill	ECP	Inject-ables	Condom /*nirodh*	Female condom	Rhythm method	With-drawal method	Other	Number of women**
**Age group**														
15 - 19	23.5	14.2	0.0	0.8	0.7	2.9	0 .2	0.1	11.1	0.1	10.1	6.1	0.0	1,155
20 - 24	51.9	38.5	0.4	9.0	5.1	8.5	0.6	0.4	24.1	0.1	21.1	9.8	0.2	3,961
25 - 29	76.8	67.7	1.0	30.1	11.6	14.2	0.8	0.7	31.1	0.0	24.0	12.0	0.4	3,936
30 - 34	85.7	78.6	1.4	48.4	13.9	14.4	0.6	0.7	25.5	0.1	25.4	11.9	0.4	3,787
35 - 39	87.8	80.6	1.6	57.4	12.8	12.8	0.4	0.6	18.6	0.1	25.0	10.3	0.3	3,167
40 - 44	86.4	79.2	1.8	60.9	12.5	7.9	0.2	0.5	15.2	0.1	24.7	9.3	0.3	2,569
45 – 49	83.8	76.5	2.8	61.3	10.2	4.7	0.1	0.4	11.4	0.1	22.7	7.3	0.1	1,819
**No. of living children**														
0	16.3	10.0	0.0	0.0	0.1	1.2	0.1	0.0	9.1	0.0	6.5	3.7	0.1	2,161
1	57.2	43.7	0.3	4.5	7.4	9.4	0.9	0.6	31.5	0.1	22.0	12.1	0.2	3,038
2	86.4	78.0	1.5	41.1	16.0	14.7	0.7	0.6	30.6	0.1	25.1	12.8	0.2	6,400
3	88.6	82.3	1.6	61.0	11.2	11.6	0.4	0.6	18.4	0.1	25.4	9.8	0.3	4,706
4+	81.5	70.8	2.0	54.7	8.1	9.2	0.3	0.5	12.1	0.1	26.6	8.5	0.6	4,089
**Residence**														
Rural	73.6	64.7	1.3	42.1	8.7	10.1	0.4	0.5	18.3	0.1	23.5	9.2	0.3	15,306
Urban	75.6	66.7	1.0	28.0	15.2	12.2	0.8	0.7	32.6	0.1	21.5	13.1	0.2	5,088
**Education**														
Non-literate[a]	72.8	63.6	1.6	51.3	5.5	6.5	0.2	0.4	8.7	0.0	22.7	6.6	0.3	8,310
Less than five years	72.9	65.1	0.9	45.0	8.7	10.5	0.1	0.7	16.6	0.1	22.8	7.7	0.3	869
5-9 years	75.0	66.6	1.3	38.5	11.2	13.1	0.4	0.6	25.0	0.1	23.2	11.1	0.3	6,111
10 or more years	75.3	66.1	0.7	16.6	17.4	14.5	1.2	0.6	40.7	0.2	23.3	15.4	0.3	5,104
**Religion**														
Hindu	76.5	68.2	1.3	41.7	10.1	10.7	0.5	0.5	22.2	0.1	23.5	10.3	0.3	17,769
Muslim	40.6	22.8	0.5	6.3	3.7	7.2	0.4	0.3	11.2	0.0	22.0	7.9	0.5	1,369
Sikh	80.4	74.3	1.2	33.8	20.8	14.1	0.5	1.0	30.6	0.3	17.3	11.0	0.2	1,231
Others	80.6	68.1	0.0	33.0	16.4	19.5	0.0	4.1	39.4	0.0	24.7	16.6	0.0	25
**Castes/tribes**														
Scheduled castes	70.4	61.1	1.7	42.0	4.6	8.5	0.3	0.4	16.4	0.1	22.1	8.0	0.4	4,651
Scheduled tribes	73.5	63.8	2.3	33.7	7.3	13.3	1.2	1.1	22.9	2.4	19.6	9.6	0.0	83
Other backward classes	70.5	60.5	1.2	36.8	7.8	10.0	0.4	0.5	20.4	0.0	24.0	10.5	0.3	7,344
Others	79.3	71.7	1.1	38.3	15.8	12.3	0.7	0.5	26.3	0.1	22.7	11.1	0.2	8,316
**Wealth index**														
Lowest	49.5	32.8	1.3	24.9	0.8	3.8	0.5	0.4	4.6	0.0	25.5	5.6	0.4	236
Second	60.1	49.0	0.6	39.3	2.9	6.9	0.1	0.4	6.9	0.0	22.1	5.7	0.5	1,185
Middle	69.3	59.2	1.4	45.0	4.5	7.4	0.2	0.4	11.0	0.1	22.7	6.9	0.3	3,930
Fourth	73.9	65.0	1.3	44.0	7.5	9.4	0.3	0.5	16.9	0.1	23.4	8.9	0.3	6,386
Highest	79.1	71.3	1.2	32.0	16.3	13.7	0.8	0.6	33.1	0.1	23.0	13.4	0.3	8,656
**Haryana (15-49)**	74.1	65.2	1.3	38.6	10.3	10.6	0.5	0.5	21.9	0.1	23.0	10.2	0.3	20,394
**Haryana (15-44)** †	73.1	64.1	1.1	36.4	10.3	11.2	0.5	0.5	22.9	0.1	23.1	10.5	0.3	18,575

Note: IUD = Intra Uterine Device; ECP = Emergency contraceptive pill.
** Unweighted cases.
[a] Literates but did not attend school, are also included. † Represents figures for currently married women aged 15-44 years.

TABLE 6.5 (A) CURRENT USE OF CONTRACEPTIVE METHOD

Percentage of currently married women aged 15-49 years who are currently using specific contraceptive method according to selected background characteristics, Haryana, 2007-08

Background characteristics	Any method	Any modern method	Male sterili-zation	Female sterili-zation	IUD	Pill	ECP	Condom/ *nirodh*	Rhythm method	With-drawal method	Other	Number of women**
**Age group**												
15 - 19	12.5	7.1	0.0	0.8	0.3	1.1	0.3	4.6	3.5	1.9	0.0	1,155
20 - 24	33.5	25.5	0.4	9.1	2.8	2.1	0.7	10.4	5.9	1.9	0.0	3,961
25 - 29	64.1	56.0	0.9	30.0	5.4	3.5	0.9	15.0	5.2	2.8	0.0	3,936
30 - 34	78.5	70.9	1.3	48.4	5.5	2.8	0.6	12.2	4.7	2.7	0.0	3,787
35 - 39	81.5	73.2	1.5	57.4	3.6	2.0	0.5	8.2	5.5	2.7	0.0	3,167
40 - 44	77.4	71.5	1.7	60.7	2.1	0.6	0.1	6.2	3.9	1.9	0.0	2,569
45 – 49	71.5	68.5	2.7	61.3	1.0	0.2	0.1	3.1	1.9	1.1	0.0	1,819
**No. of living children**												
No children	4.8	3.1	0.0	0.0	0.0	0.1	0.1	2.8	1.1	0.5	0.0	2,161
1 child	37.1	27.2	0.3	4.5	3.8	2.4	0.6	15.4	7.0	2.9	0.0	3,038
1 son	45.0	34.2	0.4	7.2	5.6	2.7	0.9	17.2	7.2	3.6	0.0	1,798
No son	25.8	17.1	0.1	0.6	1.1	2.0	0.2	12.9	6.8	1.8	0.0	1,240
2 children	76.1	67.7	1.4	41.0	6.0	3.2	0.9	15.2	5.2	3.2	0.0	6,400
1 or more sons	79.8	71.5	1.5	44.4	6.2	3.3	0.9	15.2	5.2	3.0	0.0	5,880
No sons	34.2	24.5	0.1	2.8	3.7	1.9	0.5	15.0	4.6	5.1	0.0	520
3 children	81.2	75.4	1.4	61.0	3.1	1.9	0.5	7.5	3.9	1.8	0.0	4,706
1 or more sons	83.3	77.8	1.5	63.2	3.2	1.9	0.5	7.4	3.7	1.8	0.0	4,526
No sons	28.6	17.5	0.0	4.6	1.7	2.9	0.0	8.2	9.1	2.0	0.0	180
4+ children	71.4	63.8	1.8	54.6	2.0	1.3	0.3	3.7	5.3	1.9	0.0	4,089
1 or more sons	72.6	64.9	1.8	55.7	2.0	1.3	0.3	3.7	5.3	2.0	0.0	4,002
No sons	14.4	9.4	0.0	1.0	2.3	2.3	0.0	3.7	3.7	1.2	0.0	87
**Residence**												
Rural	62.8	56.4	1.3	42.1	3.1	1.9	0.5	7.4	4.5	1.8	0.0	15,306
Urban	63.1	53.8	0.9	27.9	4.8	2.7	0.6	16.9	5.4	3.8	0.0	5,088
**Education**												
Non-literate[a]	64.0	58.4	1.4	51.2	1.4	1.0	0.2	2.9	4.3	1.2	0.0	8,310
Less than five years	63.6	57.4	0.9	45.0	2.7	2.3	0.5	5.8	3.9	2.1	0.0	869
5-9 years	62.8	55.6	1.2	38.4	3.4	2.2	0.6	9.6	4.8	2.4	0.0	6,111
10 or more years	61.0	51.3	0.7	16.6	7.4	3.6	0.9	21.9	5.6	3.9	0.0	5,104
**Religion**												
Hindu	65.9	58.8	1.2	41.6	3.5	2.1	0.5	9.9	4.7	2.3	0.0	17,769
Muslim	22.8	14.4	0.6	6.1	1.4	1.3	1.0	3.9	6.4	1.8	0.0	1,369
Sikh	69.1	62.1	1.0	33.8	7.5	3.4	0.3	16.0	3.9	3.1	0.0	1,231
Others	64.1	51.7	0.0	33.0	4.0	0.0	0.0	14.7	8.2	4.3	0.0	25

Contd …

TABLE 6.5 (A) CURRENT USE OF CONTRACEPTIVE METHOD —*Continued*

Background characteristics	Any method	Any modern method	Male sterili-zation	Female sterili-zation	IUD	Pill	ECP	Condom/ *nirodh*	Rhythm method	With-drawal method	Other	Number of women**
**Castes/tribes**												
Scheduled castes	60.0	53.5	1.5	42.0	1.4	1.5	0.3	6.7	4.8	1.6	0.0	4,651
Scheduled tribes	58.0	54.3	2.3	33.7	3.6	2.2	1.4	11.0	3.7	0.0	0.0	83
Other backward classes	58.8	51.4	1.1	36.7	2.4	1.9	0.4	8.7	5.0	2.2	0.0	7,344
Others	68.3	61.0	1.0	38.3	5.8	2.6	0.8	12.4	4.5	2.7	0.0	8,316
**Wealth index**												
Lowest	37.5	27.9	1.3	24.9	0.4	0.0	0.5	0.9	8.3	0.9	0.0	236
Second	50.7	44.2	0.4	39.3	0.9	1.4	0.4	1.6	4.9	1.3	0.0	1,185
Middle	59.5	53.4	1.2	45.0	1.3	1.1	0.4	4.1	4.8	1.3	0.0	3,930
Fourth	63.0	56.8	1.3	44.0	2.7	1.7	0.5	6.6	4.3	1.8	0.0	6,386
Highest	66.8	58.5	1.2	31.9	5.6	3.0	0.7	16.1	5.0	3.2	0.0	8,656
**Haryana (15-49)**	62.9	55.7	1.2	38.5	3.5	2.1	0.5	9.8	4.8	2.3	0.0	20,394
**Haryana (15-44)** [†]	62.0	54.5	1.0	36.3	3.8	2.3	0.6	10.4	5.0	2.4	0.0	18,575

Note: IUD = Intra Uterine Device; ECP = Emergency contraceptive pill.
** Unweighted cases.
[a] Literates but did not attend school, are also included.
[†] Represents figures for currently married women aged 15-44 years.

TABLE 6.5 (B) DURATION OF USE OF SPACING METHOD

Percentage of currently married women aged 15-49 years who are currently using spacing method by duration of use according to selected background characteristics, Haryana, 2007-08

Background characteristics	IUD < 2 years	IUD 2-3 years	IUD 4 or more years	IUD Number of IUD users**	Pill > 6 months	Pill Number of Pill users**	Condom/*Nirodh* > 6 months	Condom/*Nirodh* Number of condom/ *Nirodh* users	Spacing method[c†] > 6 months
**Age group**									
15 - 19	*	*	*	4	(20.0)	15	27.5	55	1.9
20 - 24	74.1	22.1	3.7	113	53.6	109	49.5	415	9.7
25 - 29	56.3	32.9	10.8	210	64.0	177	71.0	592	19.8
30 - 34	35.6	37.9	26.5	211	80.1	130	79.6	464	18.3
35 - 39	31.9	36.5	31.6	113	82.8	75	88.1	261	12.9
40 - 44	12.3	44.6	43.0	53	(84.2)	19	88.2	160	8.4
45 - 49	(17.6)	(58.8)	(23.5)	18	*	5	89.6	58	na
**No. of living children**									
0	*	*	*	1	*	5	28.2	62	0.8
1	59.9	26.1	14.1	114	59.4	93	60.6	472	16.2
2	44.8	35.8	19.4	377	68.8	257	77.0	968	21.8
3	37.7	38.1	24.2	148	74.9	111	76.9	353	11.7
4+	38.1	36.4	25.5	82	72.2	64	75.4	150	7.0
**Residence**									
Rural	45.3	35.6	19.1	481	65.4	367	66.6	1,136	10.4
Urban	44.3	33.2	22.5	241	74.2	163	78.1	869	22.6
**Education**									
Non-literate[a]	43.4	32.8	23.8	116	75.1	107	72.9	245	4.9
Less than five years	(45.8)	(25.0)	(29.8)	24	(54.2)	24	67.1	50	8.7
5-9 years	39.5	40.7	19.8	207	74.2	169	69.1	585	13.0
10 or more years	48.4	32.8	18.8	375	61.8	230	72.7	1,125	28.6
**Religion**									
Hindu	45.9	35.0	19.1	609	68.2	454	71.8	1,750	13.4
Muslim	(45.0)	(35.0)	(20.0)	20	61.0	31	65.9	55	5.4
Sikh	38.6	33.6	27.8	92	73.6	45	70.6	196	23.9
Others	*	*	*	1	nc	0	*	4	21.6
**Castes/tribes**									
Scheduled castes	34.6	45.9	19.5	61	64.4	84	65.4	312	7.7
Scheduled tribes	*	*	*	3	*	3	*	9	17.3
Other backward classes	52.8	30.0	17.2	173	66.1	168	70.8	649	11.1
Others	43.7	34.9	21.4	485	70.2	275	73.8	1,035	18.8
**Wealth index**									
Lowest	*	*	*	1	*	1	*	2	1.1
Second	(63.6)	(9.1)	(27.3)	11	(50.0)	22	(57.9)	19	2.8
Middle	44.1	34.6	21.3	53	65.5	61	63.5	162	5.3
Fourth	46.4	35.4	18.3	174	69.0	135	69.6	422	9.4
Highest	44.3	35.1	20.6	482	69.5	311	73.3	1,400	22.6
Haryana	45.0	34.8	20.2	722	68.2	530	71.5	2,005	13.4

Note: IUD = Intra Uterine Device.
( ) Based on 10-24 unweighted cases.
* Percentage not shown, based on less than 10 unweighted cases.
** Unweighted cases.
[a] Literates but did not attend school, are also included.
[c] Spacing method includes IUD, daily/weekly pills, Injectables, condom/*nirodh* and female condom. Percentage calculated out of total women but excluding pregnant women.
[†] Represents figures for currently married women aged 15-44 years.

TABLE 6.6 AGE AT THE TIME OF STERILIZATION

Percent distribution of women aged 15-49 years by age at the time of sterilization, according to selected background characteristics, Haryana, 2007- 2008

	Age at the time of sterilization								
Background characteristics	<20	20 -24	25 -29	30 -34	35 -39	40 +	Total[1]	Mean age of sterilization	Number of Women**
**Years since sterilization**									
<2	1.9	36.8	35.1	18.9	4.9	2.5	100.0	26.9	732
2-3	1.6	35.5	38.3	17.9	5.0	1.7	100.0	26.8	822
4-5	1.9	33.0	41.5	17.4	5.2	1.0	100.0	26.8	934
6-7	1.8	30.7	40.6	19.5	6.3	1.2	100.0	27.1	937
8-9	2.9	30.5	39.1	20.5	6.2	0.9	100.0	26.8	832
10+	2.6	31.4	45.2	18.2	2.4	0.0	100.0	26.3	3,934
**No. of living children**									
0	nc	nc	nc	nc	nc	nc	100.0	nc	0
1	7.3	50.6	35.9	4.1	0.7	1.3	100.0	24.0	148
2	5.2	49.9	34.5	9.0	1.1	0.2	100.0	24.7	2,740
3	1.0	32.0	48.2	15.1	3.2	0.4	100.0	26.4	2,950
4+	0.2	11.0	43.4	34.8	8.8	1.7	100.0	29.3	2,355
**Residence**									
Rural	2.4	33.7	41.8	17.7	3.9	0.6	100.0	26.5	6,724
Urban	2.1	26.1	43.4	22.4	4.6	1.3	100.0	27.2	1,469
**Education**									
Non-literate[a]	2.3	26.8	42.6	22.0	5.2	1.1	100.0	27.2	4,456
Less than five years	3.2	37.0	45.3	11.4	3.0	0.0	100.0	25.8	403
5-9 years	2.6	40.6	40.9	13.1	2.4	0.4	100.0	25.7	2,441
10 or more years	0.9	35.1	41.1	19.3	3.1	0.5	100.0	26.5	893
**Religion**									
Hindu	2.4	33.0	41.7	18.1	4.0	0.7	100.0	26.5	7,658
Muslim	0.0	31.3	30.9	28.9	6.3	2.5	100.0	27.8	99
Sikh	1.4	19.8	50.3	23.2	4.6	0.7	100.0	27.6	428
Others	*	*	*	*	*	*	100.0	*	8
**Castes/tribes**									
Scheduled castes	2.0	27.2	43.2	21.3	5.1	1.2	100.0	27.2	2,029
Scheduled tribes	6.5	30.2	36.1	17.6	6.5	3.1	100.0	27.2	30
Other backward classes	2.4	33.3	41.6	18.4	3.7	0.6	100.0	26.5	2,847
Others	2.4	34.7	41.8	16.9	3.6	0.6	100.0	26.3	3,287
**Wealth index**									
Lowest	3.1	26.8	42.5	12.4	9.7	5.5	100.0	27.9	64
Second	2.2	32.0	40.0	20.5	4.7	0.6	100.0	26.9	485
Middle	2.4	33.4	39.9	18.8	4.6	0.9	100.0	26.7	1,849
Fourth	2.7	33.4	41.8	17.5	4.0	0.6	100.0	26.4	2,914
Highest	1.9	30.6	44.0	19.3	3.4	0.7	100.0	26.7	2,881
Haryana	2.3	32.3	42.1	18.5	4.0	0.7	100.0	26.6	8,193

nc = Not calculated because there are no cases.
* Percentage not shown, based on less than 10 unweighted cases.
** Unweighted cases.
[1] Total figure may not add to 100 percent due to ‘don’t know’ or ‘missing cases’.
[a] Literates but did not attend school, are also included.

TABLE 6.7 CONTRACEPTIVE PREVALENCE RATE BY DISTRICT

Percentage of currently married women aged 15-49 years who are currently using any contraceptive method, by districts, Haryana, 2007-08

District	Any method	Any modern method	Male sterili-zation	Female sterili-zation	IUD	Pill	ECP	Condom/ *nirodh*	Rhythm method	With-drawal method	Other	Number of Women**
Panchkula	66.7	58.9	1.3	29.8	5.0	2.9	1.0	18.9	5.1	2.5	0.0	909
Ambala	65.4	61.3	0.9	36.7	2.9	3.4	0.3	17.2	1.9	1.9	0.0	946
Yamunanagar	63.8	56.2	1.2	33.6	1.7	3.5	0.3	15.8	4.3	3.2	0.0	1,019
Kurukshetra	65.6	58.5	1.4	33.0	3.6	3.1	0.0	17.3	4.0	2.9	0.0	949
Kaithal	67.0	60.1	1.9	38.3	5.8	2.6	0.9	10.7	4.6	2.2	0.0	1,039
Karnal	64.3	56.7	3.5	33.2	3.2	2.7	0.2	13.8	4.4	3.0	0.0	971
Panipat	59.1	46.8	0.8	29.5	3.4	1.6	0.9	10.4	7.1	4.9	0.0	980
Sonipat	60.8	52.9	1.0	33.3	5.7	2.4	0.8	9.6	5.3	2.4	0.0	998
Jind	66.2	60.5	1.5	45.1	5.1	2.6	0.8	5.5	4.5	1.2	0.0	1,008
Fatehabad	67.3	61.1	0.1	46.3	2.9	1.9	0.3	9.4	4.1	1.8	0.0	1,000
Sirsa	69.4	64.3	0.3	45.3	4.0	2.0	0.2	12.4	3.1	2.0	0.0	1,040
Hisar	67.6	64.2	0.2	50.6	4.6	1.1	0.7	6.7	2.8	0.6	0.0	950
Bhiwani	68.3	61.9	1.0	52.4	1.8	1.6	0.0	5.0	5.6	0.9	0.0	1,048
Rohtak	66.8	56.3	1.4	37.0	5.3	1.2	0.9	10.5	6.0	4.4	0.0	981
Jhajjar	61.5	56.3	1.0	41.5	4.1	1.8	0.8	6.9	3.4	1.8	0.0	964
Mahendragarh	71.3	65.5	1.3	53.1	2.0	1.7	0.4	6.9	4.3	1.4	0.0	1,057
Rewari	69.8	61.5	1.5	49.4	0.8	1.5	0.6	7.7	5.5	2.7	0.0	1,076
Gurgaon	65.0	55.2	1.0	37.7	3.6	2.3	0.7	9.7	6.6	3.3	0.0	1,212
Faridabad	54.4	45.4	0.5	28.2	4.6	1.8	0.1	9.8	6.8	1.6	0.0	960
Mewat	27.9	18.7	0.4	12.7	1.7	0.6	0.4	2.9	7.1	2.0	0.0	1,287
**Haryana (15-49)**	62.9	55.7	1.2	38.5	3.5	2.1	0.5	9.8	4.8	2.3	0.0	20,394
**Haryana (15-44)** †	62.0	54.5	1.0	36.3	3.8	2.3	0.6	10.4	5.0	2.4	0.0	18,575

Note: IUD = Intra Uterine Device; ECP = Emergency contraceptive pill.
** Unweighted cases.
† Represents figures for currently married women aged 15-44 years.

TABLE 6.8 SOURCES OF MODERN CONTRACEPTIVE METHOD

Percent distribution of currently married women aged 15-49 years who are currently using modern contraceptive methods by source according to selected background characteristics, Haryana, 2007-08

Background Characteristics	Spacing Method				Number of women**	Limiting method				Number of women**
	Govern-ment[2]	Private[3]	Other[4]	Total[1]		Govern-ment[5]	Private[6]	Other[7]	Total[1]	
**Age group**										
15 - 19	10.5	50.7	38.7	100.0	70	(90.0)	(10.0)	(0.0)	100.0	10
20 - 24	22.4	41.3	36.3	100.0	526	78.8	21.2	0.0	100.0	382
25 - 29	18.1	46.6	35.3	100.0	774	84.7	15.1	0.2	100.0	1,234
30 - 34	17.8	50.6	31.6	100.0	598	85.7	14.1	0.2	100.0	1,902
35 - 39	21.5	46.7	31.8	100.0	339	89.2	10.5	0.3	100.0	1,883
40 - 44	13.6	48.7	37.7	100.0	182	92.2	7.7	0.1	100.0	1,612
45 – 49	11.3	42.8	45.9	100.0	63	91.5	8.1	0.4	100.0	1,170
**No. of living children**										
0	4.4	58.9	36.7	100.0	67	nc	nc	nc	100.0	0
1	15.2	48.4	36.3	100.0	569	88.0	11.3	0.7	100.0	148
2	18.4	45.8	35.8	100.0	1,230	83.8	15.9	0.3	100.0	2,740
3	22.7	44.0	33.2	100.0	467	89.2	10.6	0.2	100.0	2,950
4+	24.6	48.6	26.7	100.0	219	91.8	7.9	0.2	100.0	2,355
**Residence**										
Rural	25.4	42.9	31.8	100.0	1,515	89.2	10.6	0.2	100.0	6,724
Urban	8.8	52.2	39.0	100.0	1,037	83.1	16.4	0.5	100.0	1,469
**Education**										
Non-literate[a]	27.4	40.5	32.1	100.0	359	91.8	8.0	0.2	100.0	4,456
Less than five years	24.1	49.4	26.5	100.0	75	89.3	10.7	0.0	100.0	403
5-9 years	23.4	41.2	35.4	100.0	759	86.1	13.7	0.2	100.0	2,441
10 or more years	13.3	51.2	35.5	100.0	1,359	74.6	24.6	0.8	100.0	893
**Religion**										
Hindu	18.3	47.1	34.6	100.0	2,219	88.2	11.6	0.2	100.0	7,658
Muslim	21.9	42.1	36.0	100.0	87	88.3	11.7	0.0	100.0	99
Sikh	21.0	44.6	34.4	100.0	242	86.6	12.9	0.5	100.0	428
Others	*	*	*	100.0	4	*	*	*	100.0	8
**Castes/tribes**										
Scheduled castes	28.7	40.9	30.4	100.0	401	94.4	5.3	0.3	100.0	2,029
Scheduled tribes	(25.0)	(25.0)	(50.0)	100.0	12	96.7	3.3	0.0	100.0	30
Other backward classes	21.9	43.3	34.9	100.0	823	90.5	9.4	0.1	100.0	2,847
Others	13.5	50.7	35.8	100.0	1,316	82.1	17.6	0.3	100.0	3,287
**Wealth index**										
Lowest	*	*	*	100.0	3	95.2	4.8	0.0	100.0	64
Second	39.1	32.7	28.2	100.0	43	94.8	4.7	0.4	100.0	485
Middle	34.5	37.8	27.7	100.0	230	92.1	7.8	0.1	100.0	1,849
Fourth	26.2	39.4	34.3	100.0	559	90.0	9.9	0.2	100.0	2,914
Highest	13.6	50.5	35.9	100.0	1,717	82.5	17.1	0.4	100.0	2,881
Haryana	18.7	46.6	34.7	100.0	2,552	88.1	11.6	0.2	100.0	8,193

Note: Spacing method includes – pill (Daily/Weekly), condom (Male/Female), and Injectables and limiting method includes – male sterilization and female sterilization.
nc = Not calculated because there are no cases.
( ) Based on 10-24 unweighted cases.
* Percentage not shown, based on less than 10 unweighted cases.
** Unweighted cases.
[1] Total figure may not add to 100 percent due to 'missing cases'.
[2] Hospital, dispensary, UHC/UHP/UFWC, CHC/ Rural Hospital, PHC, Sub-Centre/ANM, Mobile clinic, *Anganwadi*/ICDS centre, ASHA, Other Community Based Worker, AYUSH Hospital/Clinic and Other Public Health Sector.
[3] Hospital, Doctor/Clinic, Mobile Clinic, AYUSH Hospital/Clinic, Traditional Healer, Pharmacy/Drugstore and Other Private Medical Sector.
[4] NGO or Trust Hospital/clinic Private, Shop, Vending Machine, Husband, Relatives/Friends, Others and Don't Know.
[5] Hospital, Dispensary, CHC/Rural Hospital, PHC, Mobile Clinic, Camp and Other Public Sector Health Facility.
[6] Hospital, Doctor/Clinic, Mobile Clinic and Other Private Health Facility.
[7] NGO or Trust Hospital/Clinic, Other and Don't Know.
[a] Literates but did not attend school, are also included.

TABLE 6.9 CASH BENEFITS RECEIVED AFTER STERILIZATION

Percent distribution of currently married women and wives of sterilized men who received cash benefits after sterilization, by districts, Haryana, 2007-08

District	Received cash benefits	Cash benefits received: At the time of discharge	At the time of first follow-up	After several visits	Total[1]	Number of Women**
Panchkula	70.8	93.1	4.5	2.5	100.0	323
Ambala	57.1	87.1	10.4	2.5	100.0	360
Yamunanagar	62.7	94.2	3.1	2.7	100.0	367
Kurukshetra	63.0	93.7	5.8	0.5	100.0	334
Kaithal	70.2	98.3	1.7	0.0	100.0	427
Karnal	72.0	96.1	2.7	1.2	100.0	367
Panipat	60.0	97.8	0.6	1.7	100.0	313
Sonipat	62.5	97.7	1.9	0.5	100.0	331
Jind	68.9	91.7	8.0	0.3	100.0	476
Fatehabad	62.1	90.5	5.3	4.2	100.0	449
Sirsa	63.7	95.7	3.0	1.3	100.0	488
Hisar	42.1	91.5	5.5	3.0	100.0	485
Bhiwani	63.2	88.9	8.5	2.6	100.0	555
Rohtak	71.2	97.8	1.9	0.4	100.0	394
Jhajjar	68.5	90.0	7.8	2.1	100.0	417
Mahendragarh	74.2	92.8	6.5	0.7	100.0	582
Rewari	78.8	95.3	3.5	1.2	100.0	548
Gurgaon	73.5	92.8	3.2	4.1	100.0	495
Faridabad	66.8	92.4	5.4	2.2	100.0	303
Mewat	67.3	92.9	4.4	2.7	100.0	179
Haryana	66.7	93.7	4.7	1.7	100.0	8,193

** Unweighted cases.
[1] Total figure may not add to 100 percent due to ‘don’t know’ or ‘missing cases’.

TABLE 6.10 HEALTH PROBLEMS WITH CURRENT USE OF CONTRACEPTION AND TREATMENT RECEIVED

Percentage of currently married women aged 15-49 years who are currently using contraceptive method and who were informed about side effects, had side effects with the method, treatment taken for side effect with the method, Haryana, 2007-08

Health problems/side effect	Type of method		
	Female sterilization	IUD	Pill
Women who were informed about the side effects before adoption of the method	31.1	39.7	25.8
Women who had side-effect/health problem due to use of contraceptive method	12.1	11.0	8.4
**Number of current users****	7,951	722	530
**Type of health problems/side effects**[1]			
Weakness/inability to work	16.9	10.4	12.8
Body ache/ backache	32.6	22.2	15.0
Abdominal pain	32.4	32.6	12.2
Weight gain	9.6	8.7	12.7
Dizziness	8.0	6.9	25.6
Nausea/vomiting	3.1	2.7	5.5
Fever	2.6	0.0	0.0
Breast tenderness	0.9	0.0	0.0
Irregular periods	16.4	22.0	28.1
Excessive bleeding	13.2	36.9	11.4
Spotting	0.6	2.5	0.0
Amenorrhoea	0.7	0.0	0.0
Cramps	4.5	0.0	5.7
Decreased libido	1.2	3.6	2.2
Rashes/allergy	2.0	2.5	6.0
Infection	2.2	1.2	0.0
Others	22.8	5.9	15.1
**Number of users with side effects****	960	80	45
**Source of treatment**			
Government health facility	32.2	31.5	15.5
Private health facility	63.8	66.5	84.5
Other	4.0	1.9	0.0
**Number of women with treatment taken****	651	48	25

** Unweighted cases.
[1] Percentages may add to more than 100 because of multiple responses.

TABLE 6.11 REASONS FOR DISCONTINUATION OF CONTRACEPTION

Percent distribution of currently married women aged 15-49 years who are past users (currently non-users) by reason for discontinuation of the contraceptive method according to selected background characteristics, Haryana, 2007-08.

Background characteristics	Reasons for discontinuation			Number of women**
	Fertility related[1]	Side effect related	Others[2]	
**Age group**				
15 - 19	88.5	2.2	9.3	129
20 - 24	85.4	4.9	9.6	728
25 - 29	76.9	8.3	14.8	497
30 - 34	60.2	14.6	25.2	259
35 - 39	41.3	21.3	37.3	169
40 - 44	27.3	22.2	50.5	168
45 - 49	30.6	12.3	57.1	112
**No. of living children**				
0	93.8	0.8	5.4	247
1	82.7	5.1	12.2	602
2	60.6	13.7	25.8	605
3	52.3	18.2	29.5	290
4+	56.2	11.6	32.3	318
**Residence**				
Rural	70.9	9.0	20.1	1,497
Urban	64.3	12.6	23.1	565
**Education**				
Non-literate[a]	67.5	10.3	22.2	614
Less than five years	66.8	7.8	25.4	78
5-9 years	68.7	10.3	20.9	687
10 or more years	71.4	9.4	19.2	683
**Religion**				
Hindu	68.7	10.2	21.1	1,727
Muslim	77.6	4.6	17.8	222
Sikh	58.1	16.3	25.5	111
Others	*	*	*	2
**Castes/tribes**				
Scheduled castes	75.9	8.0	16.1	463
Scheduled tribes	*	*	*	8
Other backward classes	72.8	9.0	18.2	769
Others	61.9	12.1	26.0	822
**Wealth index**				
Lowest	86.7	6.7	6.6	26
Second	77.6	2.8	19.7	102
Middle	73.6	7.8	18.7	352
Fourth	72.4	10.0	17.6	633
Highest	63.8	11.6	24.6	949
Haryana	69.1	9.9	20.9	2,062

* Percentage not shown, based on less than 10 unweighted cases.
** Unweighted cases
[1] Wanted child, method failed/became pregnant.
[2] Others include supply not available, difficult to get method, lack of pleasure, method was inconvenient, cost too much, family/husband opposed, not having sex, infrequent sex, husband away and others.
[a] Literates but did not attend school, are also included.

TABLE 6.12 FUTURE INTENTION TO USE

Percent distribution of currently married women aged 15-49 years who are not using contraceptive method but having intention to use contraception in future by background characteristics, Haryana, 2007-08

Background Characteristics	Future intention to use[1]		Want to use any family planning method			Number of non-users**
	Spacing Method	Limiting Method	Within 12 months	12 months and more	Undecided	
**Age group**						
15 - 19	3.7	12.8	8.4	4.2	87.4	1,010
20 - 24	5.3	20.9	19.0	9.3	71.7	2,621
25 - 29	6.1	25.1	26.2	11.3	62.5	1,404
30 - 34	5.0	18.3	34.2	9.0	56.9	795
35 - 39	1.8	8.4	33.2	7.1	59.7	573
40 - 44	1.7	2.0	48.0	12.6	39.4	572
45 - 49	0.2	0.2	0.0	34.1	65.9	512
**No. of living children**						
0	2.7	8.8	1.9	5.0	93.1	2,057
1	4.8	19.0	13.0	8.1	79.0	1,904
2	5.3	22.6	28.8	10.4	60.7	1,522
3	4.9	22.0	31.8	13.2	55.0	872
4+	4.6	14.0	47.1	10.3	42.6	1,132
**Residence**						
Rural	4.5	17.1	22.4	9.0	68.6	5,610
Urban	3.7	14.7	22.5	10.0	67.5	1,877
**Education**						
Non-literate[a]	3.9	13.8	26.4	10.1	63.5	2,921
Less than five years	3.9	20.6	26.3	7.5	66.2	313
5-9 years	3.7	19.8	22.5	7.3	70.1	2,268
10 or more years	5.7	16.2	17.2	10.6	72.2	1,985
**Religion**						
Hindu	3.8	19.2	21.3	9.4	69.3	6,049
Muslim	6.9	2.6	31.9	6.1	62.0	1,047
Sikh	4.4	15.6	26.8	9.3	63.9	382
Others	*	*	*	*	*	9
**Castes/tribes**						
Scheduled castes	3.6	23.6	21.6	10.0	68.5	1,864
Scheduled tribes	2.8	22.2	37.0	17.9	45.1	35
Other backward classes	4.7	12.7	23.8	9.0	67.2	2,960
Others	4.4	15.9	21.7	8.5	69.9	2,628
**Wealth index**						
Lowest	5.1	10.2	36.9	6.9	56.2	146
Second	5.2	16.2	24.5	6.9	68.6	570
Middle	3.9	16.7	23.6	10.9	65.5	1,560
Fourth	3.6	19.6	21.5	8.7	69.8	2,349
Highest	4.9	14.2	21.6	9.3	69.1	2,862
Haryana	4.3	16.5	22.5	9.2	68.4	7,487

Note: Spacing method includes IUD, pills, condom (Male/Female) and Injectables. Limiting method includes male sterilization and female sterilization.
* Percentage not shown, based on less than 10 unweighted cases.
** Unweighted cases.
[1] Total figure may not add to 100 percent due to exclusion of other methods (Rhythm/ Periodic abstinence, Withdrawal, Undecided and Others).
[a] Literates but did not attend school, are also included.

TABLE 6.13 ADVICE ON CONTRACEPTIVE USE

Percentage of currently married women aged 15-49 years who are currently not using any contraceptive and were advised by the ANM/health worker to use modern contraception by suggested method and place of residence, Haryana, 2007-08

		Residence	
Advice	Total	Rural	Urban
Percent Non-users advised to use modern contraceptive method[1]	9.3	9.5	8.8
**Number of Non-users****	7,487	5,610	1,877
Percent of Traditional method users advised to use modern method	24.7	25.1	23.8
**Number of traditional method users****	1,440	975	465
**Percent of non-users or traditional method users who were advised to use**			
Female sterilization	51.5	54.0	44.7
Male sterilization	2.0	2.1	1.4
IUD	40.3	37.2	48.8
Pill(Daily/weekly)	100.0	100.0	100.0
Injectables	5.5	5.2	6.4
Condom/*nirodh*	31.1	30.1	33.8
Female condom	0.8	0.8	1.1
Rhythmic /periodic abstinence	5.3	5.2	5.7
Withdrawal	2.0	2.0	2.1
Others	0.2	0.0	0.7
Non-users who ever received counselling/advised by health personnel to adopt family planning (15-44) †	12.4	12.7	11.4
**Number of non-users (15-44)**** †	5,334	4,027	1,307
Current users ever told about side-effects of family planning methods (15-44)[v]	27.0	27.9	24.1
**Number of current users (15-44)**** †	10,216	7,784	2,432
Users who received follow-up services for IUD/sterilization within 48 hours (15-44) †	60.9	61.3	58.9
**Number of users (15-44)**** †	7,727	6,282	1,445

Note: Exclude women in menopause or those who have undergone hysterectomy.
** Unweighted cases.
[1] Includes Doctor, ANM, Health Worker, *Anganwadi* Worker and ASHA.
† Represents figures for currently married women aged 15-44 years.

TABLE 6.14 REASONS FOR NOT USING MODERN CONTRACEPTIVE METHODS AMONG RHYTHM AND WITHDRAWAL METHOD USERS

Percent distribution of currently married women aged 15-49 years who are currently using rhythm or withdrawal method by reasons for not using modern contraceptive method, according to selected background characteristics, Haryana, 2007-08

	Reason for not using modern contraceptive method			
Background characteristics	Fertility related	Opposition to use/lack of knowledge	Method related	Number of women**
**Age group**				
15 - 19	3.0	54.9	42.1	61
20 - 24	7.8	38.9	53.3	313
25 - 29	6.2	36.1	57.7	310
30 - 34	5.1	34.5	60.5	276
35 - 39	5.1	33.2	61.7	254
40 - 44	4.8	32.5	62.7	146
45 - 49	7.6	33.5	58.9	56
**No. of living children**				
0	7.2	45.2	47.6	35
1	5.8	35.6	58.5	301
2	5.9	36.3	57.8	528
3	6.7	33.0	60.3	264
4+	5.0	38.5	56.4	288
**Residence**				
Rural	6.3	35.8	57.9	958
Urban	5.0	37.1	57.8	458
**Education**				
Non-literate[a]	5.5	39.8	54.7	447
Less than five years	7.6	32.3	60.0	52
5-9 years	6.7	34.1	59.2	433
10 or more years	5.3	35.2	59.5	484
**Religion**				
Hindu	5.9	35.0	59.2	1,215
Muslim	5.4	55.3	39.3	112
Sikh	7.1	25.5	67.5	86
Others	*	*	*	3
**Castes/tribes**				
Scheduled castes	5.5	36.8	57.7	290
Scheduled tribes	*	*	*	3
Other backward classes	6.5	39.3	54.2	533
Others	5.6	33.0	61.4	590
**Wealth index**				
Lowest	(4.8)	(38.1)	(57.1)	21
Second	5.3	36.7	58.1	72
Middle	7.1	41.1	51.8	235
Fourth	4.1	35.3	60.7	382
Highest	6.6	35.0	58.4	706
Haryana	5.9	36.2	57.9	1,416

( ) Based on 10-24 unweighted cases.
* Percentage not shown, based on less than 10 unweighted cases.
** Unweighted cases.
[a] Literates but did not attend school, are also included.

TABLE 6.15 UNMET NEED FOR FAMILY PLANNING SERVICES

Percentage of currently married women aged 15-49 years by unmet need for family planning services according to selected background characteristics, Haryana, 2007-08

Background characteristics	Unmet need for FP			Number of women**
	Spacing[1]	Limiting[2]	Total	
**Age group**				
15 – 19	22.4	1.3	23.7	1,155
20 - 24	14.3	8.9	23.2	3,961
25 - 29	4.5	11.4	15.9	3,936
30 - 34	1.1	11.0	12.1	3,787
35 - 39	0.3	11.2	11.5	3,167
40 - 44	0.2	12.3	12.5	2,569
45 - 49	0.1	9.4	9.5	1,819
**Number of living children**				
0	7.9	0.3	8.2	2,161
1	18.7	5.8	24.5	3,038
2	2.9	11.8	14.7	6,400
3	1.5	10.0	11.5	4,706
4+	1.7	16.3	18.0	4,089
**Residence**				
Rural	5.6	10.0	15.6	15,306
Urban	4.0	10.7	14.7	5,088
**Education**				
Non-literate[a]	4.2	12.2	16.4	8,310
Less than five years	5.6	10.1	15.7	869
5-9 years	5.7	8.9	14.6	6,111
10 or more years	6.2	8.3	14.5	5,104
**Religion**				
Hindu	4.6	9.0	13.6	17,769
Muslim	13.9	24.0	37.9	1,369
Sikh	2.6	9.3	11.9	1,231
Others	4.1	12.2	16.3	25
**Castes/tribes**				
Scheduled castes	5.8	9.9	15.7	4,651
Scheduled tribes	6.2	9.5	15.7	83
Other backward classes	6.2	11.1	17.3	7,344
Others	4.0	9.5	13.5	8,316
**Wealth index**				
Lowest	8.7	20.3	29.0	236
Second	8.1	16.2	24.3	1,185
Middle	5.9	11.7	17.6	3,930
Fourth	5.8	9.5	15.3	6,386
Highest	3.9	8.8	12.7	8,656
**Haryana (15-49)**	5.2	10.2	15.4	20,394
**Haryana (15-44)** [†]	5.7	10.2	15.9	18,575

Note: Total unmet need refers to unmet for limiting and spacing.

** Unweighted cases.

[1] Unmet need for spacing includes the proportion of currently married women who are neither in menopause or had hysterectomy nor are currently pregnant and who want more children after two years or later and are currently not using any family planning method. The women who are not sure about whether and when to have next child are also included in unmet need for spacing.

[2] Unmet need for limiting includes the proportion of currently married women who are neither in menopause or had hysterectomy nor are currently pregnant and do not want any more children but are currently not using any family planning method. Total unmet need refers to unmet for limiting and spacing.

[a] Literates but did not attend school, are also included.

[†] Represents figures for currently married women aged 15-44 years.

TABLE 6.16 UNMET NEED FOR FAMILY PLANNING SERVICES BY DISTRICT

Percentage of currently married women aged 15-49 years by unmet need for family planning services by districts, Haryana, 2007-08

District	Unmet need for FP			Number of women**
	Spacing[1]	Limiting[2]	Total	
Panchkula	4.6	7.4	12.0	909
Ambala	3.9	10.7	14.7	946
Yamunanagar	3.9	12.2	16.1	1,019
Kurukshetra	2.7	9.6	12.3	949
Kaithal	3.5	8.3	11.8	1,039
Karnal	5.4	10.6	16.0	971
Panipat	4.9	11.6	16.5	980
Sonipat	4.8	10.9	15.7	998
Jind	6.1	7.8	13.9	1,008
Fatehabad	4.4	6.7	11.0	1,000
Sirsa	3.5	7.4	10.8	1,040
Hisar	6.2	7.3	13.5	950
Bhiwani	5.6	8.8	14.4	1,048
Rohtak	4.2	7.8	12.0	981
Jhajjar	7.5	10.6	18.0	964
Mahendragarh	4.1	8.8	12.9	1,057
Rewari	4.2	8.6	12.8	1,076
Gurgaon	3.8	10.0	13.8	1,212
Faridabad	7.7	13.6	21.3	960
Mewat	13.3	21.9	35.1	1,287
**Haryana (15-49)**	5.2	10.2	15.4	20,394
**Haryana (15-44)** †	5.7	10.2	15.9	18,575

Note: Total unmet need refers to unmet for limiting and spacing.
** Unweighted cases.
[1] Unmet need for spacing includes the proportion of currently married women who are neither in menopause or had hysterectomy nor are currently pregnant and who want more children after two years or later and are currently not using any family planning method. The women who are not sure about whether and when to have next child are also included in unmet need for spacing.
[2] Unmet need for limiting includes the proportion of currently married women who are neither in menopause or had hysterectomy nor are currently pregnant and do not want any more children but are currently not using any family planning method.
† Represents figures for currently married women aged 15-44 years.

REPRODUCTIVE HEALTH PROBLEMS AND AWARENESS

TABLE 7.1 MENSTRUATION RELATED PROBLEMS BY BACKGROUND CHARACTERISTICS

Percentage of ever married women aged 15-49 years who had any menstruation related problem during three months prior to survey and among them, reported specific symptoms according to selected background characteristics, Haryana, 2007-08.

Background characteristics	Who had any menstruation related problem (%)	Total number of women[1]**	Reported symptoms among who had any menstruation problem: No periods	Painful periods	Frequent or short periods	Irregular periods	Prolonged bleeding	Scanty bleeding	Inter-menstrual bleeding	Blood clots/ excessive bleeding	Number of women who had menstruation problem**
**Age group**											
15-19	16.0	818	3.9	77.0	3.1	29.4	5.4	4.6	1.6	7.9	132
20-24	15.5	2,597	3.8	57.1	8.3	32.9	8.1	7.0	2.0	9.7	401
25-29	16.6	3,338	4.2	51.4	4.9	29.5	12.4	12.0	2.3	8.6	555
30-34	18.3	3,624	4.2	52.3	8.7	27.9	9.3	17.9	2.8	8.5	667
35-39	19.0	3,091	5.7	46.8	9.1	27.4	10.8	16.7	1.6	12.1	587
40-44	20.0	2,236	4.2	48.7	9.8	32.6	13.5	11.4	1.3	11.3	444
45-49	21.6	1,063	5.7	42.6	10.3	40.6	13.7	10.9	3.9	14.1	230
**Place of residence**											
Rural	18.3	12,585	4.0	52.8	8.0	29.9	11.1	12.7	2.2	10.5	2,286
Urban	17.1	4,182	6.2	47.4	8.4	32.5	9.8	14.1	2.0	9.1	730
**Age at consummation of marriage**											
Below18 years	19.6	7,143	4.6	50.8	8.7	30.0	12.2	12.5	2.7	11.2	1,398
18 years & above	16.8	9,624	4.4	52.1	7.6	30.9	9.5	13.5	1.7	9.3	1,618
**Marital duration**											
0-4	15.2	2,759	3.1	65.4	5.2	29.3	7.5	6.4	1.7	8.2	421
5-9	15.7	2,824	5.0	51.5	6.7	32.5	10.7	10.6	2.4	9.5	443
10-14	18.4	3,098	5.0	50.5	7.9	28.9	9.9	14.9	2.3	8.4	571
15+	19.6	8,086	4.6	48.1	9.3	30.8	12.0	14.8	2.2	11.6	1,581
**Education**											
Non-literate[a]	18.2	6,697	4.6	50.6	8.4	30.1	10.8	13.4	2.4	11.1	1,219
Less than 5 yrs	19.1	732	5.5	55.6	9.2	28.1	8.1	15.4	0.7	11.2	139
5-9 years	19.7	5,036	4.1	54.9	7.5	29.2	11.7	12.1	1.8	9.8	990
10 or more years	15.4	4,302	4.9	47.1	8.2	33.7	9.9	13.3	2.5	8.9	668
**Husband's education**											
Non-literate[a]	17.9	2,900	4.8	51.9	9.5	29.8	12.3	10.1	2.8	11.3	515
Less than 5 yrs	18.6	555	5.1	54.5	12.4	27.5	4.3	16.9	7.4	7.7	103
5-9 years	19.9	5,338	4.9	53.4	8.5	28.5	11.7	12.5	1.7	10.7	1,062
10 or more years	16.7	7,974	4.1	49.6	6.9	32.6	10.0	14.3	1.9	9.5	1,336
**Religion**											
Hindu	18.0	14,845	4.3	51.4	7.9	30.4	10.7	13.1	2.2	10.2	2,668
Muslim	20.7	856	4.4	56.0	9.3	33.6	10.6	11.4	1.8	11.9	178
Sikh	16.0	1,045	7.8	48.4	9.8	27.8	13.3	12.8	1.9	9.0	165
Other	(9.1)	21	*	*	*	*	*	*	*	*	5

Contd…

TABLE 7.1 MENSTRUATION RELATED PROBLEMS BY BACKGROUND CHARACTERISTICS — *Continued*

Background characteristics	Who had any menstruation related problem	Total number of women[1]**	Reported symptoms								Number of women who had menstruation problem**
			No periods	Painful periods	Frequent or short periods	Irregular periods	Prolonged bleeding	Scanty bleeding	Inter-menstrual bleeding	Blood clots/excessive bleeding	
**Castes/Tribes**											
Scheduled Castes	18.4	3,762	5.0	52.2	7.7	30.0	8.8	13.5	2.3	9.8	692
Scheduled Tribes	10.3	68	*	*	*	*	*	*	*	*	7
Other Backward Classes	18.9	5,834	4.5	53.8	7.7	29.5	10.4	11.4	2.2	9.1	1,100
Others	17.1	7,101	4.3	48.9	8.5	31.7	12.2	14.3	2.1	11.4	1,217
**Wealth index**											
Lowest	18.3	175	6.6	53.7	9.9	19.5	9.4	16.2	3.6	9.4	31
Second	18.9	947	1.8	50.9	12.1	31.6	10.6	15.2	2.5	8.0	177
Middle	17.9	3,220	4.2	57.7	8.7	27.8	9.8	11.5	3.0	10.4	578
Fourth	18.8	5,238	5.2	52.7	7.7	30.6	10.1	11.6	1.5	9.7	977
Highest	17.3	7,186	4.5	47.6	7.4	31.8	11.9	14.5	2.2	10.8	1,253
**Haryana**	18.0	16,767	4.5	51.5	8.1	30.5	10.8	13.0	2.2	10.2	3,016

Note: Total figure may not add to 100 percent due to multiple responses.
* Percentage not shown; based on less than 10 unweighted cases.
** Unweighted cases.
( ) Based on 10-24 unweighted cases.
[a] Literates but did not attend school, are also included.
[1] Excludes pregnant, in amenorrhea, in menopause, had hysterectomy and ever menstruated women.

TABLE 7.2 SOURCE OF KNOWLEDGE ABOUT RTI/STI BY BACKGROUND CHARACTERISTICS

Percentage of ever married women aged 15- 49 years who have heard about RTI/STI, among them, who received information from specific sources according to selected background characteristics, Haryana, 2007-08

Background characteristics	Who have heard RTI/STI	Total number of women**	Source of Knowledge										Number of women heard of RTI/STI**
			Radio	T.V.	Cinema	Print media[1]	Health personnel[2]	School/adult education programs[3]	Leaders/ community meeting[4]	Husband	Relative/ friends	Other	
**Age group**													
15-19	32.1	1,235	7.3	44.9	1.0	19.8	7.5	2.7	0.2	2.2	55.3	1.0	399
20-24	43.3	4,012	7.1	56.1	1.9	25.0	11.9	0.7	0.3	4.1	48.6	1.8	1,735
25-29	44.4	4,032	7.8	55.7	1.7	24.7	11.3	0.3	0.9	3.7	49.1	2.2	1,797
30-34	40.7	3,918	7.0	53.5	1.7	21.7	11.6	0.2	1.1	3.2	51.0	2.7	1,605
35-39	37.8	3,364	7.1	46.3	1.3	16.5	12.6	0.2	1.7	2.7	59.3	2.5	1,272
40-44	34.1	2,830	5.0	41.3	1.2	15.7	12.0	0.5	1.5	1.8	63.4	2.7	966
45-49	30.3	2,093	6.2	40.8	2.0	15.5	11.5	0.1	1.8	1.9	64.1	3.2	637
**Residence**													
Rural	36.4	16,154	7.3	46.0	1.3	16.4	11.9	0.4	1.1	3.1	58.0	2.4	5,859
Urban	46.9	5,330	6.2	61.5	2.4	31.9	10.9	0.6	0.8	3.1	44.3	2.2	2,552
**Age at consummation of marriage**													
Below 18 years	31.2	9,243	5.9	35.7	0.6	9.7	10.4	0.3	1.0	2.4	67.8	1.9	2,889
18 years & above	45.0	12,241	7.5	58.5	2.1	26.9	12.2	0.6	1.0	3.5	46.6	2.6	5,522
**Marital duration**													
0-4	44.8	4,265	7.0	58.7	2.0	30.7	10.9	1.2	0.4	3.7	44.3	1.5	1,912
5-9	44.2	3,584	8.6	57.7	2.0	23.3	11.7	0.4	0.6	3.8	47.7	2.3	1,592
10-14	42.0	3,482	7.2	52.2	1.6	22.1	12.1	0.3	1.4	3.4	51.9	2.5	1,466
15+	33.8	10,153	6.1	42.2	1.2	14.1	11.7	0.2	1.5	2.3	63.1	2.8	3,441
**Education**													
Non-literate[a]	24.8	8,928	3.7	25.5	0.1	0.4	9.2	0.0	0.9	2.1	78.0	1.8	2,210
Less than 5 yrs	31.6	925	4.8	38.9	0.0	3.3	9.2	0.0	1.0	2.4	67.7	2.8	294
5-9 years	41.3	6,384	7.5	52.8	0.9	13.7	11.8	0.2	0.8	3.3	54.1	2.8	2,629
10 or more years	62.2	5,247	8.9	67.2	3.3	42.7	13.3	1.0	1.3	3.7	36.1	2.3	3,278
**Husband's education**													
Non-literate[a]	25.1	3,883	4.7	26.1	0.2	2.8	9.2	0.0	1.2	1.7	78.0	2.8	973
Less than 5 years	30.9	765	7.9	33.8	0.4	4.3	12.3	0.0	0.4	1.7	70.8	2.6	238
5-9 years	34.0	6,896	5.2	45.6	0.8	9.9	11.3	0.1	1.0	2.1	59.2	2.5	2,343
10 or more years	48.7	9,940	8.2	58.9	2.4	31.0	12.2	0.8	1.0	4.0	45.6	2.1	4,857
**Religion**													
Hindu	39.8	18,724	6.9	52.0	1.6	22.0	11.6	0.4	1.1	3.1	52.7	2.3	7,448
Muslim	25.8	1,419	3.2	13.2	0.5	3.7	6.4	0.0	1.1	1.2	83.1	4.0	367
Sikh	44.2	1,315	9.8	59.7	2.0	19.6	15.1	1.4	0.5	4.2	50.4	1.8	581
Others	53.8	26	(0.0)	(66.7)	(0.0)	(33.3)	(26.7)	(0.0)	(6.7)	(0.0)	(40.0)	(6.7)	15

Contd…

TABLE 7.2 SOURCE OF KNOWLEDGE ABOUT RTI/STI BY BACKGROUND CHARACTERISTICS —*Continued*

Background characteristics	Who have heard RTI/STI	Total number of women**	Source of knowledge: Radio	T.V.	Cinema	Print media[1]	Health personnel[2]	School/adult education programs[3]	Leaders/ community meeting[4]	Husband	Relative/ friends	Other	Number of women heard of RTI/STI**
**Castes/Tribes**													
Scheduled Castes	33.5	4,924	6.0	42.1	0.9	10.7	12.6	0.4	1.2	3.7	61.5	2.8	1,652
Scheduled Tribes	37.9	87	9.4	42.2	0.0	15.3	21.4	0.0	3.1	11.8	51.0	3.1	34
Other Backward Classes	36.5	7,684	6.5	47.3	1.6	17.0	10.0	0.5	0.9	2.9	57.3	2.1	2,807
Others	44.5	8,786	7.7	56.7	2.0	28.3	12.2	0.5	1.0	3.0	48.3	2.4	3,917
**Wealth index**													
Lowest	27.2	258	4.2	10.1	0.0	3.9	5.5	0.0	0.0	0.0	90.3	1.3	69
Second	23.0	1,270	5.6	15.8	0.7	1.7	10.8	0.0	0.8	1.0	82.8	4.6	292
Middle	26.9	4,213	5.1	26.3	0.3	5.7	11.2	0.1	1.5	2.8	72.3	2.3	1,133
Fourth	35.1	6,730	6.9	43.4	0.7	12.0	12.1	0.5	1.0	3.2	59.7	2.3	2,354
Highest	50.4	9,012	7.6	63.5	2.5	31.1	11.5	0.6	1.0	3.3	43.8	2.3	4,563
**Haryana (15-49)**	39.0	21,484	6.9	50.6	1.6	21.0	11.6	0.5	1.0	3.1	53.9	2.3	8,411
**Haryana(15-44)** [†]	40.3	18,575	7.1	51.8	1.6	21.6	11.6	0.5	0.9	3.2	52.8	2.3	7,513

Note: Total figure may not add to 100 percent due to multiple responses.
( ) Based on 10-24 unweighted cases.
** Unweighted cases.
[a] Literates but did not attend school, are also included.
[1] Includes News papers/books/magazines/slogans/pamphlets and posters.
[2] Includes Doctor/ASHA/health workers.
[3] Includes school/teacher, adult education programs.
[4] Includes religious/ political leaders, community meetings and exhibition/ *Mela*.
[†] Represents figures for currently married women aged 15-44 years.

TABLE 7.3 KNOWLEDGE OF MODE OF TRANSMISSION OF RTI/STI BY BACKGROUND CHARACTERISTICS

Percentage of ever married women aged 15-49 years who have heard of RTI/STI and among them, who have knowledge of transmission of RTI/STI, according to selected background characteristics, Haryana, 2007-08

		knowledge of transmission of RTI/STI							
Background characteristics	Heard of RTI/STI	Unsafe delivery	Unsafe abortion	Unsafe IUD insertion	Unsafe sex with homosexuals	Unsafe sex with persons who have many partners	Unsafe sex with sex workers	Other	Number of women heard of RTI/STI **
**Age group**									
15-19	32.1	6.4	5.0	4.3	10.1	77.3	8.9	14.2	399
20-24	43.3	5.8	3.9	3.9	10.1	78.4	8.4	14.0	1,735
25-29	44.4	7.5	4.9	3.5	11.2	72.7	9.9	16.7	1,797
30-34	40.7	8.7	6.4	4.0	9.0	69.3	9.1	19.2	1,605
35-39	37.8	8.4	7.4	6.2	8.2	67.3	11.0	23.6	1,272
40-44	34.1	11.1	6.0	6.8	12.9	62.1	11.0	21.7	966
45-49	30.3	11.3	8.7	5.2	8.4	63.7	9.4	22.1	637
**Residence**									
Rural	36.4	8.2	4.7	3.7	10.3	70.5	10.0	17.7	5,859
Urban	46.9	8.0	7.8	6.3	9.6	71.4	9.0	20.1	2,552
**Age at consummation of marriage**									
Below 18 years	31.2	5.7	3.0	2.7	7.7	67.2	9.8	22.2	2,889
18 years & above	45.0	9.0	6.7	5.3	10.9	72.1	9.6	17.1	5,522
**Marital duration**									
0-4	44.8	6.2	4.7	4.2	12.3	77.5	9.0	14.4	1,912
5-9	44.2	8.3	5.9	4.7	9.2	74.6	9.3	14.1	1,592
10-14	42.0	8.0	5.5	3.7	8.4	70.9	9.7	19.9	1,466
15+	33.8	9.3	6.4	5.2	9.9	64.4	10.2	22.8	3,441
**Education**									
Non-literate[a]	24.8	7.2	3.7	2.4	6.9	59.8	11.1	25.3	2,210
Less than 5 yrs	31.6	8.2	8.7	1.8	4.4	64.7	10.0	21.1	294
5-9 years	41.3	7.9	2.6	2.9	10.8	69.4	9.9	19.0	2,629
10 or more years	62.2	8.7	8.3	6.6	11.0	75.9	9.0	15.6	3,278
**Husband's education**									
Non-literate[a]	25.1	8.4	2.0	2.0	6.3	65.7	9.9	24.3	973
Less than 5 years	30.9	3.4	3.6	1.1	6.1	71.4	11.7	23.0	238
5-9 years	34.0	8.7	3.6	3.1	10.6	65.8	9.0	20.5	2,343
10 or more years	48.7	8.1	7.1	5.6	10.5	73.3	9.8	16.8	4,857
**Religion**									
Hindu	39.8	7.4	5.7	4.3	10.0	71.6	9.4	18.1	7,448
Muslim	25.8	9.8	2.6	2.6	9.2	44.2	5.4	44.2	367
Sikh	44.2	16.1	7.8	8.1	11.0	68.4	14.5	15.1	581
Others	(57.7)	(11.1)	(0.0)	(0.0)	(22.2)	(66.7)	(0.0)	(33.3)	15
**Castes/Tribes**[b]									
Scheduled Castes	33.5	8.1	3.6	2.5	8.7	70.2	9.6	18.3	1,652
Scheduled Tribes	37.9	7.6	0.0	0.0	23.0	54.2	15.6	30.3	34
Other Backward Classes	36.5	7.7	5.2	4.4	9.8	68.5	8.9	21.3	2,807
Others	44.5	8.4	7.0	5.5	10.7	72.7	10.1	16.5	3,917
**Wealth index**									
Lowest	27.2	0.0	7.8	0.0	8.3	24.7	9.4	67.0	69
Second	23.0	5.9	0.0	1.5	10.6	67.5	7.6	23.6	292
Middle	26.9	8.9	2.5	1.1	8.4	66.0	7.5	20.7	1,133
Fourth	35.1	7.4	4.3	2.6	9.7	69.5	10.4	19.5	2,354
Highest	50.4	8.4	7.0	6.1	10.5	72.4	9.8	17.3	4,563
**Haryana (15-49)**	39.0	8.1	5.7	4.6	10.1	70.8	9.7	18.5	8,411
**Haryana(15-44)**[†]	40.3	7.6	5.6	4.5	10.3	71.5	9.7	18.2	7,513

Note: Total figure may not add to 100 percent due to multiple responses.
( ) Based on 10-24 unweighted cases.
** Unweighted cases.
[a] Literates but did not attend school, are also included.
[b] Total figure may not add to N, sample total, due to 'don't know' or 'missing cases'.
[†] Represents figures for currently married women aged 15-44 years.

TABLE 7.4 SYMPTOMS OF RTI/STI BY BACKGROUND CHARACTERISTICS

Percentage of ever married women aged 15-49 years who had reported abnormal vaginal discharge, other RTI/STI symptoms during three months prior to survey according to selected background characteristics, Haryana, 2007-08

Background characteristics	Women reported abnormal vaginal discharge	Women reported other RTI/STI symptoms[1]	Percentage reported specific symptom of RTI/STI[1]							Total number of women**
			Itching or irritation over vulva	Boils/Ulcers/ warts around vulva	Pain in lower abdomen not related menses	Swelling in the groin	Painful blister like lesions	Pain during sexual intercourse[2]	Spotting after sexual intercourse[2]	
**Age group**										
15-19	10.9	12.2	3.4	1.3	2.3	0.7	0.2	4.0	1.1	1,235
20-24	12.9	14.1	3.4	0.8	3.0	0.4	0.1	3.2	0.2	4,012
25-29	17.2	16.7	3.4	1.2	3.4	0.6	0.3	2.9	0.2	4,032
30-34	17.6	18.7	3.5	0.6	3.8	0.6	0.1	2.9	0.2	3,918
35-39	15.3	19.4	4.2	0.9	3.8	0.7	0.1	1.6	0.2	3,364
40-44	12.0	15.7	2.8	0.5	3.2	0.5	0.2	1.5	0.1	2,830
45-49	8.3	13.3	3.4	0.5	2.1	0.6	0.1	0.9	0.1	2,093
**Residence**										
Rural	15.0	17.3	3.7	0.9	3.5	0.6	0.2	2.6	0.2	16,154
Urban	11.9	13.2	2.8	0.5	2.5	0.3	0.1	1.9	0.2	5,330
**Age at consummation of marriage**										
Below 18 years	16.7	17.7	3.8	0.8	3.7	0.6	0.1	2.6	0.3	9,243
18 years & above	12.4	15.2	3.2	0.8	2.9	0.5	0.2	2.4	0.2	12,241
**Marital duration**										
0-4	9.6	12.2	3.1	0.9	2.5	0.4	0.2	3.3	0.4	4,265
5-9	17.0	15.9	3.3	0.8	3.2	0.5	0.1	3.0	0.2	3,584
10-14	18.0	19.0	4.1	1.0	4.1	0.6	0.3	3.0	0.2	3,482
15+	14.0	17.2	3.4	0.7	3.3	0.6	0.1	1.7	0.2	10,153
**Education**										
Non-literate[a]	15.3	17.4	3.7	0.8	3.6	0.7	0.1	2.3	0.2	8,928
Less than 5 yrs	16.6	19.1	4.3	1.0	3.8	0.7	0.2	3.3	0.5	925
5-9 years	15.4	17.7	3.9	0.9	3.6	0.5	0.2	2.7	0.4	6,384
10 or more years	10.6	12.3	2.4	0.7	2.1	0.5	0.1	2.4	0.1	5,247
**Husband's education**										
Non-literate[a]	14.8	17.1	4.0	0.7	3.8	0.6	0.2	2.3	0.3	3,883
Less than 5 years	16.5	19.2	4.6	1.2	4.4	0.5	0.1	2.2	0.1	765
5-9 years	15.9	18.4	4.0	1.1	3.6	0.6	0.1	3.0	0.3	6,896
10 or more years	12.7	14.3	2.8	0.7	2.7	0.5	0.1	2.2	0.2	9,940
**Religion**										
Hindu	13.7	15.6	3.2	0.7	2.9	0.5	0.1	2.4	0.2	18,724
Muslim	25.4	26.9	6.7	2.4	7.7	1.0	0.3	4.6	0.8	1,419
Sikh	8.5	13.4	3.4	0.9	2.9	0.3	0.3	1.1	0.2	1,315
Others	0.0	7.7	0.0	0.0	0.0	0.0	0.0	0.0	0.0	26

Contd…

TABLE 7.4 SYMPTOMS OF RTI/STI BY BACKGROUND CHARACTERISTICS — *Continued*

Background characteristics	Women reported abnormal vaginal discharge	Women reported other RTI/STI symptoms[1]	Percentage reported specific symptom of RTI/STI[1]							Total number of women **
			Itching or irritation over vulva	Boils/Ulcers/ Warts around vulva	Pain in lower abdomen not related menses	Swelling in the groin	Painful blister like lesions	Pain during sexual intercourse[2]	Spotting after sexual intercourse[2]	
**Castes/Tribes**										
Scheduled Castes	14.5	16.9	3.5	0.7	3.3	0.5	0.1	3.1	0.2	4,924
Scheduled Tribes	7.2	13.9	1.2	1.2	2.3	1.1	0.0	1.2	0.0	87
Other Backward Classes	16.1	17.8	4.2	1.1	3.8	0.6	0.2	2.6	0.3	7,684
Others	12.6	14.6	2.9	0.7	2.7	0.5	0.1	2.0	0.1	8,786
**Wealth index**										
Lowest	24.3	18.9	5.2	1.3	4.9	0.4	0.0	3.6	0.0	258
Second	17.9	17.9	4.1	0.8	5.4	0.6	0.2	2.8	0.3	1,270
Middle	17.0	18.5	4.0	1.0	4.0	0.6	0.1	2.6	0.3	4,213
Fourth	14.9	17.6	3.7	0.9	3.3	0.6	0.2	2.8	0.3	6,730
Highest	11.7	14.0	2.9	0.7	2.5	0.5	0.2	2.1	0.1	9,012
**Haryana (15-49)**	14.3	16.3	3.5	0.8	3.3	0.6	0.2	2.5	0.2	21,484
**Haryana(15-44)** †	15.0	16.7	3.5	0.9	3.4	0.6	0.2	2.6	0.2	18,575

Note: Total figure may not add to 100 percent due to 'do not know' or 'missing cases'.
** Unweighted cases.
[a] Literates but did not attend school, are also included.
[1] Excluding women having abnormal vaginal discharge problem.
[2] Only for currently married women.
† Represents figures for currently married women aged 15-44 years.

TABLE 7.5 DISCUSSED ABOUT RTI/STI PROBLEMS WITH HUSBAND AND SOUGHT TREATMENT BY BACKGROUND CHARACTERISTICS

Percentage of ever married women aged 15-49 years discussed RTI /STI problem with husband/partner and sought treatment among who reported any RTI/STI[1] problem and source of treatment according to selected background characteristics, Haryana, 2007-08.

Background characteristics	Women discussed RTI/STI problems with husband/ partner[1]	Women sought treatment[1] for RTI/STI problems	Number of women having any RTI/STI[1]**	Source of treatment			Number of women who sought treatment**
				Government	Private	Other	
**Age group**							
15-19	71.4	31.7	216	21.0	71.7	7.3	69
20-24	79.0	42.6	797	19.4	71.5	9.0	341
25-29	83.9	53.2	962	19.2	72.3	8.5	512
30-34	84.0	57.9	1,016	18.5	71.5	10.0	586
35-39	81.4	57.2	851	18.6	69.8	11.6	486
40-44	78.5	55.0	583	19.3	69.9	10.7	322
45-49	74.6	56.4	345	19.8	72.7	7.4	194
**Residence**							
Rural	79.8	50.9	3,762	19.7	71.0	9.3	1,919
Urban	84.6	58.9	1,008	17.1	71.9	10.9	591
**Age at consummation of marriage**							
Below 18 years	78.3	49.6	2,256	19.5	70.1	10.4	1,123
18 years & above	83.0	55.3	2,514	18.8	72.1	9.1	1,387
**Marital duration**							
0-4	77.5	39.7	708	20.8	71.7	7.4	282
5-9	83.5	51.9	844	18.6	73.0	8.4	439
10-14	83.5	55.5	913	19.3	71.9	8.8	507
15+	79.7	55.7	2,305	18.8	70.3	11.0	1,282
**Education**							
Non-literate[a]	77.3	48.9	2,068	20.2	68.9	10.9	1,011
Less than 5 yrs	81.0	51.8	240	30.6	61.4	8.0	125
5-9 years	82.0	53.9	1,547	18.5	71.7	9.8	834
10 or more years	86.6	59.0	915	15.1	77.4	7.5	540
**Husband's education**							
Non-literate[a]	76.9	46.8	863	23.4	66.0	10.6	404
Less than 5 years	82.2	50.9	192	21.5	65.8	12.7	97
5-9 years	79.5	50.0	1,718	20.3	69.8	9.9	860
10 or more years	83.4	57.6	1,997	16.4	74.7	8.9	1,149
**Religion**							
Hindu	81.1	52.7	4,042	20.3	70.1	9.7	2,128
Muslim	77.0	46.2	510	13.2	75.3	11.5	235
Sikh	85.6	67.8	216	12.7	80.5	6.8	146
Others	*	*	2	*	*	*	1
**Castes/Tribes**							
Scheduled Castes	79.9	50.9	1,125	25.2	62.9	11.9	572
Scheduled Tribes	(81.3)	(62.5)	16	(30.0)	(60.0)	(10.0)	10
Other Backward Classes	81.2	51.4	1,878	18.3	71.6	10.1	970
Others	80.8	54.8	1,751	16.1	76.0	8.0	958
**Wealth index**							
Lowest	83.1	47.4	75	8.6	62.9	28.6	35
Second	73.4	41.1	329	26.3	58.7	15.0	134
Middle	76.1	42.9	1,052	23.1	67.8	9.1	452
Fourth	81.2	52.5	1,584	21.9	67.5	10.5	831
Highest	84.6	61.2	1,730	14.5	77.7	7.9	1,058
**Haryana**	80.8	52.6	4,770	19.1	71.2	9.7	2,510

Note: Total figure may not add to 100 percent due to ‘do not know’ or ‘missing cases’.
( ) Based on 10-24 unweighted cases
* Percentage not shown; based on less than 10 unweighted cases.
** Unweighted cases.
[a] Literates but did not attend school, are also included.
[1] Any RTI/STI (Including abnormal vaginal discharge or other RTI/STI problem).

TABLE 7.6 RTI/STI INDICATORS BY DISTRICTS

Percentage of ever married women aged 15-49 years who reported RTI/STI problem during three months prior to the survey and among them percentage sought treatment for the problem, by district, Haryana , 2007-08

Districts	Who heard about RTI/STI	Who reported any abnormal vaginal discharge	Who have any other symptoms of RTI/STI[1]	Total number of women**	Who sought treatment for any RTI/STI[2]	Number of women having any RTI/STI[2]
Panchkula	58.8	12.9	20.3	951	51.3	212
Ambala	40.8	10.1	21.3	980	55.3	274
Yamunanagar	50.5	13.0	25.6	1,067	69.1	337
Kurukshetra	54.1	9.7	20.0	1,002	55.8	237
Kaithal	31.7	10.4	11.2	1,097	56.9	175
Karnal	53.1	16.1	21.0	1,051	53.7	244
Panipat	47.4	16.5	22.9	1,026	52.5	276
Sonipat	42.8	13.9	19.3	1,048	51.0	222
Jind	42.7	14.5	17.6	1,070	41.3	283
Fatehabad	27.3	10.8	9.3	1,073	49.1	155
Sirsa	35.5	10.1	9.9	1,096	57.7	150
Hisar	41.3	9.9	11.7	1,010	45.1	161
Bhiwani	36.8	14.9	15.0	1,112	42.4	217
Rohtak	31.3	15.7	11.1	1,056	44.4	203
Jhajjar	42.2	12.2	11.3	1,022	54.6	169
Mahendragarh	34.2	22.2	18.2	1,117	48.5	327
Rewari	46.5	14.2	11.8	1,117	56.3	213
Gurgaon	28.9	15.6	14.1	1,265	52.6	265
Faridabad	30.7	9.4	8.6	994	53.1	158
Mewat	27.0	27.0	24.3	1,331	43.8	492
**Haryana (15-49)**	39.0	14.3	16.3	21,484	52.6	4,770
**Haryana(15-44)** †	40.3	15.0	16.7	18,575	52.5	4,661

** Unweighted cases.
[1] Excluding women having abnormal vaginal discharge.
[2] Any RTI/STI (Including abnormal vaginal discharge problem or other RTI/STI problem).
† Represents figures for currently married women aged 15-44 years.

TABLE 7.7 KNOWLEDGE OF HIV/AIDS BY BACKGROUND CHARACTERISTICS

Percentage of ever married women aged 15–49 years who have heard of HIV/AIDS and among them, who received information from specific sources according to selected background characteristics, Haryana, 2007-08

			Sources of knowledge for HIV/AIDS										
Background characteristics	Who have heard of HIV/AIDS	Total women**	Radio	T.V.	Cinema	Print media[1]	Health personnel[2]	School/ adult education programs[3]	Leaders/ community meetings[4]	Husband	Relatives/ friends	Other	Number of women heard of HIV/AIDS**
**Age group**													
15-19	60.3	1,235	16.7	79.3	2.2	28.2	8.3	5.4	0.7	5.7	35.7	1.8	757
20-24	74.3	4,012	15.8	84.4	2.7	35.0	11.5	1.8	0.9	6.8	32.9	2.1	2,989
25-29	74.1	4,032	16.3	85.1	3.0	34.1	11.2	1.2	1.3	6.4	33.1	2.7	3,001
30-34	67.0	3,918	14.6	80.9	2.6	29.7	10.7	0.6	1.6	5.0	37.6	3.6	2,634
35-39	59.2	3,364	14.1	77.0	1.7	24.5	12.7	0.4	1.7	4.7	43.2	2.7	1,997
40-44	53.0	2,830	11.6	78.7	1.8	22.7	10.8	0.3	1.6	3.4	42.8	2.3	1,501
45-49	48.3	2,093	12.4	78.9	2.3	23.3	9.7	0.5	2.3	3.1	40.0	3.2	1,016
**Residence**													
Rural	59.8	16,154	16.3	77.7	2.1	24.7	11.4	1.2	1.6	5.7	40.9	2.8	9,700
Urban	78.6	5,330	11.3	90.3	3.3	41.5	10.3	1.2	1.0	4.6	28.2	2.3	4,195
**Age at consummation of marriage**													
Below 18 years	49.1	9,243	14.8	72.1	1.4	17.7	9.9	0.8	1.3	5.1	45.8	2.3	4,576
18 years & above	76.1	12,241	14.7	86.1	3.0	35.7	11.6	1.4	1.4	5.5	32.8	2.9	9,319
**Marital duration**													
0-4	76.5	4,265	15.9	86.9	3.2	39.7	10.6	3.2	0.9	6.5	30.2	1.9	3,278
5-9	75.6	3,584	16.0	84.3	3.0	33.1	11.9	1.0	1.2	6.3	34.2	3.0	2,720
10-14	71.0	3,482	15.5	82.0	2.5	30.8	11.1	0.4	1.4	5.6	35.2	3.3	2,487
15+	53.1	10,153	13.1	76.6	1.7	21.5	11.0	0.4	1.8	4.1	43.5	2.8	5,410
**Education**													
Non-literate[a]	35.6	8,928	12.8	66.5	0.2	1.3	9.1	0.2	1.2	5.0	51.6	2.2	3,211
Less than 5 yrs	60.7	925	12.5	73.5	0.5	4.6	9.3	0.4	0.9	5.4	48.2	2.2	563
5-9 years	78.8	6,384	14.3	82.5	1.8	22.7	10.2	0.5	1.3	5.2	35.9	2.8	5,020
10 or more years	97.2	5,247	16.8	91.0	4.8	57.5	13.4	2.6	1.7	5.8	27.8	2.9	5,101
**Husband's education**													
Non-literate[a]	34.4	3,883	14.9	67.5	0.5	5.8	10.6	0.3	1.5	2.8	48.2	2.3	1,346
Less than 5 years	47.9	765	20.4	73.3	1.6	8.6	11.1	0.8	0.5	4.7	43.0	3.6	369
5-9 years	59.6	6,896	13.2	77.7	1.6	17.0	10.2	0.5	1.1	4.6	39.8	2.5	4,124
10 or more years	81.0	9,940	15.3	86.2	3.3	41.3	11.6	1.7	1.6	6.2	33.5	2.8	8,056
**Religion**													
Hindu	67.3	18,724	14.7	81.6	2.4	30.2	10.9	1.2	1.4	5.4	36.9	2.7	12,574
Muslim	21.8	1,419	13.4	58.5	0.9	12.8	11.6	0.6	0.3	7.1	46.8	4.3	326
Sikh	74.1	1,315	17.1	88.7	4.2	29.0	13.2	1.6	1.7	4.8	35.6	2.6	972
Others	84.6	26	(9.1)	(90.9)	(9.1)	(63.6)	(9.1)	(0.0)	(0.0)	(0.0)	(18.2)	(0.0)	23

Contd….

TABLE 7.7 KNOWLEDGE OF HIV/AIDS BY BACKGROUND CHARACTERISTICS — *Continued*

Background characteristics	Who have heard of HIV/AIDS AIDS	Total women**	Sources of knowledge for HIV/AIDS										Number of women heard of HIV/AIDS**
			Radio	T.V.	Cinema	Print media[1]	Health personnel[2]	School/ adult education programs[3]	Leaders/ community meetings[4]	Husband	Relatives/ friends	Other	
**Castes/Tribes**													
Scheduled Castes	55.3	4,924	14.6	77.4	1.4	19.0	12.2	1.1	1.3	4.9	38.9	2.9	2,711
Scheduled Tribes	58.1	87	8.2	80.3	1.9	31.4	8.0	3.8	3.9	7.9	36.8	4.0	51
Other Backward Classes	58.8	7,684	15.0	81.5	2.4	27.4	10.5	1.2	1.4	6.3	37.1	2.5	4,568
Others	74.7	8,786	14.8	83.2	3.0	35.8	11.0	1.2	1.4	4.9	36.2	2.7	6,563
**Wealth index**													
Lowest	13.9	258	18.9	43.0	2.6	5.1	19.3	0.0	8.0	2.2	46.9	10.9	37
Second	25.7	1,270	17.5	48.5	0.6	7.9	15.0	0.6	1.6	5.4	54.2	3.4	332
Middle	41.3	4,213	16.3	61.6	0.7	12.2	11.4	0.8	1.8	4.7	49.5	2.9	1,756
Fourth	61.8	6,730	14.5	76.7	1.6	19.3	11.4	1.0	1.2	5.3	41.2	2.4	4,155
Highest	84.5	9,012	14.4	90.4	3.4	40.6	10.6	1.4	1.4	5.6	31.1	2.7	7,615
**Haryana(15-49)**	64.4	21,484	14.8	81.5	2.5	29.8	11.1	1.2	1.4	5.4	37.1	2.7	13,895
**Haryana(15-44)** [†]	66.6	18,575	15.0	81.9	2.5	30.5	11.2	1.2	1.3	5.7	36.6	2.6	12,423

Note: Total figure may not add to 100 percent due to multiple responses
** Unweighted cases.
( ) Based on 10-24 unweighted cases
[a] Literates but did not attend school, are also included.
[1] Includes News papers/books/magazines//slogans/pamphlets and posters.
[2] Includes Doctor/ASHA/health workers.
[3] Includes school/teacher, adult education programs.
[4] Includes religious/ political leaders, community meetings and exhibition/ *Mela*.
[†] Represents figures for currently married women aged 15-44 years.

TABLE 7.8 KNOWLEDGE ABOUT MODE OF TRANSMISSION OF HIV/AIDS BY BACKGROUND CHARACTERISTICS

Percentage of ever married women aged 15-49 years having knowledge of mode of transmission of HIV/AIDS among who have heard about HIV/AIDS according to selected background characteristics, Haryana, 2007-08.

Background characteristics	Percentage of women who reported mode of transmission as: Unsafe sex with homosexuals	Unsafe sex with person having many partners	Unsafe sex with sex workers	Unprotected sex with HIV/AIDS infected person	Infected mother to child	Transfusion of infected blood	Other	Number of women who heard of HIV/AIDS**
**Age group**								
15-19	1.0	82.2	8.9	25.2	12.1	37.3	36.8	757
20-24	3.1	80.8	10.5	25.6	14.0	43.8	39.2	2,989
25-29	3.0	81.6	11.0	26.6	15.4	44.4	39.0	3,001
30-34	3.9	80.9	10.0	26.2	13.3	40.9	36.8	2,634
35-39	3.2	80.0	12.7	26.1	12.6	39.5	36.4	1,997
40-44	4.2	79.8	12.2	24.2	11.6	36.7	32.1	1,501
45-49	3.3	78.2	12.9	24.0	11.1	36.0	33.3	1,016
**Residence**								
Rural	3.2	80.5	11.2	25.7	11.5	38.7	35.6	9,700
Urban	3.4	81.0	10.9	25.9	17.9	46.7	40.3	4,195
**Age at consummation of marriage**								
Below 18 years	1.3	81.7	10.2	24.2	8.6	31.9	31.8	4,576
18 years & above	4.1	80.2	11.5	26.5	15.6	45.4	39.4	9,319
**Marital duration**								
0-4	3.4	80.4	10.7	27.3	16.5	47.2	42.7	3,278
5-9	2.9	82.0	10.5	25.0	14.7	43.4	37.6	2,720
10-14	3.5	81.5	10.6	26.1	13.2	41.8	37.2	2,487
15+	3.3	79.8	11.9	25.0	10.9	35.7	32.9	5,410
**Education**								
Non-literate[a]	2.1	78.7	13.5	22.2	5.1	21.9	22.2	3,211
Less than 5 yrs	1.9	81.1	11.6	22.4	4.9	28.7	27.1	563
5-9 years	3.4	80.1	10.5	24.5	9.1	35.4	33.1	5,020
10 or more years	3.9	82.1	10.4	28.9	22.0	56.6	48.5	5,101
**Husband's education**								
Non-literate[a]	1.6	78.6	16.0	24.6	6.2	24.5	23.6	1,346
Less than 5 years	3.1	75.2	13.7	22.0	4.9	28.5	23.2	369
5-9 years	3.5	79.8	11.1	24.4	8.8	31.7	29.8	4,124
10 or more years	3.4	81.6	10.4	26.7	16.9	48.2	42.6	8,056
**Religion**								
Hindu	3.0	81.1	10.9	25.5	13.3	41.0	37.2	12,574
Muslim	3.5	82.8	10.2	20.7	8.2	28.0	28.6	326
Sikh	6.2	75.0	13.7	31.2	17.1	47.5	37.7	972
Others	(0.0)	(72.7)	(18.2)	(27.3)	(18.2)	(63.6)	(45.5)	23
**Castes/Tribes**								
Scheduled Castes	3.0	79.0	12.7	25.4	9.6	33.6	31.4	2,711
Scheduled Tribes	10.3	79.8	18.0	20.3	13.1	39.5	26.4	51
Other Backward Classes	3.9	81.3	10.9	26.8	12.2	39.3	35.4	4,568
Others	2.9	80.9	10.6	25.2	15.8	45.4	40.4	6,563
**Wealth index**								
Lowest	9.5	89.7	0.0	30.1	4.6	13.6	13.5	37
Second	1.3	84.3	16.8	18.3	6.9	21.5	21.5	332
Middle	3.1	78.7	12.8	22.8	7.0	26.8	25.7	1,756
Fourth	2.5	80.7	11.4	24.1	9.1	34.5	32.3	4,155
Highest	3.7	80.9	10.5	27.4	17.1	48.1	42.2	7,615
**Haryana**	3.3	80.7	11.1	25.8	13.5	41.2	37.1	13,895

Note: Total figure may not add to 100 percent due to multiple responses.
( ) Based on 10-24 unweighted cases;
** Unweighted cases.
[a] Literates but did not attend school, are also included.

TABLE 7.9 KNOWLEDGE OF HIV/AIDS PREVENTION METHODS BY BACKGROUND CHARACTERISTICS

Among ever married women aged 15-49 years who heard about HIV/AIDS, percentage who reported HIV/AIDS can be prevented in specific ways, according to selected background characteristics, Haryana, 2007-08

	Percentage who say that HIV/AIDS can be prevented by					
Background characteristics	Abstain from sex	Using condom correctly during each sexual intercourse	Sex with one partner/ avoid homosexual[1]	Avoid risk of getting infected through blood[2]	Avoid pregnancy when having HIV/AIDS	Number of women having knowledge of HIV/AIDS**
**Age group**						
15-19	6.1	28.9	65.0	38.6	5.2	757
20-24	7.1	37.4	72.5	47.4	5.9	2,989
25-29	7.0	37.9	76.0	50.2	6.3	3,001
30-34	7.5	35.3	72.8	44.8	5.3	2,634
35-39	8.8	30.6	69.9	41.5	4.4	1,997
40-44	9.0	28.0	67.1	37.5	4.6	1,501
45-49	8.3	27.9	64.6	36.7	3.6	1,016
**Residence**						
Rural	7.6	31.4	70.1	41.4	4.7	9,700
Urban	7.6	40.1	74.3	51.2	6.7	4,195
**Age at consummation of marriage**						
Below 18 years	6.1	22.8	67.6	33.2	3.6	4,576
18 years & above	8.3	39.1	73.3	49.8	6.1	9,319
**Marital duration**						
0-4	7.4	40.2	72.7	52.2	7.4	3,278
5-9	6.8	38.0	74.8	48.0	5.7	2,720
10-14	7.5	36.1	74.3	46.0	5.0	2,487
15+	8.3	26.9	67.5	36.9	4.0	5,410
**Education**						
Non-literate[a]	6.5	15.4	59.3	21.7	1.8	3,211
Less than 5 yrs	7.0	16.0	69.5	29.0	2.3	563
5-9 years	7.9	29.7	69.5	38.9	3.1	5,020
10 or more years	7.9	47.9	81.1	65.9	9.1	5,101
**Husband's education**						
Non-literate[a]	7.3	17.9	59.7	23.4	2.5	1,346
Less than 5 years	6.2	20.2	59.4	23.3	1.2	369
5-9 years	8.2	26.9	66.3	33.7	3.4	4,124
10 or more years	7.4	39.9	76.5	54.3	6.7	8,056
**Religion**						
Hindu	7.5	34.0	71.6	44.3	5.3	12,574
Muslim	6.7	22.7	61.0	29.0	2.3	326
Sikh	9.9	39.0	71.9	50.4	6.6	972
Others	(18.2)	(54.5)	(81.8)	(81.8)	(0.0)	23
**Castes/Tribes**						
Scheduled Castes	6.2	28.6	66.2	34.3	4.0	2,711
Scheduled Tribes	22.4	44.3	59.0	37.3	11.2	51
Other Backward Classes	8.1	33.9	71.1	42.8	4.5	4,568
Others	7.7	36.3	73.8	49.7	6.4	6,563
**Wealth index**						
Lowest	0.0	14.8	51.6	13.4	0.0	37
Second	7.0	17.3	59.1	21.4	2.9	332
Middle	6.7	19.4	61.5	26.7	2.8	1,756
Fourth	7.3	27.1	68.8	36.7	3.1	4,155
Highest	8.0	40.9	75.7	53.7	7.0	7,615
**Haryana**	7.6	34.2	71.4	44.4	5.3	13,895

Note: Total figure may not add to 100 percent due to multiple responses.
** Unweighted cases.
( ) Based on 10-24 unweighted cases
[a] Literates but did not attend school, are also included.
[1] Includes sex with one partner, Limit number of sexual partners, Avoid sex with sex workers and avoids sex with homosexuals.
[2] Includes avoid sex with who inject drugs, use tested blood, use only new/ sterilized needles, avoid IV drip and avoid razors/blades.

TABLE 7.10 MISCONCEPTION ABOUT TRANSMISSION OF HIV/AIDS BY BACKGROUND CHARACTERISTICS

Percentage of ever married women aged 15-49 years having misconception about the transmission of HIV/AIDS among who have heard of HIV/AIDS, according to selected background characteristics, Haryana,2007-08

	Percentage who have misconception about the transmission of HIV/AIDS by						
Background characteristics	Shaking hand	Hugging	Sharing clothes	Sharing food	Stepping on someone's urine/stool	Get HIV/AIDS from mosquito, flea or bedbug	Number of women heard of HIV/AIDS**
**Age group**							
15-19	11.8	12.3	13.9	16.4	12.7	20.2	757
20-24	7.8	7.9	9.2	9.6	7.7	15.6	2,989
25-29	6.9	7.0	8.8	9.7	8.1	15.9	3,001
30-34	8.7	9.2	11.0	12.6	10.6	16.8	2,634
35-39	10.2	10.0	13.0	14.5	12.0	18.5	1,997
40-44	10.3	10.1	14.0	14.4	12.3	19.1	1,501
45-49	11.8	12.1	16.6	16.8	12.1	20.5	1,016
**Residence**							
Rural	9.6	9.8	12.2	13.3	10.9	18.3	9,700
Urban	7.2	7.3	9.1	9.9	8.0	14.9	4,195
**Age at consummation of marriage**							
Below 18 years	11.4	11.8	14.3	15.5	12.8	18.9	4,576
18 years & above	7.7	7.7	9.8	10.7	8.7	16.5	9,319
**Marital duration**							
0-4	8.3	8.6	9.7	10.6	8.3	16.6	3,278
5-9	6.1	6.3	7.7	8.5	7.1	14.6	2,720
10-14	8.2	8.2	10.3	11.8	9.7	17.1	2,487
15+	11.0	11.1	14.5	15.5	12.8	19.2	5,410
**Education**							
Non-literate[a]	14.0	14.5	18.5	19.5	16.1	23.9	3,211
Less than 5 yrs	12.0	12.3	14.6	15.6	12.4	20.5	563
5-9 years	9.0	8.9	11.5	12.8	10.6	17.9	5,020
10 or more years	5.2	5.4	6.2	6.9	5.4	12.2	5,101
**Husband's education**							
Non-literate[a]	14.7	14.4	17.5	18.5	16.0	23.1	1,346
Less than 5 years	10.7	11.3	16.1	16.2	14.4	21.6	369
5-9 years	10.6	11.2	13.8	14.8	12.6	20.0	4,124
10 or more years	6.9	7.0	8.7	9.8	7.5	14.7	8,056
**Religion**							
Hindu	8.7	9.0	11.1	12.2	9.8	16.8	12,574
Muslim	14.2	13.2	16.2	17.0	17.2	25.5	326
Sikh	9.1	8.3	11.6	12.8	10.6	20.3	972
Others	(0.0)	(0.0)	(0.0)	(0.0)	(0.0)	(9.1)	23
**Castes/Tribes**							
Scheduled Castes	10.1	10.6	12.8	14.1	11.6	20.0	2,711
Scheduled Tribes	13.4	13.2	15.1	19.0	17.2	23.4	51
Other Backward Classes	8.9	8.7	11.0	12.0	10.2	17.7	4,568
Others	8.4	8.6	10.8	11.7	9.3	15.9	6,563
**Wealth index**							
Lowest	10.5	13.1	15.6	15.6	13.1	7.8	37
Second	14.8	15.1	19.2	22.3	17.8	27.6	332
Middle	14.5	14.9	16.9	18.7	15.2	22.4	1,756
Fourth	10.0	9.9	12.5	13.6	11.3	18.5	4,155
Highest	6.7	6.9	9.0	9.7	7.8	15.1	7,615
**Haryana**	8.9	9.0	11.3	12.3	10.0	17.3	13,895

** Unweighted cases.
( ) Based on 10-24 unweighted cases
[a] Literates but did not attend school, are also included.

TABLE 7.11 KNOWLEDGE ABOUT THE PLACE WHERE HIV/AIDS TEST CAN BE DONE BY BACKGROUND CHARACTERISTICS

Percentage of ever married women aged 15-49 years having knowledge about place where HIV/AIDS test can be done according to selected background characteristics, Haryana, 2007-08

Background characteristics	Who know the place of HIV/AIDS test	Total women heard of HIV/AIDS**	Places where people can go to test for HIV /AIDS: Government: Hospital/ dispensary	Government: CHC/PHC/ Sub-Centre	Government: VCTC/ICTC/ RTI/STI Clinic	Government: Other public/NGO hospItal	Private: Hospital/ Clinic	Private: VCTC/ICTC/ RTI/STI Clinic	Private: Other Private centre	Number of women who know the place for HIV/AIDS test**
**Age group**										
15-19	65.5	757	54.3	9.3	0.1	0.2	36.0	0.2	0.0	497
20-24	71.9	2,989	53.7	9.5	0.2	0.6	35.3	0.4	0.3	2,145
25-29	72.8	3,001	51.5	12.2	0.2	0.5	35.0	0.5	0.1	2,186
30-34	74.1	2,634	54.7	11.2	0.2	0.3	33.0	0.2	0.4	1,955
35-39	68.6	1,997	54.6	10.4	0.1	0.8	33.6	0.3	0.3	1,373
40-44	68.8	1,501	57.2	10.4	0.3	0.6	31.0	0.3	0.2	1,035
45-49	66.4	1,016	59.1	9.0	0.5	0.3	30.1	0.4	0.6	678
**Residence**										
Rural	69.0	9,700	53.6	12.3	0.1	0.4	33.1	0.2	0.2	6,671
Urban	75.5	4,195	55.6	7.0	0.4	0.7	35.2	0.7	0.4	3,198
**Age at consummation of marriage**										
Below 18 years	64.1	4,576	55.1	11.4	0.1	0.3	32.9	0.1	0.3	2,931
18 years & above	74.4	9,319	54.0	10.3	0.3	0.6	34.1	0.5	0.3	6,938
**Marital duration**										
0-4	74.1	3,278	52.2	9.2	0.2	0.5	36.9	0.5	0.3	2,427
5-9	71.6	2,720	52.5	10.9	0.2	0.5	35.3	0.5	0.1	1,948
10-14	74.2	2,487	53.9	12.1	0.3	0.4	32.8	0.2	0.4	1,846
15+	67.3	5,410	56.8	10.6	0.2	0.6	31.3	0.2	0.3	3,648
**Education**										
Non-literate[a]	57.2	3,211	55.1	11.7	0.1	0.4	32.4	0.1	0.2	1,833
Less than 5 yrs	58.7	563	56.0	14.5	0.0	0.6	28.5	0.0	0.3	328
5-9 years	69.2	5,020	56.5	12.7	0.2	0.3	30.0	0.1	0.1	3,475
10 or more years	82.8	5,101	52.0	8.1	0.3	0.7	37.9	0.7	0.4	4,233
**Husband's education**										
Non-literate[a]	56.4	1,346	59.7	13.8	0.1	0.5	25.8	0.0	0.1	757
Less than 5 years	63.0	369	57.8	15.1	0.0	0.8	26.3	0.0	0.0	233
5-9 years	65.8	4,124	56.6	12.1	0.1	0.4	30.5	0.2	0.2	2,710
10 or more years	76.5	8,056	52.5	9.4	0.3	0.5	36.5	0.5	0.3	6,169
**Religion**										
Hindu	71.0	12,574	54.7	10.9	0.2	0.5	33.2	0.3	0.3	8,931
Muslim	65.3	326	47.5	9.5	0.0	0.0	42.5	0.5	0.0	211
Sikh	72.8	972	51.4	8.1	0.1	0.9	38.6	0.7	0.1	708
Others	(90.9)	23	(60.0)	(0.0)	(0.0)	(0.0)	(40.0)	(0.0)	(0.0)	19

Contd…

TABLE 7.11 KNOWLEDGE ABOUT THE PLACE WHERE HIV/AIDS TEST CAN BE DONE BY BACKGROUND CHARACTERISTICS — ***Continued***

Background characteristics	Who know the place of HIV/AIDS test	Total women heard of HIV/AIDS**	Places where people can go to test for HIV /AIDS: Government: Hospital/ dispensary	Government: CHC/PHC/ Sub-Centre	Government: VCTC/ICTC/ RTI/STI Clinic	Government: Other public/NGO hospItal	Private: Hospital/ Clinic	Private: VCTC/ICTC/ RTI/STI Clinic	Private: Other Private centre	Number of women who know the place for HIV/AIDS test**
**Castes/Tribes**										
Scheduled Castes	64.5	2,711	57.6	13.1	0.1	0.7	28.2	0.1	0.2	1,751
Scheduled Tribes	60.9	51	52.8	9.9	0.0	0.0	37.3	0.0	0.0	31
Other Backward Classes	72.0	4,568	53.1	11.8	0.3	0.4	33.9	0.2	0.3	3,288
Others	73.0	6,563	53.9	8.9	0.2	0.5	35.7	0.5	0.2	4,797
**Wealth index**										
Lowest	53.6	37	(55.0)	(10.0)	(0.0)	(0.0)	(35.0)	(0.0)	(0.0)	20
Second	58.7	332	52.3	20.0	0.5	0.0	27.2	0.0	0.0	194
Middle	62.5	1,756	57.3	14.9	0.0	0.6	27.0	0.0	0.1	1,093
Fourth	66.4	4,155	56.3	11.8	0.0	0.3	31.1	0.2	0.3	2,759
Highest	76.0	7,615	52.8	8.9	0.3	0.6	36.5	0.5	0.3	5,803
**Haryana (15-49)**	71.0	13,895	54.3	10.6	0.2	0.5	33.8	0.3	0.3	9,869
**Haryana(15-44)**[†]	71.6	12,423	53.7	10.6	0.2	0.5	34.4	0.4	0.2	8,898

Note: Total figure may not add to 100 percent due to ‘do not know’ or ‘missing cases’.
CHC= Community Health Centre; PHC= Primary Health Centre; VCTC/ICTC= voluntary/Integrated counseling and testing centre, NGO= Non Governmental Organization.
( ) Based on 10-24 unweighted cases
** Unweighted cases.
[a] Literates but did not attend school, are also included.
[†] Represents figures for currently married women aged 15-44 years.

TABLE 7.12 UNDERGONE HIV/AIDS TEST BY BACKGROUND CHARACTERISTICS

Percentage of ever married women aged 15-49 years undergone HIV/AIDS test and time of the tested, according to selected background characteristics, Haryana, 2007-08

Background characteristics	Who have been tested for HIV	Number of women heard HIV/AIDS**	Percentage who have been tested for HIV: Less than 12 months ago	Percentage who have been tested for HIV: 1 or more than 1 year ago	Number of women went for HIV/AIDS test**
**Age group**					
15-19	3.7	757	81.7	18.3	28
20-24	7.9	2,989	55.6	44.4	233
25-29	7.1	3,001	40.5	59.5	211
30-34	4.7	2,634	27.9	72.1	126
35-39	3.1	1,997	23.3	76.7	62
40-44	1.8	1,501	32.8	67.2	27
45-49	1.4	1,016	(46.7)	(53.3)	15
**Residence**					
Rural	4.2	9,700	43.7	56.3	398
Urban	7.1	4,195	42.7	57.3	304
**Age at consummation of marriage**					
Below 18 years	2.6	4,576	43.6	56.4	121
18 years & above	6.2	9,319	43.2	56.8	581
**Marital duration**					
0-4	9.4	3,278	58.3	41.7	310
5-9	6.8	2,720	34.4	65.6	182
10-14	4.0	2,487	24.5	75.5	100
15+	2.0	5,410	32.6	67.4	110
**Education**					
Non-literate[a]	1.7	3,211	44.5	55.5	54
Less than 5 yrs	3.1	563	(35.3)	(64.7)	17
5-9 years	3.1	5,020	44.5	55.5	151
10 or more years	9.4	5,101	43.1	56.9	480
**Husband's education**					
Non-literate[a]	2.4	1,346	40.4	59.6	31
Less than 5 years	2.2	369	*	*	8
5-9 years	2.9	4,124	49.5	50.5	118
10 or more years	6.8	8,056	42.1	57.9	545
**Religion**					
Hindu	4.9	12,574	42.1	57.9	620
Muslim	4.4	326	(53.8)	(46.2)	13
Sikh	6.8	972	52.5	47.5	66
Others	(0.0)	23	*	*	3
**Castes/Tribes**					
Scheduled Castes	4.1	2,711	49.3	50.7	113
Scheduled Tribes	12.0	51	*	*	6
Other Backward Classes	4.2	4,568	42.6	57.4	190
Others	6.0	6,563	41.5	58.5	393
**Wealth index**					
Lowest	0.0	37	nc	nc	0
Second	1.0	332	*	*	3
Middle	2.3	1,756	36.3	63.7	39
Fourth	3.2	4,155	42.2	57.8	131
Highest	6.9	7,615	43.7	56.3	529
**Haryana (15-49)**	5.1	13,895	43.3	56.7	702
**Haryana(15-44)** †	5.4	12,423	43.3	56.7	675

nc= Not calculated because there are no cases.
( ) Based on 10-24 unweighted cases.
* Percentage not shown; based on less than 10 unweighted cases.
** Unweighted cases.
[a] Literates but did not attend school, are also included.
† Represents figures for currently married women aged 15-44 years.

TABLE 7.13 HIV/AIDS INDICATORS BY DISTRICTS

Percentage of ever married women aged 15-49 years who have heard of HIV/AIDS, know HIV/AIDS prevention, transmission, places where people can go to test for HIV /AIDS and who have been tested for HIV/AIDS in the past 12 months, by districts, Haryana, 2007-08

Districts	Who have heard of HIV/AIDS	Who know that HIV/AIDS can be prevented by using condom	Who know that HIV/AIDS can be transmitted from mother to her baby	Who know the places where people can go to get tested for HIV /AIDS	Who ever been tested for HIV/AIDS (%)	Who underwent HIV/AIDS test in the past 12 months among ever tested
Panchkula	86.0	42.9	12.4	83.8	16.3	33.5
Ambala	79.9	57.4	17.1	70.5	7.1	38.0
Yamunanagar	76.5	50.5	9.7	76.2	7.3	40.7
Kurukshetra	83.9	24.9	15.1	71.2	6.3	44.6
Kaithal	65.1	40.5	28.3	78.2	6.4	29.4
Karnal	82.5	29.2	6.7	74.5	3.3	26.4
Panipat	77.4	27.9	5.9	83.1	5.3	43.3
Sonipat	81.4	29.8	13.4	87.6	4.7	48.2
Jind	61.7	27.9	11.4	73.2	2.4	30.8
Fatehabad	45.4	34.5	11.8	42.7	3.0	24.6
Sirsa	57.5	39.8	22.0	73.0	6.5	48.1
Hisar	57.7	33.9	16.3	51.0	2.1	43.0
Bhiwani	60.8	27.0	19.0	72.9	1.3	38.5
Rohtak	71.0	34.8	14.0	68.8	6.4	61.5
Jhajjar	71.1	28.9	11.5	56.5	6.8	57.0
Mahendragarh	55.2	31.7	13.5	74.5	3.6	21.2
Rewari	69.2	42.0	17.6	75.5	3.6	31.1
Gurgaon	67.0	33.5	16.5	64.2	7.1	43.4
Faridabad	56.2	16.3	9.3	54.1	1.8	61.1
Mewat	18.9	23.5	8.3	67.5	3.6	64.3
**Haryana (15-49)**	64.4	34.2	13.5	71.0	5.1	43.3
**Haryana(15-44)** †	66.6	34.9	13.7	71.6	5.4	43.3

† Represents figures for currently married women aged 15-44 years.

TABLE 7.14 EVER HAD INFERTILITY PROBLEM BY BACKGROUND CHARACTERISTICS

Percentage of ever married women aged 15-49 years who ever had infertility problem according to selected background characteristics, Haryana, 2007-08

Background Characteristics	Who ever had infertility problem	Primary infertility	Secondary infertility	Number of women**[1]	Percentage of women who have ever had infertility problem: In the first con-ception	After live birth/still birth	After induced/ spontaneous abortion	Other incidents[2]	Number of women having primary/ secondary infertility**
**Age group**									
15-19	4.9	3.8	1.0	1,168	79.0	2.0	18.9	0.0	57
20-24	11.0	8.4	2.6	4,006	76.0	9.1	13.2	1.4	445
25-29	12.2	8.9	3.4	4,032	72.6	14.4	12.0	1.0	495
30-34	11.1	8.0	3.1	3,918	72.4	16.0	10.3	0.7	434
35-39	11.4	9.0	2.4	3,364	79.0	13.4	6.1	0.5	379
40-44	11.3	9.1	2.2	2,830	80.7	10.1	7.3	1.3	318
45-49	12.2	10.3	1.9	2,093	84.3	8.8	6.1	0.0	253
**Residence**									
Rural	10.8	8.4	2.4	16,086	77.7	10.9	9.9	0.8	1,733
Urban	12.3	9.1	3.2	5,325	74.0	15.2	9.8	0.9	648
**Age at consummation of marriage**									
Below 18 years	12.2	9.6	2.6	9,184	78.4	12.1	9.0	0.3	1,116
18 years & above	10.3	7.8	2.6	12,227	75.3	12.1	10.7	1.2	1,265
**Marital duration**									
0-4	7.6	5.9	1.7	4,202	78.0	5.2	15.0	1.6	325
5-9	14.1	10.2	3.8	3,575	72.7	14.4	12.4	0.6	506
10-14	11.3	8.2	3.0	3,481	73.0	14.8	10.5	1.2	390
15+	11.5	9.1	2.4	10,153	79.4	12.1	7.2	0.6	1,160
**Education**									
Non-literate[a]	11.5	9.2	2.3	8,920	80.1	12.8	5.9	0.8	1,023
Less than 5 yrs	13.9	10.4	3.5	918	75.1	13.4	9.9	0.0	128
5-9 years	11.6	8.9	2.7	6,348	76.6	10.2	11.7	1.1	734
10 or more years	9.5	6.7	2.8	5,225	70.3	13.0	15.6	0.8	496
**Husband's education**									
Non-literate[a]	10.9	8.6	2.3	3,876	78.6	13.6	5.5	0.9	424
Less than 5 years	13.4	10.4	3.0	765	77.8	7.1	14.1	0.0	103
5-9 years	12.0	9.2	2.7	6,873	77.2	13.6	8.1	1.1	820
10 or more years	10.5	7.9	2.6	9,897	75.5	10.7	12.7	0.7	1,034
**Religion**									
Hindu	11.3	8.7	2.6	18,662	76.7	11.7	10.3	0.8	2,102
Muslim	9.8	7.8	2.0	1,408	79.4	12.3	6.4	0.6	138
Sikh	10.7	7.9	2.8	1,315	73.6	17.2	6.4	1.4	141
Others	0.0	0.0	0.0	26	nc	nc	nc	nc	0
**Castes/Tribes**									
Scheduled Castes	11.3	8.3	2.9	4,907	74.2	15.6	9.4	0.7	554
Scheduled Tribes	7.7	3.3	4.4	87	*	*	*	*	7
Other Backward Classes	11.1	8.7	2.4	7,655	78.4	10.8	9.7	0.8	846
Others	11.1	8.6	2.6	8,759	77.0	11.2	10.2	0.9	973
**Wealth index**									
Lowest	7.7	6.1	1.6	258	(80.0)	(10.0)	(10.0)	(0.0)	20
Second	11.2	8.7	2.4	1,262	78.3	11.9	9.1	0.0	142
Middle	10.2	8.0	2.3	4,183	77.9	13.5	8.2	0.2	429
Fourth	11.8	9.1	2.6	6,705	77.5	11.9	8.8	1.5	782
Highest	11.2	8.4	2.8	9,002	75.3	11.7	11.6	0.7	1,008
**Haryana (15-49)**	11.1	8.5	2.6	21,411	76.7	12.1	9.9	0.8	2,381
**Haryana(15-44)**[†]	11.1	8.4	2.7	18,575	75.5	12.5	10.6	1.0	2,064

Note: Total figure may not add to 100 percent due to 'do not know' or 'missing cases'.
nc= Not calculated because there are no cases.
* Percentage not shown; based on less than 10 unweighted cases.
** Unweighted cases.
( ) Based on 10-24 unweighted cases.
[a] Literates but did not attend school, are also included.
[1] Excluded women got married but g*auna* not performed.
[2]Includes problems after pelvic surgery and other incidents.
[†] Represents figures for currently married women aged 15-44 years.

TABLE 7.15 CHILDLESSNESS AND INFERTILITY BY BACKGROUND CHARACTERISTICS

Percentage of currently married women aged 20-49 and 40-49 years having no living children, and who have been married for at least five years, according to selected background characteristics, Haryana, 2007-08

Background characteristics	20-49 aged women			40-49 aged women		
	Percentage childless	Infertility[1]	Number of women**	Percentage childless	Infertility[1]	Number of women**
**Residence**						
Rural	1.9	1.6	12,137	1.0	1.0	3,169
Urban	2.1	1.7	4,032	0.5	0.4	1,219
**Age at consummation of marriage**						
Below 18 years	1.9	1.4	7,730	0.9	0.9	2,238
18 years & above	2.1	1.8	8,439	0.9	0.8	2,150
**Marital duration**						
5-9	4.9	3.7	3,450	*	*	2
10-14	1.6	1.3	3,386	*	*	9
15+	1.0	1.0	9,333	0.9	0.8	4,377
**Education**						
Non-literate[a]	1.8	1.6	7,529	0.9	0.9	2,750
Less than 5 yrs	1.8	1.2	731	1.4	1.4	147
5-9 years	2.1	1.7	4,614	0.7	0.7	896
10 or more years	2.2	1.6	3,295	0.8	0.6	595
**Husband's education**						
Non-literate[a]	1.9	1.5	3,224	1.2	1.2	1,134
Less than 5 years	2.3	2.2	634	0.6	0.6	177
5-9 years	1.8	1.5	5,302	0.6	0.6	1,371
10 or more years	2.2	1.6	7,009	0.9	0.8	1,706
**Religion**						
Hindu	2.0	1.6	14,133	0.9	0.9	3,832
Muslim	2.1	1.6	1,019	0.0	0.0	225
Sikh	2.1	1.9	996	1.2	1.2	324
Others	(0.0)	(0.0)	21	*	*	7
**Castes/Tribes**						
Scheduled Castes	1.8	1.5	3,650	1.0	1.0	952
Scheduled Tribes	0.0	0.0	67	(0.0)	(0.0)	16
Other Backward Classes	2.2	1.8	5,728	0.8	0.8	1,505
Others	1.9	1.5	6,721	0.9	0.8	1,913
**Wealth index**						
Lowest	1.0	1.0	203	0.0	0.0	46
Second	3.5	2.5	1,010	3.0	3.0	230
Middle	1.6	1.2	3,303	0.6	0.6	776
Fourth	2.0	1.7	5,034	1.1	1.1	1,262
Highest	1.9	1.6	6,618	0.6	0.5	2,074
**Haryana**	2.0	1.6	16,169	0.9	0.8	4,388

( ) Based on 10-24 unweighted cases.
* Percentage not shown; based on less than 10 unweighted cases.
** Unweighted cases.
[a] Literates but did not attend school, are also included.
[1] Women with no living children and reported problem in conceiving (involuntary infertility).

TABLE 7.16 TREATMENT FOR INFERTILITY BY BACKGROUND CHARACTERISTICS

Percentage of currently married women age d15-49 years who sought treatment for primary/secondary infertility problem, according to selected background characteristics, Haryana, 2007-08

	For primary Infertility			For secondary Infertility		
Background Characteristics	Percentage of women sought treatment	Percentage of women taken allopathic treatment	Number of women having primary infertility**	Percentage of women sought treatment	Percentage of women taken allopathic treatment	Number of women having secondary infertility**
**Age group**						
15-19	72.7	63.9	45	(83.3)	(83.3)	12
20-24	92.2	76.4	338	83.4	68.5	107
25-29	92.9	81.9	359	86.0	75.9	136
30-34	95.0	81.0	314	86.0	68.8	120
35-39	88.5	72.8	300	85.9	77.1	79
40-44	87.9	66.6	257	91.5	69.6	61
45-49	77.8	55.8	213	87.5	67.5	40
**Residence**						
Rural	88.6	71.5	1,344	85.5	69.3	389
Urban	91.9	79.3	482	87.5	78.0	166
**Age at consummation of marriage**						
Below 18 years	85.2	66.5	874	84.8	69.4	242
18 years & above	93.4	80.1	952	87.2	73.9	313
**Marital duration**						
0-4	89.0	77.3	253	86.9	77.3	72
5-9	94.4	80.2	368	87.5	75.4	138
10-14	93.9	81.7	284	82.0	69.2	106
15+	86.2	67.4	921	86.9	69.6	239
**Education**						
Non-literate[a]	85.2	64.9	819	81.8	59.8	204
Less than 5 yrs	89.4	73.8	96	71.9	53.1	32
5-9 years	91.4	75.6	561	90.1	80.5	173
10 or more years	96.3	90.9	350	90.6	82.8	146
**Husband's education**						
Non-literate[a]	84.2	60.0	333	81.1	53.4	91
Less than 5 years	90.1	67.7	80	(82.6)	(60.9)	23
5-9 years	89.5	74.5	632	83.1	69.0	188
10 or more years	91.6	79.3	781	90.6	81.8	253
**Religion**						
Hindu	90.1	74.7	1,613	86.6	72.4	489
Muslim	77.3	51.2	109	75.9	51.7	29
Sikh	93.2	82.6	104	89.2	83.8	37
Others	nc	nc	0	nc	nc	0
**Castes/Tribes**						
Scheduled Castes	90.6	71.4	411	77.6	62.3	143
Scheduled Tribes	*	*	3	*	*	4
Other Backward Classes	87.7	69.5	664	89.1	71.4	182
Others	90.4	78.3	748	88.8	77.9	225
**Wealth index**						
Poorest	(68.8)	(43.8)	16	*	*	4
Second	81.9	61.0	111	70.0	60.0	31
Middle	88.3	65.3	334	81.0	56.7	95
Fourth	87.4	71.7	606	83.2	66.3	176
Highest	93.2	81.3	759	91.8	83.5	249
**Haryana**	89.4	73.6	1,826	86.1	71.9	555

nc= Not calculated because there are no cases.
( ) Based on 10-24 unweighted cases.
* Percentage not shown; based on less than 10 unweighted cases.
** Unweighted cases.
[a] Literates but did not attend school, are also included.

TABLE 7.17 INFERTILITY PROBLEM AND SOUGHT TREATMENT BY DISTRICTS

Percentage of ever married women aged 15-49 years who had ever menstruation problem during last three months prior to survey, ever had infertility problem and among women who had infertility problem, sought treatment for infertility, by districts, Haryana, 2007-08

Districts	Who have menstruation problem	Who have infertility problem[1]	Who sought treatment for infertility
Panchkula	22.0	12.2	90.8
Ambala	24.9	10.6	94.5
Yamunanagar	25.7	11.0	97.0
Kurukshetra	16.8	13.7	92.1
Kaithal	17.8	15.0	88.0
Karnal	17.5	12.8	84.0
Panipat	21.2	14.9	92.4
Sonipat	15.4	13.3	93.7
Jind	18.6	11.5	84.4
Fatehabad	10.5	7.8	88.3
Sirsa	12.2	10.1	87.3
Hisar	13.9	7.3	90.0
Bhiwani	19.0	8.6	77.5
Rohtak	14.8	11.9	92.4
Jhajjar	14.7	10.8	89.5
Mahendragarh	20.0	10.2	78.5
Rewari	18.2	13.1	81.5
Gurgaon	16.9	11.8	90.0
Faridabad	15.1	12.7	95.3
Mewat	19.8	8.4	73.8
**Haryana (15-49)**	18.0	11.1	88.6
**Haryana(15-44)** [†]	17.5	11.1	89.7

Note: Women excluding pregnant, in amenorrhea, in menopause, had hysterectomy and never menstruated women.
[1] Included both primary and secondary infertility.
[†] Represents figures for currently married women aged 15-44 years.

CHARACTERISTICS OF UNMARRIED WOMEN

TABLE 8.1 BACKGROUND CHARACTERISTICS OF UNMARRIED WOMEN

Percent distribution of unmarried women aged 15-24 years, according to selected background characteristics, Haryana, 2007-08

Background characteristics	Total	Residence	
		Rural	Urban
**Age group**			
15-19	78.8	83.8	66.5
20-24	21.2	16.2	33.5
**Education**			
Non-literate[a]	5.5	6.5	3.0
Less than five years	3.6	4.2	2.2
5-9 years	35.9	41.8	21.1
10 or more years	55.0	47.5	73.6
**Religion**			
Hindu	87.1	85.5	91.2
Muslim	4.9	6.0	2.3
Sikh	7.6	8.4	5.5
Others	0.4	0.1	0.1
**Castes/tribes**			
Scheduled castes	24.7	25.7	22.2
Scheduled tribes	0.5	0.4	0.6
Other backward classes	32.8	34.6	28.5
Others	42.0	39.3	48.6
**Wealth index**			
Lowest	0.9	1.0	0.5
Second	5.3	7.1	0.9
Middle	18.4	23.8	5.1
Fourth	30.6	36.1	16.9
Highest	44.8	31.9	76.5
**Haryana****	5,031	3,585	1,446

** Unweighted cases.
[a] Literates but did not attend school, are also included.

TABLE 8.2 AT WHAT AGE AND STANDARD FAMILY LIFE EDUCATION SHOULD BE INTRODUCED

Percentage of unmarried women aged 15-24 years aware of family life education, perceived family life education is important and percent distribution of women stating ideal age and standard at which family life education should be introduced, according to selected background characteristics, Haryana, 2007-08

	Percentage of women			At what age family life education should be introduced[b]					From which standard family life education should be introduced[b]				
Background characteristics	Aware of family life education	Perceived family life education is important	Number of women**	Below 12	12-14	15 - 17	18 or above	Total	Below 8	8-9	10 or more	Total	Number of women who perceived family life education is important**
**Age group**													
15-19	73.1	88.0	3,966	4.7	33.4	37.5	24.4	100.0	18.1	36.9	45.0	100.0	3,497
20-24	85.8	95.3	1,065	4.6	29.4	36.7	29.4	100.0	14.0	34.5	51.5	100.0	1,013
**Residence**													
Rural	72.7	88.1	3,585	4.8	32.8	36.4	25.9	100.0	18.4	36.8	44.8	100.0	3,170
Urban	83.4	93.1	1,446	4.3	31.6	39.6	24.5	100.0	14.3	35.4	50.2	100.0	1,340
**Education**													
Non-literate[a]	48.4	60.2	262	6.9	27.9	32.5	32.7	100.0	18.0	24.1	58.0	100.0	157
Less than five years	51.3	64.3	177	2.8	25.1	39.2	32.9	100.0	23.9	26.3	49.8	100.0	114
5-9 years	66.5	84.7	1,816	5.6	33.6	34.0	26.8	100.0	25.3	39.2	35.4	100.0	1,540
10 or more years	86.2	97.2	2,776	4.1	32.4	39.5	24.0	100.0	12.2	36.0	51.8	100.0	2,699
**Religion**													
Hindu	76.4	90.2	4,402	4.4	32.8	37.3	25.4	100.0	17.2	36.8	46.0	100.0	3,972
Muslim	62.3	68.3	222	6.6	31.7	33.2	28.5	100.0	17.3	26.9	55.7	100.0	152
Sikh	76.9	94.8	389	5.9	28.8	40.1	25.2	100.0	16.6	37.2	46.2	100.0	369
Others	(88.9)	(94.4)	18	(11.8)	(29.4)	(41.2)	(17.6)	(100.0)	(17.6)	(11.8)	(70.6)	(100.0)	17
**Castes/tribes[#]**													
Scheduled castes	68.5	83.1	1,246	4.5	31.7	34.2	29.5	100.0	22.1	34.3	43.5	100.0	1,035
Scheduled tribes	(79.2)	(91.7)	24	(9.1)	(31.8)	(22.7)	(36.4)	(100.0)	(36.4)	(27.3)	(36.4)	(100.0)	22
Other backward classes	73.9	88.2	1,647	5.2	31.5	37.0	26.3	100.0	18.1	36.7	45.1	100.0	1,460
Others	81.6	94.3	2,112	4.3	33.6	39.3	22.7	100.0	13.6	37.3	49.1	100.0	1,991
**Wealth index**													
Lowest	52.8	56.5	40	(4.3)	(56.5)	(21.7)	(17.4)	(100.0)	(47.8)	(39.1)	(13.0)	(100.0)	23
Second	59.8	74.9	263	5.6	31.7	33.4	29.4	100.0	21.8	35.2	43.1	100.0	198
Middle	63.8	82.6	923	5.3	33.0	35.3	26.4	100.0	23.0	36.2	40.9	100.0	764
Fourth	73.4	87.7	1,555	4.9	32.9	36.7	25.5	100.0	18.1	36.3	45.6	100.0	1,366
Highest	84.7	96.0	2,250	4.2	31.8	39.1	24.9	100.0	13.7	36.6	49.6	100.0	2,159
**Haryana**	75.8	89.5	5,031	4.7	32.5	37.4	25.5	100.0	17.2	36.4	46.4	100.0	4,510

[#] Total figure may not add to sample total (N), due to 'don't know' or 'missing cases'.
( ) Based on 10-24 unweighted cases.
* Percentage not shown, based on less than 10 unweighted cases.
** Unweighted cases.
[a] Literates but did not attend school, are also included.
[b] Among women who perceived the family life education is important.

TABLE 8.3 SOURCES OF FAMILY LIFE EDUCATION

Percentage of unmarried women aged 15-24 years who perceived family life education to be important and sources of family life education, according to selected background characteristics, Haryana, 2007-08

Background characteristics	Perceived family life education to be important	Number of women**	Sources of family life education: Parents	Brother/ sister / sister in law	Husband/ partner	Friends/ peers	Health care provider/ sex education experts	Youth club/ *mandal* / NGO worker	Teacher / school/ college	Other	Number of women who perceived family life education to be important**
**Age group**											
15-19	88.0	3,966	83.4	57.5	1.2	25.3	3.5	0.9	47.8	1.0	3,497
20-24	95.3	1,065	84.9	62.0	2.1	29.8	6.6	2.6	54.5	1.6	1,013
**Residence**											
Rural	88.1	3,585	82.1	58.3	1.3	25.1	3.9	1.1	47.1	1.1	3,170
Urban	93.1	1,446	87.6	58.8	1.6	29.2	4.8	1.7	54.6	1.2	1,340
**Education**											
Non-literate[a]	60.2	262	79.0	52.8	0.7	15.0	1.2	1.3	8.1	1.4	157
Less than five years	64.3	177	73.0	61.8	0.0	24.0	0.9	0.0	13.4	0.0	114
5-9 years	84.7	1,816	82.1	59.4	1.4	22.8	2.1	0.5	37.9	0.9	1,540
10 or more years	97.2	2,776	85.4	58.2	1.5	29.1	5.6	1.8	59.8	1.3	2,699
**Religion**											
Hindu	90.2	4,402	84.3	58.2	1.3	27.0	4.0	1.1	50.4	1.2	3,972
Muslim	68.3	222	75.8	62.4	1.4	19.9	0.7	0.7	13.8	0.7	152
Sikh	94.8	389	81.2	58.8	1.4	22.4	7.2	3.3	54.6	1.0	369
Others	*	4	*	*	*	*	*	*	*	*	4
**Castes/tribes**[#]											
Scheduled castes	83.1	1,246	83.7	58.3	1.1	21.9	2.9	0.8	43.0	1.1	1,035
Scheduled tribes	(91.7)	24	(95.5)	(72.7)	(0.0)	(13.6)	(0.0)	(0.0)	(36.4)	(0.0)	22
Other backward classes	88.2	1,647	81.2	60.1	1.6	24.3	4.3	1.4	45.8	1.0	1,460
Others	94.3	2,112	85.5	57.1	1.4	30.2	4.8	1.4	55.4	1.2	1,991
**Wealth index**											
Lowest	56.5	40	(91.3)	(56.5)	(0.0)	(21.7)	(0.0)	(0.0)	(8.7)	(0.0)	23
Second	74.9	263	81.1	51.4	1.5	24.4	0.0	0.0	28.3	1.5	198
Middle	82.6	923	82.5	57.9	1.7	25.1	1.5	0.7	37.1	1.0	764
Fourth	87.7	1,555	81.1	59.8	0.8	24.0	3.2	0.9	46.5	0.7	1,366
Highest	96.0	2,250	86.0	58.6	1.7	28.4	6.1	1.9	57.8	1.4	2,159
**Haryana**	89.5	5,031	83.7	58.5	1.4	26.3	4.2	1.3	49.3	1.1	4,510

[#] Total figure may not add to N, sample total due to 'do not know' or 'missing cases'.
( ) Based on 10-24 unweighted cases.
* Percentage not shown, based on less than 10 unweighted cases.
** Unweighted cases.
[a] Literates but did not attend school, are also included.

TABLE 8.4 EVER RECEIVED FAMILY LIFE EDUCATION BY SOURCES

Percentage of unmarried women aged 15-24 years who ever received family life education by sources, according to selected background characteristics, Haryana, 2007-08

Background characteristics	Ever received family life education	Number of women**	Source of family life education					Number of women ever received family life education**
			NGO programme/ camp	Govt. programme/ camp	School/ college	Youth club	Other	
**Age group**								
15-19	42.0	3,966	1.5	2.2	85.6	3.5	15.7	1,674
20-24	55.0	1,065	3.4	5.3	84.0	7.9	16.7	587
**Residence**								
Rural	41.5	3,585	2.0	2.8	85.0	4.3	15.0	1,499
Urban	52.8	1,446	1.8	3.3	85.5	5.4	17.8	762
**Education**								
Non-literate[a]	7.1	262	(0.0)	(10.5)	(5.3)	(21.1)	(63.2)	19
Less than five years	8.5	177	(0.0)	(0.0)	(31.3)	(25.0)	(56.3)	16
5-9 years	30.7	1,816	1.3	2.5	79.2	4.2	20.4	558
10 or more years	60.1	2,776	2.2	3.1	88.6	4.5	13.6	1,668
**Religion**								
Hindu	46.0	4,402	1.5	2.9	85.4	4.3	16.0	2,023
Muslim	13.9	222	0.0	2.5	65.6	15.5	27.3	33
Sikh	49.7	389	7.2	3.6	86.3	7.2	14.5	194
Others	*	4	*	*	*	*	*	3
**Castes/tribes[#]**								
Scheduled castes	36.1	1,246	1.5	3.7	78.2	5.2	22.5	447
Scheduled tribes	(33.3)	24	*	*	*	*	*	8
Other backward classes	41.6	1,647	1.7	2.8	84.8	4.1	16.1	697
Others	52.5	2,112	2.3	2.7	88.3	4.7	13.2	1,108
**Wealth index**								
Lowest	0.0	40	nc	nc	nc	nc	nc	nc
Second	24.2	263	1.1	4.2	81.8	4.8	18.5	66
Middle	30.4	923	1.6	1.5	84.6	4.3	16.5	282
Fourth	41.3	1,555	2.4	3.3	87.0	4.9	14.8	643
Highest	56.4	2,250	1.5	6.0	69.7	2.9	22.8	1,270
**Haryana**	44.8	5,031	2.0	3.0	85.2	4.7	16.0	2,261

[#] Total figure may not add to N, sample total due to ‘do not know’ or ‘missing cases’.
nc = Not calculated because there are no cases.
( ) Based on 10-24 unweighted cases.
* Percentage not shown, based on less than 10 unweighted cases.
** Unweighted cases.
[a] Literates but did not attend school, are also included.

TABLE 8.5 KNOWLEDGE OF LEGAL AGE AT MARRIAGE AND REPORTED IDEAL AGE AT MARRIAGE FOR BOYS AND GIRLS

Percentage of unmarried women aged 15-24 years who have knowledge about legally prescribed minimum age at marriage in India for boys and girls and reported ideal age at marriage for boys and girls, according to selected background characteristics, Haryana, 2007-08

	Knowledge of minimum legal age at marriage		Reported ideal age at marriage for boys			Reported ideal age at marriage for girls			
Background characteristics	Boys	Girls	Less than 21	21 and above	Total	Less than 18	18 and above	Total	Number of women**
**Age group**									
15-19	92.0	93.7	11.8	88.2	100.0	1.8	98.2	100.0	3,966
20-24	97.1	97.4	7.3	92.7	100.0	0.7	99.3	100.0	1,065
**Residence**									
Rural	92.3	93.9	11.6	88.4	100.0	1.7	98.3	100.0	3,585
Urban	95.1	95.9	8.8	91.2	100.0	1.1	98.9	100.0	1,446
**Education**									
Non-literate[a]	60.8	63.4	34.0	66.0	100.0	10.4	89.6	100.0	262
Less than five years	73.6	77.9	22.3	77.7	100.0	4.5	95.5	100.0	177
5-9 years	91.1	93.2	14.3	85.7	100.0	1.3	98.7	100.0	1,816
10 or more years	99.0	99.6	6.7	93.3	100.0	1.0	99.0	100.0	2,776
**Religion**									
Hindu	94.1	95.6	9.9	90.1	100.0	1.2	98.8	100.0	4,402
Muslim	71.4	73.1	29.8	70.2	100.0	10.7	89.3	100.0	222
Sikh	95.6	96.1	12.0	88.0	100.0	0.5	99.5	100.0	389
Others	*	*	*	*	*	*	*	*	4
**Castes/tribes**[#]									
Scheduled castes	89.4	91.6	12.3	87.7	100.0	1.1	98.9	100.0	1,246
Scheduled tribes	(100.0)	(95.8)	(20.8)	(79.2)	(100.0)	(4.3)	(95.7)	(100.0)	24
Other backward classes	91.1	92.6	12.3	87.7	100.0	2.3	97.7	100.0	1,647
Others	96.8	97.6	8.8	91.2	100.0	1.1	98.9	100.0	2,112
**Wealth index**									
Lowest	71.3	73.9	14.7	85.3	100.0	0.0	100.0	100.0	40
Second	81.5	82.0	23.6	76.4	100.0	3.8	96.2	100.0	263
Middle	88.7	91.1	15.6	84.4	100.0	2.9	97.1	100.0	923
Fourth	92.5	94.0	10.5	89.5	100.0	1.0	99.0	100.0	1,555
Highest	97.1	98.1	7.9	92.1	100.0	1.1	98.9	100.0	2,250
**Haryana**	93.1	94.5	10.8	89.2	100.0	1.5	98.5	100.0	5,031

[#] Total figure may not add to N, sample total due to 'do not know' or 'missing cases'.
( ) Based on 10-24 unweighted cases.
* Percentage not shown, based on less than 10 unweighted cases.
** Unweighted cases.
[a] Literates but did not attend school, are also included.

TABLE 8.6 CURRENT STATUS OF MENSTRUATION AND EXPERIENCED MENSTRUATION RELATED PROBLEMS DURING LAST THREE MONTHS AND REPORTED PROBLEMS

Percentage of unmarried women aged 15-24 years who are currently menstruating and reported menstruation related problems during the last three months preceding the survey, according to selected background characteristics, Haryana, 2007-08

Background characteristics	Having menstruation related problems during last three months	Number of women**	During last three months having menstruation related problems								Number of women with menstruation related problems**
			No periods	Painful periods	Frequent or short periods	Irregular periods	Prolonged bleeding	Scanty bleeding	Inter-menstrual bleeding	Blood clots/ excessive bleeding	
**Age group**											
15-19	16.0	3,966	5.6	80.9	5.8	11.7	5.6	3.4	0.6	2.3	626
20-24	15.3	1,065	5.0	82.7	6.3	11.0	4.3	4.5	0.6	2.4	164
**Residence**											
Rural	16.2	3,585	4.0	82.5	7.1	10.7	5.5	3.4	0.8	2.2	572
Urban	15.0	1,446	9.4	78.1	2.6	14.0	5.1	4.4	0.0	2.6	218
**Education**											
Non-literate[a]	16.7	262	7.5	83.0	7.0	4.9	3.2	3.3	0.0	0.0	41
Less than five years	19.1	177	5.7	97.3	3.0	5.6	0.0	6.0	0.0	0.0	33
5-9 years	15.8	1,816	3.5	84.3	5.3	12.1	5.1	2.7	1.4	2.0	283
10 or more years	15.6	2,776	6.5	77.9	6.4	12.5	6.2	4.1	0.2	2.9	433
**Religion**											
Hindu	15.8	4,402	5.4	80.2	6.0	12.1	5.9	4.0	0.4	2.4	688
Muslim	19.5	222	7.6	90.3	2.1	2.7	0.0	0.8	0.0	0.0	42
Sikh	14.6	389	5.1	87.5	6.8	13.9	3.4	1.6	3.6	3.4	57
Others	*	4	nc	nc	nc	nc	nc	nc	nc	nc	nc
**Castes/tribes**[#]											
Scheduled castes	18.9	1,246	3.4	79.7	7.2	14.0	6.1	5.7	0.4	2.8	231
Scheduled tribes	(25.0)	24	*	*	*	*	*	*	*	*	6
Other backward classes	16.7	1,647	5.9	87.0	4.6	8.2	3.8	1.2	0.0	2.5	274
Others	13.2	2,112	6.9	76.5	6.2	13.3	6.0	4.5	1.4	1.7	278
**Wealth index**											
Lowest	18.8	40	*	*	*	*	*	*	*	*	7
Second	18.4	263	2.5	83.5	8.7	8.1	4.0	5.8	0.0	0.0	46
Middle	17.1	923	4.5	83.1	2.5	14.0	5.3	2.0	0.6	0.6	155
Fourth	16.6	1,555	4.0	84.1	7.6	9.4	4.2	4.1	0.7	3.0	256
Highest	14.5	2,250	7.3	77.8	5.9	13.0	6.6	3.8	0.6	2.9	326
**Haryana**	15.8	5,031	5.4	81.3	5.9	11.6	5.4	3.6	0.6	2.3	790

[#] Total figure may not add to N, sample total due to 'do not know' or 'missing cases'.
nc = Not calculated because there are no cases.
( ) Based on 10-24 unweighted cases.
* Percentage not shown, based on less than 10 unweighted cases.
** Unweighted cases.
[a] Literates but did not attend school, are also included.

TABLE 8.7 PRACTICES DURING MENSTRUAL PERIOD

Percentage of unmarried women aged 15-24 years who are currently menstruating by practices during menstrual period, according to selected background characteristics, Haryana, 2007-08

	Practices during menstrual period				
Background characteristics	Used cloth	Locally prepared napkins	Use sanitary napkins	Used other	Number of women currently menstruating
**Age group**					
15-19	77.4	3.4	31.7	0.0	3,966
20-24	60.1	6.4	54.4	0.1	1,065
**Residence**					
Rural	81.5	2.8	28.4	0.1	3,585
Urban	54.4	7.0	56.8	0.0	1,446
**Education**					
Non-literate[a]	96.2	0.5	2.2	0.0	262
Less than five years	93.6	0.9	4.2	0.0	177
5-9 years	89.7	2.1	17.8	0.1	1,816
10 or more years	59.9	5.8	54.1	0.1	2,776
**Religion**					
Hindu	72.6	3.5	37.5	0.0	4,402
Muslim	93.5	3.4	6.0	0.0	222
Sikh	75.2	9.0	44.1	0.2	389
Others	*	*	*	*	4
**Castes/tribes**[#]					
Scheduled castes	84.6	2.4	21.6	0.1	1,246
Scheduled tribes	(79.2)	(12.5)	(20.8)	(0.0)	24
Other backward classes	80.6	4.5	30.3	0.1	1,647
Others	61.8	4.5	50.4	0.0	2,112
**Wealth index**					
Lowest	100.0	0.0	0.0	0.0	40
Second	96.7	0.5	6.6	0.0	263
Middle	91.1	1.1	12.1	0.0	923
Fourth	85.6	2.2	24.2	0.1	1,555
Highest	55.3	7.0	59.1	0.1	2,250
**Haryana**	73.7	4.0	36.6	0.1	5,031

[#] Total figure may not add to N, sample total due to ‘do not know’ or ‘missing cases’.
( ) Based on 10-24 unweighted cases.
* Percentage not shown, based on less than 10 unweighted cases.
** Unweighted cases.
[a] Literates but did not attend school, are also included.

TABLE 8.8 KNOWLEDGE OF CONTRACEPTIVE METHODS

Percentage of unmarried women aged 15-24 years who know any contraceptive method by specific methods, according to selected background characteristics, Haryana, 2007-08

Background characteristics	Any method	Any modern method	Male sterilization	Female sterilization	IUD	Pill	Emergency contraception	Injectable	Condom/ Nirodh	Female condom	Rhythm method	Withdrawal	Other	Number of Women**
**Age group**														
15-19	96.8	96.7	78.2	94.2	58.7	83.6	25.5	46.0	76.0	8.9	14.6	4.1	0.3	3,966
20-24	98.5	98.5	88.7	96.5	79.0	94.8	48.0	59.7	92.0	16.3	26.8	9.1	0.7	1,065
**Residence**														
Rural	96.8	96.7	79.8	94.6	61.9	84.2	24.5	47.3	77.3	9.2	16.0	4.4	0.4	3,585
Urban	98.1	98.1	81.9	94.9	65.8	90.5	44.5	52.9	84.7	13.7	19.9	7.1	0.4	1,446
**Education**														
Non-literate[a]	89.6	89.2	59.0	87.8	30.9	56.3	4.0	27.4	39.9	0.0	7.2	0.4	0.0	262
Less than five years	94.3	94.3	65.4	92.1	46.2	69.3	6.5	30.6	57.0	1.3	8.2	0.7	0.0	177
5-9 years	95.8	95.7	72.9	92.6	52.2	78.1	17.1	39.7	70.0	6.2	11.6	3.6	0.5	1,816
10 or more years	99.0	99.0	88.4	97.0	74.4	95.2	43.0	58.3	91.0	14.9	22.4	7.0	0.4	2,776
**Religion**														
Hindu	97.5	97.5	81.8	95.4	64.4	86.9	31.4	50.2	81.2	10.7	17.3	5.4	0.4	4,402
Muslim	90.6	89.6	68.0	87.5	42.7	68.2	6.9	34.6	43.0	2.0	11.0	1.8	0.0	222
Sikh	97.1	97.1	72.2	91.9	59.5	86.1	29.9	41.5	81.8	13.1	18.6	4.0	0.2	389
Others	*	*	*	*	*	*	*	*	*	*	*	*	*	4
**Castes/tribes[#]**														
Scheduled castes	96.6	96.6	76.5	93.9	56.0	81.6	22.2	42.7	74.7	7.2	13.6	3.4	0.4	1,246
Scheduled tribes	(91.7)	(91.7)	(66.7)	(87.5)	(58.3)	(87.5)	(37.5)	(50.0)	(75.0)	(29.2)	(25.0)	(16.7)	(0.0)	24
Other backward classes	96.3	96.1	80.1	93.9	60.0	84.3	25.5	48.9	76.4	10.4	17.5	4.4	0.3	1,647
Others	98.3	98.3	83.0	95.9	69.5	89.8	38.6	52.4	84.5	12.3	18.9	6.7	0.5	2,112
**Wealth index**														
Lowest	95.4	95.4	61.1	93.4	53.9	71.2	2.1	42.3	47.2	0.0	16.0	2.8	0.0	40
Second	93.1	93.1	69.1	90.9	41.6	66.8	9.5	27.6	52.6	2.9	14.9	3.9	0.7	263
Middle	95.7	95.6	74.8	93.5	51.3	77.5	13.4	41.1	67.8	5.1	9.8	2.7	0.5	923
Fourth	97.1	97.0	80.8	94.8	62.1	84.9	23.7	47.6	78.6	8.5	15.5	4.6	0.4	1,555
Highest	98.3	98.3	84.1	95.6	71.2	92.8	44.7	55.7	88.6	15.2	21.6	6.8	0.3	2,250
**Haryana**	97.2	97.1	80.4	94.7	63.0	86.0	30.2	48.9	79.4	10.5	17.2	5.2	0.4	5,031

[#] Total figure may not add to N, sample total due to 'do not know' or 'missing cases'.
( ) Based on 10-24 unweighted cases.
* Percentage not shown, based on less than 10 unweighted cases.
** Unweighted cases.
[a] Literates but did not attend school, are also included.

TABLE 8.9 SOURCES FROM WHERE TO GET PILL AND CONDOM

Percentage of unmarried women aged 15-24 years who reported source from where to get pill and condom, according to selected background characteristics, Haryana, 2007-08

Background characteristics	Sources from where to get pills and condoms			Number of women**
	Government facility	Private facility	Other	
**Age group**				
15-19	66.0	55.7	0.2	3,966
20-24	76.6	68.4	0.5	1,065
**Residence**				
Rural	68.7	54.1	0.3	3,585
Urban	67.0	69.2	0.3	1,446
**Education**				
Non-literate[a]	38.0	31.7	0.0	262
Less than five years	55.9	42.3	0.0	177
5-9 years	64.1	48.5	0.3	1,816
10 or more years	74.7	68.6	0.3	2,776
**Religion**				
Hindu	68.9	59.3	0.3	4,402
Muslim	46.2	41.2	0.5	222
Sikh	73.5	58.5	0.3	389
Others	*	*	*	4
**Castes/tribes**[#]				
Scheduled castes	65.9	48.7	0.4	1,246
Scheduled tribes	(79.2)	(41.7)	(0.0)	24
Other backward classes	67.2	56.1	0.4	1,647
Others	70.2	66.1	0.2	2,112
**Wealth index**				
Lowest	57.2	28.6	0.0	40
Second	55.3	35.5	0.0	263
Middle	65.2	47.1	0.1	923
Fourth	68.9	54.1	0.2	1,555
Highest	70.8	69.3	0.4	2,250
**Haryana**	68.2	58.4	0.3	5,031

[#] Total figure may not add to N, sample total due to ‘do not know’ or ‘missing cases’.
( ) Based on 10-24 unweighted cases.
* Percentage not shown, based on less than 10 unweighted cases
** Unweighted cases.
[a] Literates but did not attend school, are also included.

TABLE 8.10 DISCUSSION ABOUT FAMILY PLANNING METHOD BY SOURCE OF INFORMATION

Percentage of unmarried women aged 15-24 years who discussed family planning methods with any one and sources of information, according to selected background characteristics, Haryana, 2007-08

			Sources of information on family planning							
Background characteristics	Ever discussed about family planning with any one	Number of women**	Parents	Brother/ Sister/ Sister in law	Friends/ peers	Health care provider/ sex education experts	Youth club/ *mandal*/ NGO worker	Teacher / school/ college	Other	Number of women ever discussed about family planning with any one**
**Age group**										
15-19	11.9	3,966	7.6	30.7	71.9	2.6	0.0	12.4	1.1	475
20-24	20.6	1,065	13.0	38.6	76.2	7.8	1.0	16.6	0.0	223
**Residence**										
Rural	12.3	3,585	7.6	37.1	71.0	3.5	0.2	10.4	1.2	446
Urban	17.4	1,446	12.4	26.5	77.4	5.6	0.4	19.7	0.0	252
**Education**										
Non-literate[a]	3.8	262	(20.0)	(60.0)	(50.0)	(0.0)	(0.0)	(0.0)	(0.0)	10
Less than five years	6.6	177	(0.0)	(54.5)	(63.6)	(0.0)	(9.1)	(0.0)	(9.1)	11
5-9 years	8.9	1,816	6.8	36.6	67.1	2.4	0.0	5.7	1.9	163
10 or more years	18.3	2,776	10.1	31.1	76.0	5.0	0.2	16.9	0.2	514
**Religion**										
Hindu	13.8	4,402	8.8	32.4	73.9	4.1	0.3	13.2	0.7	613
Muslim	7.1	222	(6.7)	(40.0)	(66.7)	(0.0)	(0.0)	(0.0)	(0.0)	15
Sikh	16.1	389	12.7	38.2	68.3	6.4	0.0	20.5	1.8	63
Others	*	4	nc	nc	nc	nc	nc	nc	nc	nc
**Castes/tribes[#]**										
Scheduled castes	10.7	1,246	5.0	40.7	68.2	7.4	0.8	15.7	0.9	135
Scheduled tribes	(20.8)	24	*	*	*	*	*	*	*	5
Other backward classes	13.3	1,647	10.9	36.4	70.4	4.1	0.0	9.0	0.9	222
Others	15.7	2,112	9.9	27.7	76.7	3.2	0.3	16.1	0.6	335
**Wealth index**										
Lowest	2.8	40	*	*	*	*	*	*	*	1
Second	8.2	263	(4.5)	(31.8)	(81.8)	(4.5)	(0.0)	(13.6)	(0.0)	22
Middle	8.6	923	3.5	26.7	77.1	3.4	0.0	7.5	0.0	80
Fourth	11.6	1,555	7.0	39.6	69.8	3.2	0.6	8.3	1.7	183
Highest	18.2	2,250	11.7	31.6	73.9	4.8	0.3	17.4	0.5	412
**Haryana**	13.7	5,031	9.3	33.2	73.3	4.3	0.3	13.7	0.8	698

[#] Total figure may not add to N, sample total due to ‘do not know’ or ‘missing cases’.
nc = Not calculated because there are not cases.
( ) Based on 10-24 unweighted cases.
* Percentage not shown, based on less than 10 unweighted cases.
** Unweighted cases.
[a] Literates but did not attend school, are also included.

TABLE 8.11 KNOWLEDGE OF RTI AND STI BY SOURCES

Percentage of unmarried women aged 15-24 years who are aware of RTI/STI and source of information , according to selected background characteristics, Haryana, 2007-08

Background characteristics	Ever heard of RTI/ STI	Number of women**	Sources of information: Radio	T.V.	Cinema	Print media	Health personnel	Partner	Relatives/ friends	Adult Education programme/ school teacher	Religious/ political leader/ community meetings/ exhibition/ *mela*	Others	Number of women who ever heard of RTI/STI**
**Age group**													
15-19	32.3	3,966	9.3	58.8	1.4	43.6	4.8	0.4	29.1	19.4	0.9	3.2	1,286
20-24	51.7	1,065	12.4	68.3	1.6	55.6	6.0	1.5	28.5	16.9	1.6	4.7	552
**Residence**													
Rural	34.7	3,585	11.7	60.2	1.2	43.4	5.0	0.7	30.1	16.9	1.2	3.4	1,248
Urban	40.5	1,446	7.2	64.8	2.0	55.3	5.5	0.7	26.3	22.3	0.9	4.3	590
**Education**													
Non-literate[a]	11.1	262	2.9	33.9	0.0	0.0	0.0	0.0	72.4	0.0	0.0	0.0	29
Less than five years	9.2	177	(11.8)	(58.8)	(0.0)	(0.0)	(11.8)	(0.0)	(52.9)	(0.0)	(0.0)	(0.0)	17
5-9 years	27.7	1,816	8.8	58.1	0.4	28.6	5.5	0.4	36.7	13.1	0.8	3.5	501
10 or more years	46.4	2,776	11.0	63.8	1.9	56.2	5.1	0.8	24.5	21.5	1.3	3.9	1,291
**Religion**													
Hindu	37.6	4,402	10.0	62.1	1.5	47.5	4.6	0.8	28.1	18.7	0.8	3.5	1,655
Muslim	15.9	222	5.0	43.0	0.0	19.5	5.3	0.0	61.5	5.5	0.0	0.0	36
Sikh	35.6	389	15.0	61.0	1.5	50.5	11.6	0.0	28.7	19.7	4.3	6.4	140
Others	*	4	nc	nc	nc	nc	nc	nc	nc	nc	nc	nc	nc
**Castes/tribes[#]**													
Scheduled castes	30.9	1,246	11.0	61.3	0.2	38.9	5.4	0.2	29.1	20.3	1.7	3.0	386
Scheduled tribes	(33.3)	24	*	*	*	*	*	*	*	*	*	*	8
Other backward classes	34.5	1,647	9.0	62.5	1.7	44.1	6.1	0.4	31.6	20.0	1.2	3.3	572
Others	41.2	2,112	10.8	61.1	1.8	52.9	4.5	1.1	27.0	16.5	0.7	4.1	872
**Wealth index**													
Lowest	17.5	40	*	*	*	*	*	*	*	*	*	*	7
Second	19.3	263	7.4	44.7	0.0	37.0	9.5	2.0	34.8	16.8	3.7	3.4	53
Middle	23.8	923	11.7	50.7	0.4	36.7	2.7	1.1	35.0	15.9	0.9	0.4	220
Fourth	34.3	1,555	12.3	60.5	1.3	40.3	4.7	0.2	30.8	18.5	1.1	3.4	532
Highest	45.5	2,250	9.1	65.9	1.8	53.8	5.8	0.8	25.9	19.5	1.1	4.5	1,026
**Haryana**	36.4	5,031	10.3	61.7	1.4	47.2	5.2	0.7	28.9	18.6	1.1	3.7	1,838

[#] Total figure may not add to N, sample total due to ‘do not know’ or ‘missing cases’.
nc = Not calculated because there are not cases.
( ) Based on 10-24 unweighted cases.
* Percentage not shown, based on less than 10 unweighted cases.
** Unweighted cases.
[a] Literates but did not attend school, are also included.

TABLE 8.12 KNOWLEDGE OF RTI/STI TRANSMISSION

Percentage of unmarried women aged 15-24 years who know about transmission of RTI/STI, according to selected background characteristics, Haryana, 2007-08

Background characteristics	Unsafe delivery	Unsafe abortion	Unsafe IUD insertion	Unsafe sex with homosexual	Unsafe sex with persons who have many partners	Unsafe sex with sex worker	Other	Number of women**
**Age group**								
15-19	8.0	5.5	3.1	12.4	73.9	8.8	13.6	1,286
20-24	9.3	5.1	5.7	12.6	77.6	9.7	14.8	552
**Residence**								
Rural	10.0	4.1	3.4	14.0	72.7	8.3	13.7	1,248
Urban	5.6	7.5	5.3	9.6	79.7	10.6	14.6	590
**Education**								
Non-literate[a]	0.0	0.0	0.0	38.8	61.2	0.0	19.5	29
Less than five years	(0.0)	(0.0)	(0.0)	(0.0)	(60.0)	(20.0)	(20.0)	17
5-9 years	4.7	1.7	1.1	10.3	75.7	8.6	15.7	501
10 or more years	9.6	6.4	4.9	13.0	75.3	9.2	13.4	1,291
**Religion**								
Hindu	6.7	4.5	3.6	12.6	75.7	8.7	14.0	1,655
Muslim	19.6	19.6	0.0	19.6	57.0	19.6	43.0	36
Sikh	26.1	15.4	8.6	10.5	70.9	13.7	11.8	140
Others	nc	nc	nc	nc	nc	nc	nc	nc
**Castes/tribes[#]**								
Scheduled castes	6.0	3.8	1.6	9.0	82.0	6.6	11.0	386
Scheduled tribes	*	*	*	*	*	*	*	8
Other backward classes	10.9	5.5	3.6	14.5	70.6	7.7	15.5	572
Others	7.4	6.0	5.2	13.0	75.1	11.4	13.8	872
**Wealth index**								
Lowest	*	*	*	*	*	*	*	7
Second	14.9	9.7	4.9	4.8	60.0	15.1	15.0	53
Middle	7.9	3.5	1.2	10.5	69.3	6.2	17.0	220
Fourth	8.1	2.4	2.3	12.7	76.2	8.7	13.5	532
Highest	8.4	6.7	5.2	13.0	76.3	9.5	13.7	1,026
**Haryana**	8.4	5.3	4.1	12.5	75.2	9.1	14.0	1,838

[#] Total figure may not add to N, sample total due to 'do not know' or 'missing cases'.
nc = Not calculated because there are no cases.
( ) Based on 10-24 unweighted cases.
* Percentage not shown, based on less than 10 unweighted cases.
** Unweighted cases.
[a] Literates but did not attend school, are also included.

TABLE 8.13 KNOWLEDGE OF HIV/AIDS BY SOURCES

Percentage of unmarried women aged 15-24 years who are aware of HIV/AIDS, according to selected background characteristics, Haryana, 2007-08

Background characteristics	Ever heard of HIV/AIDS	Number of women**	Source of information						Number of women who ever heard of HIV/AIDS**
			Radio	Television	Cinema	Print media	Health personnel	Adult education programme	
**Age group**									
15-19	80.7	3,966	16.8	83.3	1.6	50.8	4.9	0.2	3,230
20-24	94.5	1,065	20.1	92.0	2.1	65.4	7.4	0.6	1,005
**Residence**									
Rural	80.5	3,585	19.8	82.9	1.5	49.6	5.8	0.3	2,922
Urban	91.4	1,446	12.8	90.8	2.1	64.6	4.9	0.5	1,313
**Education**									
Non-literate[a]	19.4	262	10.9	79.1	0.0	0.0	3.6	0.0	55
Less than five years	42.1	177	16.6	76.4	0.0	4.0	6.6	0.0	77
5-9 years	74.8	1,816	16.5	80.7	1.1	33.5	4.6	0.1	1,369
10 or more years	98.5	2,776	18.4	88.1	2.0	67.1	6.0	0.5	2,734
**Religion**									
Hindu	86.3	4,402	17.3	85.2	1.6	54.5	5.1	0.3	3,809
Muslim	25.4	222	12.5	76.1	1.8	39.8	5.1	0.0	61
Sikh	89.3	389	22.8	90.1	2.2	53.6	9.4	0.4	347
Others	*	4	*	*	*	*	*	*	4
**Castes/tribes[#]**									
Scheduled castes	75.6	1,246	17.2	81.8	0.6	42.6	6.0	0.3	944
Scheduled tribes	(91.7)	24	(4.5)	(86.4)	(4.5)	(50.0)	(13.6)	(4.5)	22
Other backward classes	78.7	1,647	17.0	85.9	1.5	53.4	5.9	0.3	1,317
Others	92.1	2,112	18.4	86.8	2.3	60.6	4.9	0.4	1,950
**Wealth index**									
Lowest	16.4	40	*	*	*	*	*	*	7
Second	49.5	263	18.6	63.9	0.0	37.3	6.1	0.0	136
Middle	69.7	923	18.6	72.0	0.2	38.0	5.7	0.3	653
Fourth	81.7	1,555	19.8	83.8	1.4	47.9	4.7	0.4	1,279
Highest	96.0	2,250	16.0	91.7	2.4	64.0	5.9	0.3	2,160
**Haryana**	83.6	5,031	17.6	85.4	1.7	54.3	5.5	0.3	4,235

[#] Total figure may not add to N, sample total due to 'do not know' or 'missing cases'.
( ) Based on 10-24 unweighted cases.
* Percentage not shown, based on less than 10 unweighted cases.
** Unweighted cases.
[a] Literates but did not attend school, are also included.

TABLE 8.14 KNOWLEDGE OF HIV TRANSMISSION /AIDS

Percentage of unmarried women aged 15-24 years who know about transmission of HIV/AIDS, according to selected background characteristics, Haryana, 2007-08

Background characteristics	Unsafe Sex with homosexual	Unsafe sex with persons who have many partners	Unsafe sex with sex worker	Unprotected sex with HIV/AIDS infected person	Infected mother to child	Transfusion of blood	Other	Number of women who ever heard of HIV/AIDS**
**Age group**								
15-19	4.9	71.1	7.0	25.3	18.2	57.3	50.5	3,230
20-24	5.8	76.1	10.7	31.4	30.2	69.9	56.4	1,005
**Residence**								
Rural	4.8	72.6	7.0	25.3	19.3	56.9	51.2	2,922
Urban	5.8	71.9	9.8	30.2	25.4	67.9	53.7	1,313
**Education**								
Non-literate[a]	0.0	66.6	7.0	16.3	0.0	29.6	36.7	55
Less than five years	0.0	70.1	13.2	19.4	2.2	17.8	28.9	77
5-9 years	5.4	68.6	6.6	22.7	10.6	48.4	42.8	1,369
10 or more years	5.2	73.9	8.3	28.7	26.0	66.3	56.2	2,734
**Religion**								
Hindu	4.6	72.8	7.7	26.6	21.5	60.4	52.0	3,809
Muslim	2.2	78.1	6.5	26.8	13.5	65.3	52.7	61
Sikh	10.4	68.0	10.1	29.8	20.2	60.8	52.4	347
Others	*	*	*	*	*	*	*	4
**Castes/tribes[#]**								
Scheduled castes	4.9	70.7	9.6	23.7	16.2	52.5	46.1	944
Scheduled tribes	(25.0)	(62.5)	(18.8)	(43.8)	(37.5)	(62.5)	(37.5)	22
Other backward classes	6.0	71.0	7.3	29.3	21.1	60.5	50.3	1,317
Others	4.5	74.2	7.5	26.5	23.4	64.0	55.8	1,950
**Wealth index**								
Lowest	*	*	*	*	*	*	*	7
Second	7.0	68.2	9.1	17.5	13.1	42.6	43.8	136
Middle	3.8	74.1	7.3	18.8	9.7	47.0	45.4	653
Fourth	4.8	72.2	8.0	24.9	19.7	56.2	50.3	1,279
Highest	5.6	72.3	8.0	30.4	25.5	67.1	55.0	2,160
**Haryana**	5.1	72.4	7.9	26.9	21.3	60.5	52.0	4,235

[#] Total figure may not add to N, sample total due to ‘do not know’ or ‘missing cases’.
( ) Based on 10-24 unweighted cases.
* Percentage not shown, based on less than 10 unweighted cases.
** Unweighted cases.
[a] Literates but did not attend school, are also included.

TABLE 8.15 MISCONCEPTION ABOUT TRANSMISSION OF HIV/AIDS

Percentage of unmarried women aged 15-24 years who have misconception of transmission of HIV/AIDS, according to selected background characteristics, Haryana, 2007-08

Background characteristics	Shaking hands	Hugging	Kissing	Sharing cloths	Sharing food	Stepping on urine/stool of some one who has AIDS	Mosquito/ flea or bedbugs bites	Number of women**
**Age group**								
15-19	10.1	9.7	12.5	13.1	13.2	11.2	20.4	3,230
20-24	4.4	4.2	7.0	6.7	7.2	6.5	11.9	1,005
**Residence**								
Rural	9.6	9.1	12.0	12.5	13.1	11.3	19.9	2,922
Urban	7.0	6.7	9.3	9.4	9.0	7.4	15.1	1,313
**Education**								
Non-literate[a]	20.9	24.4	31.5	33.4	33.1	33.6	44.3	55
Less than five years	13.4	13.8	17.8	18.7	17.0	20.4	36.9	77
5-9 years	14.0	13.2	16.5	17.2	18.0	15.5	24.9	1,369
10 or more years	5.8	5.5	7.9	8.1	8.1	6.7	14.1	2,734
**Religion**								
Hindu	8.9	8.6	11.4	11.9	12.1	10.2	18.3	3,809
Muslim	12.3	12.3	17.2	15.7	13.8	18.2	25.5	61
Sikh	6.4	5.5	7.2	6.9	8.7	7.1	17.7	347
Others	*	*	*	*	*	*	*	4
**Castes/tribes[#]**								
Scheduled castes	8.3	8.4	11.7	12.4	12.9	10.6	20.7	944
Scheduled tribes	(18.2)	(9.1)	(13.6)	(13.6)	(18.2)	(18.2)	(18.2)	22
Other backward classes	9.7	9.1	10.8	11.2	12.1	11.0	19.1	1,317
Others	8.2	7.9	11.1	11.4	11.0	9.1	16.8	1,950
**Wealth index**								
Lowest	*	*	*	*	*	*	*	7
Second	22.2	20.8	25.9	24.5	26.0	20.0	30.3	136
Middle	12.4	12.5	17.2	17.5	18.0	16.5	25.2	653
Fourth	9.1	8.8	10.9	11.7	12.3	10.7	19.7	1,279
Highest	6.7	6.1	8.6	8.9	8.8	7.2	14.8	2,160
**Haryana**	8.8	8.4	11.1	11.6	11.8	10.1	18.4	4,235

[#] Total figure may not add to N, sample total due to ‘do not know’ or ‘missing cases’.
( ) Based on 10-24 unweighted cases.
* Percentage not shown, based on less than 10 unweighted cases.
** Unweighted cases.
[a] Literates but did not attend school, are also included.

TABLE 8.16 KNOWLEDGE ABOUT HOW TO AVOID OR REDUCE THE CHANCES OF INFECTING HIV/AIDS

Percentage of unmarried women aged 15-24 years who know how to avoid or reduce the chances of getting of HIV/AIDS, according to selected background characteristics, Haryana, 2007-08

Background characteristics	Abstain from sex	Using condom correctly during each sexual intercourse	Limit no. of sexual partners and avoid sex with sex workers	Avoid sex with person who inject drugs	Avoid pregnancy when having HIV/ AIDS	Other	Number of women who ever heard of HIV/AIDS**
**Age group**							
15-19	10.4	32.0	55.5	58.0	8.1	1.7	3,230
20-24	13.4	46.7	65.8	73.3	14.2	1.8	1,005
**Residence**							
Rural	10.4	33.7	57.3	58.2	8.6	1.6	2,922
Urban	12.6	40.4	59.4	69.1	11.9	2.1	1,313
**Education**							
Non-literate[a]	7.6	12.3	33.9	20.0	0.0	4.1	55
Less than five years	11.3	9.5	41.2	19.7	2.4	6.9	77
5-9 years	11.3	27.3	47.0	42.1	3.9	2.3	1,369
10 or more years	11.1	39.8	64.3	73.3	12.1	1.4	2,734
**Religion**							
Hindu	11.0	35.8	57.5	61.2	9.7	1.7	3,809
Muslim	6.1	30.1	52.0	52.1	11.3	0.0	61
Sikh	12.4	37.4	64.1	66.5	8.7	2.7	347
Others	*	*	*	*	*	*	4
**Castes/tribes**[#]							
Scheduled castes	10.0	33.0	52.3	51.8	6.9	1.6	944
Scheduled tribes	(25.0)	(43.8)	(40.9)	(54.5)	(25.0)	(6.3)	22
Other backward classes	13.1	38.3	57.6	59.8	9.1	1.9	1,317
Others	10.2	35.6	61.1	67.6	11.1	1.6	1,950
**Wealth index**							
Lowest	*	*	*	*	*	*	7
Second	10.2	23.3	40.9	41.2	4.5	0.0	136
Middle	10.6	24.5	47.8	43.4	5.3	2.3	653
Fourth	11.8	33.4	56.9	57.6	7.5	1.0	1,279
Highest	11.0	40.6	62.7	70.7	12.1	2.1	2,160
**Haryana**	11.1	35.9	58.0	61.6	9.7	1.7	4,235

[#] Total figure may not add to N, sample total due to 'do not know' or 'missing cases'.
( ) Based on 10-24 unweighted cases.
* Percentage not shown, based on less than 10 unweighted cases.
** Unweighted cases.
[a] Literates but did not attend school, are also included.

TABLE 8.17 KNOWLEDGE REGARDING WHERE TO GET TESTED FOR HIV/AIDS AND SOURCES

Percentage of unmarried women aged 15-24 years who are aware where to get tested for HIV/AIDS and place for testing HIV/AIDS, according to selected background characteristics, Haryana, 2007-08

Background characteristics	Know place where to get Tested for HIV/AIDS**	Number of women**	Health Facility							Number of women who Knew place where to get Tested for HIV/AIDS **
			Government				Private			
			Hospital	CHC/ PHC/Sub-Centre	RTI/STI clinic VCT/ICTC	Other public/NGO hospitals	Hospital	RTI/STI VCT/ICTC	Other private hospitals	
**Age group**										
15-19	74.0	3,230	88.7	14.2	0.4	3.6	32.3	0.3	0.3	2,395
20-24	81.8	1,005	90.3	13.5	1.4	4.5	38.7	0.4	0.6	821
**Residence**										
Rural	74.2	2,922	88.8	15.8	0.4	3.7	31.3	0.2	0.1	2,173
Urban	79.3	1,313	89.8	10.4	1.2	4.2	39.6	0.6	0.7	1,043
**Education**										
Non-literate[a]	64.5	55	77.1	17.1	0.0	0.0	28.2	0.0	0.0	36
Less than five years	60.9	77	92.7	8.1	0.0	6.4	30.2	0.0	0.0	47
5-9 years	67.1	1,369	86.6	17.7	0.1	3.6	28.1	0.1	0.2	921
10 or more years	80.8	2,734	90.2	12.6	0.9	4.0	36.6	0.4	0.4	2,212
**Religion**										
Hindu	75.6	3,809	89.4	14.1	0.6	3.8	33.7	0.3	0.4	2,886
Muslim	80.7	61	87.0	11.1	0.0	2.2	42.0	2.2	0.0	48
Sikh	76.5	347	85.4	14.7	1.5	4.4	36.0	0.0	0.0	266
Others	*	4	*	*	*	*	*	*	*	4
**Castes/tribes[#]**										
Scheduled castes	73.1	944	88.6	16.9	0.1	3.3	28.3	0.1	0.5	693
Scheduled tribes	(77.3)	22	(94.1)	(5.9)	(0.0)	(5.9)	(23.5)	(0.0)	(0.0)	17
Other backward classes	74.3	1,317	87.7	15.0	0.2	3.9	33.6	0.2	0.3	978
Others	78.3	1,950	90.1	12.2	1.1	4.1	36.9	0.5	0.3	1,528
**Wealth index**										
Lowest	*	7	*	*	*	*	*	*	*	6
Second	70.8	136	83.8	19.5	0.0	4.2	32.0	0.0	0.0	96
Middle	71.3	653	87.2	19.5	0.2	2.1	27.3	0.0	0.2	467
Fourth	71.3	1,279	87.8	15.5	0.2	3.9	29.2	0.3	0.1	915
Highest	80.1	2,160	90.6	11.5	1.0	4.3	38.4	0.4	0.5	1,732
**Haryana**	75.9	4,235	89.1	14.0	0.6	3.8	34.0	0.3	0.3	3,216

[#] Total figure may not add to N, sample total due to ‘do not know’ or ‘missing cases’.
( ) Based on 10-24 unweighted cases.
* Percentage not shown, based on less than 10 unweighted cases.
** Unweighted cases.
[a] Literates but did not attend school, are also included.

TABLE 8.18 KNOWLEDGE OF SOME SELECTED STATEMENTS

Percentage of unmarried women aged 15-24 years by awareness on reproductive issues, according to selected background characteristics, Haryana, 2007-08

	Selected statements to test the level of awareness										
	It is possible to know the sex of the baby before the baby is born by medical test		Pregnancy can occur after kissing or hugging		When a woman has intercourse for the first time she has to bleed		A women can get pregnant on the very first time she has sexual intercourse		A woman is most likely to get pregnant if she has sexual intercourse half way between her menstrual periods		
Background characteristics	True	Don't know	True	Don't know	True	Don't know	True	Don't know	True	Don't know	Number of women**
**Age group**											
15-19	69.7	9.1	3.3	20.5	15.8	67.5	20.7	61.9	16.7	73.6	3,966
20-24	73.6	3.0	3.0	8.7	20.1	56.6	27.3	52.8	25.6	61.5	1,065
**Residence**											
Rural	69.7	9.0	3.1	19.6	16.7	67.0	21.7	61.1	18.2	72.5	3,585
Urban	72.6	5.0	3.6	14.2	16.8	60.6	23.2	57.2	19.7	67.6	1,446
**Education**											
Non-literate[a]	52.5	33.9	4.1	39.1	12.2	79.2	11.4	77.8	6.9	85.9	262
Less than five years	57.2	24.1	3.5	36.8	14.3	77.6	15.6	70.8	8.9	86.9	177
5-9 years	69.7	10.0	3.7	23.3	16.2	67.4	19.4	62.6	15.8	73.8	1,816
10 or more years	73.8	2.7	2.9	11.2	17.7	61.5	25.4	55.8	22.3	66.7	2,776
**Religion**											
Hindu	71.3	6.8	3.2	17.4	16.4	65.0	22.7	59.0	18.7	70.5	4,402
Muslim	60.5	28.5	6.6	38.3	11.8	79.0	12.4	79.4	10.9	83.7	222
Sikh	68.9	6.0	2.1	13.1	22.9	58.9	21.3	59.3	21.8	70.5	389
Others	*	*	*	*	*	*	*	*	*	*	4
**Castes/tribes[#]**											
Scheduled castes	65.7	9.8	2.8	21.9	16.4	66.9	20.0	63.2	17.9	72.8	1,246
Scheduled tribes	(62.5)	(8.3)	12.5)	(33.3)	(33.3)	(50.0)	(33.3)	(54.2)	(25.0)	(70.8)	24
Other backward classes	70.3	10.2	3.5	19.5	16.7	66.9	21.9	61.3	18.9	72.0	1,647
Others	73.6	4.7	3.3	14.5	16.8	63.0	23.5	57.1	18.7	69.3	2,112
**Wealth index**											
Lowest	55.1	22.1	0.0	41.8	2.1	75.7	9.3	79.0	12.1	83.4	40
Second	60.8	20.1	3.8	30.0	15.0	73.6	17.0	68.4	17.5	72.0	263
Middle	68.5	12.5	3.8	26.3	15.0	71.6	19.0	65.9	14.1	77.8	923
Fourth	68.6	8.6	3.3	20.1	16.8	66.7	21.2	61.1	17.3	73.1	1,555
Highest	74.1	3.6	3.0	11.3	17.9	60.3	24.9	55.4	21.6	66.6	2,250
**Haryana**	70.5	7.8	3.3	18.0	16.7	65.2	22.1	60.0	18.6	71.1	5,031

[#] Total figure may not add to N, sample total due to 'do not know' or 'missing cases'.
( ) Based on 10-24 unweighted cases.
* Percentage not shown, based on less than 10 unweighted cases.
** Unweighted cases.
[a] Literates but did not attend school, are also included.

TABLE 8.19 AWARENESS OF REPRODUCTIVE HEALTH ISSUES

Percentage of unmarried women aged 15-24 years who perceived family life education is important, having menstruation related problems, are aware of specific contraceptive methods and who have ever discussed about family planning with any one by district, Haryana, 2007-08

District	Perceived family life education to be important	Having menstruation related problems during last three months	Knowledge of contraceptive pills	Knowledge of emergency contraception	Ever discussed about family planning with any one	Number of women**
Panchkula	95.3	21.6	93.3	50.8	16.9	255
Ambala	84.7	17.6	82.8	32.7	12.6	336
Yamuna Nagar	91.5	22.1	90.9	44.2	13.2	349
Kurukshetra	88.4	17.8	89.5	34.4	16.6	276
Kaithal	90.0	15.1	90.0	24.6	11.5	260
Karnal	92.3	20.1	93.0	37.1	9.0	299
Panipat	90.0	18.3	92.5	34.0	12.4	241
Sonipat	90.8	13.9	90.8	44.6	7.6	250
Jind	94.0	9.0	80.2	25.7	13.8	267
Fatehabad	90.4	12.2	74.6	16.8	9.3	280
Sirsa	89.2	9.5	84.7	23.3	19.0	295
Hisar	84.1	12.7	79.9	21.0	9.3	214
Bhiwani	90.7	10.7	78.5	32.0	11.7	209
Rohtak	94.7	4.8	95.6	42.1	18.4	228
Jhajjar	89.5	18.2	89.5	26.9	14.4	200
Mahendragarh	93.7	23.9	82.6	21.2	19.5	190
Rewari	96.0	19.9	96.8	34.3	19.4	248
Gurgaon	92.5	11.0	88.7	25.8	15.1	236
Faridabad	81.3	13.4	79.9	27.9	8.8	204
Mewat	71.1	17.8	63.4	6.7	10.8	194
**Haryana**	89.5	15.8	86.0	30.2	13.8	5,031

** Unweighted cases.

HEALTH FACILITIES - AVAILABILITY AND QUALITY

TABLE 9.1: AVERAGE POPULATION COVERED BY HEALTH FACILITY BY DISTRICTS, HARYANA, 2007-08

District	Average population covered by		
	Sub-Centre	PHC	CHC
Panchkula	6,570	34,595	1,62,673
Ambala	7,208	47,742	--
Yamunanagar	7,312	40,046	1,29,136
Kurukshetra	6,478	43,451	1,43,719
Kaithal	6,410	38,369	--
Karnal	7,689	47,933	2,20,523
Panipat	9,100	47,442	2,52,167
Sonipat	6,239	40,993	1,74,923
Jind	9,794	37,549	1,35,312
Fatehabad	7,687	38,626	1,74,891
Sirsa	6,699	36,504	1,48,852
Hisar	7,865	36,283	1,38,755
Bhiwani	6,692	35,128	--
Rohtak	5,925	29,177	1,37,271
Jhajjar	6,976	35,205	1,63,043
Mahendragarh	7,480	39,255	1,19,331
Rewari	7,924	39,320	1,49,688
Gurgaon	9,310	61,791	2,51,050
Faridabad	7,658	46,166	2,24,391
Mewat	10,395	64,096	2,63,275
**Haryana**	7,585	41,500	1,67,422

PHC= Primary Health Centre; CHC= Community Health Centre.

TABLE 9.2: PERCENTAGE OF VILLAGES HAVING SUB-CENTRES WITHIN VILLAGES & ANM AVAILABLE AT SUB-CENTRE AND STAYING IN SUB-CENTRE QUARTER BY DISTRICTS, HARYANA, 2007-08

District	Villages having Sub-Centre within village	No. of villages	ANM/FHW Available at Sub-Centre	ANM residing in Sub-Centre quarter[1]	Total number of Sub-Centres
Panchkula	21.4	28	95.5	42.9	22
Ambala	31.3	32	100.0	30.0	23
Yamunanagar	9.7	31	84.0	66.7	25
Kurukshetra	43.2	37	96.9	0.0	32
Kaithal	60.0	40	74.3	20.0	35
Karnal	32.4	37	93.9	37.5	33
Panipat	50.0	30	96.6	0.0	29
Sonipat	62.2	37	100.0	10.0	35
Jind	57.5	40	100.0	25.0	33
Fatehabad	68.3	41	86.1	33.3	36
Sirsa	48.6	37	93.9	53.3	33
Hisar	81.1	37	91.4	63.6	35
Bhiwani	56.1	41	97.4	42.9	39
Rohtak	78.1	32	100.0	0.0	32
Jhajjar	71.8	39	96.9	100.0	32
Mahendragarh	30.2	43	91.4	24.0	35
Rewari	41.5	41	100.0	22.2	34
Gurgaon	25.0	32	100.0	40.0	25
Faridabad	25.0	20	88.9	100.0	18
Mewat	19.6	46	66.7	0.0	39
**Haryana**	46.6	721	92.3	31.4	625

ANM= Auxiliary Nurse Midwife; FHW=Female Health Worker.
[1] Based on availability of quarter at Sub-Centre.

TABLE 9.3: STATUS OF INFRASTRUCTURE AT SUB-CENTRE FUNCTIONING IN GOVERNMENT BUILDING BY DISTRICTS, HARYANA, 2007-08

District	Number of Sub-Centres					Total number of Sub-Centres
	Regular Electricity	Water	Toilet	Labor room	Labor room in current use[1]	
Panchkula	2	9	7	9	6	11
Ambala	2	9	6	7	5	12
Yamunanagar	2	12	10	11	9	16
Kurukshetra	0	7	6	7	6	12
Kaithal	2	11	11	9	8	11
Karnal	8	16	15	15	12	18
Panipat	3	13	10	9	7	17
Sonipat	1	13	6	9	8	18
Jind	6	14	7	5	4	17
Fatehabad	2	15	13	14	12	16
Sirsa	13	29	27	29	29	30
Hisar	3	17	14	15	15	18
Bhiwani	5	16	10	5	3	24
Rohtak	2	9	11	14	13	22
Jhajjar	4	9	13	9	7	18
Mahendragarh	4	26	16	15	12	28
Rewari	5	16	9	9	7	19
Gurgaon	1	5	7	9	8	11
Faridabad	3	7	9	2	2	12
Mewat	1	4	4	2	1	13
**Haryana**	69	257	211	204	174	343
**(%)**	(20.1)	(74.9)	(61.5)	(59.5)	(85.3)	(100.0)

[1] Percentage calculated from number of labor room available.

TABLE 9.4: NUMBER OF SUB-CENTRES HAVING ADEQUATELY EQUIPPED AND ESSENTIAL DRUGS BY DISTRICTS, HARYANA, 2007-08

District	Adequately equipped[1] (at least 60%)	Essential drugs[2] (at least 60%)	Total number of Sub-Centres
Panchkula	22	7	22
Ambala	23	17	23
Yamunanagar	24	13	25
Kurukshetra	32	11	32
Kaithal	32	12	35
Karnal	32	11	33
Panipat	28	2	29
Sonipat	35	5	35
Jind	32	0	33
Fatehabad	36	7	36
Sirsa	33	18	33
Hisar	32	22	35
Bhiwani	35	9	39
Rohtak	32	7	32
Jhajjar	26	13	32
Mahendragarh	34	5	35
Rewari	34	5	34
Gurgaon	25	0	25
Faridabad	16	10	18
Mewat	31	8	39
**Haryana**	594	182	625
**(%)**	(95.0)	(29.1)	(100.0)

[1] Equipments include Instrument sterilizer, Auto disposal syringes, Hub cutter, B. P. instrument, Stethoscope, Weighing Machine (infant/adult), hemoglobinometer, Foetoscope, SIMS speculam, IUD insertion kit, Vaccine carrier.
[2] Drugs includes, Drug kit-A/B, IFA tablets, Vitamine A solution, ORS packet.

TABLE 9.5: NUMBER OF SUB-CENTRES HAVING DIFFERENT ACTIVITIES BY DISTRICTS, HARYANA, 2007-08

District	Citizen's Charter displayed	VHSC Facilitated*	Untied Fund Received	Total number of Sub-Centres
Panchkula	13	20	21	22
Ambala	13	20	22	23
Yamunanagar	18	22	23	25
Kurukshetra	5	28	32	32
Kaithal	12	31	33	35
Karnal	16	28	31	33
Panipat	10	22	26	29
Sonipat	10	34	35	35
Jind	8	27	31	33
Fatehabad	17	25	35	36
Sirsa	24	33	33	33
Hisar	23	31	34	35
Bhiwani	23	32	39	39
Rohtak	23	23	31	32
Jhajjar	20	32	31	32
Mahendragarh	13	30	29	35
Rewari	12	33	34	34
Gurgaon	17	17	25	25
Faridabad	10	18	18	18
Mewat	3	31	31	39
**Haryana**	290	537	594	625
**(%)**	(46.4)	(91.2)	(95.0)	(100.0)

VHSC= Village Health and Sanitation Committee.
*Based on availability of VHSC.

TABLE 9.6: AVAILABLE HUMAN RESOURCES AT PRIMARY HEALTH CENTRES BY DISTRICTS, HARYANA, 2007-08

District	Human resources Status of PHCs				Total number of PHCs
	Medical officer	Lady Medical Officer	AYUSH Doctor	Pharmacist	
Panchkula	8	9	1	9	9
Ambala	12	5	1	11	12
Yamunanagar	12	5	0	12	13
Kurukshetra	11	4	0	10	11
Kaithal	11	6	0	16	16
Karnal	13	10	1	16	17
Panipat	9	4	0	13	13
Sonipat	19	7	0	20	21
Jind	1	1	1	13	13
Fatehabad	6	4	0	11	11
Sirsa	12	0	0	10	14
Hisar	14	6	0	17	17
Bhiwani	12	2	0	16	18
Rohtak	11	8	0	12	12
Jhajjar	13	1	0	13	13
Mahendragarh	7	2	0	11	12
Rewari	11	1	0	10	11
Gurgaon	7	4	0	9	9
Faridabad	7	1	0	9	9
Mewat	6	1	0	8	12
**Haryana**	202	81	4	246	263
**(%)**	(76.8)	(30.8)	(1.5)	(93.5)	(100.0)

LMO= Lady Medical Officer.
AYUSH = Ayurveda, Yoga and Naturopathy, Unani, Siddha and Homeopathy.

TABLE 9.7: AVAILABLE INFRASTRUCTURE AT PRIMARY HEALTH CENTRES BY DISTRICTS, HARYANA, 2007-08

Districts	Number of PHCs having					Total number of PHCs
	Residential Quarter for MO	Functioning 24 hours basis	At least 4 beds	Regular power supply	Having functional vehicle	
Panchkula	4	5	6	7	4	9
Ambala	7	7	9	7	1	12
Yamunanagar	5	5	6	5	3	13
Kurukshetra	3	6	7	4	1	11
Kaithal	5	2	7	8	0	16
Karnal	11	9	17	8	2	17
Panipat	5	4	6	5	3	13
Sonipat	6	5	8	11	2	21
Jind	3	3	7	8	5	13
Fatehabad	3	5	5	2	0	11
Sirsa	11	11	13	10	1	14
Hisar	12	12	15	1	2	17
Bhiwani	11	11	13	14	5	18
Rohtak	5	5	8	1	0	12
Jhajjar	4	2	12	4	2	13
Mahendragarh	4	3	8	4	3	12
Rewari	4	2	6	5	0	11
Gurgaon	1	5	5	2	0	9
Faridabad	4	1	5	2	2	9
Mewat	5	0	6	2	1	12
**Haryana**	113	103	169	110	37	263
**(%)**	(43.0)	(38.3)	(64.3)	(41.8)	(14.1)	(100.0)

MO= Medical Officer.

TABLE 9.8 SPECIFIC HEALTH FACILITIES AVAILABLE AT PRIMARY HEALTH CENTRES BY DISTRICTS, HARYANA, 2007-08

District	Number of PHCs having						Total number of PHCs
	New born care equipments	Functional OT	Cold chain equipments at least 60 %)[1]	Essential drugs2 (at least 60%)	Referral services for delivery*	Conducted at least 10 deliveries*	
Panchkula	5	6	8	9	2	3	9
Ambala	6	9	12	12	4	2	12
Yamunanagar	7	9	11	11	5	1	13
Kurukshetra	2	4	11	10	5	4	11
Kaithal	4	7	9	14	1	1	16
Karnal	7	12	15	17	5	6	17
Panipat	7	8	7	10	2	4	13
Sonipat	6	10	14	20	2	4	21
Jind	1	4	5	8	1	1	13
Fatehabad	1	7	5	7	2	1	11
Sirsa	5	14	10	12	4	3	14
Hisar	0	14	10	16	5	4	17
Bhiwani	2	9	8	17	5	4	18
Rohtak	6	8	10	12	1	1	12
Jhajjar	0	11	8	12	1	0	13
Mahendragarh	1	5	6	5	1	0	12
Rewari	0	6	7	9	0	0	11
Gurgaon	4	6	9	8	2	1	9
Faridabad	0	7	4	7	0	0	9
Mewat	1	3	5	7	nc	nc	12
**Haryana**	65	159	174	223	48	40	263
**(%)**	(24.7)	(60.5)	(66.2)	(84.8)	(46.6)	(38.8)	(100.0)

* Services based on during last one month and PHCs functioning on 24 hour basis.
[1] Ice lined refrigerator (large/small), Deep freezer (large/ small), Cold Box, Vaccine carrier.
[2] Essential Drugs includes, Antiallergics, Anti hypertensive, Anti Diabetics, Anti anginal, Anti tubercular, Anti leprosy, Anti Filariasis, Anti bactirials, Anti Helminthic, Anti protozoal, Antidots, Solution correcting water and electrolyte imbalance, Essentials obstetric care drugs. OT= Operation Theatre.

TABLE 9.9: NUMBER OF PRIMARY HEALTH CENTRES HAVING DIFFERENT ACTIVITIES BY DISTRICTS, HARYANA, 2007-08

District	Number of PHCs				Total number of PHCs
	Citizen's charter displayed	RKS constituted	Received untied fund	Utilized untied fund	
Panchkula	8	8	7	7	9
Ambala	11	10	12	12	12
Yamunanagar	11	10	12	12	13
Kurukshetra	3	6	11	11	11
Kaithal	9	10	12	12	16
Karnal	11	10	16	16	17
Panipat	10	10	12	12	13
Sonipat	13	20	21	21	21
Jind	4	13	12	11	13
Fatehabad	6	7	10	8	11
Sirsa	10	12	14	14	14
Hisar	14	15	16	15	17
Bhiwani	13	4	16	15	18
Rohtak	9	10	11	11	12
Jhajjar	11	11	11	11	13
Mahendragarh	8	12	10	9	12
Rewari	4	9	9	9	11
Gurgaon	7	8	9	9	9
Faridabad	6	4	7	7	9
Mewat	4	9	8	8	12
**Haryana**	172	198	236	230	263
**(%)**	(65.4)	(75.3)	(89.7)	(87.5)	(100.0)

RKS = Rogi Kalyan Samiti.

TABLE 9.10: HUMAN RESOURCES AVAILABLE AT COMMUNITY HEALTH CENTRES BY DISTRICTS, HARYANA, 2007-08

District	Number of CHCs having:				Total number of CHCs
	Obstetric Gynecologist	Pediatrician	Anesthetist	Health Manager	
Panchkula	0	1	0	0	2
Ambala	nc	nc	nc	nc	0
Yamunanagar	2	1	2	3	6
Kurukshetra	0	1	1	1	4
Kaithal	nc	nc	nc	nc	0
Karnal	1	2	0	1	5
Panipat	0	0	0	0	3
Sonipat	1	0	2	3	6
Jind	2	1	0	0	8
Fatehabad	0	1	0	0	4
Sirsa	0	0	3	0	7
Hisar	3	0	1	0	8
Bhiwani	nc	nc	nc	nc	0
Rohtak	0	2	0	0	5
Jhajjar	0	1	0	0	4
Mahendragarh	1	1	0	0	6
Rewari	0	0	0	0	5
Gurgaon	0	0	0	0	3
Faridabad	0	0	0	0	3
Mewat	1	0	0	0	5
**Haryana**	11	11	9	8	84
**(%)**	(13.1)	(13.1)	(10.7)	(9.5)	(100.0)

nc = Not calculated because there are no cases.

TABLE 9.11: SPECIFIC HEALTH CARE FACILITIES AVAILABLE AT COMMUNITY HEALTH CENTRES BY DISTRICTS, HARYANA, 2007-08

District	Number of CHCs having:					Total number of CHCs
	Functional OT	Designated as FRU	New born care services[1]	LBW babies managed[2]	Blood storage facility[1]	
Panchkula	1	2	1	1	1	2
Ambala	nc	nc	nc	nc	nc	0
Yamunanagar	4	0	nc	3	nc	6
Kurukshetra	2	1	0	2	0	4
Kaithal	nc	nc	nc	nc	nc	0
Karnal	5	4	3	3	1	5
Panipat	0	1	1	2	0	3
Sonipat	4	3	2	2	0	6
Jind	4	4	2	1	2	8
Fatehabad	4	3	2	1	0	4
Sirsa	6	2	2	0	1	7
Hisar	5	4	3	1	1	8
Bhiwani	nc	nc	nc	nc	nc	0
Rohtak	4	3	2	0	0	5
Jhajjar	2	1	1	1	0	4
Mahendragarh	4	3	2	0	1	6
Rewari	1	2	2	0	0	5
Gurgaon	2	0	nc	0	nc	3
Faridabad	0	1	0	1	0	3
Mewat	3	3	0	0	0	5
**Haryana**	51	37	23	18	7	84
**(%)**	(60.7)	(44.1)	(62.2)	(21.4)	(18.9)	(100.0)

LBW=low birth weight. ; OT= Operation Theatre; FRU= First Referral Unit.
[1] Based on 24 hours and CHCs designated as First Referral Unit.
[2] Based on last one month services. nc = Not calculated because there are no cases.

TABLE 9.12: NUMBER OF COMMUNITY HEALTH CENTRES HAVING DIFFERENT ACTIVITIES BY DISTRICTS, HARYANA, 2007-08

District	Number of CHCs having:				Total number of CHCs
	Citizen's charter displayed	RKS constituted	RKS Monitored regularly*	Utilized untied fund	
Panchkula	2	2	2	1	2
Ambala	nc	nc	nc	nc	0
Yamunanagar	5	5	5	6	6
Kurukshetra	4	1	1	3	4
Kaithal	nc	nc	nc	nc	0
Karnal	4	2	2	4	5
Panipat	2	3	2	2	3
Sonipat	5	6	6	6	6
Jind	7	8	7	8	8
Fatehabad	3	3	3	4	4
Sirsa	7	7	7	7	7
Hisar	6	8	8	7	8
Bhiwani	nc	nc	nc	nc	0
Rohtak	4	5	5	5	5
Jhajjar	3	3	3	4	4
Mahendragarh	4	6	6	6	6
Rewari	4	5	5	5	5
Gurgaon	3	2	2	3	3
Faridabad	3	2	2	3	3
Mewat	3	5	5	5	5
**Haryana**	69	73	71	79	84
**(%)**	(82.1)	(86.9)	(97.3)	(94.1)	(100.0)

RKS= Rogi Kalyan Samiti. * RKS monitored regularly is from number of RKS constituted.
nc = Not calculated because there are no cases.

APPENDIX

SAMPLING ERROR FOR SELECTED INDICATORS

ESTIMATES OF SAMPLING ERRORS

Sampling Errors, Haryana, 2007-08

District	Estimate (R)	Sampling Error (SE)	Number of Cases: Unweighted	Number of Cases: Weighted	Relative Error (%)	95% Conf. Interval: R-1.96 SE	95% Conf. Interval: R+1.96 SE
**Contraceptive Prevalence Rate (Currently Married Women age 15-44)**							
Panchkula	0.667	0.021	909	914	3.164	0.626	0.709
Ambala	0.654	0.013	946	941	1.939	0.629	0.679
Yamunanagar	0.638	0.022	1,019	1,026	3.394	0.596	0.681
Kurukshetra	0.656	0.016	949	948	2.505	0.623	0.688
Kaithal	0.670	0.017	1,039	1,036	2.579	0.636	0.704
Karnal	0.643	0.021	971	974	3.312	0.601	0.684
Panipat	0.591	0.022	980	988	3.789	0.547	0.635
Sonipat	0.608	0.021	998	1,001	3.532	0.566	0.651
Jind	0.662	0.016	1,008	1,016	2.397	0.631	0.693
Fatehabad	0.673	0.021	1,000	986	3.082	0.632	0.713
Sirsa	0.694	0.016	1,040	1,033	2.357	0.662	0.726
Hisar	0.676	0.019	950	943	2.848	0.638	0.714
Bhiwani	0.683	0.019	1,048	1,039	2.768	0.646	0.721
Rohtak	0.668	0.020	981	987	3.008	0.629	0.707
Jhajjar	0.615	0.021	964	965	3.401	0.574	0.656
Mahendragarh	0.713	0.019	1,057	1,062	2.662	0.676	0.750
Rewari	0.698	0.017	1,076	1,076	2.376	0.666	0.731
Gurgaon	0.650	0.018	1,212	1,215	2.836	0.614	0.686
Faridabad	0.544	0.025	960	963	4.572	0.495	0.593
Mewat	0.279	0.036	1,287	1,284	12.717	0.210	0.349
**Unmet Need for family planning (Currently Married Women age 15-44)**							
Panchkula	0.120	0.020	909	914	16.939	0.080	0.160
Ambala	0.147	0.011	946	941	7.288	0.126	0.168
Yamunanagar	0.161	0.013	1,019	1,026	8.399	0.134	0.187
Kurukshetra	0.123	0.013	949	948	10.264	0.098	0.148
Kaithal	0.118	0.014	1,039	1,036	11.497	0.091	0.145
Karnal	0.160	0.015	971	974	9.195	0.131	0.189
Panipat	0.164	0.014	980	988	8.728	0.136	0.193
Sonipat	0.156	0.015	998	1,001	9.692	0.127	0.186
Jind	0.138	0.014	1,008	1,016	10.252	0.111	0.166
Fatehabad	0.110	0.012	1,000	986	10.822	0.087	0.133
Sirsa	0.109	0.011	1,040	1,033	10.153	0.087	0.130
Hisar	0.135	0.011	950	943	7.804	0.114	0.155
Bhiwani	0.144	0.014	1,048	1,039	9.516	0.117	0.170
Rohtak	0.120	0.013	981	987	10.592	0.095	0.144
Jhajjar	0.180	0.016	964	965	8.964	0.148	0.212
Mahendragarh	0.129	0.014	1,057	1,062	10.737	0.102	0.156
Rewari	0.128	0.010	1,076	1,076	7.622	0.109	0.148
Gurgaon	0.138	0.013	1,212	1,215	9.689	0.111	0.164
Faridabad	0.213	0.019	960	963	8.717	0.176	0.249
Mewat	0.352	0.023	1,287	1,284	6.499	0.307	0.396

Contd….

Sampling Errors, Haryana, 2007-08—*Continued*							
			Number of Cases			95% Conf. Interval	
District	Estimate (R)	Sampling Error (SE)	Unweighted	Weighted	Relative Error (%)	R-1.96 SE	R+1.96 SE
**Institutional Delivery (last live/still birth of past 3 years)**							
Panchkula	0.646	0.059	296	325	9.100	0.531	0.762
Ambala	0.551	0.043	260	239	7.833	0.466	0.636
Yamunanagar	0.521	0.050	296	300	9.566	0.423	0.619
Kurukshetra	0.642	0.032	288	299	5.013	0.579	0.706
Kaithal	0.476	0.057	342	362	11.907	0.365	0.587
Karnal	0.521	0.052	333	336	9.993	0.419	0.623
Panipat	0.390	0.041	368	352	10.480	0.310	0.470
Sonipat	0.542	0.045	331	331	8.366	0.453	0.632
Jind	0.427	0.047	312	305	10.965	0.335	0.519
Fatehabad	0.488	0.038	338	326	7.840	0.413	0.563
Sirsa	0.536	0.047	277	270	8.853	0.443	0.629
Hisar	0.483	0.045	287	289	9.289	0.395	0.571
Bhiwani	0.354	0.048	331	346	13.506	0.260	0.447
Rohtak	0.530	0.041	306	313	7.724	0.450	0.611
Jhajjar	0.477	0.043	298	301	8.969	0.393	0.561
Mahendragarh	0.567	0.035	286	292	6.122	0.499	0.635
Rewari	0.649	0.042	300	293	6.438	0.567	0.731
Gurgaon	0.523	0.042	434	435	7.960	0.441	0.604
Faridabad	0.391	0.042	379	384	10.863	0.308	0.474
Mewat	0.149	0.020	722	725	13.254	0.110	0.188
**Received Any Antenatal Check up (last live/still birth of past 3 years)**							
Panchkula	0.919	0.043	296	325	4.705	0.834	1.004
Ambala	0.843	0.030	260	239	3.601	0.783	0.902
Yamunanagar	0.908	0.026	296	300	2.818	0.858	0.958
Kurukshetra	0.920	0.023	288	299	2.464	0.875	0.964
Kaithal	0.951	0.013	342	362	1.322	0.927	0.976
Karnal	0.903	0.026	333	336	2.827	0.853	0.953
Panipat	0.872	0.031	368	352	3.602	0.810	0.934
Sonipat	0.955	0.013	331	331	1.376	0.929	0.980
Jind	0.932	0.017	312	305	1.817	0.899	0.965
Fatehabad	0.910	0.022	338	326	2.468	0.866	0.954
Sirsa	0.928	0.017	277	270	1.814	0.895	0.961
Hisar	0.929	0.019	287	289	2.002	0.892	0.965
Bhiwani	0.910	0.030	331	346	3.308	0.851	0.969
Rohtak	0.966	0.011	306	313	1.162	0.944	0.988
Jhajjar	0.921	0.022	298	301	2.350	0.878	0.963
Mahendragarh	0.952	0.016	286	292	1.694	0.920	0.983
Rewari	0.962	0.015	300	293	1.533	0.933	0.991
Gurgaon	0.941	0.021	434	435	2.236	0.899	0.982
Faridabad	0.815	0.037	379	384	4.559	0.743	0.888
Mewat	0.542	0.038	722	725	6.981	0.468	0.616

Contd.…

Sampling Errors, Haryana, 2007-08 —*Continued*							
			Number of Cases			95% Conf. Interval	
District	Estimate (R)	Sampling Error (SE)	Unweighted	Weighted	Relative Error (%)	R-1.96 SE	R+1.96 SE
**Received 3+ Antenatal Check up (last live/still birth of past 3 years)**							
Panchkula	0.728	0.077	296	325	10.570	0.577	0.879
Ambala	0.557	0.043	260	239	7.766	0.472	0.642
Yamunanagar	0.654	0.048	296	300	7.355	0.560	0.748
Kurukshetra	0.614	0.039	288	299	6.269	0.539	0.690
Kaithal	0.645	0.036	342	362	5.639	0.574	0.717
Karnal	0.627	0.047	333	336	7.538	0.534	0.720
Panipat	0.515	0.035	368	352	6.735	0.447	0.583
Sonipat	0.610	0.040	331	331	6.562	0.531	0.688
Jind	0.587	0.037	312	305	6.341	0.514	0.659
Fatehabad	0.430	0.041	338	326	9.493	0.350	0.510
Sirsa	0.544	0.034	277	270	6.195	0.478	0.610
Hisar	0.461	0.039	287	289	8.483	0.385	0.538
Bhiwani	0.485	0.046	331	346	9.417	0.395	0.575
Rohtak	0.613	0.051	306	313	8.254	0.514	0.712
Jhajjar	0.642	0.042	298	301	6.570	0.559	0.725
Mahendragarh	0.560	0.038	286	292	6.870	0.485	0.636
Rewari	0.696	0.034	300	293	4.895	0.629	0.763
Gurgaon	0.613	0.046	434	435	7.548	0.522	0.703
Faridabad	0.460	0.044	379	384	9.511	0.374	0.546
Mewat	0.171	0.025	722	725	14.348	0.123	0.220
**Birth order 2+ (birth in last three years)**							
Panchkula	0.379	0.033	296	315	8.828	0.314	0.445
Ambala	0.346	0.037	226	212	10.652	0.274	0.419
Yamunanagar	0.392	0.056	289	283	14.307	0.282	0.502
Kurukshetra	0.377	0.043	282	293	11.485	0.292	0.462
Kaithal	0.467	0.035	336	353	7.556	0.397	0.536
Karnal	0.433	0.035	332	333	8.088	0.364	0.502
Panipat	0.514	0.033	360	341	6.450	0.449	0.579
Sonipat	0.415	0.034	307	306	8.196	0.349	0.482
Jind	0.550	0.033	302	302	6.015	0.485	0.614
Fatehabad	0.457	0.030	320	312	6.547	0.398	0.516
Sirsa	0.420	0.035	287	281	8.335	0.351	0.488
Hisar	0.487	0.035	265	270	7.241	0.418	0.557
Bhiwani	0.482	0.026	318	334	5.309	0.432	0.533
Rohtak	0.478	0.027	317	323	5.555	0.426	0.530
Jhajjar	0.435	0.039	271	270	9.066	0.358	0.513
Mahendragarh	0.486	0.029	273	279	5.970	0.429	0.543
Rewari	0.473	0.034	308	300	7.107	0.407	0.539
Gurgaon	0.483	0.026	431	437	5.463	0.431	0.535
Faridabad	0.520	0.030	380	377	5.781	0.461	0.579
Mewat	0.633	0.021	826	838	3.331	0.592	0.675

Contd.…

Sampling Errors, Haryana, 2007-08 —*Continued*							
			Number of Cases			95% Conf. Interval	
District	Estimate (R)	Sampling Error (SE)	Unweighted	Weighted	Relative Error (%)	R-1.96 SE	R+1.96 SE
**Received BCG Vaccination (last and last but one living children (age 12-35 months)**							
Panchkula	0.948	0.028	99	105	2.922	0.894	1.003
Ambala	0.952	0.042	73	66	4.446	0.869	1.035
Yamunanagar	0.977	0.023	101	110	2.323	0.932	1.022
Kurukshetra	0.937	0.022	107	115	2.348	0.894	0.981
Kaithal	0.917	0.028	113	120	3.027	0.863	0.972
Karnal	0.981	0.014	110	112	1.446	0.953	1.009
Panipat	0.838	0.056	114	100	6.634	0.729	0.947
Sonipat	0.956	0.029	94	99	2.985	0.900	1.012
Jind	0.925	0.029	91	91	3.100	0.869	0.982
Fatehabad	0.866	0.066	82	78	7.669	0.735	0.996
Sirsa	0.941	0.030	83	75	3.230	0.881	1.000
Hisar	0.937	0.026	88	86	2.825	0.885	0.989
Bhiwani	0.885	0.039	100	101	4.446	0.808	0.963
Rohtak	0.951	0.022	102	114	2.363	0.907	0.995
Jhajjar	0.906	0.037	87	88	4.068	0.834	0.979
Mahendragarh	0.921	0.027	94	93	2.981	0.867	0.975
Rewari	0.948	0.020	99	103	2.144	0.908	0.987
Gurgaon	0.906	0.033	134	132	3.629	0.842	0.971
Faridabad	0.790	0.050	114	112	6.294	0.692	0.887
Mewat	0.490	0.044	240	237	9.069	0.403	0.578
**Received Measles Vaccines (last and last but one living children (age 12-35 months)**							
Panchkula	0.826	0.041	99	105	4.991	0.745	0.907
Ambala	0.919	0.047	73	66	5.142	0.826	1.012
Yamunanagar	0.855	0.036	101	110	4.231	0.784	0.926
Kurukshetra	0.825	0.036	107	115	4.305	0.755	0.895
Kaithal	0.734	0.053	113	120	7.159	0.631	0.837
Karnal	0.869	0.042	110	112	4.836	0.786	0.951
Panipat	0.718	0.061	114	100	8.517	0.598	0.838
Sonipat	0.839	0.047	94	99	5.558	0.748	0.931
Jind	0.673	0.072	91	91	10.640	0.533	0.814
Fatehabad	0.757	0.073	82	78	9.639	0.614	0.900
Sirsa	0.762	0.056	83	75	7.379	0.651	0.872
Hisar	0.657	0.075	88	86	11.362	0.511	0.804
Bhiwani	0.718	0.054	100	101	7.566	0.612	0.825
Rohtak	0.806	0.040	102	114	4.955	0.727	0.884
Jhajjar	0.696	0.054	87	88	7.742	0.591	0.802
Mahendragarh	0.719	0.045	94	93	6.254	0.631	0.807
Rewari	0.743	0.046	99	103	6.153	0.654	0.833
Gurgaon	0.764	0.061	134	132	8.046	0.643	0.884
Faridabad	0.577	0.069	114	112	11.890	0.442	0.711
Mewat	0.205	0.033	240	237	16.283	0.140	0.271
