# Supplementary material for: Effectiveness of Multiple-Strategy Community Intervention in Reducing Geographical, Socioeconomic and Gender Based Inequalities in Maternal and Child Health Outcomes in Haryana, India
Source: PLoS One. 2016 Mar 22;11(3):e0150537. doi: 10.1371/journal.pone.0150537 (PMC4803212; doi:10.1371/journal.pone.0150537)
Supplement: S3 File — Haryana Report. Round 4. 2012–13. (PDF) [file pone.0150537.s007.pdf]

DLHS-4

सत्यमेव जयते

Government of India

Ministry of Health and Family Welfare

HARYANA

DISTRICT LEVEL HOUSEHOLD AND FACILITY SURVEY (2012-13)

(स्थापना / Established in 1956)

बेहतर भविष्य के लिए क्षमता निर्माण

Capacity Building for a Better Future

International Institute for Population Sciences

(*Deemed University*)

Mumbai

INTERNATIONAL INSTITUTE FOR POPULATION SCIENCES

Vision: "To position IIPS as a premier teaching and research institution in population sciences responsive to emerging national and global needs based on values of inclusion, sensitivity and rights protection."

Mission: "The Institute will strive to be a centre of excellence on population, health and development issues through high quality education, teaching and research. This will be achieved by (a) creating competent professionals, (b) generating and disseminating scientific knowledge and evidence, (c) collaboration and exchange of knowledge, and (d) advocacy and awareness."

सत्यमेव जयते

Government of India

Ministry of Health and Family Welfare
New Delhi-110 011

District Level Household and Facility Survey 2012-13

Haryana

(स्थापना / Established in 1956)

बेहतर भविष्य के लिए क्षमता निर्माण

Capacity Building for a Better Future

International Institute for Population Sciences
(*Deemed University*)
Mumbai-400 088

2014

Suggested citation:- International Institute for Population Sciences (IIPS), 2014. *District Level Household and Facility Survey (DLHS-4), 2012-13: India. Haryana:* Mumbai: IIPS.

For additional information, please contact:
Director/Project Coordinator (DLHS-4)
International Institute for Population Sciences
Govandi Station Road, Deonar
Mumbai - 400 088 (India)
Telephone: 022-2556 3254/5/6, 022-4237 2465, 42372411
Fax: 022-25563257, 25555895
Email: rchpro@iips.net, director@iips.net
Website: http://www.rchiips.org
http://www.iipsindia.org

Additional Director General (Stat.)
Ministry of Health and Family Welfare
Government of India
Nirman Bhavan
New Delhi 110 011
Telephone: 011 - 23061334
Fax: 011 - 23061334
Email: adg-mohfw@nic.in

Chief Director (Stat.)
Ministry of Health and Family Welfare
Government of India
Nirman Bhavan
New Delhi 110 011
Telephone: 011 - 23062699
Fax: 011 - 23062699
Email: cdstat@nic.in

Website: http: //www.mohfw.nic.in

CONTRIBUTORS

B. Paswan
S. K. Singh
Imran Ahmad
Ashish Kumar Upadhyay

CONTENTS **PAGE**

1. INTRODUCTION AND HOUSEHOLD CHARACTERISTICS	1
2. SURVEY DESIGN	2
3. SURVEY INSTRUMENTS	4
4. DEMOGRAPHIC BACKGROUND OF HARYANA	6
5. CHARACTERISTICS OF WOMEN AND FERTILITY	8
6. MATERNAL HEALTH CARE	12
7. CHILD HEALTH AND IMMUNIZATION	15
8. FAMILY PLANNING AND CONTRACEPTIVE USE	19
9. REPRODUCTIVE HEALTH	22
10. PERSONAL HABITS	24
11. MORBIDITY STATUS	28
12. NUTRITION AND HEALTH	32
13. HEALTH FACILITIES	38
TABLES	43-173
**APPENDIX**	175-179

LIST OF TABLES PAGE

Table 1.1	Basic demographic indicators	43
Table 1.2	Number of households, ever-married women	43
Table 1.3	Distance from the nearest educational facility	44
Table 1.4(a)	Distance from the nearest health facility	44
Table 1.4(b)	Programmes beneficiaries	44
Table 1.5	Reasons for dropping out of school	44
Table 1.6(a)	Housing characteristics and household assets	45
Table 1.6(b)	Housing characteristics by district	46
Table 1.7	Household characteristics	47
Table 1.8	Household population by age and sex	48
Table 1.9	Marital status of the household population	49
Table 1.10	Age at marriage	50
Table 1.11	Educational level of the household population	51
Table 1.12	Educational level of the household population	51
Table 1.13	Educational level of the household population	52
Table 1.14	Currently attending school	52
Table 1.15	Availability of facility and health personnel by district	53
Table 1.16	Birth registration	54
Table 1.17	Birth registration	54
Table 2.1	Background characteristics of ever married women	57
Table 2.2	Level of education of ever married women	58
Table 2.3	Birth order	59
Table 2.4	Birth order by districts	60
Table 2.5	Children ever born	61
Table 2.6	Outcomes of pregnancy	62
Table 2.7	Outcomes of pregnancy	63
Table 2.8	Fertility preferences	63
Table 3.1	Place of Antenatal Check-Up	67
Table 3.2	Antenatal Care by district	68
Table 3.3	Components of Antenatal Check-Up	69
Table 3.4	Women received advice during Antenatal care	70
Table 3.5(a)	Antenatal care: ANC visits and time of first ANC	71
Table 3.5(b)	Antenatal care: TT, IFA and ANC	72
Table 3.6	Antenatal care indicators and pregnancy complications	73
Table 3.7	Place of delivery and assistance	74
Table 3.8	Mode of transportation used for delivery and arrangement of transportation	75
Table 3.9	Place of delivery and assistance characteristics by district	76
Table 3.10	Reasons for not going to health institutions for delivery	77
Table 3.11	Delivery complications	78
Table 3.12	Post-delivery complications	79
Table 3.13	Any check-up after delivery	80
Table 3.14	Complications during pregnancy, delivery and post-delivery period	81
Table 3.15	Complications during pregnancy, delivery and post-delivery period	82
Table 3.16	Awareness of the danger signs of new born	83
Table 4.1	Timing and childhood check-ups	87
Table 4.2	Initiation of breastfeeding	88
Table 4.3	Breastfeeding and weaning status	88
Table 4.4	Exclusive breastfeeding	89
Table 4.5	Breastfeeding by districts	90
Table 4.6	Vaccination of children	91
Table 4.7	Status of childhood vaccination by districts	92

LIST OF TABLES		PAGE
Table 4.8	Place of childhood vaccination	93
Table 4.9	Vitamin-A and Hepatitis-B supplementation for children	94
Table 4.10	Awareness regarding diarrhoea management	95
Table 4.11	Treatment of diarrhoea	96
Table 4.12	Awareness and treatment of Acute Respiratory Infection (ARI)	97
Table 4.13	Awareness of ors and Acute Respiratory Infection (ARI) by districts	98
Table 5.1	Awareness of contraceptive methods	101
Table 5.2	Awareness of contraceptive methods	102
Table 5.3	Awareness of contraceptive methods by district	103
Table 5.4	Ever use of contraceptive method	104
Table 5.5(a)	Current use of contraceptive methods	105
Table 5.5(b)	Duration of use of spacing methods	107
Table 5.6	Age at the time of sterilization	108
Table 5.7	Contraceptive prevalence rate by district	109
Table 5.8	Sources of modern contraceptive methods	110
Table 5.9	Cash benefits received after sterilization	111
Table 5.10	Health problems with current use of contraception and treatment received	112
Table 5.11	Reasons for discontinuation of contraception	113
Table 5.12	Future intention to use contraception	114
Table 5.13	Advice on contraceptive use	115
Table 5.14	Reasons for not using modern contraceptive methods among rhythm and withdrawal method users	116
Table 5.15	Unmet need for family planning services	117
Table 5.16	Unmet need for family planning services by district	118
Table 6.1	Menstruation related problems by background characteristics	121
Table 6.2	Source of knowledge about RTI/STI by background characteristics	123
Table 6.3	Knowledge of mode of transmission of RTI/STI by background characteristics	125
Table 6.4	Symptoms of RTI/STI by background characteristics	126
Table 6.5	Discussed about RTI/STI problems with husband and sought treatment by background characteristics	128
Table 6.6	RTI/STI indicators by districts	129
Table 6.7	Knowledge of HIV/AIDS	130
Table 6.8	Knowledge about mode of transmission of HIV/AIDS by background characteristics	132
Table 6.9	Knowledge of HIV prevention methods by background characteristics	133
Table 6.10	Misconception about transmission of HIV/AIDS by background characteristics	134
Table 6.11	Knowledge about the place where HIV/AIDS test can be done	135
Table 6.12	Undergone HIV/AIDS test	137
Table 6.13	HIV/AIDS indicators by districts	138
Table 7.1	Personal habits	141
Table 7.2	Personal habits-Men	142
Table 7.3	Personal habits-Women	143
Table 7.4	Personal habits	144
Table 7.5	Personal habits tobacco	144
Table 7.6	Personal habits smoke	144
Table 7.7	Personal habits drink alcohol	145
Table 7.8	Morbidity details	145
Table 7.9	Morbidity details	145
Table 7.10	Morbidity details	145
Table 7.11	Morbidity details	146
Table 7.12	Morbidity details	146
Table 7.13	Morbidity details	147
Table 7.14	Morbidity details	147

LIST OF TABLES **PAGE**

Table 7.15	Morbidity details	147
Table 7.16	Tuberculosis	148
Table 8.1	Nutritional status of children	151
Table 8.2	Nutritional status of children by districts	152
Table 8.3	BMI (Body Mass Index) of women	153
Table 8.4	BMI (Body Mass Index) of women	153
Table 8.5	Prevalence of anaemia among children	154
Table 8.6	Anaemia among school going/adolescent population	154
Table 8.7	Anaemia among population aged 20 years and above	155
Table 8.8	Anaemia among population children, adolescents aged 20 years and above	156
Table 8.9	Anaemia among pregnant women	156
Table 8.10	Prevalence of diabetes	157
Table 8.11	Prevalence of diabetes	157
Table 8.12	Prevalence of diabetes	158
Table 8.13	Prevalence of diabetes	158
Table 8.14	Blood pressure	159
Table 8.15	Blood pressure	160
Table 8.16	Blood pressure	161
Table 8.17	Blood pressure	162
Table 8.18	Presence of iodized salt in household	163
Table 8.19	Presence of iodized salt in household	163
Table 9.1	Average population covered by health facility by districts	167
Table 9.2	Status of infrastructure at Sub-Health Centre functioning in government building by districts	167
Table 9.3	Percentage of Sub-Health Centres having different activities by districts	168
Table 9.4	Available human resources at Sub-Health Centres by districts	168
Table 9.5	Available human resources at Primary Health Centres by districts	169
Table 9.6	Available infrastructure at Primary Health Centres by districts	169
Table 9.7	Specific health facilities available at Primary Health Centres by districts	170
Table 9.8	Number of Primary Health Centres having different activities by districts	170
Table 9.9	Human resources available at Community Health Centres by districts	171
Table 9.10	Specific health care facilities available at Community Health Centres by districts	171
Table 9.11	Number of Community Health Centres having different activities by districts	172
Table 9.12	Human resources & other services available at Sub-Divisional Hospitals by districts	172
Table 9.13	Human resources & other services available at District Hospitals by districts	173

LIST OF FIGURES — PAGE

Figure 1	Source of drinking water	7
Figure 2	Toilet facilities	7
Figure 3	Age-sex composition of Haryana, 2012-13	7
Figure 4	School attendance by age and sex	8
Figure 5	Mean children ever born by districts	10
Figure 6	Desire for the additional child/next child	11
Figure 7	Any ANC by selected background characteristics	12
Figure 8	Progress in institutional delivery	14
Figure 9	Change in full immunization coverage of children	16
Figure 10	Percent of currently married women using contraceptive methods	19
Figure 11	Change in contraceptive prevalence rate	19
Figure 12	Change in unmet need for contraception	20
Figure 13	Contraceptive prevalence rate and unmet need by districts	22

LIST OF MAPS — PAGE

Map 1	Full ante-natal checkup by districts	13
Map 2	Institutional delivery by districts	14
Map 3	Full immunization coverage of children aged 12-23 months by districts	18
Map 4	Contraceptive prevalence rate for any method by districts	21

ACRONYMS

AFMC	Administrative and Financial Management Committee
AHS	Annual Health Survey
AIDS	Acquired Immuno Deficiency Syndrome
ANC	Antenatal Care
ANM	Auxiliary Nurse Midwife
ARI	Acute Respiratory Infection
ASHA	Accredited Social Health Activist
AWW	Anganwadi Worker
AYUSH	Ayurveda, Yoga and Naturopathy, Unani, Siddha and Homeopathy
BCG	Bacillus Calmette Guerin
BP	Blood Pressure
BPL	Below Poverty Line
CAB	Clinical Anthropometric Biochemical (Test)
CAPI	Computer Assisted Personnel Interviewing
CHC	Community Health Centre
CPR	Contraceptive Prevalence Rate
DBS	Dried Blood Spot
DH	District Hospital
DLHS	District Level Household and Facility Survey
DPT	Diphtheria, Pertussis and Tetanus
EAG	Empowered Action Group
ECG	Electrocardiogram
ECP	Emergency Contraceptive Pill
ELISA	Enzyme-linked Immunosorbent Assay
EPI	Expanded Programme on Immunization
FA	Field Agency
FBS	Fasting Blood Sugar
FHW	Female Health Worker
FRU	First Referral Unit
FOD	Field Operation Division
FP	Family Planning
FS	Female Sterilization
FSU	First Stage Unit
GPS	Global Positioning System
GoI	Government of India
HH	Household
HIV	Human Immuno Deficiency Virus
ICDS	Integrated Child Development Scheme
ICTC	Integrated Counselling and Testing Centre
IEC	Information, Education and Communication
IFA	Iron and Folic Acid
IIPS	International Institute for Population Sciences
IMNCI	Integrated Management of Neonatal and Childhood Illnesses
IMR	Infant Mortality Rate
IPHS	Indian Public Health Standards
IUD	Intra-uterine Device
JSY	Janani Suraksha Yojana
LMO	Lady Medical Officer
LPG	Liquefied Petroleum Gas
MCEB	Mean Children Ever Born
MDG	Millennium Development Goal
MMR	Maternal Mortality Ratio
MO	Medical Officer
MoHFW	Ministry of Health and Family Welfare
MoU	Memorandum of Understanding

ACRONYMS

MoA	Memorandum of Agreement
MTP	Medical Termination of Pregnancy
NC	Natal Care
NIC	National Informatics Centre
NIHFW	National Institute of Health and Family Welfare
NGO	Non-Governmental Organisation
NPP	National Population Policy
NRHM	National Rural Health Mission
NSSO	National Sample Survey Organization
NSV	Non-scalpel Vasectomy
OBC	Other Backward Class
OPD	Out-Patient Department
ORS	Oral Re-hydration Salt
ORT	Oral Re-hydration Therapy
OT	Operation Theatre
PHC	Primary Health Centre
PI	Partner Institute
PNC	Post Natal Care
PRC	Population Research Centre
PPS	Probability Proportional to Size
PSU	Primary Sampling Unit
RCH	Reproductive and Child Health
RKS	Rogi Kalyan Samiti
RTI	Reproductive Tract Infection
SDH	Sub-Divisional Hospital
SDRD	Survey Design and Research Division
SC	Scheduled Caste
SHC	Sub-Health Centre
ST	Scheduled Tribe
STI	Sexually Transmitted Infection
TBA	Trained Birth Attendant
TAC	Technical Advisory Committee
TOT	Training of Trainers
TT	Tetanus Toxoid
TV	Television
UFS	Urban Frame Survey
UFWC	Urban Family Welfare Centre
UHP	Urban Health Post
UIP	Universal Immunization Programme
UNFPA	United Nations Population Fund
UNICEF	United Nation Children's Fund
USU	Ultimate Stage Sampling Unit
UT	Union Territory
VCTC	Voluntary Counseling and Testing Centre
VHNSC	Village Health Nutrition and Sanitation Committee
WHO	World Health Organisation

Preface and Acknowledgements

The District Level Household and Facility Survey-4 (DLHS-4) is a nationwide survey covering 640 districts from 36 States and Union Territories of India. This is the fourth round of the district level household survey which was conducted during 2012-13. The Survey was funded by the Ministry of Health and Family Welfare, Government of India.

At the outset, we acknowledge our sincere gratitude to the Ministry of Health & Family Welfare, Government of India for designating the International Institute for Population Sciences (IIPS) as the nodal agency for conducting District Level Household and Facility Survey-4 (DLHS-4) in India. We would also like to take this opportunity to acknowledge Shri Bhanu Pratap Sharma, Secretary-Ministry of Health and Family Welfare (MoHFW), Government of India for his advice, suggestions and support. Our special thanks are due to Shri Lov Verma and Shri Keshav Desiraju former Secretaries, Ministry of Health and Family Welfare (MoHFW), Government of India, for providing overall guidance and support extended to the project. We gratefully acknowledge the active involvement, assistance, help, co-operation and suggestions received time to time from Shri C.R.K. Nair, Additional Director General, Dr. Rattan Chand, Chief Director and Shri Biswajit Das, Director-Statistics Division, Ministry of Health and Family Welfare, Government of India. We also extend our thanks to Smt. Madhu Bala, former Additional Director General and Shri Rajesh Bhatia, former Director-Statistics Division, Ministry of Health and Family Welfare, Government of India for their support from time to time.

We gratefully acknowledge the NIHFW, New Delhi, especially Dr. M. M. Misro, Dr. T. G. Srivastava and Dr. Kalpna, for their immense help, assistance, support and coordination with all Partner Institutes to bring out quality DBS results/data. We also acknowledge our sincere gratitude to all Partner Institutes for providing training and support of CAB components and bringing out the quality DBS results.

We sincerely extend our appreciation to HLL Life Care Ltd., New Delhi, for procuring CAB equipments and consumables also supply chain to different states across the country.

Our special thanks are all the members of Technical Advisory Committee of DLHS-4, particularly Dr. N. S. Shastry, Chairman, Former DG & CEO (NSSO), for their constant involvement and technical inputs and support at various stages of the survey.

We also gratefully acknowledge all members of Sub-Committee on Sampling especially Shri G. C. Manna, Chairman, DDG, CSO, MoSPI for their technical support received from time to time.

Thanks are also due to Dr. Rajiv Mehta and Shri A. K. Mehra, former Additional Director Generals at the National Sample Survey Organisations, Kolkata for providing UFS blocks.

We thank Dr. T. K. Roy, Former Director, IIPS, for reviewing the model report and for his useful suggestions.

This acknowledgement cannot be concluded without expressing appreciation for the efforts and hard work put in by the field investigators, supervisors, health investigators in collecting data and timely transferring data to IIPS.

Last but not the least, we are grateful and appreciate the efforts of all the respondents who participated and spared their valuable time with us by providing the required information.

DLHS-4 Coordinators
International Institute for Population Sciences

1. INTRODUCTION AND HOUSEHOLD CHARACTERISTICS

This state report for Haryana pertains to the fourth round of District Level Household and Facility Survey (DLHS-4) 2012-13 following the preceding three rounds undertaken by the Ministry of Health and Family Welfare (MoHFW), Government of India (GoI). In the past (Round-I in 1998-99, Round-II in 2002-04, and Round-III in 2007-08) with the main objective to provide reproductive and child health related database at district level in India. The data from these surveys have been useful in setting the benchmarks and examining the progress the country has made after the implementation of RCH programme. In addition, the evidences generated by these surveys have been useful for the purpose of monitoring and evaluation of the ongoing programmes and the aspect of planning of suitable strategies by the central and state governments. In view of the completion of eight years of National Rural Health Mission (2005-12), that it was a felt there was a need to focus on the achievements and improvements. The Ministry of Health and Family Welfare, Government of India, therefore initiated the process of conducting DLHS-4 and designated the International Institute for Population Sciences (IIPS) as the nodal agency to carry out the survey. MoHFW, provided funds for implementation of DLHS-4, guided by a duly constituted Technical Advisory Committee (TAC).

The main objective of District Level Household and Facility Survey-4 (DLHS-4) is to provide maternal and child health care (MCH) indicators and prevalence of morbidity for a wide range of common, communicable, non-communicable and lifestyle diseases for the year 2012-13 covering the following aspects:

- Household basic amenities
- Prevalence of morbidity
- Coverage of ante-natal services and immunization services.
- Proportion of institutional/safe deliveries
- JSY Beneficiaries
- Economic burden of delivery
- Contraceptive prevalence rate
- ASHA's involvement
- Unmet need for family planning
- Awareness about RTI / STI and HIV / AIDS
- Infrastructure, manpower, equipments, drugs, services of public health facilities
- Linkage between health facility and MCH indicators

Bilingual questionnaires prepared in Haryanavi and English language pertaining to Household, Clinical, Anthropometric and Bio-Chemical tests (CAB) and Ever Married Women (age 15-49) were used and canvassed using Computer Assisted Personal Interviewing (CAPI). It was for the first time in the country that large scale demographic and health survey at the district level was successfully carried out by using Computer Assisted Personal Interviewing (CAPI) in DLHS-4. The CAPI software was developed by using MMIC (Multi-Mode Interviewing Capability) tool. Mini laptops were also loaded with CAPI software and bilingual questionnaires and provided to the Field Agencies authorized to carry

out the survey with the designated states. Each team was provided four CAPIs/Mini laptops, one for each investigator. Supervisors were responsible for directly uploading the completed PSU's data to the IIPS, FTP server located in Mumbai on day-to-day basis. The use of CAPI optimized resources were required for transferring the filled questionnaires from field to the state office, data entries and received at IIPS. For the first time biomarkers were also used in DLHS-4. The village and health facility questionnaires were canvassed by using paper & pen method in DLHS-4. In the household questionnaire, information of all the members of the household and socio-economic characteristics of the household, possessed assets, number of marriages, morbidities and deaths in the household since January 2008, and also drinking water, toilet, drainage and kitchen facilities data were collected. The ever-married women questionnaire contained information on women's characteristics, maternal care, immunization and childcare, contraception and fertility preferences, reproductive health including knowledge about HIV/AIDS. The village questionnaire contained information on the availability of health, education and other facilities in the village, and whether the facilities are accessible throughout the year. The health facility questionnaire contained information on human resources, infrastructure, equipments, drugs and services. For the first time, a population-linked facility survey has been conducted in DLHS-4. At the district level, all Community Health Centres, Sub-Divisional Hospitals and District Hospitals were covered. Further, all Sub-Health Centres and Primary Health Centres which cater to the needs of the population of the selected PSUs were also covered. Fieldwork in Haryana for all the 21 districts was conducted during June to December 2013, gathering information from 33,772 households and 28,776 ever married women (15 to 49 years). Table 1.2 provides breakup of PSUs and households by district and rural urban residence.

2. SURVEY DESIGN

DLHS-4 is a district level survey and a multi-stage stratified designed adopted for selection of representative sample of each district in Haryana. Rural and urban areas of a district are considered as natural strata. Wherever applicable, urban population in a district was further stratified into million class cities and non-million class cities. For the purpose of sampling of the urban samples, two-stage sampling was used where the primary sampling unit (PSU) is the NSSO urban frame survey (UFS) blocks and second stage sampling unit (SSU) is the household. The urban PSUs are selected by equal probability without replacement and USU selected by process of circular systematic sampling. The allocation of PSUs to million and non-million class cities was proportional to relative sizes. Distribution of PSUs of a district is proportional to projected urban population of the district. For districts with less than projected 30 percent urban population, urban PSUs are oversampled. The sampling frame used for urban sampling is the town and city wise list of NSSO UFS blocks for 2007-08 provided by the SRD Unit of National Sample Survey Organisation (NSSO), Kolkata.

In rural areas of each district, sampling design is two-stage sampling with census villages as PSU and household as the second stage sampling unit (SSU). The PSUs are selected by PPS with replacement and SSU are selected by circular systematic sampling. Large selected PSU with more than 300 households are divided into at least three segments in such a way that each segment has by and large the same number of households and two segments are then

selected by SRS. List of villages in a district in Census 2001 are updated by removing villages of 2001 which have been designated as urban in 2007-08, NSSO UFS block list and this serves as the sampling frame for sampling of rural PSUs from a district.

Selection of rural health facilities in DLHS-4 is linked with the sampled rural PSUs. Primary Health Centres (PHC) and Sub-Health Centres (SHC) catered to the health care needs of the sampled rural PSUs were included in the Facility Survey (FS) of DLHS-4. All Community Health Centres (CHC), Sub-Divisional Hospitals and District Hospitals are covered under the Facility Survey of DLHS-4.

2.1. Sampling Weight

In generating district level demographic indicators, sample weight for household, women and children will be used. The weights for a particular district are based on three selection probabilities

f_1^i, f_2^i and f_3^i pertaining to i^{th} PSU of the district. These probabilities are defined as

f_1^i = Probability of selection of ith PSU in a district

$= (n_r * Hi)/H,$

Where n_r is the number of rural PSU to be selected in a district, H_i refers to the number of household in the ith PSU and $H = \Sigma H_i$, total number of household in a district.

f_2^i = Probability of selecting segment (s) from segmented PSU (in case the ith selected PSU is segmented) = (Number of segments selected after segmentation of PSU)/(number of segment created a PSU)

The value of f_2^i is to be equal to one for un-segmented PSUs.

f_3^i = probability of selecting a household from the total listed households of a PSU or in segment(s) of a PSU

$= (25*HR_i)/ HL_i$

Where HR_i is the household response rate of the ith sampled PSU and HL_i is the number of households listed in i^{th} PSU in a district.

For urban PSU, f_1^i is computed either as the ratio of number of UFS blocks included in the sample to the total number of UFS blocks of the district.

The probability of selecting a household from the district works out to be

$$f^i = f_1^i * f_2^i * f_3^i$$

The non-normalized weight for the ith PSU of the district is, $w^i = 1/f^i$ while the normalized weight used in the generation of district indicators for the ith district would be

$$= \frac{\sum_i n_i}{\sum_i n_i * w^i} * w^i$$

Where n_i is the number of households interviewed in the ith PSU. The weight for women and children are computed in the similar manner considering corresponding response rate.

3. SURVEY INSTRUMENTS

The main instrument for collection of data in DLHS-4 was a set of structured questionnaires, namely, household, ever married woman, and village questionnaires as components of household survey. In the facility, separate questionnaires are used for Sub-Health Centre (SHC), Primary Health Centre (PHC), Community Health Centre (CHC), Sub-Divisional Hospital (SDH) and District Hospital (DH). Household and ever married women questionnaires are bilingual, with questions in both Haryanavi and English languages.

3.1 Household Questionnaire:- The household questionnaire starts with listing of all usual residents in each sample household including visitors who had stayed the night before the interview. The listing of usual resident members is used for identification of eligible respondents for ever married women and CAB (Clinical, Anthropometric and Biochemical) tests. For individual household member information on age, sex and marital status, relationship to the head of the household and education were collected. Marriages and deaths to members of household were also recorded. Efforts were made to get information about maternal deaths. Information were also collected on the main source of drinking water, type of toilet facility, source of lighting, type of cooking fuel, religion and caste of household head and ownership of durable goods in the household.

An added feature of household questionnaire of DLHS-4 is the collection of data on disability status, injury, acute and chronic illness for all members of the household.

Clinical, Anthropometric and Biochemical (CAB) tests: An important component of household questionnaire is the collection of biomarkers of eligible household members for the first time on a large scale demographic and health survey in the country at district level. This includes weight and height for all household members of age one month and above, Haemoglobin level for all household members aged 6 months and older, random blood sugar test and blood pressure measurements for all household members aged 18 years and above.

3.2 Ever Married Woman's Questionnaire:- The respondents for the ever married woman's questionnaire are ever married women in 15-49 years of age living in the sampled households. Details on age, age at marriage, place of birth, educational attainment, number of biological children ever born and surviving by sex were collected. Accounts of ante-natal checks, experience of pregnancy related complications, place of delivery, delivery attendant and post-partum care, together with history of contraceptive use, sex preference of children and fertility intentions were recorded. For the recent births, immunization status of children was collected either from the immunization card or asking the mother about the status of immunization of the child. The other information collected includes knowledge and awareness about RTI/STI and HIV/AIDS by source and treatment seeking behavior of RTI/STI.

3.3 Village Questionnaire:- This questionnaire was designed to collect information on availability and accessibility of education, health, transport and communication facilities at village level. Functioning of village committees and utilization of fund were additionally collected from the sampled villages. Information relating to implementation and beneficiaries of various government programmes on girl child, maternal care, sanitation, food security,

employment generation, and women's empowerment are also gathered as part of village information.

3.4 Facility Questionnaire:- In the facility survey, the information collected at the SHC level were availability of human resources, physical infrastructure, equipments and essential drugs and MCH service provided in one month preceding the survey. From the PHC, status of availability for 24x7 facility and services for delivery and new born care were collected. Additional information collected at PHC level were availability of Lady Medical Officer, functional Labour Room, Operation Theater, number of beds, drug storage facilities, waiting room for OPD, availability of RCH related equipments, essential drugs and essential laboratory testing facilities. Information that were collected for Community Health Centre (CHC) includes availability of 24X7 services for delivery and new born care, status of in-position clinical, supporting and Para-medical staff, availability of specialists trained for NSV (Non-Scalpel Vasectomy), emergency obstetric, MTP, new born care, treatment of RTI / STI, IMNCI, ECG etc. Physical infrastructure of CHC such as water supply, electricity, communication, waste disposal facilities, OT, Labour Room and availability of residential quarters for medical doctors were also collected in the facility survey. From the Sub-Divisional and District Hospitals status of availability of essential laboratory and ambulance services, emergency obstetric care service, availability of specialists, nurses, paramedics and technicians either on regular or contractual basis were collected in addition to infrastructure, provision for bio-medical and waste disposal and availability of residential quarters for doctors, nurses and staff. The mode of collection of information for health facilities is directly asking to the concerned officials, physical inspection and recording from relevant registers.

3.5 Sample Implementation

The field implementation starts with the preparation of location and layout maps of sampled PSUs in rural areas and obtaining map of sampled NSSO UFS blocks in urban areas. This is followed by preparation of list of households which served as the sampling frame for selection of representative households and it involved mapping and listing of structures and households for each sampled primary sampling unit (PSU) following the preparation of location and layout maps. The mapping and listing was carried out for each PSU by a team comprising of a mapper, a lister and a supervisor. A PSU in rural area is a village or part of a village or a group of small villages and it is NSSO UFS block in an urban area.

From the sampling frame of households prepared by mapping and listing, a sample of 28 households were selected by circular systematic sampling. Household and ever married women's questionnaires were canvassed by a team of 3 female and one male investigators, one supervisor and two health investigators were assigned for collection of CAB information. For quality assurance, field teams were monitored constantly by Project Officers, Officials of PRC, MoHFW, and Partner Institutes who facilitates DBS testing. Time to time DLHS-4 Project Coordinators of IIPS also made field visits to check and provide support to field teams.

4. DEMOGRAPHIC BACKGROUND OF HARYANA

Basic demographic indicators of Haryana and its districts based on Census 2011 are shown in Table 1.1. The population of the state in Census 2011 is enumerated as 25,352 in thousands and population of the state is concentrated mainly in the districts of Karnal, Sonipat, Hisar, Bhiwani, Gurgaon and Mewat. The decadal growth rate of the state during 2001-2011 Census is 19.9 percent. Decadal growth rate is highest in Gurgaon (73.1%) and lowest in Jhajjar district (8.9%) during this period. The sex ratio of the state is 879 females per 1000 males, it is lowest (854) in Gurgaon and highest (907) in Faridabad district. The overall literacy rate is 75.6 percent and literacy rate is 84.1 percent for males and 65.9 percent for females.

4.1 Sample Coverage

DLHS-4 surveyed a total of 1,259 primary sampling units (PSUs) covering 33,772 households with 95.7 percent response rate and 28,776 ever married women in reproductive age 15-49 years with 91.3 percent response rate. Table 1.2 shows the number of PSUs, households and ever married women interviewed and corresponding response rates by districts. Household response rate in the districts varies from 90.4 in Fatehabad district to 97.5 percent in Gurgaon district while that for the ever married women it varied from 84.7 percent in Fatehabad to 97.8 percent in Panchkula district.

4.2 Village Facilities

Total number of PSUs surveyed in Haryana is 1,259 out of this 711 are rural PSUs. Most villages (93.1%) have a primary school in the village (Table 1.1). In 60 percent of the villages, there is Sub-Health Centre (SHC) (Table 1.4a). Out of total 708 villages 80 percent (565 villages) have beneficiaries of ICDS, while 94 percent have JSY beneficiaries and 86 percent (606 villages) have beneficiaries of JSSK (Table 1.4b). As can be seen from Table 1.15 almost all sampled villages 99 percent have Anganwadi centre, 66 percent have access to any government health facility, 23 and 44 percent of the sampled villages have Primary Health Centre (PHC) and Village Health Nutrition and Sanitation Committee (VHNSC) in the villages respectively.

4.3 Household Amenities and Characteristics

As regards housing condition, as can be noted from Table 1.6 (a), 69 percent of the surveyed households live in pucca houses, 7 percent in kachha houses and 24 percent in semi-pucca houses. As many as 98 percent of households have electricity connection, 23 percent of households use woods for cooking while 46 percent use LPG, 89 percent of households have mobile phone, 85 percent owned television, 42 percent owned bicycle while 41 percent owned motor cycle/ scooter and 10 percent owned car/jeep/van.

The sources of drinking water are shown in figure 1 and it is noted that 72 percent of households are using piped water in to dwelling/ yard/plot and public tap/stand pipe for drinking and 26 percent of households are using tube well or borehole water. As can be seen from figure 2, households which do not have access to improved clean toilet constitute 16 percent of the total surveyed households and more than two-third of the households have access to improved flush/septic/pit toilets.

Table 1.6 (b) provides household access to electricity, drinking water, toilet and cooking gas and type of house by districts. The mean household size of the state is 4.9 while it is 5.1 in rural and 4.6 in urban (Table 1.7). One member households constitute 3.5 percent of all surveyed households, 89 percent household heads are male, median age of household members is 48 years. Hindu's are majority among houholds that is 92 percent. A significant share of 30 percent of the household heads are scheduled caste (SC) and 36 percent of household heads are from the general caste.

Figure 1. Source of drinking water

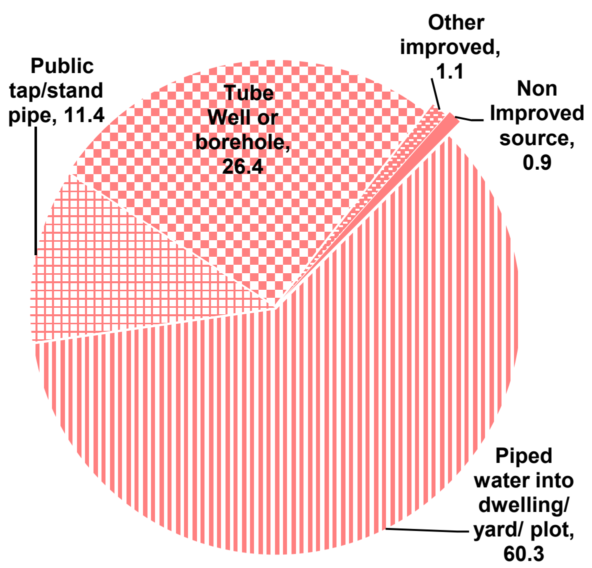

Figure 2. Toilet facility

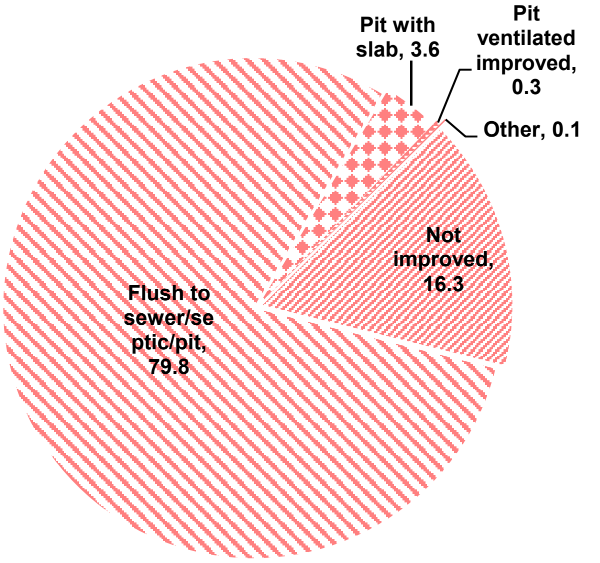

The age-sex composition of the population of Haryana is depicted in the population pyramid shown in figure 3. The pyramid is characterized by a shrinking base indicating declining trend in fertility, more males than females in 15-49 years and at older ages.

Figure 3: Age- sex composition of Haryana

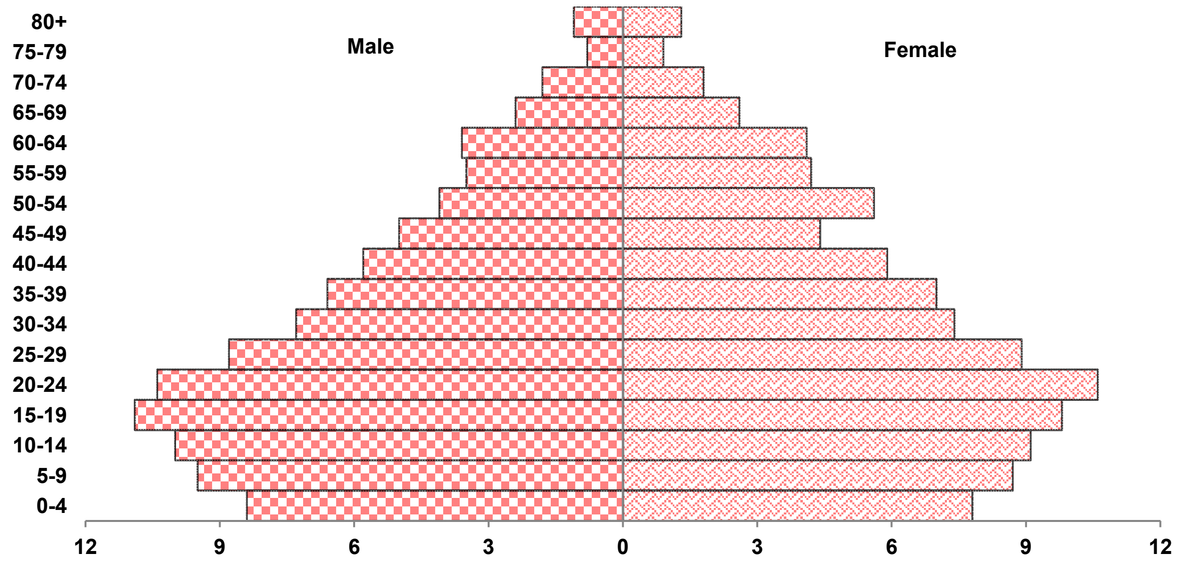

Table 1.8 provides differential in age-sex structures of rural and urban population of the state. As evident from Table 1.9 there is sizeable proportion of unmarried males and females in 20-

29 years and another distinct feature is that there are more widowed/divorced/separated among females than males. The mean age at marriage for girls is 21 years while it is 24 years among boys. Mean age at marriage for girls and boys by districts are shown in Table 1.10. Six percent of the marriages among girls is below the legal age of 18 years and 17 percent of the boys got married below the legal age of 21 years. Tables 1.11 through 1.13 provide details about years of schooling of sampled household members by age, sex, caste and religion by rural-urban residence. Among females 7 years and older 0.7 percent are non-literate and corresponding figure among males is 0.4 percent. More among females, 50.1 percent have 11 or more years of schooling as compared to 40 percent among males. Regardless of sex individuals about 24 percent of the literate population has less than five years of schooling. The non-literate persons are less in urban than in rural and more persons have 11 or more years of schooling than among rural residents. Table 1.14 provides rate of current school attendance by age, residence, religion and caste.

Figure 4 shows the school attendance by age, 6-11 years and 12-17 years, the stage of primary and secondary education respectively by sex. The state achieved 98 percent school attendance among 6-11 years children and 94 percent among 12-17 years suggesting the existence of dropout at the secondary level. There is no evidence of sex differential in school attendance among 6-11 years but for 12-17 years, school attendance among female is 93 percent as compared to 95 percent among males.

Figure 4. School attendance by age and sex

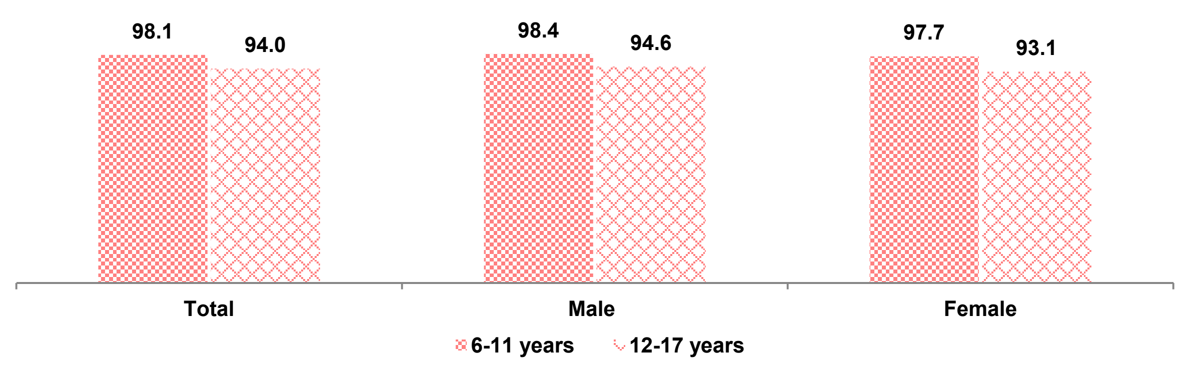

5. CHARACTERISTICS OF WOMEN AND FERTILITY

The distribution by age of women surveyed remains almost constant above age 25 years and above in both rural and urban settings. Age at consummation of marriage is below 18 years is found to be low only 30.1 percent of ever-married sampled women between 15-49 years irrespective of residence background. In rural, 32.9 percent of surveyed women reported their age at consummation of marriage below 18 years. In urban area, 25.1 percent of surveyed women reported that they had started living with husband before reaching at age 18 years (Table 2.1). There are more non-literate women in rural (41.1%) than in urban areas (24.4%). Non-literate husbands are less by 15.4 percentage points compared to non-literate wives/women in rural areas. Around thirty five percent of women are non-literate whereas 36.7 percent of women are educated at least for 10 years. The proportion of husbands with 10 years or more schooling is 44.7 percent in rural, 61.8 percent in urban and 51 percent as combined. Nearly half of the ever-married women (44.5%) were married for 15 years or more

with a marginal difference between rural and urban settings. In case of less than 15 years of marital duration, the distribution of ever-married women in the categories of less than 5 years, 5-9 years and 10-14 years marital duration are almost uniform, with around 18 percent in each category. The proportion of Hindu women is highest and found to be 92 percent followed by 4.5 percent Muslim. The proportion of Muslim women is higher in rural areas (5.4%) as compared to urban areas (2.8%). The proportion of Hindu women is higher (93.9%) in urban areas as compared to rural areas (90.9%). The percent distribution of women by caste/tribes is skewed towards 'Others' (38.2%), followed by women belonging to other backward classes (28.4%) and Scheduled caste (28.1%). The percent of women who belong to Scheduled caste is higher (31.2%) in rural areas than in the urban areas (22.6%).

Table 2.2 shows the distribution of years of schooling among surveyed women by background characteristics. The percentage of non-literate women (21.8%) is found to be lowest in the age group 20-24. More than two fifth of rural women and one-fourth of urban women reported as non-literate in the survey. Relatively higher proportion of Muslim (73.8%) and scheduled caste women (46.7%) are found to be non-literate than other religion or cast/tribe groups respectively. Slightly above one-fifth of surveyed women (22%) had 11 or more years of schooling in Haryana comprising 14 percent in rural and 36 percent in urban. In contrast, only 2.6 percent of Muslim and 11.1 percent of scheduled caste women have 11 or more years of schooling. It is to be noted that at least 7.9 percent of women with 0-5 years of schooling and 3.7 percent of 6-8 years of schooling reported that their husband is non-literate. The percentage of women possessing beyond 11 years of schooling is lowest among Muslim (2.6%). This proportion remains highest (55.2%) for other religion women. Similarly, the proportion of women educated beyond 11 years of schooling is also found to be dismal among schedule caste (11.1%) and scheduled tribes (14.8%) even in state like Haryana.

5.1 Birth Order

Out of the total births since January 1, 2008 to ever-married women, around 67 percent births comes from rural areas and the rest 33 percent from urban areas. Almost 43 percent of them belong to women in the age group 25-29, followed by 31.3 percent from women in the age group 20-24 (Table 2.3). The distribution of these births by religion shows that 89.3 percent births belong to Hindus and 7.6 percent to Muslims and rest belong to other than these two religions. The distribution of births by caste/tribes indicates that births from the scheduled caste contribute maximum 33 percent followed by 'Others' (32%) and Other Backward Classes (30%). Out of the total births since January 1, 2008 to ever-married women, 58.3 percent were of second or higher order births and the corresponding figures are 71 percent and 67 percent respectively for non-literate and women with less than 5 years of schooling (Table 2.3).

The births of second and higher order are more in proportion among ever-married women aged 15-49 who are from rural area (58.8%), belonging to Muslim (71.9%), belonging to scheduled caste (62.7%), and among 40-45 years or older women (91.1%), compared to ever-married women educated at least up to 10 years (47%), and those belonging (52%) to other caste (Table 2.3). Table 2.4 shows that the proportion of second and higher order births is the

highest in Mewat district (70.3%) and the lowest in Panchkula district (50.8%). The proportion of first order birth has crossed the mark of 45 percent in Panchkula, Ambala, Yamunanagar, Kurukshetra, Rewari and Gurgaon. Each of Hisar, Karnal and Sirsa contributes around 7 percent of the all births.

5.2 Mean Children Ever Born

Mean children ever born (CEB) to ever-married women aged 15-49 years is 2.3 with marginal differential by residence, while it is 2.9 for non-literate and 1.7 to women with at least 10 years of education. The completed fertility measured in terms of average children ever born to ever-married women aged 40-49 years is nearly 3. The differentials by caste/tribes are marginal and ranges between 2 children for 'other' caste and 2.5 children for scheduled caste. Similarly, differential by religion are wide and ranges between 2 children for Sikh to 3.2 for Muslim. The state level estimates for mean children ever born by sex of children are also shown. It indicates that on an average an excess of 0.3 male children to per female children ever born to ever-married women aged 15-49 years in the state as a whole. The sex differentials in mean children ever born to ever-married women aged 15-49 years is found to be higher for 'Others' caste group, non-literates and older women (age 40 years and above). In case of women 40-49 years, the sex differential in mean children ever born is measured as 0.36. In this age group of women, the gap between male and female mean children ever born is higher among scheduled tribes (0.39), other caste (0.37), and non-literate women (0.36) (Table 2.5).

Figure 5
Mean children ever born by districts

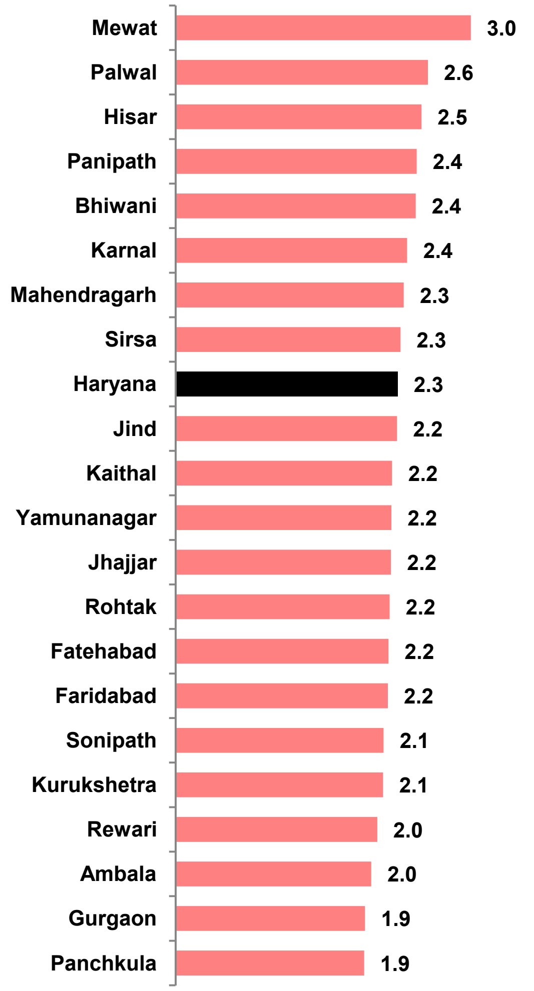

The mean children ever born to ever-married women by district of Haryana is shown in Figure 5, it varies from 1.9 children in Panchkula and Gurgaon districts to 3 children in Mewat district, while the state average is 2.3 children.

In Haryana, most of the outcomes (92%) of pregnancies which occurred since January 1, 2008 to currently married women aged 15-49 years turned to be live birth. Only 5 percent of the pregnancies outcomes are reported as spontaneous abortion. Percentage of pregnancies that resulted in induced abortion is 1.5 percent for the state as a whole. Interestingly, age of women and sex composition of children depict a large variation in the percentage of

pregnancies resulted as induced abortion. For example, women having one daughter (7.5%) or three daughters (4.9%) have relatively higher rate of spontaneous abortion. Similarly, women aged above 40 years have a relatively higher induced abortion rate than their younger counterparts (Table 2.6). Percentage of pregnancies resulted into spontaneous abortions varies from 0.8 percent in Gurgaon district to 10.1 percent in Mahendragarh district. The induced abortion rate (3.5%) and still birth rate (2.4%) are found to be highest in district Bhiwani and Sonipath respectively. As a result 92 percent of pregnancies since Jan 1, 2008, among in the low state, are reported as live births, which should draw attention of reproductive and child health programme managers (Table 2.7).

5.3 Fertility Intention and Sex Preferences for Additional Child

Fertility preferences of currently married women in terms of desire for an additional child, timing to have and preferred sex of desired additional child by number of living children are given in Table 2.8. Among those women with no living children, nearly 28 percent wanted a child soon (within the next two years) and around 9 percent wanted a child two or more years later. Among the currently married women aged 15-49 with one living child, 7 percent wanted an additional child soon i.e. within two years. Most of the currently married women with two living children are either sterilized (39.2%) or want no more children (45.7%). In addition, not more than 2 percent of women desired another child once they attain two or more surviving children.

Figure 6: Desire for the additional child/Next child

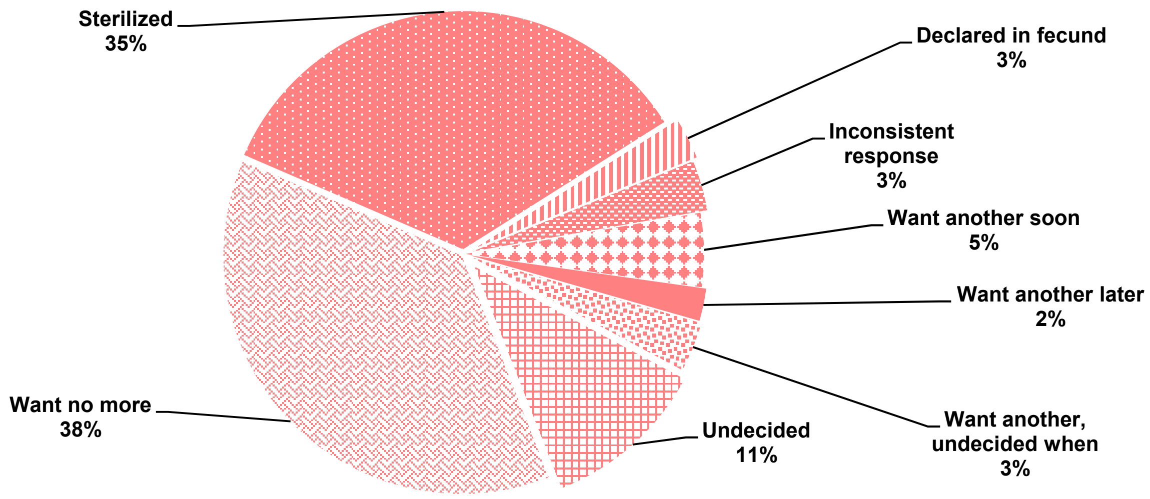

Figure 6 depicts the fertility preference of all currently married women regardless of number of living children. Thirty eight percent of currently married women want no more children, 5 percent want additional child soon, 11 percent are undecided about having an additional child and about 35 percent have undergone sterilization. Among the currently married women with no living children but wanted to have an additional child, 47.8 percent reported that sex of the child does not matter, 46.3 percent say it is up to God while 4.3 and 1.6 percent want to have an additional child as a boy and a girl respectively. Among those who had at least one living child and wanted to have another child, the percentage of women who were able to tell about preferred sex of additional child is quite high (21.2% preferance of male and 11.3% preferring female child). With increasing number of living children, longing for an additional

male child becomes more and more magnified from 21.2 percent among currently married women with one child to 43.9 percent among currently married women with four and more living children (Table 2.8).

6. MATERNAL HEALTH CARE

Maternal Health Care package of RCH programme focused more on ANC. The Maternal health care activities are implemented to strengthen and fulfill the RCH goals. ANC services provided by medical and paramedical professionals comprises of the regular physical checks with weight, height and blood pressure measure, Hemoglobin level test, consumption of IFA, Tetanus (TT) injection and growth status and position of fetus. These primary services are made compulsory to be provided during the ANC check up from health facility. At least four checkups are made compulsory to complete the full ANC course in order to prevent and protect women from pregnancy related complication faced during the pregnancy and till the delivery. Janani Suraksha Yojna (JSY) scheme is implemented in health facilities under the aegis of NRHM/NHM to promote the Institutional Delivery and post natal care to prevent from maternal deaths.

6.1 ANC by Selected Background Characteristics

In Haryana, 71 percent of the women had received at least one antenatal care (ANC) service during the pregnancy of their last birth in the three years period preceding the survey. Utilization of government health facility for ANC care is around 64 percent compare to 52 percent of private health facility (Table 3.1).

Any ANC coverage by selected background characteristics are illustrated in Figure 7. Any ANC received among the non-literate are 50 percent as against 82 percent among the women educated for 10 or more years. There is significant rural-urban gap of 10.5 percentage point in availing any ANC, with 78 percent among urban women and 67 percent among rural women. Women who had received ANC with one living child is 77 percent whereas women with 4 and above living children received 54 percent.

Figure 7
Progress in institutional delivery

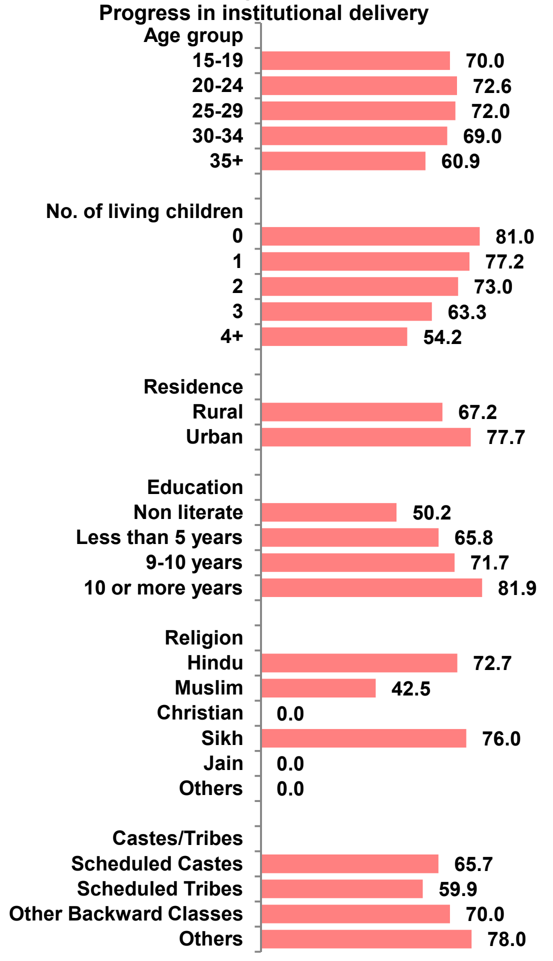

The coverage of any ANC is highest in Panchkula district (88%) and lowest in Mewat and Sonipath district (45.6%). Majority of women from Panchkula, Kaithal, Mahendragarh, Ambala, Mewat, Bhiwani and Jhajjar district are availed ANC care from government health facilities (71 to 87%). The lowest ANC coverage in government health

facilities was in Panipath district (29%), where women prefer more the private health facilities. The DLHS-4 data reveals that more women availed from government health facilities for ANC as compared to private health facilities.

The specific components of ANC checkup which are supposed to be received by the women during the pregnancy were asked to response. The proportion of women who received weight, height and blood pressure measurement, blood and urine tested, abdomen examined and sonography/ ultrasound test done are 61.2, 25.7, 41.1, 49.7, 51.9, 34.5 & 56.5 percent respectively (Table 3.3). One important feature of ANC check up in Haryana in case of ultrasound test done is high among women who are having one children than four or more living children (63.8% and 36.4% respectively), having ten years of education (68.3%), rural-urban residence (52.8% and 63.7% respectively), and Sikh religion (59%). The women from other caste are the highest (64.5%) as compared to all others caste (Table 3.3).

The proportion of women who had received at least three ANC (45%) and the women who had received first ANC in the first trimester of the pregnancy (50%) (Table 3.5A). The proportion of women who had three ANC are highest among women who have one living children (52%), having 10 years and above education (55%), urban residence (52%), Sikh religion (58%), other caste (52%). There is no much difference by age group between 15-35 years.

MAP 1
FULL ANTE-NATAL CHECK UP BY DISTRICTS

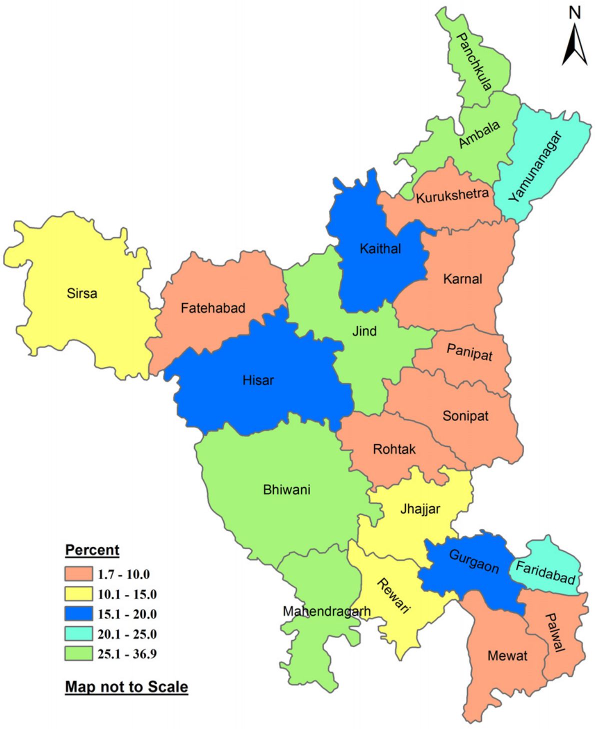

The women who had full ANC (i.e. at least 3 ANC visits with 100+ IFA tablets/ Syrups consumed at least 1 TT) in Haryana is 15 percent. But 58 percent of women had 2 TT+ injections against 20.9 percent who had consumed 100+ IFA tablets/Syrups. The proportion of women who had received full ANC is highest in Mahendragarh district (37%) and lowest

is 1.6 percent in Mewat district (Table 3.6). District wise variation in coverage of full ANC is shown in the Map 1. The proportions of women who consumed 100 IFA tablets/syrup and received at least one TT injection are 15 percent and 66 percent respectively in Haryana during DLHS-4 (Table 3.6).

6.2 Institutional Delivery

In Haryana, the institutional delivery increased from 26 percent in DLHS-1 (1998-99) to 35 percent in DLHS-2 (2002-04) to 47 percent in DLHS-3 (2007-08) and further substantially increased to 77 percent in DLHS-4 (2012-13). The institutional delivery in Haryana is presented in the Figure 8.

Figure 8
Progress in institutional delivery

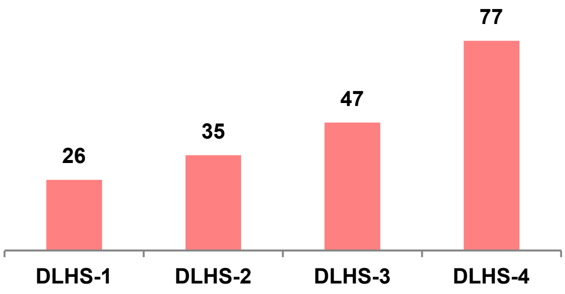

Around 77 percent of deliveries in the three years period preceding the survey which resulted either in still or live births were in both government and private health facilities,(Table 3.7).

MAP 2
INSTITUTIONAL DELIVERY BY DISTRICTS

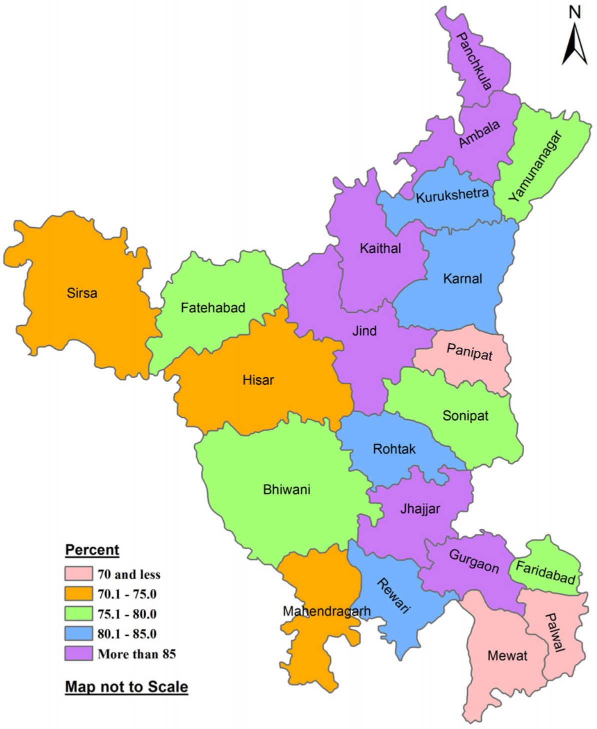

The proportion of women having background of ten years or more education (88%) and having one living child (86%) are going for institutional delivery. The percentage of institutional delivery ranges from 94 percent in Panchkula to 51 percent in Mewat districts (Table 3.9). The home delivery cases (22.5%) which are assisted by skilled persons is only 14.1 percent. The mean delivery cost in Haryana ranges with a maximum of Rs.10,309 in Mahendragarh district and minimum of Rs.3,648 in Gurgaon district. In Haryana, out of the

21 districts, only 4 districts are having the institutional delivery 90 percent and above and in remaining 17 districts the percentage is varies from 51 to 89 percent of institutional delivery.District wise variations in institutional delivery is presented in the Map 2.

In Haryana, 24.9 percent of institutional delivery used ambulance and 31.6 percent jeep or car for transportation of delivery with an average cost of Rs.1,058. The use of ambulance for transportation for institutional delivery was low among women with background of having zero living children (16%), 10 or more years of education (21.9%), Muslim (17.1%) and Other caste (21.8%). The mean delivery cost is Rs.3,464 in government health facilities and Rs.10,222 in private health facilities. There is a large variation of institutional delivery cost compare to government and private health facilities.

The JSY financial assistance for institutional delivery had benefitted to 8.3 percent and Home delivery 2.6 percent (Table 3.8). The highest benefitted women for institutional delivery are those in the age group of 25-29 years (8.8%), rural residence (9.5%), having 3 and above living children (11.1%), Hindus (8.4%) and scheduled caste (13.2%).

6.3 Complications during Pregnancy, Delivery and Post-delivery Period

Women who either do not take ANC or had an incomplete course of ANC are exposed to the risk of maternal death. In Haryana as much as 37.5 percent women who had still/live births in the three years preceding the survey had some complications during pregnancy (Table 3.6). Out of 21 districts, in 8 districts women faced pregnancy complication percentage ranges from 56 percent in Sonipath to 40.7 percent in Panchkula. The remaining 13 districts' women faced pregnancy complication ranges from 15.1 percent in Palwal to 40.0 percent in Kaithal. Forty five percent of women who had reported complication during pregnancy had sought treatment for the problem in Haryana (Table 3.15).

Around 42.9 percent of women in Haryana had faced at least one delivery complication. The main type of delivery complications experienced by women who had still or live births in the three years period preceding the survey are mainly premature labour (61%), obstructed labour (43.7%), prolonged labour (17.5%), excessive bleeding (10.7%) and convulsion or high blood pressure (5.6%). Delivery complications are higher among those who undergone by caesarean (54.6%) compared to normal delivery (41.7%) (Table 3.11). In all the districts of Haryana, in Yamunanagar district is the highest proportion of women who had a delivery complication (66.6%) and is lowest in Faridabad (27.7%) (Table 3.15).

In Haryana, around 15.2 percent women reported post-delivery complications. The major problem during post delivery period is high fever (52.4%), lower abdominal pain (49.5%) and followed by excessive bleeding (15.6%) (Table 3.12). Among the women who had post-delivery complications 53.3 percent had sought treatment (Table 3.15). Women sought treatment for post delivery complications are highest in Mahendragarh (86.2%) and lowest in Rohtak district (35.9%).

7. CHILD HEALTH AND IMMUNIZATION

To promote child survival and prevent infant mortality, NHM/NRHM envisages new born care, breastfeeding initiation, infant food supplementation at the right time, and a complete package of routine immunization for children. About 56 percent of newborns were examined

within 24 hours of birth (Table 4.1). In Haryana, women who availed newborn care from government health facility constitute 49.9 percent as compared to 42.9 percent from private, at home (6.4%) and others (0.7%). There was a substantial increase from DLHS-3 (22.9%). There is variation in rural areas in utilization of government health facilities (56%) and private health facilities (36.3%), whereas in urban areas it is 38.3 percent for government health facilities and 55.6 percent for private health facilities. Majority of women from Scheduled caste and Scheduled tribes communities check-up in government health facilities (60.5% & 55.2%) than in the private health facilities (32.4% & 31.6%). Other back ward classes also use government health facilities (52.9%) more than private 39.2 precent (Table 4.1).

Majority (84%) of children under 3 years of age (born after January 1, 2008) were fed with colostrums and there is not much variation across selected background characteristics of women (Table 4.2). Highest proportion of children being fed with colostrums (95.3%) is observed in Faridabad district and the lowest in Panipat district (73.9%) (Table 4.5).

In Haryana, only 50.9 percent of women had initiated breastfeeding within one hour of the birth of the child. Among the women, initiation of breastfeeding within one hour of birth is least practiced in Panipat district (32.2%) and most widely practiced in Rewari (69%). However, 82.8 percent of women in Haryana initiated breastfeeding within 24 hours of birth of their children, ranging from 66.2 percent in Panipat district to 97 percent in Faridabad (Table 4.5). The proportion of women who initiated breastfeeding within one hour, within 24 hours and after 24 hours of birth are 51, 83 and 8.5 percent respectively.

Duration of exclusive breast feeding practiced is high (among infant under 2 to 5 months old) and is ranged from 59.7 to 46.6 percent and it declines as the age increases in Haryana. The introduction of food supplementation with semi-solid and solid food started below the age of 2 months along with breastfeeding. As the age increases the percentage of the breast feeding declines and 19 percent of children under 24 to 35 months were breast fed along with other fluids, semi solid and solid foods (Table 4.3).

7.1 Immunization Coverage of Children (aged 12-23 Months)

Figure 9
Change in full immunization coverage of children

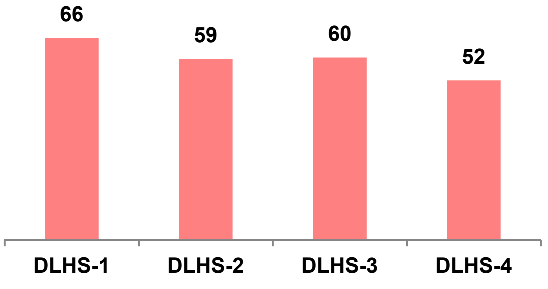

The immunization coverage of children (aged 12-23 months) has been recorded either from vaccination card or by asking the mother in case the card was not available. About 27 percent of children's immunization details was recorded from the vaccination card (Table 4.7). The full immunization coverage was 52.1 percent among children (aged 12-23 months). The full immunization comprises of BCG, three doses of DPT & Polio and measles vaccines (Table 4.6). In Haryana, the coverage of BCG and measles are 84 percent and 70 percent respectively. About 7 percent of children have not received any immunization.

In the districts of Haryana, the highest coverage of full immunization was in Kaithal with 74 percent and lowest in Mewat with 27.4 percent. Out of 21 districts of Haryana, 13 districts recorded full immunization coverage less than 60 percent whereas the remaining 8 districts had more than 60 percent coverage (Table 4.7). The coverage of BCG is above 75 percent in all districts except Sonipat (55%) and Mewat (53%). In case of measles the coverage fluctuates from 40.2 percent to 87.9 percent in the districts, for DPT it ranges from 50.2 percent to 93.6 percent and coverage of Polio 3 ranges from 45.2 percent to 86 percent. Higher proportion of children (58.4%) of women educated up to 10 years and above received full immunization. About 41 percent of children of Non-literate women's received full immunization (Table 4.6). In Haryana full immunization coverage of children (aged 12-23 months) in urban areas (54.5%) is higher than in rural areas (51%) as expected.

The coverage of full immunization decreased from DLHS-1 to DLHS-3 (from 66 to 60%) and has further decreased by eight percent point in DLHS-4 (52%) as depicted in figure 9. The coverage of full immunization of children is below 50 percent in Panipat, Rohtak Sonipat, Rohtak, Rewari, Gurgaon, Faridabad and Mewat while it is more than 50 percent in remaining districts (Table 4.7).

With regard to the place of vaccination of children, it was reported that 47.3 percent children received it from a Anganwadi-Centre, 10.6 percent from Sub-Health Centre and 33.2 percent from other government health facility (Table 4.8). District-wise variation in the coverage of full Immunization is depicted spatially in Map 3.

Proportion of children aged 9-35 months who received at least one dose of Vitamin-A supplement is 61.4 percent and who received 3-5 doses is 43.3 percent in the state (Table 4.9). In Karnal district 80.5 percent of children aged 12-35 months received at least one dose of Vitamin-A, while in Sonipat and Mewat districts less than 50 percent of children received Vitamin-A (Table 4.7). Panchkula, Kurukshetra, Kaithal and Mahendragarh are other districts of Haryana where coverage of Vitamin-A is more than 70 percent and in remaining districts the coverage of Vitamin-A is above 50 percent (Table 4.7). Higher proportion of children received one dose of vitamin A whose mother is educated 10 or more years (69.4%), living in urban area (64.4%) and belongs to other caste (63.9%) than their counterparts (Table 4.9).

About 58 percent of children in Haryana had received Hepatitis-B vaccination. There is not much difference between children living in urban and rural areas (Table 4.9).

MAP 3
FULL IMMUNIZATION COVERAGE OF CHILDREN (AGED 12-23 MONTHS) BY DISTRICTS

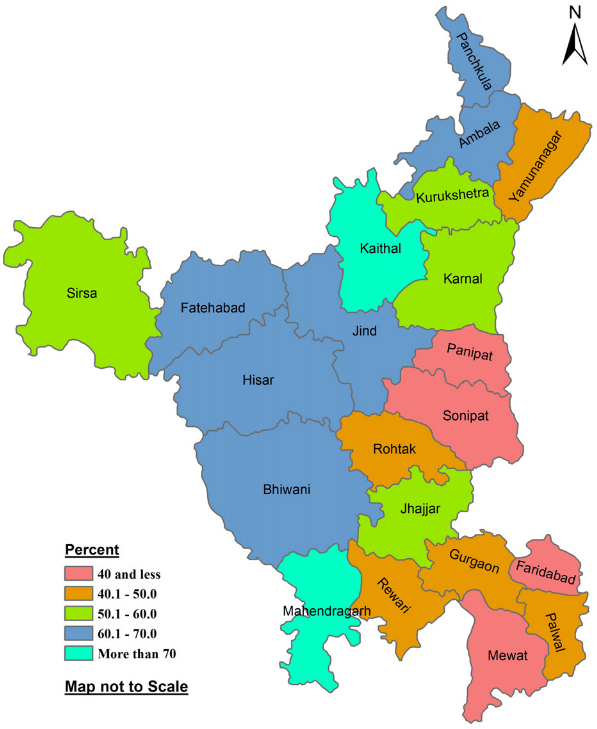

7.2 Management of Diarrhoea and Acute Respiratory Infection (ARI)

The information on knowledge of diarrhoea and ARI management was collected from women respondents as part of assessment of child care knowledge. Majority of women (82%) have knowledge of diarrhoea management (Table 4.10) and more than 75 percent of the women are aware of danger signs of ARI (Table 4.12).

The common practice followed by women for treatment of children who had diarrhoea was to give ORS (68%), salt and sugar solution (54%), plenty of fluids (32%), continue normal food (25%) and continue breastfeeding (15%) (Table 4.10). In Haryana, 45 percent children who suffered from diarrhoea were treated by ORS, while 69 percent of them were given some treatment or the other (Table 4.11). Majority (79%) of children who had suffered from diarrhoea are treated in a private health facility and 22.3 percent in a government health facility (Table 4.11).

In Haryana, 75.2 percent of women are aware of danger signs of ARI. Among them, 46 percent of women knew that difficulty in breathing, 37 percent knew pain in chest and productive cough, 27 percent consider rapid breathing, 24 percent knew wheezing/whistling, and 30.1 percent having knowledge of others signs of ARI (Table 4.12).

More than three percent children had suffered from ARI in the last two weeks prior to the survey. Out of total children suffered from ARI, 85 percent had sought advice/treatment. Majority (71%) of children had treatment at private health facility and only 30 percent went to government health facility (Table 4.12).

The prevalence of ARI among children varies from 0.3 percent in Ambala district to 7 percent in Bhiwani district. The treatment seeking for ARI or fever is 90 percent and more in the districts of Ambala, Yamunanagar, Kurukshetra, Sirsa, Bhiwani, Mahendragarh, Rewari and Faridabad (Table 4.13).

8. FAMILY PLANNING AND CONTRACEPTIVE USE

Family planning program in India has undergone sea changes in terms of strategies, focus and objectives. Post ICPD 1996 programme oriented has evolved itself in to human right framework keeping in mind the central point to reduce the unmet need for family planning. Strategies under NRHM were to create demand for family planning through enhancing child survival and improving maternal health.

Figure 10
Percent of currently married women using contraceptive methods

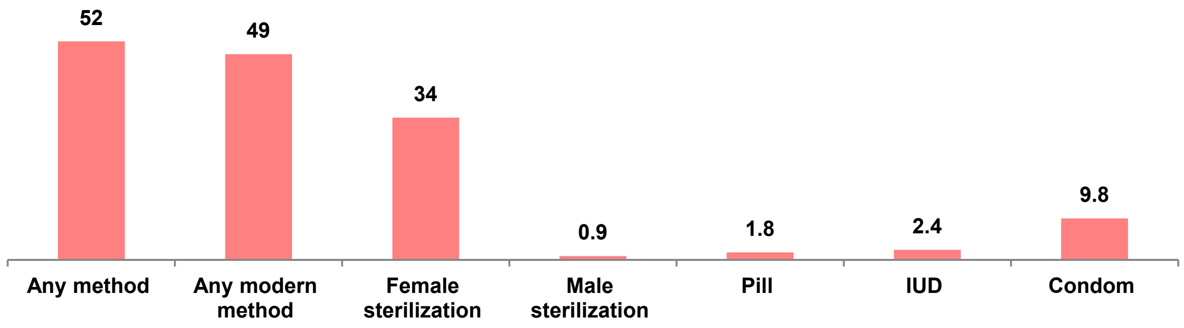

There is awareness to the extent of 84 percent about female sterilization but male sterilization is known to only 55 percent of women. Nearly half of the women know about IUD, Condom and Pills respectively. The knowledge of traditional method is 32 percent among women in Haryana. However new methods on menu of Indian program/or in market like female condom is known only 14 percent among women. Female sterilization is the predominant limiting method being used by 33 percent of currently married women in 15-49 years and popular male oriented spacing or temporary method is Condom ever being used by 14 percent of husbands of currently married women. Among the currently married women the proportion ever using any modern method is about 53 percent, while 55.3 percent of women ever used either modern or traditional methods. There is no substantial rural-urban difference in the ever use of any modern contraceptive use. However, female sterilization among rural women is 37 percent which comparatively more than among urban women with corresponding figures is 28 percent.

Figure 11
Change in contraceptive prevalance rate

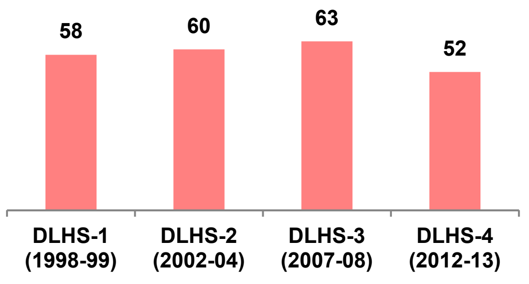

The status of current contraceptive use among currently married women or their husband shows that almost half of them were using one or other modern method mostly female sterilization (34%) at the time of survey. Condom is in use by 10 percent of currently married women's husbands which is same as it was in DLHS-3. The female sterilizations are more

among rural women (37%) and non-literate (44%) women compared to that among urban women (28%) and women educated for at least 10 years (22%).

Female sterilization regardless of family size is more among currently married women who have one or more living sons compared to those with no living son. Nearly 7 percent of women in 20-24 years, 20 percent of women in 25-29 years and about 34 percent of women in 30-34 years have been found to have undergone female sterilization at the time of survey. The mean age at the time sterilization is 29 years. Among the currently married women, proportion continuing IUD use for less than 2, 2-3 and more than 3 years are respectively 21, 13 and 52 percent respectively. Oral pill users continuing for more than 6 months constitute 59 percent of the total pill users and 64 percent of condom users are continuing for longer than 6 months.

Contraceptive prevalence rate (CPR) for any modern method is above 50 percent in following districts namely Panchkula, Ambala, Kaithal, Sonipat, Fatehabad, Sirsa, Hisar, Bhiwani, Rohtak, Mahendragarh which lies between 50 to 66 percent. Highest CPR for any modern method is 65 percent in Mahendragarh district and lowest is 26 percent in Palwal district. The prevalence of female sterilization in half of the total number of districts are above the state average of 34 percent. The contrast in the source of terminal and temporary methods of contraceptive is that 79 percent of sterilization has been done in government health facility and 24 percent have availed government health facility service for spacing methods. The high and low utilization rate of government health facility for limiting and spacing methods is true for all the districts of Haryana. Thirty five percent of sterilized women and wives of sterilized men got monetary compensation for sterilization, with variation of lowest 17 percent in Sonipat and highest 54 percent in Karnal district. As many as in 81 percent of sterilization cases monetary compensation is given at the time of discharge.

Nearly 11 percent of sterilized women, 20 and 15 percent, users of IUD and Pills were informed about the side effects before the adoption and 3, 4 and 2 percents of women using the aforesaid methods have experienced side effect or health problem. Among the currently married women who have discontinued contraception, the main reason cited is related to other method (43%) while 29 percent mentioned fertility related problems and 29 percent mentioned side effects. For the younger women in 15-29 years reasons for discontinuation of contraception are mostly fertility related.

About 6 percent of currently married women aged 15-49 years, not using any contraception intend to adopt limiting method and 3 percent spacing method in future. Those who intend to adopt either limiting or spacing methods in future within 12 months, after 12 months and still undecided about the timing constitute 36, 20 and 44 percents respectively.

Figure 12
Change in unmet need for contraception

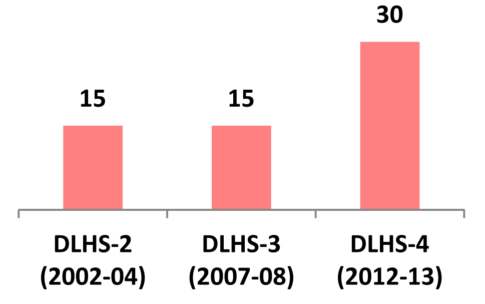

Unmet need for spacing includes the proportion of currently married women who are neither in menopause nor had hysterectomy nor are currently pregnant and who want more children after two years or later and are

currently not using any family planning method. The women who are not sure about whether and when to have next child are also included in unmet need for spacing. In Haryana 12 percent of currently married women have unmet need for spacing. The unmet need for spacing is 6 percent for women with 4 or more living children and 36, 29, 16 percents for women aged 15-19, 20-24 and 25-29. On the other hand, currently married women who are still have physiologically potential for conceiving and want no more children are categorized as having unmet need for limiting. The unmet need of contraceptive for limiting is about 19 percent in the state.

MAP 4
CONTRACEPTIVE PREVALENCE RATE FOR ANY METHOD BY DISTRICTS

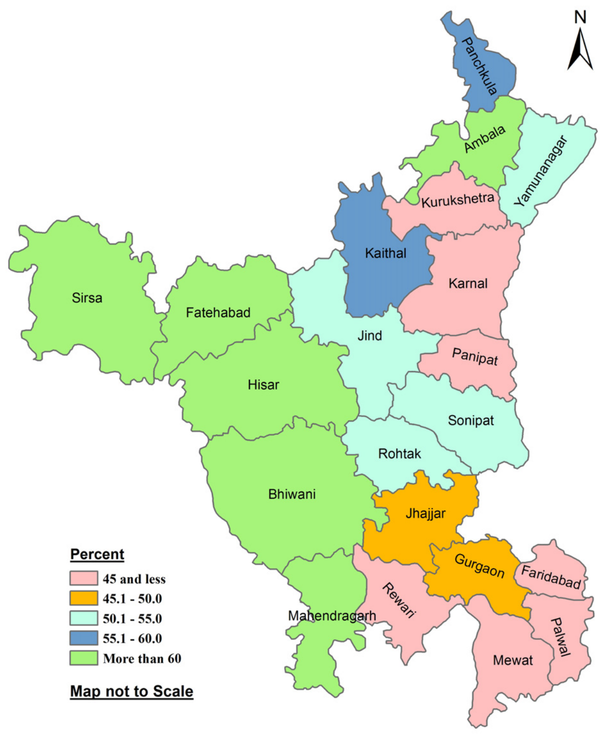

Currently married women with unmet need for spacing is highest in Sonipat and Mewat districts (19%) and lowest around 8 percent in Panchkula, Sirsa and Bhiwani districts. On the other hand unmet need for limiting is highest in Palwal (43%) and lowest is Hisar (7%).

The total unmet need of contraceptive has been almost constant in DLHS-2 to DLHS-3. It was 15 percent in 2002-04 and in 2007-08. In DLHS-4 (2012-13) it seems unmet need has substantially increased to 30 percent. This is basically due to higher unmet need for spacing among the younger cohort, a sign of decline in the desire for large family size. District wise contraceptive prevalence rate and unmet need are presented in figure 13.

Figure 13:
Contraceptive prevalance rate and unmet need by districts

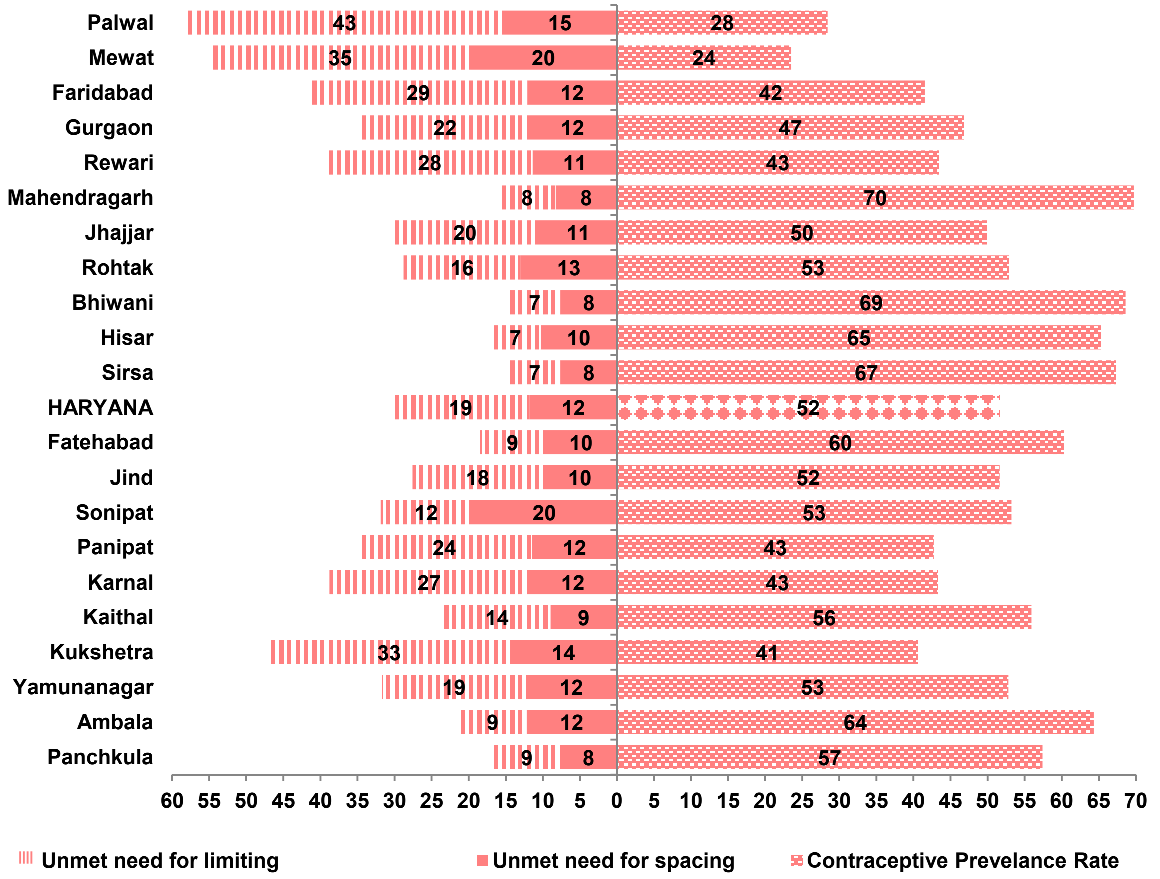

9. REPRODUCTIVE HEALTH

Reproductive health addresses the issues of reproductive processes, encompassing the functions and system at all stages of life. The reproductive health is the ability for the people to have a responsible, satisfying and safe sex life and have the capability to reproduce and the freedom to decide if when and how often to do so. This means that the right of men and women alike are to be informed of and to have access to safe, effective, affordable and acceptable methods of fertility regulation of their choice, and the right of access to appropriate health care services that will enable women to go safely through pregnancy and childbirth and provide couples with the best chance of having a healthy infant. The burden of diseases among women is due to reproductive function and system. The five main causes of the disease burden among women in developing countries are maternal, Sexually transmitted disease, tuberculosis, HIV infection, Depressive disorders. DLHS-4 has obtained information on awareness and prevalence of RTI/STI, HIV/AIDS, information and ways to avoid AIDS.

9.1 Menstruation Related Problems

The women reported to have menstruation related problems and have specific symptoms during three months preceding the survey by background characteristics is given in Table 6.1. Menstrual problems are experienced by 6.5 percent of women in Haryana. The problems of painful periods (48.5%) and irregular period (36%) are the main menstrual problems experienced by women. The other problems reported are frequent or short periods (15%),

prolonged bleeding (14%), scanty bleeding (7%), absences of periods (13%) and blood clots/excessive bleeding (4%). The differentials in menstruation related problems are found by age, place of residences and education of both husband and the women. Women who had consummated their marriage below 18 years of age have had more menstruation related problems (7%). The menstrual related problems also increased with duration of marriage. Around 7 percent of women had reported to have menstrual problems whose marital duration was 10-14 years and just above 7 percent for women whose marital duration was more than 15 years. The menstrual related problems did not differ drastically by caste and religion.

9.2 Awareness of RTI/STI

The awareness of RTI/STI was obtained from ever married women. The women who are aware about RTI/STI were further asked on the modes of transmission and symptoms of the disease. Table 6.2 shows the percentage of women who have heard of RTI/STI by background characteristics. About one-fourth of women in Haryana had heard of RTI/STI. The proportion of women who were aware of RTI/STI was comparatively higher in urban areas (34%) than in rural areas (20%). Awareness of RTI/STI was lower among young women, women with low age at consummation of marriage, non-literate and women from Scheduled tribes. Awareness of RTI/STI increases with education of women. Thirty seven percent of women who had completed ten or more years of schooling were aware about RTI/STI.

Television is an important source of knowledge about RTI/STI, 66 percent women reported they had heard about RTI/STI from TV. Forty five percent of women reported Leaders/Community meeting as source of knowledge of RTI/STI. Another major important sources of information about RTI/STI are from print media (39%), cinema (33%), health personnel (20%). The sources of knowledge about RTI/STI differ by education of women and husband.

9.3 Knowledge Regarding Mode of Transmission of RTI/STI

The knowledge regarding mode of transmission of RTI/STI was asked to women who had heard of RTI/STI (Table 6.3). About 43 percent of women reported unsafe delivery, 40 percent unsafe abortion, 33 percent unsafe IUD insertion, 30 percent unsafe sex with persons who have many partners and 21 percent who have unsafe sex with sex workers as a mode of transmission of RTI/STI. More than one fourth of the women reported that unsafe sex with homosexuals were also as mode of the transmission of RTI/STI. The knowledge varies by residence, age at consummation, education of women and education of the husband.

Table 6.4 shows the common symptoms of reproductive tract infections/sexually transmitted infections among women. More than 12 percent of ever married women have reported having symptoms related RTIs/STIs and 6 percent experienced abnormal vaginal discharge. The women reported itching or irritation over vulva (3%), pain in lower abdomen not related to menses (3%). More than 55 percent of women discussed the RTI/STI related problems with their husband or partner (Table 6.5). The women mostly sought treatment for RTI/STI (72%) from private health facility.

The women who have heard about RTI/STI varies from 7 percent in Panipath district to 56 percent in Panchkula district. The districts where more than one-third of the women have heard about RTI/STI are Kaithal, Jind, Hisar, Bhiwani, Jhajjar, Gurgaon and faridabad. Women reporting any abnormal vaginal discharge varied from 2 percent in Ambala district to 11 percent in Mahendragarh district. More than half of the women in five districts (Panchkula, Yamunanagar, Fatehabad, Sirsa and Mahendragarh) had Sought treatment for any RTI/STI including abnormal discharge.

9.4 Awareness of HIV/AIDS

The awareness on HIV/AIDS was asked to ever-married women age 15-49 years. More than 52 percent of the women had heard about HIV/AIDS. Television is the one of the major source of knowledge on HIV/AIDS. Ninety percent of women reported that TV is the main source of knowledge on HIV/AIDS, followed by cinema and Community's leaders meetings (43% each), print media (36%), school adult education programs (31%), health personal (22%), husband (6%) and radio (9%) (Table 6.7). More than half of the women reported unsafe sex with person having many partners, transfusion of infected blood (51%) and sharing of injection/needle (43%) as mode of transmission of HIV/AIDS (Table 6.8). The reported modes of transmission of HIV/AIDS differ by residence, education of women and husband.

Table 6.9 shows knowledge of methods of preventing HIV. About two-fifth of women were of the view that HIV/AIDS can be prevented by avoiding risks of getting infected through blood. Nearly one-third of women were of the opinion that by using condom correctly during each sexual intercourse and having sex with one uninfected partner can prevent HIV/AIDS. The differences in the preventing HIV/AIDs were found by residence, age at consummation of marriage, education of women and husband. The misconception about transmission of HIV/AIDS from mosquito, flea or bedbug was reported by 9 percent of women. The other misconception was sharing food (3.5%), stepping on someone’s urine/stool (4.7%), sharing clothes (3%), hugging and shaking hand (2.2%) respectively.

The women who had heard about HIV/AIDS were asked the place to test the HIV/AIDS (Table 6.11). More than two-fifth of the women know the place where the HIV/AIDS could be tested. The differences in the place of test were found by residence, age at consummation of marriage, education of women and husband. Forty three percent of women reported government hospital/dispensary and 46 percent reported private hospital/clinic as a place where people can go to test HIV/AIDS. The women who have heard about HIV/AIDS were asked if they had gone for the test. Thirteen percent of women had undergone for HIV/AIDS test (Table 6.12).

10. PERSONAL HABITS

Personal habits of adults (age 15 and above) such as consumption or abuse of tobacco and alcohol, and eating unhealthy foods are usually viewed from the lens of risk-taking behaviour due to their adverse health outcomes. The emerging morbidity pattern from the personal habits is a crucial predictor of current as well as future health status of a population. It has become increasingly important to understand and examine the impact, these habits have on

overall health status in India in the context of the epidemiologic and demographic transitions. Besides, the treatment seeking behaviours for these illnesses reflects the availability, accessibility, as well as quality of health care services. Studies have shown evidences of correlation between the shift towards non-communicable diseases (NCD) and increasing risk-taking behaviours among adult individuals. For instance, excessive drinking is linked to acute and chronic physical health problems, particularly those related to the heart, blood circulation, respiratory, diabetes, mental health, cancer, crime and disorder, domestic violence, unprotected sex, unintended pregnancy, etc., (Room, Baboor, and Rehm, (2005). Alcohol consumption contributes to many diseases and is now the fifth leading risk-factor for the global disease (Lim, Vos, Flaxman, et al, 2012). Also, the economic burden of these NCD is equally serious - i.e., a 10 percent rise in NCDs is found associated with 1 percent lower rates of annual economic growth. However, programmes to combat NCDs were tremendously underfunded, and a low priority policy, as it is not part of the millennium development goals (MDG).

The WHO has recently stated that NCD such as cancer, diabetes, and hypertension are largest causes of death, and by 2020 cardiovascular diseases will be the largest cause of death and disability, including developing countries like India (WHO 2010). In 2012, the UN conference on sustainable development (Rio+20), referred to non-communicable diseases as "one of the major challenges for sustainable development in the 21st century", emphasising the fundamental link between health and development. In the same year, the World Health Assembly endorsed an important new health goal: to reduce avoidable mortality from non-communicable diseases (NCDs) by 25% by 2025 (the 25 by 25 goal). The future threat to health is from NCDs, as the world also urbanizes faster than before.

Since 1990s India's overall health status has shown tremendous improvement, which signals a change and shift in pattern of morbidity and causes of death to non-communicable diseases (NCD), despite still substantial contribution of communicable diseases. The NCD accounted for 42 percent of all deaths in India (56% in urban areas and 40% in rural areas), as compared to communicable diseases with 38 percent (RGI, 2003). Estimated prevalence of diabetes, hypertension, ischemic heart diseases (IHD) and stroke is 62.5, 159.5, 37.0 and 1.54 per 1000 respectively. In the National Health Policy (NHP), the Government of India (GOI) has committed to eradicating infectious illnesses and reducing the mortality associated with such illness (MOHFW, 2002). One of the goals of the NHP 2002 is a 50 percent reduction of deaths from TB, malaria, and other vector and water borne diseases by the year 2010.

This chapter presents findings on the personal risk-taking habits, acute and chronic (infectious and non-communicable) diseases, and their treatment-seeking behaviours.

10.1 Tobacco and Alcohol Use in India

Tobacco and alcohol use have been associated with a wide range of major diseases, including several types of cancers and heart and lung diseases. Studies have shown that in addition to sharing the same health risks as men, women who use tobacco or alcohol also experience difficulty in becoming pregnant, are at an increased risk of infertility, pregnancy complications, premature births, low-birth-weight infants, stillbirths, and infant deaths (USDHHS, 2004).

In India, information about tobacco and alcohol use among adults has been collected by various household surveys such as the National Sample Survey (NSSO) (50th round, 1998), NFHS (1992-93, 1998-99, and 2005-06), DLHS (2007-08), the Global Adult Tobacco Survey-India (GATS-India, 2009-10), etc., each survey with specific objectives and methodology. According to GATS India, 35 percent of adults in India age 15 and over use tobacco in some form or the other, with higher use among adults in most North eastern region (39-67%), east (36-50%), and central (40-53%) (IIPS & MoH&FW, 2010).

In India, there are varieties of tobacco products and its use is also very diverse. The most common ways of tobacco use are the smoking and oral (smokeless) variety. Dominant among the smoking form include cigarettes and bidis, while in case of the oral use of smokeless tobacco, chewing or applying to the teeth or gums (scented/unscented, with paan) are the popular forms, which has also become very popular in most parts of the country. The DLHS-4 also collects information related to tobacco and alcohol usage directly from among the eligible adults (women age 15-49 and men age 15-54).

In case of alcohol consumption, household surveys in India show that alcohol use among adults, both men and women, is not uncommon, but the use is found much lesser than tobacco use. Alcohol consumption is higher among men than females. Study in India indicated the prevalence of current use of alcohol ranged from about 7 percent in Gujarat (state officially under prohibition) to a very high 75 percent in Arunachal Pradesh, and its consumption among women exceeded 5 percent only in the North Eastern region. DLHS-4 information on alcohol use by adult men and women comes from a substantial number of respondents (40,830 men and 46,415 women).

10.2 Tobacco Use in Haryana

As shown in Table 7.1, overall 16 percent of adults in Haryana use any kind of tobacco in one way or the other. However, tobacco use varies across the state by age, residence, education, region, etc. The survey clearly indicates that tobacco use is highest and more likely among men (29%), older persons age 40 plus (over 20%), non literates and people with less than 5 years of education (18-24%), rural residents (18%), and among scheduled caste (18%), etc.

In Haryana, the adults are using more of the smoking form (14%) as compared to the oral form (smokeless variety) of tobacco (3%). Use of smoke tobacco is higher among men (27%) as compared with females (2%), in rural areas (16%) and also more likely to be higher among the older adults aged 40 and above (19-22%), and those with less than 5 years of education (22%), and also among the scheduled caste (17%).

In general, it is observed that in Haryana use of tobacco among adults increases with age, but in contrast, declines with increase in educational status. The pattern of using tobacco among adults also shows an interesting scenario across the districts in Haryana. Of the 21 districts in the state, two districts, namely Karnal and Kuruksherta stand out as lower use of tobacco in the state, but it is highest in Fatehabad and Yamunanagar (about 6% each), followed by Ambala and Sirsa (5% each) in the state. In case of smoking form of tobacco the use among adults ranges from 9 percent in Fatehabad and yamunanagar to 23 percent in Bhiwani (Table

7.4). Other districts in which at least 15-20 percent of adults are likely to smoke are Kaithal, Panipat, Jind, Hisar, Jhajjar, Mahendragarh, Gurgaon, Mewat and Palwal.

The use of tobacco (all forms) among men is not substantially high in Haryana (5% for oral or smokeless and 27% for smoking). It is interesting to note that both forms of tobacco use is the lowest among teenagers (1% for smokeless and 3% for smoking), but increases sharply from age 20 onwards (among youth) to older ages. Smoking is more common among men in rural areas (30%) than urban males (22%). Among men also, age and education emerge as important factors. The older males say age 45 and above are more likely (6% for smokeless and 38% for smoking) to use tobacco products than those aged less than 20 (1% for smokeless and 3% for smoking). Similarly, as education level increases use of tobacco in any form is more likely to diminish among men. The more educated males are nearly two to three times less likely to use tobacco than their counterparts who are illiterate (Table 7.2).

In Haryana, strictly speaking, smoking form of tobacco is used widely by men (about 27%) than oral or smokeless form (5%). Around 2 percent of adult men use tobacco with paan, while 3 percent use other forms of tobacco. About 19 percent of men are usual smokers (smoke at least once a day) in Haryana. The proportion of usual smokers is 22 percent in rural areas as compared with 15 percent in urban areas (Table 7.6).

Generally, use of tobacco is found to be very less among women, more so when compared with men. However, in Haryana (Table 7.1), a small proportion of adult women (2%) reported using any kind of tobacco. Among the women tobacco users, while only about 2 percent smoke, a very small proportion of them (below 1%) use the oral form or chew tobacco (Table 7.3).

The pattern of tobacco use observed among women is similar to that found among men. For instance, majority of women tobacco users prefer the smoking form and they belong to rural areas (3%), as compared with urban women (about 1%). As presented in Tables 7.3, women tobacco users are mostly to be over age 30 and those with less education. Clearly, age and education play an important role in influencing such personal habits. Among those women who use non-smoke form/chew tobacco, below 1 percent use betel nut or paan (Table 7.5). Among women who smoke, just about 1 percent are usual smokers (smoke at least once a day), which is found to be higher in rural areas (Table 7.6).

10.3 Use of Alcohol in Haryana

Household surveys in India show that alcohol use among adults, both men and women, is not uncommon, but the use is found much lesser than tobacco use. Alcohol consumption is higher among men than women. Study in India indicated the prevalence of current use of alcohol ranged from about 7 percent in Gujarat (state officially under prohibition) to a very high 75 percent in Arunachal Pradesh, and its consumption among women exceeded 5 percent only in the North Eastern region. DLHS-4 information on alcohol use by adult men and women comes from a substantial number of respondents (40,830 men and 46,415 women).

As presented in Table 7.1, in Haryana 10 percent of adults consume alcohol. In the state, the level of consumption is found much higher among adults age above 30 (13-16%), in rural areas (11%), with less than 5 years of education (14%), and highest among scheduled caste

(12%). The proportion of men consuming alcohol is much higher (20%) as compared with women (below 1%). Unlike use of tobacco, education does not make much impact as an important factor. Undoubtedly, use of alcohol is higher among adults with lesser education, as compared to the more educated persons but the proportions do not differ drastically. The level of alcohol consumption by religious affiliation shows that the least consumption is among Jains (3%) and highest among 'Others' (18%).

In Haryana, alcohol consumption across the districts shows that of the 21 districts, only in 10 districts the level is less than 10 percent (Table 7.4). The prevalence of alcohol use across the state ranges from 5 percent in Mewat to about 19 percent in Gurgaon. Consumption of alcohol is found high in districts such as Bhiwani (14%), followed by Panchkula, Jind, Jhajjar, Faridabad and Kaithal (around 13% each), Sirsa (12%), Ambala (11%) and Hisar and Rohtak (about 10% each). Districts with less than 10 percent of adults consuming alcohol are Yamunanagar, Kuruksherta, Karnal, Panipat, Sonipat, Fatehabad, Mahendragarh, Rewari, Mewat and Palwal.

In Haryana, consumption of alcohol is found more concentrated among males (20%), much higher than among females (below 1%). Men who are more likely to consume alcohol are those in their 30's and 40's (around 25%), non-literates (27%), who follow 'Other' religions (28%) and scheduled caste (22%). The reporting of consumption of alcohol is seen among the teenagers (2%). It is interesting to note that more or less equal proportion of men in both rural and urban areas consume alcohol (around 20%).

Only about 1 percent of adult females reported consuming alcohol in Haryana (Table 7.3). The consumption of alcohol increases by age, with higher intake among older females age 45 and above (0.8%). A very small proportion of adult women (0.2%) reported as being a usual drinker.

11. MORBIDITY STATUS

In DLHS-4, for the first time, information on morbidity status of the household members was collected from the household respondent. The main objective is to get a somewhat fair idea about the prevalence of both acute illnesses (suffered for a week) and chronic illnesses (for a month or more), including disability (current) and injury (in last one year), suffered by any household member prior to the survey. Respondents were asked about occurrences of such illnesses among the household members, and to name the illness, including those diagnosed. Further, in case of occurrence of any disability, injury or illness, respondents were also asked about the nature of care sought, the type and place of health facility where treatment was done.

Depending on the nature and duration, all the illnesses or diseases are classified as (a) acute, and (b) chronic. Acute illness refers to those that occur suddenly with severe symptoms for short period during the last 15 days prior to the survey. Example includes diarrhoea, dysentery, acute respiratory tract infection (ARI), jaundice with fever, fever with chill/rigors/malaria, fever with rash, reproductive tract infections (RTI), etc. In case of chronic illness, those symptoms that persist for longer than one month in the past one year

prior to the survey. The list provided includes both symptoms and associated diseases categories.

11.1 Disability and Injury

From each of the selected household, DLHS-4 collected information from the head of the household or adult respondent on any injury and on five specific disabilities that household members may have suffered from such as mental, visual, hearing, speech, and locomotor. As it is difficult to capture the type of injury and its severity from lay reporting, assessment is made indirectly from the type and duration of hospitalization required for the injury.

As presented in Table 7.8, in Haryana about 2 percent of the sample population reported suffering from any injury. The prevalence of any injury is a little higher in the rural areas (1.9%) compared to urban areas (1.4%). The prevalence of any injury is observed to be higher in males (1.8%) as compared with females (1.3%) in the state.

About 4 percent of the injuries reported were treated in intensive care. However, 13 percent of injuries were treated as in-patient with stay for less than a week, and 9 percent reported they treated as in-patient with stay for more than 2 weeks. Interestingly, in Haryana, about 67 percent of injuries were treated using other form of treatments, i.e. other than intensive care or staying/in-patient, such as out-patient, traditional healers, or home remedies. More of females go for other treatments (68%) as compared with males. There is not much variation observed in terms of treatment of any injury by residence in Haryana.

In Haryana, among the five disabilities, the prevalence of visual disability is a little higher (0.3%) as compared to other disabilities. Mental, Speech and hearing disabilities are the other disabilities reported in Haryana (0.2%, 0.1% and 0.1% respectively). The prevalence of all the disabilities is more or less similar between men and women. No variation is observed in all the disabilities between the rural and urban areas (Table 7.9).

11.2 Reported Illnesses: Acute and Chronic

In order to assess the prevalence of illnesses from the selected household level in DLHS-4, the household respondents were first asked if any member of their households had suffered from any illness in the past one month or year. If reported that someone had suffered, more detail of the illness recorded, including main source of treatment. As mentioned earlier, all the illnesses are classified into (a) acute and (b) chronic, based on the nature and duration, and the information is collected from head or any adult member of the household.

Acute Illnesses

The prevalence of acute illness at the household level in Haryana is 13 percent. The differential in the prevalence of acute illness by residence shows little variation, with a higher rate in the rural areas (13.4 against 12.5 in urban areas).

About 13 percent of household members reported suffering from any acute illness in Haryana, and more of women (14%) than men (13%) reported to have suffered from any acute illness. Among the prevailing acute illnesses, fever (other than those with rash or jaundice) is reported by nearly 38 percent, followed by acute respiratory tract infection (ARTI) (27%), malaria (13%), diarrhoea/dysentery (4%) and fever with rash (3%).

Interestingly, more men are suffered from acute respiratory tract infection (ARTI) than women, whereas a higher proportion of women (39%) reported as being suffered with other type of fever than men (37%). Prevalence of acute respiratory tract infection (ARTI) is found higher in urban areas, whereas malaria and other type of fever is found to be higher in rural areas.

Nearly everyone who had suffered from any acute illness sought treatment. Among those who had sought treatment, 80 percent preferred treatment at private facility, mainly in a clinic/dispensary (61%), followed by hospital (19%). About 10 percent of those who had acute illnesses got treated in a government facility, mainly in a hospital (7%). About 3 percent with any acute illnesses were treated at DOTS centre or at home. In Haryana, use of private health facility for treatment of acute illness is quite common even in rural areas (81%), and more or less equally accessed by both males and females.

Chronic Illnesses

Survey results of chronic illnesses described pertain to prevalence, type, and source of treatment by sex and residence. In Haryana about 15 percent of the households reported a member suffering from chronic illnesses that lasted for over a month in the past one year prior to the survey (Table 7.8). As shown in Table 7.13, reporting by symptoms of chronic illnesses suffered by household members is highest for diseases of the musculoskeletal system (24%), followed by diseases of respiratory system (10%) and gastrointestinal system (7%). Diseases related to cardiovascular system, central nervous system and skin account for about 5 percent respectively. Interestingly, reporting of symptoms of chronic diseases other than the twelve identified diseases account for 32 percent.

In Haryana, not much differences observed in the reporting of symptoms of chronic diseases between males and females, except in case of some diseases. For instance, more males reported symptoms related to respiratory system (12% as compared with 8% among females), cardiovascular system (7% versus 4% by females) and skin diseases (6% against 4% among females). In contrast, females reported more of symptoms related to disease of musculoskeletal system (28% compared to 18% among males).

By and large, most chronic illnesses show more or less similar prevalence in both rural and urban areas. However, some of these chronic illnesses show slightly higher prevalence either in rural or urban areas. For instance, urban residents reported more of diseases of cardiovascular system (6%) and 'others' (39%) than rural residents. In case of rural areas, much higher reporting related to diseases of respiratory system (10%), musculoskeletal system (25%), gastrointestinal system (8%), and skin (6%) is observed.

Household respondents were also asked about the nature and source of treatment for chronic illnesses suffered by any of their household member. In Haryana, only 55 percent of those who suffered from chronic illnesses have details of diagnosis or treatment. About one-third has no details of diagnosis or treatment, and about 14 percent do not sought treatment at all. The proportion not seeking treatment (16%) or with no details of treatment (33%) is more in the rural areas as compared to urban areas (12% and 29% respectively). Overall, 51 percent of rural residents and 61 percent of urban residents have details of diagnosis or treatment for

the chronic illnesses. It is also observed that a higher proportion of males (57%) have details of diagnosis or treatment than females (53%).

Interestingly, in Haryana most people who suffered from chronic illnesses sought treatment at private facility (78%), which is observed to be equally accessible for both urban and rural residents. About 19 percent were treated at government facility, while 1 percent reported being treated at home, and 2 percent sought other form of treatment.

Persons who sought treatment for chronic illnesses were also asked about the details of the diagnoses at the facility. In Haryana, hypertension (12%) and diabetes (8%) are the most commonly diagnosed chronic illnesses, followed by diseases related to heart and asthma or chronic respiratory failure (around 4% each). Goitre accounts for about 1 percent of the diagnosed chronic illnesses, and about 1 percent with tuberculosis (TB). As expected, the proportion diagnosed with these chronic illnesses is much higher in urban areas, particularly hypertension and diabetes. The results show that more females suffered from hypertension (14% against 10% among males), while men are more suffered from diabetes (10% against 6% among females), diseases related to heart (6% against 3% among females), asthma or chronic respiratory failure (4% against 3% among females) and tuberculosis (2% against 1%).

Contrast to situation in the general population, among the older persons age 60 and above the prevalence of most chronic illnesses is much higher. For instance, about 14 percent of older persons were diagnosed with hypertension, 11 percent with diabetes, and about 6 percent each with diseases related to heart and asthma or chronic respiratory failure (Table 7.15). The prevalence of some of these chronic illnesses indicates that higher proportion of older persons in urban areas suffered from hypertension (19% as compared with 12% in rural areas), diabetes (19% against 7% in rural areas) and diseases related to heart (7% against 6%), while in rural areas a higher proportion older persons reported as being suffered from asthma or chronic respiratory failure (7% as compared with 4% in urban areas) and tuberculosis (2% against 1%). Similar to the situation in the general population, among the older persons also it is found that more females are diagnosed with hypertension (16% as compared with 13% among males). More of male older persons are diagnosed with diabetes, diseases related to heart, asthma or chronic respiratory failure, TB and stroke. The proportion of older persons diagnosed with these chronic illnesses is much higher in urban areas, particularly hypertension and diabetes.

11.3 Tuberculosis (TB)

Tuberculosis has re-emerged as a major public health problem in many parts of the world, often as a concomitant illness to HIV/AIDS. Tuberculosis, once known as the 'White Plague', is contagious and spreads through droplets that can travel through the air when a person with the infection coughs, talks, or sneezes. Today, TB is a leading cause of death among people who are HIV-positive. Worldwide, an estimated one-third of the nearly 40 million people living with HIV/AIDS are co-infected with TB. In most developing countries, TB would continue to be a serious health threat even in the absence of HIV/AIDS due to the public health challenges posed by poverty, high illiteracy, and poor sanitation. The GOI has stated that 'In 2005, a total of 97 percent population was covered under the Revised National

Tuberculosis Programme.' The government allocated Rs. 680 crores for the National Tuberculosis Control Programme (NTCP) in the 10th Plan (DGHS and WHO, 2005).

In Haryana, about 1 percent of the household population diagnosed with TB, which is found higher among males and in rural areas.

12. NUTRITION AND HEALTH

The DLHS-4 collected data on the nutritional status of children by measuring the height and weight of all children under age five in the selected households. The nutritional status assessment helps to identify sub groups of child population that face increase risk of faltered growth and potential health risks and vulnerabilities. The nutritional status of children in the survey population is compared with WHO child growth standards, which are based on an international sample of ethnically, culturally and genetically diverse healthy children living under optimum condition that are conducive to achieving a Child's full genetic growth potential (WHO, 2006)[1]. These standards can therefore be used to assess nutritional status of children all over the world, regardless of ethnicity, social and economic influence and child feeding practices. Accordingly, three standard indices of physical growth that describes the nutritional status of children are height-for-age (stunting), weight-for-height (wasting) and weight-for-age (underweight). Each of these indices provides different information about growth and body composition that can be used to assess nutritional status.

In DLHS-4, all children listed in the household, who were born in year 2008 or later were eligible for measurement of their height and weight. Thus, height and weight measurements were collected even from those children whose mothers may not have been interviewed in the survey. For this purpose, all the survey team carried with them two scales and two height boards, which were standardized in all aspects and calibrated for accuracy. Recumbent length was recorded for children under age two years. Standing height was measured for all other children. Table 8.1 represents percentage of children below age five classified as malnourished according to three anthropometric indices of nutritional status (height for age, weight for height and weight for age) by some selected background characteristics. The analysis is based on information collected from 12153 children from Haryana for whom complete and erodible anthropometric and age data are available.

12.1 Height-for-Age (Stunting)

Height-for-age measures linear growth. A child who is more than two standard deviations below the median (-2SD) of the WHO reference population in terms of height-for-age is considered short for his or her age are stunted. This condition reflects the cumulative effect of chronic malnutrition. If a child is below three standard deviations (-3SD) from the reference median, he or she is considered to be severely stunted. In Haryana 32 percent children under age five are stunted and more than 15 percent are severely stunted. Variation in the prevalence of stunting by age group shows that stunting is highest (42%) in children age 19-24 months, followed by those in age 25-35 months (37%) and the lowest (12%) in children

---

[1] World Health Organization (WHO) Multicentre Growth Reference Study Group. 2006. *WHO Child Growth Standards: Length/Height-for-Age, Weight-for-Length, Weight-for-Height and Body Mass Indexfor-Age: Methods and Development*. Geneva, Switzerland: WHO.

below age 6 months. Prevalence of severe stunting shows a similar pattern, with the height proportion of severe stunting in children age 19-24 months (30%), followed by among those age 25-35 months (19%). Sex differential in the prevalence of stunting is not pronounced as both male and female children are equally likely to be stunted (32% each). The sex differential remains by and large the same even in ease of severe stunting. Children under age five living in rural areas and coming from schedule caste families as more likely to be stunted than others.

The prevalence of stunting is not uniform across different districts in Haryana. Stunting is the lowest in Mewat district (7%) followed by Karnal and Rewari (17% each). While, the prevalence of stunting is the highest in Kaithal (59%) followed by Ambala (48%). Severe stunting is the lowest in Mewat (3%), followed by Karnal and Panipat (9%). On the other hand, Kaithal portrays the highest prevalence even in case of severe stunting.

12.2 Weight-for-Height (Wasting)

Weight-for-height describes the current nutritional status. A child who is more than two standard deviations below (-2SD) the reference median for weight-for-height is considered to be too thin for his or her height, or wasted. This condition reflects acute or recent nutritional deficit. As with stunting, wasting is considered sever if the child is more than three standard deviations below the reference median. Overall 32 percent children in Haryana are wasted and 19 percent are severely wasted. Analysis by age group shows that wasting rages from a minimum 29 percent in children age 19-24 months to the maximum 40 percentage in children in age 0-6 months. Children residing in rural areas are more likely to be wasted (34%) than children living in urban areas (31%). Children from scheduled caste are more likely to be wasted (35%) than the children in other caste groups. Variations by district portray that wasting in children ranges from 15 percent in each of Mahendragarh to 46 percent in Karnal.

12.3 Weight-for-Age (Underweight)

Weight-for-age is a composite index of weight-for-height and height-for-age. Thus, it is does not distinguish between acute malnutrition (wasting) and chronic malnutrition (stunting). A child can be underweight for his age because he or she is stunted, because he or she is wasted, or both.

Table 8.1 reveals that 36 percent of children under age 5 are underweight and 15 percent are severely underweight. The proportion of underweight children is the highest (40%) among children age 25-35 months and the lowest (27%) among children age 7-12 months. The sex differential in the proportion of underweight children is not so much pronounced. Rural children are more likely to be underweight (37%) than the urban children (35%). Children from scheduled tribes are relatively more likely to be underweight than other caste groups. By districts, proportion of underweight children ranges from 52 percent in Kaithal to 20 percent in Mewat.

12.4 Body Mass Index of Women

In many countries, chronic energy deficiency characterized by BMI of less than 18.5 among adults remains the predominant problem, leading to low productivity and reduced resistance to illness. Prevalence of overweight among women is also growing problem in developing countries. Overweight individuals are predisposed to a wide range of health problem including diabetes and heart diseases and also poor birth outcomes for pregnant women. The BMI is used to measure thinness or obesity. It is defined as weight in kilograms divided by height in meters squared (Kg/m2). A BMI of less than 18.5 is used to define thinness or acute under nutrition. A BMI of 25 or above usually indicates overweight and a BMI of 30 or above indicates obesity.

In DLHS-4, height and weight measurements in Haryana were obtained for 29,378 women age 15-49 years who were present in the sample households at the time of survey. Table 8.3 presents percentage of women age 15-49 by their BMI. The mean BMI is 22.5, which falls in the normal BMI classification. Around 59 percent of the women age 15-49 have a normal BMI , 22 percent are undernourished or thin (BMI less than 18.5) and 19 percent are overweight or obese (BMI 25 or higher). It is evident from the table that there is profound variation in BMI by some selected background characteristics of the women. The women age 15-19 are more likely to be thin are or undernourished (44%) than women in other age cohorts. Rural women are more likely to be thin or undernourished (25%) than their urban counterpart (17%), whereas urban women are more likely to be overweight or obese as compared to rural women (25% and 16% respectively). Educational attainment doesn't show any consistent relationship with the proportion of thin or undernourished women. Among women who are non literate, nearly one fifth of them (21%) are thin or underweight and 18 percent are overweight or obese. The percentage of overweight or obese is highest among women having more than 10 years of schooling. The women from scheduled caste households, having larger potential to have food insecurity, are comparatively more likely to be thin in comparison to those households from rest caste-groups. Proportion of the women who are thin or underweight is not uniform across districts of Haryana. It ranges from the minimum of 13 percent in Kurukshetra and Faridabad to 29 percent in Hisar. On the other hand, proportion of ever married women who are overweight or obese is the highest in Sonipat (27%) and lowest in Mewat.

12.5 Prevalence of Anaemia

Anaemia, characterized by a low level of hemoglobin in the blood, is major health problem in developing countries, especially among young children and pregnant women. Anaemia among pregnant women may be an underlying cause of maternal mortality, spontaneous abortion, premature births, and low birth weight. The most common cause of anaemia is inadequate dietary intake of nutrients necessary for synthesis of hemoglobin, such as iron, folic acid, and vitamin B12. Anaemia also results from sickle cell disease, malaria, and parasitic infections (Benoist et al. 2008)[2]. It is against this background, a number of

---

[2] Benoist, B. D., E. McLean, I. Egli, I., and M. Cogswell (eds.). 2008. *Worldwide Prevalence of Anaemia 1993–2005: WHO Global Database on Anaemia*. Geneva, Switzerland: World Health Organization

interventions have been put in place to address anaemia in children in developing countries. These include expanded distribution of iron supplements and deworming medication to children age 1-5 every six months.

In DLHS-4, all the usual residents of the selected households including children age 6-59 months were included in the anaemia testing, where blood drops were collected using dried blood spot (DBS) method and tested in designated laboratories. The process of blood collection consists of obtaining blood droplets by pricking in the middle or ring finger with a retractable and non-reusable lancet. Before pricking, the finger was cleaned with a swab containing 70 percent isopropyl alcohol and allowed to dry. In case of those children where blood droplets were not possible from middle or ring finger, heel pricking was practiced and DBS were prepared.

Table 8.5 shows the anaemia status of children age 6-59 months by some selected background characteristics. Over three-fifths (63%) of children age 6-59 months suffer from some level of anaemia (Hb <11.0g/dl), 16 percent of children have mild anaemia and 38 percent have moderate anaemia (Hb 7.0-9.9g/dl). About nine percent of children age 6-59 months have severe anaemia (Hb <7.0 g/dl). There is no differential in prevalence of anaemia among children age 6-59 months by sex of child and place of residence. The prevalence any anaemia does not vary significantly by caste of the household to which children belong to. Of course, Sikh's children are comparatively less likely to suffer from any anaemia than those from other religion.

The prevalence of any anaemia declines sharply among school going population age 6-19 years (56%). The proportions of school going population age 6-19 years who suffer from mild and moderate anaemia are 17 and 33 percent respectively. The pattern in decline in any anaemia with increasing age is linear in nature with the maximum (58%) among those in the age-group 6 to 10 years and minimum (53%) in those age 17-19 years. Female children and those living in rural areas are more likely to be anaemic (Table 8.6). The prevalence in anaemia also declining linearly with increasing years of schooling, from 59 percent among non-literate to 52 percent among those having 10 or more years of schooling. Scheduled caste are also more likely to be anaemic even in the schooling going population age 6-19 years.

Prevalence of anaemia declines further in case of adult population age 20 years and above. It is evident from Table 8.7 that over fifty percent (53%) of adult age 20 years and above are anaemic in Haryana. Adult women are approximate 1.2 times more likely to be anaemic than their male counterparts. Likewise to the school age population (age 6-19), years adults population portrays a decreasing prevalence of anaemia with increasing age groups. There is no difference in the prevalence of anaemia by place of residence (Table 8.7). Increasing years of schooling shows a linear decline in the prevalence of anaemia but the pattern gets changed in case of males and females where prevalence of anaemia remains by and large the same (47% to 50%) among males and (56% to 59%) females respectively across the different years of schooling. Islam religion portrays a distinct pattern with relatively higher prevalence of anaemia even among adult age 20 years and above, which may have definite implication for the food security scheme in the state.

Prevalence of anaemia among pregnant women poses much severe health consequences and may be an underlying course of maternal mortality, spontaneous abortion, premature births and low birth weight. Table 8.9 presents the percent of pregnant of women age 15-49 classified as having iron-deficiency (anaemia) by degree of anaemia and some selected background characteristics.

It is evident from the table that around three-fifths (60%) of pregnant women in Haryana are anaemic. About seventeen percent of them have mild anaemia (10.0-10.9 g/dl), 36 percent have moderate anaemia and 7 percent have severe anaemia. Pregnant women in Haryana who are younger in age (specifically age 15-29), those living in rural areas, and coming from other backward caste households are more likely to be anaemic.

12.6 Prevalence of Diabetes

Diabetes has serious consequences for individuals and poses a large burden on health services, especially in developing countries. According to the International Diabetes Federation (IDF), diabetes poses a daunting challenge to the sustainable development of the nation, as almost every tenth adult in India is estimated to be affected by either diabetes or pre-diabetes (IDF 2011). The latest global figures on diabetes, released by the International Diabetes Federation (IDF), has raised a serious alarm for India by saying that nearly 52% of Indians aren't aware that they are suffering from high blood sugar (IDF, 2011)[3]. In DLHS-4, women and men age 18 and older in the selected households were eligible to have their blood glucose level tested. The blood glucose was measured using portable glucometer namely SD code free, where blood droplets were obtained by pricking in the middle or ring finger with a retractable and non-reusable lancet. Before pricking, the finger was cleaned with a swab containing 70 percent isopropyl alcohol and allowed to dry. In the process, the first two drops of blood were wiped away and third drop was drawn into the glucose strips.

Table 8.10 and 8.12 present data on random blood glucose values for men and women age 18 and above from the sample households included in DLHS-4. Data shows that 6 percent of men and women age 18 and above in Haryana suffer from diabetics as the level of blood glucose among those have been 160mg/dL or higher. Another 9 percent of men and women age 18 and above in Haryana are pre-diabetic. However, over four-fifth of men as well as women have normal level or even lower level of blood glucose.

Prevalence of diabetes increases linearly with increasing age among men as well as among women age 18 years and above. Men age 60 & above are over 8 times more likely to suffer with diabetes as compared to those in the age-group 18-29 years. The pattern remains by and large same even among women age 18 years and above - 1.8 percent in the age group 18-29 years and 13.3 percent in age 60 years & above (Table 8.12). Men and women age 18 and above who reside in urban areas are more likely to suffer from diabetes than their rural counterparts (1.4 times). Persons who have less than 5 year of schooling are more likely to suffer from diabetes than their counter parts. Level of diabetes prevalence cuts across religion group, which may have implication for the differences in life style and food habits. Adult

[3] International Diabetes Federation (IDF). 2011. *Diabetes Atlas, 5th edition*. Brussels, Belgium*:* IDF

men and women in Muslim religion are very less likely to suffer from diabetes than the other religion categories. Though cast differentials in prevalence of diabetes is not profound but men and women who are non SC/ST and non OBC are more likely to suffer from diabetes than others, which may be primarily due to changes in their life styles and dietary practices.

Tables 8.11 and 8.13 present variation in the prevalence of diabetes among adult men and women age 18 and above across different districts of Haryana. Among districts, men in Sirsa, Bhiwani, Mahendragarh and Palwal have the highest prevalence of diabetes (9% each) followed by Sonipat, Fatehabad, Hisar, Rohtak (8%) and Panipat, Jhajjar and Faridabad, (6% each). On the other hand, men in Karnal (3%) have relatively lower prevalence of diabetes among different districts of Haryana. The pattern remains by and large the same even if we analyze the prevalence of diabetes among adult women in different districts of Haryana. The prevalence is the highest among adult women in Fatehabad, Sirsa and Palwal (8%) and lowest in Kaithal, Rewari and Mewat (4% each).

12.7 Prevalence of Hypertension

Blood pressure rises and falls throughout the day. When blood pressure stays elevated over time. It is called high blood pressure. The medical term for high blood pressure is hypertension. Raised or high blood pressure acts as one of the contributing and intermediate risk factors for developing coronary heart disease, stroke, and kidney disease. The measurements taken for blood pressure in DLHS-4 were not intended to provide a medical diagnosis of the disease but rather to provide a cross-sectional assessment of the prevalence of high blood pressure in the population at the time of the survey. Although the results of the blood pressure measurements are regarded only as a statistical description of the survey population, they provide insight into the size and characteristics of the population at risk for hypertension. The DLHS-4 is used Ross Max AW150 blood pressure monitor model; the automatic devise included separate cuffs for measuring blood pressure in respondents with small, medium and large arm circumferences. Interviewers were adequately trained to use this device according to the recommended protocol. Two health investigators were included in each team for data collection.

Two measurements of both systolic and diastolic blood pressure were taken during the survey at approximately ten minutes interval and the average measurement was used to report respondent's blood pressure values. Tables 8.14 and 8.16 present the information on blood pressure values for men and women age 18 years and above by some selected background characteristics. In the table the blood pressure level has been defined into six categories depending upon various combinations of systolic blood pressure (SBP) and diastolic blood pressure (DBP). The value of SBP greater than 140 mmHg or DBP greater than 90 mmHg is defined as hypertensive with elevated blood pressure, which may have implications for need for medication on a priority basis (AHA, 2003)[4]. In DLHS-4 we have taken SBP 130-139 or DBP 85-89 as the pre-hypertension.

It is evident from Tables 8.14 and 8.16 that 15 percent of men and 11 percent of women age 18 years and above in Haryana are in the stage of pre-hypertension, while 28 percent men and

[4] American Heart Association. http://www.heart.org/HEARTORG/.

21 percent of women age 18 years and above are in the stage of hypertension and require medical attention on a priority basis. A relatively larger proportion of men as well as women age 18 years and above living in urban areas are found to be hypertensive (30% and 23%) than those living in rural areas (27% and 20% respectively). The pattern is by and large the same even in case of the pre-hypertension though the magnitude of difference reduces considerably. The pattern in prevalence of pre-hypertension and hypertension across different districts of Haryana are not uniform. Among men age 18 years and above, prevalence of pre-hypertension ranges from a minimum in Hisar (10%) to the maximum in Rohtak (20%).

In case of hypertension, the prevalence ranges from a minimum in Hisar (19%) to the maximum in Mahendragarh (39%) and Faridabad (36%). In case of women age 18 years and above, the prevalence of pre-hypertension is the lowest in Hisar (6%) among the 21 districts of Haryana. On the other hand, Kurukshetra (16%), Rewari (15%) and Rohtak (14%) are some of the leading districts having higher prevalence of pre-hypertension among women age 18 years and above. District wise variation in the proportion of women age 18 years and above suffering with hypertension (SBP>= 140 or DBP>=90) also portrays the same pattern with the lowest proportion of women in Panipat and Hisar (16% each) the highest proportion in Faridabad (28%), Mahendragarh (27%) and Ambala and Jind (26% each).

12.8 Use of Iodized Salt

Salt used in the household is the most common vehicle for iodine fortification to prevent the public health concerns of iodine deficiency disorders. The compound used for fortification of salt is potassium iodide (KIO3).According to the World Health Organization, a country's salt iodization program is considered to be on a good track to eliminate iodine deficiency when 90 percent of households use iodized salt. The DLHS-4 made an effort to assess household iodized salt consumption by testing iodine contents in the salt being used by the household. Table 8.18 shows the proportion of households using iodized salt according to some selected background characteristics.

Over all, salt was tested for iodine contain in 85 percent of household in Haryana and 56 percent households were found to use salt with adequate iodine contain. Another 24 percent households were found to use salt with iodine but the proportion of iodine contain was not adequate. There is no significant difference in use of iodine salt by place of residence (Table 8.18). Analyzing the variation in proportion of household using iodized salt by districts; it is evident from Table 8.19 that it ranges from a maximum in Jind (79%) to the minimum in Panipat (48%).

13. HEALTH FACILITIES

The basic objective of the population linked facility survey conducted in DLHS-4 is to collect data on health personnel, availability of drugs/medicines, equipments, basic RCH care amenities, communication means and infrastructure at the level of, PHC and CHC, in order to assess the adequacy of RCH services in rural areas.

In DLHS-4 facility survey, at the district level, all Community Health Centres (CHCs), Sub-Divisional Hospital (SDH) and the District Hospitals (DH) were covered. All Sub-Health

Centres and Primary Health Centres (PHCs) which were expected to serve the sampled population of selected PSU were also covered.

In Haryana, the average sampled rural population served per Sub-Health Centre, PHC and CHC are 8,239, 46,503 and 1,56,701 respectively (Table 9.1). In total, villages were surveyed in DLHS-4 and the RCH services of these sampled villages were catered by 673 Sub-Health Centres, 246 PHCs and 106 CHCs.

Out of the total 673 Sub-Health Centers, 388 SHCs are functioning in government buildings, 24.2 percent SHC are have regular electricity (Table 9.2). Forty percent of these Sub-Health Centres are having labour rooms and out of this 82.5 percent are currently in use. Toilet facility is available in 92.3 percent of the sampled Sub-Health Centre located in government buildings. More than 92 percent of these Sub-Health Centres running in government buildings have provision for water supply.

Citizen's charters are displayed in 85.1 percent of the sampled Sub-Health Centres (Table 9.3). The proportion of sampled Sub-Health Centre facilitated by Village Health Nutrition & Sanitation Committee (VHNSC) and those that received untied funds is 89.2 percent and 88.5 percent respectively.

In Haryana, more than 90 percent of the sampled Sub-Health Centers are having Auxiliary-Nurse-Mid-Wife (ANM) and additional ANM are available in 83.5 percent of the SHCs. Less than 20 percent of the sampled SHCs are having Male Health Worker (MHW) in the districts of Kurukshetra, Palwal and Ambala. As compared to other districts in Rohatak district 100 percent of the SHCs are having Male health Worker at the time of the survey (Table 9.4).

In Haryana, out of 246 sampled PHCs, 81 percent of the PHCs have Medical Officers. The state as a whole, the availability of other human resources like Lady Medical Officers, AYUSH Doctors, and Pharmacists in position turned out to be about 34.2 percent, 19.1 percent and 81.7 percent of the sampled PHCs respectively.

More than 60 percent of the sampled PHC in all the districts of Haryna are having Medical Officer except Jind (41%). Hundred percent of the sampled PHCs of Kurukshetra, Sirsa, Rewari, Faridabad and Mewat districts are having Medical Officers during the survey (Table 9.5).

Out of 246 sampled PHCs around 48 percent of the PHCs are having residential quarters for Medical Officer. Seventy nine percent of the sampled PHCs are functioning on a 24 hour basis. About 76 percent of the sampled PHCs catering to the sampled villages have at least four beds. Two-fifths of the PHCs have regular power supply and 31 percent have functional vehicles (Table 9.6).

Newborn care services are available in 91 percent of the sampled PHCs, 66 percent provide referral services for delivery case, and 74 percent have conducted at least 10 deliveries (Table 9.7).

Ninety six percent of sampled PHCs have received 'Untied Fund' and almost all the sampled PHCs (99%) have utilized the funds (Table 9.8). Rogi Kalyan Samiti (RKS) has been constituted in 94 percent and the Citizen's Charter displayed in 90 percent of sampled PHCs.

In Haryna total 106 CHCs have surveyed. Out of this, 14 CHCs are having an Obstetric Gynecologist in position, 7 CHCs are having Pediatrician, 9 CHCs are having Anesthetist and 12 CHCs are having Public Health Manager (Table 9.9).

Out of the total surveyed CHCs, only 8 CHCs have blood storage facility and 76 CHCs have designated as First Referral Units (FRUs). Out of total 106 CHCs around 49 and 97 CHCs are having functional Operation Theaters (OTs) and newborn care services respectively (Table 9.10).

In Haryana out of the total surveyed CHCs, 95 CHCs have utilized untied funds, 100 CHCs have constituted RKS and 99 CHCs are monitoring regularly and displayed citizen's charter in 98 CHCs (Table 9.11).

Total 21 Sub-Divisional Hospitals (SDHs) have surveyed in Haryana out of this only 6 SDHs are having pediatricians and only 2 SDHs are having radiographers in position. Only one Sub-Divisional Hospital namely Faridabad district is having 2D echo facility. Ultra sound facilities are available in only 3 Sub-Divisional Hospitals, three phase connection is available in nineteen SDHs, critical care area is available in 9 Sub-Divisional Hospitals and suggestions and complaint boxes are available in all Sub-Divisional Hospitals.

In Haryana total 21 District Hospitals have surveyed out of this 20 District Hospitals having pediatricians in position and eight of the District Hospitals are having radiographers. Eight District Hospitals are having 2D echo facility and 19 District Hospitals are having ultra sound facilities. Three phase connection is available in all districts, critical care area is available in 16 District Hospitals and suggestions and complaint boxes are available in all the District Hospitals.

BACKGROUND CHARACTERISTICS

TABLE 1.1 BASIC DEMOGRAPHIC INDICATORS

Basic demographic indicators of H and its districts, Census 2011, India.

State/Districts	Population (in thousands)	Percentage urban	Percentage decadal growth rate[1]	Sex ratio[2]	Percentage literate 7+ Male	Female	Total
Panchkula	561.3	54.9	+19.83	873	87.0	76.0	81.9
Ambala	1,128.4	44.4	+11.23	885	87.3	75.5	81.7
Yamunanagar	1,214.2	38.9	+16.57	878	83.8	71.4	78.0
Kuruksherta	964.7	28.9	+16.86	888	83.0	68.8	76.3
kaithal	1,074.3	22.0	+13.55	881	78.0	59.2	69.2
Karnal	1,505.3	30.3	+18.14	887	81.8	66.8	74.7
Panipat	1,205.4	46.0	+24.60	864	83.7	67.0	75.9
Sonipat	1,450.0	30.5	+13.35	856	87.2	69.8	79.1
Jind	1,334.2	22.8	+12.13	871	80.8	60.8	71.4
Fatehabad	942.0	19.0	+16.85	902	76.1	58.9	67.9
Sirsa	1,295.2	24.7	+15.99	898	76.4	60.4	68.8
Hisar	1,743.9	31.7	+13.45	872	82.2	62.3	72.9
Bhiwani	1,634.4	19.8	+14.70	886	85.6	63.5	75.2
Rohtak	1,061.2	42.0	+12.88	867	87.7	71.7	80.2
Jhajjar	958.4	25.4	+8.90	862	89.3	70.7	80.6
Mahendragarh	922.1	14.4	+13.48	895	89.7	64.6	77.7
Rewari	900.3	25.8	+17.64	898	91.4	69.6	81.0
Gurgaon	1,514.4	68.8	+73.14	854	90.5	78.0	84.7
Faridabad	1,089.3	11.4	+38.65	907	69.9	36.6	54.1
Mewat	1,809.7	79.4	+32.54	873	88.6	73.8	81.7
Palwal	1,042.7	22.6	+25.76	881	82.7	54.2	69.3
**Haryana**	25,351.5	34.8	+19.90	879	84.1	65.9	75.6

Source: Primary Census Abstract, Series 20, Census of India, 2011.

[1] 2001-2011. [2] Female per 1,000 males.

TABLE 1.2 NUMBER OF HOUSEHOLDS, EVER-MARRIED WOMEN

Number of households and ever-married women interviewed by district, Haryana, 20012-13.

State/Districts	No of PSU Rural	No of PSU Urban	Number of households interviewed Total	Rural	Urban	HH response rate	Number of ever-married women interviewed Total	Rural	Urban	EW response rate
Panchkula	25	24	1,320	684	636	94.3	986	561	425	97.8
Ambala	33	27	1,621	904	717	96.5	1,303	769	534	97.2
Yamunanagar	34	26	1,607	902	705	95.7	1,271	761	510	90.1
Kuruksherta	44	26	1,893	1,206	687	96.6	1,659	1,107	552	94.7
kaithal	44	26	1,899	1,201	698	96.9	1,558	974	584	94.0
Karnal	44	26	1,904	1,199	705	97.1	1,800	1,169	631	92.5
Panipat	32	28	1,618	858	760	96.3	1,372	756	616	85.4
Sonipat	44	26	1,792	1,137	655	91.4	1,552	957	595	86.0
Jind	44	26	1,900	1,201	699	96.9	1,484	907	577	89.2
Fatehabad	25	25	1,266	655	611	90.4	1,027	530	497	84.7
Sirsa	44	26	1,873	1,193	680	95.6	1,717	1,151	566	89.4
Hisar	44	26	1,893	1,206	687	96.6	1,652	1,123	529	87.6
Bhiwani	25	25	1,345	687	658	96.1	1,230	641	589	88.1
Rohtak	34	26	1,582	904	678	94.2	1,365	820	545	89.4
Jhajjar	44	26	1,873	1,189	684	95.6	1,521	945	576	89.7
Mahendragarh	25	25	1,361	687	674	97.2	1,267	644	623	89.6
Rewari	25	25	1,346	673	673	96.1	1,118	576	542	97.0
Gurgaon	26	34	1,638	708	930	97.5	1,321	636	685	97.1
Faridabad	25	25	1,361	677	684	97.2	1,188	640	548	96.5
Mewat	25	25	1,351	677	674	96.5	1,156	592	564	95.1
Palwal	25	25	1,329	668	661	94.9	1,229	636	593	92.5
**Haryana**	711	548	33,772	19,216	14,556	95.7	28,776	16,895	1,1881	91.3

Note: Table based on unweighted cases.

TABLE 1.3 DISTANCE FROM THE NEAREST EDUCATIONAL FACILITY

Percent distribution of sampled villages by distance from the nearest educational facility, Haryana, 2012-13.

Educational facility	Within village	Distance from the village < 5 km	5-9 km	10+ km	Total percent (100%)
Primary school	93.1	5.8	0.8	0.3	100.0
Middle school	83.6	12.3	3.1	1.0	100.0
Secondary school	67.8	21.5	9.0	1.7	100.0
Higher secondary school	50.4	27.7	14.7	7.2	100.0
College	9.7	11.4	28.0	50.8	100.0
Madarsa	10.6	12.3	21.1	55.9	100.0

Note: Distance from the village is calculated for only those villages which do not have the facility within the village. Table is based on unweighted cases.

TABLE 1.4(a) DISTANCE FROM THE NEAREST HEALTH FACILITY

Percent distribution of sampled villages by distance from the nearest health facility, Haryana, 2012-13.

Health facility	Within village	Distance from the village[1] Within 3km	Within 5km	Within 10km	More than 10km
Sub-Health Centre	59.9	80.4	87.9	94.2	5.8
Primary Health Centre	22.7	41.0	60.2	87.3	12.7
Community Health Centre	6.4	17.1	28.4	56.2	43.8
District/Govt. Hospital	4.4	10.6	17.2	35.2	64.8
Government Dispensary	19.5	33.5	43.1	66.1	33.9
Private Clinic	47.3	59.0	66.1	84.6	15.4
Private Hospital	8.5	23.6	35.6	68.1	31.9
AYUSH Health Facility[2]	7.2	23.4	31.9	57.2	42.8

[1] Including facilities within village.
[2] AYUSH-Ayurveda, Yoga and Naturopathy, Unani, Siddha and Homeopathy.

TABLE 1.4(b) PROGRAMMES BENEFICIARIES

Percentage villages having any beneficiaries from selected programs , Haryana, 2012-13

**Programmes**	Percentage of villages	Number of villages
Janani Suraksha Yojana (JSY)	94.1	666
Janani Shishu Suraksha Karyakram (JSSK)	85.6	606
Integrated Child Development Scheme (ICDS)	79.8	565
Total number of villages		708

TABLE 1.5 REASONS FOR DROPPING OUT OF SCHOOL

Percentage of household population aged 6 to 17 years who dropped out of school by main reasons, Haryana, 2012-13.

	Total			Rural			Urban		
Reason	Male	Female	Total	Male	Female	Total	Male	Female	Total
School too far	4.6	8.1	6.4	4.6	9.2	7.1	4.7	5.5	5.0
Further education not necessary	2.5	5.1	3.8	2.8	6.3	4.7	1.8	2.3	2.0
Required for work in household activities/ farm family/business	7.1	10.4	8.8	6.5	10.6	8.7	8.2	10.0	9.1
Required for outside work	5.6	2.9	4.2	4.8	1.6	3.1	7.1	6.2	6.7
Not interested in studies	33.2	24.7	28.8	33.5	24.9	28.9	32.6	24.1	28.7
Cost too much	14.2	16.7	15.5	12.1	15.4	13.8	18.0	20.3	19.0
Repeated failures	1.9	1.6	1.7	2.7	1.4	2.0	0.3	2.0	1.1
Got married	0.4	4.2	2.3	0.0	4.7	2.6	1.2	2.7	1.9
Others	30.6	26.3	28.4	33.0	26.0	29.2	26.1	26.9	26.5
Total percent	100.0	100.0	100.0	100.0	100.0	100.0	100.0	100.0	100.0
Number of persons**	800	817	1,617	474	547	1,021	326	270	596

** Unweighted cases.

TABLE 1.6a HOUSING CHARACTERISTICS AND HOUSEHOLD ASSETS

Percent of households by housing characteristics and household assets and goods by residence, Haryana, 2012-13.

		Residence	
Housing characteristics	Total	Rural	Urban
**Electricity**			
Having electricity	97.7	97.2	98.7
**Source of drinking water**			
Improved source[1]	99.1	98.9	99.4
**Sanitation facility**			
Improved sanitation[2]	83.7	77.3	94.5
**Fuel used for cooking**			
Liquefied Petroleum Gas (LPG)	45.9	25.1	81.2
Electricity	0.2	0.2	0.3
Kerosene	0.4	0.3	0.5
Wood	23.4	31.4	10.0
Others	0.2	0.3	0.0
**Type of house**			
Kachha	6.7	8.9	3.1
Semi - pucca	23.8	29.7	13.8
Pucca	69.4	61.4	82.8
**Number of rooms**			
1	17.2	16.5	18.3
2	27.8	30.3	23.5
3+	55.1	53.2	58.3
**Household assets**			
Radio/transistor	3.3	3.3	3.3
Television	85.0	81.9	90.1
Computer/ laptops without internet	4.6	2.4	8.3
Computer/ laptops with internet	6.5	2.4	13.6
Telephone only	6.1	3.2	10.9
Mobile only	89.4	87.6	92.4
Washing Machine	40.9	31.6	56.5
Refrigerator	57.3	49.2	70.9
Sewing machine	61.5	60.9	62.4
Watch/ clock	86.9	85.8	88.9
Bicycle	41.7	44.4	37.0
Motor cycle/ scooter	40.5	35.1	49.6
Car / Jeep/van	10.4	5.4	18.9
Tractor	5.0	7.5	0.8
Water pump/tube well	4.9	5.9	3.2
Cart driven by animal	4.2	6.5	0.4
Cart driven by Machine	1.5	2.2	0.4
Other cart	1.0	1.0	0.9
Cooler/AC	46.2	38.3	59.6
**Number of households****	33,772	19,216	14,556

[1] Includes piped into dwelling piped to yard/plot, public tap/stand pipe/hand pump,/tube well/ bore well/well covered/spring tanker, cart with small tank and bottled water. [2] includes flush to sewer/septic/twin pit, pit with slab, pit ventilated improved, other. ** Unweighted cases.

TABLE 1.6b HOUSING CHARACTERISTICS BY DISTRICT

Percentage of households with selected characteristics by districts, Haryana, 2012-13.

District	With electricity	Improved source of drinking water[1]	Improved access to Sanitation[2]	Using Liquefied Petroleum Gas	Living in *pucca* house	Having BPL card
Panchkula	99.2	99.9	89.5	77.3	85.1	10.5
Ambala	99.6	100.0	84.9	62.0	69.6	19.8
Yamunanagar	98.9	99.9	73.9	49.7	72.6	17.7
Kuruksherta	99.8	100.0	91.6	59.1	60.9	22.8
kaithal	91.8	99.8	75.7	38.0	50.2	26.6
Karnal	99.7	99.8	83.4	53.0	57.6	23.5
Panipat	99.0	99.2	84.2	57.7	60.0	17.8
Sonipat	98.7	98.4	82.2	46.3	78.7	15.0
Jind	87.9	96.8	81.0	32.6	43.9	22.2
Fatehabad	97.6	99.9	90.9	45.4	69.6	25.5
Sirsa	97.9	99.4	90.2	39.6	78.6	25.7
Hisar	98.5	99.3	88.7	33.4	83.8	29.3
Bhiwani	99.2	98.6	87.7	37.7	89.4	29.1
Rohtak	98.7	96.7	86.5	46.7	82.9	16.9
Jhajjar	99.4	97.4	84.0	44.4	83.8	17.8
Mahendragarh	96.8	99.0	77.4	37.3	88.5	27.2
Rewari	98.6	99.6	90.6	63.9	73.0	19.1
Gurgaon	99.4	99.8	93.4	73.3	88.7	7.3
Faridabad	99.9	100.0	93.6	61.9	81.7	8.2
Mewat	99.0	99.5	70.7	38.7	40.6	17.9
Palwal	97.8	99.2	79.9	47.3	52.3	16.5
**DLHS-4**	97.7	99.1	83.7	45.9	69.4	20.5
**DLHS-3**	92.4	96.0	56.3	26.3	58.0	18.6

[1] Includes piped into dwelling piped to yard/plot, public tap/stand pipe/hand pump,/tube well/ bore well/well covered/spring tanker, cart with small tank and bottled water. [2] Household having access to toilet facility = improved source of sanitation + flush not to sewer/septic/pit/twin pit + pit without slab + dry toilet.

TABLE 1.7 HOUSEHOLD CHARACTERISTICS
Percentage of the households by selected characteristics of the household head, household size and residence, Haryana, 2012-13.

Characteristics	Total	Residence	
		Rural	Urban
**Sex**			
Male	88.5	88.5	88.5
Female	11.5	11.5	11.5
**Age**			
< 30	7.1	6.8	7.6
30-44	32.5	32.5	32.6
45-59	33.5	32.6	35.0
60+	26.9	28.1	24.8
**Median age**	48	48	48
**Religion**			
Hindu	91.9	90.8	93.6
Muslim	4.4	5.4	2.8
Sikh	3.4	3.6	3.0
Christian	0.1	0.0	0.3
Jain	0.1	0.0	0.3
Others	0.1	0.0	0.1
**Castes/Tribes**			
Scheduled Caste	29.8	33.8	23.0
Scheduled Tribes	5.5	6.3	4.3
Other Backward Classes	28.3	29.5	26.2
Others	36.4	30.4	46.5
**Number of usual members**			
1	3.5	2.4	5.5
2	7.7	6.6	9.6
3	11.7	10.6	13.5
4	23.1	22.1	24.8
5	21.4	22.3	19.9
6	14.3	15.6	12.1
7	7.7	8.5	6.4
8	4.1	4.6	3.3
9+	6.4	7.2	5.0
**Total percent**	100.0	100.0	100.0
**Mean household size**	4.9	5.1	4.6
**Number of households****	33,772	19,216	14,556

Note: Total figure may not add to 100 percent due to ‘do not know’ or ‘missing cases.
** Unweighted cases.

TABLE 1.8 HOUSEHOLD POPULATION BY AGE AND SEX

Percent distribution of the household population by age, residence and sex, Haryana, 2012-13.

	Total			Rural			Urban		
Age group	Total	Male	Female	Total	Male	Female	Total	Male	Female
<1	1.6	1.7	1.6	1.7	1.8	1.7	1.4	1.4	1.4
1-4	6.4	6.7	6.2	6.7	7.0	6.3	6.0	6.2	5.8
5-9	9.2	9.5	8.7	9.4	9.9	8.9	8.6	8.9	8.4
10-14	9.6	10.0	9.1	10.0	10.4	9.5	8.8	9.1	8.4
15-19	10.4	10.9	9.8	10.7	11.2	10.3	9.7	10.3	8.9
20-24	10.5	10.4	10.6	10.6	10.4	10.8	10.4	10.4	10.2
25-29	8.9	8.8	8.9	8.6	8.6	8.6	9.4	9.3	9.5
30-34	7.4	7.3	7.4	7.1	7.0	7.1	8.0	7.9	8.0
35-39	6.8	6.6	7.0	6.5	6.4	6.7	7.2	7.0	7.5
40-44	5.8	5.8	5.9	5.5	5.5	5.5	6.4	6.3	6.6
45-49	4.7	5.0	4.4	4.5	4.8	4.1	5.2	5.5	4.9
50-54	4.8	4.1	5.6	4.5	3.8	5.4	5.3	4.6	6.0
55-59	3.8	3.5	4.2	3.6	3.3	4.0	4.2	4.0	4.4
60-64	3.9	3.6	4.1	3.9	3.7	4.2	3.8	3.6	4.0
65-69	2.5	2.4	2.6	2.5	2.4	2.7	2.4	2.4	2.4
70-74	1.8	1.8	1.8	1.8	1.8	1.8	1.6	1.6	1.7
75-79	0.9	0.8	0.9	0.9	0.8	1.0	0.8	0.8	0.9
80+	1.2	1.1	1.3	1.4	1.3	1.4	0.9	0.7	1.0
Total percent	100.0	100.0	100.0	100.0	100.0	100.0	100.0	100.0	100.0
Number of Persons**	1,66,334	87,983	78,245	99,288	52,373	46,853	67,046	35,610	31,392
Sex ratio at birth[1]	123.7	na	na	120.3	na	na	130.0	na	na
Sex ratio 0-4[1]	122.6	na	na	123.0	na	na	122.0	na	na

Note: Table is based on the *de facto* population, i.e. persons who stayed in the household the night before the interview (including both usual residents and visitors). [1] Females per 1000 males. na = Not applicable. ** Unweighted cases.

TABLE 1.9 MARITAL STATUS OF THE HOUSEHOLD POPULATION

Percent distribution of the household population (age10 years and above) by marital status, age and sex, Haryana, 2012-13.

Age group	Marital status: Never married	Married, *gauna* not performed	Currently Married	Widowed/ divorced/ separated	Total percent	Number of persons**
**Total**						
10-14	99.1	0.1	0.7	0.0	100.0	15,759
15-19	93.5	0.4	5.9	0.0	100.0	17,083
20-24	56.8	0.3	42.5	0.4	100.0	17,339
25-29	20.7	0.2	77.7	1.3	100.0	14,810
30-44	3.9	0.1	92.2	3.8	100.0	33,338
45-49	1.3	0.1	90.9	7.6	100.0	7,885
50-54	1.1	0.1	88.1	10.6	100.0	7,981
55-59	0.9	0.1	86.4	12.6	100.0	6,400
60+	1.0	0.1	69.9	28.9	100.0	16,793
Total	33.8	0.2	59.7	6.3	100.0	1,37,388
**Male**						
10-14	99.1	0.1	0.8	0.0	100.0	8,711
15-19	97.3	0.3	2.3	0.0	100.0	9,490
20-24	74.1	0.3	25.3	0.2	100.0	9,106
25-29	32.8	0.2	65.9	1.1	100.0	7,780
30-44	6.5	0.2	91.0	2.3	100.0	17,392
45-49	2.0	0.2	94.8	3.0	100.0	4,467
50-54	1.8	0.2	93.4	4.5	100.0	3,591
55-59	1.4	0.1	93.0	5.4	100.0	3,126
60+	1.4	0.1	82.6	15.8	100.0	8,466
Total	39.9	0.2	56.7	3.2	100.0	72,129
**Female**						
10-14	99.0	0.1	0.7	0.0	100.0	7,046
15-19	88.9	0.5	10.5	0.1	100.0	7,592
20-24	37.7	0.3	61.5	0.5	100.0	8,229
25-29	7.3	0.3	90.9	1.5	100.0	7,029
30-44	1.0	0.1	93.5	5.4	100.0	15,933
45-49	0.3	0.1	85.9	13.7	100.0	3,416
50-54	0.5	0.0	83.8	15.6	100.0	4,386
55-59	0.4	0.0	80.0	19.6	100.0	3,272
60+	0.5	0.1	57.1	42.2	100.0	8,320
Total	27.1	0.2	62.9	9.7	100.0	65,223

** Unweighted cases

TABLE 1.10 AGE AT MARRIAGE

Mean age at marriage and percentage of marriages below legally prescribed minimum age at marriage by sex, residence and districts Haryana, 2012-13.

Place of residence/ district	Mean age at marriage		Percentage of marriages below legal age at marriage		Currently married women aged 20-24 who were married before age 18 years
	Boys	Girls	Boys (<21 years)	Girls (<18 years)	
Panchkula	26.0	22.5	8.7	0.0	18.7
Ambala	24.5	21.9	6.1	0.8	15.4
Yamunanagar	24.0	21.5	13.6	2.8	15.7
Kuruksherta	24.5	21.9	10.3	1.3	14.1
kaithal	24.1	21.2	17.5	4.3	29.7
Karnal	23.3	20.9	17.5	4.5	26.0
Panipat	23.5	20.7	19.6	11.2	35.2
Sonipat	24.2	20.8	10.7	3.4	21.7
Jind	24.0	20.9	17.0	5.2	28.8
Fatehabad	23.0	20.0	20.3	10.2	22.9
Sirsa	23.6	20.7	18.6	5.9	25.9
Hisar	24.1	20.1	15.8	11.3	32.3
Bhiwani	23.7	20.8	20.4	6.9	32.5
Rohtak	24.3	20.9	12.0	4.5	30.4
Jhajjar	24.4	20.9	13.3	4.5	29.1
Mahendragarh	24.0	20.7	16.7	5.8	28.2
Rewari	24.1	21.3	13.2	3.2	30.9
Gurgaon	24.0	20.5	16.1	7.8	38.9
Faridabad	23.5	20.4	24.2	11.3	29.4
Mewat	21.7	19.7	37.2	10.6	38.0
Palwal	22.8	20.8	25.0	7.8	32.7
Rural	23.4	20.3	19.7	6.5	29.0
Urban	24.5	21.9	12.5	4.6	25.7
**DLHS-4**	23.8	20.8	17.3	5.9	28.0
**DLHS-3**	22.7	19.7	27.4	15.9	28.0

Reference period: January 1st, 2008 to survey date.

TABLE 1.11 EDUCATIONAL LEVEL OF THE HOUSEHOLD POPULATION<br>Percent distribution of household population aged 7 years and above by literacy levels, years of schooling and background characteristics Haryana, 2012-13.								
Background characteristics	Non-literate	Years of schooling among those who are literate<br>Less than 5	6 – 8	9 – 10	11 or more	Missing	Total Percent	Number of persons**
				Total				
**Age**								
7-9	0.9	95.6	0.9	0.0	0.8	1.7	100.0	8,934
10-14	0.1	65.7	27.3	4.2	1.0	1.7	100.0	15,759
15-19	0.1	9.5	21.7	34.7	31.9	2.1	100.0	17,083
20-29	0.3	13.4	13.2	15.9	57.2	0.0	100.0	32,149
30-39	0.5	16.5	14.9	18.7	49.4	0.0	100.0	23,582
40-49	0.7	15.3	12.2	18.6	53.2	0.0	100.0	17,641
50+	1.2	11.6	8.3	12.4	66.5	0.0	100.0	31,174
**Sex**								
Male	0.4	24.8	15.2	18.8	40.3	0.5	100.0	77,058
Female	0.7	23.1	12.8	12.7	50.1	0.6	100.0	69,226
**Religion**								
Hindu	0.5	23.5	14.2	16.4	45.1	0.3	100.0	1,33,897
Muslim	1.2	33.8	11.5	6.4	43.0	4.0	100.0	6,973
Sikh	0.9	23.4	14.2	17.1	44.2	0.3	100.0	4,891
Christian	0.0	19.1	18.9	18.4	43.6	0.0	100.0	204
Jain	0.0	10.2	10.3	24.2	54.7	0.6	100.0	178
Others	0.0	16.7	14.5	8.6	54.8	5.4	100.0	179
**Castes/Tribes**								
Scheduled Caste	0.6	28.6	15.1	12.9	42.1	0.7	100.0	41,400
Scheduled Tribes	0.6	22.4	16.1	15.4	44.8	0.7	100.0	7,660
Other Backward Classes	0.8	25.3	14.6	15.5	43.2	0.7	100.0	40,469
Others	0.4	19.8	12.6	18.6	48.4	0.3	100.0	53,716
Total	0.6	24.0	14.1	15.9	45.0	0.5	100.0	1,46,322
** Unweighted cases.								

TABLE 1.12 EDUCATIONAL LEVEL OF THE HOUSEHOLD POPULATION<br>Percent distribution of household population age 7 years and above by literacy levels, years of schooling and background characteristics Haryana, 2012-13.								
Background characteristics	Non-literate	Years of schooling among those who are literate<br>Less than 5	6 - 8	9 - 10	11 or more	Missing	Total Percent	Number of persons**
				Rural				
**Age**								
7-9	1.0	95.4	1.0	0.0	1.0	1.6	100.0	5,530
10-14	0.1	66.0	27.2	4.2	1.0	1.5	100.0	9,885
15-19	0.1	9.9	23.3	35.0	29.6	2.1	100.0	10,643
20-29	0.3	15.2	14.7	17.4	52.4	0.0	100.0	18,938
30-39	0.6	19.8	16.3	19.0	44.4	0.0	100.0	13,432
40-49	0.9	17.6	12.4	17.0	52.1	0.0	100.0	9,888
50+	1.3	11.1	7.2	9.1	71.3	0.0	100.0	18,477
**Sex**								
Male	0.5	26.5	16.1	18.8	37.6	0.5	100.0	45,573
Female	0.8	24.5	13.1	11.6	49.4	0.6	100.0	41,220
**Religion**								
Hindu	0.6	25.1	14.9	16.0	43.2	0.3	100.0	78,306
Muslim	1.3	33.9	11.6	6.0	43.0	4.3	100.0	5,175
Sikh	1.1	24.6	15.2	15.6	43.2	0.3	100.0	3,150
Christian	0.0	13.2	26.2	19.6	41.0	0.0	100.0	35
Jain	0.0	10.0	16.2	32.8	37.6	3.3	100.0	26
Others	0.0	17.3	13.5	9.6	55.6	4.0	100.0	101
**Castes/Tribes**								
Scheduled Caste	0.6	29.3	15.2	12.5	41.8	0.7	100.0	27,880
Scheduled Tribes	0.6	22.5	15.9	15.3	45.0	0.7	100.0	5,145
Other Backward Classes	0.9	26.2	14.8	15.1	42.2	0.7	100.0	25,059
Others	0.5	21.8	13.9	18.6	45.0	0.2	100.0	26,709
Total	0.6	25.5	14.7	15.4	43.2	0.5	100.0	86,793
** Unweighted cases.								

TABLE 1.13 EDUCATIONAL LEVEL OF THE HOUSEHOLD POPULATION

Percent distribution of household population aged 7 years and above by literacy levels, years of schooling and background characteristics Haryana, 2012-13.

Background characteristics	Non-literate	Years of schooling among those who are literate: Less than 5	6 - 8	9 - 10	11 or more	Missing	Total Percent	Number of persons**
**Urban**								
**Age**								
7-9	0.9	96.1	0.5	0.1	0.6	1.9	100.0	3,404
10-14	0.1	65.2	27.5	4.3	1.0	1.9	100.0	5,874
15-19	0.1	8.7	18.5	34.2	36.5	2.0	100.0	6,440
20-29	0.3	10.1	10.6	13.2	65.7	0.0	100.0	13,211
30-39	0.3	11.2	12.5	18.3	57.6	0.0	100.0	10,150
40-49	0.6	11.8	11.7	21.2	54.8	0.0	100.0	7,753
50+	0.9	12.4	10.4	18.3	57.9	0.0	100.0	12,697
**Sex***								
Male	0.4	21.7	13.6	18.7	45.1	0.5	100.0	31,504
Female	0.6	20.7	12.2	14.8	51.2	0.6	100.0	28,006
**Religion**								
Hindu	0.4	20.8	13.1	17.0	48.2	0.4	100.0	55,591
Muslim	1.0	33.7	11.1	8.1	43.1	3.0	100.0	1,798
Sikh	0.5	20.7	12.1	20.3	46.3	0.1	100.0	1,741
Christian	0.0	20.8	16.9	18.0	44.3	0.0	100.0	169
Jain	0.0	10.3	9.0	22.2	58.5	0.0	100.0	152
Others	0.0	15.8	16.1	7.0	53.6	7.5	100.0	78
**Castes/Tribes**								
Scheduled Caste	0.7	26.8	14.8	13.8	43.0	1.0	100.0	13,520
Scheduled Tribes	0.6	22.2	16.4	15.8	44.2	0.6	100.0	2,515
Other Backward Classes	0.5	23.4	14.2	16.2	45.1	0.6	100.0	15,410
Others	0.3	17.2	11.1	18.8	52.3	0.3	100.0	28,084
Total	0.4	21.2	13.0	16.9	48.0	0.5	100.0	59,529

* Other category in sex is excluded. ** Unweighted cases.

TABLE 1.14 CURRENTLY ATTENDING SCHOOL

Percentage of household population (aged 6 to 17 years) attending school/college , Haryana, 2012-13.

	Total			Male			Female		
	Rural	Urban	Total	Rural	Urban	Total	Rural	Urban	Total
**Age**									
6-10	98.0	97.8	98.0	98.3	98.3	98.3	97.7	97.3	97.6
11-13	98.0	97.3	97.7	98.5	97.3	98.1	97.3	97.2	97.3
14-17	92.0	92.7	92.3	93.4	92.5	93.1	90.4	93.0	91.2
Total	95.7	95.8	95.7	96.5	95.8	96.3	94.8	95.7	95.1
**Religion**									
Hindu	97.1	96.3	96.8	97.3	96.2	96.9	96.8	96.3	96.6
Muslim	82.5	85.8	83.2	88.1	87.6	88.0	76.4	83.9	77.9
Sikh	95.2	97.6	95.9	94.8	96.7	95.4	95.8	98.9	96.7
Christian	100.0	100.0	100.0	--	100.0	100.0	100.0	100.0	100.0
Jain	91.3	100.0	97.1	84.2	100.0	94.9	100.0	100.0	100.0
Others	88.8	67.3	81.3	100.0	62.5	80.9	83.1	76.2	81.6
Total	95.7	95.8	95.7	96.4	95.9	96.2	94.8	95.8	95.1
**Castes/Tribes**									
Scheduled Caste	94.9	92.7	94.3	95.3	92.3	94.5	94.4	93.1	94.0
Scheduled Tribes	94.6	95.4	94.8	95.8	95.0	95.6	93.1	95.9	93.8
Other Backward Classes	95.0	95.7	95.2	96.0	96.4	96.1	93.8	94.8	94.1
Others	97.6	97.8	97.7	98.3	97.7	98.0	96.8	98.1	97.3
Total	95.7	95.8	95.7	96.4	95.9	96.2	94.8	95.8	95.1

TABLE 1.15 AVAILABILITY OF FACILITY AND HEALTH PERSONNEL BY DISTRICT

Percentage of villages with facility and health personnel by district, Haryana, 2012-13.

	Number of villages having facility*						
District	Primary or middle school	Sub-Health Centre	PHCs	Any government health facility[1]	Anganwadi Centre	VHNSC	Number of villages
Panchkula	95.7	43.5	39.1	52.2	100.0	39.1	23
Ambala	93.9	48.5	33.3	57.6	100.0	36.4	33
Yamunanagar	97.1	47.1	14.7	50.0	94.1	26.5	34
Kuruksherta	100.0	40.9	9.1	43.2	100.0	43.2	44
kaithal	90.9	52.3	27.3	65.9	100.0	20.5	44
Karnal	100.0	56.8	20.5	63.6	97.7	45.5	44
Panipat	100.0	61.3	22.6	61.3	100.0	22.6	31
Sonipat	93.2	61.4	34.1	72.7	100.0	25.0	44
Jind	97.7	45.5	25.0	61.4	100.0	18.2	44
Fatehabad	88.0	76.0	32.0	88.0	96.0	80.0	25
Sirsa	97.7	63.6	15.9	70.5	97.7	84.1	44
Hisar	100.0	84.1	22.7	86.4	100.0	88.6	44
Bhiwani	100.0	76.0	28.0	76.0	100.0	92.0	25
Rohtak	97.1	73.5	26.5	82.4	100.0	14.7	34
Jhajjar	97.7	75.0	25.0	77.3	100.0	50.0	44
Mahendragarh	100.0	72.0	24.0	76.0	96.0	92.0	25
Rewari	96.0	56.0	24.0	60.0	92.0	12.0	25
Gurgaon	96.2	42.3	7.7	46.2	100.0	19.2	26
Faridabad	100.0	44.0	20.0	48.0	100.0	56.0	25
Mewat	100.0	80.0	16.0	84.0	100.0	20.0	25
Palwal	96.0	60.0	12.0	60.0	100.0	32.0	25
**Haryana**	97.0	59.9	22.7	66.1	98.9	43.5	708

Note: Table is based on unweighted cases.
* This information was collected from Sarpanch/Pradhan, PRI member, Gram Sevak, Village Secretary/officer or any other official at village level.
[1] Includes Sub-Health Centre, Primary Health Centre (including Block PHC), Community Health Centre or referral hospital, government hospital, and government dispensary within the village. VHNSC = Village Health Nutrition and Sanitation Committee.

TABLE 1.16 BIRTH REGISTRATION
Proportion of children below aged 5 years who have registered the birth with civil authority and received birth certificate by background characteristics, Haryana, 2012-13.

Background characteristics	Birth Registered	Having birth certificate[1]	Number of children below 5 years**
**Age of the children**			
Below 1 year	70.7	79.2	2,512
1 to 2 years	74.1	85.5	2,834
3-4 years	72.0	85.0	5,123
**Sex of the children**			
Male	71.6	82.6	6,890
Female	72.9	83.3	5,652
**Place of residence**			
Rural	71.1	81.6	7,796
Urban	74.5	84.9	4,746
**Religion**			
Hindu	73.3	83.8	11,342
Muslim	54.0	68.5	825
Sikh	78.2	87.4	356
Christian	76.9	66.7	09
Jain	80.2	87.5	09
Others	0.0	100.0	01
**Castes/Tribes**			
Scheduled Caste	72.5	84.2	4,056
Scheduled Tribes	50.4	72.6	674
Other Backward Classes	69.8	80.3	3,630
Others	77.6	85.1	4,182
Total	72.2	82.9	12,542

[1] Out of those registered. ** Unweighted cases.

Table 1.17 BIRTH REGISTRATION
Proportion of children below aged 5 years whose birth have been registered with civil authority and received birth certificate, Haryana, 2012-13.

	Birth Registered			Received birth certificate[1]			Number of children below 5 years**
District	Rural	Urban	Total	Rural	Urban	Total	
Panchkula	74.7	62.9	69.9	94.8	95.2	95.0	345
Ambala	80.4	76.8	78.8	93.5	94.6	94.0	419
Yamunanagar	72.7	73.2	72.9	94.1	96.8	95.0	601
Kuruksherta	76.2	80.7	77.7	95.8	89.3	93.6	589
kaithal	72.5	76.7	73.9	95.9	91.6	94.4	688
Karnal	79.7	85.3	81.4	96.5	93.5	95.5	758
Panipat	77.3	64.6	72.5	93.4	88.1	91.6	704
Sonipat	47.8	51.5	49.0	85.8	88.7	86.8	772
Jind	67.7	93.6	78.2	91.5	95.7	93.5	612
Fatehabad	89.8	76.9	84.0	94.7	97.4	95.8	481
Sirsa	76.8	79.1	77.5	92.2	90.2	91.6	815
Hisar	81.9	84.0	82.6	92.6	91.9	92.4	800
Bhiwani	78.1	91.3	83.6	94.1	92.9	93.5	567
Rohtak	58.5	77.6	65.0	85.5	86.7	86.0	585
Jhajjar	80.9	70.8	77.7	89.7	85.6	88.5	681
Mahendragarh	75.2	88.6	82.4	94.5	93.0	93.6	575
Rewari	70.0	68.5	69.3	87.3	93.4	90.1	372
Gurgaon	63.1	57.7	60.6	90.8	85.4	88.4	542
Faridabad	60.1	57.1	58.9	85.2	91.3	87.5	450
Mewat	38.0	69.3	52.0	80.9	88.5	85.4	552
Palwal	65.6	69.3	67.4	89.5	93.4	91.4	634
**Haryana**	71.1	74.5	72.2	92.0	91.7	91.9	12,542

[1] Out of those registered. ** Unweighted cases.

CHARACTERISTICS OF WOMEN AND FERTILITY

TABLE 2.1 BACKGROUND CHARACTERISTICS OF EVERMARRIED WOMEN

Percentage of ever married women aged 15-49 years according to selected background characteristics, and place of residence, Haryana, 2012-13.

	Place of residence		
Background characteristics	Total	Rural	Urban
**Age Group**			
15-19	2.5	2.9	1.7
20-24	15.5	17.4	12.2
25-29	20.2	20.2	20.2
30-34	18.1	17.5	19.2
35-39	17.7	17.3	18.5
40-44	14.8	14.1	16.2
45-49	11.2	10.6	12.1
**Consummation of marriage**			
Below 18 years	30.1	32.9	25.1
18 years & above	69.9	67.1	74.9
**Marital Duration**			
Less than 5 years	18.5	18.7	18.0
5-9 years	18.9	19.2	18.3
10-14 years	18.2	18.0	18.5
15 or more years	44.5	44.0	45.3
**Woman's education**			
Non-literate[a]	35.0	41.1	24.4
Less than 5 years	1.7	1.9	1.5
5-9 years	26.6	29.7	21.1
10 or more years	36.7	27.4	53.1
**Husband's education**			
Non-literate[a]	22.0	25.4	16.1
Less than 5 years	1.7	1.9	1.4
5-9 years	25.4	28.0	20.7
10 or more years	51.0	44.7	61.8
**Religion**			
Hindu	92.0	90.9	93.9
Muslim	4.5	5.4	2.8
Christian	0.1	0.0	0.2
Sikh	3.4	3.7	2.8
Jain	0.1	0.0	0.2
**Castes/Tribes**			
Scheduled Caste	28.1	31.2	22.6
Scheduled Tribes	5.4	5.9	4.4
Other Backward Classes	28.4	29.7	26.1
Others	38.2	33.2	46.9
**DLHS-4****	28,776	16,895	11,881
**DLHS-3****	21,484	16,154	5,330

[a] Literate but did not attend school, are also included. ** Unweighted cases.

TABLE 2.2 LEVEL OF EDUCATION OF EVER MARRIED WOMEN

Percent distribution of ever married women aged 15-49 years according to selected background characteristics and years of schooling, Haryana, 2012-13.

Background characteristics	Non-literate	Literate but no schooling	Years of schooling: 0 -5 years	6-8 years	9-10 years	11 or more years	Total	Number of women**
**Age group**								
15-19	25.4	0.0	12.8	18.5	21.1	22.1	100.0	687
20-24	21.8	0.1	15.5	16.1	15.7	30.7	100.0	4,375
25-29	23.1	0.1	15.5	14.2	16.9	30.2	100.0	5,832
30-34	30.5	0.1	15.7	14.8	15.3	23.6	100.0	5,221
35-39	40.1	0.1	16.2	12.5	14.3	16.9	100.0	5,133
40-44	49.9	0.1	13.7	9.9	12.5	13.9	100.0	4,294
45-49	56.1	0.1	13.9	7.6	10.5	11.7	100.0	3,234
**Place of residence**								
Rural	41.1	0.1	17.4	14.1	13.5	13.8	100.0	16,895
Urban	24.4	0.1	11.2	11.2	16.7	36.3	100.0	11,881
**Husband's education**								
Non-literate[a]	84.2	0.0	7.9	3.7	2.4	1.7	100.0	6,176
Less than 5 years	47.9	1.1	34.7	9.1	5.6	1.7	100.0	462
5-9 years	37.3	0.2	30.8	19.0	8.7	4.0	100.0	7,224
10 or more years	12.1	0.0	9.8	14.2	23.3	40.5	100.0	14,914
**Religion**								
Hindu	33.3	0.1	15.3	13.2	15.1	22.9	100.0	26,476
Muslim	73.8	0.6	12.5	7.0	3.5	2.6	100.0	1,259
Christian	25.9	0.0	11.1	18.5	14.8	29.6	100.0	34
Sikh	27.5	0.1	15.3	15.9	18.1	23.2	100.0	947
Jain	6.9	0.0	3.4	10.3	24.1	55.2	100.0	31
Others	--	--	--	--	--	--	--	9
**Castes/Tribes**								
Scheduled Caste	46.7	0.1	18.7	12.8	10.6	11.1	100.0	7,950
Scheduled Tribes	43.9	0.3	15.1	13.2	12.8	14.8	100.0	1,500
Other Backward Classes	36.1	0.1	16.2	14.3	14.2	19.0	100.0	8,124
Others	24.2	0.1	11.8	12.3	18.3	33.4	100.0	11,202
**Haryana**	35.0	0.1	15.2	13.0	14.7	22.0	100.0	28,776

Note: Total includes women with missing information on husband's education, who are not shown separately.
[a] Literate but did not attend school, are also included. -- Percentage not shown for less than 10 cases. ** Unweighted cases.

TABLE 2.3 BIRTH ORDER

Percent distribution of births[##] among ever married women aged 15-49 years according to selected background characteristics and birth order, Haryana, 2012-13.

Background characteristics	Distribution of births	Birth order 1	2	3	4+	2 & above	Total	Number of births**
**Age group**								
15-19	1.7	85.4	12.0	2.1	0.4	14.5	100.0	198
20-24	31.3	62.2	30.1	6.2	1.5	37.8	100.0	3,707
25-29	42.8	38.6	38.9	16.3	6.2	61.4	100.0	5,180
30-34	17.1	19.8	36.0	23.0	21.1	80.0	100.0	2,103
35-39	5.4	13.6	24.9	22.0	39.6	86.5	100.0	668
40-45	1.3	9.5	19.7	23.9	46.9	91.1	100.0	163
45-49	0.4	16.4	11.4	3.1	69.1	82.6	(100.0)	50
**Place of residence**								
Rural	66.9	41.2	33.6	14.8	10.4	58.8	100.0	7,510
Urban	33.1	43.0	35.1	13.6	8.3	57.1	100.0	4,559
**Education**								
Non-literate[a]	26.6	29.0	29.2	19.8	22.0	71.1	100.0	3,156
Less than 5 years	1.9	33.4	31.3	17.7	17.7	66.9	100.0	235
5-9 years	30.4	38.4	36.1	16.7	8.9	61.7	100.0	3,617
10 or more years	41.1	53.0	35.9	9.1	2.0	47.0	100.0	5,061
**Religion**								
Hindu	89.3	42.6	34.6	14.4	8.4	57.5	100.0	10,785
Muslim	7.6	28.2	27.9	17.0	27.0	71.9	100.0	900
Christian	0.1	(50.4)	(41.2)	(8.3)	(0.0)	(49.6)	(100.0)	10
Sikh	3.0	52.3	34.2	8.5	4.9	47.8	100.0	359
Jain	0.1	51.4	--	--	--	--	--	08
**Caste/Tribes**								
Scheduled Caste	33.0	37.3	33.0	17.6	12.1	62.7	100.0	3,920
Scheduled Tribes	5.5	38.5	34.9	16.4	10.2	61.6	100.0	647
Other Backward Classes	29.5	40.5	33.6	14.4	11.5	59.6	100.0	3,554
Others	32.0	48.2	35.4	10.8	5.6	51.8	100.0	3,948
**Haryana**	100.0	41.8	34.1	14.4	9.7	58.3	100.0	12,069

[##] Last live/still birth since 01-01-2008. [a] Literate, but did not attend school are also included ( ) Based on 10 -20 cases.
-- Percentage not shown for less than 10 cases. ** Unweighted cases.

TABLE 2.4 BIRTH ORDER BY DISTRICTS

Percent distribution of births[##] among ever married women aged 15-49 years by birth order and districts, Haryana, 2012-13.

Districts	Distribution of births	Birth order 1	2	3	4+	2 & above	Total	Number of births**
Panchkula	3.0	49.2	36.3	10.4	4.2	50.8	100.0	363
Ambala	3.5	46.3	37.2	11.7	4.8	53.6	100.0	430
Yamunanagar	4.7	47.0	32.5	11.5	9.0	53.0	100.0	569
Kurukshetra	4.5	47.0	35.1	10.9	7.1	53.0	100.0	543
Kaithal	5.9	42.1	32.8	15.9	9.3	57.9	100.0	712
Karnal	6.5	42.8	34.4	14.9	7.9	57.2	100.0	768
Panipath	6.1	37.1	31.6	16.5	14.8	62.9	100.0	733
Sonipath	5.9	44.0	34.7	15.4	5.9	56.0	100.0	700
Jind	4.5	44.5	34.4	12.9	8.3	55.5	100.0	550
Fatehabad	3.6	38.5	36.2	16.2	9.2	61.5	100.0	438
Sirsa	6.6	44.0	34.7	12.3	9.0	56.0	100.0	800
Hisar	6.6	38.3	34.1	15.6	12.0	61.7	100.0	805
Bhiwani	4.6	41.4	34.7	15.4	8.4	58.6	100.0	564
Rohtak	4.6	42.1	36.7	13.7	7.5	57.8	100.0	550
Jhajjar	4.8	43.8	37.3	12.9	6.0	56.2	100.0	587
Mahendragarh	4.5	40.5	36.6	16.6	6.3	59.5	100.0	543
Rewari	3.0	46.0	37.0	10.5	6.5	54.0	100.0	359
Gurgaon	3.6	47.7	34.6	12.9	4.8	52.3	100.0	429
Faridabad	3.0	40.4	34.4	15.9	9.3	59.6	100.0	367
Mewat	5.1	29.7	26.8	16.7	26.8	70.3	100.0	612
Palwal	5.3	34.4	30.3	17.7	17.6	65.6	100.0	647
**Haryana**	100.0	41.8	34.2	14.3	9.7	58.2	100.0	12,069

[##] Last live/still birth since 01-01-2008. ** Unweighted cases.

TABLE 2.5 CHILDREN EVER BORN

Mean children ever born (MCEB) according to selected background characteristics of ever married women aged 15-49 years and 40-49 years, Haryana, 2012-13.

Background characteristics	Mean children ever born to women age 15-49 years				Mean children ever born to women age 40-49 years			
	Total	Males	Females	Number of Women**	Total	Males	Females	Number of Women**
**Age Group**								
15-19	0.36	0.21	0.16	687	na	na	na	na
20-24	1.08	0.60	0.47	4,375	na	na	na	na
25-29	1.86	1.02	0.84	5,832	na	na	na	na
30-34	2.38	1.33	1.05	5,221	na	na	na	na
35-39	2.75	1.53	1.22	5,133	na	na	na	na
40-44	2.98	1.66	1.31	4,294	2.98	1.66	1.31	4,294
45-49	3.12	1.74	1.38	3,234	3.12	1.74	1.38	3,234
**Residence**								
Rural	2.33	1.30	1.04	16,895	3.19	1.77	1.42	4,178
Urban	2.15	1.20	0.95	11,881	2.81	1.59	1.22	3,350
**Education**								
Non-literate[a]	2.86	1.57	1.29	9,834	3.40	1.88	1.52	3,840
Less than 5 years	2.66	1.44	1.23	472	3.34	1.83	1.58	126
5-9 years	2.28	1.26	1.02	7,534	2.92	1.62	1.30	1,580
10 or more years	1.67	0.95	0.72	10,936	2.35	1.37	0.99	1,982
**Religion**								
Hindu	2.23	1.24	0.99	26,476	2.99	1.67	1.32	6,976
Muslim	3.17	1.71	1.47	1,259	4.89	2.60	2.29	242
Christian	1.59	0.97	0.62	34	(2.33)	(1.45)	(0.87)	11
Sikh	1.98	1.13	0.84	947	2.55	1.48	1.07	278
Jain	2.27	1.37	0.90	31	2.53	1.48	1.04	15
Others	--	--	--	09	--	--	--	2
**Caste/Tribes**								
Scheduled Caste	2.50	1.36	1.14	7,950	3.49	1.91	1.58	1,927
Scheduled Tribes	2.26	1.26	1.01	1,500	2.99	1.69	1.30	344
Other Backward Classes	2.33	1.29	1.04	8,124	3.19	1.77	1.42	2,024
Others	2.04	1.16	0.88	11,202	2.66	1.52	1.15	3,233
**Haryana**	2.26	1.26	1.01	28,776	3.04	1.70	1.34	7,528

Note: Total figure may not add to 100 due to 'don't know' and 'missing cases. [a] Literate but did not attend school are also included. na = Not applicable. ( ) based on 10 -20 cases. -- Percentage not shown for less than 10 cases. ** Unweighted cases.

TABLE 2.6 OUTCOMES OF PREGNANCY

Percent distribution of all pregnancies of currently married women aged 15-49 years by outcomes since 01-01-2008 according to background characteristics, Haryana, 2012-13.

Background characteristics	Number of Currently Married Women	Percentage of Currently pregnant women	Pregnancy outcome: Live birth	Still birth	Induced abortion	Spontaneous abortion	Total percent	Number of pregnancies**
**Age group**								
15-19	649	17.5	85.4	2.1	2.1	10.4	100.0	233
20-24	4,300	16.0	90.8	1.7	1.7	5.9	100.0	4,100
25-29	5,721	7.7	92.7	1.5	1.4	4.4	100.0	5,576
30-34	5,049	3.8	92.4	1.1	1.6	4.8	100.0	2,276
35-39	4,867	1.7	94.2	2.2	0.1	3.5	100.0	706
40-44	3,991	1.6	93.6	2.1	2.2	2.1	100.0	182
45-49	2,837	0.8	97.4	0.0	2.6	0.0	100.0	51
**Place of resident**								
Urban	16,093	8.1	92.4	1.6	1.3	4.7	100.0	8,137
Rural	11,321	6.1	91.3	1.4	1.8	5.5	100.0	4,987
**Sex-composition of living children**								
One son only	3,323	6.6	90.3	2.1	2.0	5.5	100.0	2,107
One daughter only	1,734	10.7	89.0	1.8	1.7	7.5	100.0	1,487
One son, one daughter only	5,836	1.8	94.6	1.2	1.1	3.1	100.0	2,997
Two sons only	3,548	2.1	95.4	1.2	1.3	2.1	100.0	1,495
Three sons only	654	0.8	95.9	1.0	1.2	1.9	100.0	166
Three daughters only	229	8.6	92.8	1.2	1.1	4.9	100.0	303
Four and above	3,338	2.9	95.8	1.1	0.4	2.7	100.0	1,270
**Woman's Education**								
Non-literate[a]	9,110	6.3	94.5	1.6	.6	3.4	100.0	3,336
Less than 5 years	447	8.8	91.3	2.4	1.0	5.2	100.0	278
5-9 years	7,254	7.7	90.4	2.0	1.6	6.0	100.0	3,998
10 or more years	10,603	7.7	91.7	1.1	1.9	5.2	100.0	5,512
**Husband's education**								
Non-literate[a]	5,541	6.5	95.2	1.5	.5	2.8	100.0	2,030
Less than 5 years	447	10.5	86.6	4.2	1.5	7.6	100.0	279
5-9 years	6,965	8.4	91.3	1.6	1.6	5.5	100.0	3,760
10 or more years	14,461	7.1	91.7	1.4	1.7	5.2	100.0	7,055
**Religion**								
Hindu	25,199	7.3	91.9	1.5	1.5	5.0	100.0	11,743
Muslim	1,221	9.0	93.3	1.8	.4	4.6	100.0	960
Christian	32	10.4	(69.4)	(0.0)	(0.0)	(30.6)	(100.0)	14
Sikh	904	5.7	91.8	1.3	2.9	4.0	100.0	392
Jain	29	6.5	--	--	--	--	--	8
Others	09	--	na	na	na	na	na	na
**Caste/Tribes**								
Scheduled Caste	7,522	8.1	92.7	1.4	1.2	4.6	100.0	4,230
Scheduled Tribes	1,433	7.9	96.5	0.9	0.9	1.7	100.0	676
Other Backward Classes	7,785	8.4	91.5	1.7	1.3	5.5	100.0	3,889
Others	10,674	5.9	91.1	1.6	2.0	5.3	100.0	4,329
**Haryana**	27,414	7.3	92.0	1.5	1.5	5.0	100.0	13,125

[a] Literate but did not attend school, are also included. ( ) based on 10 -20 cases. na = Not applicable -- Percentage not shown for less than 10 cases. ** Unweighted cases.

TABLE 2.7 OUTCOMES OF PREGNANCY

Percent distribution of all pregnancies of currently married women aged 15-49 years by outcomes since 01-01-2008 according to Districts, Haryana, 2012-13.

Districts	Number of Currently Married Women**	Percentage of Currently pregnant women	Pregnancy outcome: Live birth	Still birth	Induced abortion	Spontaneous abortion	Total percent	Number of pregnancies**
Panchkula	945	2.9	93.1	0.2	1.0	5.7	100.0	389
Ambala	1,247	3.6	96.4	0.4	1.3	1.9	100.0	455
Yamunanagar	1,212	6.3	94.0	1.5	1.1	3.3	100.0	604
Kurukshetra	1,586	5.8	92.6	1.3	0.4	5.7	100.0	584
Kaithal	1,488	4.5	91.2	2.0	1.2	5.6	100.0	769
Karnal	1,694	8.7	90.6	2.1	2.1	5.2	100.0	849
Panipath	1,300	6.0	90.7	1.3	1.1	6.8	100.0	810
Sonipath	1,458	6.1	91.3	2.4	2.4	4.0	100.0	769
Jind	1,416	4.0	90.7	1.9	0.4	7.0	100.0	601
Fatehabad	971	3.3	94.1	2.1	1.3	2.5	100.0	466
Sirsa	1,665	6.1	93.0	1.2	2.5	3.4	100.0	868
Hisar	1,539	5.7	89.8	1.9	2.7	5.7	100.0	893
Bhiwani	1,162	3.9	87.6	1.6	3.5	7.4	100.0	646
Rohtak	1,302	5.9	88.7	2.1	1.6	7.7	100.0	624
Jhajjar	1,467	4.2	90.6	1.8	1.7	5.9	100.0	643
Mahendragarh	1,162	3.9	86.5	1.0	2.4	10.1	100.0	626
Rewari	1,081	2.8	94.7	1.1	0.5	3.7	100.0	378
Gurgaon	1,274	4.5	97.7	1.3	0.2	0.8	100.0	440
Faridabad	1,150	2.9	97.7	0.6	0.8	1.0	100.0	378
Mewat	1,112	4.7	94.4	1.3	0.0	4.3	100.0	649
Palwal	1,183	4.1	95.0	1.3	0.6	3.0	100.0	684
**Haryana**	27,414	100.0	92.0	1.5	1.5	5.0	100.0	13,125

** Unweighted cases.

TABLE 2.8 FERTILITY PREFERENCES

Percent distribution of currently married women aged 15-49 years by desire since January 2008 for additional child, by number of surviving children, Haryana, 2012-13.

Desire for children	Number of surviving children: 0	1	2	3	4+	Total
**Desire for additional/next child**						
Want another soon[1]	28.1	7.0	1.3	0.9	1.0	5.0
Want another later[2]	8.6	5.7	0.6	0.2	0.1	2.2
Want another, undecided when	18.8	5.5	0.8	0.4	0.7	3.4
Undecided	18.2	21.6	8.8	6.0	6.4	11.2
Want no more	12.2	35.4	45.7	37.3	36.3	37.4
Sterilized[3]	2.4	9.6	39.2	52.6	52.6	34.5
Declared in fecund	6.8	4.0	2.1	1.7	1.9	2.8
Inconsistent response	4.9	11.1	1.4	0.8	1.1	3.4
Total percent	100.0	100.0	100.0	100.0	100.0	100.0
Number of women**	2,822	5,057	10,250	5,939	3,338	27,406
**Preferred sex of additional/ next child**						
Boy	4.3	21.2	48.6	61.2	43.9	19.2
Girl	1.6	11.3	6.9	1.4	3.1	6.0
Doesn't matter	47.8	35.6	18.0	12.6	36.8	38.0
Up to God	46.3	31.9	26.5	24.8	16.3	36.9
Total percent	100.0	100.0	100.0	100.0	100.0	100.0
Number of women[4]**	1,729	1,537	470	151	98	3,985

[1] Want next birth within 2 years. [2] Want to delay next birth for 2 or more years. [3] Includes both female and male sterilization. [4] Includes women who want another/next child. ** Unweighted cases.

MATERNAL HEALTH CARE

TABLE 3.1 PLACE OF ANTENATAL CHECK-UP

Percentage of women (aged 15-49)[##] who received any antenatal check-up (ANC) during pregnancy by source and place of antenatal check-ups, according to selected background characteristics, Haryana, 2012-13.

Background characteristics	Any antenatal check-up[1]	Place of antenatal check-up[b]				Number of women[**]
		Government health facility[2]		Private health facility[3]	Others[4]	
		Health facility	ICDS/Mobile unit			
**Age group**						
15-19	70.0	68.9	26.1	55.9	6.0	171
20-24	72.6	65.6	19.8	50.0	2.9	2,605
25-29	72.0	62.4	20.9	52.3	3.2	3,500
30-34	69.0	61.5	18.2	54.6	2.9	1,532
35+	60.9	67.4	21.6	43.8	3.6	650
**No. of living children**						
0	81.0	85.8	35.1	33.0	12.5	23
1	77.2	58.6	17.7	57.3	2.7	2,915
2	73.0	63.3	19.6	51.5	3.2	3,195
3	63.3	70.3	22.7	43.5	3.6	1,391
4+	54.2	76.0	29.4	40.5	4.0	934
**Residence**						
Rural	67.2	69.0	26.4	47.5	3.7	5,139
Urban	77.7	55.1	10.1	58.0	2.3	3,319
**Education**						
Non literate[a]	50.2	75.8	27.3	37.7	4.6	2,058
Less than 5 years	65.8	75.8	27.3	40.3	3.6	155
9-10 years	71.7	71.6	24.9	46.8	3.4	2,398
10 or more years	81.9	54.8	14.9	59.2	2.5	3,847
**Religion**						
Hindu	72.7	64.1	19.8	51.3	3.2	7,611
Muslim	42.5	69.1	34.9	46.5	1.4	561
Christian	--	--	--	--	--	7
Sikh	76.0	48.3	15.7	62.6	3.2	268
Jain	--	--	--	--	--	6
Others	--	--	--	--	--	5
**Caste/Tribes**						
Scheduled Caste	65.7	73.3	24.3	41.6	3.9	2,598
Scheduled Tribes	59.9	64.8	13.8	39.6	2.2	445
Other Backward Classes	70.0	65.8	23.4	51.2	3.3	2,486
Others	78.0	54.5	15.4	60.8	2.6	2,929
**DLHS-4**	70.8	63.7	20.2	51.5	3.1	8,458
**DLHS-3**	87.2	45.7	3.8	45.8	NA	6,784

Total figure may not add to 100 percent due to multiple responses, 'do not know' and 'missing cases.
[##] Women who had their last live/still birth since 01-01-2008. [a] Literate but did not attend school are also included. [b] Among those who had received any ANC those who had received any ANC. [1] Antenatal check-up done outside home or at home. [2] Includes sub-health centre, primary health centre, community health centre or rural hospital, urban health centre/ urban health post/ urban family welfare centre, government hospital or dispensary. [3] Includes private hospital/clinic. [4] Includes own home , parents home, other home and other. -- Percentage not shown for less than 10 cases NA: Not available. ** Unweighted cases.

TABLE 3.2 ANTENATAL CARE BY DISTRICT

Percentage of women (aged 15-49)[##] who received any antenatal check-up (ANC) during pregnancy by source and place of antenatal check-ups by districts, Haryana, 2012-13.

District	Any ANC Check up	Place of antenatal check-up[a]				Number of Women**
		Government health facility[1]		Private health facility[2]	Others[3]	
		Health Facility	ICDS/Mobile Unit			
Panchkula	88.0	80.6	18.0	26.1	0.0	271
Ambala	87.2	76.5	28.2	35.9	0.6	335
Yamunanagar	70.3	58.7	22.9	46.3	1.6	406
Kurukshetra	79.3	52.1	28.3	70.8	4.9	427
Kaithal	85.8	79.0	19.3	30.8	0.9	493
Karnal	82.1	51.5	16.6	63.6	5.3	535
Panipath	59.9	28.5	9.3	74.5	8.4	479
Sonipath	45.6	49.2	8.1	53.6	8.9	485
Jind	79.1	63.4	13.5	48.4	0.6	395
Fatehabad	60.7	60.1	19.4	50.8	14.9	305
Sirsa	64.5	48.7	7.6	53.8	3.3	544
Hisar	72.3	68.1	14.6	51.2	1.9	523
Bhiwani	79.9	73.6	23.9	49.5	0.6	386
Rohtak	76.4	70.9	21.4	42.0	5.0	411
Jhajjar	78.4	71.7	18.3	38.6	3.1	416
Mahendragarh	70.1	76.7	13.3	52.9	0.5	353
Rewari	76.7	70.8	39.7	71.4	0.9	273
Gurgaon	76.2	61.3	15.7	54.1	1.4	331
Faridabad	72.9	59.9	19.8	54.5	0.0	264
Mewat	45.6	73.8	35.4	65.0	2.5	397
Palwal	58.3	52.9	27.3	70.8	2.1	429
**DLHS-4**	70.8	63.7	20.2	51.5	3.1	8,458
**DLHS-3**	87.2	45.7	3.8	45.8	NA	6,784

[##] Women who had their last live/still birth since 01-01-2008. [a] Total figure may not add to 100 percent due to 'do not know' and 'missing cases. [1] Includes sub-health centre, primary health centre, community health centre or rural hospital, urban health centre/ urban health post/ urban family welfare centre, government hospital or dispensary. [2] Includes Private hospital/clinic, [3] Includes own home, parents home, other home and others. NA: Not available. ** Unweighted cases.

TABLE 3.3 COMPONENTS OF ANTENATAL CHECK-UP

Percentage of women (aged 15-49)[##] who received specific components of antenatal check-up according to selected background characteristics, Haryana, 2012-13.

Background characteristics	Weight measured	Height measured	Blood pressure checked	Blood tested (Hb)	Urine tested	Abdomen examined	Sonography /ultrasound	Number of Women**
**Age group**								
15-19	59.2	24.1	44.6	50.2	56.3	31.7	57.2	171
20-24	63.5	26.2	41.4	51.5	52.2	35.1	59.5	2,605
25-29	62.2	26.3	42.0	50.5	53.8	35.6	57.6	3,500
30-34	59.8	24.6	39.9	48.1	50.6	33.4	53.3	1,532
35+	49.9	23.3	36.5	41.3	42.6	29.8	45.9	650
**No. of living children**								
0	67.9	36.3	47.2	57.9	57.9	31.0	63.2	23
1	68.7	29.1	46.3	55.7	57.6	37.6	63.8	2,915
2	63.5	26.1	42.4	51.3	53.9	36.6	59.0	3,195
3	52.8	23.0	35.8	43.8	45.4	30.8	49.3	1,391
4+	42.9	17.2	28.3	34.5	37.2	23.4	36.4	934
**Residence**								
Rural	57.1	23.7	35.4	45.6	47.1	30.4	52.8	5,139
Urban	69.1	29.4	51.9	57.5	61.1	42.3	63.7	3,319
**Education**								
Non- literate[a]	38.9	16.5	25.5	29.9	30.9	21.6	33.5	2,010
Less than five years	50.5	20.9	34.5	41.5	48.1	31.4	47.6	2,553
5-9 years	61.5	24.2	39.0	50.1	53.7	33.1	58.6	1,281
10 or more years	73.9	32.0	51.4	60.9	62.6	42.7	68.3	2,566
**Religion**								
Hindu	63.0	26.6	42.4	51.5	53.6	36.0	58.5	7,611
Muslim	33.8	13.9	22.8	25.2	28.3	15.9	28.9	561
Christian	--	--	--	--	--	--	--	7
Sikh	68.0	23.4	40.7	51.9	54.7	31.7	59.0	268
Jain	--	--	--	--	--	--	--	6
Others	--	--	--	--	--	--	--	5
**Caste/Tribes**								
Scheduled Caste	54.8	24.3	36.8	44.6	46.8	30.7	50.3	2,598
Scheduled Tribes	50.9	20.1	31.3	34.6	39.4	29.7	40.9	445
Other Backward Classes	61.1	25.7	40.5	49.5	51.9	35.0	56.9	2,486
Others	69.0	27.89	47.2	57.1	58.6	38.4	64.5	2,929
**DLHS-4**	61.2	25.7	41.1	49.7	51.9	34.5	56.5	8,458
**DLHS-3**	47.6	9.0	42.7	50.2	47.1	58.0	42.1	6,784

Note: Percentage may not add to 100.0 due to multiple responses.
[##] Women who had their last live/still birth since 01-01-2008. [a] Literate but did not attend school, are also included. -- Percentage not shown for less than 10 cases. ** Unweighted cases.

TABLE 3.4 WOMEN RECEIVED ADVICE DURING ANTENATAL CARE

Percentage of women (aged 15-49)[##] who received advice on different components, according to selected background characteristics, Haryana, 2012-13.

Background characteristics	Nutrition for mother and child	Cleanliness at the time of delivery	Institutional delivery	Keep baby warm	Breast feeding	Advice for family planning		Number of Women**
						Spacing	Limiting	
**Age group**								
15-19	38.7	41.1	27.0	50.3	61.4	34.3	25.0	171
20-24	38.0	43.8	25.3	46.7	55.2	37.4	27.9	2,605
25-29	37.0	42.9	24.6	45.0	53.3	37.0	28.5	3,500
30-34	37.8	45.3	27.0	49.4	58.0	39.7	31.4	1,532
35+	33.0	37.2	25.0	39.9	49.0	31.7	27.5	650
**No. of living children**								
0	29.8	43.8	22.3	48.2	62.2	51.1	38.7	23
1	38.5	44.2	25.5	47.9	56.1	38.3	28.2	2,915
2	38.1	43.8	25.9	45.4	53.5	38.0	29.4	3,195
3	34.9	42.2	25.7	46.1	56.8	36.7	31.0	1,391
4+	32.0	37.9	21.8	40.8	49.5	29.2	23.0	934
**Residence**								
Rural	37.1	41.5	25.9	44.5	54.7	36.7	28.3	5,139
Urban	37.5	46.1	24.4	48.7	54.5	38.0	29.3	3,319
**Education**								
Non-literate[a]	30.2	37.6	23.5	38.8	50.1	31.7	23.0	2,010
Less than 5 years	31.9	50.6	20.8	47.1	58.8	32.5	25.8	2,553
5-9 years	36.4	41.1	25.3	45.5	55.0	35.2	27.9	1,281
10 or more year	40.3	46.1	26.1	48.9	55.8	40.3	31.1	2,566
**Religion**								
Hindu	37.2	43.5	25.4	46.5	54.9	37.6	28.8	7,611
Muslim	32.8	39.5	22.4	37.2	46.2	31.0	24.9	561
Christian	--	--	--	--	--	--	--	7
Sikh	40.8	38.9	25.7	43.7	56.3	33.0	26.6	268
Jain	--	--	--	--	--	--	--	6
Others	--	--	--	--	--	--	--	5
**Caste/Tribes**								
Scheduled Caste	34.8	42.1	24.0	47.3	56.6	36.6	27.3	2,598
Scheduled Tribes	29.9	44.8	26.7	57.3	56.5	32.0	22.7	445
Other Backward Classe	37.5	44.8	26.8	44.5	52.6	38.2	30.4	2,486
Others	39.8	42.6	25.1	45.0	54.5	37.6	29.1	2,929
**DLHS-4**	37.2	43.2	25.3	46.1	54.6	37.2	28.7	8,458
**DLHS-3**	48.8	33.8	40.1	32.1	45.7	27.0	26.8	6,784

[##] Women who had their last live/still birth since 01-01-2008. [a] Literate but did not attend school are also included. -- Percentage not shown for less than 10 cases. ** Unweighted cases

TABLE 3.5 (A) ANTENATAL CARE: ANC VISITS AND TIME OF FIRST ANC

Percent distribution of women aged 15-49[##] by the number of antenatal check-up and the stage of pregnancy at the time of first check-up during pregnancy according to selected background characteristics, Haryana, 2012-13.

Background characteristics	Number of ANC Check up				Stage of pregnancy at the time of the first antenatal check-up			Number of Women**
	No Check up	1	2	3+	First trimester	Second trimester	Third trimester	
**Age group**								
15-19	40.5	3.9	11.3	44.3	49.3	8.0	0.7	171
20-24	38.4	3.0	13.1	45.5	49.3	10.0	1.9	2,605
25-29	38.8	2.6	13.0	45.7	51.0	9.6	1.3	3,500
30-34	40.6	2.9	11.1	45.4	49.0	9.1	1.5	1532
35+	48.3	3.2	12.3	36.2	44.2	6.3	0.6	650
**No. of living children**								
0	28.0	3.7	14.0	54.3	72.0	0.0	0.0	23
1	33.2	2.2	12.4	52.2	54.7	10.0	1.5	2,915
2	38.5	2.7	12.6	46.2	51.0	9.4	1.5	3,195
3	47.1	3.3	12.6	37.0	44.2	7.9	1.4	1,391
4+	53.6	4.5	13.0	28.9	36.2	9.5	1.4	934
**Residence**								
Rural	43.5	3.1	12.6	40.9	45.3	10.0	1.4	5,139
Urban	32.6	2.4	12.6	52.4	57.8	8.1	1.5	3,319
**Education**								
Non-literate[a]	59.8	3.5	10.2	26.5	31.5	7.7	1.2	2,010
Less than 5 years	46.4	4.1	12.5	37.0	43.4	11.0	1.3	2,553
5-9 years	37.9	3.4	13.1	45.6	49.7	10.6	1.8	1,281
10 or more year	29.5	2.0	13.6	54.9	59.9	9.4	1.4	2,566
**Religion**								
Hindu	38.4	2.7	13.0	45.9	50.9	9.5	1.5	7,611
Muslim	61.9	5.3	9.4	23.4	28.2	7.9	1.7	561
Christian	--	--	--	--	--	--	--	7
Sikh	32.6	0.8	8.6	58.0	57.0	9.3	0.0	268
Jain	--	--	--	--	--	--	--	6
Others	--	--	--	--	--	--	--	5
**Castes/Tribes**								
Scheduled Caste	44.7	3.6	13.3	38.4	44.1	9.9	1.6	2,598
Scheduled Tribes	51.0	2.1	10.3	36.6	36.2	11.3	0.8	445
Other Backward Classes	39.0	2.5	13.0	45.4	49.7	9.3	1.7	2,486
Others	33.9	2.5	12.0	51.6	56.7	8.5	1.1	2,929
**DLHS-4**	39.7	2.8	12.6	44.8	49.6	9.4	1.5	8,458
**DLHS-3**	13.3	6.6	28.3	51.8	55.0	29.1	2.8	6,784

Note: Percentage may not add to 100.0 due to multiple responses, do not know or missing cases.
[##] Women who had their last live/still birth since 01-01-2008. [a] Literate but did not attend school are also included. -- Percentage not shown for less than 10 cases. ** Unweighted cases.

TABLE 3.5 (B) ANTENATAL CARE: TT, IFA AND ANC

percent distribution of women (aged 15-49)[##] by the number of tetanus toxoid (TT) injections and iron folic acid (IFA) tablets/syrup received during pregnancy, and the percentage who received full antenatal check-up (ANC) according to selected background characteristics, Haryana, 2012-13.

Background characteristics	Women who received TT			Women who received IFA tablets/syrup equivalent		Full ANC[b]	Number of Women**
	No TT	1	2+	No IFA/ syrup	100+ IFA tablets		
**Age group**							
15-19	33.4	5.6	61.0	0.5	19.1	13.4	171
20-24	32.1	8.6	59.3	2.4	20.8	14.2	2,605
25-29	32.5	8.5	59.0	1.7	22.6	16.2	3,500
30-34	35.5	7.3	57.2	1.7	19.7	14.7	1,532
35+	43.6	6.4	50.0	1.7	15.5	11.6	650
**No. of living children**							
0	31.7	6.9	61.4	3.7	27.0	22.7	23
1	27.7	7.7	64.6	1.3	23.7	18.0	2,915
2	31.1	8.7	60.2	2.2	22.0	15.6	3,195
3	41.2	8.5	50.2	2.4	17.8	11.5	1,391
4+	50.3	6.9	42.8	1.9	12.6	8.2	934
**Residence**							
Rural	37.5	8.1	54.4	2.4	18.6	12.5	5,139
Urban	26.7	8.1	65.2	1.0	25.3	19.6	3,319
**Education**							
Non literate[a]	55.0	7.1	37.9	2.0	10.2	6.1	2,010
Less than 5 years	39.8	11.6	48.5	3.7	12.9	10.5	2,553
5-9 years	31.6	8.2	60.3	2.4	19.2	13.8	1,281
10 or more years	23.1	8.5	68.4	1.4	28.3	20.8	2,566
**Religion**							
Hindu	31.9	8.4	59.7	2.0	21.9	15.6	7,611
Muslim	61.5	4.8	33.7	1.1	8.6	5.4	561
Christian	--	--	--	--	--	--	7
Sikh	28.1	7.6	64.3	0.4	18.8	16.4	268
Jain	--	--	--	--	--	--	6
Others	--	--	--	--	--	--	5
**Caste/Tribes**							
Scheduled Caste	38.2	8.6	53.2	2.3	17.8	12.1	2,598
Scheduled Tribes	46.7	9.5	43.8	2.8	16.4	10.3	445
Other Backward Classes	34.8	7.4	57.8	1.6	21.0	15.5	2,486
Others	26.6	8.1	65.3	1.6	24.4	17.8	2,929
**DLHS-4**	33.7	8.1	58.1	1.9	20.9	14.9	8,458
**DLHS-3**	14.0	6.4	79.4	17.8	17.2	13.2	6,784

Note: Percentage may not add to 100.0 due to multiple responses, do not know or missing cases.
[##] Women who had their last live/still birth since 01-01-2008. [a] Literate but did not attend school are also included. [b] At least three visits for antenatal check-up, at least one TT injection received and 100+ IFA tablets/ syrup consumed. -- Percentage not shown for less than 10 cases.
** Unweighted cases.

TABLE 3.6 ANTENATAL CARE INDICATORS AND PREGNACY COMPLICATIONS

Percentage of women (aged 15-49)[##] who received different types of antenatal care (ANC) by districts, Haryana, 2012-13.

District	Antenatal check-up in the first trimester of pregnancy	Three or more antenatal check-up	At least one tetanus toxoid injection	100+ IFA tablets/ syrup[1]	Full antenatal check-up[2]	Any complications	Number of women**
Panchkula	72.1	75.9	85.7	33.0	25.2	40.7	271
Ambala	67.2	68.2	77.9	28.0	25.4	33.1	335
Yamunanagar	51.3	50.0	67.7	28.3	22.8	43.8	406
Kurukshetra	49.2	42.4	73.4	11.4	6.7	24.3	427
Kaithal	65.6	62.5	81.7	22.4	16.1	39.9	493
Karnal	49.8	44.7	79.1	17.9	9.4	33.8	535
Panipath	38.3	31.1	56.9	12.2	7.8	42.7	479
Sonipath	20.8	15.9	39.9	15.8	6.2	56.0	485
Jind	66.5	57.3	74.2	31.3	26.9	33.3	395
Fatehabad	36.6	32.6	57.2	14.3	9.2	38.9	305
Sirsa	44.0	44.0	61.5	16.0	11.3	53.9	544
Hisar	48.9	49.1	69.1	23.5	19.9	40.9	523
Bhiwani	60.6	61.5	77.1	40.3	34.0	34.2	386
Rohtak	41.9	33.4	68.8	12.2	6.1	55.4	411
Jhajjar	55.1	43.3	70.9	24.3	13.6	46.1	416
Mahendragarh	61.9	57.6	69.1	43.3	36.9	34.7	353
Rewari	55.6	46.2	72.5	17.5	11.2	19.6	273
Gurgaon	53.5	51.2	66.0	26.6	16.4	35.9	331
Faridabad	51.0	51.1	66.6	31.8	23.1	25.9	264
Mewat	33.8	22.5	40.5	2.9	1.6	18.9	397
Palwal	49.5	35.1	55.6	9.4	6.2	15.1	429
**DLHS-4**	49.6	44.8	66.2	20.9	14.9	37.5	8,458
**DLHS-3**	55.0	51.8	85.9	29.0	13.2	56.5	6,784

[##] Women who had their last live/still birth since 01-01-2008. [1]100 or more iron folic acid tablets including syrup. [2] At least three visits for antenatal check-up, at least one TT injection received and 100+ IFA tablets/ syrup consumed. ** Unweighted cases.

TABLE 3.7 PLACE OF DELIVERY AND ASSISTANCE

Percent distribution of women (aged 15-49)[##] according to place of delivery, assistance during home deliveries, and safe deliveries according to background characteristics, Haryana, 2012-13.

Background characteristics	Institutional delivery			Delivery at home	Home delivery assisted by skilled persons[1]	Percentage of SBA deliveries[2]	Number of women**
	Government	Private	Total				
**Age group**							
15-19	46.4	31.1	77.4	22.0	9.9	87.3	171
20-24	46.4	33.2	79.6	20.1	12.8	92.4	2,605
25-29	41.0	36.0	77.0	22.4	14.1	91.1	3,500
30-34	39.0	36.5	75.5	24.2	14.7	90.1	1,532
35+	39.7	29.2	68.9	29.2	18.8	87.7	650
**No. of living children**							
0	42.0	35.2	77.2	11.7	3.8	81.0	23
1	41.7	43.8	85.5	14.1	9.1	94.6	2,915
2	43.5	35.7	79.2	20.4	13.0	92.2	3,195
3	44.6	23.7	68.3	31.0	18.9	87.2	1,391
4+	37.2	18.4	55.6	43.3	26.1	81.7	934
**Residence**							
Rural	45.7	28.7	74.3	25.0	15.1	89.4	5,139
Urban	36.1	45.8	81.9	17.8	12.1	94.0	3,319
**Education**							
Non literate[a]	44.0	16.0	60.0	38.9	22.3	82.2	2,010
Less than 5 years	50.5	22.7	73.2	25.1	13.9	87.0	2,553
5-9 years	46.7	27.6	74.3	25.2	16.4	90.7	1,281
10 or more years	38.3	50.0	88.3	11.5	8.0	96.3	2,566
**Religion**							
Hindu	43.9	35.2	79.1	20.5	12.7	91.8	7,611
Muslim	25.9	18.8	44.7	53.7	34.0	78.7	561
Christian	--	--	--	--	--	--	7
Sikh	33.7	50.6	84.4	14.4	9.9	94.3	268
Jain	--	--	--	--	--	--	6
Others	--	--	--	--	--	--	5
**Caste/Tribes**							
Scheduled Caste	48.0	24.0	71.9	27.6	16.0	87.8	2,598
Scheduled Tribes	45.4	25.5	71.0	26.7	11.2	82.2	445
Other Backward Classes	41.6	33.1	74.7	24.8	16.4	91.1	2,486
Others	37.4	47.2	84.6	15.1	10.7	95.2	2,929
**DLHS-4**	42.4	34.6	77.0	22.5	14.1	91.0	8,458
**DLHS-3**	NA	NA	46.8	52.7	6.4	53.2	6,784

Note: Percentage of women who had institutional and home delivery may not add to 100.0, as some deliveries took place on the way to the institute, working place, other place etc.
[##] Women who had their last live/still birth since 01-01-2008. [a] Literate but did not attend school are also included. [1] Includes Doctor/ANM/Nurse. [2] Skilled Birth Attendant. -- Percentage not shown for less than 10 cases. NA: Not available.
** Unweighted cases.

TABLE 3.8 MODE OF TRANSPORTATION USED FOR DELIVERY AND ARRANGEMENT OF TRANSPORTATION

Percent distribution of women (aged 15-49)[##] who had institutional delivery, according to the transportation used to reach the health facility for delivery and transportation arrangement made according to selected background characteristics, Haryana, 2012-13.

Background characteristics	Mode of transportation used to reach the health facility for delivery: Ambulance	Jeep/car	Motor cycle/ scooter	Others[1]	Govt. financial assistance for delivery care (JSY)[c]: Institutional	Home	Number of women**	Mean Transport cost (Rupees)	Mean Delivery cost (Rupees): Govt.	Private	Number of women**
**Age group**											
15-19	26.4	29.0	14.8	7.2	8.4	0.0	171	1000	2,869	12,975	36
20-24	29.0	30.1	11.4	9.4	8.2	2.9	2,605	868	3,528	10,442	734
25-29	22.9	33.7	11.7	9.1	8.8	2.5	3,500	1200	3,686	10,239	974
30-34	22.1	31.9	11.8	10.1	7.8	3.2	1,532	1030	2,766	9,605	424
35+	24.9	25.9	8.5	9.6	6.8	2.1	650	1192	3,538	10,460	152
**No. of living children**											
0	16.0	49.1	0.0	12.0	6.3	0.0	23	0	13,500	8,900	9
1	26.8	39.1	11.5	8.3	6.6	3.2	2,915	1001	3,383	10,538	948
2	24.3	33.5	12.7	9.0	8.8	3.0	3,195	1027	3,508	10,180	905
3	24.8	22.8	10.1	11.3	11.1	2.3	1,391	1233	3,695	9,711	280
4+	21.4	14.3	9.3	10.9	8.8	1.9	934	1224	2,715	9,250	178
**Residence**											
Rural	28.1	29.7	9.9	6.9	9.5	3.1	5,139	1010	3,397	10,526	1,300
Urban	18.7	35.2	14.3	14.0	6.2	1.4	3,319	1191	3,594	9,914	1,020
**Education**											
Non literate[a]	27.4	15.7	7.7	9.7	10.6	2.0	2,010	1255	2,950	8,609	333
Less than 5	35.0	18.7	7.0	12.9	13.0	2.8	2,553	1000	5,683	12,755	40
5-9 years	26.6	23.8	12.4	11.9	9.9	4.5	1,281	835	3,461	9,549	644
10 or more years	21.9	46.0	13.1	7.3	6.4	1.3	2,566	1109	3,654	10,629	1,303
**Religion**											
Hindu	25.6	32.2	11.8	9.7	8.4	3.1	7,611	1041	3,454	10,350	2,120
Muslim	17.1	12.1	8.3	7.4	6.8	0.3	561	1456	3,683	9,087	87
Christian	--	--	--	--	--	--	7	0	3,000	0	1
Sikh	21.6	52.6	6.9	3.7	6.5	1.7	268	975	3,489	8,855	108
Jain	--	--	--	--	--	--	6	0	0	11,667	3
Others	--	--	--	--	--	--	5	0	0	3,000	1
**Caste/Tribes**											
Scheduled Caste	29.4	21.2	11.0	10.5	13.2	4.4	2,598	1204	3,021	10,892	583
Scheduled Tribes	24.6	29.8	8.3	9.4	7.7	0.6	445	2009	2,412	9,796	111
Other Backward Classes	23.6	29.8	11.7	9.8	7.8	1.7	2,486	976	3,576	9,748	673
Others	21.8	43.0	12.1	4.9	4.9	1.4	2,929	885	4,055	10,263	953
**DLHS-4**	24.9	31.6	11.4	9.3	8.3	2.6	8,458	1058	3,464	10,222	2,320
**DLHS-3**	1.1	47.8	14.6	34.7	4.6	3.5	6,784	299	3,483	7,523	3,233

Note: Total figure may not add to 100 percent due to don't know or missing cases.
[##] Women who had their last live/still birth since 01-01-2008. [a] Literate but did not attend school are also included. [1] Includes bus/train, tempo/auto/tractor, animal drawn cart, foot march. [c] Percentage women who got JSY assistance. -- Percentage not shown for less than 10 cases.
** Unweighted cases.

TABLE 3.9 PLACE OF DELIVERY AND ASSISTANCE CHARACTERISTICS BY DISTRICT

Percent distribution of women (aged 15-49)## according to place of delivery, assistance during home deliveries, and safe deliveries by districts, Haryana, 2012-13.

District	Percentage of women who had institutional delivery	Percentage of women who had delivery at home	Home delivery assisted by skilled persons[1]	Percentage of SBA Delivery[2]	Mean Delivery cost	Number of Women**
Panchkula	93.8	6.2	5.4	99.2	5,190	271
Ambala	91.0	9.0	7.5	98.4	5,667	335
Yamunanagar	75.8	23.7	17.4	93.2	7,199	406
Kurukshetra	83.8	15.6	11.5	95.3	9,743	427
Kaithal	87.5	12.5	8.9	96.4	8,812	493
Karnal	81.6	18.4	12.6	94.3	10,196	535
Panipath	58.9	40.3	24.9	83.8	9,835	479
Sonipath	75.6	22.7	13.7	89.3	7,899	485
Jind	88.0	11.5	8.5	96.4	5,076	395
Fatehabad	78.1	21.6	16.1	94.2	5,497	305
Sirsa	70.0	29.0	19.4	89.4	7,397	544
Hisar	70.4	29.4	11.5	81.9	10,230	523
Bhiwani	79.1	20.9	11.8	91.0	9,277	386
Rohtak	84.6	15.4	10.8	95.4	4,720	411
Jhajjar	90.0	9.7	7.0	97.0	7,775	416
Mahendragarh	73.5	26.2	6.7	80.2	10,309	353
Rewari	83.3	16.0	11.0	94.4	6,932	273
Gurgaon	90.9	9.1	3.2	94.1	3,648	331
Faridabad	75.9	23.8	12.6	88.5	5,614	264
Mewat	51.3	48.5	32.6	83.9	5,631	397
Palwal	56.8	40.3	29.3	86.1	9,254	429
**DLHS-4**	77.0	22.5	14.1	91.0	7,766	8,458
**DLHS-3**	46.8	52.7	6.4	53.2	NA	6,784

Note: Percentage of women who had institutional and home delivery may not add to 100.0, as some deliveries took place on the way to the institute, working place, other place etc.
Women who had their last live/still birth since 01-01-2008. [1] Includes Doctor/ANM/Nurse. [2] Skilled Birth Attendants. ** Unweighted cases.

TABLE 3.10 REASONS FOR NOT GOING TO HEALTH INSTITUTIONS FOR DELIVERY

Percent distribution of women (aged 15-49)[##] according to main reasons for not going to health institution for delivery, according to selected background characteristics, Haryana, 2012-13.

Background characteristics	Reasons[b]										Number of women**
	Cost too much	Poor quality service	Too far/ No transport	No time to go	Not Necessary	Not Customary	Better care at home	Family did not allow	Lack of know ledge	Other	
**Age group**											
15-19	25.1	3.2	0.0	25.5	16.3	4.5	5.0	20.2	0.0	0.0	38
20-24	17.1	5.1	2.4	26.4	17.9	2.2	6.3	15.3	5.6	1.6	519
25-29	16.6	4.0	1.9	28.6	20.1	1.5	8.1	12.8	4.9	1.6	775
30-34	20.9	5.5	0.8	28.2	15.6	1.9	8.2	11.4	6.7	0.8	358
35+	21.8	4.7	1.7	21.2	18.3	1.0	9.3	14.4	7.2	0.6	186
**No. of living children**											
0	--	--	--	--	--	--	--	--	--	--	3
1	18.1	5.9	1.8	28.8	13.7	2.2	7.2	16.0	4.5	1.8	408
2	14.5	5.0	2.2	30.7	19.4	2.0	6.3	14.7	3.9	1.2	636
3	20.3	2.8	1.4	27.5	20.3	0.9	8.1	10.6	6.5	1.6	425
4+	22.3	4.6	1.5	19.4	19.3	1.9	9.9	12.1	8.3	0.7	404
**Residence**											
Rural	15.2	4.8	2.3	29.3	17.8	1.8	8.2	13.0	6.5	1.3	1,282
Urban	26.4	4.3	0.4	21.3	19.8	1.8	6.3	15.1	3.0	1.5	594
**Education**											
Non literate[a]	23.3	5.0	1.4	20.6	18.2	2.2	8.6	12.0	7.5	1.2	799
Less than 5 years	16.1	9.9	4.8	12.8	18.1	2.7	15.2	17.5	0.0	2.8	41
5-9 years	14.6	4.1	1.6	31.8	20.9	1.6	7.5	12.8	3.9	1.2	602
10 or more years	14.0	4.2	2.5	33.9	15.2	1.3	5.5	17.0	4.8	1.6	434
**Religion**											
Hindu	17.3	4.8	1.8	30.2	17.2	1.5	6.8	14.1	4.7	1.4	1,538
Muslim	23.9	2.8	1.4	10.7	23.9	3.3	12.1	9.9	10.9	1.1	298
Christian	--	--	--	--	--	--	--	--	--	--	--
Sikh	10.1	11.2	2.0	32.1	20.3	0.0	5.7	18.6	0.0	0.0	40
Jain	--	--	--	--	--	--	--	--	--	--	--
Others	--	--	--	--	--	--	--	--	--	--	--
**Castes/Tribes**											
Scheduled Caste	19.5	5.3	1.3	25.9	19.9	1.7	6.6	13.8	4.1	1.8	714
Scheduled Tribes	15.7	2.9	0.7	21.4	13.2	2.3	5.0	29.4	9.4	0.0	121
Other Backward Classes	18.4	3.7	2.4	26.3	18.0	2.1	9.9	10.9	6.8	1.5	609
Others	16.5	5.3	1.9	32.0	17.7	1.3	7.2	12.3	5.3	0.7	432
**DLHS-4**	18.2	4.6	1.8	27.1	18.4	1.8	7.7	13.5	5.6	1.3	1,876
**DLHS-3**	22.7	3.5	1.8	14.2	45.7	4.5	25.3	5.2	1.4	1.9	3,593

[##] Women who had their last live/still birth since 01-01-2008. [a] Literate but did not attend school, are also included. [b] Total figure may not add to 100 percent due to ' Multiple responses', 'don't know' or 'missing cases. -- Percentage not shown for less than 10 cases. ** Unweighted cases.

TABLE 3.11 DELIVERY COMPLICATIONS

Percentage of women (aged 15-49)## who had complication during delivery and type of complications during delivery, according to selected background characteristics, Haryana, 2012-13.

Background characteristics	Any delivery complication	Type of delivery complications: Premature labour	Excessive bleeding	Prolonged labour	obstructed labour	Breech presentation	Convulsion/ high BP	Number of Women**
**Age group**								
15-19	37.9	64.1	13.1	19.1	40.7	5.1	0.0	171
20-24	42.1	60.1	11.6	19.2	43.8	5.4	6.1	2,605
25-29	42.9	60.8	10.9	17.5	44.0	6.5	5.5	3,500
30-34	45.3	61.5	9.4	15.2	43.9	7.8	6.0	1,532
35+	42.2	64.1	8.4	16.0	41.3	3.2	4.4	650
**No. of living children**								
0	43.3	73.3	0.0	17.9	26.8	9.8	8.1	23
1	43.3	59.1	11.0	16.7	43.4	6.3	5.6	2,915
2	41.6	60.6	10.6	19.5	45.9	6.5	5.8	3,195
3	43.8	63.3	11.4	16.2	41.6	5.7	5.0	1,391
4+	45.1	64.5	9.1	15.9	41.0	5.4	5.8	934
**Residence**								
Rural	41.6	61.1	10.0	16.9	44.4	5.9	5.6	5,139
Urban	45.5	60.9	11.8	18.7	42.5	6.6	5.7	3,319
**Number of ANC Visits**								
0	37.6	60.2	10.3	14.5	47.0	4.5	4.5	3,308
1	35.4	69.5	22.8	13.8	29.9	3.7	5.6	238
2	39.9	55.6	10.4	17.3	43.1	5.5	4.7	1,064
3+	49.0	62.5	10.4	19.9	42.2	7.5	6.5	3,848
**Delivery**								
Normal	41.7	62.6	10.9	16.6	43.1	5.2	5.3	7,502
Caesarean	54.6	52.3	10.7	25.2	42.2	12.7	8.0	749
By Instrument or Assisted	48.6	45.8	5.0	18.9	70.6	9.7	4.3	203
**Place of Delivery**								
Government facility	41.7	64.1	11.2	19.3	41.4	6.2	6.2	3,544
Private facility	45.7	55.6	10.7	18.9	47.0	7.9	5.8	2,994
Home	41.4	65.1	9.7	11.6	42.0	3.2	4.2	1,876
Other	30.1	13.9	12.6	36.9	60.9	0.0	0.0	28
**DLHS-4**	42.9	61.0	10.7	17.5	43.7	6.1	5.6	8,458
**DLHS-3**	55.8	29.6	4.8	24.5	80.9	6.4	3.2	6,784

Note: Total figure may not add to 100 percent due to 'multiple responses', 'don't know' or 'missing cases.

Women who had their last live/still birth since 01-01-2008. ** Unweighted cases.

TABLE 3.12 POST-DELIVERY COMPLICATIONS

Percentage of women (aged 15-49)[##] who had post delivery complication and type of complications, according to selected background characteristics, Haryana, 2012-13.

Background characteristics	Any post delivery complication	Type of post delivery complication				Number of women**
		High fever	Lower abdominal pain	Foul smelling vaginal discharge	Excessive bleeding	
**Age group**						
15-19	17.6	35.3	66.2	16.2	14.6	171
20-24	15.2	54.0	50.6	14.3	17.1	2,605
25-29	15.3	51.7	51.3	16.9	15.7	3,500
30-34	14.6	50.4	40.9	14.4	14.3	1,532
35+	15.2	59.6	48.8	14.8	12.8	650
**No. of living children**						
0	19.1	34.0	67.2	17.2	0.0	23
1	14.9	51.9	49.9	15.4	15.8	2,915
2	15.1	50.8	52.1	15.0	17.5	3,195
3	15.1	53.3	43.2	16.3	13.5	1,391
4+	16.4	57.8	47.9	15.9	12.7	934
**Residence**						
Rural	14.7	52.5	49.5	12.8	15.3	5,139
Urban	16.2	52.1	49.5	20.1	16.2	3,319
**Delivery**						
Normal	14.4	53.3	48.8	16.2	16.1	7,502
Caesarean	22.3	44.1	56.8	13.2	14.9	749
By Instrument or Assisted	20.4	60.1	38.4	6.3	5.0	203
**Place of Delivery**						
Government facility	15.9	55.9	51.7	16.7	16.5	3,544
Private facility	16.0	47.6	47.5	14.1	15.9	2,994
Home	12.4	53.5	47.8	15.4	13.3	1,876
Others	29.4	47.6	62.0	13.8	13.7	28
**Who Conducted the Last Delivery**						
Doctor	16.1	54.1	54.1	23.9	23.9	78
ANM/Nurse/Midwife/LHV	15.8	38.2	32.4	18.5	2.3	231
Dai	12.2	55.6	50.6	14.5	15.9	1,421
Relatives/Friends	12.8	43.4	59.8	16.0	8.6	169
None	11.1	100.0	29.7	0.0	0.0	30
**DLHS-4**	15.2	52.4	49.5	15.5	15.6	8,458
**DLHS-3**	28.3	56.1	44.6	12.5	15.4	6,784

[##] Women who had their last live/still birth since 01-01-2008. ** Unweighted cases.

TABLE 3.13 ANY CHECK-UP AFTER DELIVERY

Percentage of women (aged 15-49)[##] whether received any check-up after delivery according to background characteristics, Haryana, 2012-13.

Background characteristics	Check up within 48 hours after delivery	Check up within 48 hours after delivery at Home	Check up within 2 weeks after delivery	Check up within 14 to 42 days after delivery	Number of Women**
**Age group**					
15-19	60.2	15.3	61.9	61.9	171
20-24	56.6	24.5	58.1	58.3	2,605
25-29	60.0	28.0	61.8	62.0	3,500
30-34	58.0	30.5	59.8	60.0	1,532
35+	52.5	29.8	54.3	54.5	650
**No. of living children**					
0	55.4	0.0	49.2	49.2	23
1	63.6	29.0	65.0	65.2	2,915
2	58.4	25.0	60.5	60.6	3,195
3	53.3	32.6	54.8	54.9	1,391
4+	46.7	24.2	48.4	48.9	934
**Residence**					
Rural	56.3	26.2	58.0	58.2	5,139
Urban	61.2	30.5	63.0	63.2	3,319
**Education**					
Non literate[a]	43.8	24.5	45.6	45.9	2,010
Less than 5 years	56.9	22.6	58.8	58.8	2,553
5-9 years	58.6	29.5	60.1	60.2	1,281
10 or more years	65.6	30.3	67.4	67.5	2,566
**Religion**					
Hindu	59.2	28.4	61.0	61.2	7,611
Muslim	38.3	22.3	39.5	39.5	561
Christian	--	--	--	--	7
Sikh	65.8	26.8	66.1	66.1	268
Jain	--	--	--	--	6
Others	--	--	--	--	5
**Castes/Tribes**					
Scheduled Caste	52.8	24.0	54.5	54.6	2,598
Scheduled Tribes	56.3	29.0	57.8	58.4	445
Other Backward Classes	59.0	26.9	61.0	61.2	2,486
Others	62.3	33.6	63.7	63.9	2,929
**DLHS-4**	58.0	27.4	59.7	59.9	8,458
**DLHS-3**	48.8	NA	49.4	NA	6,784

[##] Women who had their last live/still birth since 01-01-2008. [a] Literate but did not attend school are also included. NA: Not available.
-- Percentage not shown for less than 10 cases. ** Unweighted cases.

TABLE 3.14 COMPLICATIONS DURING PREGNANCY, DELIVERY AND POST-DELIVERY PERIOD

Percentage of women (aged 15-49)[##] who had extent of pregnancy, delivery and post-delivery complications and sought treatment for the problem according to background characteristics, Haryana, 2012-13.

Background characteristics	Who had complication during pregnancy	Sought treatment for pregnancy complication[1]	Who had delivery complication	Who had post-delivery complication	Sought treatment for post-delivery complication[2]	Number of women**
**Age group**						
15-19	38.2	45.0	37.9	17.6	51.1	171
20-24	37.9	45.3	42.1	15.2	51.7	2,605
25-29	38.3	47.0	42.9	15.3	58.2	3,500
30-34	35.6	45.7	45.3	14.6	50.5	1,532
35+	35.2	36.4	42.2	15.2	40.7	650
**No. of living Children**						
0	43.5	52.2	43.3	19.1	59.7	23
1	38.4	47.2	43.3	14.9	52.0	2,915
2	38.9	46.7	41.6	15.1	57.1	3,195
3	34.5	42.6	43.8	15.1	51.4	1,391
4+	34.0	38.5	45.1	16.4	46.8	934
**Residence**						
Rural	37.5	42.1	41.6	14.7	53.3	5,139
Urban	37.5	51.8	45.5	16.2	53.3	3,319
**Education**						
Non literate[a]	31.7	34.4	38.1	14.5	48.0	2,010
Less than five years	45.7	34.6	55.6	19.0	59.3	2,553
5-9 years	40.4	44.7	47.0	16.2	48.4	1,281
10 or more years	38.5	51.5	42.5	14.8	58.9	2,566
**Religion**						
Hindu	38.2	45.1	42.9	15.0	52.9	7,611
Muslim	27.3	40.8	38.5	15.5	49.1	561
Christian	--	--	--	--	--	7
Sikh	39.2	62.8	51.6	20.4	66.1	268
Jain	--	--	--	--	--	6
Others	--	--	--	--	--	5
**Castes/Tribes**						
Scheduled Caste	37.1	41.2	42.3	15.9	45.5	2,598
Scheduled Tribes	33.8	37.7	30.0	11.6	62.0	445
Other Backward Classes	38.3	48.4	47.0	16.7	59.5	2,486
Others	37.7	47.7	42.0	13.8	53.7	2,929
**DLHS-4**	37.5	45.4	42.9	15.2	53.3	8,458
**DLHS-3**	56.5	60.1	55.8	28.3	71.3	6,784

[##] Women who had their last live/still birth since 01-01-2008. [a] Literate but did not attend school are also included. [1] Women who reported at least one complication of pregnancy. [2] Women who reported at least one post delivery complication. -- Percentage not shown for less than 10 cases. ** Unweighted cases.

TABLE 3.15 COMPLICATIONS DURING PREGNANCY, DELIVERY AND POST-DELIVERY PERIOD

Percentage of women (aged 15-49)## who had extent of pregnancy, delivery and post-delivery complications and sought treatment for the problem according to by districts, Haryana, 2012-13.

Districts	Who had complication during pregnancy	Sought treatment for pregnancy complication[1]	Who had delivery complication	Who had post-delivery complication	Sought treatment for post-delivery complication[2]	Number of women**
Panchkula	40.7	54.4	44.5	18.5	48.9	271
Ambala	33.1	26.7	48.7	9.6	39.2	335
Yamunanagar	43.8	40.4	66.6	14.6	51.9	406
Kurukshetra	24.3	53.8	36.4	11.6	55.2	427
Kaithal	39.9	43.0	45.9	21.5	44.7	493
Karnal	33.8	46.6	45.4	13.8	56.6	535
Panipath	42.7	45.0	62.7	25.8	48.7	479
Sonipath	56.0	27.0	51.7	14.3	45.8	485
Jind	33.3	48.6	38.7	8.5	56.5	395
Fatehabad	38.9	42.5	64.6	23.0	58.0	305
Sirsa	53.9	58.9	51.0	23.1	74.5	544
Hisar	40.9	51.0	34.5	9.5	50.9	523
Bhiwani	34.2	57.9	48.3	13.0	65.4	386
Rohtak	55.4	33.7	36.4	26.5	35.9	411
Jhajjar	46.1	38.1	35.6	19.8	46.3	416
Mahendragarh	34.7	67.5	41.0	6.6	86.2	353
Rewari	19.6	72.9	29.6	5.9	51.1	273
Gurgaon	35.9	39.5	28.0	15.1	51.1	331
Faridabad	25.9	40.7	27.7	7.1	67.8	264
Mewat	18.9	65.9	28.7	15.9	63.1	397
Palwal	15.1	52.6	28.5	8.3	51.9	429
**DLHS-4**	37.5	45.4	42.9	15.2	53.3	8,458
**DLHS-3**	56.5	60.1	55.8	28.3	71.3	6,784

Women who had their last live/still birth since 01-01-2008. [1] Women who reported at least one complication of pregnancy. [2] Women who reported at least one post delivery complication. ** Unweighted cases.

TABLE 3.16 AWARENESS OF THE DANGER SIGNS OF NEW BORN

Percentage of women (aged 15-49)[##] who had awareness of the danger signs of new born, according to selected background characteristics, Haryana, 2012-13.

Background characteristic	Difficulty in breathing	Cold/ hot to touch	Develop yellow staining on palm and soles	Blue tongue & Lips	Abnormal movement	Poor sucking of breast	Baby did not cry	Number of Women**
**Age group**								
15-19	11.8	14.2	4.8	12.7	5.5	16.5	10.9	171
20-24	10.6	16.4	4.6	9.7	4.6	17.2	11.6	2,605
25-29	11.9	19.2	5.3	11.1	6.9	18.5	11.7	3,500
30-34	11.0	19.3	5.0	11.7	7.5	18.4	14.8	1,532
35+	10.1	15.1	3.3	7.2	5.2	17.1	10.5	650
**Children ever born**								
0	6.9	17.4	0.0	12.2	8.1	13.7	24.7	23
1	11.9	18.5	5.2	10.9	7.3	18.1	12.5	2,915
2	11.8	18.9	5.2	10.3	6.2	19.1	12.4	3,195
3	10.7	18.2	4.3	11.4	4.4	17.2	10.9	1,391
4+	7.6	12.7	3.6	8.6	4.7	14.6	11.2	934
**Residence**								
Rural	9.4	16.1	4.0	10.2	4.5	17.3	11.2	5,139
Urban	14.6	21.5	6.6	11.1	9.2	19.1	13.8	3,319
**Education**								
Non literate[a]	6.4	11.2	2.1	5.9	2.4	11.8	7.7	2,010
Less than 5 years	9.0	16.9	3.4	7.8	7.6	20.7	14.4	2,553
9-10 years	9.8	18.1	4.0	10.8	4.5	18.8	12.8	1,281
10 or more years	14.8	21.6	7.0	12.9	9.2	20.7	14.0	2,566
**Religion**								
Hindu	11.6	18.4	5.1	11.0	6.3	18.3	12.1	7,611
Muslim	6.0	10.8	2.8	4.2	3.2	11.6	9.9	561
Christian	--	--	--	--	--	--	--	7
Sikh	9.6	21.1	3.6	10.8	6.8	21.3	15.8	268
Jain	--	--	--	--	--	--	--	6
Others	--	--	--	--	--	--	--	5
**Castes/Tribes**								
Scheduled Caste	9.9	16.5	4.0	9.4	3.6	15.7	9.8	2,598
Scheduled Tribes	3.2	8.8	0.5	2.8	3.1	7.7	4.7	445
Other Backward Classes	11.7	18.1	5.3	11.5	6.9	19.3	14.8	2,486
Others	13.2	20.5	6.0	11.9	8.2	20.4	13.1	2,929
**DLHS-4**	11.2	17.9	4.9	10.5	6.1	17.9	12.1	8,458
**DLHS-3**	48.1	37.9	26.8	41.8	29.2	54.4	52.9	6,784

[##] Women who had their last live/still birth since 01-01-2008. [a] Literate but did not attend school are also included. -- Percentage not shown for less than 10 cases. ** Unweighted cases.

CHILD HEALTH CARE AND IMMUNIZATION

TABLE 4.1 TIMING AND CHILDHOOD CHECK-UPS

Percentage of children aged under 3 years received check up and place of check-up according to selected background characteristics, Haryana, 2012-13.

Background characteristics	Children received Check-up within 24 hours of birth	Number of children**	Place of check-up [4] Government[1]	Private[2]	Home[3]	Others	Total	Number of children[4]**
**Age group**								
15-19	58.4	177	58.9	33.1	5.0	2.9	100.0	106
20-24	56.3	2,340	52.3	40.9	6.3	0.6	100.0	1,331
25-29	57.5	2,361	47.6	46.1	5.8	0.5	100.0	1,363
30-34	52.9	850	47.6	44.5	7.2	0.7	100.0	458
35-39	55.2	258	50.6	38.0	9.6	1.8	100.0	143
40-44	53.9	55	41.1	28.6	23.5	6.8	100.0	29
45-49	(31.8)	11	--	--	--	--	--	4
**Residence**								
Rural	56.1	3,742	56.0	36.3	7.0	0.7	100.0	2,121
Urban	56.5	2,310	38.3	55.6	5.3	0.7	100.0	1,313
**Mother's education**								
Non-literate[a]	46.9	1,459	57.8	25.6	14.6	1.9	100.0	693
Less than 5 years	50.3	103	75.0	20.2	4.8	0.0	100.0	51
5-9 years	55.4	1,738	61.3	31.5	6.5	0.8	100.0	966
10 or more years	62.1	2,752	39.7	56.4	3.6	0.3	100.0	1,724
**Religion**								
Hindu	57.5	5,418	51.0	43.0	5.3	0.6	100.0	3,142
Muslim	42.4	436	40.9	32.8	24.0	2.3	100.0	186
Christian	38.0	6	--	--	--	--	--	2
Sikh	52.3	183	37.4	57.7	5.0	0.0	100.0	96
Jain	--	4	--	--	--	--	--	4
Others	--	5	--	--	--	--	--	4
**Castes/Tribes**								
Scheduled Caste	53.2	1,919	60.5	32.4	6.3	0.8	100.0	1,023
Scheduled Tribes	55.3	316	55.2	31.6	13.2	0.0	100.0	175
Other Backward Classes	56.4	1,778	52.9	39.2	6.9	1.0	100.0	1,012
Others	59.1	2,039	37.4	57.1	5.1	0.4	100.0	1,224
**DLHS-4**	56.2	6,052	49.9	42.9	6.4	0.7	100.0	3,434
**DLHS-3**	49.5	6,278	22.9	54.8	19.2	3.0	100.0	3,000

Note: Table based on youngest living child born *since 01.01.2008*

[a] Literate but did not attend school are also included. [1] Includes government hospital or dispensary, urban health centre/ urban health post/ urban family welfare centre, community health centre or rural hospital, primary health centre, sub-health centre, ICDS and Govt. AYUSH hospital /clinic. [2] Includes non-governmental hospital/ trust hospital or clinic, private hospital/clinic and private AYUSH hospital /clinic. [3] Includes Doctor ASHA and ANM/Nurse. [4] Among those Children who received check-up within 24 hours of birth. () based on 10-20 unweighted cases. -- Percentage not shown for less than 10 cases.** Unweighted cases.

TABLE 4.2 INITIATION OF BREASTFEEDING

Percentage of youngest living child born since 01.01.2008 aged under 3 years whose mother started breastfeeding within one hour of birth, within 24 hours of birth and after 24 hours of birth according to selected background characteristics, Haryana, 2012-13.

Background characteristics	Children received Colostrum/*Khees*[b]	Initiation of breastfeeding			Number of children**
		Within one hour of birth	Within 24 hours of birth[1]	After 24 hours of birth	
**Age group**					
15-19	77.2	49.0	73.3	12.8	177
20-24	84.6	51.0	82.7	8.3	2,340
25-29	84.7	52.5	84.0	8.6	2,361
30-34	83.8	47.3	83.4	7.6	850
35-39	82.3	51.8	81.1	8.8	258
40-44	66.0	42.7	71.8	9.7	55
45-49	(81.8)	(36.4)	(81.8)	(9.1)	11
**Residence**					
Rural	84.0	51.0	83.0	8.4	3,742
Urban	84.0	50.7	82.5	8.7	2,310
**Mother's education**					
Non-literate[a]	80.9	50.9	81.9	10.5	1,459
Less than 5 years	77.7	47.9	80.3	10.5	103
5-9 years	85.1	49.0	82.6	8.1	1,738
10 or more years	85.3	52.4	83.6	7.5	2,752
**Religion**					
Hindu	84.3	51.4	83.2	7.9	5,418
Muslim	78.7	44.6	77.5	14.8	436
Christian	--	--	--	--	6
Sikh	87.3	52.1	84.7	10.2	183
Jain	--	--	--	--	4
Others	--	--	--	--	5
**Castes/Tribes**					
Scheduled Caste	84.6	51.9	84.2	7.9	1,919
Scheduled Tribes	88.4	66.1	88.4	5.3	316
Other Backward Classes	82.6	49.3	81.3	9.5	1,778
Others	84.1	49.0	82.1	8.7	2,039
**DLHS-4**	84.0	50.9	82.8	8.5	6,052
**DLHS-3**	80.8	16.5	55.4	44.6	6,278

[a] Literate but did not attend school are also included. [b] Yellowish thick milk secretion during the first few days after child birth. [1] Includes children whose mother started breastfeeding within one hour of birth. ( ) based on 10-20 unweighted cases. -- Percentage not shown due to less number of cases. ** Unweighted cases.

TABLE 4.3 BREASTFEEDING AND WEANING STATUS

Percentage of children aged under 3 years who had exclusive breastfeeding and weaning status, Haryana, 2012-13.

Age in months	Exclusive breastfeeding	Weaning status[1]				Number of children**
		Other fluids	Semisolid food	Solid food	Solid/semi-solid food	
<2	59.1	0.3	0.6	0.3	0.6	352
2-3	57.0	0.2	0.0	0.0	0.0	380
4-5	46.6	1.0	0.8	0.3	0.8	363
6-8	39.4	4.8	6.8	1.6	7.7	580
9-11	33.4	12.7	14.8	7.2	15.8	619
12-17	33.6	15.3	17.7	11.8	18.8	1,150
18-23	25.7	19.9	21.1	16.0	22.5	953
24-35	19.0	19.7	23.5	20.8	27.1	1,655
6-9	38.2	5.2	7.8	2.2	9.0	792
6-35[2]	27.8	16.1	18.7	13.9	20.6	4,957

Note: Table based on youngest living child born since 01.01.2008.
[1] Based on those children who had breastfeeding with other fluids, semi solid food and solid food. [2] Children aged 6-35 months breastfed for at least 6 months. ** Unweighted cases.

TABLE 4.4 EXCLUSIVE BREASTFEEDING

Percentage of youngest living child born since 01.01.2008 aged 0-5 months who received exclusive breastfeeding according to selected background characteristics, Haryana, 2012-13.

Background characteristics	Exclusive breastfeeding	
	0-5 months	Number of children**
**Age group**		
15-19	62.9	57
20-24	66.0	451
25-29	60.5	286
30-34	67.5	91
35-39	66.5	33
40-44	(50.0)	10
45-49	--	1
**Residence**		
Rural	63.0	607
Urban	66.2	322
**Mother's education**		
Non-literate[a]	60.8	237
Less than 5 years	(50.0)	12
5-9 years	60.5	256
10 or more years	68.5	424
**Religion**		
Hindu	64.6	822
Muslim	62.0	82
Christian	na	0
Sikh	54.9	24
Jain	--	1
Others	na	0
**Castes/Tribes**		
Scheduled Caste	61.9	302
Scheduled Tribes	59.9	55
Other Backward Classes	65.8	262
Others	65.3	310
**DLHS-4**	64.0	929
**DLHS-3**	9.4	862

[a] Literate but did not attend school are also included. ( ) based on 10-20 cases.
-- Percentage not shown for less than 10 cases. na: not applicable. ** Unweighted cases.

TABLE 4.5 BREASTFEEDING BY DISTRICTS

Percentage of children aged under 3 years whose mother started breastfeeding within one hour of birth, within 24 hours of birth, and after 24 hours of birth by districts, Haryana, 2012-13.

Districts	Children received Colostrum/*Khees*[a]	Initiation of breastfeeding: Within one hour of birth	Within 24 hours of birth[1]	After 24 hours of birth	Number of children**
Panchkula	85.5	55.5	89.4	0.8	189
Ambala	92.7	68.5	95.0	0.4	234
Yamunanagar	93.6	47.4	91.7	4.2	303
Kuruksherta	81.5	61.3	83.8	9.3	291
kaithal	77.9	46.4	79.2	6.8	342
Karnal	77.0	46.8	81.1	8.8	390
Panipat	73.9	32.2	66.2	21.6	359
Sonipat	87.9	55.3	83.3	9.0	355
Jind	80.2	46.8	79.7	3.6	278
Fatehabad	90.3	43.6	82.7	10.9	221
Sirsa	82.9	51.4	78.8	13.7	375
Hisar	83.4	41.0	78.1	10.5	389
Bhiwani	82.3	46.6	84.1	4.7	266
Rohtak	87.4	49.5	85.7	5.7	299
Jhajjar	89.0	55.5	87.4	5.0	299
Mahendragarh	81.5	42.4	79.6	6.8	260
Rewari	91.0	69.0	88.3	8.4	175
Gurgaon	88.0	59.3	95.0	1.6	222
Faridabad	95.3	68.2	96.9	2.1	181
Mewat	77.9	41.3	72.8	19.0	307
Palwa	82.0	66.8	83.0	11.1	317
**DLHS-4**	84.0	50.9	82.8	8.5	6,052
**DLHS-3**	80.8	16.5	55.4	44.6	6,278

Note: Table based on youngest living child born since 01.01.2008. [a] Yellowish thick milk secretion during the first few days after child birth. [1] Includes children whose mother started breastfeeding within one hour of birth. ** Unweighted cases.

TABLE 4.6 VACCINATION OF CHILDREN

Percentage of children aged 12-23 months who received specific vaccination according to selected background characteristics Haryana, 2012-13.

Background characteristics	BCG	DPT 1	DPT 2	DPT 3	Polio 0	Polio 1	Polio 2	Polio 3	Measles	Full vaccination[1]	No vaccination	Vaccination card seen	Number of children**
**Residence**													
Rural	83.9	85.3	81.4	71.8	15.9	82.9	79.3	70.0	68.0	50.9	7.3	28.1	1,237
Urban	84.7	87.6	84.8	74.5	14.8	85.7	82.3	73.2	73.1	54.5	5.4	26.3	801
**Sex of child**													
Male	83.8	86.3	82.7	72.2	14.9	84.3	80.7	71.1	69.8	51.9	6.5	28.2	1,142
Female	84.7	85.8	82.5	73.5	16.3	83.4	79.8	71.1	69.7	52.5	6.8	26.5	896
**Birth order**													
1	86.4	88.2	84.5	74.6	15.3	86.5	83.2	72.4	73.7	55.0	5.0	29.1	831
2	83.3	85.8	82.7	73.0	14.2	83.6	79.6	71.1	70.1	53.4	7.2	28.1	720
3	81.9	83.1	78.2	69.2	16.6	80.4	76.5	69.3	63.2	45.6	5.7	26.2	309
4+	81.3	82.2	80.7	69.0	19.7	78.8	76.2	67.9	61.4	44.9	13.3	19.6	178
**Mother's education**													
Non-literate[a]	74.2	78.2	72.4	63.7	17.4	76.7	72.0	64.1	55.5	40.5	13.6	18.4	493
Less than 5 years	73.4	78.5	78.5	71.1	15.6	70.9	67.9	67.8	58.5	45.7	19.2	30.7	37
5-9 years	87.7	88.7	85.9	74.0	16.1	86.4	83.6	73.1	71.0	52.9	4.0	30.5	590
10 or more years	87.9	89.0	86.3	77.1	14.1	86.8	83.3	73.7	77.4	58.4	4.0	30.4	918
**Religion**													
Hindu	85.3	87.1	83.7	73.7	15.7	84.9	81.8	72.2	71.1	53.4	5.1	28.3	1,813
Muslim	65.4	67.9	63.0	54.8	15.9	66.0	59.3	53.2	45.8	30.5	26.6	15.3	152
Christian	--	--	--	--	--	--	--	--	--	--	--	--	4
Sikh	97.3	98.6	96.5	86.2	8.9	96.7	89.6	83.9	88.5	69.4	1.4	34.2	65
Jain	--	--	--	--	--	--	--	--	--	--	--	--	1
Others	--	--	--	--	--	--	--	--	--	--	--	--	3
**Castes/Tribes**													
Scheduled Caste	82.7	85.4	82.0	71.0	15.7	83.4	79.3	67.7	67.6	48.0	8.0	26.1	661
Scheduled Tribes	81.6	83.2	77.9	67.8	21.2	79.5	79.5	70.8	62.4	47.7	6.9	26.2	114
Other Backward Classes	83.6	84.9	80.9	71.0	14.9	82.7	79.4	71.9	66.2	50.3	7.8	28.0	603
Others	86.7	88.4	85.7	77.2	15.2	86.4	82.4	73.9	76.7	59.0	4.0	28.7	660
**DLHS-4**	84.2	86.1	82.6	72.7	15.5	83.9	80.3	71.1	69.8	52.1	6.6	27.5	2,038
**DLHS-3**	86.5	84.6	79.4	69.0	28.1	97.0	82.2	67.8	69.0	59.6	1.9	38.8	2,125

Note: Table based on youngest living child born since 01.01.2008. [a] Literate but did not attend school are also included. [1] BCG, three injections of DPT, three doses of Polio (excluding Polio "0") and measles. -- Percentage not shown for less than 10 cases. ** Unweighted cases.

TABLE 4.7 STATUS OF CHILDHOOD VACCINATION BY DISTRICTS

Percentage of children aged 12-23 months received specific vaccination and Vitamin-A supplementation by districts, Haryana, 2012-13.

		Vaccination Status							
District	Vaccination card seen	BCG	DPT 3	Polio 3	Measles	Full[1]	None	Percentage received at least one dose of Vitamin-A[2]	Number of children**
Panchkula	24.7	90.8	75.1	85.6	82.8	65.9	3.1	78.0	64
Ambala	20.2	91.9	80.5	82.5	72.7	64.8	6.8	61.5	65
Yamunanagar	33.7	93.0	74.1	81.2	65.7	50.0	4.0	66.8	122
Kuruksherta	29.7	84.7	74.6	69.1	74.7	52.2	4.4	77.1	96
kaithal	31.5	93.9	87.9	85.8	87.9	73.8	0.8	76.9	119
Karnal	31.5	89.8	75.2	77.8	79.5	58.2	2.5	80.5	133
Panipat	20.6	82.1	62.9	71.2	70.6	39.9	10.1	52.8	114
Sonipat	19.1	55.0	55.6	48.8	40.2	32.6	9.9	41.9	119
Jind	30.5	92.0	83.2	76.1	83.0	65.0	2.2	73.8	98
Fatehabad	31.8	85.6	74.0	79.5	72.6	60.1	5.3	66.4	79
Sirsa	38.0	86.7	77.2	71.1	67.8	52.2	9.0	54.2	129
Hisar	33.5	94.6	84.2	80.2	76.4	62.3	2.2	59.8	122
Bhiwani	32.6	91.2	93.6	83.8	72.4	62.6	3.6	64.7	81
Rohtak	15.6	79.6	69.5	63.9	62.9	43.7	6.1	59.2	108
Jhajjar	29.7	91.7	72.1	65.8	85.1	52.8	2.7	64.0	96
Mahendragarh	38.7	94.8	89.4	80.5	83.7	70.7	2.0	69.3	100
Rewari	30.7	75.5	62.2	64.3	58.5	43.0	7.4	66.4	49
Gurgaon	19.5	81.7	56.5	68.8	62.3	40.3	14.1	60.9	79
Faridabad	23.0	86.9	66.6	57.5	57.6	38.7	5.9	49.9	53
Mewat	21.4	52.6	50.2	45.2	41.2	27.4	29.7	49.3	99
Palwa	13.2	77.1	61.8	58.3	67.9	42.6	11.0	63.5	113
**DLHS-4**	27.5	84.2	72.7	71.1	69.8	52.1	6.6	63.3[#]	2,038
**DLHS-3**	38.8	86.5	69.0	67.8	69.0	59.6	1.9	49.4	2,125

Note. Table based on youngest living child born since 01.01.2008.. [1] BCG, three injections of DPT, three doses of Polio (excluding Polio 0) and measles. [2] Children aged 12-35 months. [#] Percentage in fact sheet children aged 9-35 months** Unweighted cases.

TABLE 4.8 PLACE OF CHILDHOOD VACCINATION

Percentage of children aged 3 years received vaccination by place of vaccination, according to selected background characteristics, Haryana, 2012-13.

	Place of vaccination						
	Government health sector						
Background characteristics	Anganwadi Centre	Sub-Health Centre	Primary Health Centre	Other government health facility	Private health sector[1]	Others	Number of children**
**Residence**							
Rural	57.8	14.3	5.2	24.4	8.8	0.1	2,980
Urban	26.9	3.4	2.3	50.3	26.4	0.3	1,889
**Sex of the child**							
Male	46.8	10.3	4.5	33.7	14.7	0.1	2,730
Female	47.9	11.0	3.9	32.6	15.0	0.3	2,139
**Birth order**							
1	45.5	9.8	3.8	33.5	19.0	0.1	1,935
2	44.8	11.4	4.4	34.8	15.0	0.1	1,776
3	50.8	11.6	4.1	32.5	7.6	0.4	710
4+	59.2	9.5	5.9	27.1	7.3	0.7	447
**Mother's education**							
Non-literate[a]	55.1	10.4	5.3	29.9	8.2	0.6	1,056
Less than 5 years	55.4	13.7	5.3	30.2	5.2	0.0	75
5-9 years	53.9	12.1	4.9	31.4	7.4	0.1	1,432
10 or more years	39.0	9.6	3.3	36.1	23.0	0.0	2,306
**Religion**							
Hindu	46.2	10.9	4.5	33.5	15.1	0.2	4,415
Muslim	72.1	2.8	1.6	26.5	7.5	0.0	281
Christian	--	--	--	--	--	--	3
Sikh	36.8	16.3	1.8	37.4	17.2	0.0	161
Jain	--	--	--	--	--	--	4
Others	--	--	--	--	--	--	5
**Castes/Tribes**							
Scheduled Caste	52.2	13.0	4.3	31.0	10.5	0.3	1,554
Scheduled Tribes	53.0	8.4	3.8	31.5	7.9	0.3	243
Other Backward Classes	51.6	10.1	4.1	33.8	11.8	0.2	1,392
Others	37.9	9.1	4.4	35.2	22.6	0.1	1,680
**DLHS-4**	47.3	10.6	4.2	33.2	14.8	0.2	4,869
**DLHS-3**	NA	12.9	8.4	85.2	9.2	2.6	6,043

Note: Table based on youngest living child born since 01.01.2008.
[a] Literate but did not attend school are also included. [1] Includes non-governmental hospital/trust hospital or clinic, private hospital and private doctor/clinic. -- Percentage not shown for less than 10 cases. NA: Not available. ** Unweighted cases

TABLE 4.9 VITAMIN-A AND HEPATITIS-B SUPPLEMENTATION FOR CHILDREN

Percentage of children aged 12-35 months received at least one dose of Vitamin-A, 3-5 doses of Vitamin-A and Hepatitis-B injection, according to selected background characteristics, Haryana, 2012-13.

Background characteristics	Children who received at least one dose of Vitamin-A[##]	Children who received 3-5 doses of Vitamin-A	Children who received Hepatitis-B injection	Number of children**
**Age of the child**				
12-23 months	59.1	41.6	55.4	2,612
24-35 months	65.0	45.7	62.0	1,637
**Residence**				
Rural	59.9	41.4	55.8	2,612
Urban	64.4	46.7	62.2	1,637
**Sex of the child**				
Male	63.0	44.5	59.1	2,393
Female	59.3	41.6	56.5	1,856
**Birth order**				
1	64.9	44.0	61.6	1,644
2	62.5	43.0	58.4	1,549
3	57.5	43.1	53.5	644
4+	49.0	40.6	48.9	412
**Mother's education**				
Non-literate[a]	49.9	45.4	47.6	1,034
Less than 5 years	47.6	33.2	36.2	71
5-9 years	59.7	40.4	55.3	1,213
10 or more years	69.4	44.3	66.3	1,931
**Religion**				
Hindu	62.6	43.1	59.3	3,808
Muslim	43.4	48.8	40.7	300
Christian	--	--	--	4
Sikh	66.1	38.4	57.6	130
Jain	--	--	--	2
Others	--	--	--	5
**Castes/Tribes**				
Scheduled Caste	58.2	42.8	55.1	1,356
Scheduled Tribes	58.7	54.6	53.2	230
Other Backward Classes	62.6	41.4	56.4	1,249
Others	63.9	43.6	63.1	1,414
**DLHS-4**	61.4	43.3	58.0	4,249
**DLHS-3**	49.4	9.3	32.9	4,205

Note: Table based on youngest living child born since 01.01.2008.
[a] Literate but not attend school are also included. [##] Children aged 9-35 months. -- Percentage not shown for less than 10 cases. ** Unweighted cases

TABLE 4.10 AWARENESS REGARDING DIARRHOEA MANAGEMENT

Percentage of women who are aware of diarrhoea management according to selected background characteristics, Haryana, 2012-13.

Background characteristics	Knowledge of diarrhoea management	Type of practices followed if child gets diarrhoea[1]						Number of women**
		Give ORS	Salt and sugar solution	Continue normal food	Continue breast-feeding	Give plenty of fluids	Others	
**Age group**								
15-19	64.2	54.0	40.9	16.7	12.3	22.2	3.0	649
20-24	76.2	65.7	48.9	20.9	13.2	28.3	4.9	4,300
25-29	82.1	70.2	53.9	24.2	15.2	31.3	5.5	5,721
30-34	84.2	71.0	55.2	26.7	15.9	33.3	5.6	5,049
35-39	83.8	68.3	55.1	26.9	15.4	33.0	6.2	4,867
40-44	83.9	66.4	56.7	26.0	15.0	32.0	6.5	3,991
45-49	82.3	65.4	56.8	26.2	15.1	32.8	6.2	2,837
**Residence**								
Rural	79.6	64.4	50.3	22.4	14.6	29.7	6.5	16,093
Urban	85.4	73.9	60.2	29.2	15.6	34.8	4.3	11,321
**Mother's education**								
Non-literate[a]	75.2	57.8	47.0	20.7	12.7	28.7	5.5	9,110
Less than 5 years	77.0	53.0	45.4	19.6	11.6	19.8	10.6	447
5-9 years	80.9	64.9	51.7	23.8	14.8	28.7	6.9	7,254
10 or more years	88.3	79.8	62.2	29.7	17.3	36.7	4.8	10,603
**Religion**								
Hindu	82.5	68.7	54.7	25.3	15.0	32.1	5.9	25,199
Muslim	65.6	49.6	43.0	19.1	12.5	24.2	2.6	1,221
Christian	79.1	74.8	64.3	19.9	10.8	19.5	3.7	32
Sikh	80.8	69.7	46.9	19.8	16.9	27.8	3.9	904
Jain	92.9	82.0	79.1	39.5	6.9	38.1	0.0	29
Others	72.8	41.9	52.3	52.5	16.1	29.6	15.9	29
**Castes/Tribes**								
Scheduled Caste	78.2	63.8	49.5	23.4	14.0	30.1	5.2	7,522
Scheduled Tribes	79.8	62.9	54.0	19.8	11.3	31.2	1.8	1,433
Other Backward Classes	79.7	65.5	52.8	25.2	14.9	30.3	6.3	7,785
Others	86.0	73.3	58.0	26.5	16.2	33.6	6.2	10,674
**DLHS-4**	81.7	67.9	53.9	24.9	14.9	31.6	5.7	27,414
**DLHS-3**	79.0	43.3	53.9	18.1	3.0	24.6	38.9	21,484

Note: Table based on women with last two surviving children born since 01.01.2008. [a] Literate but did not attend school are also included. [1] Among women aware of diarrhoea management. ** Unweighted cases.

TABLE 4.11 TREATMENT OF DIARRHOEA

Percentage of children suffered from diarrhoea and sought advice/ treatment according to selected background characteristics, Haryana, 2012-13.

Background characteristics	Children suffered from diarrhoea[1]	Number of children	Given ORS	Children sought advice/treatment	Source of treatment: Government health facility[2]	Source of treatment: Private Health facility[3]	Source of treatment: Other	Number of children**
**Age group**								
Less than 25	4.8	3,550	50.4	66.6	19.9	80.7	1.0	174
25-29	3.7	4,332	38.7	68.7	25.5	76.1	0.0	163
30-34	3.4	1,675	40.6	75.0	27.9	72.3	2.3	56
35-39	3.1	530	59.6	73.8	0.0	100.0	0.0	16
40-49	1.8	149	0.0	66.2	0.0	100.0	0.0	3
**Residence**								
Rural	4.1	6,348	44.7	71.8	21.2	79.7	0.6	267
Urban	3.7	3,888	44.1	62.1	25.3	75.7	1.2	145
**Mother's education**								
Non-literate[a]	3.7	2,605	34.0	68.1	17.7	82.3	1.4	101
Less than 5 years	3.9	190	27.1	67.9	0.0	100.0	0.0	7
5-9 years	4.2	3,011	45.3	67.0	17.6	80.9	1.3	125
10 or more years	4.0	4,429	50.7	70.7	29.0	74.1	0.0	179
**Religion**								
Hindu	3.9	9,143	45.7	68.3	22.9	77.6	0.9	361
Muslim	5.7	761	37.3	72.5	18.6	84.9	0.0	44
Christian	--	9	na	na	na	na	na	0
Sikh	2.0	310	33.3	81.3	15.2	84.8	0.0	6
Jain	--	7	na	na	na	na	na	0
Others	--	6	na	na	na	na	na	0
**Castes/Tribes**								
Scheduled Caste	3.3	3,293	47.3	65.4	13.6	84.0	1.3	108
Schedule Tribes	2.9	540	40.8	69.4	31.2	75.7	0.0	17
Other Backward Classes	4.8	3,026	41.3	68.3	20.4	81.1	0.0	148
Others	4.1	3,377	46.1	72.1	29.6	72.4	1.2	139
**DLHS-4**	4.0	10,236	44.5	68.8	22.3	78.6	0.7	412
**DLHS-3**	16.5	9,011	31.7	81.7	9.0	86.5	6.2	1,209

Note: Table based on women with last two surviving children born since 01.01.2008. [a] Literate but did not attended school are also included. [1] Last two weeks prior to survey. [2] Includes government hospital or dispensary, urban health centre/ urban health post/ urban family welfare centre, community health centre or rural hospital, primary health centre, sub-health centre, ICDS and Govt. AYUSH hospital /clinic. [3] Includes non-governmental hospital/ trust hospital or clinic, private hospital/clinic and private AYUSH hospital /clinic. -- Percentage not shown for less than 10 cases. na: Not applicable.** Unweighted cases.

TABLE 4.12 AWARENESS AND TREATMENT OF ACUTE RESPIRATORY INFECTION (ARI)

Percentage of women who are aware of danger signs of ARI and whose children suffer from ARI and sought advice/treatment according to selected background characteristics, Haryana, 2012-13

			Danger signs of Acute Respiratory Infection (ARI)[1]							Source of treatment[5]			
Background characteristics	Women aware of danger signs of ARI	Number of women**	Difficulty in breathing	Pain in chest and productive cough	Wheezing/ whistling	Rapid breathing	Other Signs[2]	Children suffered from ARI[3]	Children sought advice/ treatment[4]	Government health facility[6]	Private health facility[7]	Others	Number of children**
**Age group**													
15-19	57.4	649	32.3	25.1	14.9	20.0	23.1	4.6	--	--	--	--	181
20-24	67.3	4,300	39.6	31.1	18.9	23.1	26.8	3.5	83.8	29.9	74.8	0.0	3,369
25-29	73.8	5,721	45.5	35.3	23.5	26.5	29.7	3.7	84.2	35.3	64.5	2.2	4,332
30-34	78.0	5,049	48.8	39.0	24.6	28.0	31.9	3.4	87.0	19.9	74.7	1.7	1,675
35-39	78.6	4,867	48.2	38.7	24.6	29.5	31.3	2.5	(84.6)	(9.1)	(90.9)	(0.0)	530
40-44	79.0	3,991	47.2	40.8	25.2	28.1	31.0	3.6	--	--	--	--	121
45-49	78.4	2,837	47.5	37.7	26.1	29.0	31.5	0.0	na	na	na	na	28
**Residence**													
Rural	73.6	16,093	44.0	35.8	21.7	25.5	28.5	3.3	82.1	30.5	70.4	1.1	6,348
Urban	77.9	11,321	48.7	38.2	26.5	29.8	33.1	3.9	89.4	27.8	71.6	2.0	3,888
**Mother's education**													
Non-literate[a]	70.1	9,110	39.6	33.0	19.7	22.2	28.0	3.2	84.4	31.2	70.3	0.0	2,605
Less than 5 years	72.2	447	39.9	31.0	20.3	24.3	20.9	5.5	(90.9)	(10.0)	(90.0)	(0.0)	190
5-9 years	74.3	7,254	44.7	35.8	22.9	27.1	27.1	3.8	83.0	34.4	64.4	1.8	3,011
10 or more years	80.6	10,603	52.3	40.9	27.4	31.6	34.7	3.4	86.1	26.1	74.8	2.2	4,429
**Religion**													
Hindu	76.0	25,199	46.4	37.2	23.6	27.3	30.8	3.5	84.0	29.1	70.1	1.7	9,143
Muslim	59.4	1,221	32.2	27.2	21.1	20.0	21.2	4.0	90.6	32.3	79.2	0.0	761
Christian	70.1	32	44.5	31.4	7.1	21.9	46.3	--	--	--	--	--	9
Sikh	75.9	904	45.5	35.2	22.5	31.3	24.7	1.5	--	--	--	--	310
Jain	80.8	29	48.7	45.9	39.8	41.0	32.9	--	--	--	--	--	7
Others	75.2	29	59.2	29.3	22.9	19.4	36.2	--	--	--	--	--	6
**Castes/Tribes**													
Scheduled Caste	71.3	7,522	41.8	33.7	21.1	25.3	28.7	3.7	84.0	24.7	73.6	2.5	3,293
Scheduled Tribes	77.3	1,433	38.7	33.6	20.3	24.6	40.1	3.2	(88.9)	(43.8)	(62.5)	(0.0)	540
Other Backward Classes	72.8	7,785	45.1	36.0	23.0	25.2	28.6	3.8	87.5	36.0	65.6	1.8	3,026
Others	79.5	10,674	50.2	39.9	26.0	30.2	31.0	3.1	81.0	24.5	76.0	0.0	3,377
**DLHS-4**	75.2	27,414	45.7	36.7	23.5	27.1	30.1	3.5	84.8	29.5	70.9	1.5	10,236
**DLHS-3**	76.9	21,484	62.3	61.8	49.5	38.3	41.0	8.3	88.1	7.4	86.2	6.4	9,011

Note: Table based on women with last two surviving children born since 01.01.2008.[a] Literate but did not attend school are also included. [1] Among women who are aware of any danger signs of ARI. [2] Includes not able to drink or take a feed, excessive drowsy and difficulty to keep awake, running nose and others.[3] Last two weeks prior to survey. [4] Among children with ARI or fever in last two weeks who sought advice/ treatment. [5] Among children who sought advice/treatment. [6] Includes government hospital or dispensary, urban health centre/urban health post/urban family welfare centre, community health centre or rural hospital, primary health centre, sub- health centre, ICDS and Govt. AYUSH hospital/clinic. [7] Includes non-governmental hospital/trust hospital or clinic, private hospital/clinic and private AYUSH hospital/clinic. -- Percentage not shown for less than 10 cases. ** Unweighted cases.

Table 4.13 AWARENESS OF ORS AND ACUTE RESPIRATORY INFECTION (ARI) BY DISTRICTS

Percentage of women by awareness of ORS and percentage of children suffered from diarrhoea and ARI and sought advice/treatment by districts, Haryana, 2012-13.

	Oral Rehydration Therapy/Solution (ORS)			Acute Respiratory Infection(ARI)		
District	Women aware of ORS	Children suffered from diarrhoea[1]	Children sought advice/treatment[2]	Children suffered from ARI[1]	Children sought advice/treatment[2]	Number of children**
Panchkula	84.4	2.7	61.3	4.3	83.3	309
Ambala	72.1	0.8	32.6	0.3	100.0	381
Yamunanagar	67.3	3.0	70.5	3.9	94.4	498
Kuruksherta	72.2	2.3	81.9	0.6	100.0	483
kaithal	58.6	2.1	55.0	5.4	88.6	587
Karnal	65.2	7.6	69.1	3.0	78.2	651
Panipat	52.5	14.3	68.3	5.5	80.7	616
Sonipat	73.8	7.7	62.8	2.9	61.4	604
Jind	59.9	0.7	67.8	3.3	93.4	459
Fatehabad	65.3	4.9	78.7	2.9	89.5	373
Sirsa	68.6	1.7	81.9	2.6	100.0	681
Hisar	69.9	3.1	81.6	2.7	89.6	664
Bhiwani	71.9	3.5	87.6	7.0	93.2	457
Rohtak	74.6	5.6	70.4	5.4	70.9	472
Jhajjar	67.7	1.9	82.3	4.4	72.5	505
Mahendragarh	69.1	3.4	83.3	5.6	96.9	444
Rewari	79.3	2.5	53.9	3.0	100.0	304
Gurgaon	65.6	3.3	28.5	4.9	73.7	370
Faridabad	68.9	1.3	46.4	0.6	100.0	316
Mewat	63.1	2.4	43.4	2.3	85.5	521
Palwa	63.2	2.2	74.2	2.5	84.9	541
**DLHS-4**	68.0	4.0	68.4	3.5	85.2	10,236
**DLHS-3**	43.3	16.5	81.7	8.3	88.1	9,011

Note: Table based on women with last two surviving children born since 01.01.2008.
[1] Last two weeks prior to survey. [2] Among children with ARI or fever in last two weeks sought advice /treatment. ** Unweighted cases.

FAMILY PLANNING

TABLE 5.1 AWARENESS OF CONTRACEPTIVE METHODS

Percentage of ever married and currently married women aged 15-49 years who are aware of specific contraceptive method by place of residence, Haryana, 2012-13.

	Ever married women			Currently married women		
Contraceptive methods	Total	Rural	Urban	Total	Rural	Urban
Any method	93.8	92.7	95.7	93.9	92.8	95.8
Any modern method[1]	93.0	91.8	95.0	93.1	92.0	95.1
Female sterilization	84.0	82.7	86.2	84.0	82.7	86.2
Male sterilization	55.3	51.4	62.0	55.4	51.5	62.1
Intra Uterine Device	54.3	50.6	60.7	54.5	50.8	60.9
Pill	53.5	49.6	60.3	53.8	50.0	60.5
Emergency contraceptive pill	22.9	18.7	30.0	23.0	18.9	30.3
Injectables	25.3	21.7	31.5	25.4	21.8	31.5
Condom	46.8	42.7	53.8	47.2	43.2	54.2
Female condom	14.1	11.1	19.4	14.2	11.2	19.6
Rhythm method	19.4	18.7	20.8	19.5	18.7	20.8
Withdrawal method	12.3	10.3	15.7	12.4	10.4	15.8
Others	1.9	1.9	2.0	1.9	1.9	1.9
**Number of women****	28,776	16,895	11,881	27,414	16,093	11,321

[1] Includes female sterilization, male sterilization, Intra-Uterine Device, pill, condom, female condom, emergency contraceptive Pill and Injectables

** Unweighted cases.

TABLE 5.2 AWARENESS OF CONTRACEPTIVE METHODS

Percentage of currently married women aged 15-49 years who are aware of specific contraceptive method according to selected background characteristics, Haryana, 2012-13.

Background characteristics	Any method	Any modern method	Male sterilization	Female sterilization	IUD	Pill	ECP	Injectables	Condom	Female condom	Rhythm method	Withdrawal method	Other	Number of women**
**Age group**														
15-24	90.2	89.3	53.6	77.2	52.3	54.6	23.1	25.0	47.3	14.4	18.9	10.8	1.9	4,949
25-29	93.9	93.3	57.5	83.5	56.7	57.2	23.9	26.8	51.6	14.9	20.1	13.4	1.8	5,721
30-34	94.7	93.8	55.3	84.4	55.5	54.9	24.4	25.8	49.9	15.1	20.3	12.6	1.8	5,049
35-39	95.2	94.6	55.7	86.2	53.8	52.4	23.2	24.5	46.0	14.2	19.4	12.7	1.9	4,867
40-49	95.1	94.1	54.8	87.6	53.8	50.7	21.2	24.8	42.4	13.0	18.9	12.3	2.1	6,828
**No. of living children**														
0	87.5	86.3	51.5	73.3	49.0	50.7	24.6	24.3	44.7	15.5	17.9	10.0	2.4	2,825
1	93.3	92.7	56.8	81.4	57.8	57.6	25.2	27.6	53.1	16.4	19.5	12.4	1.9	5,058
2	95.8	95.0	56.5	86.1	56.7	55.9	24.2	26.7	49.8	14.8	20.8	13.4	2.0	10,250
3	95.0	94.2	55.0	87.1	52.7	51.7	21.1	23.9	43.9	12.5	18.6	11.9	1.8	5,940
4+	92.4	91.8	53.9	85.0	50.4	48.3	18.4	21.6	38.3	11.4	18.4	11.9	1.6	3,341
**Residence**														
Rural	92.8	92.0	51.5	82.7	50.8	50.0	18.9	21.8	43.2	11.2	18.7	10.4	1.9	16,093
Urban	95.8	95.1	62.1	86.2	60.9	60.5	30.3	31.6	54.2	19.6	20.9	15.8	1.9	11,321
**Education**														
Non-literate[a]	89.5	88.1	49.0	80.3	45.9	43.8	16.2	18.9	34.8	9.3	15.5	9.1	2.2	9,110
Less than five years	96.2	95.7	57.0	87.6	55.6	56.7	21.9	25.5	49.8	13.2	22.4	14.1	1.9	4,085
5-9 years	95.8	95.4	59.1	85.8	56.2	57.7	24.5	27.3	51.1	14.9	20.2	13.1	2.1	3,616
10 or more years	96.2	95.7	59.2	85.2	61.1	60.5	29.2	30.5	56.1	19.0	21.7	14.4	1.6	10,603
**Religion**														
Hindu	94.6	93.8	56.1	84.7	55.2	54.4	23.8	26.1	47.9	14.7	20.0	12.7	1.9	25,199
Muslim	77.8	76.7	45.7	65.9	35.8	37.3	12.4	13.3	28.8	9.2	9.7	5.7	2.2	1,221
Christian	86.9	86.9	61.5	78.5	66.7	58.0	15.9	15.9	35.3	12.2	16.0	5.4	0.0	32
Sikh	97.2	96.6	50.5	88.6	58.8	60.7	15.6	21.4	51.9	9.3	18.9	12.2	1.7	904
Jain	96.5	96.5	57.2	88.6	72.1	66.1	45.2	33.5	53.1	17.8	24.8	19.8	0.0	29
Others	--	--	--	--	--	--	--	--	--	--	--	--	--	09
**Castes/Tribes**														
Scheduled Caste	92.5	91.8	53.5	83.3	50.5	51.1	19.7	22.9	43.2	12.9	17.6	10.5	1.7	7,522
Scheduled Tribes	92.4	90.4	43.7	75.7	40.1	41.8	18.8	19.9	36.3	12.7	12.0	4.7	3.1	1,433
Other Backward Classes	93.3	92.8	59.5	84.3	56.3	55.2	23.8	26.8	49.0	14.8	21.0	14.9	2.3	7,785
Others	95.6	94.6	55.4	85.4	57.9	56.6	25.5	26.9	50.3	15.1	20.8	12.9	1.6	10,674
**DLHS-4**	93.9	93.1	55.4	84.0	54.4	53.8	23.0	25.4	47.2	14.2	19.5	12.4	1.9	27,414
**DLHS-3**	100.0	99.9	95.9	99.8	93.3	96.1	24.0	71.5	93.5	6.2	69.4	51.6	0.6	20,394

[a] Literate but did not attend school are also included. IUD = Intra-Uterine Device; ECP = Emergency Contraceptive Pill. -- Percentage not shown for less than 10 cases. ** unweighted cases.

TABLE 5.3 AWARENESS OF CONTRACEPTIVE METHODS BY DISTRICT

Percentage of currently married women aged 15-49 years who are aware of specific contraceptive method by district, Haryana, 2012-13.

District	Any method	Any modern method	Male sterilization	Female sterilization	IUD	Pill	ECP	Inject-able	Condom	Female condom	Rhythm method	Withdrawal method	Other	Number of women**
Panchkula	97.7	97.2	45.3	84.7	46.8	41.2	16.4	16.7	47.9	10.8	6.5	2.0	0.4	945
Ambala	98.6	97.8	47.3	90.2	46.3	43.9	12.2	12.9	50.5	10.2	11.1	6.5	5.1	1,247
Yamunanagar	98.7	97.9	54.5	90.8	49.3	58.4	12.1	14.9	40.7	11.4	15.9	8.3	7.1	1,212
Kukshetra	83.4	80.5	48.5	65.8	40.2	47.6	13.7	20.6	37.6	13.9	13.2	10.8	3.2	1,586
Kaithal	97.1	96.8	58.6	82.9	57.0	47.6	20.3	20.7	48.8	9.6	6.0	1.0	0.1	1,488
Karnal	88.8	88.1	46.7	75.6	44.3	55.3	18.4	21.6	43.8	17.9	18.6	16.0	5.6	1,694
Panipat	97.2	93.6	38.3	80.3	38.6	53.1	13.8	15.6	39.4	15.1	13.1	11.5	5.6	1300
Sonipat	95.1	94.9	31.7	90.5	28.9	24.1	11.2	12.3	25.6	8.1	6.8	3.2	0.6	1,458
Jind	91.9	91.5	64.3	79.0	58.1	43.9	19.4	17.7	35.4	8.8	8.6	4.0	0.9	1,416
Fatehabad	97.6	97.5	36.6	91.5	63.1	62.5	10.9	9.4	44.7	4.2	14.7	5.2	0.5	971
Sirsa	99.6	99.5	47.5	95.8	74.7	70.1	17.5	24.3	61.6	8.3	23.2	14.0	1.0	1,665
Hisar	100.0	100.0	86.4	99.7	90.5	90.8	40.3	51.0	75.5	16.6	53.6	34.9	0.2	1,539
Bhiwani	100.0	100.0	95.7	99.5	94.7	90.4	44.0	52.5	73.9	15.4	52.9	46.0	0.2	1,162
Rohtak	98.8	98.7	48.0	92.5	47.6	41.4	24.5	25.5	43.1	17.7	12.7	5.3	0.4	1,302
Jhajjar	99.4	99.3	63.1	87.8	64.9	66.9	43.0	41.7	57.8	27.9	23.0	10.8	0.5	1,467
Mahendragarh	99.8	99.8	86.1	97.1	87.4	86.5	67.3	71.5	87.6	25.4	60.5	48.7	0.9	1,162
Rewari	89.6	88.3	50.2	72.3	36.1	34.7	22.9	20.7	35.6	16.1	12.5	9.9	2.8	1,081
Gurgaon	96.7	96.7	69.1	86.4	66.3	65.7	36.1	38.2	54.3	27.5	23.0	4.4	0.3	1,274
Faridabad	88.6	88.4	60.8	77.7	51.6	48.9	29.8	29.0	47.3	24.1	16.3	6.7	0.2	1,150
Mewat	78.0	77.4	51.3	65.5	28.5	26.4	10.7	11.0	18.9	8.5	8.0	6.6	0.7	1,112
Palwal	76.6	73.3	42.8	59.8	31.9	31.9	11.4	10.0	18.8	8.3	8.2	7.1	3.2	1,183
**DLHS-4**	94.0	93.1	55.4	84.0	54.4	53.8	23.0	25.4	47.2	14.2	19.5	12.4	1.9	27,414
**DLHS-3**	100.0	99.9	95.9	99.8	93.3	96.1	24.3	71.5	93.5	6.2	69.4	51.6	0.6	20,394

Note: IUD = Intra-Uterine Device; ECP = Emergency Contraceptive Pill.
** Unweighted cases.

TABLE 5.4 EVER USE OF CONTRACEPTIVE METHOD

Percentage of currently married women aged 15-49 years who ever used specific contraceptive method according to selected background characteristics, Haryana, 2012-13.

Background characteristics	Any method	Any modern method	Male sterilization	Female sterilization	IUD	Pill	ECP	Inject-ables	Condom	Female condom	Rhythm method	Withdrawal method	Other	Number of women**
**Age group**														
15 - 19	13.9	11.2	0.4	1.5	0.9	3.1	0.0	0.3	6.2	0.4	2.7	1.4	0.0	649
20 - 24	28.9	25.2	0.4	6.6	2.1	4.7	0.4	0.2	13.2	0.7	4.3	1.4	0.1	4,300
25 - 29	49.6	46.5	0.6	19.9	4.7	6.9	0.8	0.4	19.5	0.8	4.8	2.3	0.1	5,721
30 - 34	60.8	58.4	1.8	33.3	5.6	5.9	1.2	0.5	16.9	0.7	4.2	1.7	0.0	5,049
35 - 39	66.4	64.6	1.7	46.6	4.6	5.0	1.0	0.4	13.2	0.7	3.7	1.3	0.1	4,867
40 - 44	69.7	67.8	1.7	54.4	4.4	4.1	0.8	0.6	10.1	0.3	4.1	1.4	0.0	3,991
45 – 49	68.4	66.6	1.7	57.8	3.2	2.5	0.8	0.4	6.9	0.2	3.5	1.4	0.1	2,837
**No. of living children**														
0	11.5	9.7	0.2	2.1	0.4	2.2	0.2	0.1	5.9	0.3	1.9	1.0	0.0	2,825
1	41.2	37.8	0.4	9.0	4.5	6.2	0.9	0.5	20.6	1.0	4.5	2.0	0.0	5,058
2	64.2	61.5	1.4	37.9	5.7	6.2	1.0	0.5	17.0	0.7	4.9	2.0	0.2	10,250
3	68.0	65.9	2.0	50.8	3.9	4.5	0.9	0.3	10.7	0.5	3.9	1.2	0.0	5,940
4+	63.5	61.7	1.6	51.1	2.6	3.7	0.5	0.5	7.2	0.3	3.7	1.1	0.0	3,341
**Residence**														
Rural	55.1	52.8	1.4	36.5	3.6	4.8	0.8	0.4	10.8	0.5	4.6	1.0	0.0	16,093
Urban	55.7	52.8	1.0	27.9	5.1	5.6	0.9	0.5	19.3	0.7	3.3	2.6	0.1	11,321
**Education**														
Non-literate	54.6	52.6	1.3	43.3	1.8	2.7	0.7	0.3	6.3	0.3	3.7	0.9	0.1	9,110
Less than five years	62.3	59.0	1.7	41.5	3.8	5.0	0.5	0.4	12.0	0.6	4.6	1.5	0.1	4,085
5-9 years	55.0	52.4	1.2	31.8	4.7	6.3	1.0	0.4	13.8	0.5	4.5	1.4	0.0	3,616
10 or more years	53.2	50.5	1.0	21.7	6.2	6.9	1.1	0.6	21.6	0.9	4.2	2.4	0.1	10,603
**Religion**														
Hindu	56.6	54.1	1.3	34.6	4.1	5.2	0.8	0.4	14.1	0.6	4.2	1.6	0.1	25,199
Muslim	27.5	24.9	0.7	12.4	2.1	4.3	0.6	0.5	7.7	0.3	3.2	0.8	0.0	1,221
Christian	52.7	52.7	0.0	17.5	12.4	9.8	2.7	3.7	17.3	0.0	0.0	2.7	0.0	32
Sikh	58.5	54.8	0.9	28.8	6.7	3.7	1.1	0.5	17.5	0.3	5.1	2.5	0.2	904
Jain	70.8	70.8	0.0	36.0	9.3	9.9	0.0	0.0	27.7	3.7	3.2	6.4	0.0	29
Others	---	---	---	---	---	---	---	---	---	---	---	---	---	09
**Castes/Tribes**														
Scheduled Caste	53.1	50.7	1.2	35.6	2.6	4.2	0.8	0.3	10.9	0.5	4.0	1.1	0.1	7,522
Scheduled Tribes	41.8	39.7	1.0	25.6	2.2	6.2	0.5	0.1	8.8	0.9	3.2	0.5	0.0	1,433
Other Backward Classes	54.9	52.4	1.5	34.1	3.9	4.8	0.7	0.5	13.0	0.6	4.0	1.8	0.2	7,785
Others	59.1	56.5	1.1	32.3	5.7	5.8	1.0	0.5	17.5	0.7	4.5	2.0	0.0	10,674
**DLHS-4**	55.3	52.8	1.2	33.4	4.1	5.1	0.8	0.4	13.9	0.6	4.1	1.6	0.1	27,414
**DLHS-3**	74.1	65.2	1.3	38.6	10.3	10.6	0.5	0.5	21.9	0.1	23.0	10.2	0.3	20,394

Note: IUD = Intra-Uterine Device; ECP = Emergency Contraceptive Pill. -- Percentage not shown for less than 10 cases. ** Unweighted cases.

TABLE 5.5 (A) CURRENT USE OF CONTRACEPTIVE METHODS

Percentage of currently married women aged 15-49 years who are currently using specific contraceptive method according to selected background characteristics, Haryana, 2012-13.

Background characteristics	Any method	Any modern method	Male sterilization	Female sterilization	IUD	Pill	ECP	Condom	Rhythm method	Withdrawal method	Other	Number of women**
**Age group**												
15 - 19	12.5	8.3	0.2	1.5	0.4	0.7	0.0	5.2	0.9	1.1	2.3	649
20 - 24	23.8	20.5	0.3	6.7	1.6	2.0	0.5	9.8	1.3	0.5	1.5	4,300
25 - 29	45.0	41.2	0.5	20.0	3.1	2.9	1.0	14.7	1.5	0.7	1.5	5,721
30 - 34	57.2	54.1	1.5	33.5	3.5	2.3	1.5	13.0	1.0	0.7	1.4	5,049
35 - 39	63.5	60.8	1.3	46.8	2.5	1.5	1.2	8.7	1.0	0.4	1.2	4,867
40 - 44	66.6	64.4	1.2	54.5	1.6	1.1	0.9	5.8	1.0	0.4	0.7	3,991
45 – 49	65.8	64.0	1.1	58.1	1.2	0.5	0.9	3.1	0.5	0.4	0.9	2,837
**No. of living children**												
No children	8.8	6.6	0.2	2.3	0.0	0.4	0.3	3.7	0.4	0.3	1.5	2,825
1 child 1 son	39.4	35.6	0.5	11.4	4.1	2.8	1.1	16.6	1.4	0.8	1.6	3,324
1 child No son	29.0	25.3	0.1	5.0	1.8	2.1	1.0	15.9	1.3	1.0	1.5	1,734
2 children												
1 or more sons	62.3	58.9	1.1	40.7	3.2	2.2	1.2	11.6	1.3	0.6	1.4	9,384
No sons	34.7	30.4	0.2	9.7	1.6	2.4	1.7	16.3	1.9	0.9	1.5	866
3 children												
1 or more sons	66.7	64.3	1.5	52.6	2.0	1.5	1.1	6.7	0.9	0.4	1.1	5,711
No sons	31.7	27.8	0.0	13.0	0.8	2.7	0.0	10.1	2.6	1.0	0.3	229
4+ children												
1 or more sons	62.3	60.5	1.3	52.8	1.2	1.2	0.6	4.1	0.8	0.4	0.6	3,230
No sons	25.1	22.1	2.5	9.4	2.7	0.7	0.0	6.9	0.9	2.1	0.0	111
**Residence**												
Rural	52.0	49.2	1.0	36.7	2.1	1.7	0.9	7.6	1.0	0.4	1.4	16,093
Urban	50.9	47.7	0.9	28.0	2.8	2.0	1.1	13.8	1.2	0.8	1.1	11,321
**Education**												
Non-literate[a]	52.5	50.5	1.0	43.5	0.8	1.1	0.7	4.1	0.9	0.3	0.8	9,110
Less than five years	58.6	55.8	1.2	41.6	2.2	1.8	0.6	8.8	1.3	0.5	1.1	4,085
5-9 years	51.5	47.8	1.1	31.8	2.6	2.5	1.2	9.7	1.3	0.6	1.7	3,616
10 or more years	48.0	44.3	0.8	21.8	3.7	2.2	1.4	15.5	1.1	0.9	1.7	10,603
**Religion**												
Hindu	52.8	49.9	1.0	34.8	2.3	1.8	1.0	9.9	1.1	0.6	1.3	25,199
Muslim	24.0	22.5	0.7	12.5	1.2	2.2	0.6	5.6	1.1	0.1	0.4	1,221
Christian	40.7	40.7	0.0	17.5	8.6	4.1	2.9	6.8	0.0	0.0	0.0	32
Sikh	55.2	50.8	0.6	28.8	5.1	1.7	1.3	14.4	1.9	1.1	1.4	904
Jain	58.9	58.9	0.0	36.0	6.0	3.9	0.0	13.0	0.0	0.0	0.0	29
Others	---	---	---	---	---	---	---	---	---	---	---	09

Contd …

TABLE 5.5 (A) CURRENT USE OF CONTRACEPTIVE METHODS —*Continued*

Background characteristics	Any method	Any modern method	Male sterilization	Female sterilization	IUD	Pill	ECP	Condom	Rhythm method	Withdrawal method	Other	Number of women**
**Castes/Tribes**												
Scheduled Caste	50.4	47.5	1.0	35.8	1.3	1.7	0.9	7.7	1.1	0.4	1.3	7,522
Scheduled Tribes	39.0	35.0	0.7	25.7	1.1	1.6	0.6	6.0	0.9	0.1	2.9	1,433
Other Backward Classes	52.2	48.6	1.1	34.3	2.2	1.6	0.8	9.3	1.2	0.6	1.7	7,785
Others	53.9	51.4	0.8	32.5	3.4	2.2	1.2	12.3	1.1	0.7	0.7	10,674
**DLHS-4**	51.6	48.6	0.9	33.6	2.3	1.8	1.0	9.8	1.1	0.6	1.3	27,414
**DLHS-3**	62.9	55.7	1.2	38.5	3.5	2.1	0.5	9.8	4.8	2.3	0.0	20,394

Note: IUD = Intra-Uterine Device; ECP = Emergency Contraceptive Pill.
[a] Literates but did not attend school, are also included. -- Percentage not shown for less than 10 cases. ** Unweighted cases.

TABLE 5.5 (B) DURATION OF USE OF SPACING METHODS

Percentage of currently married women aged 15-49 years who are currently using spacing method by duration of use according to selected background characteristics, Haryana, 2012-13.

	IUD					Pill		Condom	
Background characteristics	< 6 months	6 months to 2 years	2-3 years	4 or more years	Number of IUD users**	> 6 months	Number of Pill users**	> 6 months	Number of condom users
**Age group**									
15 - 19	---	---	---	---	02	---	05	26.5	34
20 - 24	16.1	33.7	17.5	13.9	70	37.5	85	44.7	433
25 - 29	9.9	18.4	20.0	34.1	173	56.8	163	61.0	867
30 - 34	4.5	12.7	13.5	60.1	183	64.4	120	71.6	686
35 - 39	1.5	5.4	4.6	71.6	123	68.2	74	74.5	442
40 - 44	1.7	5.9	11.9	77.2	66	78.8	45	72.0	241
45 - 49	8.7	7.5	0.0	69.4	34	(78.1)	14	68.2	94
**No. of living children**									
0	---	---	---	---	01	(14.8)	12	44.0	111
1	6.7	15.4	16.3	46.4	167	55.0	130	57.4	855
2	7.8	12.0	12.6	55.6	319	62.9	231	68.7	1,270
3	6.0	17.4	12.1	47.7	122	58.9	93	64.1	419
4+	0.0	16.9	7.9	63.5	42	60.0	40	73.2	142
**Residence**									
Rural	7.9	16.2	14.0	48.4	336	53.2	280	59.4	1,225
Urban	5.2	11.5	12.0	57.1	315	66.8	226	68.0	1,572
**Education**									
Non-literate[a]	5.3	18.0	12.6	48.1	79	58.5	103	58.2	384
Less than five years	10.2	18.9	11.0	42.3	88	58.5	76	59.9	367
5-9 years	5.7	17.2	11.4	55.1	100	56.5	91	63.5	358
10 or more years	6.5	11.5	14.2	54.7	384	59.8	236	66.1	1,688
**Religion**									
Hindu	7.2	13.4	12.9	52.0	581	58.6	458	63.8	2,580
Muslim	(0.0)	(33.3)	(13.3)	(40.0)	15	68.5	29	53.9	72
Christian	---	---	---	---	03	---	01	---	02
Sikh	4.3	17.5	15.2	59.3	48	(47.0)	17	68.6	135
Jain	---	---	---	---	02	---	01	---	04
Others	---	---	---	---	---	---	---	---	02
**Castes/Tribes**									
Scheduled Caste	8.7	18.6	10.6	37.1	99	53.1	128	55.5	596
Scheduled Tribes	(12.5)	(0.0)	(18.8)	(37.5)	16	23.4	22	50.3	89
Other Backward Classes	8.6	12.2	11.6	52.8	182	55.3	125	67.8	752
Others	4.9	14.5	14.4	56.9	354	67.3	231	66.3	1,360
**DLHS-4**	6.7	14.2	13.1	52.2	651	58.7	506	63.8	2,797
**DLHS-3**	NA	NA	NA	NA	722	68.2	530	71.5	2,005

Note: IUD = Intra-Uterine Device. [a] Literates but did not attend school, are also included. ( ) Based on 10-20 cases. -- Percentage not shown for less than 10 case. NA: Not available. ** Unweighted cases.

TABLE 5.6 AGE AT THE TIME OF STERILIZATION

Percent distribution of women aged 15-49 years by age at the time of sterilization, according to selected background characteristics, Haryana, 2012-13.

Background characteristics	Age at the time of sterilization <20	20-24	25-29	30-34	35-39	40 +	Total[1]	Mean age of sterilization	Number of women**
**Years since sterilization**									
<2	0.8	19.7	39.2	20.2	10.7	9.5	100.0	29.43	537
2-3	1.4	22.2	35.7	20.8	11.5	8.4	100.0	29.34	816
4-5	2.0	23.8	32.2	23.1	13.4	5.5	100.0	28.85	933
6-7	4.0	28.5	31.8	21.6	11.5	2.6	100.0	28.17	852
8-9	3.0	22.0	35.3	25.1	11.9	2.7	100.0	28.36	865
10+	5.0	29.6	40.5	20.4	4.5	0.0	100.0	26.40	3,612
**No. of living children**									
0	12.6	21.0	19.7	11.6	16.4	18.8	100.0	29.86	66
1	5.1	27.0	24.8	14.5	14.0	14.6	100.0	29.67	486
2	4.4	30.5	31.8	16.9	8.0	8.4	100.0	28.07	3,960
3	1.9	19.7	36.0	22.4	10.6	9.4	100.0	29.47	3,093
4+	0.8	8.5	31.1	27.7	17.7	14.2	100.0	31.87	1,754
**Residence**									
Rural	3.1	24.6	32.8	19.5	10.4	9.6	100.0	28.96	6,077
Urban	2.7	18.0	32.1	23.2	12.6	11.5	100.0	30.05	3,282
**Education**									
Non-literate[a]	2.8	19.3	30.6	21.6	12.4	13.3	100.0	30.18	4,042
Less than five years	3.3	28.3	34.1	18.2	8.9	7.1	100.0	28.15	1,732
5-9 years	3.8	27.4	36.1	18.4	8.6	5.6	100.0	27.96	1,186
10 or more years	2.6	21.3	33.3	21.8	11.5	9.4	100.0	29.47	2,399
**Religion**									
Hindu	2.9	22.6	32.8	20.6	11.1	10.0	100.0	29.31	8,912
Muslim	2.7	25.6	22.1	25.5	10.1	13.9	100.0	30.30	163
Christian	---	---	---	---	---	---	---	---	06
Sikh	6.5	19.4	31.8	18.5	11.8	12.0	100.0	29.29	258
Jain	(0.0)	(0.0)	(45.5)	(18.2)	(0.0)	(36.4)	(100.0)	(34.45)	11
Others	---	---	---	---	---	---	---	---	02
**Castes/Tribes**									
Scheduled Caste	2.8	20.8	32.3	21.6	11.9	10.5	100.0	29.53	2,746
Scheduled Tribes	3.9	22.2	29.3	17.5	16.1	11.1	100.0	29.89	376
Other Backward Classes	3.3	25.6	33.8	19.3	9.9	8.2	100.0	28.69	2,737
Others	2.8	21.6	32.3	21.1	10.8	11.4	100.0	29.64	3,500
**DLHS-4**	3.0	22.5	32.6	20.6	11.1	10.2	100.0	29.34	9,359
**DLHS-3**	2.3	32.4	42.1	18.5	4.0	0.7	100.0	26.6	8,193

Note: Total may not add up to N due to missing cases. [a] Literates but did not attend school, are also included.
[1] Total figure may not add to 100 percent due to 'don't know' or 'missing cases. ( ) Based on 10-20 unweighted cases. -- Percentage not shown for less than 10 cases.** Unweighted cases.

TABLE 5.7 CONTRACEPTIVE PREVALENCE RATE BY DISTRICT

Percentage of currently married women aged 15-49 years who are currently using any contraceptive method, by districts, Haryana, 2012-13.

District	Any method	Any modern method	Male sterilization	Female sterilization	IUD	Pill	Condom	Rhythm method	Withdrawal method	Other	Number of Women**
Panchkula	57.4	55.0	1.2	32.5	1.9	1.3	17.9	1.2	0.2	1.0	945
Ambala	64.3	61.5	0.2	39.3	2.4	1.3	18.3	2.3	0.5	0.0	1,247
Yamunanagar	52.8	48.3	0.8	26.5	2.3	3.3	15.4	3.9	0.5	0.2	1,212
Kukshetra	40.6	35.4	0.7	19.8	2.6	1.6	10.5	0.7	0.2	4.3	1,586
Kaithal	55.9	54.2	1.1	34.3	2.3	1.3	15.1	1.2	0.2	0.4	1,488
Karnal	43.3	39.4	2.7	22.5	1.7	3.4	9.1	0.6	0.8	2.5	1,694
Panipat	42.7	39.6	0.9	23.3	2.0	3.6	9.3	1.6	0.6	1.0	1,300
Sonipat	53.2	51.6	1.4	39.9	3.1	1.7	5.4	0.6	0.4	0.5	1,458
Jind	51.6	49.3	0.9	30.9	2.1	2.5	12.7	1.0	0.3	1.0	1,416
Fatehabad	60.3	56.5	0.5	38.9	3.7	2.1	11.2	2.1	1.3	0.3	971
Sirsa	67.3	64.1	0.6	43.3	4.6	2.1	13.6	1.2	1.5	0.6	1,665
Hisar	65.3	62.5	0.9	48.7	3.3	2.1	7.5	1.3	1.5	0.1	1,539
Bhiwani	68.6	64.9	0.3	47.2	3.8	1.7	11.8	1.6	1.4	0.7	1,162
Rohtak	52.9	51.3	1.3	39.3	2.6	1.2	6.8	0.4	0.4	0.8	1,302
Jhajjar	49.9	48.5	1.0	36.1	2.5	2.5	6.3	0.7	0.4	0.4	1,467
Mahendragarh	69.7	65.4	2.3	46.1	2.2	1.4	13.2	2.0	1.4	0.9	1,162
Rewari	43.4	35.7	0.6	27.8	1.7	1.0	4.4	0.0	0.1	7.7	1,081
Gurgaon	46.8	45.6	0.8	33.6	0.9	1.3	8.5	0.8	0.2	0.2	1,274
Faridabad	41.5	39.3	0.2	30.1	0.7	0.9	6.9	0.7	0.0	1.5	1,150
Mewat	23.5	21.7	0.4	16.5	0.6	0.5	3.4	0.1	0.1	1.5	1,112
Palwal	28.4	26.4	0.3	17.6	2.0	0.7	5.8	0.1	0.1	1.8	1,183
**DLHS-4**	51.6	48.6	0.9	33.6	2.3	1.8	9.8	1.1	0.6	1.3	27,414
**DLHS-3**	62.9	55.7	1.2	38.5	3.5	2.1	9.8	4.8	2.3	0.0	20,394

Note: IUD = Intra Uterine Device;
** Unweighted cases.

TABLE 5.8 SOURCES OF MODERN CONTRACEPTIVE METHODS

Percent distribution of currently married women aged 15-49 years who are currently using modern contraceptive methods by source according to selected background characteristics, Haryana, 2012-13.

Background Characteristics	Spacing Method				Number of women**	Limiting method				Number of women**
	Govern-ment[2]	Private[3]	Other[4]	Total[1]		Govern-ment[5]	Private[6]	Other[7]	Total[1]	
**Age group**										
15 - 19	28.8	33.6	37.7	100.0	43	(70.0)	(20.0)	(10.0)	100.0	11
20 - 24	28.7	35.9	35.4	100.0	594	76.6	23.4	0.0	100.0	293
25 - 29	23.9	40.6	35.5	100.0	1,211	78.9	20.9	0.2	100.0	1,140
30 - 34	21.5	44.0	34.6	100.0	999	78.7	21.2	0.1	100.0	1,731
35 - 39	22.2	43.9	33.9	100.0	647	78.3	21.7	0.1	100.0	2,318
40 - 44	23.2	41.0	35.8	100.0	363	79.7	20.1	0.2	100.0	2,198
45 – 49	20.2	34.9	44.9	100.0	144	80.5	19.4	0.1	100.0	1,668
**No. of living children**										
0	16.6	46.3	37.2	100.0	125	72.6	25.7	1.7	100.0	66
1	23.2	43.3	33.5	100.0	1,168	69.3	30.7	0.0	100.0	486
2	22.7	42.2	35.1	100.0	1,839	76.1	23.7	0.1	100.0	3,960
3	29.1	32.1	38.8	100.0	642	81.6	18.2	0.2	100.0	3,093
4+	24.8	38.8	36.4	100.0	227	84.2	15.8	0.0	100.0	1,754
**Residence**										
Rural	32.7	37.1	30.2	100.0	1,862	81.8	18.1	0.2	100.0	6,077
Urban	14.9	44.8	40.3	100.0	2,139	73.1	26.8	0.1	100.0	3,282
**Education**										
Non-literate	28.0	31.5	40.5	100.0	574	84.0	15.8	0.2	100.0	4,042
Less than five years	31.7	35.0	33.3	100.0	539	82.6	17.4	0.0	100.0	1,732
5-9 years	26.9	39.1	33.9	100.0	553	80.7	19.2	0.1	100.0	1,186
10 or more years	20.0	45.2	34.8	100.0	2,335	66.7	33.1	0.2	100.0	2,399
**Religion**										
Hindu	24.2	40.9	34.9	100.0	3,660	79.2	20.6	0.1	100.0	8,912
Muslim	26.5	34.6	38.9	100.0	120	78.4	21.6	0.0	100.0	163
Christian	--	--	--	--	07	--	--	--	--	06
Sikh	13.8	45.2	41.0	100.0	201	75.6	23.9	0.5	100.0	258
Jain	--	--	--	--	07	(54.5)	(45.5)	(0.0)	100.0	11
Others	--	--	--	--	02	--	--	--	--	02
**Castes/Tribes**										
Scheduled Caste	30.2	35.7	34.1	100.0	834	85.6	14.4	0.1	100.0	2,746
Scheduled Tribes	41.1	37.1	21.8	100.0	127	79.7	19.5	0.8	100.0	376
Other Backward Classes	25.3	37.6	37.0	100.0	1,073	81.9	18.0	0.0	100.0	2,737
Others	18.7	45.5	35.8	100.0	1,967	71.5	28.3	0.2	100.0	3,500
**DLHS-4**	23.7	40.9	35.3	100.0	4,001	79.1	20.8	0.1	100.0	9,359
**DLHS-3**	18.7	46.6	34.7	100.0	2,552	88.1	11.6	0.2	100.0	8,193

Note: Spacing method includes pill (Daily/Weekly), condom (Male/Female), and Injectables and limiting method includes – male sterilization and female sterilization.
[a] Literates but did not attend school, are also included. [1] Total figure may not add to 100 percent due to 'missing cases'.[2] Hospital, dispensary, UHC/UHP/UFWC, CHC/ Rural Hospital, PHC, Sub-Health Centre/ANM, Mobile clinic, *Anganwadi* / ICDS centre, ASHA, Other Community Based Worker, AYUSH Hospital/Clinic and Other Public Health Sector. [3] Hospital, Doctor/Clinic, Mobile Clinic, AYUSH Hospital/Clinic, Traditional Healer, Pharmacy/Drugstore and Other Private Medical Sector. [4] NGO or Trust Hospital/clinic Private, Shop, Vending Machine, Husband, Relatives/Friends, Others and Don't Know. [5]Hospital, Dispensary, CHC/Rural Hospital, PHC, Mobile Clinic, Camp and Other Public Sector Health Facility. [6] Hospital, Doctor/Clinic, Mobile Clinic and Other Private Health Facility.[7] NGO or Trust Hospital/Clinic, Other and Don't Know. ( ) Base on 10-20 unweighted cases. -- Percentage not shown for less than 10 cases.** Unweighted cases.

TABLE 5.9 CASH BENEFITS RECEIVED AFTER STERILIZATION

Percent distribution of currently married women and wives of sterilized men who received cash benefits after sterilization, by districts, Haryana, 2012-13.

District	Received cash benefits	Cash benefits received: At the time of discharge	At the time of first follow-up	After several visits	Total (100%)	Number of women**
Panchkula	28.7	77.4	18.3	4.3	100.0	319
Ambala	30.0	82.8	5.3	11.9	100.0	489
Yamunanagar	39.7	87.1	8.3	4.5	100.0	327
Kukshetra	44.0	82.2	10.3	7.5	100.0	321
Kaithal	34.1	68.5	15.5	16.0	100.0	518
Karnal	53.7	87.1	10.3	2.6	100.0	427
Panipat	36.9	81.9	9.5	8.6	100.0	319
Sonipat	16.8	85.7	6.3	8.0	100.0	606
Jind	30.2	72.2	17.4	10.4	100.0	463
Fatehabad	42.6	81.4	15.6	3.0	100.0	383
Sirsa	38.1	87.1	8.4	4.5	100.0	727
Hisar	26.7	78.0	13.9	8.1	100.0	769
Bhiwani	38.9	77.1	15.0	7.9	100.0	549
Rohtak	21.4	75.2	16.5	8.3	100.0	532
Jhajjar	33.1	85.7	7.1	7.1	100.0	542
Mahendragarh	51.1	93.9	3.4	2.7	100.0	565
Rewari	51.0	63.9	30.4	5.7	100.0	308
Gurgaon	23.4	89.5	2.9	7.6	100.0	441
Faridabad	25.9	79.1	8.8	12.1	100.0	347
Mewat	49.5	76.8	16.8	6.3	100.0	193
Palwal	52.6	82.3	9.7	8.0	100.0	214
**DLHS-4**	35.0	81.4	11.6	7.0	100.0	9,358
**DLHS**-3	66.7	93.7	4.7	1.7	100.0	8,193

** Unweighted cases.

TABLE 5.10 HEALTH PROBLEMS WITH CURRENT USE OF CONTRACEPTION AND TREATMENT RECEIVED

Percentage of currently married women aged 15-49 years who are currently using contraceptive method and who were informed about side effects, had side effects with the method, treatment taken for side effect with the method, Haryana, 2012-13.

Health problems/side effect	Type of method: Female sterilization	IUD	Pill
Women who were informed about the side effects before adoption of the method	10.6	20.1	14.5
Women who had side-effect/health problem due to use of contraceptive method	2.6	4.4	2.0
**Number of current users****	**9105**	**651**	**506**
**Type of health problems/side effects[1]**			
Weakness/inability to work	29.4	5.8	(20.0)
Body ache/ backache	18.7	24.7	(20.0)
Abdominal pain	26.7	12.9	(10.0)
Weight gain	16.4	11.0	--
Dizziness	4.5	0.0	--
Nausea/vomiting	2.6	3.7	(20.0)
Fever	11.1	0.0	(20.0)
Breast tenderness	0.9	3.5	--
Irregular periods	10.1	17.0	(20.0)
Excessive bleeding	7.0	25.2	--
Spotting	1.0	3.7	--
Amenorrhoea	0.5	3.7	--
Cramps	0.8	3.7	--
Decreased libido	0.0	3.1	--
Rashes/allergy	2.2	0.0	--
Infection	11.5	5.8	--
Others	9.1	7.2	--
**Number of users with side effects****	**237**	**28**	**10**
**Percentage of women received treatment**	64.4	54.8	20.0
Source of treatment			
Government health facility	30.8	(32.3)	--
Private health facility	33.2	(22.6)	--
Other	0.4	--	--
**Number of women with treatment taken****	**150**	**17**	**02**

[1] Percentages may add to more than 100 because of multiple responses. ( ) Based on 10-20 unweighted cases. -- Percentage not shown for less than 10 cases.** Unweighted cases.

TABLE 5.11 REASONS FOR DISCONTINUATION OF CONTRACEPTION

Percent distribution of currently married women aged 15-49 years who are past users (currently non-users) by reason for discontinuation of the contraceptive method according to selected background characteristics, Haryana, 2012-13.

Background characteristics	Reasons for discontinuation			Number of women**
	Fertility related[1]	Side effect related	Others[2]	
**Age group**				
15 - 19	44.0	15.7	40.3	27
20 - 24	44.0	26.9	29.2	292
25 - 29	39.0	23.4	37.6	400
30 - 34	23.0	36.0	41.0	295
35 - 39	17.1	32.4	50.5	231
40 - 44	11.2	30.8	58.0	166
45 - 49	8.4	26.2	65.3	119
**No. of living children**				
0	48.3	24.7	27.0	135
1	40.3	25.4	34.3	376
2	21.9	30.7	47.5	629
3	21.2	30.4	48.4	270
4+	20.5	29.5	50.0	120
**Residence**				
Rural	27.6	31.6	40.7	809
Urban	29.7	24.7	45.6	721
**Education**				
Non-literate[a]	20.4	30.9	48.6	317
Less than five years	33.7	27.9	38.4	227
5-9 years	27.1	34.4	38.5	199
10 or more years	30.8	26.5	42.8	787
**Religion**				
Hindu	29.0	28.8	42.2	1,421
Muslim	29.2	35.6	35.2	58
Christian	--	--	--	04
Sikh	14.5	16.6	68.9	43
Jain	--	--	--	03
Others	--	--	--	--
**Castes/Tribes**				
Scheduled Caste	28.0	29.2	42.8	351
Scheduled Tribes	19.5	43.3	37.2	86
Other Backward Classes	30.0	27.4	42.7	387
Others	29.2	27.2	43.6	706
**DLHS-4**	28.5	28.7	42.8	1,530
**DLHS-3**	69.1	9.9	20.9	2,062

[a] Literates but did not attend school, are also included.[1] Wanted child, method failed/became pregnant. [2] Others include supply not available, difficult to get method, lack of pleasure, method was inconvenient, cost too much, family/husband opposed, not having sex, infrequent sex, husband away and others. -- Percentage not shown for less than 10 cases.** Unweighted cases

TABLE 5.12 FUTURE INTENTION TO USE CONTRACEPTION

Percent distribution of currently married women aged 15-49 years who are not using contraceptive method but having intention to use contraception in future by background characteristics, Haryana, 2012-13.

Background Characteristics	Future intention to use[1] Spacing Method	Limiting Method	Want to use any family planning method Within 12 months	12 months and more	Undecided	Number of non-users**
**Age group**						
15 - 19	3.9	7.1	35.3	26.3	38.5	461
20 - 24	4.8	9.2	29.1	23.0	47.9	2,696
25 - 29	3.6	8.9	33.9	19.3	46.8	2,906
30 - 34	3.2	6.7	41.6	18.5	39.9	2,120
35 - 39	2.1	3.9	49.1	15.4	35.5	1,802
40 - 44	1.3	1.8	42.6	6.8	50.6	1,361
45 - 49	1.4	1.2	54.0	11.4	34.6	1,000
**No. of living children**						
0	3.9	6.2	25.7	21.2	53.0	1,945
1	4.2	7.2	27.9	27.1	45.0	2,946
2	3.0	6.9	42.6	15.1	42.3	4,089
3	2.5	5.5	45.2	13.4	41.4	2,079
4+	1.3	4.9	46.0	18.7	35.4	1,287
**Residence**						
Rural	3.1	7.0	34.7	20.9	44.4	7,128
Urban	3.3	5.4	38.3	17.2	44.5	5,218
**Education**						
Non-literate[a]	1.4	4.5	34.9	19.1	46.0	4,099
Less than five years	3.0	9.7	33.3	20.6	46.1	1,567
5-9 years	3.7	8.5	38.4	17.8	43.8	1,613
10 or more years	4.5	6.3	36.6	20.1	43.3	5,067
**Religion**						
Hindu	3.2	6.6	35.3	19.3	45.3	11,087
Muslim	2.5	2.4	52.2	21.0	26.7	841
Christian	(0.0)	(5.9)	(0.0)	(0.0)	(100.0)	17
Sikh	2.8	7.9	34.4	24.6	41.0	376
Jain	0.0	(20.0)	0.0	(50.0)	(50.0)	10
Others	--	--	--	--	--	05
**Castes/Tribes**						
Scheduled Caste	2.5	7.7	36.7	18.4	44.9	3,456
Scheduled Tribes	3.3	3.9	43.2	16.9	39.8	819
Other Backward Classes	3.3	6.9	34.6	18.9	46.5	3,385
Others	3.5	5.4	35.5	21.6	42.9	4,686
**DLHS-4**	3.2	6.4	36.0	19.6	44.4	12,346
**DLHS-3**	4.3	16.5	22.5	9.2	68.4	7,487

Note: Spacing method includes IUD, pills, condom (Male/Female) and Injectables. Limiting method includes male sterilization and female sterilization.
[a] Literates but did not attend school, are also included. [1] Total figure may not add to 100 percent due to exclusion of other methods (Rhythm/ Periodic abstinence, Withdrawal, Undecided and Others). ( ) Based on 10-20 unweighted cases. -- Percentage not shown for less than 10 cases.
** Unweighted cases

TABLE 5.13 ADVICE ON CONTRACEPTIVE USE
Percentage of currently married women aged 15-49 years who are currently not using any contraceptive and were advised by the ANM/health worker to use modern contraception by suggested method and place of residence, Haryana, 20012-13.

Advice	Total	Residence	
		Rural	Urban
Percent Non-users advised to use modern contraceptive method[1]	37.5	38.2	36.2
**Number of Non-users****	**12,346**	**7,128**	**5,218**
Percent of Traditional method users advised to use modern method	24.9	32.9	14.8
**Number of traditional method users****	**469**	**237**	**232**
**Percent of non-users or traditional method users who were advised to use**			
Female sterilization	24.2	24.8	23.2
Male sterilization	7.8	8.7	6.4
IUD	11.5	12.0	10.6
Pill(Daily/weekly)	11.6	12.6	10.0
Injectables	4.2	5.0	3.1
Condom/*Nimrod*	16.1	15.5	17.1
Female condom	2.6	2.8	2.4
Rhythmic /periodic abstinence	1.9	2.2	1.4
Withdrawal	2.8	2.2	4.1
Others	4.8	2.1	4.1

Note: Exclude women in menopause or those who have undergone hysterectomy. [1] Includes Doctor, ANM, Health Worker, *anganwadi* Worker and ASHA. ** Unweighted cases.

TABLE 5.14 REASONS FOR NOT USING MODERN CONTRACEPTIVE METHODS AMONG RHYTHM AND WITHDRAWAL METHOD USERS

Percent distribution of currently married women aged 15-49 years who are currently using rhythm or withdrawal method by reasons for not using modern contraceptive method, according to selected background characteristics, Haryana, 2012-13.

	Reason for not using modern contraceptive method			
Background characteristics	Fertility related	Opposition to use/lack of knowledge	Method related	Number of women**
**Age group**				
15 - 19	(11.1)	(22.2)	(66.7)	12
20 - 24	57.2	27.4	15.4	81
25 - 29	43.8	23.8	32.4	131
30 - 34	45.6	23.7	30.7	91
35 - 39	52.9	16.4	30.7	72
40 - 44	50.1	8.6	41.2	57
45 - 49	42.9	19.7	37.4	25
**No. of living children**				
0	(70.0)	(10.0)	(20.0)	18
1	44.9	28.3	26.8	114
2	49.2	21.4	29.4	208
3	45.4	16.1	38.5	84
4+	45.2	16.2	38.6	45
**Residence**				
Rural	48.8	23.5	27.7	237
Urban	46.3	18.3	35.4	232
**Education**				
Non-literate[a]	53.6	12.7	33.7	114
Less than five years	47.3	29.3	23.4	73
5-9 years	43.3	25.8	30.9	71
10 or more years	46.1	22.0	31.9	211
**Religion**				
Hindu	46.8	20.6	32.6	428
Muslim	(83.3)	(8.3)	(8.3)	15
Christian	na	na	na	00
Sikh	41.3	40.6	18.1	26
Jain	na	na	na	00
Others	na	na	na	00
**Castes/Tribes**				
Scheduled Caste	50.5	17.0	32.5	117
Scheduled Tribes	(77.8)	(0.0)	(22.2)	16
Other Backward Classes	43.4	24.7	31.9	142
Others	47.6	22.6	29.8	194
**DLHS-4**	47.7	21.3	31.0	469
**DLHS-3**	5.9	36.2	57.9	1,416

[a] Literates but did not attend school, are also included. () based on 10-20 unweighted cases. na= Not applicable. ** Unweighted cases.

TABLE 5.15 UNMET NEED FOR FAMILY PLANNING SERVICES

Percentage of currently married women aged 15-49 years by unmet need for family planning services according to selected background characteristics, Haryana, 2012-13.

Background characteristics	Unmet need for FP			Number of women**
	Spacing[1]	Limiting[2]	Total	
**Age group**				
15 – 19	35.9	9.7	45.7	649
20 - 24	28.8	16.5	45.3	4,300
25 - 29	15.6	19.1	34.7	5,721
30 - 34	9.0	19.5	28.5	5,049
35 - 39	5.5	20.4	25.9	4,867
40 - 44	4.0	18.3	22.3	3,991
45 - 49	2.9	18.6	21.5	2,837
**Number of living children**				
0	33.7	8.6	42.2	2,825
1	23.5	16.7	40.1	5,058
2	7.3	20.4	27.8	10,250
3	5.0	19.1	24.2	5,940
4+	6.1	21.9	28.1	3,341
**Residence**				
Rural	12.2	18.2	30.4	16,093
Urban	11.1	19.4	30.5	11,321
**Education**				
Non-literate[a]	10.2	22.2	32.4	9,110
Less than five years	10.3	15.0	25.3	4,085
5-9 years	11.3	17.6	28.9	3,616
10 or more years	14.1	17.2	31.3	10,603
**Religion**				
Hindu	11.4	17.9	29.3	25,199
Muslim	22.3	34.1	56.5	1,221
Christian	6.7	32.9	39.6	32
Sikh	8.4	17.9	26.3	904
Jain	9.5	3.5	13.0	29
Others	--	--	--	09
**Castes/Tribes**	12.4	19.3	31.7	7,522
Scheduled Caste	12.1	30.7	42.8	1,433
Scheduled Tribes	11.6	18.2	29.8	7,785
Other Backward Classes	11.4	16.8	28.2	10,674
Others				
**DLHS-4**	11.8	18.6	30.4	27,414
**DLHS-3**	5.2	10.2	15.4	20,394

Note: Total unmet need refers to unmet for limiting and spacing.
[1] Unmet need for spacing includes the proportion of currently married women who are neither in menopause or had hysterectomy nor are currently pregnant and who want more children after two years nor later and are currently not using any family planning method. The women who are not sure about whether and when to have next child are also included in unmet need for spacing. [2] Unmet need for limiting includes the proportion of currently married women who are neither in menopause nor had hysterectomy nor are currently pregnant and do not want any more children but are currently not using any family planning method. Total unmet need refers to unmet for limiting and spacing. [a] Literates but did not attend school, are also included. -- Percentage not shown for less than 10 cases.** Unweighted cases.

TABLE 5.16 UNMET NEED FOR FAMILY PLANNING SERVICES BY DISTRICT

Percentage of currently married women aged 15-49 years by unmet need for family planning services by districts, Haryana, 2012-13.

District	Unmet need for FP			Number of women**
	Spacing[1]	Limiting[2]	Total	
Panchkula	7.7	8.9	16.6	945
Ambala	12.2	9.1	21.3	1,247
Yamunanagar	12.3	19.4	31.7	1,212
Kukshetra	14.4	32.7	47.1	1,586
Kaithal	9.0	14.4	23.4	1,488
Karnal	11.9	26.9	38.8	1,694
Panipat	11.5	23.6	35.1	1,300
Sonipat	19.6	12.3	31.9	1,458
Jind	10.0	17.6	27.6	1,416
Fatehabad	9.7	8.8	18.5	971
Sirsa	7.6	7.0	14.6	1,665
Hisar	10.3	6.8	17.2	1,539
Bhiwani	7.6	7.0	14.6	1,162
Rohtak	13.0	15.8	28.9	1,302
Jhajjar	10.5	20.0	30.5	1,467
Mahendragarh	8.3	7.6	15.9	1,162
Rewari	11.4	27.7	39.1	1,081
Gurgaon	12.0	22.4	34.4	1,274
Faridabad	12.1	29.4	41.5	1,150
Mewat	19.8	35.1	54.9	1,112
Palwal	15.4	42.7	58.1	1,183
**DLHS-4**	11.8	18.6	30.4	27,414
**DLHS-3**	5.2	10.2	15.4	20,394

Note: Total unmet need refers to unmet for limiting and spacing.
[1] Unmet need for spacing includes the proportion of currently married women who are neither in menopause nor had hysterectomy nor are currently pregnant and who want more children after two years or later and are currently not using any family planning method. The women who are not sure about whether and when to have next child are also included in unmet need for spacing. [2] Unmet need for limiting includes the proportion of currently married women who are neither in menopause nor had hysterectomy nor are currently pregnant and do not want any more children but are currently not using any family planning method. ** Unweighted cases.

REPRODUCTIVE HEALTH PROBLEMS AND AWARENESS

TABLE 6.1 MENSTRUATION RELATED PROBLEMS BY BACKGROUND CHARACTERISTICS

Percentage of ever married women aged 15-49 years who had any menstruation related problem during three months prior to survey and among them, reported specific symptoms according to selected background characteristics, Haryana, 2012-13.

Background characteristics	Who had any menstruation related problem (%)	Total number of women[1]	Reported Symptoms among who had any menstruation problem: No periods	Painful periods	Frequent or short periods	Irregular periods	Prolonged bleeding	Scanty bleeding	Inter-menstrual bleeding	Blood clots/excessive bleeding	Number of women who had menstruation problem**
**Age group**											
15-19	4.9	597	8.4	52.7	6.2	34.6	19.7	0.0	3.2	2.9	29
20-24	5.5	3,866	18.0	54.5	17.0	32.8	5.7	6.1	4.1	3.2	216
25-29	6.1	5,519	11.2	53.2	18.1	35.0	14.4	7.9	2.6	4.8	336
30-34	7.1	5,092	12.1	49.2	15.5	35.0	15.2	8.5	4.4	5.0	361
35-39	6.9	5,052	11.7	47.4	14.5	39.3	11.3	6.6	3.4	5.2	349
40-44	7.3	4,161	12.7	46.1	14.2	39.5	18.7	6.7	1.7	3.9	299
45-49	6.1	2,932	20.0	35.9	13.5	33.9	16.1	5.7	2.3	3.5	177
**Place of residence**											
Rural	6.6	16,037	15.0	51.8	16.5	36.9	14.1	6.0	2.3	3.6	1,066
Urban	6.3	11,182	10.4	42.2	13.4	35.0	13.4	8.7	4.6	5.8	701
**Age at consummation of marriage***											
Below18 years	7.3	7,798	12.1	48.7	15.0	36.4	14.1	6.7	2.8	3.8	577
18 years & above	6.1	18,240	13.7	49.0	15.4	36.1	13.7	7.3	3.3	4.7	1,110
**Marital duration***											
0-4	4.5	4,457	13.4	58.4	13.7	34.5	7.3	4.6	3.3	4.6	201
5-9	6.1	4,935	15.5	47.9	17.2	35.6	12.3	7.4	4.8	4.0	304
10-14	6.9	4,929	11.0	52.3	17.8	33.0	16.2	10.1	2.7	5.4	338
15+	7.2	11,847	13.5	46.0	13.8	37.9	14.8	6.3	2.7	4.1	853
**Education**											
Non-literate[a]	6.1	9,340	13.9	52.1	15.8	36.5	13.9	6.0	2.1	4.5	562
Less than 5 yrs	9.2	4,041	11.9	46.4	16.2	30.5	16.4	7.1	3.1	4.1	374
5-9 years	7.3	3,542	15.4	46.6	12.2	37.7	14.0	7.3	3.4	2.7	262
10 or more years	5.5	10,296	13.0	46.9	16.0	39.2	12.1	7.8	4.1	5.3	569
**Husband's education**											
Non-literate[a]	5.1	5,882	16.3	52.5	15.4	35.4	11.6	5.2	3.1	6.8	300
Less than 5 yrs	7.4	3,440	7.6	53.3	13.8	37.2	17.7	7.2	2.4	3.6	252
5-9 years	8.0	3,801	14.0	43.9	15.0	35.1	14.2	5.4	2.5	2.7	308
10 or more years	6.5	14,096	13.9	47.3	16.1	36.6	13.5	8.1	3.5	4.4	907
**Religion**											
Hindu	6.4	25,075	14.0	47.1	15.5	35.5	14.4	7.3	3.2	4.4	1,597
Muslim	8.2	1,166	9.0	67.7	17.1	42.1	6.5	5.9	2.3	2.3	95
Christian	6.8	31	--	--	--	--	--	--	--	--	02
Sikh	7.8	892	6.8	51.0	11.8	46.1	11.3	1.4	1.2	5.3	69
Jain	10.2	28	--	--	--	--	--	--	--	--	03
Other	6.1	27	--	--	--	--	--	--	--	--	01

Contd…

TABLE 6.1 MENSTRUATION RELATED PROBLEMSBY BACKGROUND CHARACTERISTICS – *Continued*

Background characteristics	Who had any menstruation related problem	Total number of women[1]	Reported Symptoms								Number of women who had menstruation problem**
			No periods	Painful periods	Frequent or short periods	Irregular periods	Prolonged bleeding	Scanty bleeding	Inter-menstrual bleeding	Blood clots/excessive bleeding	
**Castes/Tribes**											
Scheduled Caste	6.5	7,504	14.0	54.5	14.3	34.8	14.2	7.8	2.2	5.5	483
Scheduled Tribes	4.8	1,455	23.0	52.2	16.7	25.3	16.7	7.3	4.5	9.3	69
Other Backward Classes	7.4	7,660	13.6	44.5	16.5	37.3	13.4	5.3	4.3	3.9	572
Others	6.1	10,600	11.7	46.7	15.2	37.7	13.8	7.8	2.7	3.5	643
**DLHS-4**	6.5	27,219	13.4	48.5	15.4	36.2	13.9	7.0	3.1	4.4	1,767
**DLHS-3**	18.0	16,767	4.5	51.5	8.1	30.5	10.8	13.0	2.2	10.2	3,016

Note: Total figure may not add to 100 percent due to multiple responses.
[a] Literate but did not attend school, are also included. [1] Excludes pregnant, in amenorrhea, in menopause, had hysterectomy and ever menstruated women. -- Percentage not shown for less than 10 cases. ** Unweighted cases.

TABLE 6.2 SOURCE OF KNOWLEDGE ABOUT RTI/STIBY BACKGROUND CHARACTERISTICS

Percentage of ever married women aged 15- 49 years who have heard about RTI/STI, among them, who received information from specific sources according to selected background characteristics, Haryana, 2012-13

Background characteristics	Who have heard RTI/STI	Total number of women**	Source of Knowledge										Number of women heard of RTI/STI**
			Radio	T.V.	Cinema	Print media[1]	Health personnel[2]	School/ adult education programs[3]	Leaders/ community meeting[4]	Husband	Relative/ friends	Other	
**Age group**													
15-19	20.2	687	8.3	64.5	35.4	31.6	12.6	15.7	48.1	0.5	0.8	0.0	144
20-24	22.9	4,375	8.7	66.7	34.6	36.0	18.8	18.4	44.4	3.9	0.8	0.3	1,023
25-29	26.6	5,832	7.0	66.7	35.3	38.6	20.5	18.2	43.5	4.1	1.3	0.4	1,586
30-34	26.6	5,221	8.8	68.3	34.6	42.6	21.1	19.2	43.9	5.0	1.6	0.5	1,427
35-39	25.5	5,133	9.7	67.5	31.3	38.7	19.0	19.2	47.2	5.8	1.3	0.1	1,350
40-44	24.1	4,294	8.3	66.4	31.5	39.8	22.3	18.2	43.5	6.3	1.8	0.5	1,063
45-49	22.9	3,234	7.2	59.7	28.2	34.0	19.4	19.5	51.8	5.7	0.9	0.5	768
**Residence**													
Rural	19.9	16,895	6.9	57.6	27.2	32.4	19.7	16.0	42.9	4.3	1.1	0.3	3,363
Urban	33.6	11,881	9.8	75.3	39.0	45.0	20.4	21.4	47.8	5.6	1.5	0.5	3,998
**Age at consummation of marriage**													
Below 18 years	21.3	8,195	6.9	56.5	23.7	31.8	17.3	15.9	49.1	4.6	1.7	0.3	1,781
18 years & above	26.8	19,342	8.8	69.9	36.4	41.1	21.0	19.9	44.0	5.0	1.2	0.4	5,327
**Marital duration**													
0-4	27.4	5,101	8.1	71.3	40.2	39.9	19.4	20.8	45.7	3.4	1.1	0.5	1,439
5-9	24.4	5,197	8.1	69.4	35.0	40.2	21.0	18.3	40.8	4.8	1.1	0.3	1,299
10-14	25.5	5,034	8.9	66.7	34.3	42.3	20.2	17.8	44.9	4.5	1.3	0.2	1,319
15+	24.3	12,335	8.4	63.1	28.7	36.1	20.0	18.7	47.2	5.9	1.5	0.4	3,078
**Education**													
Non-literate[a]	13.9	9,810	8.0	46.2	16.6	25.8	15.1	15.1	51.2	5.6	1.1	0.5	1,383
Less than 5 yrs	20.8	4,296	6.7	54.3	17.8	30.2	17.5	15.5	49.5	3.4	0.8	0.2	911
5-9 years	24.4	3,734	12.5	64.3	28.8	38.2	21.3	17.5	43.6	4.9	1.0	0.4	930
10 or more years	37.2	10,936	7.9	76.8	43.4	45.2	22.2	21.0	42.7	5.1	1.6	0.4	4,137
**Husband's education**													
Non-literate[a]	13.4	6,157	9.5	53.4	21.2	25.9	15.9	13.5	44.8	6.4	1.4	0.5	836
Less than 5 years	18.8	3,679	8.4	50.3	16.7	29.2	16.8	13.6	53.5	5.4	0.6	0.4	715
5-9 years	21.4	4,026	10.7	60.7	25.7	34.1	21.4	18.3	48.2	5.3	1.0	0.4	876
10 or more years	32.3	14,914	7.7	72.1	38.9	43.1	21.1	20.4	43.7	4.6	1.5	0.4	4,934
**Religion**													
Hindu	25.3	26,476	8.2	67.3	33.5	39.3	20.0	18.1	45.0	5.0	1.3	0.4	6,882
Muslim	14.0	1,259	9.0	35.7	16.1	22.0	14.8	35.8	60.3	4.0	2.2	0.7	179
Christian	30.6	34	--	--	--	--	--	--	--	--	--	--	10
Sikh	28.1	947	10.6	62.6	32.9	30.1	26.4	20.4	43.6	2.7	0.7	0.0	272
Jain	38.2	31	(0.0)	(63.6)	(18.2)	(27.3)	(18.2)	(9.1)	(45.5)	(18.2)	(0.0)	(0.0)	11
Other	11.8	29	--	--	--	--	--	--	--	--	--	--	07

Contd…

TABLE 6.2 SOURCE OF KNOWLEDGE ABOUT RTI/STIBY BACKGROUND CHARACTERISTICS—*Continued*

Background characteristics	Who have heard RTI/STI	Total number of women**	Source of Knowledge										Number of women heard of RTI/STI**
			Radio	T.V.	Cinema	Print media[1]	Health personnel[2]	School/adult education programs[3]	Leaders/ community meeting[4]	Husband	Relative/ friends	Other	
**Castes/Tribes**													
Scheduled Caste	19.7	7,950	8.1	61.6	27.0	36.4	20.8	15.8	44.9	5.0	1.2	0.5	1,597
Scheduled Tribes	15.7	1,500	16.0	84.2	37.1	40.5	23.2	20.1	26.4	4.1	1.1	0.4	245
Other Backward Classes	25.7	8,124	7.3	59.8	28.7	32.7	18.4	17.9	51.3	3.6	1.3	0.2	2,146
Others	29.4	11,202	8.6	71.7	38.6	43.3	20.6	20.5	43.1	5.9	1.4	0.4	3,373
**DLHS-4**	24.9	28,776	8.3	66.4	33.1	38.6	20.1	18.7	45.3	4.9	1.3	0.4	7,361
**DLHS-3**	39.0	21,484	6.9	50.6	1.6	21.0	11.6	0.5	1.0	3.1	53.9	2.3	7,513

Note: Total figure may not add to 100 percent due to multiple responses.
[a] Literate but did not attend school, are also included. [1] Includes News papers/books/magazines/slogans/pamphlets and posters. [2] Includes Doctor/ASHA/health workers. [3] Includes school/teacher, adult education programs.[4] Includes religious/ political leaders, community meetings and exhibition/ *Mela*. ( ) Based on 10-20 unweighted cases. -- Percentage not shown for less than 10 cases.** Unweighted cases

TABLE 6.3 KNOWLEDGE OF MODE OF TRANSMISSION OF RTI/STIBY BACKGROUND CHARACTERISTICS
Percentage of ever married women aged 15-49 years who have heard of RTI/STI and among them, who have knowledge of transmission of RTI/STI, according to selected background characteristics, Haryana, 2012-13.

		knowledge of transmission of RTI/STI							
Background characteristics	Heard of RTI/STI	Unsafe delivery	Unsafe abortion	Unsafe IUD insertion	Unsafe sex with homose xuals	Unsafe sex with persons who have many partners	Unsafe sex with sex workers	Other	Number of women heard of RTI/STI**
**Age group**									
15-19	20.2	42.5	42.1	35.2	22.2	27.5	13.6	6.3	144
20-24	22.9	42.7	42.1	33.0	25.6	30.6	19.7	3.4	1,023
25-29	26.6	44.3	41.6	34.0	25.9	29.2	19.8	5.6	1,586
30-34	26.6	45.9	42.1	35.3	29.3	31.3	23.2	6.7	1,427
35-39	25.5	43.0	39.7	31.3	27.6	30.4	23.0	5.7	1,350
40-44	24.1	40.3	37.2	31.9	29.4	31.9	25.7	5.8	1,063
45-49	22.9	38.2	33.8	26.8	23.2	26.8	19.9	5.2	768
**Residence**									
Rural	19.9	40.9	38.1	30.4	21.7	25.5	17.9	1.7	3,363
Urban	33.6	45.0	42.0	34.9	32.4	34.8	25.7	9.5	3,998
**Age at consummation of marriage**									
Below 18 years	21.3	36.5	33.5	27.6	22.4	25.4	16.3	4.6	1,781
18 years & above	26.8	45.1	42.3	34.4	28.7	31.9	23.7	5.8	5,327
**Marital duration**									
0-4	27.4	44.4	44.4	36.7	28.3	33.2	21.6	6.0	1,439
5-9	24.4	48.2	44.1	33.9	26.6	29.2	20.4	5.0	1,299
10-14	25.5	43.9	40.4	34.6	28.5	31.1	23.7	6.0	1,319
15+	24.3	39.7	36.4	29.6	26.4	29.0	21.6	5.1	3,078
**Education**									
Non-literate[a]	13.9	31.5	28.2	25.6	18.0	19.6	12.5	2.6	1,383
Less than 5 yrs	20.8	34.8	33.9	24.4	20.9	20.2	15.6	4.1	911
5-9 years	24.4	40.0	37.7	31.5	24.8	25.9	19.0	2.3	930
10 or more years	37.2	49.5	46.1	37.3	32.1	37.1	27.1	7.6	4,137
**Husband's education**									
Non-literate[a]	13.4	35.8	35.5	30.8	21.4	22.0	15.9	1.8	836
Less than 5 years	18.8	33.2	30.4	26.0	19.9	18.9	13.0	3.6	715
5-9 years	21.4	38.8	36.1	27.9	22.4	22.1	17.2	3.7	876
10 or more years	32.3	46.4	42.9	34.8	29.8	34.7	25.0	6.8	4,934
**Religion**									
Hindu	25.3	43.1	40.2	32.7	27.4	30.6	22.2	5.7	6,882
Muslim	14.0	31.3	26.3	25.4	13.2	18.2	8.6	6.8	179
Christian	--	--	--	--	--	--	--	--	10
Sikh	28.1	45.6	44.3	34.8	24.3	26.4	20.4	1.0	272
Jain	(35.5)	(27.3)	(27.3)	(9.1)	(18.2)	(9.1)	(0.0)	(0.0)	11
Other	--	--	--	--	--	--	--	--	07
**Castes/Tribes**									
Scheduled Caste	19.7	39.7	38.8	31.1	26.4	27.5	20.8	3.0	1,597
Scheduled Tribes	15.7	50.9	54.3	36.1	38.8	28.3	23.4	1.7	245
Other Backward Classes	25.7	39.9	35.3	28.0	20.7	24.0	15.4	5.5	2,146
Others	29.4	45.9	42.6	36.1	30.4	35.5	26.2	7.0	3,373
**DLHS-4**	24.9	42.9	40.0	32.6	27.0	30.1	21.8	5.5	7,361
**DLHS-3**	39.0	8.1	5.7	4.6	10.1	70.8	9.7	18.5	8,411

Note: Total figure may not add to 100 percent due to multiple responses.
[a] Literate but did not attend school, are also included. ( ) Based on 10-20 unweighted cases. -- Percentage not shown for less than 10 cases.
** Unweighted cases.

TABLE 6.4 SYMPTOMS OF RTI/STIBY BACKGROUND CHARACTERISTICS

Percentage of ever married women aged 15-49 years who had reported abnormal vaginal discharge, other RTI/STI symptoms during three months prior to survey according to selected background characteristics, Haryana, 2012-13

Background characteristics	Women reported abnormal vaginal discharge	Women reported other RTI/STI symptoms[1]	Percentage reported specific symptom of RTI/STI[1]: Itching or irritation over vulva	Boils/Ulcers/ Warts around vulva	Pain in lower abdomen not related menses	Swelling in the groin	Painful blister like lesions	Pain during sexual intercourse[2]	Spotting after sexual intercourse[2]	Total number of women **
**Age group**										
15-19	4.4	13.7	4.1	0.9	4.5	0.2	0.4	4.3	0.8	687
20-24	4.2	12.1	3.0	0.5	2.7	0.3	0.3	2.9	1.0	4,375
25-29	6.1	12.2	3.2	0.6	3.1	0.4	0.4	2.3	0.5	5,832
30-34	7.0	13.0	3.4	0.4	3.9	0.4	0.3	1.9	0.5	5,221
35-39	6.1	12.6	3.6	0.8	3.7	0.5	0.3	1.7	0.5	5,133
40-44	5.4	12.6	3.1	0.7	3.3	0.4	0.3	0.9	0.5	4,294
45-49	4.3	11.0	2.8	0.8	2.1	0.2	0.4	0.8	0.4	3,234
**Residence**										
Rural	5.5	13.2	3.3	0.6	3.4	0.4	0.3	2.1	0.6	16,895
Urban	5.9	10.9	3.0	0.7	3.0	0.4	0.3	1.5	0.5	11,881
**Age at consummation of marriage**										
Below 18 years	6.9	13.7	3.5	0.6	3.6	0.4	0.3	2.0	0.5	8,195
18 years & above	5.1	11.8	3.1	0.7	3.2	0.4	0.4	1.9	0.6	19,342
**Marital duration**										
0-4	3.7	11.1	2.5	0.4	2.9	0.3	0.3	2.9	0.8	5,101
5-9	5.4	12.0	3.6	0.7	2.8	0.3	0.4	2.1	0.6	5,197
10-14	7.1	13.1	3.3	0.6	4.3	0.4	0.3	2.2	0.6	5,034
15+	6.0	13.0	3.4	0.8	3.3	0.4	0.3	1.4	0.5	12,335
**Education**										
Non-literate[a]	5.5	11.5	3.0	0.6	3.0	0.4	0.3	1.5	0.5	9,810
Less than 5 yrs	7.6	14.3	3.4	0.9	4.2	0.5	0.3	1.8	0.7	4,296
5-9 years	6.3	14.0	3.8	0.7	3.6	0.3	0.2	2.0	0.6	3,734
10 or more years	4.6	11.8	3.1	0.6	3.0	0.4	0.4	2.2	0.6	10,936
**Husband's education**										
Non-literate[a]	4.2	9.1	2.3	0.4	2.4	0.3	0.2	1.3	0.5	6,157
Less than 5 years	7.6	14.5	3.5	0.9	3.9	0.3	0.2	2.2	0.7	3,679
5-9 years	7.1	14.5	4.0	0.9	4.4	0.5	0.3	1.8	0.6	4,026
10 or more years	5.3	12.6	3.3	0.6	3.2	0.4	0.4	2.0	0.6	14,914
**Religion**										
Hindu	5.6	12.4	3.2	0.7	3.3	0.4	0.3	1.9	0.6	26,476
Muslim	7.1	12.0	3.7	0.4	2.7	0.6	0.4	2.7	0.3	1,259
Christian	6.3	2.5	0.0	0.0	0.0	0.0	0.0	0.0	0.0	34
Sikh	4.1	12.5	2.4	0.6	4.1	0.1	0.2	1.5	0.7	947
Jain	9.9	6.4	3.0	0.0	3.0	0.0	0.0	3.4	0.0	31
Other	--	--	--	--	--	--	--	--	--	29

Contd…

TABLE 6.4 SYMPTOMS OF RTI/STIBY BACKGROUND CHARACTERISTICS

Background characteristics	Women reported abnormal vaginal discharge	Women reported other RTI/STI symptoms[1]	Percentage reported specific symptom of RTI/STI[1]							Total number of women **
			Itching or irritation over vulva	Boils/Ulcers/ Warts around vulva	Pain in lower abdomen not related menses	Swelling in the groin	Painful blister like lesions	Pain during sexual intercourse[2]	Spotting after sexual intercourse[2]	
**Castes/Tribes**										
Scheduled Caste	5.6	12.6	3.3	0.6	3.5	0.4	0.3	2.0	0.6	7,950
Scheduled Tribes	3.1	9.8	3.3	0.9	1.9	0.3	0.1	1.4	0.4	1,500
Other Backward Classes	7.1	13.2	3.5	0.6	3.7	0.4	0.4	1.9	0.7	8,124
Others	5.0	11.9	2.9	0.7	2.9	0.3	0.3	1.8	0.5	11,202
**DLHS-4**	5.6	12.4	3.2	0.7	3.3	0.4	0.3	1.9	0.6	28,776
**DLHS-3**	14.3	16.3	3.5	0.8	3.3	0.6	0.2	2.5	0.2	21,484

Note: Total figure may not add to 100 percent due to 'do not know' or 'missing cases.
[a] Literate but did not attend school, are also included. [1] Excluding women having abnormal vaginal discharge problem. [2] Only for currently married women. -- Percentage not shown for less than 10 cases. ** Unweighted cases.

TABLE 6.5 DISCUSSED ABOUT RTI/STI PROBLEMS WITH HUSBAND AND SOUGHT TREATMENTBY BACKGROUND CHARACTERISTICS

Percentage of ever married women aged 15-49 years discussed RTI /STI problem with husband/partner and sought treatment among who reported any RTI/STI[1] problem and source of treatment according to selected background characteristics, Haryana, 2012-13.

Background characteristics	Women discussed RTI/STI problems with husband/ partner[1]	Women sought treatment[1] for RTI/STI problems	Number of women having any RTI/STI [1]	Source of treatment: Government	Source of treatment: Private	Source of treatment: Other	Number of women who sought treatment**
**Age group**							
15-19	56.5	26.9	93	16.9	83.1	0.0	24
20-24	52.5	31.1	527	26.6	71.7	1.7	169
25-29	53.0	30.8	718	26.7	70.4	2.9	226
30-34	61.6	39.8	676	26.6	71.9	1.5	271
35-39	57.8	38.2	646	24.6	73.4	2.0	248
40-44	53.2	34.6	536	29.0	69.0	2.0	186
45-49	51.4	34.1	358	24.1	73.9	1.9	124
**Residence**							
Rural	53.8	33.1	2,227	29.0	68.8	2.2	739
Urban	58.8	38.1	1,327	21.0	77.5	1.5	509
**Age at consummation of marriage**							
Below 18 years	58.5	36.1	1,120	26.9	71.7	1.4	408
18 years & above	56.0	34.3	2,287	25.3	72.4	2.2	797
**Marital duration**							
0-4	57.6	32.9	558	26.1	72.4	1.5	190
5-9	49.3	29.7	637	25.9	71.2	3.0	193
10-14	59.6	37.1	653	28.7	69.3	2.0	242
15+	58.0	37.1	1,594	25.4	72.9	1.7	596
**Education**							
Non-literate[a]	53.0	33.9	1,130	26.1	72.5	1.4	384
Less than 5 yrs	59.2	36.2	80	29.5	68.2	2.3	227
5-9 years	55.4	35.4	1,064	29.1	68.8	2.1	190
10 or more years	55.7	34.4	1,280	23.1	74.7	2.2	447
**Husband's education**							
Non-literate[a]	49.5	34.4	570	30.9	66.9	2.3	194
Less than 5 years	58.3	32.8	82	24.5	74.4	1.1	181
5-9 years	59.0	36.2	1,039	29.1	69.4	1.4	215
10 or more years	55.2	34.9	1,863	24.2	73.5	2.3	658
**Religion**							
Hindu	54.2	33.7	3,272	26.5	71.3	2.2	1,118
Muslim	69.8	36.1	154	28.2	71.8	0.0	54
Christian	--	--	01	na	na	na	00
Sikh	66.6	60.8	121	20.8	79.2	0.0	73
Jain	--	--	02	na	na	na	00
Other	--	--	04	--	--	--	01
**Castes/Tribes**							
Scheduled Caste	52.4	32.3	1,006	29.5	69.2	1.3	325
Scheduled Tribes	45.8	33.2	152	35.3	64.7	0.0	51
Other Backward Classes	59.8	37.0	1,075	25.4	72.6	2.0	406
Others	55.1	34.9	1,321	23.4	74.0	2.6	466
**DLHS-4**	55.4	34.7	3,554	26.1	71.9	2.0	1,248
**DLHS-3**	80.8	52.6	4,770	19.1	71.2	9.7	2,510

Note: Total figure may not add to 100 percent due to 'do not know' or 'missing cases'.
[a] Literate but did not attend school, are also included. [1] Any RTI/STI (Including abnormal vaginal discharge or other RTI/STI problem). na: not applicable. -- Percentage not shown for less than 10 cases. ** Unweighted cases.

TABLE 6.6 RTI/STI INDICATORS BY DISTRICTS

Percentage of ever married women aged 15-49 years who reported RTI/STI problem during three months prior to the survey and among them percentage sought treatment for the problem by district, Haryana, 2012-13.

District	Who heard about RTI/STI	Who reported any abnormal vaginal discharge	Who have any other symptoms of RTI/STI[1]	Total number of women**	Who sought treatment for any RTI/STI[2]	Number of women having any RTI/STI[2]
Panchkula	55.5	3.7	7.3	986	61.6	73
Ambala	23.9	1.8	4.4	1,303	33.9	57
Yamunanagar	11.1	4.2	11.5	1,271	51.8	145
Kurukshetra	17.0	5.2	6.1	1,659	38.2	105
Kaithal	42.5	5.4	9.3	1,558	48.3	148
Karnal	8.6	5.8	11.6	1,800	30.7	209
Panipath	6.9	4.8	21.1	1,372	33.7	293
Sonipath	8.7	4.2	18.0	1,552	27.9	277
Jind	32.7	4.3	4.0	1,484	40.7	64
Fatehabad	12.9	5.2	12.5	1,027	57.7	136
Sirsa	30.1	6.6	17.8	1,717	57.9	307
Hisar	39.0	7.5	9.6	1,652	45.4	162
Bhiwani	34.3	9.6	9.6	1,230	44.6	121
Rohtak	21.4	5.5	24.9	1,365	23.1	345
Jhajjar	45.3	6.3	28.8	1,521	19.2	436
Mahendragarh	21.8	11.3	5.3	1,267	54.7	68
Rewari	26.1	6.2	12.3	1,118	26.6	137
Gurgaon	36.7	5.5	14.2	1,321	20.0	183
Faridabad	33.3	3.8	9.8	1,188	19.3	117
Mewat	16.8	6.9	8.0	1,156	25.2	96
Palwat	16.0	4.3	6.0	1,229	30.1	75
**DLHS-4**	25.5	5.6	12.2	28,776	34.9	3,554
**DLHS-3**	39.0	14.3	16.3	21,484	52.6	4,770

[1] Excluding women having abnormal vaginal discharge. [2] Any RTI/STI (Including abnormal vaginal discharge problem or other RTI/STI problem).
** Unweighted cases.

TABLE 6.7 KNOWLEDGE OF HIV/AIDS

Percentage of ever married women aged 15–49 years who have heard of HIV/AIDS and among them, who received information from specific sources according to selected background characteristics, Haryana, 2012-13.

Background characteristics	Who have heard of HIV/AIDS	Total women**	Sources of knowledge for HIV/AIDS: Radio	T.V.	Cinema	Print media[1]	Health personnel[2]	School/adult education programs[3]	Leaders/ community meetings[4]	Husband	Relatives/ friends	Other	Number of women heard of HIV/AIDS**
**Age group**													
15-19	50.2	687	10.1	88.4	47.7	35.0	16.8	27.5	44.8	5.2	0.3	2.6	346
20-24	55.7	4,375	7.6	89.1	46.8	34.9	21.1	30.6	43.5	4.7	0.4	2.6	2,455
25-29	57.0	5,832	8.0	92.3	44.2	37.5	22.4	31.6	43.6	5.9	0.9	1.6	3,361
30-34	56.7	5,221	10.2	90.4	43.4	36.8	22.8	32.7	43.1	5.7	1.0	1.6	2,997
35-39	50.7	5,133	10.0	89.6	41.8	36.7	21.4	31.4	40.7	6.6	0.7	1.2	2,651
40-44	46.9	4,294	9.1	90.1	39.3	36.1	22.4	30.2	40.9	7.1	1.3	1.1	2,064
45-49	40.2	3,234	9.6	89.9	39.5	35.0	21.6	30.0	43.5	6.9	0.8	1.3	1,345
**Residence**													
Rural	46.0	16,895	8.0	87.8	40.5	31.4	22.1	30.5	39.0	5.5	0.8	2.4	7,763
Urban	62.6	11,881	10.2	93.0	45.7	41.4	21.7	31.9	46.5	6.5	0.9	0.8	7,456
**Age at consummation of marriage**													
Below 18 years	44.5	8,195	8.0	87.7	35.7	31.0	21.3	31.4	41.8	5.7	0.8	1.9	3,688
18 years & above	56.0	19,342	9.5	91.5	45.7	38.2	22.2	31.8	43.2	6.1	0.8	1.5	10,997
**Marital duration**													
0-4	61.2	5,101	8.6	90.9	50.2	38.7	21.7	31.4	46.9	5.1	0.7	2.2	3,153
5-9	54.6	5,197	8.7	92.1	44.3	37.6	22.4	31.4	41.4	5.7	0.9	1.6	2,874
10-14	55.9	5,034	9.1	91.1	42.5	35.7	22.2	32.4	42.6	6.1	0.8	1.8	2,842
15+	46.6	12,335	9.6	89.4	39.2	35.0	21.9	31.6	41.4	6.5	0.8	1.2	5,870
**Education**													
Non-literate[a]	24.3	9,810	9.8	81.7	26.1	27.8	22.6	26.9	36.1	5.4	0.5	1.2	2,407
Less than 5 yrs	47.1	4,296	7.0	87.2	28.8	27.6	20.0	30.3	38.9	5.7	0.2	1.5	2,035
5-9 years	60.2	3,734	10.4	90.6	39.7	33.7	21.9	30.7	39.2	5.2	0.7	1.4	2,252
10 or more years	77.7	10,936	9.0	93.4	52.1	41.5	22.1	32.7	46.3	6.5	1.1	1.8	8,525
**Husband's education**													
Non-literate[a]	24.5	6,157	11.3	81.2	32.8	30.0	25.8	22.8	33.1	5.3	0.7	1.3	1,525
Less than 5 years	39.9	3,679	8.6	88.6	30.0	26.7	20.3	28.0	40.3	5.8	0.6	1.5	1,488
5-9 years	47.9	4,026	10.8	89.1	36.2	32.3	22.2	31.8	39.2	5.7	0 .4	1.4	1,951
10 or more years	68.2	14,914	8.5	92.2	47.8	39.4	21.5	32.8	45.0	6.2	1.0	1.7	10,255
**Religion**													
Hindu	53.2	26,476	9.1	90.4	43.1	36.5	21.8	31.0	43.0	6.0	0.8	1.7	14,297
Muslim	21.5	1,259	9.6	82.6	32.7	31.3	23.5	36.7	40.2	8.9	1.1	0.7	281
Christian	(52.9)	34	(0.0)	(100.0)	(61.1)	(50.0)	(11.1)	(33.3)	(50.0)	16.7	(0.0)	(0.0)	18
Sikh	61.0	947	7.9	92.5	45.2	34.6	24.3	32.4	35.3	4.6	0.7	0.7	584
Jain	88.0	31	7.4	92.6	44.4	29.6	22.2	25.9	22.2	18.5	0.0	3.7	27
Other	--	29	--	--	--	--	--	--	--	--	--	--	03

Contd….

TABLE 6.7 KNOWLEDGE OF HIV/AIDS

Background characteristics	Who have heard of HIV/AIDS AIDS	Total women**	Sources of knowledge for HIV/AIDS: Radio	T.V.	Cinema	Print media[1]	Health personnel[2]	School/ adult education programs[3]	Leaders/ community meetings[4]	Husband	Relatives/ Friends	Other	Number of women heard of HIV/AIDS**
**Castes/Tribes**													
Scheduled Caste	43.0	7,950	9.2	88.1	36.0	34.7	23.7	30.0	40.6	5.0	0.6	1.2	3,454
Scheduled Tribes	42.2	1,500	16.5	90.0	45.2	39.6	23.1	37.4	37.9	5.0	0.3	0.6	641
Other Backward Classes	50.7	8,124	8.3	90.1	41.1	33.5	20.8	30.6	41.6	6.2	0.7	2.1	4,169
Others	61.2	11,202	8.8	91.6	47.5	38.5	21.6	31.6	44.7	6.5	1.0	1.6	6,955
**DLHS-4**	52.1	28,776	9.1	90.3	43.0	36.3	21.9	31.2	42.6	6.0	0.8	1.6	15,219
**DLHS-3**	64.4	21,484	14.8	81.5	2.5	29.8	11.1	1.2	1.4	5.4	37.1	2.7	13,895

Note: Total figure may not add to 100 percent due to multiple responses
[a] Literate but did not attend school, are also included. [1] Includes News papers/books/magazines//slogans/pamphlets and posters. [2] Includes Doctor/ASHA/health workers. [3] Includes school/teacher, adult education programs. [4] Includes religious/ political leaders, community meetings and exhibition/ *Mela*. ( ) Based on 10-20 unweighted cases. -- Percentage not shown for less than 10 cases. ** Unweighted cases.

TABLE 6.8 KNOWLEDGE ABOUT MODE OF TRANSMISSION OF HIV/AIDSBY BACKGROUND CHARACTERISTICS

Percentage of ever married women aged 15-49 years having knowledge of mode of transmission of HIV/AIDS among who have heard about HIV/AIDS according to selected background characteristics, Haryana, 2012-13.

	Percentage of women who reported mode of transmission as							
Background characteristics	Unsafe sex with homo-sexuals	Unsafe sex with person having many partners	Unsafe sex with sex workers	Unprotected sex with HIV/AIDS infected person	Infected mother to child	Transfusion of infected blood	Sharing of injection/ Needles	Number of women who heard of HIV/AIDS**
**Age group**								
15-19	33.1	50.3	31.5	31.1	35.4	49.1	41.5	346
20-24	34.8	54.7	36.8	31.8	40.3	51.5	44.4	2,455
25-29	36.1	55.0	37.6	32.3	40.7	53.5	45.8	3,361
30-34	38.8	54.0	37.2	32.3	40.3	52.6	45.0	2,997
35-39	36.7	53.5	37.0	30.7	39.7	51.6	42.9	2,651
40-44	37.5	51.4	37.8	29.8	38.5	46.8	39.4	2,064
45-49	38.6	50.4	37.0	29.7	36.8	45.3	37.8	1,345
**Residence**								
Rural	31.8	52.7	36.2	28.6	37.5	48.6	41.3	7,763
Urban	43.3	54.5	38.2	34.9	42.3	53.9	45.8	7,456
**Age at consummation of marriage**								
Below 18 years	34.9	51.2	34.8	28.5	33.5	44.0	37.7	3,688
18 years & above	37.8	54.4	38.0	32.5	41.9	53.5	45.5	10,997
**Marital duration**								
0-4	38.1	55.4	38.3	34.4	43.8	56.0	47.0	3,153
5-9	35.8	55.0	37.7	32.4	40.3	52.5	46.0	2,874
10-14	35.4	54.5	35.6	30.9	39.6	51.9	44.3	2,842
15+	37.9	51.4	37.2	29.7	37.3	47.2	39.8	5,870
**Education**								
Non-literate[a]	33.6	45.7	34.5	22.8	29.1	35.7	28.5	2,407
Less than 5 yrs	30.6	50.6	31.6	23.6	31.5	43.1	36.1	2,035
5-9 years	33.0	53.8	34.3	27.4	36.1	47.7	41.2	2,252
10 or more years	40.5	56.4	40.0	36.9	45.7	58.3	50.0	8,525
**Husband's Education**								
Non-literate[a]	35.2	46.7	39.2	25.3	33.6	37.1	31.1	1,525
Less than 5 years	31.5	50.5	31.7	22.9	31.5	42.3	36.2	1,488
5-9 years	34.8	51.9	33.0	25.2	34.6	46.5	38.3	1,951
10 or more years	38.3	55.3	38.4	34.7	42.7	55.2	47.2	10,255
**Religion**								
Hindu	36.9	53.4	37.3	31.7	39.4	50.7	43.3	14,297
Muslim	32.4	49.7	29.3	25.8	40.3	52.2	37.6	281
Christian	(55.6)	(61.1)	(22.2)	(27.8)	(44.4)	(55.6)	(44.4)	18
Sikh	37.5	57.9	35.8	24.8	43.2	55.1	46.1	584
Jain	56.8	29.2	43.3	22.8	41.6	54.7	39.3	27
Other	--	--	--	--	--	--	--	03
**Castes/Tribes**								
Scheduled Caste	34.7	50.1	37.8	28.3	36.7	46.0	38.2	3,454
Scheduled Tribes	41.6	58.4	40.8	30.0	38.5	48.5	39.2	641
Other Backward Classes	36.9	52.0	33.9	28.1	37.8	50.0	42.0	4,169
Others	37.5	55.7	38.3	35.0	42.3	54.3	47.0	6,955
**DLHS-4**	36.9	53.5	37.1	31.3	39.6	50.9	43.3	15,219
**DLHS-3**	3.3	80.7	11.1	25.8	13.5	41.2	37.1	13,895

Note: Total figure may not add to 100 percent due to multiple responses.
[a] Literate but did not attend school, are also included. ( ) Based on 10-20 unweighted cases. -- Percentage not shown for less than 10 cases.
** Unweighted cases.

TABLE 6.9 KNOWLEDGE OF HIV PREVENTION METHODSBY BACKGROUND CHARACTERISTICS

Percentage of ever married women aged 15-49 years who heard about HIV/AIDS, percentage who reported HIV/AIDS can be prevented in specific ways, according to selected background characteristics, Haryana, 2012-13.

Background characteristics	Percentage who say that HIV/AIDS can be prevented by: Using condom correctly during each sexual intercourse	Sex with one partner	Avoid homosexual[1]	Avoid risks getting infected through bloods[2]	Avoid Pregnancy when having HIV/AIDS	Number of women having knowledge of HIV/AIDS**
**Age group**						
15-19	27.3	31.5	14.9	61.4	8.4	346
20-24	30.8	33.8	14.9	64.2	9.9	2,455
25-29	32.9	33.8	14.7	63.8	9.3	3,361
30-34	34.0	34.4	15.6	65.1	8.5	2,997
35-39	33.0	35.1	16.3	63.4	7.7	2,651
40-44	32.2	32.2	17.1	60.2	7.5	2,064
45-49	29.0	30.3	16.5	58.4	8.0	1,345
**Residence**						
Rural	29.5	33.1	13.9	60.1	7.6	7,763
Urban	35.7	34.1	17.9	66.8	9.9	7,456
**Age at consummation of marriage**						
Below 18 years	28.4	31.2	13.9	55.9	7.2	3,688
18 years & above	33.8	34.6	16.4	65.8	9.1	10,997
**Marital duration**						
0-4	33.4	34.6	16.6	67.2	10.8	3,153
5-9	32.4	34.1	15.2	64.7	9.7	2,874
10-14	33.3	35.3	14.4	63.9	7.3	2,842
15+	31.4	32.3	16.2	60.0	7.5	5,870
**Education**						
Non-literate[a]	25.6	28.3	11.8	48.9	6.0	2,407
Less than 5 yrs	24.1	28.5	13.3	47.2	6.3	2,035
5-9 years	30.8	33.7	15.1	41.5	7.1	2,252
10 or more years	36.6	36.4	17.6	35.9	10.3	8,525
**Husband's education**						
Non-literate[a]	25.9	29.5	13.9	46.2	5.0	1,525
Less than 5 years	25.8	29.1	12.9	46.5	5.5	1,488
5-9 years	30.9	32.1	15.1	42.2	8.9	1,951
10 or more years	34.4	35.1	16.5	38.3	9.6	10,255
**Religion**						
Hindu	32.3	33.6	15.9	40.3	8.5	14,297
Muslim	25.6	26.8	9.7	49.2	8.0	281
Christian	(77.8)	(50.0)	(5.6)	(55.6)	(7.6)	18
Sikh	30.7	34.2	14.4	40.3	10.3	584
Jain	37.0	37.5	8.2	29.4	11.2	27
Other	--	--	--	--	--	03
**Castes/Tribes**						
Scheduled Caste	30.6	32.3	14.7	41.0	7.8	3,454
Scheduled Tribes	38.9	42.6	20.8	36.6	5.6	641
Other Backward Classes	29.7	29.9	15.5	44.3	8.4	4,169
Others	34.0	35.6	15.7	38.1	9.4	6,955
**DLHS-4**	32.2	33.6	15.7	40.4	8.6	15,219
**DLHS-3**	7.6	34.2	71.4	44.4	5.3	13,895

Note: Total figure may not add to 100 percent due to multiple responses.
[a] Literate but did not attend school, are also included. [1] Includes sex with one partner, Limit number of sexual partner, Avoid sex with sex workers and avoids sex with homosexuals.[2] Includes avoid sex with who inject drugs, use tested blood, use only new/ sterilized needles, avoid IV drip and avoid razors/blades. ( ) Based on 10-20 unweighted cases. -- Percentage not shown for less than 10 cases. ** Unweighted cases.

TABLE 6.10 MISCONCEPTION ABOUT TRANSMISSION OF HIV/AIDSBY BACKGROUND CHARACTERISTICS

Percentage of ever married women aged 15-49 years having misconception about the transmission of HIV/AIDS among who have heard of HIV/AIDS, according to selected background characteristics, Haryana, 2012-13.

	Misconception about the transmission of HIV/AIDS						Number of women heard of HIV/AIDS**
Background characteristics	Shaking hand	Hugging	Sharing clothes	Sharing food	Stepping on someone's urine/stool	Get HIV/AIDS from mosquito, flea or bedbug	
**Age group**							
15-19	3.2	0.8	3.0	3.5	5.6	9.6	346
20-24	2.6	2.5	3.3	4.1	4.4	9.4	2,455
25-29	2.0	1.9	2.6	3.3	4.8	8.2	3,361
30-34	1.7	1.7	2.6	3.0	4.0	7.3	2,997
35-39	2.7	2.7	2.9	3.4	5.1	9.4	2,651
40-44	1.9	2.4	3.6	3.8	5.2	8.8	2,064
45-49	2.7	2.9	4.1	4.2	4.9	9.9	1,345
**Residence**							
Rural	2.5	2.5	3.6	4.1	5.6	10.2	7,763
Urban	1.9	1.9	2.4	2.7	3.5	6.6	7,456
**Age at consummation of marriage**							
Below 18 years	2.7	3.0	4.0	4.9	6.4	11.4	3,688
18 years & above	2.1	2.0	2.6	3.1	4.1	7.6	10,997
**Marital duration**							
0-4	2.2	1.9	2.4	3.3	4.0	8.2	3,153
5-9	1.8	1.6	2.5	2.9	3.6	7.1	2,874
10-14	2.2	2.5	3.0	3.7	5.1	8.1	2,842
15+	2.5	2.6	3.5	3.8	5.3	9.8	5,870
**Education**							
Non-literate[a]	3.1	3.5	5.0	5.2	7.2	10.6	2,407
Less than 5 yrs	3.3	4.2	5.6	6.5	8.3	12.8	2,035
5-9 years	2.2	2.2	3.1	3.8	4.6	9.7	2,252
10 or more years	1.7	1.4	1.8	2.2	3.1	6.8	8,525
**Husband's education**							
Non-literate[a]	2.5	2.8	3.6	3.8	5.2	6.9	1,525
Less than 5 years	3.0	3.9	5.7	5.9	7.1	11.6	1,488
5-9 years	2.9	2.9	4.4	5.4	6.1	11.3	1,951
10 or more years	1.9	1.8	2.3	2.8	4.0	8.0	10,255
**Religion**							
Hindu	2.2	2.1	2.9	3.5	4.7	8.8	14,297
Muslim	3.7	5.6	7.2	6.3	9.3	11.8	281
Christian	(5.6)	(5.6)	(5.6)	(5.6)	(11.1)	(16.7)	18
Sikh	2.5	2.6	3.5	3.6	3.0	5.0	584
Jain			3.4	7.9	3.4	7.9	27
Other	--	--	--	--	--	--	03
**Castes/Tribes**							
Scheduled Caste	2.7	2.9	3.6	4.1	5.0	8.9	3,454
Scheduled Tribes	.9	1.2	1.8	2.4	2.3	3.4	641
Other Backward Classes	2.8	2.5	4.0	4.7	6.3	11.1	4,169
Others	1.8	1.8	2.3	2.6	3.8	7.5	6,955
**DLHS-4**	2.2	2.2	3.0	3.5	4.7	8.7	15,219
**DLHS-3**	8.9	9.0	11.3	12.3	10.0	17.3	13,895

Note: Total figure may not add to 100 percent due to multiple responses and missing cases.
[a] Literate but did not attend school, are also included. ( ) Based on 10-20 unweighted cases. -- Percentage not shown for less than 10 cases.
** Unweighted cases.

TABLE 6.11 KNOWLEDGE ABOUT THE PLACE WHERE HIV/AIDS TEST CAN BE DONE

Percentage of ever married women aged 15-49 years having knowledge about place where HIV/AIDS test can be done according to selected background characteristics, Haryana 2012-13.

Background Characteristics	Who know the place of HIV/AIDS test	Total women heard of HIV/AIDS**	Places where people can go to get tested for HIV /AIDS: Government: Hospital/ dispensary	Government: CHC/PHC/ Sub-Health Centre	Government: VCTC/ICTC/ RTI/STI Clinic	Government: Other public/ NGO hospItal	Private: Hospital/ Clinic	Private: VCTC/ICTC/ RTI/STI Clinic	Private: Other Private centre	Number of women who know the place for HIV/AIDS test**
**Age Group**										
15-19	37.9	346	37.2	3.6	2.8	--	51.7	3.3	--	133
20-24	41.3	2,455	40.5	2.8	1.3	0.5	49.8	4.1	--	1,018
25-29	43.8	3,361	42.1	3.6	1.1	0.1	47.6	4.5	0.2	1,488
30-34	40.1	2,997	44.8	3.3	1.0	0.4	45.7	3.6	0.2	1,215
35-39	39.2	2,651	45.1	4.0	1.2	0.5	45.5	2.7	--	1,050
40-44	37.8	2,064	44.6	3.6	1.8	0.4	42.6	4.9	--	782
45-49	38.8	1,345	46.7	4.6	0.7	0.2	43.5	1.9	0.5	531
**Residence**										
Rural	37.6	7,763	41.6	4.4	1.3	0.3	47.3	3.8	0.1	2,918
Urban	44.3	7,456	45.4	2.6	1.2	0.3	45.4	3.7	0.1	3,299
**Age at consummation of marriage**										
Below 18 years	36.9	3,688	44.8	3.0	1.1	0.3	45.1	3.6	0.2	1,359
18 years & above	41.7	10,997	42.5	3.7	1.3	0.4	47.4	3.6	0.1	4,632
**Marital Duration**										
0-4	44.3	3,153	38.6	3.2	1.8	0.4	50.9	4.4	0.1	1,413
5-9	43.3	2,874	43.5	3.2	0.6	0.2	47.1	4.3	0.1	1,255
10-14	39.6	2,842	43.4	3.8	1.2	0.4	46.6	3.2	0.1	1,141
15+	37.4	5,870	45.6	3.8	1.2	0.3	44.1	3.0	0.2	2,206
**Education**										
Non-literate[a]	26.5	2,407	52.1	4.8	0.6	0.2	38.3	1.8	--	641
Less than 5 yrs	35.6	2,035	47.9	4.2	0.4	0.8	41.9	3.8	0.2	718
5-9 years	37.5	2,252	49.2	4.5	1.0	0.2	41.1	3.2	0.1	846
10 or more years	46.7	8,525	39.8	3.1	1.6	0.3	49.7	4.2	0.1	4,013
**Husband's Education**										
Non-literate[a]	29.5	1,525	49.8	5.4	1.2	0.2	38.8	3.2	--	449
Less than 5 years	34.2	1,488	55.2	2.8	1.0	0.6	36.4	2.2	0.2	510
5-9 years	36.6	1,951	47.2	4.2	1.1	0.2	42.0	3.8	0.2	712
10 or more years	43.9	10,255	40.8	3.4	1.3	0.3	49.0	4.0	0.1	4,547
**Religion**										
Hindu	40.5	14,297	43.6	3.3	1.2	0.3	46.5	3.8	0.1	5,841
Muslim	26.6	281	36.0	4.1	3.0	--	51.1	5.9	--	75
Christian	(38.6)	18	--	--	--	--	--	--	--	06
Sikh	46.5	584	43.7	9.0	1.3	0.3	41.0	3.4	--	277
Jain	52.3	27	(23.1)	(7.7)	--	--	(53.8)		--	13
Other	--	03	--	--	--	--	--	--	--	01

Contd…

TABLE 6.11 KNOWLEDGE ABOUT THE PLACE WHERE HIV/AIDS TEST CAN BE DONE

Background Characteristics	Who know the place of HIV/AIDS test	Total women heard of HIV/AIDS**	Places where people can go to get tested for HIV /AIDS: Government: Hospital/ dispensary	Government: CHC/PHC/ Sub-Health Centre	Government: VCTC/ICTC/ RTI/STI Clinic	Government: Other public/NGO hospltal	Private: Hospital/ Clinic	Private: VCTC/ICTC/ RTI/STI Clinic	Private: Other Private centre	Number of women who know the place for HIV/AIDS test**
**Castes/Tribes**										
Scheduled Caste	35.9	3454	47.7	5.1	0.7	0.3	41.8	3.6	0.1	1,243
Scheduled Tribes	30.0	641	44.3	4.1	1.0	--	44.7	5.4	--	193
Other Backward Classes	40.3	4169	44.2	3.5	1.2	0.7	45.7	3.0	0.1	1,696
Others	44.0	6955	41.1	2.9	1.5	0.2	48.8	4.2	0.2	3,086
**DLHS-4**	40.5	15219	43.4	3.6	1.2	0.3	46.4	3.8	0.1	6,218
**DLHS-3**	71.0	13895	54.3	10.6	0.2	0.5	33.8	0.4	0.2	9,869

Note: Total figure may not add to 100 percent due to 'do not know' or 'missing cases. CHC= Community Health Centre; PHC= Primary Health Centre; VCTC/ICTC= voluntary/Integrated counseling and testing centre, NGO = Non Governmental Organization. [a] Literate but did not attend school, are also included. ( ) Based on 10-20 unweighted cases. -- Percentage not shown for less than 10 cases. ** Unweighted cases

TABLE 6.12 UNDERGONE HIV/AIDS TEST

Percentage of ever married women aged 15-49 years undergone for HIV/AIDS test and time to be tested for HIV/AIDS, according to selected background characteristics, Haryana, 2012-13.

Background Characteristics	Who have been tested for HIV	Number of women heard HIV/AIDS**	Percentage who have been tested for HIV: Less than 12 months ago	Percentage who have been tested for HIV: 1 or more than 1 years ago	Number of women went for HIV/AIDS test**
**Age group**					
15-19	8.9	346	71.6	28.4	32
20-24	17.8	2,455	41.4	58.6	442
25-29	19.2	3,361	28.3	71.7	635
30-34	12.2	2,997	19.9	80.1	359
35-39	8.9	2,651	18.4	81.6	230
40-44	6.1	2,064	18.7	81.3	123
45-49	5.5	1,345	12.8	87.2	71
**Residence**					
Rural	11.7	7,763	29.4	70.6	982
Urban	13.8	7,456	26.7	73.3	910
**Age at consummation of marriage**					
Below 18 years	9.6	3,688	21.5	78.5	354
18 years & above	14.0	10,997	29.9	70.1	1,504
**Marital duration**					
0-4	18.0	3,153	47.5	52.5	561
5-9	20.3	2,874	22.9	77.1	577
10-14	12.5	2,842	15.3	84.7	351
15+	6.5	5,870	19.8	80.2	370
**Education**					
Non-literate[a]	7.0	2,407	19.0	81.0	172
Less than 5 yrs	9.5	2,035	29.8	70.2	196
5-9 years	10.0	2,252	26.9	73.1	225
10 or more years	15.8	8,525	29.3	70.7	1,298
**Husband's education**					
Non-literate[a]	8.6	1,525	28.0	72.0	134
Less than 5 years	9.3	1,488	23.9	76.1	139
5-9 years	10.6	1,951	28.8	71.2	207
10 or more years	14.1	10,255	28.4	71.6	1,412
**Religion**					
Hindu	12.7	14,297	28.5	71.5	1,786
Muslim	11.4	281	24.2	75.8	32
Christian	(19.8)	18	--	--	03
Sikh	11.4	584	17.7	82.3	67
Jain	12.4	27	--	--	03
Other	--	03	--	--	00
**Castes/Tribes**					
Scheduled Caste	11.9	3,454	28.6	71.4	414
Scheduled Tribes	11.4	641	43.2	56.8	74
Other Backward Classes	11.9	4,169	31.9	68.1	493
Others	13.5	6,955	24.6	75.4	910
**DLHS-4**	12.6	15,219	28.1	71.9	1,891
**DLHS-3**	5.1	13,895	43.3	56.7	702

Note: Total cases may not add up to N due to missing cases.
[a] Literate but did not attend school, are also included. ( ) Based on 10-20 unweighted cases. -- Percentage not shown for less than 10 cases.
** Unweighted cases

TABLE 6.13 HIV/AIDS INDICATORS BY DISTRICTS

Percentage of ever married women aged 15-49 years who have heard of HIV/AIDS, know HIV/AIDS prevention, transmission, places where people can go to get tested for HIV /AIDS and who have been tested for HIV/AIDS in the past 12 months, by districts, Haryana, 2012-13.

District	Who have heard of HIV/AIDS	Who know that HIV/AIDS can be prevented by using condom	Who know that HIV/AIDS can be transmitted from mother to her baby	Who know the places where people can go to get tested for HIV /AIDS	Who ever been tested for HIV/AIDS (%)	Who underwent HIV/AIDS test in the past 12 months among ever tested
Panchkula	81.4	42.3	62.0	43.5	17.3	28.4
Ambala	55.5	52.9	61.0	30.1	9.0	29.9
Yamunanagar	52.5	38.8	45.9	41.6	17.6	42.8
Kurukshetra	53.7	27.7	43.7	37.5	13.4	27.6
Kaithal	66.3	32.4	40.9	49.6	28.3	22.7
Karnal	51.4	29.5	40.4	35.2	12.1	26.8
Panipath	49.9	21.4	32.6	31.0	8.0	39.9
Sonipath	45.8	21.9	25.2	24.7	12.3	33.8
Jind	49.8	31.7	50.1	52.0	20.3	17.9
Fatehabad	48.7	25.4	34.8	46.8	11.4	24.2
Sirsa	53.2	24.8	20.0	52.4	6.2	37.8
Hisar	57.7	27.1	33.8	55.8	4.2	14.7
Bhiwani	55.2	25.8	37.1	57.3	7.9	30.5
Rohtak	54.8	34.3	26.6	28.1	13.8	24.9
Jhajjar	62.2	38.3	31.4	40.6	15.6	31.0
Mahendragarh	44.3	31.5	38.7	53.5	11.3	30.8
Rewari	57.2	36.6	49.6	16.4	4.4	67.5
Gurgaon	55.3	42.1	35.3	47.9	15.6	20.9
Faridabad	51.7	42.3	44.6	45.7	15.4	22.5
Mewat	28.2	29.5	52.3	24.3	5.0	63.1
Palwat	34.8	22.5	46.1	19.5	5.8	31.6
**DLHS-4**	52.1	32.2	39.6	40.5	12.6	28.1
**DLHS-3**	64.4	34.2	13.5	71.0	5.1	43.3

PERSONAL HABITS AND MORBIDITY

TABLE 7.1 PERSONAL HABITS

Percentage of persons (age 15 years and above) who use any kind of tobacco, smoking and drinking habits by selected background characteristics, Haryana, 2012-13.

	Percentage of persons			
Background Characteristics	Percentage who use any kind of tobacco[1]	Percentage who use any kind of smoking	Percentage who Consume alcohol	No. of persons**
**Age group**				
15-24	5.2	4.3	3.9	23,526
25-29	12.4	11.2	9.9	10,506
30-34	16.3	14.4	12.7	9,019
35-39	18.3	16.7	12.9	8,428
40-44	20.6	18.9	13.6	7,227
45-49	23.9	22.2	16.1	5,893
50+	22.7	20.9	12.4	23,523
**Sex**				
Male	29.3	26.8	20.2	40,830
Female	2.3	1.9	0.6	46,415
**Residence**				
Rural	17.6	16.1	10.6	51,685
Urban	12.9	11.5	10.0	36,437
**Education**				
Non-literate[a]	18.3	16.3	9.6	24,782
Less than 5 years	23.9	21.6	14.3	2,014
5-9 years	18.3	16.7	12.0	21,882
10 or more years	12.5	11.5	9.8	39,444
**Religion**				
Hindu	16.0	14.6	10.7	81,034
Muslim	19.2	16.8	7.2	3,545
Christian	13.1	13.1	11.7	135
Sikh	8.1	6.0	7.4	3,178
Jain	3.1	3.1	3.4	113
Others	13.9	13.9	17.6	38
**Castes/Tribes**				
Scheduled Caste	18.4	16.7	11.8	24,276
Scheduled Tribes	14.8	12.9	9.6	4,772
Other Backward Classes	16.2	14.7	10.3	24,348
Others	13.9	12.8	9.6	34,726
**DLHS-4**	15.9	14.4	10.4	88,122*

[a] Literate but did not attend the school are also included. [1]Includes smoking.* Missing cases are excluded. ** Unweighted cases.

TABLE 7.2 PERSONAL HABITS-MEN

Percentage of Men (age 15 years and above) classified as having personal habits by selected background characteristics, Haryana, 2012-13.

Background characteristics	Percentage of men			Total number of Men covered**
	Using Smokeless Tobacco	Smoking	Consuming Alcohol	
**Age of the men**				
15-19	1.2	3.0	2.4	6,112
20-24	3.6	12.7	11.9	5,493
25-29	5.4	22.3	19.7	4,740
30-34	6.9	27.7	24.9	4,124
35-39	6.4	33.4	25.9	3,680
40-44	6.3	35.7	26.5	3,290
45 years and above	6.4	38.3	25.4	13,391
**Residence**				
Rural	5.4	29.6	20.6	23,673
Urban	5.3	22.1	19.5	17,157
**Education**				
Non-literate[a]	9.6	41.2	27.2	7,190
Less than 5 years	7.3	36.8	24.9	1,048
5-9 years	6.3	30.7	22.3	10,698
10 or more years	3.2	19.2	16.3	21,894
**Religion**				
Hindu	5.2	27.2	20.7	37,581
Muslim	8.6	31.0	14.1	1,634
Christian	0.0	24.0	21.5	64
Sikh	5.3	11.6	14.1	1,439
Jain	1.8	6.2	6.7	57
Others	11.0	22.3	28.2	22
**Castes/Tribes**				
Scheduled Caste	6.3	30.4	22.4	11,354
Scheduled Tribes	7.0	23.6	18.6	2,224
Other Backward Classes	5.6	27.5	20.2	11,323
Others	4.2	24.1	18.8	15,929
**DLHS-4**	5.3	26.8	20.2	40,830*

[a] Literate but did not attend the school are also included. *Missing cases are excluded. ** Unweighted cases.

TABLE 7.3 PERSONAL HABITS-WOMEN
Percentage of Women (age 15 years and above) classified as having personal habits by selected background characteristics, Haryana, 2012-13.

Background characteristics	Percentage of women			Total number of women covered**
	Using Smokeless Tobacco	Smoking	Consuming Alcohol	
**Age of the women**				
15-19	0.2	0.3	0.2	5,551
20-24	0.4	0.4	0.3	6,172
25-29	0.4	0.5	0.5	5,654
30-34	0.5	1.0	0.4	4,795
35-39	0.6	1.1	0.7	4,655
40-44	0.6	1.9	0.6	3,874
45 years and above	1.0	4.0	0.8	15,714
**Residence**				
Rural	0.8	2.5	0.6	27,477
Urban	0.4	0.9	0.5	18,938
**Education**				
Non-literate[a]	1.1	3.9	0.8	17,356
Less than 5 years	0.6	1.4	0.3	939
5-9 years	0.6	0.9	0.6	10,967
10 or more years	0.2	0.5	0.3	17,153
**Religion**				
Hindu	0.6	1.9	0.6	42,675
Muslim	2.2	3.4	0.7	1,861
Christian	0.0	0.0	0.0	71
Sikh	0.5	0.4	0.6	1,701
Jain	0.0	0.0	0.0	54
Others	(0.0)	(0.0)	(0.0)	11
**Castes/Tribes**				
Scheduled Caste	0.8	2.3	0.7	12,658
Scheduled Tribes	1.1	2.2	0.7	2,505
Other Backward Classes	0.6	1.9	0.5	12,787
Others	0.5	1.5	0.5	18,465
**DLHS-4**	0.6	1.9	0.6	46,415*

[a] Literate but did not attend the school are also included. ( ) based on 10-20 unweighted cases. * Missing cases are excluded. ** Unweighted cases.

TABLE 7.4 PERSONAL HABITS
Percentage ofl persons (age 15 years and above) classified as having personal habits by districts, Haryana, 2012-13.

District	Percentage of persons: Using Smokeless Tobacco	Smoking	Consuming Alcohol	Total number of all persons covered**
Panchkula	3.4	12.4	13.4	3,616
Ambala	5.3	10.6	11.1	4,710
Yamunanagar	5.7	9.0	7.0	4,231
Kuruksherta	1.0	13.8	9.3	5,306
Kaithal	3.8	15.4	13.0	5,802
Karnal	0.7	12.6	7.6	5,738
Panipat	4.5	14.8	8.6	4,264
Sonipat	2.7	11.3	7.6	4,179
Jind	1.6	16.5	12.5	5,294
Fatehabad	6.3	8.7	7.3	2,979
Sirsa	4.9	13.2	11.7	5,147
Hisar	3.0	17.6	10.3	4,703
Bhiwani	2.0	23.3	13.5	3,520
Rohtak	2.4	13.2	10.4	3,645
Jhajjar	2.4	15.9	12.5	4,683
Mahendragarh	3.3	16.5	8.0	3,432
Rewari	1.7	14.2	6.0	3,197
Gurgaon	4.8	19.2	19.2	3,873
Faridabad	1.3	12.1	12.5	3,462
Mewat	2.9	15.1	5.4	3,339
Palwal	1.4	15.0	8.2	3,002
**DLHS-4**	3.0	14.4	10.4	88,122

** Unweighted cases.

TABLE 7.5 PERSONAL HABITS TOBACCO
Percentage of men and women age 15 years having habits of chewing Tobacco, Haryana, 2012-13.

	Tobacco chewing						
	Women			Men			
**Tobacco use**	Rural	Urban	Total	Rural	Urban	Total	Total
**Use of Tobacco**							
Pan with tobacco	0.3	0.2	0.3	1.9	2.0	1.9	1.1
Guthaka/ Pan masala with tobacco	0.0	0.0	0.0	0.4	0.7	0.5	0.3
Other forms of tobacco	0.4	0.1	0.3	3.1	2.6	2.9	1.6
Non-user	97.0	97.6	97.2	92.6	93.0	92.7	94.9
Not known	2.2	2.0	2.2	2.1	1.8	2.0	2.1
**DLHS-4**	0.8	0.4	0.6	5.4	5.3	5.3	3.0

TABLE 7.6 PERSONAL HABITS SMOKE
Percentage of men and women age 15 years having habits of smoking, Haryana, 2012-13.

	Smoking						
	Women			Men			
**Smoking habits**	Rural	Urban	Total	Rural	Urban	Total	Total
Usual smoker*	1.8	0.6	1.3	22.3	14.5	19.4	10.4
Occasional smoker	0.7	0.3	0.6	7.3	7.6	7.4	4.0
Ex-smoker	0.2	0.2	0.2	2.0	1.9	2.0	1.1
Non smoker	95.3	97.4	96.1	66.6	74.6	69.6	82.8
Not known	2.0	1.6	1.8	1.7	1.4	1.6	1.7
**DLHS-4**	2.5	0.9	1.9	29.6	22.1	26.8	14.4

* At least once every day

TABLE 7.7 PERSONAL HABITS DRINK ALCOHOL
Percentage of men and women age 15 years having habits of drinking alcohol, Haryana, 2012-13.

	Drinking alcohol						
	Women			Men			
**Drinking habits**	Rural	Urban	Total	Rural	Urban	Total	Total
Usual drinker*	0.2	0.2	0.2	6.3	5.3	5.9	3.1
Occasional drinker	0.3	0.4	0.3	14.3	14.2	14.3	7.3
Ex-drinker	0.2	0.2	0.2	3.1	3.0	3.0	1.6
Non drinker	97.1	97.5	97.3	74.5	76.1	75.1	86.2
Not known	2.1	1.7	2.0	1.9	1.4	1.7	1.8
**DLHS-4**	0.6	0.5	0.6	20.6	19.5	20.2	10.4

* At least once every week

TABLE 7.8 MORBIDITY DETAILS
Prevalence of any injury, acute illness and chronic illness according to place of residence, Haryana, 2012-13.

		Residence	
**Morbidity**	Total	Rural	Urban
**Prevalence Rate of Any Injury[1]**			
Male	1.8	1.9	1.4
Female	1.3	1.4	1.2
Total	1.6	1.7	1.3
**Prevalence Rate of Acute Illness[2]**			
Male	12.5	12.7	12.2
Female	13.8	14.2	12.9
Total	13.1	13.4	12.5
**Prevalence Rate of Chronic Illness[1]**			
Male	12.5	11.9	13.7
Female	18.7	18.5	19.1
Total	15.4	15.0	16.2

[1] During last one year, [2] During last fifteen days

TABLE 7.9 MORBIDITY DETAILS
Percentage of household population having any form of disability as on the day of survey, Haryana, 2012-13.

	**Total**			**Rural**			**Urban**		
**Type of Disability**	**Male**	**Female**	**Total**	**Male**	**Female**	**Total**	**Male**	**Female**	**Total**
Mental Disability	0.3	0.2	0.2	0.3	0.2	0.2	0.2	0.2	0.2
Visual Disability	0.3	0.3	0.3	0.3	0.3	0.3	0.2	0.3	0.3
Hearing Disability	0.1	0.1	0.1	0.1	0.1	0.1	0.1	0.1	0.1
Speech Disability	0.1	0.1	0.1	0.1	0.1	0.1	0.1	0.0	0.1
Number of persons**	87983	78245	166228	52373	46853	99226	35610	31392	67002

** Unweighted cases & missing/others cases are excluded.

TABLE 7.10 MORBIDITY DETAILS
Percentage of household population having any injury and received treatment during last one year, Haryana, 2012-13.

	**Total**			**Rural**			**Urban**		
**Type of treatment**	**Male**	**Female**	**Total**	**Male**	**Female**	**Total**	**Male**	**Female**	**Total**
Treated in intensive care unit for any time	4.4	4.6	4.4	4.4	4.6	4.5	4.3	4.6	4.4
Treated as in-patient with stay <1 week	12.9	11.6	12.5	12.6	11.8	12.3	13.6	11.2	12.9
Treated as in-patient with stay 1-2 week	6.7	6.6	6.6	7.0	6.3	6.8	6.0	7.2	6.4
Treated as in-patient with stay >2 week	9.5	9.0	9.4	9.5	9.4	9.5	9.5	8.1	9.1
Other treatment*	66.5	68.2	67.0	66.5	67.8	66.9	66.6	68.9	67.3
Number of persons**	4409	1821	6230	2679	1118	3797	1730	703	2433

*Out patient/traditional healer/at home. ** Unweighted cases & missing/others cases are excluded.

TABLE 7.11 MORBIDITY DETAILS
Percentage of household population having acute illness during last 15 days, Haryana, 2012-13.

	Total			Rural			Urban		
**Type of acute illness**	**Male**	**Female**	**Total**	**Male**	**Female**	**Total**	**Male**	**Female**	**Total**
Diarrhoea/ Dysentery	4.6	4.1	4.4	4.6	3.8	4.2	4.5	4.9	4.7
Acute respiratory tract infection	28.9	25.6	27.3	26.8	23.0	24.9	32.9	31.2	32.1
Jaundice with fever	1.7	1.5	1.6	1.8	1.6	1.7	1.4	1.3	1.4
Malaria	12.8	13.4	13.1	14.4	14.8	14.6	9.6	10.5	10.0
Fever of short duration with rashes	2.8	2.8	2.8	2.9	3.1	3.0	2.5	2.4	2.4
Reproductive tract infection	0.6	0.6	0.6	0.6	0.6	0.6	0.6	0.5	0.6
Other type of fever	36.7	39.0	37.8	37.3	41.2	39.3	35.4	34.4	34.9
Other	12.1	12.9	12.5	11.6	12.0	11.8	13.1	14.7	13.9
Number of persons**	10,984	10,717	21,701	6,621	6,667	13,288	4,363	4,050	8,413

** Unweighted cases & missing/others cases are excluded.

TABLE 7.12 MORBIDITY DETAILS
Percentage of household population having acute illness during last 15 days and received treatment by type of health facilities, Haryana, 2012-13.

	Total			Rural			Urban		
**Place of treatment**	**Male**	**Female**	**Total**	**Male**	**Female**	**Total**	**Male**	**Female**	**Total**
**Government health facility**									
Sub-Health Centre	0.4	0.4	0.4	0.4	0.4	0.4	0.4	0.2	0.3
Primary health centre	0.6	0.9	0.8	0.8	1.1	1.0	0.2	0.5	0.3
Community Health centre	0.6	0.4	0.5	0.5	0.4	0.5	0.6	0.5	0.6
UHC/UHP/UFWC	0.1	0.1	0.1	0.0	0.1	0.0	0.1	0.1	0.1
Dispensary/ clinic	0.7	0.8	0.8	0.6	0.8	0.7	0.9	1.0	0.9
Hospital	6.5	7.0	6.8	6.1	6.7	6.4	7.4	7.7	7.5
AYUSH hospital/clinic	0.2	0.3	0.3	0.2	0.3	0.2	0.3	0.3	0.3
**Private health facility**									
Dispensary/ clinic	60.9	60.5	60.7	62.5	62.5	62.5	57.7	56.4	57.1
Hospital	18.8	18.2	18.5	18.2	17.2	17.7	20.1	20.4	20.2
AYUSH hospital/clinic	0.6	0.6	0.6	0.6	0.7	0.6	0.5	0.6	0.5
NGO/ trust hospital	0.4	0.4	0.4	0.4	0.5	0.4	0.5	0.4	0.4
Other*	2.5	3.3	2.9	2.3	2.9	2.6	3.0	4.1	3.5
Number of persons**	10,866	10,616	21,482	6,547	6,605	13,152	4,319	4,011	8,330

* DOT centre and at home. ** Unweighted cases & missing/others cases are excluded.

TABLE 7.13 MORBIDITY DETAILS

Percentage of household population having main symptoms of chronic illness persisting for more than one month and sought medical care and source of treatment, Haryana, 2012-13.

	Total			Rural			Urban		
**Place of treatment**	**Male**	**Female**	**Total**	**Male**	**Female**	**Total**	**Male**	**Female**	**Total**
**Prevalence Of Chronic Illness**									
Disease of respiratory system	12.2	7.5	9.5	13.5	8.1	10.4	10.0	6.4	8.0
Disease of cardiovascular system	6.8	4.4	5.4	6.3	4.2	5.1	7.6	4.6	5.9
Disease of central nervous system	5.7	5.0	5.3	6.1	5.1	5.5	5.2	4.7	4.9
Disease of musculoskeletal system	17.9	28.1	23.7	19.7	29.5	25.4	15.0	25.4	20.7
Disease of gastrointestinal system	7.7	7.2	7.4	8.1	7.7	7.9	6.9	6.4	6.6
Disease of genitourinary system	2.6	3.2	2.9	2.6	3.2	3.0	2.6	3.1	2.9
Skin disease	5.9	4.4	5.1	6.6	5.2	5.8	4.7	3.0	3.8
Goitre	0.2	0.4	0.3	0.2	0.4	0.3	0.2	0.6	0.4
Elephantiasis	0.5	0.6	0.6	0.5	0.6	0.6	0.5	0.6	0.6
Eye problem	3.6	3.1	3.3	3.6	3.2	3.4	3.6	3.1	3.3
ENT problem	4.0	3.5	3.7	4.2	3.5	3.8	3.6	3.5	3.5
Mouth and dental problem	1.1	1.1	1.1	1.4	1.2	1.3	0.8	0.8	0.8
Other	31.7	31.5	31.6	27.1	28.1	27.7	39.3	37.8	38.5
**Sought Medical Care**									
Details of Diagnosis/Treatment available	57.1	52.9	54.7	53.3	49.6	51.1	63.2	59.1	60.9
Details of Diagnosis/Treatment not available	30.0	32.4	31.4	31.6	34.0	33.0	27.4	29.4	28.5
Not at all	12.9	14.7	13.9	15.2	16.4	15.9	9.4	11.5	10.6
**Source of Treatment**									
At government health facility	20.4	17.8	18.9	21.0	17.6	19.0	19.5	18.0	18.7
At private health facility	76.8	79.3	78.2	76.1	79.4	78.0	77.7	79.1	78.4
At home	0.9	0.9	0.9	0.8	0.7	0.8	1.0	1.1	1.0
Other	2.0	2.1	2.0	2.1	2.2	2.2	1.8	1.8	1.8

TABLE 7.14 MORBIDITY DETAILS

Percentage of household population diagnosed with chronic illness during last one year, Haryana, 2012-13.

	Total			Rural			Urban		
**Diagnosed chronic illness**	**Male**	**Female**	**Total**	**Male**	**Female**	**Total**	**Male**	**Female**	**Total**
Diabetes	10.3	6.2	8.0	6.0	4.1	4.9	17.2	10.1	13.3
Hypertension	10.2	13.8	12.2	8.5	12.5	10.8	12.9	16.3	14.8
Disease related to heart*	6.2	2.9	4.3	6.0	2.7	4.1	6.5	3.2	4.7
Epilepsy	1.0	0.6	0.8	1.0	0.6	0.8	0.8	0.6	0.7
Asthma/chronic respiratory failure	4.2	2.9	3.5	4.8	3.2	3.8	3.2	2.4	2.8
Goitre/ thyroid disorder	0.8	1.2	1.1	0.9	1.0	1.0	0.7	1.6	1.2
Tuberculosis	1.9	0.9	1.3	2.1	0.9	1.4	1.5	1.0	1.2
Leprosy	0.5	0.8	0.7	0.6	0.9	0.8	0.4	0.7	0.6

* Chronic heart diseases, Myocardial infection/heart attack, stroke cerebro vascular accident.

TABLE 7.15 MORBIDITY DETAILS

Percentage of household population aged 60 years and above diagnosed with chronic illness during last one year, Haryana, 2012-13.

	Total			Rural			Urban		
**Diagnosed chronic illness**	**Male**	**Female**	**Total**	**Male**	**Female**	**Total**	**Male**	**Female**	**Total**
Diabetes	12.4	10.3	11.3	7.4	7.2	7.3	21.6	16.2	18.6
Hypertension	12.6	16.0	14.4	10.2	13.8	12.2	16.9	20.1	18.6
Disease related to heart*	7.9	4.4	6.0	7.6	3.9	5.6	8.4	5.2	6.7
Asthma/chronic respiratory failure	7.3	4.6	5.8	8.4	5.2	6.6	5.5	3.6	4.4
Goitre/ thyroid disorder	0.6	0.7	0.6	0.6	0.5	0.5	0.6	1.0	0.8
Tuberculosis	2.0	1.0	1.5	2.3	1.0	1.6	1.4	1.0	1.2
Leprosy	0.8	1.1	1.0	1.0	1.2	1.1	0.6	0.8	0.7
Cataract	1.0	1.2	1.1	1.0	1.2	1.1	0.9	1.3	1.1
Stroke	0.9	0.6	0.8	0.9	0.4	0.6	0.9	1.0	1.0

* Chronic heart diseases, Myocardial infection/heart attack, stroke cerebro vascular accident.

TABLE 7.16 TUBERCULOSIS
Number of persons who have tuberculosis by background characteristics, Haryana, 2012-13.

Background characteristics	Number of persons suffering from tuberculosis			Number of persons**
	Rural	Urban	Total	
**Age group**				
15-19	08	10	18	17,083
20-34	36	34	70	44,441
35-44	32	25	57	21,046
45-59	44	26	70	22,266
60 +	71	37	108	16,793
**Education**				
Non-literate[a]	109	45	154	52,908
Less than 5 years	11	05	16	13,474
5-9 years	53	32	85	42,267
10 or more years	35	54	89	57,685
**Religion**				
Hindu	190	128	318	1,51,596
Muslim	14	04	18	8,591
Christian	00	00	00	219
Sikh	04	04	08	5,439
Jain	00	00	00	191
Others	00	00	00	77
**Castes/Tribes**				
Scheduled Caste	81	48	129	47,780
Scheduled Tribes	12	12	24	8,802
Other Backward Classes	60	33	93	46,233
Others	55	43	98	63,519
**DLHS-4**	208	136	344	1,66,334

[a] Literate but did not attend the school are also included. ** Unweighted cases.

HEALTH AND NUTRITIONAL STATUS

TABLE 8.1 NUTRITIONAL STATUS OF CHILDREN

Percentage of children under age five years classified as malnourished according to three anthropometric indices of nutritional status: height-for-age, weight-for-height, and weight-for-age, by background characteristics, Haryana, 2012-13.

	Height-for-Age				Weight-for-Height				Weight-for-Age				Number of
Background Characteristics	Below -3SD	Below -2SD	Above +2SD*	Mean Z-score (SD)	Below -3SD	Below -2SD	Above +2SD*	Mean Z-score (SD)	Below -3SD	below -2SD	Above +2SD*	Mean Z-score SD)	eligible children**
**Age group (month)**													
0-6	6.3	11.9	88.1	32.6	28.0	39.6	60.4	17.4	12.8	30.4	69.6	24.0	1,032
7-12	7.7	14.3	85.7	35.5	19.5	38.0	62.0	12.5	12.0	27.3	72.7	26.0	1,416
13-18	14.2	32.1	67.9	18.7	22.5	33.8	66.2	10.0	14.8	35.2	64.8	9.1	1,140
19-24	30.1	42.1	57.9	29.3	15.7	29.3	70.7	7.6	15.9	38.0	62.0	13.1	1,133
25-35	19.3	36.7	63.3	19.8	18.7	31.2	68.8	7.8	18.3	40.0	60.0	7.9	2,338
36 +	13.7	34.6	65.4	13.0	16.5	30.4	69.6	4.8	14.5	36.8	63.2	4.9	4,883
**Sex of child**													
Male	15.5	32.3	67.7	20.3	19.4	33.2	66.8	7.6	15.2	37.2	62.8	10.0	6,616
Female	15.3	31.6	68.4	19.4	17.7	31.1	68.9	7.9	15.2	34.8	65.2	9.9	5,537
**Place of residence**													
Rural	15.8	32.1	67.9	20.7	19.2	33.5	66.5	7.6	16.0	38.0	62.0	9.6	7,744
Urban	14.8	31.8	68.2	18.4	17.6	30.3	69.7	7.9	13.8	32.9	67.1	10.5	4,409
**Religion**													
Hindu	16.4	33.9	66.1	17.8	18.6	32.0	68.0	7.2	15.4	37.1	62.9	8.8	10,453
Muslim	6.0	14.4	85.6	39.3	18.2	32.1	67.9	14.1	11.1	25.1	74.9	20.8	1,348
Christian	--	--	--	--	--	--	--	--	--	--	--	--	06
Sikh	20.6	39.1	60.9	13.0	20.2	40.4	59.6	6.4	24.7	50.2	49.8	4.5	333
Jain	--	--	--	--	--	--	--	--	--	--	--	--	08
Others	--	--	--	--	--	--	--	--	.--	--	--	--	05
**Castes/Tribes**													
Scheduled Caste	15.8	33.0	67.0	19.5	19.7	34.6	65.4	7.3	16.4	37.3	62.7	9.6	4,152
Scheduled Tribes	11.8	28.7	71.3	23.0	18.5	33.2	66.8	9.3	14.4	37.7	62.3	8.6	721
Other Backward Classes	15.7	31.8	68.2	21.8	17.5	30.8	69.2	8.6	13.9	34.2	65.8	11.6	3,581
Others	15.4	31.8	68.2	17.9	18.4	31.0	69.0	7.1	15.1	36.4	63.6	9.1	3,699
**Haryana**	15.4	32.0	68.0	19.9	18.6	32.3	67.7	7.7	15.2	36.2	63.8	9.9	12,153

Note: reference period: January 1st, 2008 to survey date. * +2SD includes Don't know. -- Percentage not shown for less than 10 cases.* *unweighted cases

TABLE 8.2 NUTRITIONAL STATUS OF CHILDREN BY DISTRICTS

Percentage of children under age five years classified as malnourished according to three anthropometric indices of nutritional status: height-for-age, weight-for-height, and weight-for-age, by districts, Haryana 2012-13.

	Height-for-Age				Weight-for-Height				Weight-for-Age				Number of
District	Below -3SD	Below -2SD	Above +2SD*	Mean Z-score (SD)	Below -3SD	Below -2SD	Above +2SD*	Mean Z-score (SD)	Below -3SD	Below -2SD	Above +2SD*	Mean Z-Score (SD)	eligible children**
Panchkula	18.3	42.6	57.4	6.7	15.5	33.5	66.5	9.1	25.7	50.9	49.1	3.2	200
Ambala	24.2	48.4	51.6	7.1	15.0	28.6	71.4	4.0	17.0	39.9	60.1	3.7	276
Yamunanagar	21.3	41.6	58.4	8.5	17.2	31.5	68.5	8.2	18.0	46.4	53.6	3.8	448
Kurukshetra	10.3	19.6	80.4	22.2	28.0	41.8	58.2	8.9	13.8	31.5	68.5	13.3	695
Kaithal	27.0	59.0	41.0	4.1	17.6	31.9	68.1	6.8	20.9	51.6	48.4	5.4	488
Karnal	8.5	16.9	83.1	22.7	32.0	45.9	54.1	9.4	16.5	33.5	66.5	9.2	885
Panipat	9.2	19.2	80.8	29.0	23.5	37.4	62.6	10.2	11.6	29.0	71.0	14.7	845
Sonipat	12.3	28.8	71.2	17.0	17.7	29.7	70.3	11.6	12.7	35.5	64.5	9.2	583
Jind	23.2	46.8	53.2	14.7	16.0	26.6	73.4	4.6	15.4	43.1	56.9	5.6	482
Fatehabad	16.9	35.1	64.9	19.5	15.0	34.4	65.6	3.7	17.1	41.8	58.2	7.8	487
Sirsa	17.3	39.3	60.7	11.4	12.6	35.4	64.6	4.1	19.0	45.0	55.0	5.0	814
Hisar	22.8	43.2	56.8	12.5	9.8	23.0	77.0	3.1	15.6	41.0	59.0	5.2	756
Bhiwani	20.0	42.4	57.6	9.3	11.0	24.0	76.0	4.4	13.5	38.5	61.5	3.8	486
Rohatak	14.6	30.4	69.6	14.6	26.2	36.2	63.8	8.4	13.4	36.2	63.8	4.2	369
Jhajjar	17.9	37.3	62.7	14.6	22.6	35.2	64.8	5.7	16.2	40.8	59.2	5.9	496
Mahendragarh	19.9	38.6	61.4	11.1	5.1	15.2	84.8	4.7	10.9	26.7	73.3	4.3	441
Rewari	10.1	16.7	83.3	28.1	26.6	41.7	58.3	14.2	18.3	33.3	66.7	18.7	486
Gurgaon	18.9	42.7	57.3	11.1	18.2	31.3	68.7	8.0	12.1	38.3	61.7	4.3	412
Faridabad	14.7	35.2	64.8	28.2	17.4	31.5	68.5	6.2	14.2	31.0	69.0	17.9	467
Mewat	2.7	6.7	93.3	50.8	22.6	32.6	67.4	19.6	11.8	19.5	80.5	28.5	1,129
Palwal	11.7	20.7	79.3	33.8	21.8	33.8	66.2	9.3	14.5	30.3	69.7	13.8	908
**Haryana**	15.4	32.0	68.0	19.9	18.6	32.3	67.7	7.7	15.2	36.2	63.8	9.9	12,153

Note: reference period: January 1st, 2008 to survey date. * +2SD includes Don't know. ** unweighted cases

TABLE 8.3 BMI (BODY MASS INDEX) OF WOMEN

Percentage of women aged 15-49 average body mass index (BMI), and percentage with specific BMI levels, by background characteristics, Haryana, 2012-13.

Background characteristics	Mean Height	Mean BMI	18.5-24.9 (normal)	Thin: <18.5 (total thin)	Thin: 17.0-18.4 (mildly thin)	Thin: <17.0 (moderately/ severely thin)	Overweight/Obese: ≥25.0 (overweight or obese)	Overweight/Obese: 25.0-29.9 (overweight)	Overweight/Obese: ≥30.0 (obese)	Total number of Women**
**Age group**										
15-19	153.4	19.61	51.3	44.4	20.4	23.0	4.2	3.3	0.9	4,917
20-29	155.7	22.18	62.3	24.2	12.2	11.6	13.5	10.5	2.9	9,979
30–39	156.3	23.52	60.6	14.4	7.2	6.8	25.1	18.1	6.8	8,417
40-49	156.7	24.08	56.2	11.9	6.4	5.3	31.9	22.7	9.1	6,065
**Place of residence**										
Rural	155.7	21.92	59.2	25.2	12.3	12.5	15.6	11.8	3.7	17,117
Urban	155.7	23.37	57.7	17.4	8.7	8.2	24.9	17.6	7.1	12,261
**Education**										
Non-literate[a]	155.5	22.21	61.2	21.2	9.9	10.8	17.6	13.3	4.2	7,185
Less than 5 years	154.6	22.01	52.7	28.2	12.0	15.5	19.1	15.6	3.4	618
5-9 years	155.1	22.41	57.2	24.4	11.5	12.5	18.3	13.4	4.9	8,044
10 or more years	156.1	22.79	58.4	21.4	11.1	9.7	20.2	14.6	5.5	13,531
**Religion**										
Hindu	155.7	22.52	58.5	22.4	11.1	10.9	19.1	14.0	5.0	26,988
Muslim	154.2	21.87	64.1	24.8	10.2	13.6	11.1	8.5	2.5	1,249
Christian	153.6	23.32	59.6	17.9	8.2	9.7	22.5	11.9	10.7	44
Sikh	156.4	23.41	57.3	17.9	9.2	8.2	24.8	18.4	6.3	1,030
Jain	155.5	24.69	58.0	7.4	5.2	2.2	34.6	15.5	19.1	36
Others	--	--	--	--	--	--	--	--	--	07
**Castes/Tribes**										
Scheduled Caste	155.0	21.82	57.9	28.1	12.7	14.9	14.0	10.8	3.2	8,214
Scheduled tribes	155.5	22.06	59.2	25.3	12.0	12.5	15.5	11.5	3.8	1,554
Other backward classes	155.5	22.34	59.4	22.3	11.1	10.6	18.3	14.1	4.1	8,252
Others	156.3	23.23	58.7	17.6	9.4	7.8	23.7	16.6	7.0	11,358
**Haryana**	155.7	22.53	58.7	22.3	11.0	10.9	19.0	14.0	5.0	29,378

Note: reference period: January 1st, 2008 to survey date.
[a] Literate but did not attend school, are also included. -- Percentage not shown for less than 10 cases. ** unweighted cases

TABLE 8.4 BMI (BODY MASS INDEX) OF WOMEN (new)

Percentage of women aged 15-49 average body mass index (BMI), and percentage with specific BMI levels, by district, Haryana, 2012-13

District	Mean Height	Mean BMI	18.5-24.9 (normal)	Thin: <18.5 (total thin)	Thin: 17.0-18.4 (mildly thin)	Thin: <17.0 (moderately/ severely thin)	Overweight/Obese: ≥25.0 (overweight or obese)	Overweight/Obese: 25.0-29.9 (overweight)	Overweight/Obese: ≥30.0 (obese)	Total number of Women**
Panchkula	157.5	22.6	62.3	19.9	10.8	8.8	17.8	13.3	4.3	1,172
Ambala	157.3	22.1	60.6	21.8	10.3	11.3	17.6	13.5	4.0	1,456
Yamunanagar	155.9	22.2	51.2	27.9	12.7	14.7	20.9	14.7	6.1	1,170
Kurukshetra	156.7	22.9	70.0	12.9	7.1	5.6	17.1	14.8	2.2	1,753
Kaithal	157.3	22.2	59.0	25.2	11.7	13.0	15.8	12.3	3.4	1,896
Karnal	155.4	21.9	64.8	18.0	9.7	7.9	17.2	13.5	3.7	1,959
Panipat	153.9	23.3	56.7	17.4	8.2	8.9	26.0	18.9	7.0	1,450
Sonipat	154.5	23.7	55.0	18.1	9.2	8.7	26.8	18.4	8.4	1,385
Jind	157.2	21.6	62.5	23.5	11.7	11.1	14.0	10.4	3.6	1,773
Fatehabad	154.7	22.1	49.4	28.3	13.1	14.4	22.2	15.4	6.7	1,042
Sirsa	155.2	22.2	51.1	27.4	13.3	13.8	21.5	15.1	6.1	1,810
Hisar	154.0	22.2	49.5	29.0	14.7	13.9	21.5	15.4	5.8	1,689
Bhiwani	154.8	22.2	49.6	26.7	13.3	12.9	23.7	15.6	7.9	1,235
Rohtak	156.5	23.1	53.3	22.3	9.3	12.6	24.4	16.6	7.7	1,232
Jhajjar	156.4	22.2	54.2	25.4	12.7	11.7	20.4	14.7	5.5	1,530
Mahendragarh	155.1	22.2	49.7	26.8	12.6	13.5	23.5	16.5	6.9	1,180
Rewari	155.3	22.6	73.9	14.5	8.2	6.0	11.6	9.5	1.9	1,056
Gurgaon	155.8	24.1	56.6	20.7	10.3	9.9	22.7	17.2	5.4	1,245
Faridabad	155.9	23.6	66.5	13.2	7.0	6.0	20.3	14.6	5.7	1,113
Mewat	153.9	22.1	73.6	18.1	9.0	8.4	8.3	6.4	1.8	1,187
Palwal	154.3	23.0	61.2	21.7	10.1	10.4	17.1	11.8	5.3	1,045
**Haryana**	155.7	22.5	58.7	22.3	11.0	10.9	19.0	14.0	5.0	29,378

** Unweighted cases

TABLE 8.5 PREVELANCE OF ANAEMIA AMONG CHILDREN

Percentage of children age (6-59 months) classified as having iron-deficiency (anaemia) by selected background characteristics, Haryana, 2012-13.

Background characteristics	Anaemia status by haemoglobin level				Total number of children <5 years**
	Mild anaemia (10.0-10.9 g/dl)	Moderate anaemia (7.0-9-9 g/dl)	Severe anaemia (< 7g/dl)	Any anaemia <11.0 g/dl	
**Sex of Child**					
Male	15.9	38.8	8.5	63.2	3,595
Female	16.2	36.5	9.7	62.4	2,885
**Place of residence**					
Rural	15.6	38.3	9.2	63.1	4,187
Urban	17.0	36.8	8.5	62.3	2,294
**Religion**					
Hindu	16.1	37.7	8.7	62.5	5,782
Muslim	15.4	41.4	11.5	68.3	552
Christian	--	--	--	--	03
Sikh	17.3	29.4	7.7	54.4	132
Jain	--	--	--	--	05
Others	--	--	--	--	03
**Castes/Tribes**					
Scheduled Caste	16.1	35.4	9.0	60.5	2,146
Scheduled Tribes	16.4	39.8	9.4	65.6	369
Other Backward Classes	14.7	39.5	10.0	64.3	2,031
Others	17.2	38.4	7.9	63.5	1,935
**Haryana**	16.0	37.8	9.0	62.8	6,481

Note: reference period: January 1st, 2008 to survey date.
-- Percentage not shown for less than 10 cases. ** Unweighted cases

TABLE 8.6 ANAEMIA AMONG SCHOOL GOING/ADOLESCENT POPULATION

Percentage of school going population (age 6-19 years) classified as having iron-deficiency (anaemia) by degree of anaemia and by selected background characteristics, Haryana, 2012-13.

Background characteristics	Anaemia status by haemoglobin level				Total number of school going population (age 6-19 years)**
	Mild anaemia (10.0-10.9 g/dl)	Moderate anaemia (7.0-9-9 g/dl)	Severe anaemia (< 7g/dl)	Any anaemia (<11.0 g/dl)	
**Age group**					
6 - 10	17.9	33.6	6.6	58.1	8,764
11 - 14	17.4	32.8	6.0	56.1	7,464
15– 16	17.5	31.7	5.9	55.1	4,031
17– 19	16.6	30.2	5.7	52.5	6,325
**Sex**					
Male	16.7	31.2	6.0	53.9	14,293
Female	18.1	33.6	6.2	58.0	12,288
**Residence**					
Rural	17.5	32.6	6.0	56.1	16,819
Urban	17.2	31.7	6.2	55.1	9,765
**Education**					
Non-literate[a]	17.7	35.0	6.7	59.4	2,828
Less than 5 years	18.1	33.2	6.4	57.8	6,361
5-9 years	17.3	32.7	6.2	56.2	10,493
10 or more years	16.7	29.8	5.3	51.7	6,902
**Religion**					
Hindu	17.3	32.2	5.9	55.4	23,872
Muslim	18.3	35.5	9.3	63.2	1,892
Christian	8.5	44.6	5.3	58.4	23
Sikh	17.7	26.8	3.3	47.8	747
Jain	17.9	33.6	4.3	55.9	31
Others	--	--	--	--	05
**Castes/Tribes**					
Scheduled Caste	17.6	33.2	6.4	57.2	8,443
Scheduled Tribes	16.9	32.2	6.1	55.1	1,498
Other Backward Classes	17.1	32.8	6.5	56.3	7,825
Others	17.6	30.9	5.4	53.9	8,818
**Haryana**	17.4	32.3	6.1	55.8	26,584

[a] Literate but did not attend school, are also included. -- Percentage not shown for less than 10 cases. **un weighted cases

TABLE 8.7 ANAEMIA AMONG POPULATION AGED 20 YEARS AND ABOVE

Percentage of population (age 20 years and above) classified as having iron-deficiency (anaemia) by degree of anaemia and selected background characteristics, Haryana, 2012-13.

Background characteristics	Male				Female				Total			
	Mild anaemia (10.0-10.9g/dl)	Moderate anaemia (7.0-9-9 g/dl)	Severe anaemia (< 7g/dl)	Any anaemia <11.0 g/dl	Mild anaemia (10.0-10.9g/dl)	Moderate anaemia (7.0-9-9g/dl)	Severe anaemia (< 7g/dl)	Any anaemia <11.0 g/dl	Mild anaemia (10.0-10.9 g/dl)	Moderate anaemia (7.0-9-9g/dl)	Severe anaemia (< 7g/dl)	Any anaemia <11.0 g/dl
**Age group**												
20 - 29	14.9	27.4	5.3	47.5	17.2	34.9	6.6	58.7	16.1	31.4	6.0	53.5
30 - 39	15.6	26.7	5.2	47.5	17.8	33.1	6.4	57.3	16.8	30.2	5.9	52.8
40– 49	15.9	27.0	4.6	47.5	17.6	33.0	5.7	56.3	16.8	30.2	5.2	52.1
50 +	15.7	28.1	4.9	48.6	17.9	31.7	4.9	54.5	16.9	30.1	4.9	51.9
**Residence**												
Rural	15.3	27.7	4.9	47.9	17.6	33.6	5.8	57.0	16.5	30.9	5.4	52.9
Urban	15.8	26.8	5.2	47.8	17.7	32.4	5.9	55.9	16.8	29.8	5.6	52.1
**Education**												
Non-literate[a]	15.5	28.1	4.9	48.5	17.6	33.2	5.7	56.5	17.0	31.7	5.5	54.2
Less than 5 years	16.1	28.0	5.5	49.6	19.8	33.1	6.1	59.0	17.8	30.4	5.8	54.0
5-9 years	15.6	27.1	5.3	48.0	17.6	34.1	6.2	58.0	16.6	30.7	5.8	53.1
10 or more years	15.3	27.2	4.9	47.4	17.4	32.5	5.8	55.7	16.2	29.4	5.3	51.0
**Religion**												
Hindu	15.4	27.3	5.1	47.8	17.5	33.3	5.8	56.6	16.5	30.6	5.5	52.6
Muslim	15.5	32.4	6.2	54.1	18.0	36.5	9.4	64.0	16.9	34.7	8.0	59.5
Christian	18.2	26.8	5.9	50.8	14.2	30.2	5.1	49.5	16.1	28.5	5.5	50.2
Sikh	15.4	23.1	3.1	41.5	19.5	27.6	4.0	51.1	17.6	25.6	3.6	46.8
Jain	14.2	26.2	2.2	42.6	28.1	24.1	5.4	57.6	21.3	25.1	3.8	50.2
Others	36.3	25.1	0.0	61.4	28.5	17.3	0.0	45.8	30.5	20.3	0.0	50.8
**Castes/Tribes**												
Scheduled Caste	16.2	27.3	5.4	48.9	17.5	33.7	6.6	57.8	16.9	30.7	6.0	53.7
Scheduled Tribes	16.9	28.7	4.4	50.0	17.9	34.5	5.5	57.9	17.4	31.8	5.0	54.2
Other Backward Classes	15.3	28.2	5.4	48.9	17.1	34.5	6.1	57.7	16.3	31.6	5.8	53.7
Others	14.8	26.7	4.6	46.1	18.0	31.7	5.2	54.9	16.5	29.4	4.9	50.9
**Haryana**	15.5	27.4	5.0	47.8	17.6	33.2	5.9	56.6	16.6	30.5	5.5	52.6

[a] Literate but did not attend school, are also included

TABLE 8.8 ANAEMIA AMONG POPULATION CHILDREN, ADOLESECENTS AGED 20 YEARS AND ABOVE

Percentage of children aged 6-59 months, adolescents aged 6-19 year and population aged 20 years and above having any and severe anaemia by districts, Haryana, 2012-13.

	Children		Adolescents		Aged 20 years and above	
District	Any anaemia <11.0 g/dl	Severe anaemia (< 7g/dl)	Any anaemia <11.0 g/dl	Severe anaemia (< 7g/dl)	Any anaemia <11.0 g/dl	Severe anaemia (< 7g/dl)
Panchkula	45.9	3.3	40.7	1.2	38.4	1.2
Ambala	58.2	5.3	51.3	3.0	48.9	2.4
Yamunanagar	54.8	6.3	52.5	4.0	50.6	3.8
Kurukshetra	59.7	7.2	52.6	7.1	54.3	7.8
Kaithal	52.3	2.7	46.9	1.7	45.0	2.1
Karnal	59.4	4.4	58.1	5.9	56.1	5.9
Panipat	59.8	5.8	49.3	3.0	47.4	3.4
Sonipat	69.4	14.0	65.7	9.8	60.8	10.0
Jind	60.6	5.2	57.4	5.2	53.0	4.6
Fatehabad	65.1	3.9	57.3	3.9	52.9	3.5
Sirsa	67.6	6.8	50.4	3.5	45.6	3.2
Hisar	66.0	11.1	49.9	3.7	45.0	3.0
Bhiwani	66.4	8.6	60.1	3.9	58.7	3.3
Rohatak	59.2	14.9	44.8	4.6	40.6	4.0
Jhajjar	66.9	10.7	52.7	5.1	50.3	5.0
Mahendragarh	63.0	11.3	56.1	6.3	53.5	5.1
Rewari	67.5	12.9	66.0	12.4	67.3	12.3
Gurgaon	56.1	6.9	59.6	6.0	58.6	7.7
Faridabad	68.6	13.0	66.1	12.1	68.6	10.7
Mewat	69.5	14.7	70.0	15.7	67.2	15.0
Palwal	67.4	11.8	60.0	8.1	53.5	6.8
**Haryana**	62.8	9.0	55.8	6.1	52.6	5.5

TABLE 8.9 ANAEMIA AMONG PREGNANT WOMEN

Percentage of pregnant women (age 15-49 years) classified as having iron-deficiency (anaemia) by degree of anaemia and by selected background characteristics and residence, Haryana, 2012-13.

	Anaemia status by haemoglobin level				
Background characteristics	Mild anaemia (10.0-10.9 g/dl)	Moderate anaemia (7.0-9-9 g/dl)	Severe anaemia (< 7g/dl)	Any anaemia <11.0 g/dl	Total number of pregnant women**
**Age group**					
15-19	12.2	38.7	7.2	58.2	161
20-29	17.2	35.9	6.9	60.0	2,180
30–39	16.0	34.7	8.7	59.4	451
40-49	14.1	31.9	7.0	53.1	81
**Residence**					
Rural	16.5	36.1	7.8	60.4	1,793
Urban	17.1	34.8	5.9	57.8	1,080
**Woman's Education**					
Non-literate[a]	17.3	34.8	10.1	62.1	606
Less than 5 years	15.2	46.6	6.9	68.8	73
5-9 years	16.3	37.2	7.4	60.9	886
10 or more years	16.8	34.4	5.6	56.9	1,308
**Religion**					
Hindu	16.8	35.6	6.8	59.2	2,592
Muslim	12.6	40.6	10.9	64.2	205
Christian	--	--	--	--	3
Sikh	23.1	24.1	9.8	57.0	69
Jain	--	--	--	--	2
Others	--	--	--	--	1
**Castes/Tribes**					
Scheduled Caste	16.3	34.2	8.7	59.2	940
Scheduled Tribes	13.6	44.4	4.9	62.8	134
Other Backward Classes	16.7	38.7	7.6	63.0	877
Others	17.6	33.0	5.5	56.1	922
**Haryana**	16.7	35.7	7.2	59.6	2,873

[a] Literate but did not attend school, are also included. -- Percentage not shown for less than 10 cases. ** Unweighted cases .

TABLE 8.10 PREVALENCE OF DIABETES

Percentage of men (age 18 years and above) classified as having Sugar by selected background characteristics and residence, Haryana, 2012-13.

Background characteristics	Any type of blood sugar level			Total number of men Tested**
	Below 140	Mild (140-160)	Moderate/High (>160)	
**Age group**				
18 - 29	92.4	6.1	1.5	12,274
30 - 39	87.4	8.2	4.4	7,580
40 - 49	84.5	9.1	6.4	6,042
50 - 59	78.9	11.0	10.2	4,259
60 +	74.6	13.1	12.3	5,953
**Residence**				
Rural	86.1	8.9	5.0	20,735
Urban	84.5	8.5	7.0	15,373
**Education**				
Non-literate[a]	83.9	9.9	6.2	6,878
Less than 5 years	81.4	10.9	7.7	951
5-9 years	85.0	9.2	5.8	9,037
10 or more years	86.6	8.0	5.4	19,242
**Religion**				
Hindu	85.6	8.6	5.8	33,283
Muslim	86.3	10.0	3.7	1,368
Christian	89.4	5.3	5.3	61
Sikh	81.9	10.8	7.3	1,301
Jain	76.5	11.7	11.8	46
Others	95.5	4.5	0.0	21
**Castes/Tribes**				
Scheduled Caste	86.5	8.5	5.0	9,954
Scheduled Tribes	85.5	8.6	5.9	1,941
Other Backward Classes	85.3	9.3	5.4	10,012
Others	84.9	8.6	6.5	14,201
**Haryana**	85.5	8.8	5.7	36,108

[a] Literate but did not attend school, are also included. * *Unweighted cases

TABLE 8.11 PREVALENCE OF DIABETES

Percentage of men aged 18 years and above classified with level of any type of blood Sugar by districts, Haryana, 2012-13.

District	Any type of blood sugar level			Total number of men Tested**
	Below (140)	Mild (140-160)	Moderate/High (>160)	
Panchkula	89.4	6.7	4.0	1,577
Ambala	89.4	6.4	4.2	2,039
Yamunanagar	89.5	5.4	5.0	1,679
Kurukshetra	82.2	12.6	5.2	2,160
Kaithal	88.3	7.3	4.4	2,576
Karnal	88.6	8.3	3.2	2,374
Panipat	88.1	6.2	5.6	1,805
Sonipat	85.3	6.6	8.2	1,547
Jind	86.5	9.8	3.7	2,290
Fatehabad	82.7	9.0	8.3	1,096
Sirsa	81.8	9.4	8.8	2,050
Hisar	82.6	9.5	7.8	1,858
Bhiwani	82.1	9.0	8.9	1,362
Rohatak	84.2	7.5	8.3	1,379
Jhajjar	84.7	8.9	6.4	1,912
Mahendragarh	80.3	11.0	8.7	1,218
Rewari	81.9	13.7	4.4	1,396
Gurgaon	89.3	6.4	4.3	1,873
Faridabad	85.1	9.3	5.6	1,480
Mewat	83.1	12.0	4.9	1,308
Palwal	80.0	11.3	8.7	1,129
**Haryana**	85.5	8.8	5.7	36,108

** Unweighted cases

TABLE 8.12 PREVALENCE OF DIABETES

Percentage of Women (age 18 years and above) classified as having Sugar by selected background characteristics and residence, Haryana, 2012-13.

Background characteristics	Any type of blood sugar level			Total number of women Tested**
	Below 140	Mild (140-160)	Moderate/High (>160)	
**Age group**				
18 - 29	93.3	5.0	1.8	13,699
30 - 39	89.4	7.3	3.4	9,237
40 - 49	84.0	9.4	6.6	6,612
50 - 59	78.6	11.7	9.7	6,048
60 +	73.2	13.5	13.3	6,471
**Residence**				
Rural	86.6	8.2	5.1	24,795
Urban	84.1	8.9	7.0	17,272
**Education**				
Non-literate[a]	82.1	10.3	7.6	16,796
Less than 5 years	83.7	8.6	7.7	863
5-9 years	87.1	7.7	5.3	9,544
10 or more years	89.4	6.7	3.9	14,864
**Religion**				
Hindu	85.8	8.5	5.7	38,713
Muslim	89.1	7.6	3.3	1,615
Christian	77.2	10.4	12.3	68
Sikh	82.5	7.6	9.9	1,578
Jain	81.8	8.6	9.6	47
Others	(64.6)	(17.3)	(18.1)	11
**Castes/Tribes**				
Scheduled Caste	86.9	8.2	4.9	11,346
Scheduled Tribes	86.6	8.3	5.1	2,238
Other Backward Classes	86.1	8.2	5.7	11,602
Others	84.5	8.8	6.7	16,881
**Haryana**	85.7	8.8	5.8	42,067

[a] Literate but did not attend school, are also included. ( ) Based on 10-20 unweighted cases. ** Unweighted cases

TABLE 8.13 PREVALENCE OF DIABETES

Percentage of women aged 18 years and above classified with level of any type of blood Sugar by districts, Haryana, 2012-13.

District	Any type of blood sugar level			Total number of women Tested**
	Below 140	Mild (140-160)	Moderate/High (>160)	
Panchkula	88.9	6.2	4.9	1,657
Ambala	88.5	5.6	5.9	2,181
Yamunanagar	88.9	5.5	5.6	1,875
Kurukshetra	83.8	10.7	5.5	2,632
Kaithal	89.1	6.5	4.4	2,682
Karnal	88.9	6.4	4.7	2,701
Panipat	88.0	6.4	5.6	1,878
Sonipat	86.0	7.0	7.0	1,938
Jind	85.6	10.2	4.2	2,457
Fatehabad	83.5	8.2	8.4	1,506
Sirsa	81.5	10.7	7.8	2,549
Hisar	82.8	10.4	6.8	2,276
Bhiwani	83.5	9.5	7.0	1,810
Rohatak	84.5	8.1	7.3	1,827
Jhajjar	83.9	9.5	6.7	2,282
Mahendragarh	83.0	10.2	6.8	1,864
Rewari	86.5	9.9	3.6	1,533
Gurgaon	85.5	9.4	5.0	1,681
Faridabad	86.7	8.0	5.3	1,667
Mewat	86.9	8.8	4.3	1,552
Palwal	80.6	11.4	8.0	1,519
**Haryana**	85.7	8.8	5.8	42,067

** Unweighted cases

TABLE 8.14 BLOOD PRESSURE

Percentage of men (age 18 years and above) classified as having Blood Pressure by selected background characteristics, Haryana, 2012-13.

Background characteristics	Status of Blood Pressure						Number of men blood pressure measured**	Number of men Covered**
	1	2	3	4	5	6		
**Age group**								
18- 29	68.9	13.2	15.2	1.9	0.5	0.3	12,522	12,696
30 - 39	57.6	16.6	20.9	3.4	0.8	0.7	7,702	7,804
40 - 49	51.7	16.2	24.1	5.3	1.6	1.1	6,134	6,213
50 - 59	46.4	16.0	27.3	6.2	2.2	1.9	4,318	4,372
60 +	45.5	14.3	26.8	8.4	3.2	1.7	6,034	6,096
**Residence**								
Rural	58.7	14.8	20.5	4.0	1.3	0.9	21,106	21,384
Urban	54.9	15.1	22.4	5.0	1.6	1.0	15,604	15,797
**Education**								
Non-literate[a]	54.4	14.5	23.2	4.8	1.7	1.2	6,979	7,054
Less than 5 years	57.9	13.8	22.5	4.0	1.1	0.6	969	977
5-9 years	57.7	15.4	20.1	4.4	1.6	0.9	9,174	9,301
10 or more years	58.2	14.9	20.8	4.2	1.2	0.8	19,588	19,849
**Religion**								
Hindu	57.5	14.9	21.1	4.3	1.4	0.9	33,829	34,264
Muslim	59.2	16.2	20.9	2.3	0.9	0.4	1,395	1,417
Christian	47.4	12.7	18.0	15.8	4.6	1.5	62	62
Sikh	51.9	14.7	23.5	6.6	2.3	1.1	1,319	1,332
Jain	45.4	14.2	27.7	9.4	1.7	1.7	52	53
Others	72.6	11.0	16.4	0.0	0.0	0.0	21	21
**Castes/Tribes**								
Scheduled Caste	60.3	14.4	19.7	3.3	1.2	1.0	10,090	10,222
Scheduled Tribes	55.9	15.0	21.7	4.8	1.6	1.0	1,967	2,007
Other Backward Classes	57.0	15.1	21.4	4.4	1.3	0.8	10,176	10,284
Others	55.5	15.0	22.0	5.0	1.6	1.0	14,477	14,668
**Haryana**	57.3	14.9	21.2	4.3	1.4	0.9	36,710	37,181

[a] Literate but did not attend school, are also included.** unweighted cases.

Average Systolic	Average Diastolic					
	≤84	85-89	90-99	100-109	110-119	≥120
≤ 129	1	2	3	4	5	6
130-139	2	2	3	4	5	6
140-159	3	3	3	4	5	6
160-179	4	4	4	4	5	6
180-209	5	5	5	5	5	6
≥ 210	6	6	6	6	6	6

TABLE 8.15 BLOOD PRESSURE

Percentage of men (age 18 years and above) classified as having Blood Pressure by districts, Haryana, 2012-13.

	Status of Blood Pressure						Number of men	Number of men
District	1	2	3	4	5	6	blood pressure measured**	aged 18 years & above**
Panchkula	55.3	13.5	22.2	5.1	2.0	2.0	1,602	1,648
Ambala	53.3	17.0	20.9	5.1	2.0	1.9	2,079	2,121
Yamunanagar	58.0	15.4	20.2	4.3	1.4	0.7	1,777	1,855
Kurukshetra	54.0	16.5	26.0	2.4	0.8	0.3	2,172	2,201
Kaithal	59.1	12.6	20.5	4.8	1.8	1.2	2,589	2,619
Karnal	57.0	15.7	23.9	2.2	0.7	0.5	2,408	2,417
Panipat	59.3	16.9	20.9	1.8	0.8	0.2	1,838	1,872
Sonipat	59.7	12.7	21.8	3.8	1.3	.6	1,671	1,716
Jind	54.0	12.9	24.3	5.9	1.8	1.1	2,297	2,315
Fatehabad	66.2	11.9	16.0	4.7	1.0	0.2	1,139	1,147
Sirsa	63.5	11.9	18.4	3.9	1.2	1.2	2,080	2,089
Hisar	70.6	10.4	13.0	4.0	1.2	0.8	1,881	1,890
Bhiwani	58.0	11.8	19.6	7.0	2.0	1.5	1,376	1,379
Rohatak	52.6	19.7	20.8	4.5	1.7	0.8	1,426	1,447
Jhajjar	60.1	16.5	16.2	4.6	1.5	1.1	1,926	1,970
Mahendragarh	46.6	14.6	27.6	7.0	2.5	1.7	1,229	1,232
Rewari	54.2	18.4	21.8	4.0	1.0	0.7	1,397	1,400
Gurgaon	52.6	18.1	22.6	4.8	1.0	0.9	1,886	1,902
Faridabad	48.1	16.4	24.3	8.1	2.3	0.8	1,483	1,494
Mewat	52.5	17.2	25.5	3.8	0.6	0.4	1,315	1,317
Palwal	62.2	13.2	19.2	3.0	1.5	0.8	1,139	1,150
**Haryana**	57.3	14.9	21.2	4.3	1.4	0.9	36,710	37,181

** Unweighted cases

**Average Systolic**	**Average Diastolic**					
	≤84	85-89	90-99	100-109	110-119	≥120
≤ 129	1	2	3	4	5	6
130-139	2	2	3	4	5	6
140-159	3	3	3	4	5	6
160-179	4	4	4	4	5	6
180-209	5	5	5	5	5	6
≥ 210	6	6	6	6	6	6

TABLE 8.16 BLOOD PRESSURE

Percentage of women (age 18 years and above) classified as having Blood Pressure by selected background characteristics, Haryana, 2012-13.

Background characteristics	Status of Blood Pressure 1	2	3	4	5	6	Number of women blood pressure measured**	Number of women aged 18 year & above**
**Age group**								
18- 29	82.1	8.1	8.1	1.2	0.3	0.2	14,013	14,156
30 - 39	72.9	11.3	12.7	2.1	0.5	0.4	9,374	9,450
40 - 49	63.8	12.8	17.3	3.9	1.2	1.0	6,727	6,764
50 - 59	55.0	14.7	20.5	5.8	2.5	1.5	6,141	6,178
60 +	45.5	12.3	25.8	9.1	4.4	2.8	6,591	6,646
**Residence**								
Rural	69.0	11.0	14.4	3.4	1.3	0.9	25,234	25,446
Urban	66.1	11.2	15.9	4.1	1.6	1.1	17,612	17,748
**Education**								
Non-literate[a]	60.8	12.3	18.3	5.0	2.2	1.4	17,063	17,191
Less than 5 years	70.2	9.8	12.7	4.8	1.0	1.5	881	889
5-9 years	71.1	10.5	13.7	2.9	1.0	0.8	9,712	9,778
10 or more years	74.3	10.1	11.9	2.5	0.8	0.5	15,190	15,336
**Religion**								
Hindu	68.0	11.1	14.9	3.6	1.4	0.9	39,437	39,750
Muslim	69.5	11.0	15.5	2.4	0.7	1.0	1,644	1,667
Christian	60.4	9.1	8.0	16.7	2.9	2.9	68	68
Sikh	65.9	11.0	14.1	4.8	2.3	1.8	1,598	1,608
Jain	48.6	18.5	20.5	8.8	1.8	1.8	50	50
Others	(67.3)	(10.4)	(22.3)	(0.0)	(0.0)	(0.0)	11	11
**Castes/Tribes**								
Scheduled Caste	70.6	11.1	13.6	2.6	1.1	0.8	11,554	11,649
Scheduled Tribes	66.1	12.7	14.3	4.1	1.9	0.9	2,280	2,304
Other Backward Classes	67.6	11.0	15.0	4.0	1.2	1.1	11,774	11,861
Others	66.7	10.8	15.8	4.1	1.7	1.0	17,238	17,380
**Haryana**	68.0	11.1	14.9	3.6	1.4	1.0	42,846	43,194

[a] Literate but did not attend school, are also included. () Based on 10-20 unweighted cases. ** Unweighted cases.

**Average Systolic**	**Average Diastolic**					
	≤84	85-89	90-99	100-109	110-119	≥120
≤ 129	1	2	3	4	5	6
130-139	2	2	3	4	5	6
140-159	3	3	3	4	5	6
160-179	4	4	4	4	5	6
180-209	5	5	5	5	5	6
≥ 210	6	6	6	6	6	6

TABLE 8.17 BLOOD PRESSURE

Percentage of women (age 18 years and above) classified as having Blood Pressure by districts, Haryana, 2012-13.

	Status of Blood Pressure						Number of women blood pressure measured**	Number of women aged 18 years & above**
District	1	2	3	4	5	6		
Panchkula	65.9	11.7	15.6	4.3	1.0	1.5	1,688	1,706
Ambala	62.7	11.8	18.1	4.0	2.0	1.5	2,232	2,268
Yamunanagar	65.3	11.6	15.6	4.6	1.7	1.2	1,974	2,026
Kurukshetra	63.7	16.1	16.9	2.3	0.7	0.4	2,634	2,649
Kaithal	68.0	10.2	13.7	4.7	1.6	1.8	2,705	2,735
Karnal	70.0	13.1	13.4	2.0	0.8	0.7	2,745	2,757
Panipat	72.2	11.3	13.1	1.7	1.3	0.3	1,924	1,952
Sonipat	69.1	10.5	15.3	3.2	1.1	0.8	2,072	2,108
Jind	63.5	11.1	18.8	4.3	1.7	0.7	2,481	2,491
Fatehabad	76.3	6.5	11.5	3.2	1.2	1.2	1,557	1,565
Sirsa	74.6	7.6	12.2	3.0	1.5	1.0	2,592	2,600
Hisar	78.0	6.4	10.2	3.0	1.4	1.0	2,338	2,346
Bhiwani	70.6	8.1	12.7	4.8	2.4	1.3	1,834	1,835
Rohatak	64.6	13.7	15.7	4.6	1.0	0.4	1,879	1,902
Jhajjar	70.6	9.5	12.4	4.3	1.8	1.4	2,307	2,323
Mahendragarh	62.0	11.1	16.4	6.2	2.7	1.7	1,879	1,887
Rewari	64.9	14.6	16.2	3.1	0.7	0.5	1,539	1,546
Gurgaon	68.1	10.1	15.1	4.5	1.5	0.7	1,701	1,709
Faridabad	59.1	12.8	19.2	6.0	1.9	1.0	1,668	1,676
Mewat	62.4	13.8	20.1	2.6	0.8	0.3	1,558	1,565
Palwal	70.1	11.2	15.1	2.0	0.8	0.8	1,539	1,548
**Haryana**	68.0	11.1	14.9	3.6	1.4	1.0	42,846	43,194

** Unweighted cases.

TABLE 8.18 PRESENCE OF IODIZED SALT IN HOUSEHOLD

Percent distribution of household with salt tested for iodine content, by level of iodine in salt (Parts Per Million) according to background characteristics, Haryana, 2012-13.

	Iodine content of salt				
Background characteristics	None 0 ppm	Inadequate (< 15 ppm)	Adequate (15 + ppm)	Not Tested*	Number of Households**
**Age of head of Household**					
< 30	1.2	33.0	59.7	6.1	2,421
30 - 44	1.9	31.2	56.4	10.5	10,964
45 - 59	4.5	23.5	55.8	16.2	11,354
60 +	8.1	14.2	55.7	22.0	9,033
**Residence**					
Rural	5.0	22.2	56.0	16.8	19,216
Urban	3.5	27.5	56.7	12.4	14,556
**Education of head of household**					
Non-literate[a]	5.7	19.1	56.2	19.0	10,633
Less than 5 years	3.6	22.6	56.8	16.9	1,008
5-9 years	4.0	23.9	57.0	15.0	8,706
10 or more years	3.6	28.8	55.7	11.9	13,425
**Religion**					
Hindu	4.1	24.5	56.5	14.8	31,089
Muslim	9.7	17.8	48.8	23.7	1,456
Christian	4.8	28.6	45.2	21.4	50
Sikh	4.8	22.5	59.2	13.5	1,118
Jain	5.6	27.8	52.8	13.9	40
Others	5.9	17.6	52.9	23.5	17
**Castes/Tribes**					
Scheduled Caste	3.9	22.2	57.9	16.1	9,639
Scheduled Tribes	3.9	23.8	57.0	15.3	1,785
Other Backward Classes	4.6	23.7	56.2	15.5	9,294
Others	4.8	26.1	54.9	14.2	13,054
**Haryana**	4.4	24.2	56.2	15.2	33,772

Note; PPM: parts per million. * includes salt not at home, salt not tested, refused and missing cases.
[a] Literate but did not attend school, are also included. ** unweighted cases

TABLE 8.19 PRESENCE OF IODIZED SALT IN HOUSEHOLD

Percent distribution of household with salt tested for iodine content, by level of iodine in salt (Parts Per Million) by districts, Haryana, 2012-13.

	Iodine content of salt				
District	None 0 ppm	Inadequate (< 15 ppm)	Adequate (15 + ppm)	Not Tested/Missing*	Number of Households**
Panchkula	2.3	31.0	55.3	11.4	1,320
Ambala	2.0	31.8	54.7	11.5	1,621
Yamunanagar	4.5	29.0	51.1	15.4	1,607
Kurukshetra	3.1	17.5	69.5	9.9	1,893
Kaithal	3.6	26.1	54.3	15.9	1,899
Karnal	5.7	22.5	55.8	16.0	1,904
Panipat	5.7	24.9	48.1	21.3	1,618
Sonipat	6.0	27.2	49.9	16.9	1,792
Jind	1.3	12.2	78.8	7.7	1,900
Fatehabad	5.3	25.1	54.7	14.8	1,266
Sirsa	6.9	24.6	50.7	17.8	1,873
Hisar	4.7	26.6	51.8	16.9	1,893
Bhiwani	5.2	24.3	54.0	16.5	1,345
Rohatak	5.1	25.8	52.3	16.8	1,582
Jhajjar	4.8	28.6	51.0	15.7	1,873
Mahendragarh	4.0	24.4	58.8	12.8	1,361
Rewari	1.3	19.5	66.0	13.3	1,346
Gurgaon	3.2	34.3	52.5	10.0	1,638
Faridabad	0.6	24.4	68.8	6.2	1,361
Mewat	6.8	16.1	54.0	23.1	1,351
Palwal	8.6	18.3	48.6	24.5	1,329
**Haryana**	4.3	24.5	56.3	14.9	33,772

Note; PPM: parts per million. * includes salt not at home, salt not tested, refused and missing cases. ** unweighted cases

HEALTH FACILITY

TABLE 9.1: AVERAGE POPULATION COVERED BY HEALTH FACILITY BY DISTRICTS, HARYANA, 2012-13.

District	Average population covered by		
	Sub-Health Centre	PHC	CHC
Panchkula	12,712	40,369	1,96,428
Ambala	9,875	44,043	1,93,163
Yamunanagar	8,118	45,831	1,43,860
Kurukshetra	6,449	32,507	1,38,546
Kaithal	6,739	62,321	1,49,882
Karnal	7,676	42,480	1,85,881
Panipath	9,426	66,416	2,95,023
Sonipath	8,095	72,787	1,56,949
Jind	7,106	38,589	1,60,549
Fatehabad	8,444	38,593	1,50,865
Sirsa	7,968	35,504	1,38,465
Hisar	9,582	32,059	1,64,894
Bhiwani	5,989	41,595	1,41,079
Rohtak	6,235	29,230	1,38,500
Jhajjar	6,720	27,423	84,499
Mahendragarh	6,614	40,142	1,16,698
Rewari	7,420	39,646	1,19,915
Gurgaon	8,479	71,888	26,396
Faridabad	9,404	67,171	2,17,069
Mewat	14,660	97,058	2,64,387
Palwal	11,623	72,268	2,93,884
**Haryana**	8,239	46,503	1,56,701

PHC= Primary Health Centre; CHC= Community Health Centre.

TABLE 9.2: STATUS OF INFRASTRUCTURE AT SUB-HEALTH CENTRE FUNCTIONING IN GOVERNMENT BUILDING BY DISTRICTS, HARYANA, 2012-13.

District	Number of Sub-Health Centre					Number of Sub-Health Centre with govt. Building	Total number of Sub-Health Centre
	Regular Electricity	Water#	Toilet	Labor room	Labor room in current use[1]		
Panchkula	53.9	100.0	92.3	69.2	100.0	13	19
Ambala	57.9	100.0	89.5	63.2	66.7	19	29
Yamunanagar	40.0	100.0	100.0	50.0	70.0	20	31
Kurukshetra	0.0	100.0	92.9	50.0	57.1	14	39
Kaithal	44.4	88.9	88.9	61.1	90.9	18	50
Karnal	4.4	95.7	100.0	34.8	100.0	23	43
Panipath	0.0	60.0	66.7	33.3	60.0	15	30
Sonipath	6.9	93.1	89.7	55.2	93.8	29	38
Jind	4.0	76.0	96.0	32.0	100.0	25	41
Fatehabad	26.7	100.0	93.3	60.0	44.4	15	24
Sirsa	96.3	100.0	100.0	96.3	96.2	27	43
Hisar	20.0	93.3	100.0	0.0	NA	15	43
Bhiwani	12.5	93.8	93.8	6.3	100.0	16	28
Rohtak	26.9	100.0	100.0	42.3	90.9	26	33
Jhajjar	8.7	95.7	100.0	21.7	40.0	23	43
Mahendragarh	43.8	87.5	68.8	0.0	NA	16	25
Rewari	14.3	85.7	92.9	57.1	100.0	14	22
Gurgaon	4.8	100.0	100.0	9.5	0.0	21	24
Faridabad	4.8	100.0	100.0	0.0	NA	21	21
Mewat	10.0	50.0	70.0	40.0	50.0	10	23
Palwal	0.0	87.5	50.0	37.5	100.0	08	24
**Haryana**	24.2	92.3	92.3	40.0	82.5	388	673

Includes piped, bore well, well hand pump and other source of water. [1] Percentage calculated from number of labor room available.

TABLE 9.3: PERCENTAGE OF SUB-HEALTH-CENTRES HAVING DIFFERENT ACTIVITIES BY DISTRICTS, HARYANA, 2012-13.

District	Citizen's Charter displayed	VHSC Facilitated*	Untied Fund Received	Total number of Sub-Health Centre
Panchkula	84.2	88.2	68.4	19
Ambala	85.7	96.4	92.9	29
Yamunanagar	77.4	100.0	93.6	31
Kurukshetra	100.0	97.4	97.4	39
Kaithal	66.0	91.7	86.0	50
Karnal	97.7	100.0	74.4	43
Panipath	70.0	80.0	90.0	30
Sonipath	81.6	97.3	94.7	38
Jind	75.6	97.5	80.5	41
Fatehabad	75.0	59.1	95.8	24
Sirsa	95.4	55.8	95.4	43
Hisar	95.4	93.0	97.7	43
Bhiwani	96.4	96.4	100.0	28
Rohtak	87.9	100.0	75.8	33
Jhajjar	90.7	90.2	83.7	43
Mahendragarh	92.0	80.0	96.0	25
Rewari	95.5	81.8	90.9	22
Gurgaon	95.8	100.0	83.3	24
Faridabad	100.0	100.0	100.0	21
Mewat	39.1	65.2	82.6	23
Palwal	79.2	90.9	79.2	24
**Haryana**	85.1	89.2	88.5	673

VHSC= Village Health and Sanitation Committee.
* Based on availability of VHSC.

TABLE 9.4: AVAILABLE HUMAN RESOURCES AT SUB-HEALTH CENTRES BY DISTRICTS, HARYANA, 2012-13.

District	Human resources Status of Sub-Health Centre			Total number of SHCs
	ANM	MHW	Additional ANM	
Panchkula	94.7	42.1	94.4	19
Ambala	89.3	10.7	72.0	29
Yamunanagar	77.4	25.8	20.8	31
Kurukshetra	69.2	18.0	70.4	39
Kaithal	84.0	56.0	73.8	50
Karnal	86.1	48.8	56.8	43
Panipath	100.0	73.3	90.0	30
Sonipath	100.0	92.1	97.4	38
Jind	90.2	75.6	86.5	41
Fatehabad	87.5	58.3	85.7	24
Sirsa	88.4	27.9	89.5	43
Hisar	88.4	58.1	84.2	43
Bhiwani	96.4	71.4	96.3	28
Rohtak	97.0	100.0	96.9	33
Jhajjar	100.0	93.0	90.7	43
Mahendragarh	96.0	72.0	75.0	25
Rewari	100.0	59.1	100.0	22
Gurgaon	100.0	87.5	100.0	24
Faridabad	100.0	100.0	100.0	21
Mewat	95.7	30.4	100.0	23
Palwal	79.2	16.7	79.0	24
**Haryana**	90.6	58.2	83.5	673

ANM= Auxiliary Nurse Midwife. MHW= Male health Worker.

TABLE 9.5: AVAILABLE HUMAN RESOURCES AT PRIMARY HEALTH CENTRES BY DISTRICTS, HARYANA, 2012-13.

District	Human resources Status of PHC				Total number of PHCs
	Medical officer	Lady Medical Officer**	AYUSH Doctor**	Pharmacist	
Panchkula	71.4	60.0	60.0	57.1	07
Ambala	90.0	55.6	33.3	80.0	10
Yamunanagar	90.0	33.3	11.1	40.0	10
Kurukshetra	100.0	42.9	14.3	100.0	14
Kaithal	61.5	62.5	25.0	100.0	13
Karnal	76.5	23.1	15.4	76.5	17
Panipath	72.7	37.5	12.5	100.0	11
Sonipath	86.7	46.2	0.0	100.0	15
Jind	41.2	14.3	28.6	100.0	17
Fatehabad	60.0	33.3	0.0	90.0	10
Sirsa	100.0	25.0	0.0	66.7	12
Hisar	84.2	18.8	12.5	94.7	19
Bhiwani	64.3	0.0	0.0	92.9	14
Rohtak	92.9	46.2	30.8	100.0	14
Jhajjar	93.3	35.7	7.1	86.7	15
Mahendragarh	90.0	11.1	0.0	50.0	10
Rewari	100.0	55.6	11.1	77.8	09
Gurgaon	88.9	50.0	62.5	77.8	09
Faridabad	100.0	66.7	50.0	66.7	06
Mewat	100.0	0.0	50.0	16.7	06
Palwal	62.5	0.0	60.0	37.5	08
**Haryana**	80.9	34.2	19.1	81.7	246

** Out of total medical officer

TABLE 9. 6: AVAILABLE INFRASTRUCTURE AT PRIMARY HEALTH CENTRES BY DISTRICTS, HARYANA, 2012-13.

District	Percentage of PHCs having					Total number of PHCs
	Residential Quarter for MO	Functioning PHC 24 hours	At least 4 beds	Regular power supply	Having functional vehicle	
Panchkula	42.9	57.1	83.3	42.9	42.9	07
Ambala	70.0	70.0	80.0	40.0	70.0	10
Yamunanagar	40.0	30.0	55.6	60.0	0.0	10
Kurukshetra	35.7	100.0	83.3	7.1	14.3	14
Kaithal	69.2	61.5	83.3	53.9	15.4	13
Karnal	64.7	100.0	100.0	5.9	58.8	17
Panipath	9.1	100.0	27.3	0.0	36.4	11
Sonipath	26.7	46.7	60.0	0.0	6.7	15
Jind	35.3	100.0	41.2	5.9	35.3	17
Fatehabad	60.0	80.0	90.0	80.0	40.0	10
Sirsa	83.3	83.3	83.3	100.0	25.0	12
Hisar	73.7	89.5	88.2	89.5	15.8	19
Bhiwani	71.4	100.0	92.9	92.9	7.1	14
Rohtak	50.0	85.7	78.6	14.3	57.1	14
Jhajjar	6.7	73.3	84.6	46.7	26.7	15
Mahendragarh	90.0	20.0	80.0	100.0	0.0	10
Rewari	22.2	100.0	77.8	0.0	11.1	09
Gurgaon	0.0	77.8	100.0	55.6	44.4	09
Faridabad	33.3	100.0	83.3	33.3	100.0	06
Mewat	66.7	83.3	66.7	0.0	33.3	06
Palwal	37.5	75.0	50.0	0.0	75.0	08
**Haryana**	47.9	79.3	75.7	40.2	31.3	246

MO= Medical Officer.

TABLE 9.7: SPECIFIC HEALTH FACILITIES AVAILABLE AT PRIMARY HEALTH CENTRES BY DISTRICTS, HARYANA , 2012-13.

District	Percentage of PHCs having			Total number of PHCs
	New born care services*	Referral services for delivery**	Conducted at least 10 deliveries	
Panchkula	100.0	50.0	100.0	07
Ambala	85.7	100.0	85.7	10
Yamunanagar	100.0	66.7	66.7	10
Kurukshetra	92.9	100.0	64.3	14
Kaithal	92.3	62.5	76.9	13
Karnal	100.0	70.6	88.2	17
Panipath	100.0	90.9	54.6	11
Sonipath	85.7	71.4	75.0	15
Jind	100.0	82.4	68.8	17
Fatehabad	88.9	87.5	75.0	10
Sirsa	100.0	60.0	100.0	12
Hisar	88.2	17.7	61.1	19
Bhiwani	78.6	21.4	71.4	14
Rohtak	66.7	75.0	66.7	14
Jhajjar	81.8	63.6	77.8	15
Mahendragarh	75.0	100.0	25.0	10
Rewari	100.0	66.7	66.7	09
Gurgaon	100.0	71.4	100.0	09
Faridabad	80.0	0.0	80.0	06
Mewat	100.0	80.0	100.0	06
Palwal	100.0	83.3	71.4	08
**Haryana**	91.1	65.6	74.3	246

* Services based on during last one month. ** Based on PHC functioning on 24 hours basis.

TABLE 9.8: NUMBER OF PRIMARY HEALTH CENTRES HAVING DIFFERENT ACTIVITIES BY DISTRICTS, HARYANA, 2012-13.

District	Percentage of PHCs having				Total number of PHCs
	Citizen's Charter displayed	RKS constituted	Received untied fund*	Utilized untied fund**	
Panchkula	85.7	100.0	100.0	85.7	07
Ambala	100.0	100.0	100.0	100.0	10
Yamunanagar	90.0	100.0	100.0	100.0	10
Kurukshetra	100.0	100.0	100.0	100.0	14
Kaithal	76.9	100.0	92.3	100.0	13
Karnal	100.0	94.1	100.0	100.0	17
Panipath	90.9	72.7	100.0	100.0	11
Sonipath	93.3	80.0	100.0	93.3	15
Jind	94.1	100.0	88.2	100.0	17
Fatehabad	100.0	100.0	100.0	100.0	10
Sirsa	100.0	91.7	100.0	100.0	12
Hisar	100.0	100.0	100.0	100.0	19
Bhiwani	85.7	100.0	100.0	100.0	14
Rohtak	100.0	100.0	100.0	100.0	14
Jhajjar	86.7	73.3	93.3	100.0	15
Mahendragarh	100.0	90.0	90.0	100.0	10
Rewari	66.7	100.0	88.9	100.0	09
Gurgaon	88.9	88.9	88.9	100.0	09
Faridabad	100.0	100.0	100.0	100.0	06
Mewat	16.7	100.0	100.0	100.0	06
Palwal	50.0	87.5	75.0	83.3	08
**Haryana**	89.8	93.9	96.3	98.7	246

RKS = Rogi Kalyan Samiti.* Untied fund for previous financial year ** it includes full and partial utilization of fund.

TABLE 9.9: HUMAN RESOURCES AVAILABLE AT COMMUNITY HEALTH CENTRES BY DISTRICTS, HARYANA, 2012-13.

District	Number of CHCs having				Total number of CHCs
	Obstetric Gynecologist	Pediatrician	Anesthetist	Public Health Manager	
Panchkula	02	01	01	00	02
Ambala	00	00	00	01	04
Yamunanagar	00	00	00	00	06
Kurukshetra	00	00	00	00	05
Kaithal	00	00	00	00	05
Karnal	00	00	00	01	05
Panipath	00	01	00	01	03
Sonipath	03	01	01	01	06
Jind	02	01	00	01	07
Fatehabad	00	01	01	01	04
Sirsa	01	00	03	01	07
Hisar	02	00	00	02	08
Bhiwani	00	00	00	00	09
Rohtak	01	01	01	03	05
Jhajjar	03	00	01	00	06
Mahendragarh	00	01	00	00	07
	00				
Rewari		00	00	00	05
Gurgaon	00	00	00	00	03
Faridabad	00	00	00	00	02
Mewat	00	00	00	00	03
Palwal	00	00	01	00	04
**Haryana**	14	07	09	12	106

TABLE 9.10: SPECIFIC HEALTH CARE FACILITIES AVAILABLE AT COMMUNITY HEALTH CENTRES BY DISTRICTS, HARYANA, 2012-13.

District	Number of CHCs having:				Total number of CHCs
	Functional OT	Designated as FRU	New born care services[1]	Blood storage facility	
Panchkula	01	01	02	01	02
Ambala	01	00	04	00	04
Yamunanagar	04	00	06	00	06
Kurukshetra	04	04	05	00	05
Kaithal	00	00	04	00	05
Karnal	04	05	05	01	05
Panipath	02	03	03	00	03
Sonipath	04	05	05	00	06
Jind	01	05	07	00	07
Fatehabad	04	04	04	00	04
Sirsa	03	06	06	01	07
Hisar	03	08	08	00	08
Bhiwani	06	05	09	04	09
Rohtak	04	04	05	00	05
Jhajjar	00	05	06	00	06
Mahendragarh	03	07	06	01	07
Rewari	03	04	04	00	05
Gurgaon	01	02	03	00	03
Faridabad	00	02	02	00	02
Mewat	01	03	00	00	03
Palwal	00	03	03	00	04
**Haryana**	49	76	97	08	106

OT= Operation Theatre; FRU= First Referral Unit. [1] Based on last one month services.

TABLE 9.11: NUMBER OF COMMUNITY HEALTH CENTRES HAVING DIFFERENT ACTIVITIES BY DISTRICTS, HARYANA, 2012-13.

District	Number of CHCs having: Citizen's charter displayed	RKS constituted	RKS Monitored regularly*	Utilized untied fund**	Total number of CHCs
Panchkula	02	02	02	01	02
Ambala	02	04	04	04	04
Yamunanagar	06	06	06	06	06
Kurukshetra	05	04	04	05	05
Kaithal	02	05	05	05	05
Karnal	05	05	05	05	05
Panipath	03	02	02	03	03
Sonipath	06	06	06	04	06
Jind	07	07	07	07	07
Fatehabad	04	04	04	04	04
Sirsa	07	07	07	06	07
Hisar	08	08	08	07	08
Bhiwani	09	09	09	07	09
Rohtak	05	05	05	05	05
Jhajjar	05	04	03	03	06
Mahendragarh	07	07	07	07	07
Rewari	05	05	05	05	05
Gurgaon	02	01	01	02	03
Faridabad	02	02	02	02	02
Mewat	03	03	03	03	03
Palwal	03	04	04	04	04
**Haryana**	98	100	99	95	106

* RKS monitored regularly is from number of RKS constituted. ** Including full and partial utilization.

TABLE 12: HUMAN RESOURCES & OTHER SERVICES AVAILABLE AT SUB-DIVISIONAL HOSPITALS BY DISTRICTS, HARYANA, 2012-13.

District	Number of SDHs having: Pediatrician	Radiographer	2D Echo facility	Ultrasound facility	three phase connection	critical care area	suggestion and complaint box	Total number of SDHs
Panchkula	NA	NA	NA	NA	NA	NA	NA	00
Ambala	02	00	00	00	02	00	02	02
Yamunanagar	01	00	00	00	01	00	01	01
Kurukshetra	NA	NA	NA	NA	NA	NA	NA	00
Kaithal	00	00	00	00	01	00	01	01
Karnal	01	00	00	00	02	01	02	02
Panipath	NA	NA	NA	NA	NA	NA	NA	00
Sonipath	00	00	00	00	01	01	01	01
Jind	00	00	00	00	01	00	01	01
Fatehabad	00	00	00	01	00	00	01	01
Sirsa	00	00	00	00	01	00	01	01
Hisar	00	00	00	00	02	01	02	02
Bhiwani	00	00	00	01	03	02	04	04
Rohtak	NA	NA	NA	NA	NA	NA	NA	00
Jhajjar	01	00	00	00	01	01	01	01
Mahendragarh	00	00	00	00	01	01	01	01
Rewari	00	00	00	00	01	00	01	01
Gurgaon	00	01	00	00	01	01	01	01
Faridabad	01	01	01	01	01	01	01	01
Mewat	NA	NA	NA	NA	NA	NA	NA	00
Palwal	NA	NA	NA	NA	NA	NA	NA	00
**Haryana**	06	02	01	03	19	09	21	21

NA: Not applicable

TABLE 9.13: HUMAN RESOURCES & OTHER SERVICES AVAILABLE AT DISTRICT HOSPITALS BY DISTRICTS, HARYANA, 2012-13.

District	Number of DHs having:							Total number of DHs
	Pediatrician	Radiographer	2D Echo facility	Ultrasound facility	Three phase connection	Critical care area	Suggestion and complaint box	
Panchkula	01	01	01	01	01	01	01	01
Ambala	01	00	00	01	01	01	01	01
Yamunanagar	01	00	00	00	01	00	01	01
Kurukshetra	01	01	00	01	01	00	01	01
Kaithal	01	00	00	01	01	00	01	01
Karnal	01	01	00	01	01	01	01	01
Panipath	01	00	00	01	01	01	01	01
Sonipath	01	01	00	01	01	01	01	01
Jind	01	00	00	01	01	00	01	01
Fatehabad	01	01	00	01	01	01	01	01
Sirsa	01	00	01	00	01	00	01	01
Hisar	01	00	01	01	01	01	01	01
Bhiwani	00	01	01	01	01	01	01	01
Rohtak	01	01	01	01	01	01	01	01
Jhajjar	01	01	01	01	01	01	01	01
Mahendragarh	01	00	00	01	01	01	01	01
Rewari	01	00	01	01	01	01	01	01
Gurgaon	01	00	00	01	01	01	01	01
Faridabad	01	00	01	01	01	01	01	01
Mewat	01	00	00	01	01	01	01	01
Palwal	01	00	00	01	01	01	01	01
**Haryana**	20	08	08	19	21	16	21	21

APPENDIX - A
LIST OF CONTRIBUTORS

**Dr. B Paswan**	Professor and Head, Department of Population Policy and Programme, International Institute for Population Sciences, Govandi Station Road, Deonar, Mumbai-400088.
**Dr. S. K. Singh**	Professor, Department of Mathematical Demography and Statistics, International Institute for Population Sciences, Govandi Station Road, Deonar, Mumbai-400088.
**Mr. Imran Ahmad**	Project Officer, DLHS-4, International Institute for Population Sciences, Govandi Station Road, Deonar, Mumbai-400088.
**Mr. Ashish Kumar Upadhyay**	Project Officer, DLHS-4, International Institute for Population Sciences, Govandi Station Road, Deonar, Mumbai-400088.

APPENDIX - B
FIELD AGENCY INVOLVED IN DATA COLLECTION OF DLHS-4 IN HARYANA

Academy of Management of Studies (AMS)	15, Laxmanpuri, Faizabad Road, Lucknow – 226016, Uttar Pradesh

APPENDIX - C
AGENCY DEVELOPED CAPI SOFTWARE

Tech Mahindra Limited	Satyam Infocity, Unit-12, Plot 35/36, Hi-Tech City Layout, Survey No 64, Madhapur, Hyderabad-500081.

APPENDIX - D
MEMBER OF COORDINATION COMMITTEE FOR DLHS-4

Addl. DG,	Statistics Division, Ministry of Health & Family Welfare, New Delhi, Chairman
Chief Director	Statistics Division, Ministry of Health & Family Welfare, New Delhi
Representative	Office of Registrar General
Prof. F. Ram,	Director & Senior Professor, IIPS, Mumbai
Coordinators	DLHS-4, IIPS, Mumbai
Representative	NIHFW, New Delhi
Director	Statistics Division, MoHFW, Govt. of India, New Delhi, Member Secretary

APPENDIX - E

MEMBER OF STEERING COMMITTEE OF DLHS-4

Shri K. Chandramouli,	Former Secretary (H&FW), Govt. of India, New Delhi, Chairman
Shri Naved Masood,	SS & FA, MoHFW, Govt. of India, New Delhi
Shri P. K. Pradhan,	S & MD, (NRHM), MoHFW, Govt. of India, New Delhi
Smt. Madhu Bala,	Former ADG (Stats.), MoHFW, Govt. of India, New Delhi
Shri R. C. Sethi,	Addl. RGI, Office of RGI, Govt. of India, New Delhi
Dr. Shiv Lal,	Former Spl. DG & Advisor (PH), DGHS, MoHFW, Govt. of India, New Delhi
Shri Ambrish Kumar,	Advisor (Health), Planning Commission, Govt. of India, New Delhi
Dr. Rattan Chand,	Chief Director (Stats.), MoHFW, Govt. of India, New Delhi
Prof. F. Ram,	Director & Senior Professor, IIPS, Mumbai
Prof. Arvind Pandey,	Director, NIMS, ICMR, New Delhi
Prof. Deoki Nandan,	Director, NIHFW, New Delhi
Shri Bhaskar Mishra,	Deputy RGI, Office of RGI, Govt. of India, New Delhi
Shri Pravin Srivastava,	DDG, MoHFW, Govt. of India, New Delhi
Shri V. Parameswaran,	DDG, CSO, MoS&PI, Govt. of India, New Delhi
Dr. Pavitra Mohan,	Health Specialist, UNICEF, New Delhi
Shri Shantanu Gupta,	M & E Officer, UNICEF, New Delhi
Shri K. D. Maiti,	Planning, Monitoring & Evaluation Specialist, UNICEF, New Delhi
Prof. M. M. Misro,	Professor, NIHFW, New Delhi
Prof. K. Kalaivani,	Professor, NIHFW, New Delhi
Shri Rajesh Bhatia,	Director (Stats.), MoHFW, Govt. of India, New Delhi
Shri Aditya Prakash,	Statistical Advisor MoWCD, Govt. of India, New Delhi
Dr. A. K. Harit,	CMO, DGHS, MoHFW, Govt. of India, New Delhi
Smt. Kmkum Marwah,	Joint Technical Advisor, MoWCD, Govt. of India, New Delhi
Dr. Paul Fancis,	WHO, New Delhi
Smt. Anagha Khot,	NPO, WHO, New Delhi
Dr. Subodh S. Gupta,	NPO, WHO, New Delhi
Shri Ramesh Babu,	Sr. Proframme Manager, USAID, New Delhi
Shri Sathyanaraynan,	Sr. NPO, UNFPA, New Delhi
Dr. S. C. Agrawal,	AD, MoHFW, Govt. of India, New Delhi

APPENDIX -F

MEMBER OF ADMINISTRATIVE AND FINANCIAL MANAGEMENT COMMITTEE OF DLHS-4

Addl. Secretary & Financial Advisory	Ministry of Health & Family Welfare, Chairperson
Addl. DG	Statistics Division, Ministry of Health & Family Welfare
Chief Director	Statistics Division, Ministry of Health & Family Welfare
Dy. Secretary (IFD)	Ministry of Health & Family Welfare
Director	IIPS, Mumbai
Coordinators	DLHS-4, IIPS, Mumbai
Director	Statistics Division, Ministry of Health & Family Welfare, Member Secretary

APPENDIX - G
MEMBERS OF SUB-COMMITTEE ON SAMPLING OF DLHS-4

Shri G. C. Manna,	DDG, CSO, MoSPI, New Delhi, Chairman
Dr. U. C. Sud,	Director, IASRI, New Delhi
Dr. J. P. Bhattacharjee,	DDG, SDRD, NSSO, Kolkata
Prof. L. Ladu Singh,	Professor & DLHS-4 Coordinator, IIPS, Mumbai
Shri Bhaskar Mishra,	Deputy RGI, Office of RGI, Govt. of India, New Delhi
Dr. Rattan Chand,	Chief Director (Stats), MoHFW, Govt of India, New Delhi
Shri Rajesh Bhatia,	Former Director (Stats.), MoHFW, Govt. of India, New Delhi

APPENDIX -H
MEMBERS OF TECHNICAL ADVISORY COMMITTEE (TAC) FOR DLHS-4

Dr. N. S. Shastry,	Former DG & CEO, NSSO, Govt. of India, New Delhi, Chairman
Addl. DG	Statistics Division, MoHFW, Govt. of India, New Delhi
Prof. Arvind Pandey,	Director, NIMS, ICMR, New Delhi
P. M. Kulkarni	JNU, New Delhi
DG	National Sample Survey Organisation (NSSO)
Chief Director	Statistics Division, Ministry of Health Family Welfare
DDG	Statistics Division, Ministry of Health Family Welfare
Programme Officer	Ministry of Health Family Welfare
Representative	Office of Registrar General, GOI
Representative	Ministry of Women & Child Development
Prof. F. Ram,	Director Senior Professor, IIPS, Mumbai
Coordinator	DLHS-4, IIPS, Mumbai
Representatives	World Bank, UNICEF, UNFPA, WHO, DFID and USAID
Shri Rajesh Bhatia,	Former Director (Stats.), MoHFW, Govt. of India, New Delhi, Member Secretary

APPENDIX - I
MEMBERS OF SUB-COMMITTEE TO TAC OF DLHS-4 TO EXAMINE THE DATA

Dr. N. S. Shastry,	Former DG & CEO, NSSO, Govt. of India, New Delhi, Chairman
Dr. Rattan Chand,	Chief Director (Stats.), MoHFW, Govt. of India, New Delhi
Representative	Office of Registrar General
Prof. P. M. Kulkarni	JNU, New Delhi
Prof. F. Ram,	Director & Senior Professor, IIPS, Mumbai
Director	NIHFW, New Delhi
Representative	USAID
Representative	UNFPA
Representative	UNICEF
Director	Statistics Division, MoHFW, Govt. of India, New Delhi, Member Secretary

APPENDIX - J
CAB COMPONENTS NODAL AGENCY OF DLHS-4

National Institute for Health & Family Welfare (NIHFW), Baba Gang Nath Marg, Murnika, New Delhi

APPENDIX-K

LIST OF THE PARTNER INSTITUTES FOR CAB COMPONENTS INVOLVED IN DLHS-4

Name of the Partner Institute	States
Sher-E Kashmir Institute of Medical Sciences	Kashmir and Ladakh Region
Dr. R.P. Govt. Medical College, Tanda, Himachal Pradesh	Jammu region and Himachal Pradesh
PGIMER, Chandigarh	Punjab, Chandigarh and Haryana
NIHFW, New Delhi	Delhi
NIOH, Ahmedabad	Gujarat, Daman & Diu and Dadra Nagar Haveli
NIRRH, Mumbai	Maharashtra (excluding Vidharbha) and Goa
MGIMS, Sewagram	Only Vidharbha, Maharashtra
RMRC, Dibrugarh	Sikkim and Arunachal Pradesh
RIMS, Imphal	Manipur, Mizoram & Nagaland
NEIGRIHMS, Shillong	Meghalaya
Government Medical College, Agartala	Tripura
Gandhi Medical College, Hyderabad	Andhra Pradesh & Telangana
NIE, Chennai	Tamil Nadu, Puducherry and Andaman & Nicobar Island
JN Medical Collage, Belgaum	North Karnataka
Kasturba Medical College, Manglore	South Karnataka
Thiruvananthapuram Medical College,	Kerala & Lakshadweep
NICED, Kolkata	West Bengal

APPENDIX -L

PROCUREMENT OF CAB EQUIPMENTS FOR DLHS-4

HLL Life Care Limited, B-14, A, Sector-62, Noida

APPENDIX - M

STAFF INVOLVED IN DLHS-4

International Institute for Population Sciences, Mumbai

Coordinators

Prof. F. Ram
Prof. L. Ladu Singh
Prof. B. Paswan
Prof. S. K. Singh
Prof. H. Lhungdim
Prof. T. V. Sekher
Prof. K. M. Ponnapalli
Prof. Chander Shekhar
Dr. Manoj Alagarajan

Project Coordinator

Dr. Gopal Singh Kshetrimayum
Dr. Akash N. Wankhede
Dr. G. P. Kumar

Health Coordinator

Dr. Mithilesh Verma

IT & Data Manager

Mr. Dnyaneshwar Kale
Mr. Prabhu Ponnusamy
Ms. Rojalin Behura

Project Officer (Office)

Mr. L. Priyananda Singh (IT)
Mr. Ashish Kumar Upadhyay
Mr. Imran Ahmad
Mr. Mahadev Digambar Bhise
Mr. Mukesh Ranjan
Ms. Ragini Mishra
Mr. Santosh Bhagwanrao Phad
Mr: Shrikant D. Kuntla
Ms. Arpita Paul
Mr. P.R.A. Nair
Mr. Satish Kumar Chauhan
Ms. Renu Sisodia
Ms. Kakoli Borkotoky
Mr. Ankit Anand
Mr. Satish Kumar Chauhan
Mr. Ashish Pardhi
Mr. Junaid Khan
Mr. Mohd Usman,
Mr. N. Brahmanandam
Ms. Rati Parihar
Ms. Swati Srivastava
Mr. Raj Kr. Verma
Ms. Shalini Meshram
Mr. Manish Singh
Mr. AnupamVerma
Mr. Rahul Koli
Ms. Preetam D. Gaikwad
Mr. Ajit Kumar Yadav
Mr. Prakash Chand D. Meher
Ms. Mamta Rajbhar

Project Officer (Field)

Mr. Bhagawat Singh

ADMINISTRATIVE STAFF

Project Coordinator (Adm. & Finance)

C. A. Gurudutt Belhekar

ACCOUNTANT CUM OFF. ASSISTANT

Ms. Pratidnya Kasare
Mr. Jay Kavashik Davda
Ms. Sumita Bohra
Mr. Roshan D'souza

OFFICE ASSISTANT

Ms. Namarta Thorat
Ms. Ranjita Nimbalkar
Mrs. Remya Pradeep

OFFICE ATTENDANTS

Mr. Prakash Kandera
Mr. Vishal P. Patil
Mr. Ravindra P. Gawade
Mr. Nitin M. Dekhane
Mr. Asif D. Kokane

DLHS-4

HARYANA
