# Supplementary material for: Effectiveness of Multiple-Strategy Community Intervention in Reducing Geographical, Socioeconomic and Gender Based Inequalities in Maternal and Child Health Outcomes in Haryana, India
Source: PLoS One. 2016 Mar 22;11(3):e0150537. doi: 10.1371/journal.pone.0150537 (PMC4803212; doi:10.1371/journal.pone.0150537)
Supplement: S4 File — PGIMER School of Public Health, Chandigarh. (PDF) [file pone.0150537.s008.pdf]

NHM FACTSHEET- HARYANA

Concurrent Evaluation National Health Mission, Haryana

PGIMER School of Public Health, Chandigarh

Concurrent Evaluation of National Health Mission, Haryana is a community based household survey for assessing coverage and trends of various indicators on MCH, family planning, childhood conditions, curative care services utilisation and associated expenditure. The project also evaluates trends in coverage of indicators, quality and completeness of HMIS and coverage of universal health care in the state. The project involves continuous data collection from randomly selected rural Sub-centre area/ urban area from all districts of Haryana. Data collection is performed by 30 graduate level qualified field investigators trained in data collection and survey methodology posted in each district. These investigators collect data using a pre-tested electronic tool containing six different sections for collecting responses on various indicators in the selected domains. The tool software, designed in .php format for opening in web browsers, allows investigators for simultaneous data collection and offline entry in laptops.

Results are computed for more than 65 indicators related to ANC, PNC, new born care and immunisation of infants, 10 indicators for family planning, 15 for under five year childhood conditions and 24 for curative care services. 20 indicators for assessing quality and completeness of HMIS are also generated. Results have been shared with state NHM headquarters at various platforms and with districts at RAPID rounds and other meetings. The State authorities have made use of this information to take programmatic decisions on a concurrent basis. The findings have also been used to make presentations by the state at national and international conferences/ meetings.

S. No.	Districts	No. of PSUs		No. of households	Women interviewed for ANC and PNC information	12-23 month old children studied	Eligible couples interviewed	<5 year old children studied	Individuals interviewed for cost of medical care information
		1st round	2nd round						
1.	Ambala	21	14	4846	692	627	3015	2248	2601
2.	Bhiwani	21	14	3814	665	611	2779	2162	2284
3.	Faridabad	43	27	7034	1354	1157	5349	4359	4749
4.	Fatehabad	21	14	4521	620	579	2827	2117	2497
5.	Gurgaon	35	15	6880	1078	952	4354	3222	3879
6.	Hisar	21	12	4284	588	506	2731	2013	2270
7.	Jhajjar	40	27	7168	1281	1172	5444	4089	4722
8.	Jind	22	14	4518	712	638	2970	2215	2543
9.	Kaithal	22	14	4202	693	631	2873	2268	2260
10.	Karnal	20	14	4910	641	547	2803	2240	3129
11.	Kurukshetra	21	14	5425	673	602	3241	2091	2642
12.	Mahendragarh	31	13	4405	848	763	3599	2617	2721
13.	Mewat	41	20	4769	1185	1095	4519	3808	4063
14.	Palwal	43	27	6290	1281	1199	5346	4182	4707
15.	Panchkula	19	10	3663	491	447	2285	1712	1961
16.	Panipat	34	16	5572	1012	897	4003	2937	3368
17.	Rewari	37	22	7066	1158	1042	4947	3795	4927
18.	Rohtak	22	14	3409	670	571	2456	2027	2109
19.	Sirsa	18	13	3836	543	571	2450	1773	1924
20.	Sonipat	20	14	3559	643	552	2876	2114	2621
21.	Yamunanagar	41	28	10256	1399	1309	5843	4537	5012
22.	Haryana	593	356	110427	18227	16383	76710	58526	67139

Table 1: Sampling units studied under household survey for collection of information across different districts of Haryana.

The survey considers a woman to have full ANC if she reports having undergone 3 or more ante-natal check-ups, consumed 90 or more IFA tablets and received 2 TT injections during her pregnancy. The survey results, as depicted in adjoining graph, showed 34% respondents in the state to have received full ANC, with the variation from 9% in Mewat to 62% in Kurukshetra. Figures for nine districts were below the state average, whereas Gurgaon and Panchkula, along with Kurukshetra were found to be the best performers. (Figure 1)

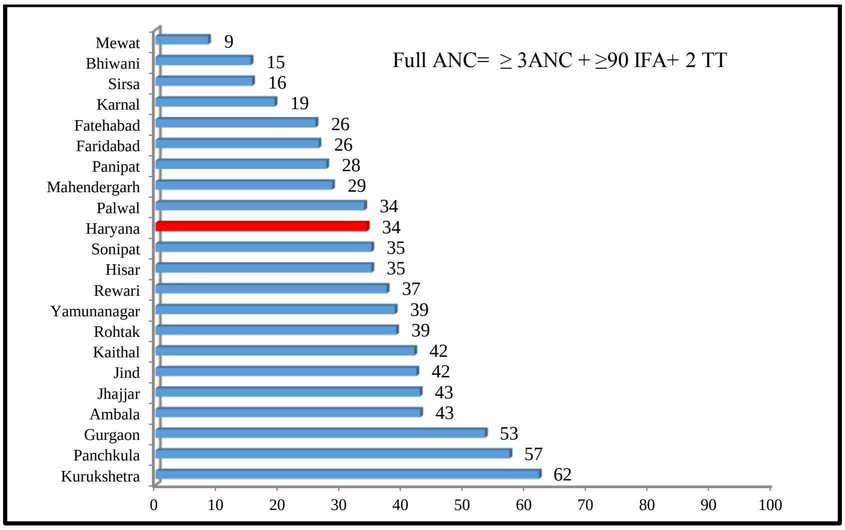

Figure 1: Full ANC rates in Haryana state (%).

The survey found 83% of respondents to have delivered their last child in healthcare facilities in the state. Highest institutional delivery rates were in Ambala district followed by Panchkula district. 54% of institutional deliveries at state level were in public sector while 29% were in private sector. The public sector deliveries were highest in Panchkula, Mahendragarh and Rohtak districts with the private sector being most active in Rewari, Ambala and Gurgaon districts. Home deliveries were highest in Mewat and Palwal districts, where almost 50% of the women reported to have delivered the child at home. (Figure 2)

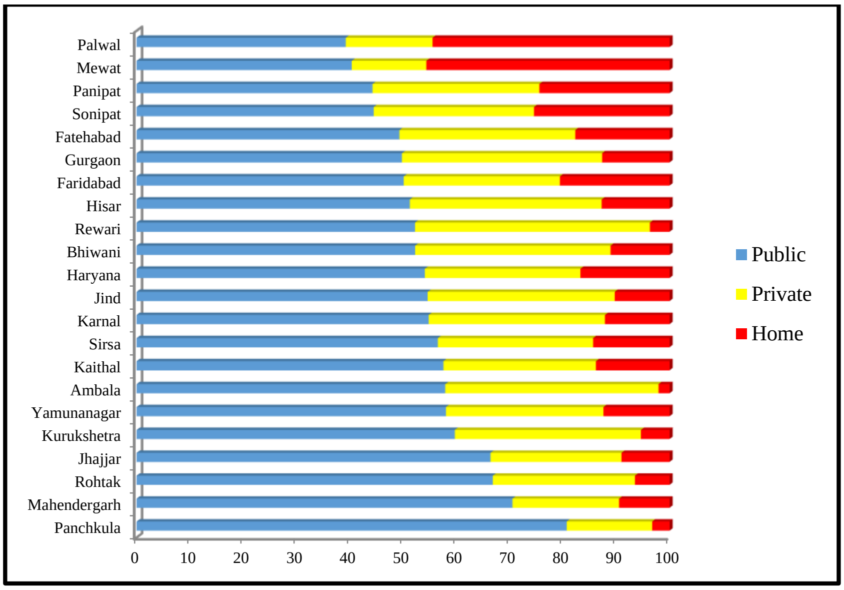

Figure 2: Institutional delivery rates in Haryana state (%).

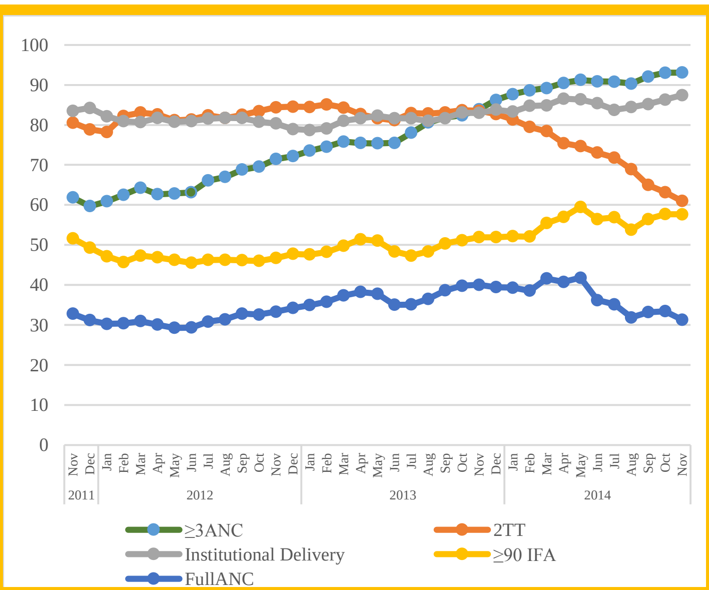

Figure 3: Time trends of ante-natal care and delivery indicators over the period of last 3 years in Haryana state (%).

Indicators	Phase 1 (2011-2012)	Phase 2 (2013-2014)	Percent change
Weight measurement during ANC	87.3	95.3	8
Height measurement during ANC	49.0	52.0	3
BP measurement during ANC	84.7	92.6	7.9
Blood test during ANC	87.6	92.7	5.1
Urine test during ANC	80.4	87.5	7.1
Abdominal examination during ANC	46.4	82.4	36
USG during ANC	80.8	89.8	9
Advice on birth preparedness	71.9	78.6	6.7
Advice on Nutrition during ANC	73.9	89.2	15.3

Table 2: Quality of ANC services delivered in 2013-14 and 2011-12 in Haryana state (%).

Figure 3 shows smoothened time trends (moving averages) in coverage of ANC and institutional delivery indicators. The months on horizontal axis depict the point of time of delivery for the cases recorded. Institutional delivery rate and consumption of ≥ 90 IFA tablets show slight improvement over time, whereas full ANC has remained more or less stagnant. Most significant changes are observed in ≥ 3 ANC, which has markedly improved over time, and receiving 2 TT injections, which shows a substantial decline initiating in last quarter of 2013 and continuing over 2014 (Figure 3).

The table 2 compares differences in coverage of quality parameters of ANC services delivered to pregnant women during 2011-12 and 2013-14. The follow up period recorded improvement in all quality parameters ranging from 3% to 36%, maximum for abdominal examination during ANC while minimum for height measurement during ANC. Excluding abdominal examination and nutritional advice during ANC, average improvement in other parameters was around 6.6%.

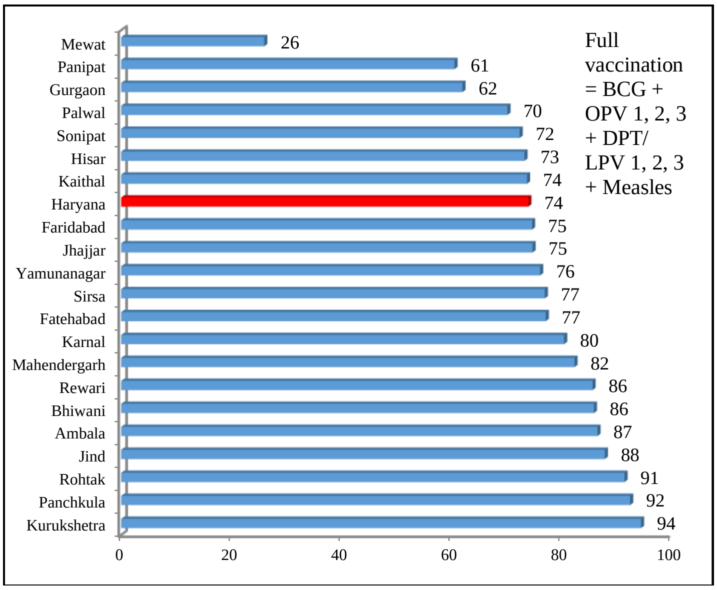

Figure 4: Full vaccination rates in 12-23 month old children in Haryana state (%).

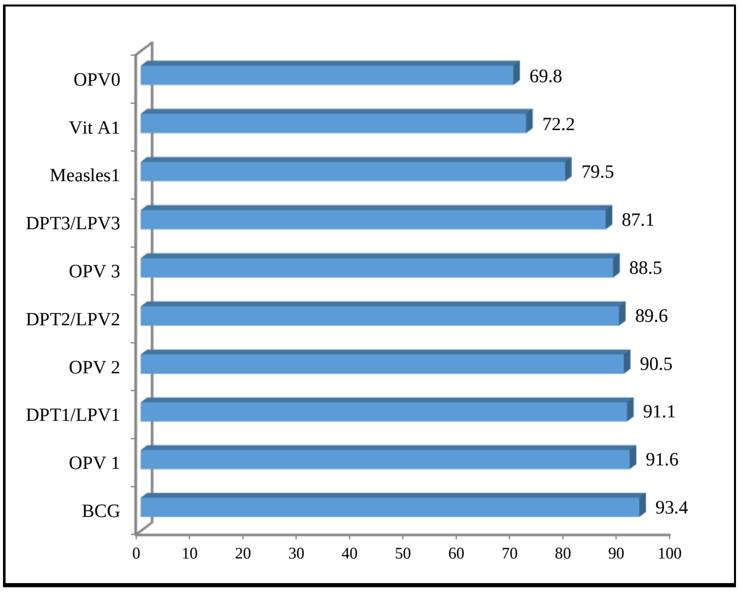

Figure 5: Individual vaccine coverage in 12-23 month old children in Haryana state (%).

Full vaccination is described as receiving a total of eight vaccines, from BCG at birth, to OPV 1, 2, 3 and DPT/ LPV 1, 2, 3 doses to Measles 1st dose at 9 months of age. The state showed 74% full vaccination rate among surveyed children, with the highest proportion being in Kurukshetra (94%) and lowest in Mewat (26%). Figures for a total of seven districts were below the state average. (Figure 4)

The household survey assessed vaccination status of 70% of 12-23 months old surveyed children from filled MCP cards available with families. For other families where cards were not available, a recall technique was used. Coverage rates for individual vaccines ranged from 70% in case of OPV 0 dose to 93% for BCG injection. There was a decline of 6% in coverage of vaccines delivered at birth to those delivered at the age of $3^1/_2$ months, followed by another decline of 7% in delivery of Measles 1 vaccine. Vitamin A1 vaccination, though done at same time as Measles 1, lagged behind by 8% points in coverage.

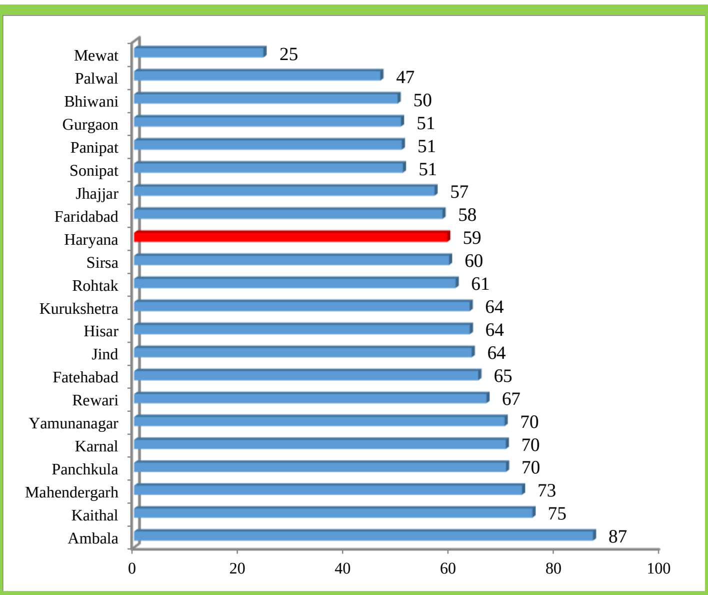

Figure 6: Contraception prevalence rate in Haryana state (%).

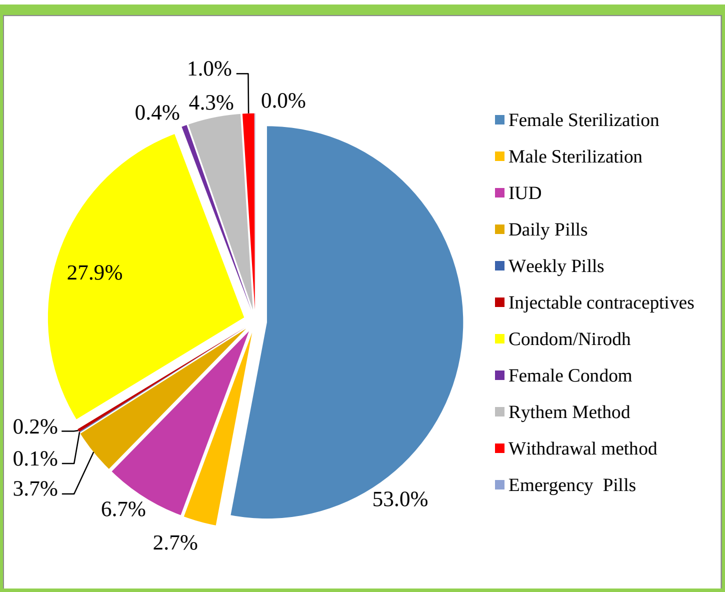

Figure 7: Proportional contraceptive utilisation in Haryana state (%).

Questions to assess contraception usage and unmet need of spacing and family planning were asked in families where eligible couples were identified. The respondents selected were either the wife or the husband. 59% of the couples reported to use any contraceptive technique at the time of survey in the state. The figures were lowest in Mewat and Palwal districts (25% and 47% respectively), while were highest in Ambala and Kaithal districts (87% and 75% respectively) (Figure 6). Along with Mewat and Palwal districts, rates for another six districts were below the state average.

More than 50% of surveyed cases utilised female sterilisation as the preferred mode of contraception. This was followed by condoms usage (27.9%), IUDs (6.7%) and daily or weekly oral pills (3.8%). Male sterilisation cases were found to be 2.7% whereas around 5% surveyed respondents relied upon traditional methods. (Figure 7)

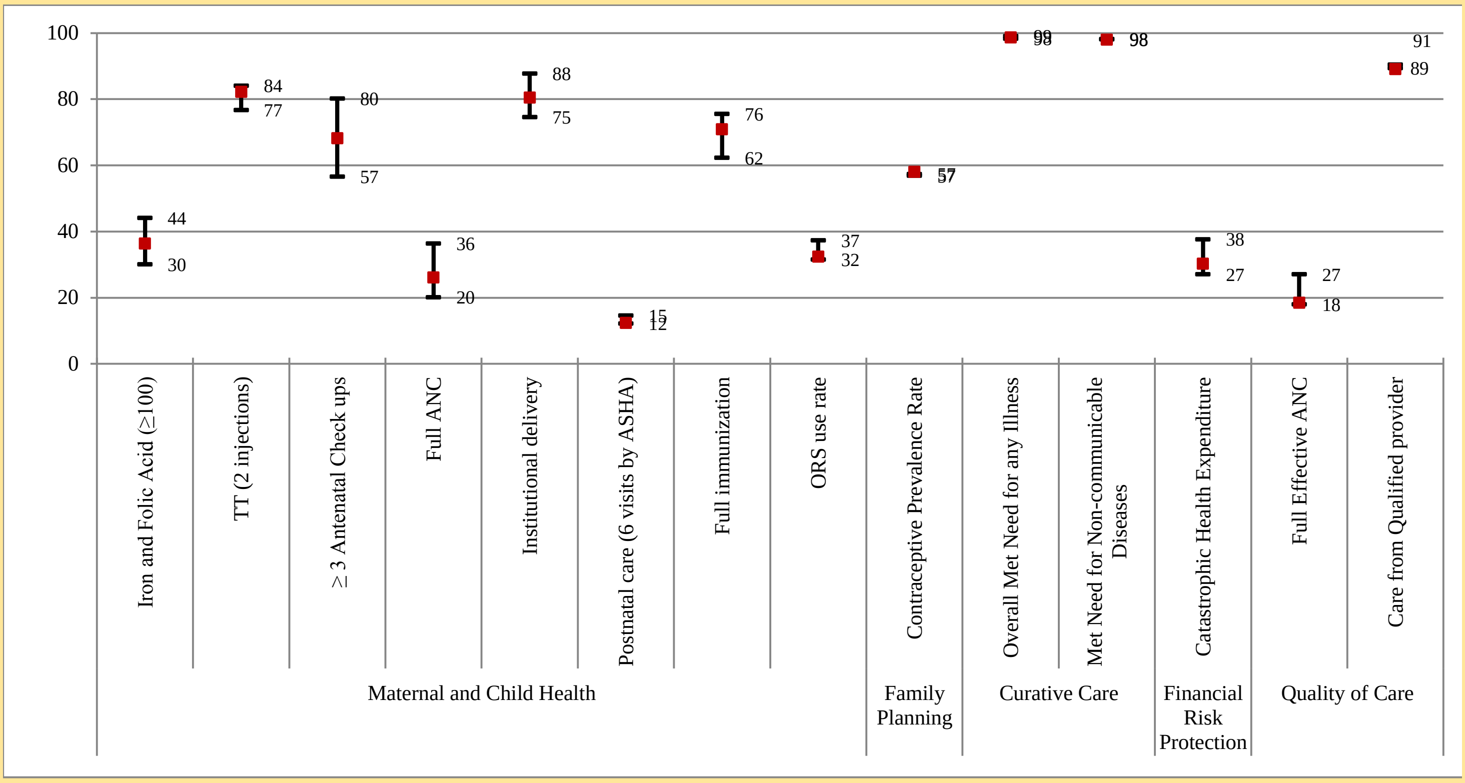

Figure 8: Wealth related inequalities in coverage of interventions across Haryana state (%).

Distinct inequalities in coverage of maternal and child health services, family planning services, curative care, financial risk protection and quality of care parameters were observed for different wealth index categories in the state. Largest gaps in coverage between richest and poorest were identified for maternal and child health indicators, such as 3 or more ante-natal check-ups, full ANC check-ups, 100 or more IFA tablet consumption, institutional delivery rate and full immunisation rate. Catastrophic health expenditure rates were also significantly different in different wealth quintiles. The need for curative care and the need for care from qualified healthcare providers were being fulfilled equally in all quintiles. (Figure 8)

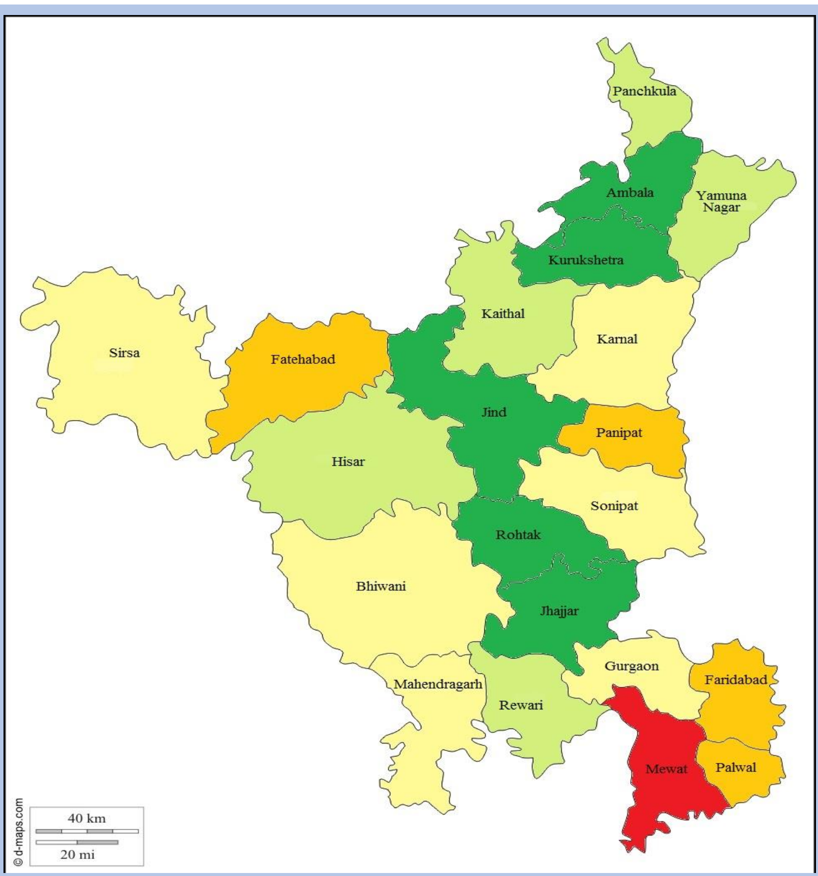

Districts and their health sub-centres in Haryana state were divided into different performance categories on basis of a composite index computed from five indicators:

1. Full ANC coverage
2. Institutional delivery rate
3. Initiation of breastfeeding within 24 hr.
4. 6 or more PNC home visits by ASHA worker
5. Full vaccination rate

Mewat district, along with Palwal, Faridabad, Panipat and Fatehabad districts were observed to be in bottom two categories. Districts of Ambala, Kurukshetra, Jind, Jhajjar and Rohtak ranked at the top of the table. (Figure 9)

Colour code	Interpretation
	Very poor performance
	Poor performance
	Average performance
	Good performance
	Very good performance

Figure 9: Geographic inequalities in coverage of maternal and child health services across Haryana state.

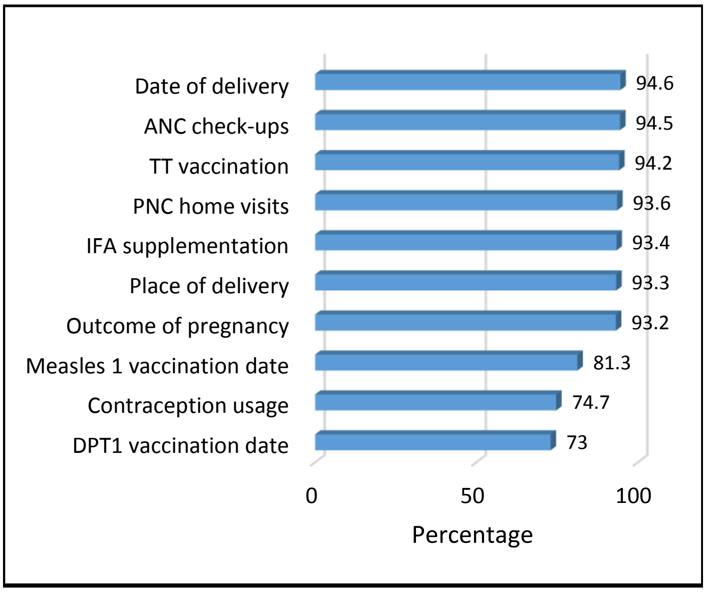

Figure 10: Completeness of sub-centre HMIS records in Haryana state (%).

Health management information system records maintained by ANMs at sub-centres were assessed for their completeness and quality. Records for found to be most incomplete in terms of information about eligible couples' contraceptive practices and children' vaccination dates. Average completeness level for other indicators was around 94%. (Figure 10)

Significant differences were observed in coverage of ante-natal and delivery indicators reported by HMIS records when compared with household survey findings for the same women. (Table 3)

Indicators	SC HMIS coverage	Household survey coverage
3 or more ANC check ups	85.0	69.0
2 or more TT injections	86.5	84.5
100 or more IFA tablets	79.8	38.1
Public sector deliveries	62.6	59.9

Table 3: Coverage of maternal and child health services in sub-centre records and household survey in Haryana state (%).

Indicators	Over-reporting
New ANC registrations	1.6
TT 2nd dose	5.3
Completion of prophylactic IFA supplementation	6.0
DPT/ LPV 1st dose	2.5
IUD insertions	5.8

Table 4: Data over-reporting in monthly performance reports over and above sub-centre records (%).

Over-reporting of coverage to an extent of 6% was observed for ante-natal care indicators on comparison of sub-centre records with submitted monthly reports. The figures were highest for coverage of IFA supplementation, 2nd dose of TT injection and IUD insertions. (Table 4)

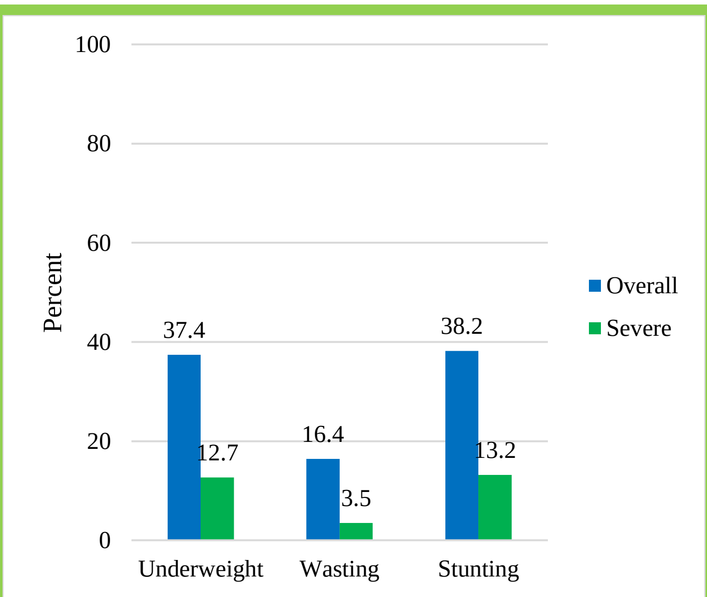

Figure 11: Status of malnutrition in under five children in Haryana state (%).

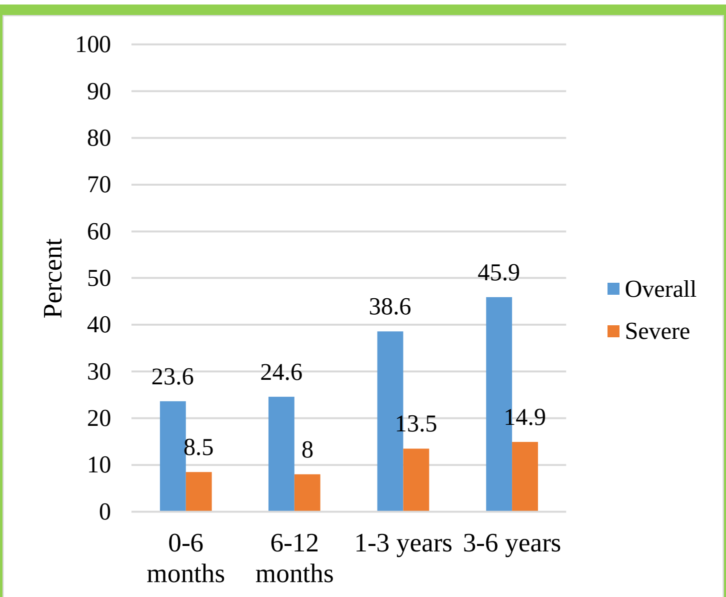

Figure 12: Age group wise prevalence of underweight in under five children in Haryana state (%).

Overall, 37.4%, 38.2% and 16.4% of under-5 year old children were underweight, stunted and wasted respectively. Nearly 12.7%, 13.2% and 3.5% of children were suffering from severe underweight, severe stunting and severe wasting respectively. (Figure 11)

Age of the child, wealth status and caste were significant determinants of malnutrition. Prevalence of under-nutrition increased as age progressed with about half (46%) of the children in the 3-6 year age group being underweight. (Figure 12)

The prevalence of underweight was more among children of slum dwellers (44.6%), illiterate parents (53.6%), scheduled castes or tribes (SC/ST) (46.3%), poorest quintile (53.2%), daily wage labourers (46.8%), those practicing open defecation (50.5%) and those mothers who consumed less than 30 iron & folic acid (IFA) tablets during pregnancy (37.3%).

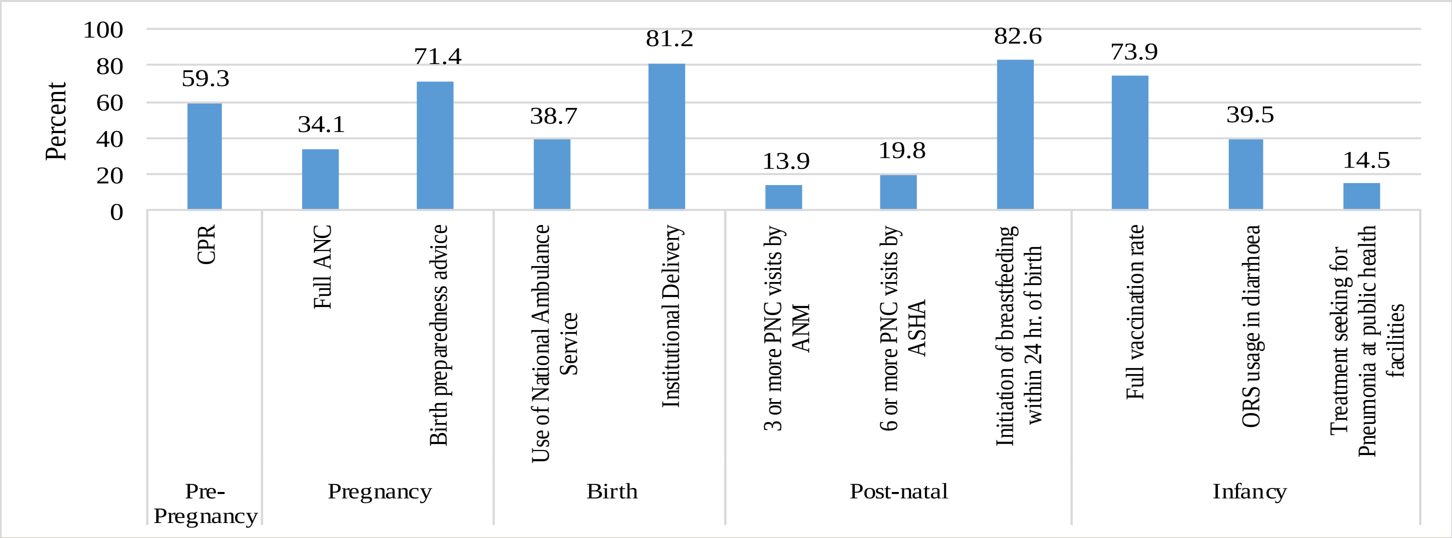

Figure 13: Coverage of interventions across the life course continuum (%).

Less than 4 in ten women receive full ANC.

In only 13% cases, mother and child received 3 or more post-natal home visits by ANM.

For more than 80% new-borns breastfeeding was initiated within 24 hours of birth.

Only 15% families approached public sector facilities for treatment of suspected pneumonia episodes suffered by their children.

Out-of-pocket expenditure	CE-NRHM (2012-15)	NSSO (2004)
Public sector deliveries	771	1080
Home deliveries	881	483
Private sector deliveries	12479	4809

Table 5: Out-of-pocket expenditure at time of delivery.

Survey results found an average expenditure of Rs. 771 per institutional delivery conducted in public health facilities, which was around 6.1% of the average amount of Rs. 12479 incurred at time of delivery in private facilities.

This also signified a reduction of Rs. 309 from NSSO findings for public sector deliveries in the state published in 2004. The expenditure in private facilities increased three fold in comparison in the same period.

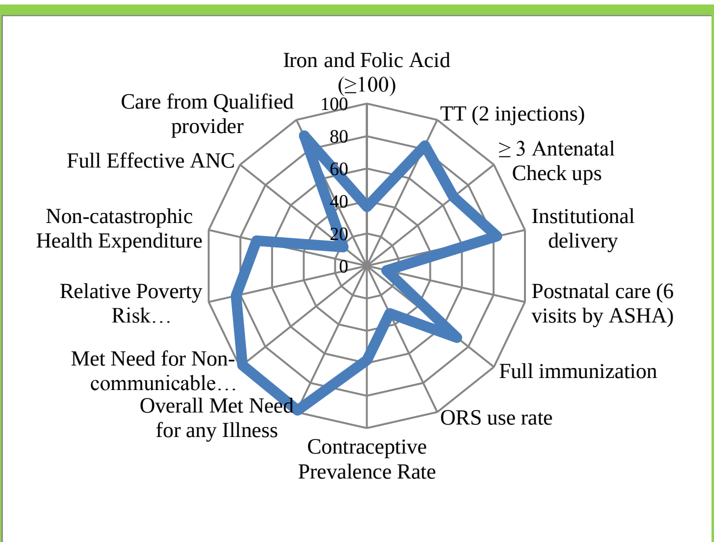

Figure 14: Universal Health Coverage in Haryana state (%).

To measure extent of universal health care at district level, indicators of service coverage, financial risk protection, equity and quality were identified as per WHO framework for UHC. (Figure 14)

Geometric mean approach was used to compute a composite UHC index (CUHCI). There were wide differences among districts in the extent of universal health care in Haryana. While districts like Kurukshetra (CUHCI= 71%) and Jhajjar (CUHCI=64%) performed well, others like Mewat (CUHCI= 12%), Palwal (CUHCI= 18%) and Fatehabad (CUHCI= 28%) were very low in terms of performance on delivery of UHC.

For more information:

Dr. Shankar Prinja
Principal Investigator, Concurrent Evaluation of NHM Haryana
Assistant Professor (Health Economics), School of Public Health
PGIMER, Chandigarh, India- 160012
0172 2755263

Dr. Amit Phogat
DD (RT, IT, M&E)
National Health Mission, Haryana
Paryatan Bhawan Building, Bays No. 55-58,
Sector 2, Panchkula, Haryana- 134109
